# Mechanistic insights into the regiodivergent insertion of bicyclo[1.1.0]butanes towards carbocycle-tethered N-heteroarenes†

**DOI:** 10.1039/d4sc08637f

**Published:** 2025-01-27

**Authors:** Johannes E. Erchinger, Madina Lenz, Poulami Mukherjee, Yan-Bo Li, Adhya Suresh, Constantin G. Daniliuc, Osvaldo Gutierrez, Frank Glorius

**Affiliations:** a Organisch-Chemisches Institut, Universität Münster Corrensstraße 36 48149 Münster Germany glorius@uni-muenster.de; b Department of Chemistry, Texas A&M University College Station Texas 77843 USA og.labs@tamu.edu

## Abstract

Ring scaffolds constitute important sub-structures in nature and across the chemical industries. However, their straight-forward introduction into a target molecule or cross-linkage between cyclic motifs of choice comprise major challenges for methodology development. Herein, the interconnection of two prominent representatives of the 2D and 3D cyclic chemical space—namely N-heteroarenes and unsaturated carbocycles—in the form of hybrid cyclobutane-tethered N-heteroarenes is targeted. The diastereoselective introduction of decorated cyclobutanes is promoted by the insertion of strained bicyclo[1.1.0]butanes (BCBs) into the C–S bond of C2–thioether aza-arenes. In-depth density functional theory (DFT) studies provide insights on the key factors governing the unexpected regiodivergent insertion outcomes. A broad scope of mono- and bicyclic aza-arenes along with mono- and disubstituted BCBs are shown to be competent. Detailed mechanistic studies support an oxidative activation of the N-heteroarenes.

## Abstract 2

Cyclobutane-tethered N-heteroarenes are obtained *via* regio- and diastereoselective insertion of BCBs into C–S bonds of C2-thioether aza-arenes. Mechanistic and DFT studies reveal the activation mode and regiodivergence.

## Graphical abstract

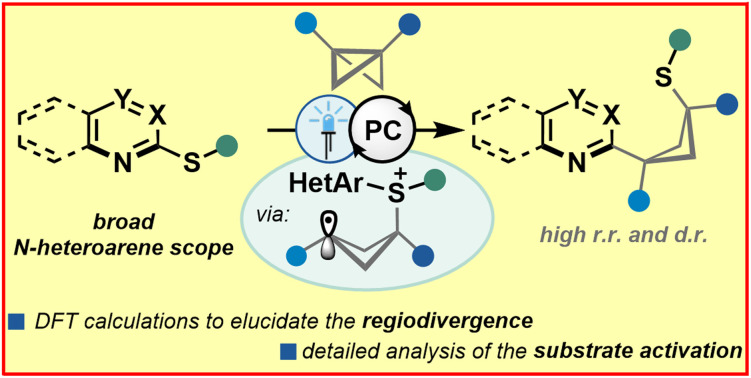

## Introduction

**Fig. 1:**
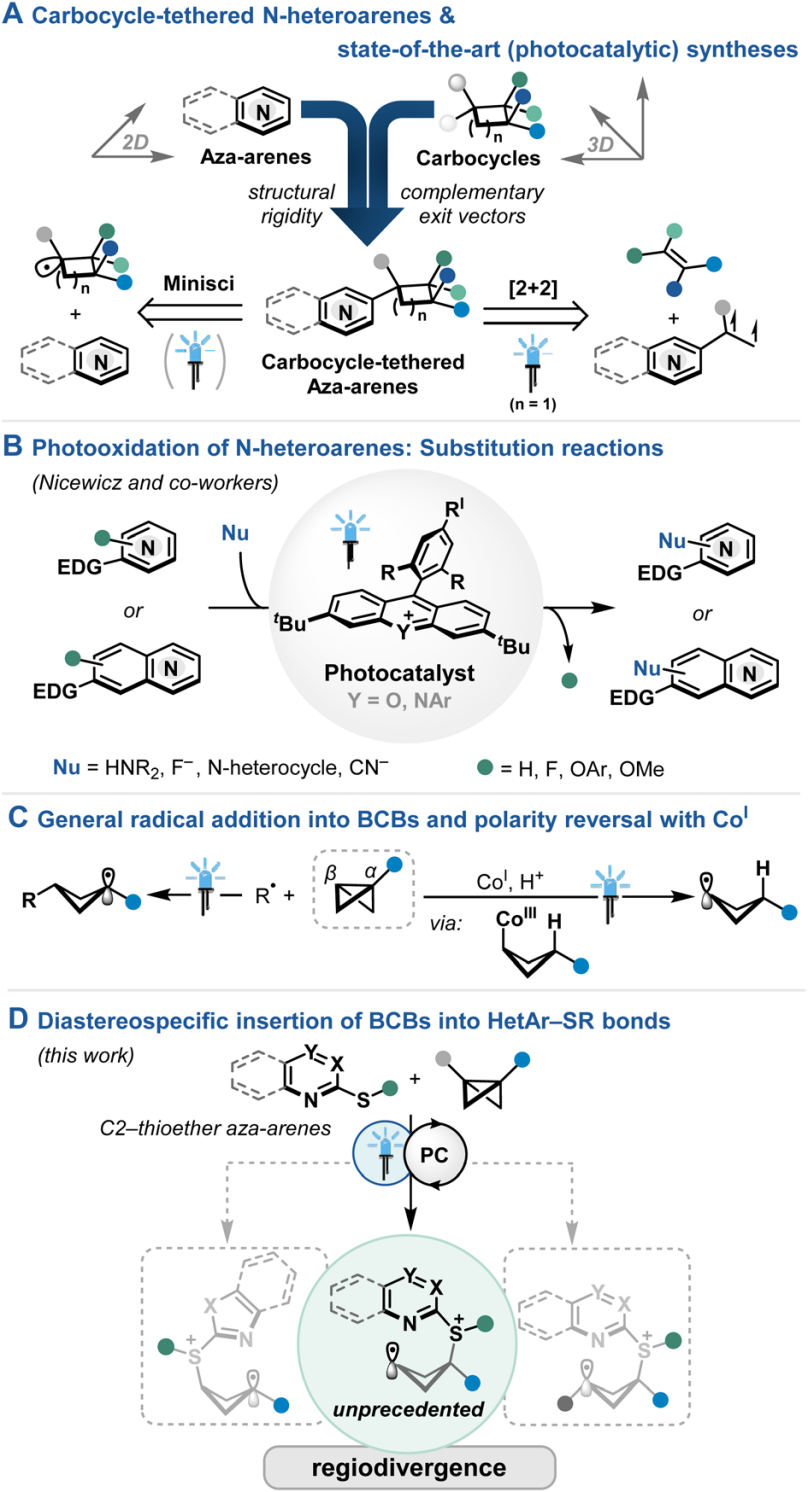
(A) Targeted hybrid product motifs and related photochemical syntheses. (B) Photoredox-catalyzed modifications of N-heteroarenes. (C) Cyclobutane radical formation through bicyclo[1.1.0]butanes. (D) This work.

Structural rigidity and well-defined exit vectors mark important features of ring structures beyond their native functionalities, distinguishing them as excellent sub-units for concise architectures, *e.g.* for ligand,^1^ organocatalyst^2,3^ or drug design.^4,5^ Notably, the careful distribution of sp^2^- and sp^3^-based ring systems are key to improve the properties of the resulting hybrid materials, including a more balanced solubility and the adjustment of binding modes.^6^ Along these lines, N-heteroarenes and saturated carbocycles comprise important cornerstones of the 2D and 3D cyclic chemical space,^4,7^ raising our interest in the amalgamation of carbocycles and aza-arenes (Fig. 1A). State-of-the-art (photochemical) strategies comprise mostly Minisci-type additions of suitable carbocyclic radical precursors, generally leading to C2- or C4-addition.^8^ While predominantly simple cyclic cores are utilized, stereoselectivity may be guided by steric repulsion.^9^

Setting the focus on cyclobutane-tethered N-heteroarenes,^10^ styrenic aza-arene precursors have been utilized in photochemical [2π+2π] cycloadditions (Fig. 1A).^11^ While elegant strategies for the stereocontrol with chiral phosphoric acid catalyst (CPA) have been pioneered by Yoon and coworkers, cooperative catalyst system^12^ or certain functional handles on the coupling partner of the aza-arene^13^ were necessary for precise substrate recognition. Towards a different direction, Aggarwal and coworkers utilized bicyclo[1.1.0]butyl boronate complexes to undergo 1,2-migration at the α-position followed by trapping with an electrophilic palladium–aryl complex at the β-carbon of the bicyclobutane upon strain-releasing C–C bond scission to deliver densely substituted cyclobutane-tethered (aza-)arenes in high regio- and diastereocontrol.^14^

Shifting the extensive photoreductive activation platform of aza-arenes^15^ towards the initial substrate oxidation, Nicewicz and coworkers successfully utilized highly oxidizing acridinium^16^ and xanthylium photocatalysts to demonstrate a diverse set of substitution reactions,^17,18^ including oxidative C–H functionalizations (Fig. 1B).^19^ While generally the addition of nucleophile to the (hetero-)aromatic radical cation has been proposed, intermediate insertion or extrusion pathways remain rare and lead to similar substitution products.^20^ Rather using pyridine boryl radical as catalysis platform, Wang and coworkers reported the formal cycloaddition of BCBs with alkenes^21^ or vinyl azides^22^ while Li and coworkers activated the pyridine motif attached to the BCB to facilitate cycloaddition reactions with alkenes and alkynes.^23^

Radical addition into monosubstituted BCBs for the formation of higher stabilized radical at the more substituted α-carbon is prevalent in literature (Fig. 1C).^24^ On the other hand, radical attack at the α-carbon is, to the best of our knowledge, unprecedented, while the formation of C-centered radicals in β-position is rare. In this context, Gryko and coworkers utilized a polarity-reversal strategy by a light-driven cleavage of a cobalt(iii) intermediate upon hydrometallation of the BCB (Fig. 1C).^25^ The resulting radicals at the β-carbon could then participate in Giese-type additions and Ni-catalyzed cross-couplings.

## Results and discussion

### Reaction development and screening results

**Fig. 2:**
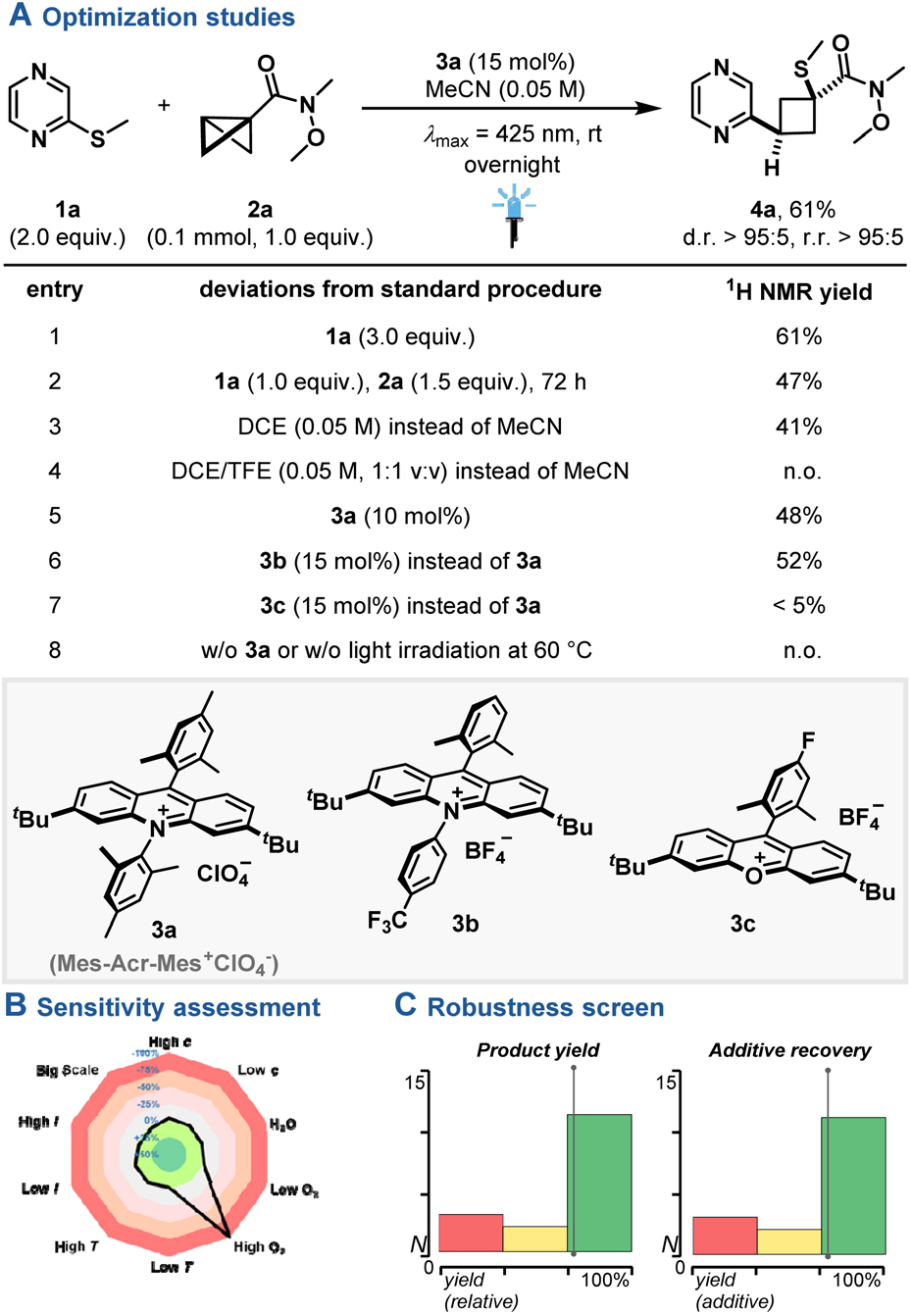
(A) Optimization studies. (B) Sensitivity assessment. (C) Robustness screen. Product yield for the optimization studies was determined by ^1^H NMR yield using CH_2_Br_2_ as internal standard.

Enlarging the synthetic plethora of N-heterocycle photooxidation towards insertion strategies,^26,27^ we commenced on a reaction setup with commercial pyrazine 1a, BCB 2a and acridinium photocatalyst 3a under blue light irradiation (*λ*_max_ = 425 nm) to yield insertion product 4a in 61% yield and excellent regio- and diastereoselectivity (Fig. 2A). To our surprise, the regioisomer, which presumably formed upon attack of the radical cation at the α-carbon of 2a, was obtained. Similar performance was observed under higher N-heterocycle loadings (entry 1), while lower reactivity was observed when using BCB 2a in excess despite prolonged reaction times (entry 2). Screening of plausible solvent systems, lower catalyst loadings or other highly oxidizing acridinium or xanthylium photocatalysts^17^ yielded the product in reduced yield (entry 3–7). Control reactions showcased that irradiation in the presence of the photocatalyst is vital for the observed reactivity (entry 8), while less oxidizing photocatalysts did not yield any insertion product (see ESI†). No reactivity was observed when activated alkenes instead of 2a were employed under standard reaction conditions, underpinning the importance of the strain-releasing element (see ESI, Fig. S3†).^28^ A reaction parameter-based sensitivity screen^29^ showcased the suitability of water addition to the standard reaction, while reactivity was shut down at high oxygen levels (Fig. 2B). Finally, an additive-based robustness screen^30^ was applied, evaluating both the relative product yield (yield with the additive compared to without) and the recovery of the additive. The screen indicated a good functional group tolerance towards various functional groups (Fig. 2C), such as alkynes, alkenes, alcohols, amides, aryl halides, ketones and ethers (see ESI, Table S2 and Fig. S7†).

### Computational studies for the formation of 4a

**Fig. 3:**
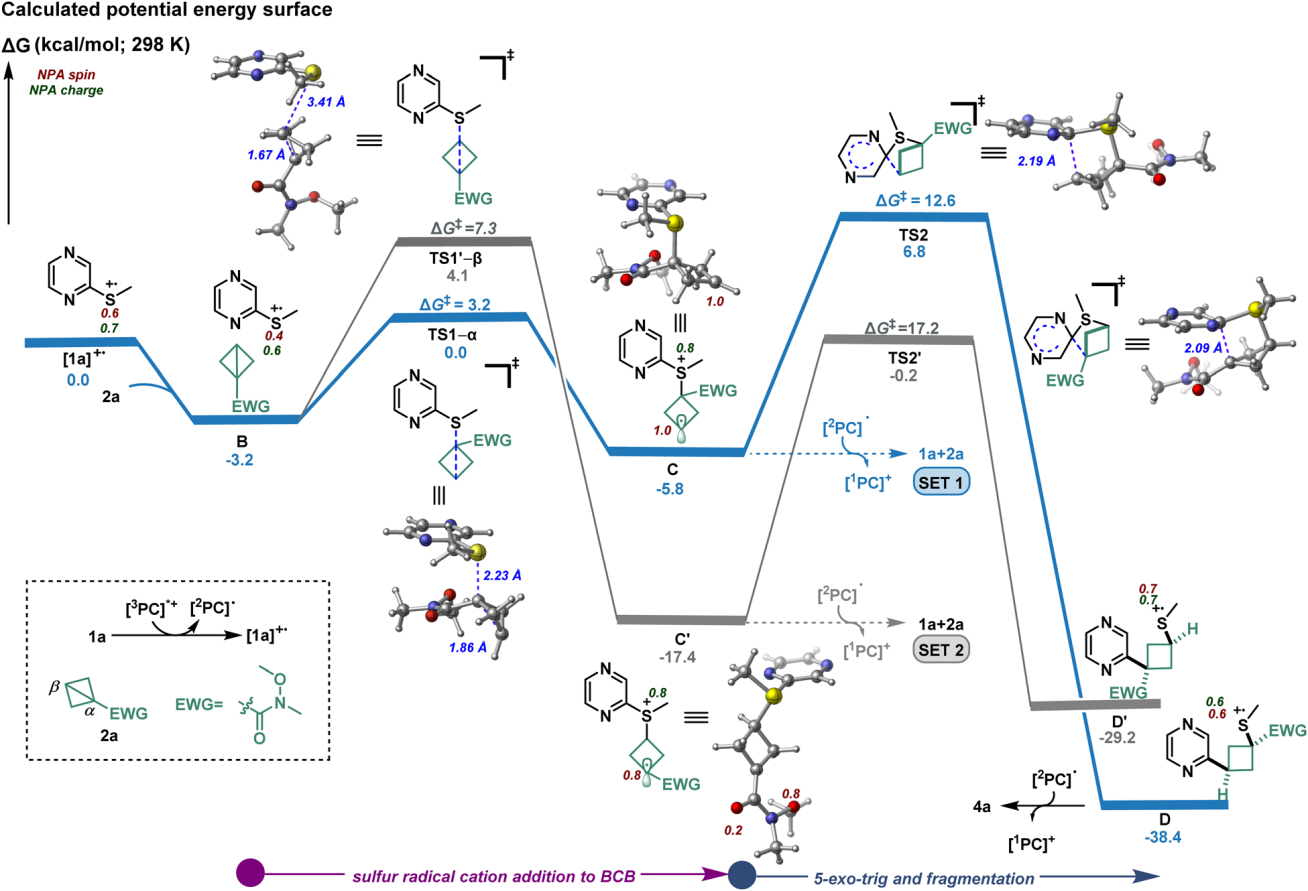
Proposed mechanism supported by computational studies. Calculated energies [uB3LYP-D3/def2-svp-CPCM(ACN)] are given in kcal mol^−1^. For details, see ESI.†

To gain a deeper insight into the origin of the unprecedented regioselectivity and high diastereoselectivity, we first turned to dispersion-corrected density functional theory (DFT) calculations (see ESI† for additional details). As shown in Fig. 3, the first step of the mechanism comprises the formation of radical cation [1a]^+·^ upon oxidation of 1a in presence of the photocatalyst.^26,27^ Natural population analysis (NPA)^31^ of [1a]^+·^ revealed significant spin density (shown as red, Fig. 3) located at the sulfur atom which, in turn, promotes a reversible and regioselective radical ring opening of the BCB *via* attack at the α-carbon atom with a low barrier transition state (TS1–α; 3.2 kcal mol^−1^ with respect to complex intermediate B) leading to radical cation intermediate C (downhill by 5.8 kcal mol^−1^). Then, C may perform an irreversible 5-*exo*-trig type addition/fragmentation (Δ*G*^‡^ = 12.6 kcal mol^−1^) which offers the thermodynamically more stable radical cationic intermediate D, downhill in energy by 38.4 kcal mol^−1^. Our calculation shows that the *syn*-diastereoselective introduction on the cyclobutane is governed by the conformation adopted in TS2 during cyclization. Finally, the [^2^PC]^·^ reduces D to deliver product 4a as the major regioisomer. Alternatively, addition to the β-carbon atom *via* a high energy transition state TS1′-β (Δ*G*^‡^ = 7.3 kcal mol^−1^) could lead to the irreversible formation of radical cationic intermediate C′ (downhill by 17.4 kcal mol^−1^). However, the significant high energy barrier for cyclization/C–S bond scission (Δ*G*^‡^ = 17.2 kcal mol^−1^, with respect to C′) to form the minor regioisomeric radical cationic intermediate D′ is consistent with the experimental observation. Specifically, these results indicate that since the barrier for C′ to undergo cyclization/fragmentation is higher, C′ is more prone to undergo SET to revert back to the starting material (1a + 2a) (see ESI, Fig. S23†). As such, the origin of the regioselectivity is the ability of C to undergo a facile 5-*exo*-trig type addition/fragmentation while C′, due to a higher barrier for cyclization, is more prone to interact with photocatalyst to undergo reduction/fragmentation to the reactants. Consequently, despite the reversible formation of C, concentration of C replenishes over the time as the major product forms which should drive the reaction forward to form the major product. Overall, the interplay between thermodynamics, kinetics, and electron transfer processes is considered to contribute to the final product distribution and regioselectivity.

### N-Heteroarene scope studies

**Fig. 4:**
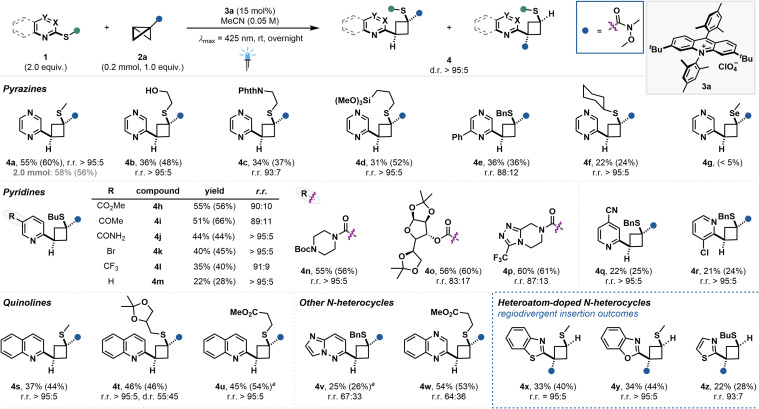
N-Heteroarene scope and regiodivergent product formation. Isolated yields are given. ^1^H NMR yields of the crude reaction mixture are given in parentheses using CH_2_Br_2_ as internal standard. ^*a*^Reaction was performed on a 0.1 mmol scale.

We then set out to investigate the N-heterocycle scope (Fig. 4). Notably, most of the shown substrates could be readily prepared from the respective commercial C2-halogenated aza-arenes by S_N_Ar reaction with the corresponding thiol (see ESI†). Initial studies highlighted the crucial role of the thioether at the C2–position relative to the heteroarene nitrogen, ruling out the use of simple arene substrates (see ESI, Fig. S4†). Scale-up of the photocatalytic reaction of model substrate 1a to 0.2 mmol and 2.0 mmol scale was well tolerated, yielding 3a in 55% and 58% isolated yield, respectively. Other cyclobutane-tethered pyrazines were prepared in moderate yield, yet excellent regio- and diastereoselectivities bearing free alcohol (4b), phthalimide (4c), silyloxy (4d) substituents on the thioether or aromatic substitution on the aza-arene (4e). Notably, the introduction of steric bulk appears to be detrimental for the reactivity, as shown for cyclohexyl substituted substrate 4f (see ESI, Fig. S4†). Surprisingly, reactivity for the selenide derivative was also drastically reduced, observing only traces of 4g. Pyridines with C2–thioether moiety could also yield the insertion products 4h–p with good to excellent regio- and diastereoselectivities, highlighting the functional group tolerance towards various carbonyl moieties—including sensitive free amide moiety (4j)—and halogenated compounds. Notably, electron-withdrawing groups had a positive influence on the reaction yield when compared to mono-substituted pyridine (4m). Utilization of other substitution patterns on the pyridine led to product formation in lower yield (4q and 4r). Quinolines bearing thiomethyl group (4s), acetal (4t) and ester (4u) moiety could be isolated in moderate yields and excellent regio- and diastereoselectivities with regard BCB insertion. However, for product 4t no diastereocontrol over the distal stereocenter on the thioether substituent could be obtained. Other N-heteroarenes like the imidazopyridazine and quinoxazoline derivatives yielded the cyclobutane-tethered aza-arenes 4v and 4w, however a significant decrease in the regioisomeric ratio was observed. Insertion into C2-thioether pyrimidine, isoquinoline, quinazoline, pyridazine and pyrazole derivatives was not successful (see ESI, Fig. S4†). Finally, heteroatom-doped N-heteroarenes bearing a five-membered ring adjacent to the insertion center such as (benzo-)thiazole and benzoxazole thioethers were successfully employed, yielding products 4x–z in moderate yields, yet excellent diastereo- and regioselectivities. The observed regioselectivity was inverted compared to the other N-heterocycle entries, presumably originating from radical cation attack at the β-carbon.^26,27^ While in cases of low product yields the amount of remaining N-heteroarene substrate was largely unaffected, the low efficiency was likely due to the unproductive depletion of the BCB during the reaction.

### Computational studies for the formation of 4x

Hence, DFT studies for the reaction of 2-(methylthio)benzothiazole (1x) with 2a to form 4x were conducted (Fig. S25†). Notably, the DFT calculations are well in line with the experimental results, indicating the first addition of [1x]^+·^ to 2a to be crucial for the determination of the regioselectivity. It was further revealed that the addition in β-position is largely governed by the irreversible formation of tertiary radical intermediate along with the lower steric repulsion between the electron-withdrawing group and benzothiazole motif (see ESI† for further information).

### Bicyclo[1.1.0]butane scope studies

**Fig. 5:**
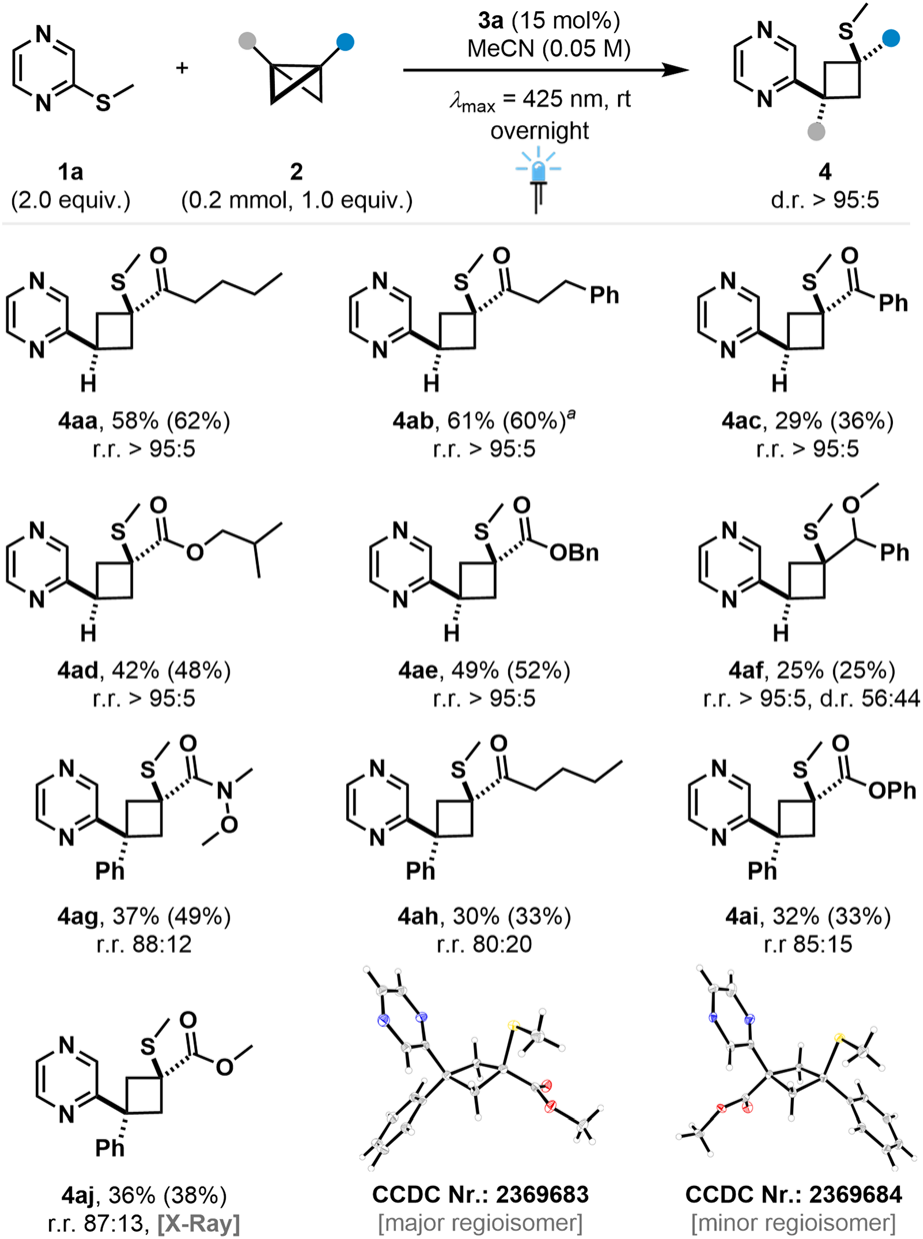
Bicyclo[1.1.0]butane scope. Isolated yields are given. ^1^H NMR yields of the crude reaction mixture are given in parentheses using CH_2_Br_2_ as internal standard. ^*a*^Reaction was performed on a 0.1 mmol scale.

We then turned towards the bicyclo[1.1.0]butane scope, which displayed good compatibility of ketone- and ester substituted BCBs to yield products 4aa–ae in moderate to good yield and excellent diastereo- and regioselectivities (Fig. 5). Aromatic substitution on the carbonyl led to reduced yield (4ac), presumably due to a higher steric bulk. Reaction of more electron-rich ether-bearing BCB led to product 4af in excellent regioselectivity and synthetically useful yield. While the BCB insertion into the C–S bond is diastereoselective, the peripheral stereocenter of the parent BCB starting material remains uncontrolled, resulting in a low diastereoselectivity with regard to the benzylic position. Surprisingly, amide substituted BCBs led to cyclobutane-tethered pyrazines in low yield, likely due to steric factors (see ESI†). Furthermore, disubstituted BCBs were successfully employed, yielding the insertion products in excellent diastereo- and good regioisomeric ratios (4ag–aj). For entry 4aj, crystal structures of both the major and minor regioisomer could be obtained. Despite the expected higher stabilization of the intermediate benzylic radical,^27,32^ the major regioisomer is not inverted compared to the incorporation of mono-substituted BCBs (Fig. 5). This suggests that different parameters govern the regioselectivity for the reaction of 1a with disubstituted BCBs, which we further analyzed in detail (*vide infra*).

### Product diversification

**Fig. 6:**
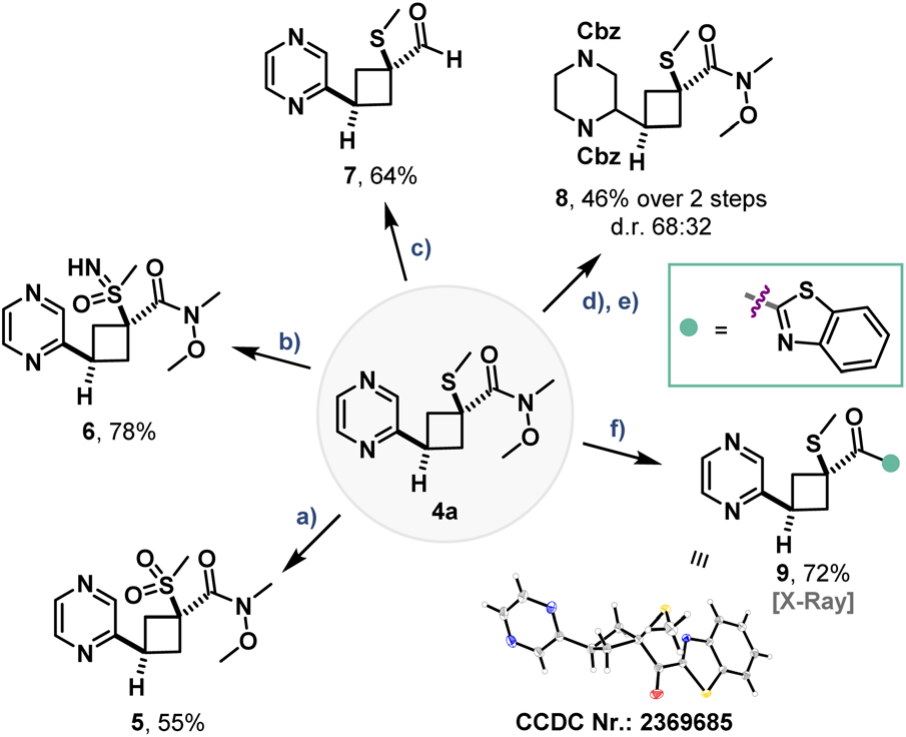
Diversification reactions of 4a. (a) *m*-CPBA (4.0 equiv.), DCM (0.1 M), 0 °C, 2 h. (b) PIDA (2.5 equiv.), H_2_NCO_2_^−^ NH_4_^+^ (2.0 equiv.), MeOH (0.25 M), rt, 3 h. (c) LiAlH_4_ (2.0 equiv.), THF (0.1 M), 0 °C, 3 h. (d) PtO_2_ (10 mol%), H_2_ (50 bar), AcOH (0.1 M), 50 °C, overnight. (e) CbzCl (3.2 equiv.), dioxane : H_2_O = 3 : 2 (v/v), aq. NaOH (50%), rt, 12 h. (f) Benzothiazol (1.73 equiv.), *n*-BuLi (1.6 equiv.), THF (0.2 M), −78 °C, 80 min; then 4a (1.0 equiv.) in THF (0.6 M), −78 °C to rt, 45 min.

Insertion product 4a was successfully employed in postfunctionalization reactions including redox modifications to yield sulfone 5, sulfoximine 6 or aldehyde 7 (Fig. 6). Full hydrogenation of the pyrazine moiety and Cbz-protection yielded piperazine 8, while addition to lithiated benzothiazole yielded ketone 9. Notably, the crystal structure of 9 corroborates the assigned regio- and diastereoselectivity of the insertion product originating from mono-substituted BCBs with C2–thioether pyrazines.

### Mechanistic investigation

**Fig. 7:**
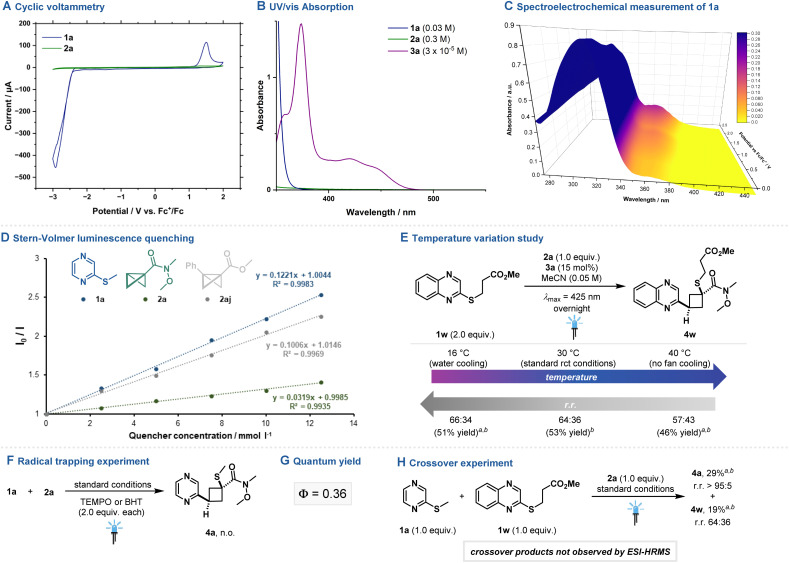
Mechanistic experiments. (A) Cyclic voltammogram. (B) UV/vis absorption spectroscopy. (C) Spectroelectrochemical measurement. (D) Stern–Volmer luminescence quenching studies. (E) Temperature variation study. (F) Radical trapping experiment. (G) Quantum yield. (H) Crossover experiment. See ESI† for further information. ^1^H NMR yields of the crude reaction mixture are given in parentheses using CH_2_Br_2_ as internal standard. ^*a*^Reaction was performed on a 0.1 mmol scale. ^*b*^The diastereomeric ratio was determined to be d.r. >95 : 5.

To gain better insights into the complex mechanisms of action and corroborate the findings from the DFT analysis, first cyclic voltammetry studies of 1a and 2a were performed (Fig. 7), only showing an irreversible oxidation of 1a at *E*_Ox_ = +1.89 V *vs.* SCE. In a previous study by our group, the oxidation potential of 2aj was determined to be *E*_Ox_ = +1.79 V *vs.* Ag/AgCl.^33^ Hence, oxidation of N-heteroarenes or disubstituted BCBs with 3a* (*E*_1/2_ [PC^+^*]/[PC^·^] = +2.00 V *vs.* SCE)^34^ should be thermodynamically feasible. UV/vis absorption studies revealed only the photocatalyst to absorb light within the employed wavelength region (*λ*_max_ = 425 nm). Furthermore, spectroelectrochemical measurement of 1a displayed a new absorption band at approx. 360 nm when applying potentials exceeding the previously determined oxidation potential of 1a, implying the intermediacy of the respective radical cationic species. Stern–Volmer luminescence quenching demonstrated quenching of both the N-heteroarene and BCB equivalent. While quenching rates of 1a were approx. four times higher compared to 2a, disubstituted BCB 4aj and 1a exhibited similar quenching rates, indicating a more competitive quenching behavior of 3a* in this reaction scenario.^35^ Next, the influence of the reaction temperature on the regioisomeric ratio of product 4w was studied, displaying higher r.r. for lowering the reaction temperature and *vice versa*. Addition of TEMPO or BHT to the standard reaction set-up halted the product formation, but no adducts with the trapping agents could be observed. The quantum yield of the reaction was determined to be *Φ* = 0.36. The reaction of two C2–thioether aza-arenes with 2a did not yield any crossover product (see ESI† for further details), corroborating a concerted mechanism for C–S σ-bond scission and product formation. Furthermore, trapping of a plausible N-heterocyclic carbocation intermediate with various nucleophiles was attempted, however no addition or substitution product could be observed. No product formation was observed by replacing 3a with stoichiometric amounts of exogenous oxidants under thermal conditions (see ESI†).

### Computational studies for the formation of 4aj

As based on the Stern–Volmer quenching studies the oxidation of aza-arenes or disubstituted BCBs^33,35^ should be plausible reaction initiation pathways to form 4aj and related products, DFT calculations for both routes were conducted. These support the addition of [1a]^+·^ to the neutral disubstituted BCBs, while the α-addition was found to be favored over the β-addition largely due to the formation of stabilized benzylic radical intermediate and lower distortion energy (see ESI for further details, Fig. S26–S28†).

## Conclusion

In summary, we provide a proof-of-concept that photooxidation of aza-arene derivatives may be a suitable strategy for insertion reactions by the strategic use of strain-release, thereby representing a non-canonical pathway towards Minisci-type products. A broad range of N-heteroarenes, alongside mono- and disubstituted BCBs, were successfully employed, achieving highly *syn*-diastereoselective and regiodivergent reaction outcomes depending on the nature of the substrates involved. The insertion products serve as versatile carbocycle-tethered N-heterocycle building blocks, as showcased by redox modifications, hydrogenation and nucleophilic addition. Mechanistic experiments provided insights into substrate activation, while in-depth DFT studies clarified the high *syn*-diastereoselectivity and divergent regioselectivities across substrate classes, indicating that the addition of heteroaromatic S-centered radical cation to the BCB framework determines the final regioisomeric outcome. Given the wide range of photoredox activation modes, we hope that this work will inspire further interest in the field of selective N-heterocycle modification by visible light photocatalysis.

## Data availability

Experimental and characterization data, including crystallo-graphic data [4aj major regioisomer (CCDC Nr.: 2369683), 4aj minor regioisomer (CCDC Nr.: 2369684), and 9 (CCDC Nr.: 2369685)], and NMR spectra, as well as mechanistic investigations and computational investigations. The data supporting this article have been included as part of the ESI.†

## Conflicts of interest

There are no conflicts to declare.

## Supplementary Material

SC-016-D4SC08637F-s001

SC-016-D4SC08637F-s002
