# Supplementary material for: Mechanistic insights into the regiodivergent insertion of bicyclo[1.1.0]butanes towards carbocycle-tethered N-heteroarenes
Source: Chem Sci. 2025 Jan 27;16(9):4006–13. doi: 10.1039/d4sc08637f (PMC11789309; doi:10.1039/d4sc08637f)
Supplement: SC-016-D4SC08637F-s001 [file SC-016-D4SC08637F-s001.pdf]

Supplementary Information (SI) for Chemical Science.
This journal is © The Royal Society of Chemistry 2025

Supporting Information

Mechanistic insights into the regiodivergent insertion of bicyclo[1.1.0]butanes towards carbocycle-tethered N-heteroarenes

Johannes E. Erchinger,[‡a] Madina Lenz,[‡a] Poulami Mukherjee,[b] Yan-Bo Li,[a] Adhya Suresh,[b] Constantin G. Daniliuc,[a] Osvaldo Gutierrez,[*b] and Frank Glorius[*a]

† *Authors contributed equally.*

a Organisch-Chemisches Institut, Universität Münster, Corrensstrasse 36, 48149 Münster (Germany).

b Department of Chemistry Texas A&M University, College Station, Texas 77843, USA.

*E-Mail: og.labs@tamu.edu; glorius@uni-muenster.de.

Table of contents

1 General information ... 4

2 Experimental procedures and characterization data ... 6

2.1 Starting material synthesis ... 6

2.1.1 Synthesis of thioether-containing aza-arenes ... 6

2.1.2 Synthesis of bicyclobutanes (BCBs) ... 16

2.2 General procedure (GP-1) for the catalytic reaction of aza-arenes with BCBs ... 16

2.3 Optimization studies and control experiments ... 17

2.4 Synthesis of insertion products ... 18

2.4.1 N-Heteroarene scope ... 18

2.4.2 Bicyclobutane scope ... 30

2.5 Scale-up experiment ... 35

2.6 Product diversification ... 35

2.7 Scope Limitations ... 38

2.7.1 (Hetero-)arene scope limitations – A practical guide ... 38

2.7.2 BCB scope limitations ... 39

2.8 Screening ... 39

2.8.1 Sensitivity assessment ... 39

2.8.2 Additive-based robustness screen ... 40

2.9 Crystallographic data ... 43

3 Mechanistic Analysis ... 47

3.1 Cyclic voltammetry studies ... 47

3.2 UV/vis absorption spectroscopy ... 48

3.3 Spectroelectrochemical measurement ... 49

3.4 Stern–Volmer quenching studies ... 50

3.5 Trapping experiments ... 51

3.5.1 Radical trapping experiments ... 51

3.5.2 Trapping with nucleophiles ... 51

3.6 Quantum yield measurement ... 52

3.6.1 Determination of the photon flux ... 52

3.6.2 Determination of the quantum yield ... 54

3.7 Evaluation of exogenous oxidizing agents ... 55

3.8 Crossover experiment ... 56

3.9 Temperature variation study ... 57

4 Computational studies ... 58

4.1 Proposed mechanism with pyrazine 1a ... 58

4.2 Proposed mechanism with benzothiazole 1x ... 61

4.2.1 Activation strain/ distortion analysis for TS3–β and TS3'–α.. 61
4.3 Proposed mechanism with disubstituted BCB 2ag 63
4.4 Coordinates 65
5 NMR spectra 148
6 References... 205

1 General information

Unless otherwise stated, all reactions were performed under a positive atmosphere of argon in oven-dried or flame-dried glassware. Prior to set-up of the reaction, glassware was evacuated and backfilled with argon three times. The solvents dichloromethane (CH_2Cl_2), methanol (MeOH), toluene, tetrahydrofuran (THF), *N,N*-dimethylformamide (*N,N*-DMF), diethyl ether (Et_2O), hexane and acetonitrile (MeCN) applied in synthesis were purified by a solvent purification system (SPS) over standard drying materials with positive argon flow and stored under argon. Pyridine and triethylamine (NEt_3) were stored over molecular sieves under argon.

Photochemical set-up and Light sources Photochemical reactions were performed in a Hepatochem EvoluChem^{TM} PhotoRedOx Box Duo device and irradiated with two EvoluChem™ HCK1012-01-012 LEDs (18 W, λ_{max} = 425 nm). To shield irradiation, the setup was covered with a small cardboard box. The reaction temperature was determined to be between 30 °C and 33 °C with the ventilation switched on, using this setup.

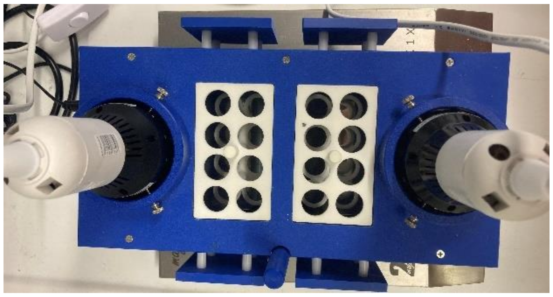

Figure S1. Hepatochem EvoluChem^{TM} PhotoRedOx Box Duo device with two EvoluChem™ LED spotlights (λ_{max} = 425nm).

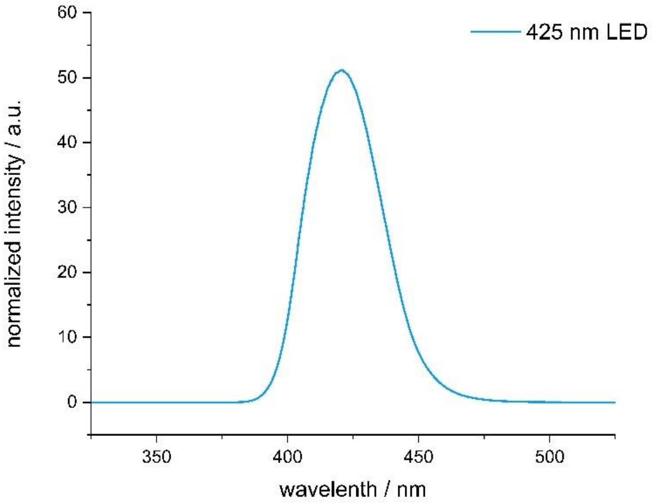

Figure S2. Emission spectrum of the employed LEDs (λ_{max} = 425 nm).

Column chromatography and Solvents Analytical thin layer chromatography (TLC) was carried out with silica gel 60 F254 aluminum plates by Merck. TLC plates were visualized by the exposure to short wave ultraviolet light (254 nm or 365 nm) and/or were dipped into a solution of $KMnO_4$ (3.0 g) and K_2CO_3 (10.0 g) in H_2O (300 mL). Column chromatography was performed using silica gel (40-63 mesh) by Merck eluting with the mentioned solvent system under positive pressurized air flow. Pentane, dichloromethane, ethyl acetate and diethyl ether for column chromatography or recrystallization were purchased of technical grade and further purified *via* distillation. Methanol and ethanol for column chromatography and recrystallization were purchased by Honeywell or Walter-CMP and used as received. Unless otherwise mentioned, dry solvents were utilized to perform the preparation of starting materials and catalytic reactions. The following solvents were purchased from ACROS Organics, Fischer Scientific and Sigma-Aldrich (HPLC grade) and purified with a custom solvent purification system (SPS) with activated alumina columns (built by the "Feinmechanische Werkstatt des Organisch-Chemischen Instituts, Münster University) and collected under positive argon pressure: MeCN, THF, Et_2O, *N,N*-DMF, hexane, toluene, MeOH and dichloromethane.

NMR and deuterated solvents NMR-spectra were recorded at room temperature (in indicated cases, high temperature NMR experiments were performed) on a Bruker Avance II 300, Bruker Avance II 400, Bruker Avance Neo 400, Agilent DD2 500 or on an Agilent DD2 600 spectrometer. Chemicals shifts (δ) are quoted in ppm downfield of tetramethylsilane. The residual solvent signals were used as references for ^1H and ^{13}C NMR spectra (relative to tetramethylsilane at 0.0 ppm, $CDCl_3$: δ_H = 7.26 ppm, δ_C = 77.16 ppm; DMSO-*d*6: δ_H = 2.50 ppm, δ_C = 39.52 ppm). ^{19}F NMR spectra are not calibrated by an internal reference. The multiplicity of all signals was described with standard abbreviations as follows: s = singlet, d = doublet, t = triplet, q = quartet, p = quintet, h = sextet; hept = heptet; m = multiplet, br = broad signal. All the NMRs were processed using Mestrenova 14 applying standard phase and baseline corrections. Coupling constants (*J*) are quoted in Hz. Crude yields were determined by ^1H using CH_2Br_2 as internal standard. If not quoted differently, analytical data is given for the major regio- and diastereoisomer.

GC-MS and GC-FID Samples for GC-MS and GC-FID were filtered over a plug of silica and eluted with EtOAc prior to analysis or were directly collected from the eluted section of the column chromatography on silica gel. GC-MS spectra were recorded on an Agilent Technologies 7890A GC-system using an Agilent 5975C VL MSD or an Agilent 5975 inert Mass Selective Detector (EI) and a HP-5MS column (0.25 mm x 30 m, film: 0.25 µm). GC-FID analysis was evaluated on an Agilent Technologies 6890A using a HP-5 quartz column (0.32 mm · 30 m, film: 0.25 µm) by flame ionization detection. GC-FID calibration was performed using mesitylene as standard.

HRMS High-resolution mass spectra (HRMS) were obtained by the mass department of the Organisch-Chemisches Institut, Universität Münster, using electron ionization (EI) on an Exactive GC-MS by Thermo Fisher Scientific or electrospray ionisation (ESI) on a Bruker Daltonics, MicroToF spectrometer.

Chemicals Photocatalyst [MesAcrMes]ClO_4 (**3a**) was prepared following a literature procedure.[1] Photocatalyst **3b** and **3c** were prepared following a literature procedure.[2] 2-(Methylthio)pyrazine was purchased by CombiBlocks and Fluorochem and used as received. All other commercially available chemicals (e.g. precursors for thioether-containing heteroarene) were used as received if not stated otherwise.

2 Experimental procedures and characterization data

2.1 Starting material synthesis

2.1.1 Synthesis of thioether-containing aza-arenes

General Procedure (GP-A) for the S$_N$Ar reactions of thiols with halogenated aza-arenes

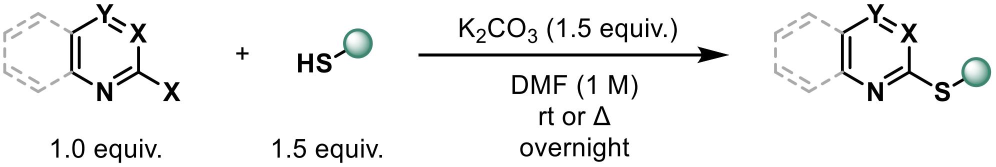

According to a modified literature procedure,[3] a Schlenk tube was equipped with a PTFE-coated stir bar, halogenated aza-arene (1.0 equiv.) and K_2CO_3 (1.5 equiv.) and DMF (1 M) under argon. The thiol (1.5 equiv.) was added and the reaction mixture was stirred at rt or elevated temperatures (indicated below) overnight. The reaction mixture was allowed to reach rt, then diluted with dist. H_2O and EtOAc, the layers were separated and the aq. phase was extracted with EtOAc (2 x). The combined org. layers were washed with brine (3 x), dried over $MgSO_4$, filtered and concentrated in vacuo. Column chromatography on silica gel afforded the desired C2–thioether aza-arenes.

General Procedure (GP-B) for the S$_N$Ar reactions of sodium thiomethoxide with chloro aza-arenes

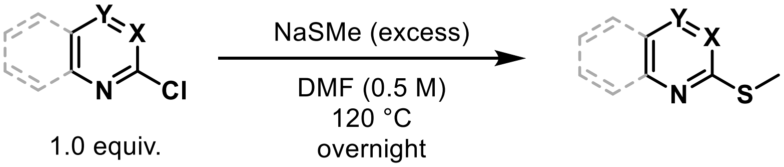

According to a modified literature procedure,[4] a Schlenk tube was equipped with a PTFE-coated stir bar, NaSMe (excess), DMF (0.5 M) and chloro aza-arene (1.0 equiv.). The reaction was stirred at 120 °C overnight. The reaction mixture was allowed to reach rt, then diluted with dist. H_2O and EtOAc, the layers were separated and the aq. phase was extracted with EtOAc (2 x). The combined org. layers were washed with brine (3 x), dried over $MgSO_4$, filtered and concentrated in vacuo. Column chromatography on silica gel afforded the desired C2–thiomethyl aza-arenes.

Note: *Sodium thiomethoxide is prone to decomposition under air and should be handled and stored under argon atmosphere.*

2-(pyrazin-2-ylthio)ethan-1-ol

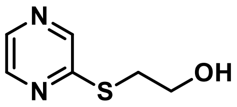

Was synthesized according to **GP-A** using 2-chloropyrazine (0.45 mL, 5.0 mmol, 1.0 equiv.), 2-mercaptoethan-1-ol (0.53 mL, 7.5 mmol, 1.5 equiv.), K_2CO_3 (1.04 g, 7.5 mmol, 1.5 equiv.) and DMF (5 mL, 1 M) at rt. Column chromatography on silica gel (pentane/EtOAc = 50:50 – 40:60) afforded the title compound (287 mg, 1.83 mmol, 37%) as a colorless oil.

R$_f$ (pentane/EtOAc = 50:50) = 0.2;

^{1}H NMR (400 MHz, $CDCl_3$) δ 8.53 (d, J = 1.6 Hz, 1H), 8.32 (dd, J = 2.7, 1.6 Hz, 1H), 8.25 (d, J = 2.7 Hz, 1H), 3.92 (t, J = 5.6 Hz, 2H), 3.41 – 3.33 (m, 2H), 3.08 (s, 1H);

^{13}C NMR (101 MHz, $CDCl_3$) δ 156.7, 144.5, 143.6, 140.1, 62.5, 33.4;

HRMS (ESI$^+$): $[M+Na]^+$ 179.0250; found 179.0249.

2-(2-(pyrazin-2-ylthio)ethyl)isoindoline-1,3-dione

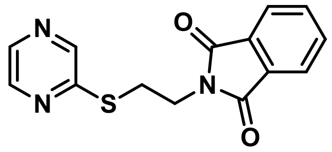

Was synthesized in two steps. 2-(2-mercaptoethyl)isoindoline-1,3-dione was synthesized according to a literature procedure.[5] The second step was performed according to modified **GP-A** using 2-chloropyrazine (0.32 mL, 3.6 mmol, 1.2 equiv.), 2-(2-mercaptoethyl)isoindoline-1,3-dione (0.62 g, 3.0 mmol, 1.0 equiv.), K_2CO_3 (0.62 g, 4.5 mmol, 1.5 equiv.) and DMF (3 mL, 1 M) at rt. Recrystallization from EtOAc/pentane afforded the title compound (0.59 g, 2.1 mmol, 69%) as colorless crystals.

R$_f$ (EtOAc) = 0.7;

^{1}H NMR (400 MHz, $CDCl_3$) δ 8.41 (d, J = 1.6 Hz, 1H), 8.29 (dd, J = 2.6, 1.6 Hz, 1H), 8.17 (d, J = 2.7 Hz, 1H), 7.87 – 7.78 (m, 2H), 7.75 – 7.66 (m, 2H), 4.05 (t, J = 6.5 Hz, 2H), 3.52 (t, J = 6.5 Hz, 2H);

^{13}C NMR (101 MHz, $CDCl_3$) δ 168.2, 155.8, 144.1, 144.0, 139.8, 134.2, 132.1, 123.4, 37.2, 28.0;

HRMS (ESI$^+$): $[M+Na]^+$ 308.0464; found 308.0465.

2-((3-(trimethoxysilyl)propyl)thio)pyrazine

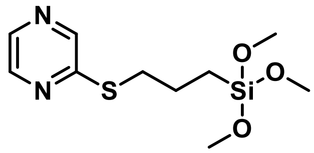

Was synthesized according to modified **GP-A** using 2-chloropyrazine (0.32 mL, 3.6 mmol, 1.2 equiv.), 3-(trimethoxysilyl)propane-1-thiol (0.56 mL, 3.0 mmol, 1.0 equiv.), K_2CO_3 (0.62 g, 4.5 mmol, 1.5 equiv.) and DMF (3 mL, 1 M) at rt. Column chromatography on silica gel (pentane/EtOAc = 80:20) afforded the title compound (0.30 g, 1.1 mmol, 36%) as a colorless oil.

R$_f$ (pentane/EtOAc = 80:20) = 0.3;

^{1}H NMR (400 MHz, $CDCl_3$) δ 8.43 (d, J = 1.6 Hz, 1H), 8.34 (dd, J = 2.7, 1.6 Hz, 1H), 8.17 (d, J = 2.7 Hz, 1H), 3.56 (s, 9H), 3.24 – 3.15 (m, 2H), 1.89 – 1.77 (m, 2H), 0.84 – 0.77 (m, 2H);

^{13}C NMR (101 MHz, $CDCl_3$) δ 157.5, 144.1, 144.0, 139.2, 50.7, 32.6, 23.0, 8.9;

HRMS (ESI$^+$): $[M+Na]^+$ 297.0700; found 297.0700.

Note**:** *Chloro aza-arene was used in excess to avoid thiol impurities upon isolation.*

2-(benzylthio)-6-phenylpyrazine

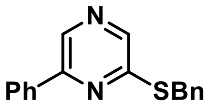

Was synthesized in two steps. According to modified **GP-A** using 2,6-dichloropyrazine (1.50 g, 10.0 mmol, 1.1 equiv.), benzylthiol (1.06 mL, 9.0 mmol, 1.0 equiv.), K_2CO_3 (1.25 g, 9.0 mmol, 1.0 equiv.) and DMF (23 mL) at rt. Column chromatography on silica gel (pentane/EtOAc = 75:25) afforded 2-(benzylthio)-6-chloropyrazine (1.64 g, 6.9 mmol, 77%) as a colorless oil. According to a modified literature procedure,[6] to a Schlenk tube equipped with a PTFE-coated stir bar was added 2-(benzylthio)-6-chloropyrazine (472 mg, 2.0 mmol, 1.0 equiv.), phenylboronic acid (293 mg, 2.4 mmol, 1.2 equiv.), $Pd(OAc)_2$ (9 mg, 0.04 mmol, 2 mol%), XPhos (22.8 mg, 0.048 mmol, 2.4 mol%), LiOH x H_2O (142 mg, 3.38 mmol, 1.69 equiv.) butanol (10 mL) and dist. deionized water (2.8 mL) under argon. The reaction mixture was stirred at rt overnight. Upon completion, brine was added and the reaction mixture was extracted with EtOAc (3 x). The combined org. layers were dried over $MgSO_4$, filtered and concentrated in vacuo. Column chromatography on silica gel (pentane/EtOAc = 95:5) afford the title compound (304 mg, 1.1 mmol, 55%) as a yellow oil.

R_f (pentane/EtOAc = 95:5) = 0.3;

^{1}H NMR (400 MHz, $CDCl_3$) δ 8.68 (s, 1H), 8.37 (s, 1H), 8.08 – 8.00 (m, 2H), 7.55 – 7.46 (m, 3H), 7.47 – 7.42 (m, 2H), 7.36 – 7.21 (m, 3H), 4.55 (s, 2H);

^{13}C NMR (101 MHz, $CDCl_3$) δ 155.6, 151.6, 141.6, 137.6, 136.7, 136.3, 130.1, 129.1, 129.1, 128.8, 127.5, 127.0, 34.0;

HRMS (ESI$^+$): $[M+Na]^+$ 301.0770; found 301.0770.

2-(cyclohexylthio)pyrazine

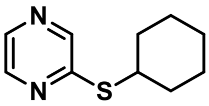

Was synthesized according to **GP-A** using 2-chloropyrazine (0.44 mL, 5.0 mmol, 1.0 equiv.), cyclohexylthiol (0.92 mL, 7.5 mmol, 1.5 equiv.), K_2CO_3 (1.04 g, 7.5 mmol, 1.5 equiv.) and DMF (5 mL, 1 M) at rt. Column chromatography on silica gel (pentane/EtOAc = 75:25) afforded the title compound (0.68 g, 3.5 mmol, 70%) as a colorless solid. The spectral data are in good agreement with the literature.[7]

2-(methylselanyl)pyrazine

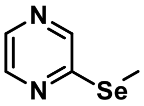

Was synthesized according to a literature procedure[8] using pyrazine (0.48 g, 6.0 mmol, 1.0 equiv.), LDA (2 M solution in heptane, 3.3 mL, 6.6 mmol, 1.2 equiv.), selenium powder (0.52 g, 6.6 mmol, 1.2 equiv.) and MeI (0.41 mL, 6.6 mmol, 1.2 equiv.) in THF (15 mL). Column chromatography on silica gel (pentane/EtOAc = 95:5 – 90:10) afforded the title compound (0.12 g, 0.7 mmol, 12%) as a brown solid. The spectral data are in good agreement with the literature.[8]

methyl 6-(butylthio)nicotinate

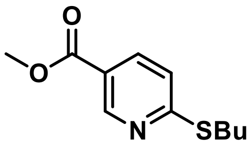

Was synthesized according to modified **GP-A** using 6-chloronicotinic acid (0.65 g, 3.8 mmol, 1.0 equiv.), butan-1-thiol (0.61 mL, 5.7 mmol, 1.5 equiv.), K_2CO_3 (788 mg, 5.7 mmol, 1.5 equiv.) and DMF (4 mL, 1 M) at rt. Column chromatography on silica gel (pentane/EtOAc = 99:1) afforded the title compound (780 mg, 3.46 mmol, 91%) as a colorless liquid.

R_f (pentane/EtOAc = 99:1) = 0.3;

^{1}H NMR (400 MHz, $CDCl_3$) δ 9.00 (dd, J = 2.3, 0.9 Hz, 1H), 8.01 (dd, J = 8.4, 2.2 Hz, 1H), 7.19 (dd, J = 8.4, 0.9 Hz, 1H), 3.91 (s, 3H), 3.27 – 3.17 (m, 2H), 1.76 – 1.61 (m, 2H), 1.57 – 1.43 (m, 2H), 0.95 (t, J = 7.3 Hz, 3H).

^{13}C NMR (101 MHz, $CDCl_3$) δ 166.1, 165.7, 150.9, 136.3, 121.6, 121.3, 52.3, 31.4, 30.0, 22.2, 13.8;

HRMS (ESI$^+$): $[M+Na]^+$ 248.0716; found 248.0716.

1-(6-(butylthio)pyridin-3-yl)ethan-1-one

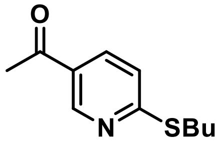

Was synthesized according to modified **GP-A** using 1-(6-bromopyridin-3-yl)ethan-1-one (497 mg, 2.5 mmol, 1.0 equiv.), butan-1-thiol (0.40 mL, 3.75 mmol, 1.5 equiv.), K_2CO_3 (518 mg, 3.75 mmol, 1.5 equiv.) and DMF (4 mL, 0.6 M) at 60 °C. Column chromatography on silica gel (pentane/EtOAc = 95:5) afforded the title compound (471 mg, 2.25 mmol, 90%) as a colorless liquid.

R_f (pentane/EtOAc = 95:5) = 0.3;

^{1}H NMR (400 MHz, $CDCl_3$) δ 8.94 (dd, J = 2.3, 0.8 Hz, 1H), 7.98 (dd, J = 8.5, 2.3 Hz, 1H), 7.21 (dd, J = 8.5, 0.8 Hz, 1H), 3.25 – 3.17 (m, 2H), 2.57 (s, 3H), 1.70 (tdd, J = 7.6, 6.5, 4.9 Hz, 2H), 1.47 (dq, J = 14.6, 7.4 Hz, 2H), 0.94 (t, J = 7.3 Hz, 3H);

^{13}C NMR (101 MHz, $CDCl_3$) δ 196.3, 166.0, 150.1, 134.8, 128.4, 121.7, 31.4, 29.9, 26.6, 22.2, 13.8;

HRMS (ESI$^+$): $[M+Na]^+$ 210.0947, found 210.0946.

6-(butylthio)nicotinamide

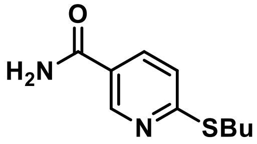

According to a modified literature procedure[9] a solution of methyl 6-(butylthio)nicotinate (448 mg, 2.00 mmol, 1.0 equiv.) in methanolic ammonia (2.0 mL, 14.0 mmol, 7.0 equiv., 7 M) was stirred at 70 °C for 16 h. After completion of the reaction, the solvent was removed under reduced pressure. The crude was triturated with diethylether (10 ml), filtered through a buchner funnel and dried under vaccum affording the desired product as a colorless solid (268 mg, 1.06 mmol, 53%).

R_f (CH_2Cl_2/MeOH = 95:5) = 0.5;

^{1}H NMR (400 MHz, $CDCl_3$) δ 8.80 (dd, J = 2.4, 0.9 Hz, 1H), 7.90 (dd, J = 8.4, 2.4 Hz, 1H), 7.22 (dd, J = 8.4, 0.9 Hz, 1H), 5.81 (s, 2H, br), 3.25 – 3.16 (m, 2H), 1.70 (tt, J = 8.6, 6.8 Hz, 2H), 1.48 (dq, J = 14.6, 7.3 Hz, 2H), 0.95 (t, J = 7.4 Hz, 3H);

^{13}C NMR (101 MHz, $CDCl_3$) δ 167.4, 164.8, 148.1, 135.0, 124.4, 121.7, 31.4, 30.0, 22.2, 13.8;

HRMS (ESI$^+$): $[M+Na]^+$ 233.0719; found 233.0718.

5-bromo-2-(butylthio)pyridine

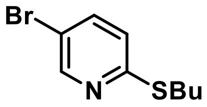

Was synthesized according to modified **GP-A** using 2-chloropyrazine (1.49 g, 7.8 mmol, 1.0 equiv.), butan-1-thiol (1.26 mL, 7.5 mmol, 1.0 equiv.), K_2CO_3 (1.62 g, 7.5 mmol, 1.5 equiv.) and DMF (8 mL, 1 M) at rt. Column chromatography on silica gel (pentane/EtOAc = 100:0 – 99:1) afforded the title compound (1.19 g, 4.85 mmol, 62%) as a colorless liquid.

R_f (pentane) = 0.2;

^{1}H NMR (400 MHz, $CDCl_3$) δ 8.46 (dd, *J* = 2.4, 0.8 Hz, 1H), 7.56 (dd, *J* = 8.5, 2.4 Hz, 1H), 7.05 (dd, *J* = 8.5, 0.8 Hz, 1H), 3.13 (t, 2H), 1.67 (tt, *J* = 8.6, 6.8 Hz, 2H), 1.51 – 1.40 (m, 2H), 0.94 (t, *J* = 7.3 Hz, 3H);

^{13}C NMR (101 MHz, $CDCl_3$) δ 158.6, 150.4, 138.4, 123.4, 115.8, 31.5, 30.2, 22.2, 13.8;

HRMS (ESI$^+$): $[M+Na]^+$ 267.9766; found 267.9766.

2-(butylthio)pyridine

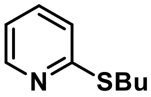

A 50 mL Schlenk tube equipped with a PTFE-coated stir bar was charged with pyridine-2-thiol (0.56 g, 5.0 mmol, 1.0 equiv.), K_2CO_3 (1.04 g, 7.5 mmol, 1.5 equiv.) and DMF (10 mL, 0.2 M). 1-Bromobutane (0.59 mL, 5.5 mmol, 1.1 equiv.) was added dropwise and the reaction mixture was stirred for 4 h at 90 °C. Upon cooling to rt, it was then diluted with dist. H_2O and Et_2O, the layers were separated and the aq. phase was extracted with Et_2O (2 x). The combined org. layers were washed with brine (3 x), dried over $MgSO_4$, filtered and concentrated in vacuo. Column chromatography on silica gel (pentane/Et_2O = 92:8) afforded the title compound (0.79 g, 4.7 mmol, 94%) as a colorless liquid. The spectral data are in good agreement with the literature.[10]

2-(butylthio)-5-(trifluoromethyl)pyridine

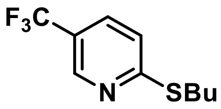

Was synthesized according to modified **GP-A** using 2-chloro-5-(trifluoromethyl)pyridine (1.0 g, 5.5 mmol, 1.0 equiv.), butan-1-thiol (1.19 mL, 11.0 mmol, 2.0 equiv.), K_2CO_3 (1.53 g, 11.0 mmol, 2.0 equiv.) and DMF (10 mL, 0.55 M) at 60 °C. Column chromatography on silica gel (pentane/EtOAc = 99:1) afforded the title compound (0.84 g, 3.57 mmol, 65%) as a colorless liquid.

R_f (pentane/EtOAc = 99:1) = 0.4;

^{1}H NMR (400 MHz, $CDCl_3$) δ 8.65 (dt, *J* = 2.5, 1.0 Hz, 1H), 7.64 (ddd, *J* = 8.5, 2.4, 0.7 Hz, 1H), 7.24 (dt, *J* = 8.5, 0.8 Hz, 1H), 3.24 – 3.15 (m, 2H), 1.70 (tt, *J* = 8.6, 6.8 Hz, 2H), 1.53 – 1.42 (m, 2H), 0.95 (t, *J* = 7.3 Hz, 3H);

^{13}C {^{19}F} NMR (101 MHz, $CDCl_3$) δ 164.9, 146.4, 132.5, 124.0, 122.1, 121.6, 31.4, 29.9, 22.2, 13.8;

^{19}F NMR (376 MHz, $CDCl_3$) δ –62.2;

HRMS (ESI$^+$): $[M+H]^+$ 236.0715; found 236.0715.

tert-butyl 4-(6-(butylthio)nicotinoyl)piperazine-1-carboxylate

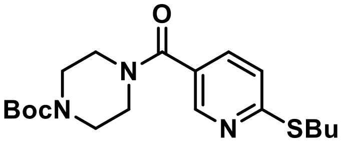

Was synthesized in two steps from 6-chloronicotinic acid according to a literature procedure.[11] The corresponding acid (551 mg, 3.5 mmol, 1.0 equiv.), 2-(1H-benzotriazol-1-yl)-1,1,3,3-tetramethyluronium hexafluorophosphate (1.33 g, 3.5 mmol, 1.0 equiv.), K_2CO_3 (967 mg, 7.0 mmol, 2.0 equiv.) was stirred in acetone (45 mL, 0.07 M) for 30 min at rt before adding *tert*-butyl piperazine-1-carboxylate (652 mg, 3.5 mmol, 1.0 equiv.) with additional stirring for 16 h at rt. The reaction mixture was then diluted with dist. H_2O, the layers were separated and the aq. phase was extracted with EtOAc (3 x). The combined org. layers were dried over $MgSO_4$, filtered and concentrated in vacuo. Column chromatography on silica gel (pentane/Et_2O = 40:60) afforded *tert*-butyl 4-((6-chloronicotinoyl)oxy)piperazine-1-carboxylate (1.14 g, 3.5 mmol, *quant*.) as a colorless solid. The title compound was synthesized according to modified **GP-A** using *tert*-butyl 4-((6-chloronicotinoyl)oxy)piperazine-1-carboxylate (1.14 g, 3.50 mmol, 1.0 equiv.), butan-1-thiol (0.57 mL, 5.25 mmol, 1.5 equiv.), K_2CO_3 (725 mg, 5.25 mmol, 1.5 equiv.) and DMF (3.5 mL, 1 M) at rt. Column chromatography on silica gel (pentane/EtOAc = 50:50) afforded the title compound (685 mg, 1.66 mmol, 47%) as a colorless solid.

R_f (pentane/EtOAc = 50:50) = 0.4;

^{1}H NMR (400 MHz, $CDCl_3$) δ 8.46 (dt, *J* = 2.3, 1.2 Hz, 1H), 7.53 (dt, *J* = 8.3, 2.3 Hz, 1H), 7.20 (dt, *J* = 8.3, 1.3 Hz, 1H), 3.90 – 3.35 (m, 8H), 3.22 – 3.14 (m, 2H), 1.76 – 1.63 (m, 2H), 1.54 – 1.40 (m, 11H), 0.94 (td, *J* = 7.3, 2.5 Hz, 3H);

^{13}C NMR (101 MHz, $CDCl_3$) δ 168.4, 162.5, 154.6, 148.0, 135.1, 126.4, 121.8, 80.6, 43.8 (br), 31.4, 30.0, 28.5, 22.2, 13.8;

HRMS (ESI^+): $[M+Na]^+$ 402.1827; found 402.1814.

(3a*S*,6*R*,6a*S*)-5-((*R*)-2,2-dimethyl-1,3-dioxolan-4-yl)-2,2-dimethyltetrahydrofuro[2,3-*d*][1,3]dioxol-6-yl 6-(butylthio)nicotinate

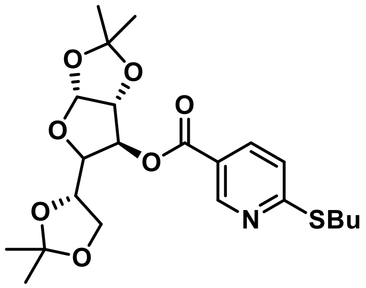

Was synthesized in two steps from 6-chloronicotinic acid. According to a modified literature procedure[12] the respective carboxylic acid (188 mg, 1.2 mmol, 1.2 equiv), (3a*S*,5*R*,6*R*,6a*S*)-5-((*R*)-2,2-dimethyl-1,3-dioxolan-4-yl)-2,2-dimethyltetrahydrofuro[2,3-*d*][1,3]dioxol-6-ol (260 mg, 1.0 mmol, 1.0 equiv), DMAP (146 mg, 1.2 mmol, 1.2 equiv), EDC·HCl (288 mg, 1.5 mmol, 1.5 equiv) and CH_2Cl_2 (1 mL, 1 M) were added to a round bottom flask equipped with a PTFE-coated stir bar on open air. The reaction was stirred overnight at rt. The solvent was removed under reduced pressure. Column chromatography on silica gel (pentane/EtOAc = 85:15) afforded the corresponding ester (399 mg, 1.00 mmol, *quant.*) as a colorless oil. The title compound was synthesized according to modified **GP-A** using the corresponding ester (399 mg, 1.0 mmol, 1.0 equiv.), butan-1-thiol (0.16 mL, 1.5 mmol, 1.5 equiv.), K_2CO_3 (207 mg, 1.5 mmol, 1.5 equiv.) and DMF (1 mL, 1 M) at rt. Column chromatography on silica gel (pentane/EtOAc = 90:10) afforded the title compound (211 mg, 0.54 mmol, 54%) as a colorless oil.

R_f (pentane/EtOAc = 90:10) = 0.4;

^{1}H NMR (400 MHz, $CDCl_3$) *δ* 8.99 (dd, *J* = 2.3, 0.9 Hz, 1H), 7.99 (dd, *J* = 8.5, 2.3 Hz, 1H), 7.21 (dd, *J* = 8.4, 0.9 Hz, 1H), 5.93 (d, *J* = 3.7 Hz, 1H), 5.48 (d, *J* = 2.6 Hz, 1H), 4.62 (d, *J* = 3.7 Hz, 1H), 4.37 – 4.25 (m, 2H), 4.08 (qd, *J* = 8.7, 4.9 Hz, 2H), 3.29 – 3.13 (m, 2H), 1.75 – 1.63 (m, 2H), 1.54 (s, 3H), 1.53 – 1.43 (m, 2H), 1.40 (s, 3H), 1.31 (s, 3H), 1.26 (s, 3H), 0.94 (t, *J* = 7.4 Hz, 3H);

^{13}C NMR (101 MHz, $CDCl_3$) *δ* 166.5, 164.4, 150.9, 136.3, 121.5, 121.0, 112.6, 109.6, 105.3, 83.5, 80.1, 72.7, 67.5, 31.4, 30.0, 27.0, 26.9, 26.4, 25.4, 22.2, 13.8;

166.4, 164.3, 150.8, 136.3, 121.5, 120.9, 112.5, 109.6, 105.2, 83.5, 80.1, 76.9, 72.7, 67.5, 31.4, 30.0, 27.0, 26.9, 26.3, 25.3, 22.2, 13.8;

HRMS (ESI$^+$): $[M+Na]^+$ 476.1713; found 476.1716.

(6-(butylthio)pyridin-3-yl)(3-(trifluoromethyl)-5,6-dihydro-[1,2,4]triazolo[4,3-*a*]pyrazin-7(8*H*)-yl)methanone

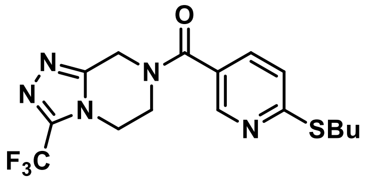

Was synthesized in two steps from 6-chloronicotinic acid according to a literature procedure.[11] The corresponding acid (630 mg, 4.0 mmol, 1.0 equiv.), 2-(1H-benzotriazol-1-yl)-1,1,3,3-tetramethyluronium hexafluorophosphate (1.52 g, 4.0 mmol, 1.0 equiv.), K_2CO_3 (1.11 g, 8.0 mmol, 2.0 equiv.) was stirred in acetone (50 mL, 0.07 M) for 30 min at rt before adding 3-(Trifluoromethyl)-5,6,7,8-tetrahydro-[1,2,4]triazolo[4,3-a]pyrazine hydrochloride (768 mg, 4.0 mmol, 1.0 equiv.) with additional stirring for 16 h at rt. The reaction mixture was then diluted with dist. H_2O, the layers were separated and the aq. phase was extracted with EtOAc (3 x). The combined org. layers were dried over $MgSO_4$, filtered and concentrated in vacuo. Column chromatography on silica gel (pentane/Et_2O = 10:90) afforded the corresponding amide (1.36 g, 3.91 mmol, 98%) as a colorless solid. The title compound was synthesized according to modified **GP-A** using the corresponding amide (993 mg, 3.0 mmol, 1.0 equiv.), butan-1-thiol (0.49 mL, 4.5 mmol, 1.5 equiv.), K_2CO_3 (622 mg, 4.5 mmol, 1.5 equiv.) and DMF (4 mL, 0.75 M) at rt for 3 h. Column chromatography on silica gel (pentane/EtOAc = 10:90) afforded the title compound (624 mg, 1.55 mmol, 52%) as a colorless sticky oil.

R_f (pentane/EtOAc = 50:50) = 0.4;

^{1}H NMR (400 MHz, $CDCl_3$) *δ* 8.38 (dd, *J* = 2.3, 0.9 Hz, 1H), 7.48 (dd, *J* = 8.3, 2.3 Hz, 1H), 7.08 (dd, *J* = 8.4, 0.9 Hz, 1H), 4.88 (s, 2H), 4.14 (t, *J* = 5.6 Hz, 2H), 4.09 – 3.83 (m, 2H), 3.06 (t, *J* = 7.4 Hz, 2H), 1.58 (tt, *J* = 8.6, 6.8 Hz, 2H), 1.43 – 1.30 (m, 2H), 0.84 (t, *J* = 7.4 Hz, 3H);

^{13}C NMR (101 MHz, $CDCl_3$) *δ* 168.6, 163.5, 149.9, 147.9, 143.4 (q, *J* = 39.9 Hz), 135.0, 124.6, 121.3, 118.0 (q, *J* = 270.5 Hz), 43.2, 41.4 (br), 38.3, 31.0, 29.7, 21.8, 13.5;

^{19}F NMR (470 MHz, $CDCl_3$) *δ* –63.0;

HRMS (ESI$^+$): $[M+Na]^+$ 408.1076; found 408.1070.

2-(benzylthio)isonicotinonitrile

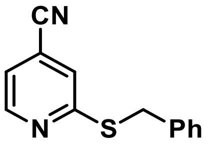

Was synthesized according to modified **GP-A** using 2-chloroisonicotinonitrile (0.42 g, 3.0 mmol, 1.0 equiv.), phenylmethanethiol (0.53 mL, 4.5 mmol, 1.5 equiv.), K_2CO_3 (0.62 g, 4.5 mmol, 1.5 equiv.) and DMF (3 mL, 1 M) at rt. Column chromatography on silica gel (pentane/EtOAc = 96:4 – 94:6) afforded the title

compound (563 mg, 2.48 mmol, 83%) as a colorless solid. The spectral data are in good agreement with the literature.[13]

2-(benzylthio)-3-chloropyridine

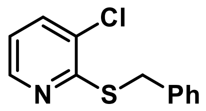

Was synthesized according to modified **GP-A** using 2,3-dichloropyridine (444 mg, 3.0 mmol, 1.0 equiv.), phenylmethanethiol (0.39 mL, 3.3 mmol, 1.1 equiv.), K_2CO_3 (0.46 g, 3.3 mmol, 1.1 equiv.) and DMF (3 mL, 1 M) at rt. Column chromatography on silica gel (100% pentane) afforded the title compound (695 mg, 2.96 mmol, 99%) as a colorless liquid.

R_f (pentane) = 0.2;

^{1}H NMR (400 MHz, $CDCl_3$) δ 8.37 (dd, *J* = 4.8, 1.5 Hz, 1H), 7.54 (dd, *J* = 7.9, 1.5 Hz, 1H), 7.47 – 7.39 (m, 2H), 7.34 – 7.28 (m, 2H), 7.26 – 7.21 (m, 1H), 6.98 (dd, *J* = 7.9, 4.8 Hz, 1H), 4.46 (s, 2H);

^{13}C NMR (101 MHz, $CDCl_3$) δ 157.4, 147.0, 137.7, 136.0, 129.3, 129.0, 128.6, 127.3, 120.0, 34.7;

HRMS (ESI$^+$): $[M+H]^+$ 258.0115; found 258.0113.

2-(methylthio)quinoline

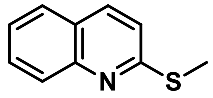

Was synthesized according to **GP-B** using 2-chloroquinoline (818 mg, 5.0 mmol, 1.0 equiv.), NaSMe (0.51 g, 7.3 mmol, 1.5 equiv.), and DMF (10 mL, 0.5 M) at rt. Column chromatography on silica gel (pentane/EtOAc = 97:3) afforded the title compound (0.74 g, 4.2 mmol, 84%) as a colorless oil. The spectral data are in good agreement with the literature.[14]

2-(((2,2-dimethyl-1,3-dioxolan-4-yl)methyl)thio)quinoline

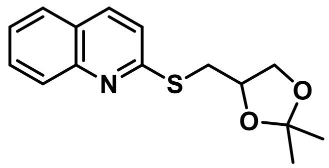

Was synthesized in two steps. According to **GP-A** using 2-chloroquinoline (494 mg, 3.0 mmol, 1.0 equiv.), 3-mercaptopropane-1,2-diol (389 µL, 4.5 mmol, 1.5 equiv.), K_2CO_3 (622 mg, 4.5 mmol, 1.5 equiv.) and DMF (3 mL, 1M) at rt. Column chromatography on silica gel (EtOAc) afforded 3-(quinolin-2-ylthio)propane-1,2-diol (677 mg, 2.88 mmol, 96%) as an light yellow oil. To this, pyridinium *p*-toluenesulfonate (73 mg, 0.29 mmol, 0.1 equiv.), $MgSO_4$ (520 mg, 4.32 mmol, 1.5 equiv.) and acetone (5.8 mL, 0.5 M) together with a stir bar in a 20 mL drum vial was added under air. The reaction mixture was stirred for 70 h at rt. Upon completion, the solvent was removed and dist. H_2O and EtOAc were added, the layers were separated and the aq. phase was extracted with EtOAc (2 x). The combined org. layers were dried over $MgSO_4$, filtered and concentrated in vacuo. Column chromatography on silica gel (pentane/EtOAc = 95:5) afforded the title compound (290 mg, 1.05 mmol, 37%) as a colorless oil.

R_f (pentane/EtOAc = 95:5) = 0.2;

^{1}H NMR (400 MHz, $CDCl_3$) δ 7.87 (dd, *J* = 8.4, 1.1 Hz, 1H), 7.82 (dd, *J* = 8.6, 0.8 Hz, 1H), 7.64 (dd, *J* = 8.1, 1.5 Hz, 1H), 7.57 (ddd, *J* = 8.4, 6.9, 1.5 Hz, 1H), 7.36 (ddd, *J* = 8.1, 7.0, 1.2 Hz, 1H), 7.14 (d, *J* = 8.6 Hz, 1H), 4.49 – 4.38 (m, 1H), 4.07 (dd, *J* = 8.5, 6.1 Hz, 1H), 3.79 (dd, *J* = 8.4, 6.1 Hz, 1H), 3.62 (dd, *J* = 13.6, 5.6 Hz, 1H), 3.44 (dd, *J* = 13.6, 6.7 Hz, 1H), 1.43 (s, 3H), 1.30 (s, 3H);

^{13}C NMR (101 MHz, $CDCl_3$) δ 158.2, 148.3, 135.7, 129.9, 128.1, 127.8, 126.2, 125.6, 121.1, 109.7, 75.1, 68.9, 32.5, 27.2, 25.8;

HRMS (ESI^+): $[M+Na]^+$ 298.0872; found 298.0873.

methyl 3-(quinolin-2-ylthio)propanoate

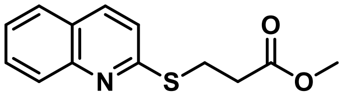

Was synthesized according to **GP-A** using 2-chloroquinoline (230 mg, 1.41 mmol, 1.0 equiv.), methyl 3-mercaptopropanoate (227 µL, 2.11 mmol, 1.5 equiv.), K_2CO_3 (283 mg, 2.11 mmol, 1.5 equiv.) and DMF (1.4 mL, 1M) at rt. Column chromatography on silica gel (pentane/EtOAc = 97:3) afforded the title compound (144 mg, 0.58 mmol, 41%) as a colorless oil. The spectral data are in good agreement with the literature.[3]

6-(benzylthio)imidazo[1,2-*b*]pyridazine

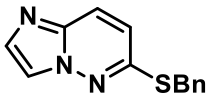

Was synthesized according to modified **GP-A** using 6-chloroimidazo[1,2-*b*]pyridazine (0.46 g, 3.0 mmol, 1.00 equiv.), benzylthiol (0.48 mL, 4.0 mmol, 1.33 equiv.), K_2CO_3 (0.62 g, 4.5 mmol, 1.50 equiv.) and DMF (10 mL) at 70 °C. Column chromatography on silica gel (pentane/EtOAc = 25:75) afforded the title compound (0.35 g, 1.0 mmol, 34%) as a colorless solid.

R_f (pentane/EtOAc = 25:75) = 0.4;

^{1}H NMR (400 MHz, $CDCl_3$) δ 7.87 (t, J = 0.9 Hz, 1H), 7.71 (dd, J = 9.4, 0.7 Hz, 1H), 7.65 (d, J = 1.2 Hz, 1H), 7.47 – 7.40 (m, 2H), 7.33 (ddt, J = 8.0, 6.4, 1.1 Hz, 2H), 7.30 – 7.24 (m, 1H), 6.82 (d, J = 9.5 Hz, 1H), 4.43 (s, 2H);

^{13}C NMR (101 MHz, $CDCl_3$) δ 153.7, 137.6, 136.6, 132.9, 129.3, 128.8, 127.7, 124.9, 117.9, 116.8, 34.9;

HRMS (ESI^+): $[M+Na]^+$ 264.0566; found 264.0565.

methyl 3-(quinoxalin-2-ylthio)propanoate

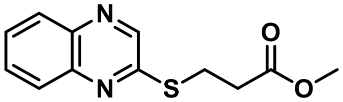

Was synthesized according to **GP-A** using 2-chloroquinoxaline (329 mg, 2.0 mmol, 1.0 equiv.), methyl 3-mercaptopropanoate (332 µL, 3.0 mmol, 1.5 equiv.), K_2CO_3 (415 mg, 3.0 mmol, 1.5 equiv.) and DMF (2 mL, 1M) at rt. Column chromatography on silica gel (pentane/EtOAc = 92:8 – 88:12) afforded the title compound (307 mg, 1.2 mmol, 62%) as a yellow solid. The spectral data are in good agreement with the literature.[3]

2-(methylthio)benzo[*d*]thiazole

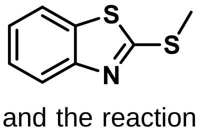

A 50 mL round bottom was charged with a PTFE-coated stir bar, benzo[*d*]thiazole-2-thiol (1.67 g, 10 mmol, 1.0 equiv.), K_2CO_3 (2.07 g, 15 mmol, 1.5 equiv.) and DMF (15 mL) at rt under air. MeI (0.75 mL, 12 mmol, 1.2 equiv.) was added dropwise and the reaction was stirred at rt overnight. The reaction mixture diluted with dist. H_2O and EtOAc,

the layers were separated and the aq. phase was extracted with EtOAc (2 x). The combined org. layers were washed with brine (1 x), dried over $MgSO_4$, filtered and concentrated in vacuo. Column chromatography on silica gel (pentane/EtOAc = 97:3) afforded the title compound (1.56 g, 8.6 mmol, 86%) as a colorless solid. The spectral data are in good agreement with the literature.[15]

2-(methylthio)benzo[d]oxazole

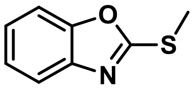

A 50 mL round bottom was charged with a PTFE-coated stir bar, benzo[*d*]oxazole-2-thiol (1.20 g, 8.0 mmol, 1.0 equiv.), K_2CO_3 (1.66 g, 12.0 mmol, 1.5 equiv.) and DMF (15 mL) at rt under air. MeI (0.60 mL, 9.6 mmol, 1.2 equiv.) was added dropwise and the reaction was stirred at rt overnight. The reaction mixture diluted with dist. H_2O and EtOAc, the layers were separated and the aq. phase was extracted with EtOAc (2 x). The combined org. layers were washed with brine (1 x), dried over $MgSO_4$, filtered and concentrated in vacuo. Column chromatography on silica gel (pentane/EtOAc = 90:10 – 80:20) afforded the title compound (1.25 g, 7.6 mmol, 95%) as a colorless oil. The spectral data are in good agreement with the literature.[16]

2-(butylthio)thiazole

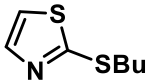

Was synthesized according to **GP-A** using 2-bromothiazole (0.27 mL, 3.0 mmol, 1.0 equiv.), butane-1-thiol (0.48 mL, 4.5 mmol, 1.5 equiv.), K_2CO_3 (622 mg, 4.5 mmol, 1.5 equiv.) and DMF (3 mL, 1 M) at 90 °C. Column chromatography on silica gel (pentane/EtOAc = 97:3) afforded the title compound (331 mg, 1.91 mmol, 63%) as a colorless oil. The spectral data are in good accordance with the literature.[15]

2.1.2 Synthesis of bicyclobutanes (BCBs)

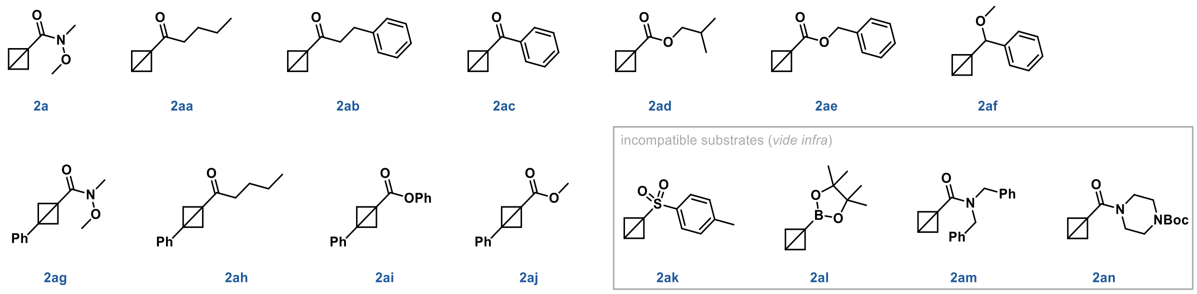

All synthesized BCBs are literature known compounds. **2a** was synthesized according to a modified literature procedure by Brown and coworkers.[17] **2aa** and **2ac** were synthesized by a modified procedure from our group.[18] **2ab** was synthesized according to a literature procedure by Procter and coworkers.[19] **2ad** and **2ae** were synthesized by a modified procedure by our group.[20] **2af** was previously synthesized in our group[21] according to a modified procedure by Walczak and Wipf.[22] **2ag** was previously synthesized in our group[23] according to a modified literature procedure by Procter and coworkers.[19] **2ah** was previously synthesized in our group.[24] **2ai** was previously synthesized in our group[23] according to a literature procedure by Ma and coworkers.[25] **2aj** was previously synthesized in our group[24] according to a literature procedure by Chen and Tan.[26] **2ak** was previously synthesized in our group[27] according to a literature procedure by Baran and coworkers.[28] **2al** was previously synthesized in our group[21] according to a literature procedure by Aggarwal and coworkers.[29] **2am** and **2an** were synthesized by a modified literature procedure by Gryko and co-workers.[30]

2.2 General procedure (GP-1) for the catalytic reaction of aza-arenes with BCBs

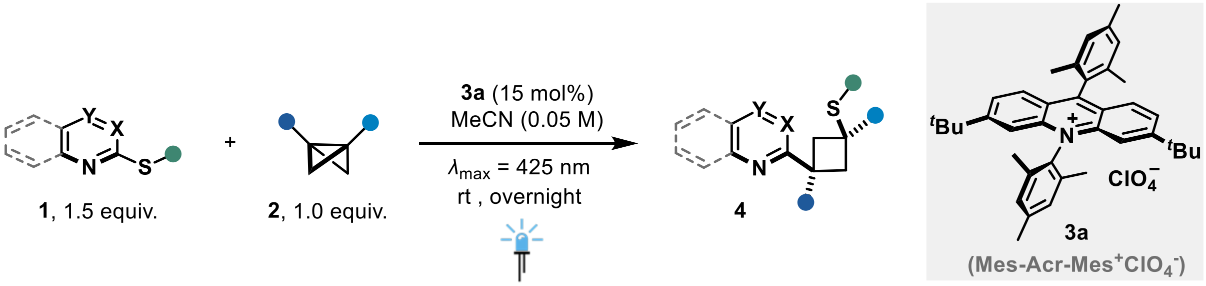

A small oven-dried Schlenk tube was equipped with a PTFE-coated stir bar, **3a** (15 mol%) as well as **1** and **2** if these are solids or high-boiling oils. If **1** or **2** were liquids, these were charged after the Schlenk tube was evacuated and back-filled with argon three times. MeCN (0.05 M) was added and the Schlenk tube was sealed. Irradiation with blue LEDs (λ_{max} = 425 nm) was conducted overnight (approx. 16 h). The solvent was removed in vacuo and the crude yield was determined by ^{1}H NMR using dibromomethane as internal standard or GC-FID using mesitylene as internal standard. Furthermore, the diastereomeric and regioisomeric ratios were determined by crude ^{1}H NMR. Purification by column chromatography on silica gel (solvent mixtures indicated below) afforded the analytically pure insertion products, either as single isomers or a mixture of isomers (indicated below).

2.3 Optimization studies and control experiments

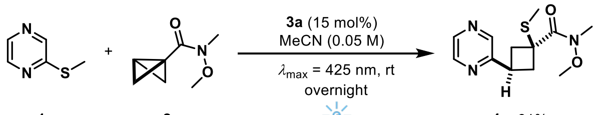

1a (2.0 equiv.) + **2a** (0.1 mmol, 1.0 equiv.) → **4a**, 61%, d.r. > 95:5, r.r. > 95:5

A Equivalent and concentration screen

entry	deviations from standard procedure	^{1}H NMR yield
1	0.2 mmol scale	60% (55%[a])
2	**1a** (3.0 equiv.)	61%
3	**1a** (1.0 equiv.), **2a** (1.5 equiv.), 72 h	47%
4	MeCN (0.2 M)	58%
6	**3a** (10 mol%)	48%

B Solvent screen

entry	deviations from standard procedure	^{1}H NMR yield
1	$MeNO_2$ (0.05 M) instead of MeCN	47%
2	DCM (0.1 M) instead of MeCN, **3a** (10 mol%), **1a** (1.5 equiv.)	26%
3	toluene (0.1 M) instead of MeCN, **3a** (10 mol%), **1a** (1.5 equiv.)	9%
4	THF (0.05 M) instead of MeCN	21%
5	DCE/TFE (0.05 M, 1:1 v:v) instead of MeCN	n.o.
6	DCE (0.05 M) instead of MeCN	41%

C Photocatalyst screen

entry	deviations from standard procedure	^{1}H NMR yield
1	Ir-F (1 mol%) instead of **3a**, λ_{max} = 450 nm	n.o.
2	4-CzIPN (5 mol%) instead of **3a**, λ_{max} = 450 nm	n.o.
3	TXT (5 mol%) instead of **3a**, λ_{max} = 405 nm	n.o.
4	$Ir(ppy)_3$ (1 mol%) instead of **3a**, λ_{max} = 450 nm	n.o.
5	$Ir(ppy)_3$ (2 mol%) instead of **3a**, λ_{max} = 450 nm, $Sc(OTf)_3$ (1.0 equiv.)	n.o.
6	$Ir(ppy)_3$ (2 mol%) instead of **3a**, λ_{max} = 450 nm, p-TosH x H_2O (1.0 equiv.)	n.o.
7	**3a** (10 mol%)	48%
8	**3b** (15 mol%) instead of **3a**	52%
9	**3c** (15 mol%) instead of **3a**	< 5%
10	**3d** (10 mol%) instead of **3a**, λ_{max} = 405 nm	n.o.

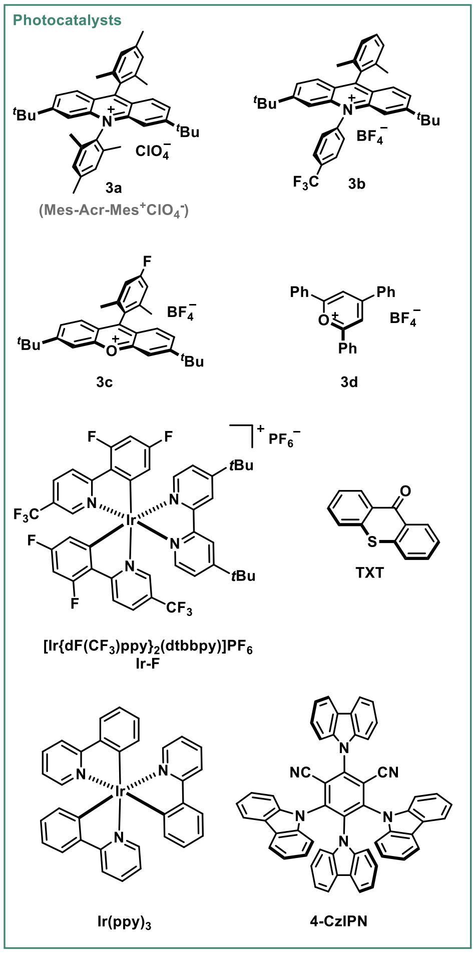

D Additive Screen

entry	deviations from standard procedure	^{1}H NMR yield
1	p-TosH x H_2O (20 mol%)	9%
2	p-TosH x H_2O (2.0 equiv.)	41%
3	$Sc(OTf)_3$ (0.5 equiv.), MeCN (0.1 M), **1a** (1.5 equiv.)	trace
4	PPh_3 (30 mol%)	18%
5	PBu_3 (30 mol%)	6%
6	H_2O (1.0 equiv.)	58%
7	$B(OMe)_3$ (1.0 equiv.)	56%
9	**3d** (10 mol%) instead of **3a**	n.o.

E Control Reactions

entry	deviations from standard procedure	^{1}H NMR yield
1	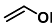 OBu instead of **2a**, 0.2 mmol scale	n.o.
2	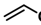 CO_2Et instead of **2a**	n.o.
3	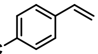 F_3C instead of **2a**	n.o.
4	w.o. **3a**	n.o.
5	**w**/o light irradiation at 60 °C	n.o.

Figure S3. Optimization studies and control experiments. All reactions performed on 0.1 mmol scale following **GP-1** unless otherwise noted. ^{1}H NMR yield was using CH_2Br_2 internal standard. [a]Isolated yield.

2.4 Synthesis of insertion products

2.4.1 N-Heteroarene scope

(*syn*)-*N*-methoxy-*N*-methyl-1-(methylthio)-3-(pyrazin-2-yl)cyclobutane-1-carboxamide (**4a**)

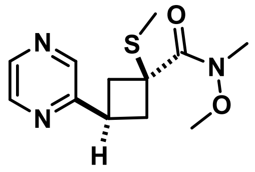

The title compound was synthesized according to **GP-1** using 2-(methylthio)pyrazine (50.5 mg, 0.40 mmol, 2.0 equiv.) and *N*-methoxy-*N*-methylbicyclo[1.1.0]butane-1-carboxamide (28.2 mg, 0.20 mmol, 1.0 equiv.). The crude ^{1}H NMR yield was determined to be 60% (r.r. > 95:5) using CH_2Br_2 as internal standard. Purification by column chromatography (pentane/EtOAc = 25:75) afforded the desired product (29.4 mg, 0.110 mmol, 55%) as a yellow oil.

R$_f$ (pentane/EtOAc = 25:75) = 0.2;

^{1}H NMR (400 MHz, $CDCl_3$) δ 8.55 – 8.46 (m, 2H), 8.41 – 8.33 (m, 1H), 3.74 (s, 3H), 3.55 (tt, *J* = 9.2, 7.5 Hz, 1H), 3.26 – 3.18 (m, 5H), 2.69 – 2.59 (m, 2H), 1.96 (s, 3H);

^{13}C NMR (101 MHz, $CDCl_3$) δ 172.9 (br), 158.8, 144.1, 143.7, 142.4, 60.7, 46.8, 37.5, 33.9 (br), 33.4, 12.3;

HRMS (ESI$^+$): [M+Na]$^+$ 290.0934; found 290.0933.

Note: The obtained regioisomer was determined by HMBC NMR experiment.

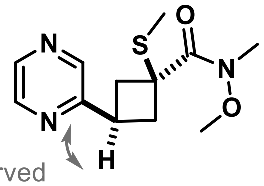

(*syn*)-1-((2-hydroxyethyl)thio)-*N*-methoxy-*N*-methyl-3-(pyrazin-2-yl)cyclobutane-1-carboxamide (**4b**)

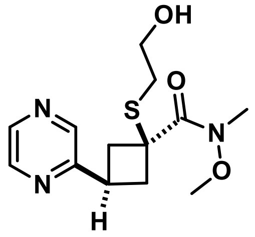

The title compound was synthesized according to **GP-1** using 2-(pyrazin-2-ylthio)ethan-1-ol (62.5 mg, 0.40 mmol, 2.0 equiv.) and *N*-methoxy-*N*-methylbicyclo[1.1.0]butane-1-carboxamide (28.2 mg, 0.20 mmol, 1.0 equiv.). The crude ^{1}H NMR yield was determined to be 48% (r.r. > 95:5) using CH_2Br_2 as internal standard. Purification by column chromatography (100% EtOAc) afforded the desired product (21.2 mg, 0.071 mmol, 36%) as a yellow oil.

R$_f$ (EtOAc) = 0.1;

^{1}H NMR (400 MHz, $CDCl_3$) δ 8.58 – 8.51 (m, 1H), 8.50 (d, *J* = 1.6 Hz, 1H), 8.41 (d, *J* = 2.6 Hz, 1H), 3.78 (s, 3H), 3.68 (t, *J* = 6.0 Hz, 2H), 3.63 – 3.53 (m, 1H), 3.31 – 3.24 (m, 5H), 2.79 – 2.66 (m, 4H), 2.51 (s, 1H, br);

^{13}C NMR (101 MHz, $CDCl_3$) δ 173.8 (br), 158.5, 144.1, 143.9, 142.6, 61.5, 60.9, 46.8, 38.3, 33.9 (br), 33.7, 33.6;

HRMS (ESI^+): $[M+Na]^+$ 320.1039; found 320.1037.

(*syn*)-1-((2-(1,3-dioxoisoindolin-2-yl)ethyl)thio)-*N*-methoxy-*N*-methyl-3-(pyrazin-2-yl)cyclobutane-1-carboxamide (**4c**)

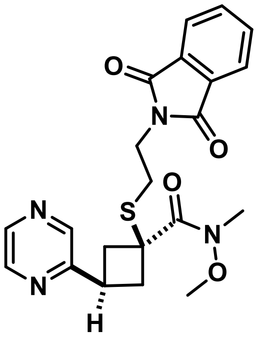

The title compound was synthesized according to **GP-1** using 2-(2-(pyrazin-2-ylthio)ethyl)isoindoline-1,3-dione (114.1 mg, 0.40 mmol, 2.0 equiv.) and *N*-methoxy-*N*-methylbicyclo[1.1.0]butane-1-carboxamide (28.2 mg, 0.20 mmol, 1.0 equiv.). The crude ^{1}H NMR yield was determined to be 37% (r.r. = 90:10) using CH_2Br_2 as internal standard. Purification by column chromatography (100% EtOAc) afforded the desired product (29.3 mg, 0.069 mmol, 34%) as a yellow oil.

R_f (EtOAc) = 0.2;

^{1}H NMR (400 MHz, $CDCl_3$) δ 8.50 – 8.45 (m, 2H), 8.35 (d, J = 2.3 Hz, 1H), 7.84 – 7.78 (m, 2H), 7.73 – 7.66 (m, 2H), 3.82 (t, J = 7.1 Hz, 2H), 3.74 (s, 3H), 3.59 – 3.49 (m, 1H), 3.30 – 3.23 (m, 2H), 3.23 (s, 3H), 2.86 (t, J = 7.1 Hz, 2H), 2.72 – 2.64 (m, 2H);

^{13}C NMR (101 MHz, $CDCl_3$) δ 173.3 (br), 168.0, 158.4, 144.1, 143.7, 142.4, 134.1, 132.2, 123.4, 60.7, 46.9, 38.3, 37.7, 33.8, 28.3;

HRMS (ESI^+): $[M+Na]^+$ 449.1254; found 449.1255.

Note: The ^{13}C signal of the weinreb amide OMe group is not visible due to peak broadening.

(*syn*)-*N*-methoxy-*N*-methyl-3-(pyrazin-2-yl)-1-((3-(trimethoxysilyl)propyl)thio)cyclobutane-1-carboxamide (**4d**)

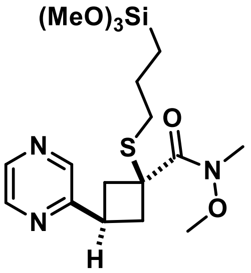

The title compound was synthesized according to **GP-1** using 2-((3-(trimethoxysilyl)propyl)thio)pyrazine (109.8 mg, 0.40 mmol, 2.0 equiv.) and *N*-methoxy-*N*-methylbicyclo[1.1.0]butane-1-carboxamide (28.2 mg, 0.20 mmol, 1.0 equiv.). The crude ^{1}H NMR yield was determined to be 52% (r.r. > 95:5) using CH_2Br_2 as internal standard. Purification by column chromatography (pentane/EtOAc = 20:80) afforded the desired product (25.4 mg, 0.061 mmol, 31%) as a colorless oil.

R_f (EtOAc) = 0.2;

^{1}H NMR (400 MHz, $CDCl_3$) δ 8.57 – 8.49 (m, 2H), 8.39 (d, J = 2.5 Hz, 1H), 3.78 (s, 3H), 3.62 – 3.44 (m, 10H), 3.27 (d, J = 1.9 Hz, 5H), 2.74 – 2.61 (m, 2H), 2.51 (t, J = 7.4 Hz, 2H), 1.68 – 1.54 (m, 2H), 0.74 – 0.61 (m, 2H);

^{13}C NMR (101 MHz, $CDCl_3$) δ 173.5 (br), 158.8, 144.1, 143.7, 142.4, 60.8, 50.6, 46.9, 38.5, 34.0, 32.9, 22.9, 9.0;

HRMS (ESI$^+$): $[M+Na]^+$ 438.1489; found 438.1489.

Note: The ^{13}C signal of the weinreb amide OMe group is not visible due to peak broadening.

(*syn*)-1-(benzylthio)-*N*-methoxy-*N*-methyl-3-(6-phenylpyrazin-2-yl)cyclobutane-1-carboxamide (4e)

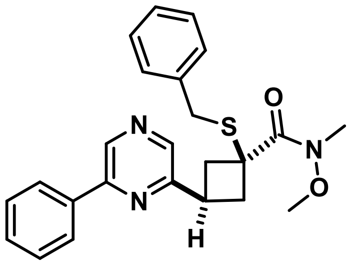

The title compound was synthesized according to **GP-1** using 2-phenyl-6-(phenylthio)pyrazine (55.6 mg, 0.2 mmol, 2.0 equiv.) and *N*-methoxy-*N*-methylbicyclo[1.1.0]butane-1-carboxamide (14.1 mg, 0.10 mmol, 1.0 equiv.). The crude ^{1}H NMR yield was determined to be 36% (r.r. = 88:12) using CH_2Br_2 as internal standard. Purification by column chromatography (pentane/EtOAc = 50:50) afforded the desired product as a regioisomeric mixture (14.2 mg, 0.034 mmol, 34%) as a yellow oil.

R_f (EtOAc) = 0.1;

^{1}H NMR (400 MHz, $CDCl_3$) δ 8.87 (s, 1H), 8.41 (s, 1H), 8.15 – 8.09 (m, 2H), 7.55 – 7.44 (m, 3H), 7.31 – 7.15 (m, 5H), 3.76 (s, 3H), 3.73 (s, 2H), 3.69 – 3.59 (m, 1H), 3.35 – 3.28 (m, 2H), 3.28 (s, 3H), 2.83 – 2.75 (m, 2H);

^{13}C NMR (101 MHz, $CDCl_3$) δ 173.3 (br), 157.7, 151.5, 141.6, 139.4, 137.7, 136.8, 129.9, 129.3, 129.1, 128.5, 127.1, 127.1, 60.8, 47.8, 38.3, 34.6, 34.0;

HRMS (ESI$^+$): $[M+Na]^+$ 442.1560; found 442.1562.

Note: The ^{13}C signal of the weinreb amide OMe group is not visible due to peak broadening.

(*syn*)-1-(cyclohexylthio)-*N*-methoxy-*N*-methyl-3-(pyrazin-2-yl)cyclobutane-1-carboxamide (4f)

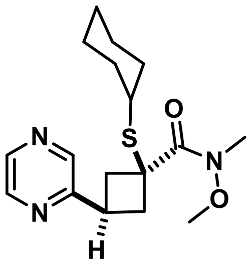

The title compound was synthesized according to **GP-1** using 2-(cyclohexylthio)pyrazine (38.8 mg, 0.2 mmol, 2.0 equiv.) and *N*-methoxy-*N*-methylbicyclo[1.1.0]butane-1-carboxamide (14.1 mg, 0.10 mmol, 1.0 equiv.). The crude ^{1}H NMR yield was determined to be 24% (r.r. > 95:5) using CH_2Br_2 as internal standard. Purification by column chromatography (pentane/EtOAc = 50:50) afforded the desired product (7.5 mg, 0.022 mmol, 22%) as a yellow oil.

R_f (pentane/EtOAc = 50:50) = 0.2;

^{1}H NMR (400 MHz, $CDCl_3$) δ 8.54 (dd, J = 2.6, 1.6 Hz, 1H), 8.52 (d, J = 1.6 Hz, 1H), 8.40 (d, J = 2.5 Hz, 1H), 3.79 (s, 3H), 3.57 (p, J = 8.6 Hz, 1H), 3.37 – 3.23 (m, 5H), 2.77 – 2.63 (m, 3H), 1.94 – 1.82 (m, 2H), 1.72 – 1.61 (m, 3H), 1.59 – 1.46 (m, 1H), 1.37 – 1.22 (m, 4H);

^{13}C NMR (101 MHz, $CDCl_3$) δ 158.8, 144.2, 143.8, 142.4, 60.8, 46.9, 43.3, 39.3, 34.6, 34.4, 26.1, 25.7;

HRMS (ESI$^+$): $[M+Na]^+$ 358.1560; found 358.1560.

Note: The ${}^{13}C$ signal of the weinreb amide carbonyl and OMe group is not visible due to peak broadening.

(*syn*)-methyl 6-(3-(butylthio)-3-(methoxy(methyl)carbamoyl)cyclobutyl)nicotinate (**4h**)

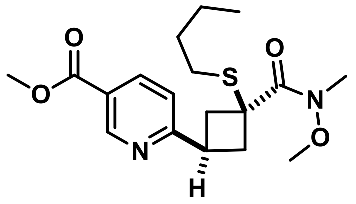

The title compound was synthesized according to **GP-1** using methyl 6-(butylthio)nicotinate (90.0 mg, 0.4 mmol, 2.0 equiv.) and *N*-methoxy-*N*-methylbicyclo[1.1.0]butane-1-carboxamide (28.3 mg, 0.20 mmol, 1.0 equiv.). The crude ${}^{1}H$ NMR yield was determined to be 56% (r.r. = 90:10) using CH_2Br_2 as internal standard. Purification by column chromatography (pentane/EtOAc = 75:25) afforded the desired product (40.0 mg, 0.109 mmol, 55%) as a yellow oil.

R_f (pentane/EtOAc = 75:25) = 0.1;

^{1}H NMR (400 MHz, $CDCl_3$) δ 9.16 (dd, *J* = 2.2, 0.9 Hz, 1H), 8.21 (dd, *J* = 8.2, 2.2 Hz, 1H), 7.33 (d, *J* = 8.1 Hz, 1H), 3.93 (s, 3H), 3.78 (s, 3H), 3.64 – 3.53 (m, 1H), 3.33 – 3.23 (m, 5H), 2.71 – 2.59 (m, 2H), 2.48 (t, *J* = 7.4 Hz, 2H), 1.48 (ddt, *J* = 8.2, 7.1, 6.0 Hz, 2H), 1.40 – 1.27 (m, 2H), 0.86 (t, *J* = 7.3 Hz, 3H);

^{13}C NMR (101 MHz, $CDCl_3$) δ 173.6 (br), 168.1, 166.1, 150.7, 137.5, 123.8, 121.0, 60.8, 52.4, 46.8, 38.6, 36.4, 34.2 (br), 31.4, 29.5, 22.2, 13.7;

HRMS (ESI$^+$): $[M+Na]^+$ 389.1506; found 389.1506.

(*syn*)-3-(5-acetylpyridin-2-yl)-1-(butylthio)-*N*-methoxy-*N*-methylcyclobutane-1-carboxamide

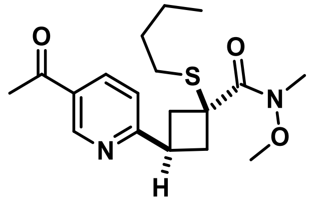

The title compound was synthesized according to **GP-1** using 1-(6-(butylthio)pyridin-3-yl)ethan-1-one (83.6 mg, 0.4 mmol, 2.0 equiv.) and *N*-methoxy-*N*-methylbicyclo[1.1.0]butane-1-carboxamide (28.3 mg, 0.20 mmol, 1.0 equiv.). The crude ${}^{1}H$ NMR yield was determined to be 66% (r.r. = 89:11) using CH_2Br_2 as internal standard. Purification by column chromatography (pentane/EtOAc = 40:60) afforded the desired product (35.9 mg, 0.103 mmol, 51%) as a yellow oil.

R_f (pentane/EtOAc = 40:60) = 0.4;

^{1}H NMR (400 MHz, $CDCl_3$) δ 9.13 (d, *J* = 2.3 Hz, 1H), 8.17 (dd, *J* = 8.2, 2.3 Hz, 1H), 7.37 (d, *J* = 8.2 Hz, 1H), 3.79 (d, *J* = 0.7 Hz, 3H), 3.67 – 3.54 (m, 1H), 3.34 – 3.24 (m, 5H), 2.72 – 2.63 (m, 2H), 2.62 (d, *J* = 0.6 Hz, 3H), 2.48 (d, *J* = 7.1 Hz, 2H), 1.58 (d, *J* = 1.9 Hz, 3H), 1.54 – 1.44 (m, 2H), 1.34 (dq, *J* = 14.5, 7.2 Hz, 2H), 0.87 (t, *J* = 7.3 Hz, 3H);

^{13}C NMR (101 MHz, $CDCl_3$) δ 196.7, 168.3, 149.9, 136.0, 130.4, 121.5, 60.8, 46.8, 38.6, 36.4, 31.4, 29.5, 26.8, 22.3, 13.8;

HRMS (ESI$^+$): $[M+Na]^+$ 373.1556; found 373.1557.

Note: The ${}^{13}C$ signal of the weinreb amide carbonyl and OMe group is not visible due to peak broadening.

6-((*syn*)-3-(butylthio)-3-(methoxy(methyl)carbamoyl)cyclobutyl)nicotinamide (4j)

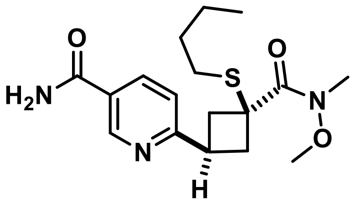

The title compound was synthesized according to **GP-1** using 6-(butylthio)nicotinamide (84.0 mg, 0.4 mmol, 2.0 equiv.) and *N*-methoxy-*N*-methylbicyclo[1.1.0]butane-1-carboxamide (28.3 mg, 0.20 mmol, 1.0 equiv.). The crude ^{1}H NMR yield was determined to be 44% (r.r. > 95:5) using CH_2Br_2 as internal standard. Purification by column chromatography (100% EtOAc) afforded the desired product (31.3 mg, 0.089 mmol, 44%) as a yellow oil.

R$_f$ (EtOAc) = 0.1;

^{1}H NMR (400 MHz, $CDCl_3$) δ 8.97 (d, *J* = 1.5 Hz, 1H), 8.10 (dd, *J* = 8.2, 2.3 Hz, 1H), 7.35 (d, *J* = 8.2 Hz, 1H), 6.37 – 5.89 (m, 2H), 3.78 (s, 3H), 3.66 – 3.52 (m, 1H), 3.35 – 3.21 (m, 5H), 2.70 – 2.60 (m, 2H), 2.47 (d, *J* = 7.3 Hz, 2H), 1.54 – 1.42 (m, 2H), 1.40 – 1.28 (m, 2H), 0.86 (t, *J* = 7.3 Hz, 3H);

^{13}C NMR (101 MHz, $CDCl_3$) δ 173.5 (br), 167.7, 167.3, 148.0, 136.0, 126.8, 121.4, 60.8, 46.7, 38.7, 36.2, 34.1 (br), 31.4, 29.5, 22.2, 13.8;

HRMS (ESI$^+$): [M+Na]$^+$ 374.1509; found 374.1509.

(*syn*)-3-(5-bromopyridin-2-yl)-1-(butylthio)-*N*-methoxy-*N*-methylcyclobutane-1-carboxamide (4k)

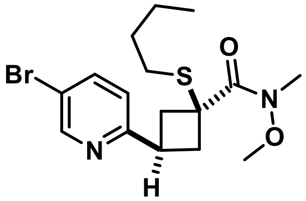

The title compound was synthesized according to **GP-1** using 5-bromo-2-(butylthio)pyridine (97.9 mg, 0.4 mmol, 2.0 equiv.) and *N*-methoxy-*N*-methylbicyclo[1.1.0]butane-1-carboxamide (28.3 mg, 0.20 mmol, 1.0 equiv.). The crude ^{1}H NMR yield was determined to be 45% (r.r. > 95:5) using CH_2Br_2 as internal standard. Purification by column chromatography (pentane/EtOAc = 75:25) afforded the desired product (30.8 mg, 0.078 mmol, 40%) as a yellow oil.

R$_f$ (pentane/EtOAc = 75:25) = 0.4;

^{1}H NMR (400 MHz, $CDCl_3$) δ 8.61 (d, *J* = 2.4 Hz, 1H), 7.72 (dd, *J* = 8.3, 2.4 Hz, 1H), 7.16 (d, *J* = 8.3 Hz, 1H), 3.77 (s, 3H), 3.55 – 3.42 (m, 1H), 3.31 – 3.18 (m, 5H), 2.64 – 2.56 (m, 2H), 2.52 – 2.42 (m, 2H), 1.54 – 1.44 (m, 2H), 1.40 – 1.29 (m, 2H), 0.87 (t, *J* = 7.3 Hz, 3H);

^{13}C NMR (101 MHz, $CDCl_3$) δ 173.6 (br), 162.1, 150.3, 138.9, 122.8, 118.2, 60.8, 46.7, 38.8, 35.7, 34.2 (br), 31.4, 29.5, 22.2, 13.8;

HRMS (ESI$^+$): [M+Na]$^+$ 409.0556; found 409.0557.

(*syn*)-1-(butylthio)-*N*-methoxy-*N*-methyl-3-(5-(trifluoromethyl)pyridin-2-yl)cyclobutane-1-carboxamide (4l)

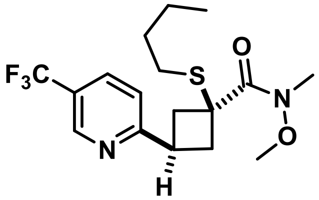

The title compound was synthesized according to **GP-1** using 2-(butylthio)-5-(trifluoromethyl)pyridine (98.0 mg, 0.4 mmol, 2.0 equiv.) and *N*-methoxy-*N*-methylbicyclo[1.1.0]butane-1-carboxamide (28.3 mg, 0.20 mmol, 1.0 equiv.). The crude ^{1}H NMR yield was determined to be 40% (r.r. = 91:9) using CH_2Br_2 as internal standard. Purification by column chromatography (pentane/EtOAc = 75:25) afforded the desired product (26.6 mg, 0.071 mmol, 35%) as a yellow oil.

R$_f$ (pentane/EtOAc = 75:25) = 0.4;

^{1}H NMR (400 MHz, $CDCl_3$) δ 9.16 (dd, *J* = 2.3, 0.9 Hz, 1H), 8.21 (dd, *J* = 8.2, 2.2 Hz, 1H), 7.34 (d, *J* = 8.1 Hz, 1H), 3.93 (s, 3H), 3.77 (s, 3H), 3.64 – 3.53 (m, 1H), 3.31 – 3.24 (m, 6H), 2.70 – 2.62 (m, 2H), 2.50 – 2.45 (m, 2H), 1.52 – 1.43 (m, 2H), 1.33 (t, *J* = 7.2 Hz, 2H), 0.86 (t, *J* = 7.3 Hz, 3H);

^{13}C {^{19}F} NMR (101 MHz, $CDCl_3$) δ 167.5, 146.3, 133.5, 124.4, 123.9, 121.2, 60.8, 46.8, 38.6, 36.2, 31.4, 29.5, 22.2, 13.7;

^{19}F NMR (376 MHz, $CDCl_3$) δ –62.2;

HRMS (ESI$^+$): [M+Na]$^+$ 399.1325; found 399.1326.

Note: The ^{13}C signal of the weinreb amide carbonyl and OMe group is not visible due to peak broadening.

(*syn*)-1-(butylthio)-*N*-methoxy-*N*-methyl-3-(pyridin-2-yl)cyclobutane-1-carboxamide (**4m**)

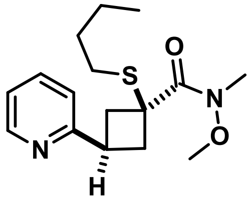

The title compound was synthesized according to **GP-1** using 2-(butylthio)pyridine (66.9 mg, 0.4 mmol, 2.0 equiv.) and *N*-methoxy-*N*-methylbicyclo[1.1.0]butane-1-carboxamide (28.3 mg, 0.20 mmol, 1.0 equiv.). The crude ^{1}H NMR yield was determined to be 28% (r.r. > 95:5) using CH_2Br_2 as internal standard. Purification by column chromatography (pentane/EtOAc = 50:50) afforded the desired product (13.5 mg, 0.044 mmol, 22%) as a yellow oil.

R$_f$ (pentane/EtOAc = 50:50) = 0.2;

^{1}H NMR (400 MHz, $CDCl_3$) δ 8.56 (ddd, *J* = 4.9, 1.8, 0.9 Hz, 1H), 7.61 (td, *J* = 7.7, 1.9 Hz, 1H), 7.29 – 7.22 (m, 1H), 7.10 (ddd, *J* = 7.5, 4.9, 1.2 Hz, 1H), 3.78 (s, 3H), 3.54 (p, *J* = 8.7 Hz, 1H), 3.30 – 3.22 (m, 5H), 2.69 – 2.59 (m, 2H), 2.49 (dd, *J* = 7.9, 6.9 Hz, 2H), 1.49 (ddt, *J* = 8.2, 7.1, 6.0 Hz, 2H), 1.41 – 1.27 (m, 2H), 0.86 (t, *J* = 7.3 Hz, 3H);

^{13}C NMR (101 MHz, $CDCl_3$) δ 173.4 (br), 163.5, 149.3, 136.4, 121.4, 121.3, 60.7, 46.6, 38.9, 36.2, 34.4 (br), 31.5, 29.5, 22.3, 13.8;

HRMS (ESI$^+$): [M+Na]$^+$ 331.1451; found 331.1448.

(*syn*)-*tert*-butyl4-(6-(3-(butylthio)-3-methoxy(methyl)carbamoyl)cyclobutyl)nicotinoyl)-piperazine-1-carboxylatemethyl 6-(3-(butylthio)-3-(methoxy(methyl)carbamoyl)cyclobutyl)-nicotinate (**4n**)

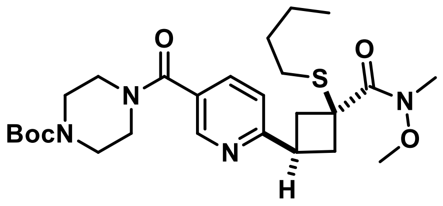

The title compound was synthesized according to **GP-1** using *tert*-butyl-4-(6-(butylthio)nicotinoyl)piperazine-1-carboxylate (165.2 mg, 0.4 mmol, 2.0 equiv.) and *N*-methoxy-*N*-methylbicyclo[1.1.0]butane-1-carboxamide (28.3 mg, 0.20 mmol, 1.0 equiv.). The crude ^{1}H NMR yield was determined to be 56% (r.r. > 95:5) using CH_2Br_2 as internal standard. Purification by column chromatography (pentane/EtOAc = 30:70) afforded the desired product (57.0 mg, 0.109 mmol, 55%) as a yellow oil.

R$_f$ (pentane/EtOAc = 30:70) = 0.1;

^{1}H NMR (400 MHz, $CDCl_3$) δ 8.61 (dd, J = 2.3, 0.9 Hz, 1H), 7.70 (dd, J = 8.0, 2.3 Hz, 1H), 7.32 (dt, J = 8.0, 0.7 Hz, 1H), 3.78 (s, 3H), 3.64 – 3.41 (m, 8H), 3.35 – 3.22 (m, 5H), 2.69 – 2.60 (m, 2H), 2.54 – 2.44 (m, 2H), 1.47 (s, 12H), 1.41 – 1.31 (m, 2H), 0.97 – 0.83 (m, 3H);

^{13}C NMR (101 MHz, $CDCl_3$) δ 173.6 (br), 168.5, 165.4, 154.6, 147.7, 135.8, 128.8, 121.4, 80.6, 60.8, 46.7, 43.9 (br), 38.8, 36.2, 34.2 (br), 31.5, 31.5, 29.5, 28.5, 22.2, 13.8;

HRMS (ESI$^+$): [M+Na]$^+$ 543.2612; found 543.2613.

(*syn*)-(3*aS*,5*R*,6*R*,6*aS*)-5-((*S*)-2,2-dimethyl-1,3-dioxolan-4-yl)-2,2-dimethyltetrahydrofuro[2,3-d][1,3]dioxol-6-yl 6-((1s,3S)-3-(butylthio)-3-(methoxy(methyl)carbamoyl)cyclobutyl)nicotinate (**4o**)

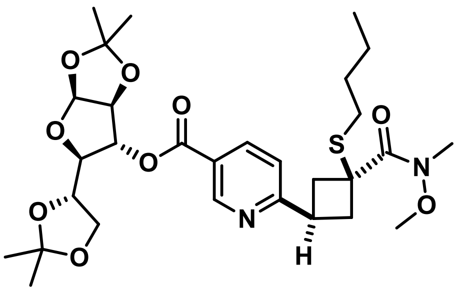

The title compound was synthesized according to **GP-1** using (3*aS*,6*R*,6*aS*)-5-((*R*)-2,2-dimethyl-1,3-dioxolan-4-yl)-2,2-dimethyltetrahydrofuro[2,3-d][1,3]dioxol-6-yl 6-(butylthio)nicotinate (158.0 mg, 0.4 mmol, 2.0 equiv.) and *N*-methoxy-*N*-methylbicyclo[1.1.0]butane-1-carboxamide (28.3 mg, 0.20 mmol, 1.0 equiv.). The crude ^{1}H NMR yield was determined to be 60% (r.r. = 83:17) using CH_2Br_2 as internal standard. Purification by column chromatography (pentane/EtOAc = 60:40) afforded the desired product (major: 59.6 mg, 0.100 mmol; minor: 7.8 mg, 0.013 mmol; in total 0.113 mmol, 56%) as a yellow oil.

R$_f$ (pentane/EtOAc = 60:40) = 0.1;

^{1}H NMR (400 MHz, $CDCl_3$) δ 9.16 (dd, J = 2.3, 0.8 Hz, 1H), 8.20 (dd, J = 8.2, 2.2 Hz, 1H), 7.35 (d, J = 8.2 Hz, 1H), 5.93 (d, J = 3.7 Hz, 1H), 5.49 (d, J = 2.8 Hz, 1H), 4.63 (d, J = 3.7 Hz, 1H), 4.38 – 4.25 (m, 2H), 4.09 (qd, J = 8.7, 5.0 Hz, 2H), 3.78 (s, 3H), 3.66 – 3.53 (m, 1H), 3.33 – 3.23 (m, 5H), 2.66 (d, J = 1.0 Hz, 2H), 2.52 – 2.43 (m, 2H), 1.55 (s, 3H), 1.52 – 1.44 (m, 2H), 1.40 (s, 3H), 1.38 – 1.29 (m, 5H), 1.26 (s, 3H), 0.86 (t, J = 7.3 Hz, 3H);

^{13}C NMR (101 MHz, $CDCl_3$) δ 173.5 (br), 168.7, 164.3, 150.7, 137.5, 123.3, 121.3, 112.6, 109.6, 105.3, 83.5, 80.1, 77.0, 72.7, 67.5, 60.8, 46.8, 38.6, 38.6, 36.4, 34.2 (br), 29.5, 27.0, 26.9, 26.3, 25.3, 22.2, 13.7;

HRMS (ESI$^+$): [M+Na]$^+$ 617.2503; found 617.2500.

Note: NMR data is given for the isolated major regioisomer.

(*syn*)-1-(butylthio)-*N*-methoxy-*N*-methyl-3-(5-(3-(trifluoromethyl)-5,6,7,8-tetrahydro-[1,2,4]triazolo[4,3-*a*]pyrazine-7-carbonyl)pyridin-2-yl)cyclobutane-1-carboxamide (**4p**)

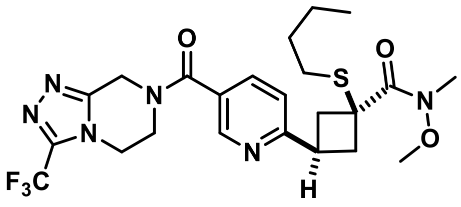

The title compound was synthesized according to **GP-1** using (154.04 mg, 0.4 mmol, 2.0 equiv.) and *N*-methoxy-*N*-methylbicyclo[1.1.0]butane-1-carboxamide (28.3 mg, 0.20 mmol, 1.0 equiv.). The crude ^{1}H NMR yield was determined to be 61% (r.r. = 87:13) using CH_2Br_2 as internal standard. Purification by column chromatography (100% EtOAc) afforded the desired product (63.8 mg, 0.121 mmol, 60%) as a yellow oil.

R$_f$ (100% EtOAc) = 0.2;

^{1}H NMR (500 MHz, $CDCl_3$) δ 8.70 (dd, J = 2.3, 0.8 Hz, 1H), 7.76 (dd, J = 8.1, 2.3 Hz, 1H), 7.40 – 7.36 (m, 1H), 5.06 (s, 2H), 4.26 (t, J = 5.4 Hz, 2H), 4.15 – 4.10 (m, 2H), 3.78 (s, 3H), 3.59 (p, J = 8.6 Hz, 1H), 3.32 – 3.23 (m, 5H), 2.70 – 2.61 (m, 2H), 2.54 – 2.45 (m, 2H), 1.55 – 1.42 (m, 2H), 1.41 – 1.29 (m, 2H), 0.86 (t, J = 7.3 Hz, 3H);

^{13}C {^{19}F} NMR (126 MHz, $CDCl_3$) δ 168.9, 166.7, 149.5, 147.8, 143.8, 135.9, 126.8, 121.5, 118.2, 60.6, 47.3, 46.6, 43.3 (br), 38.6, 36.2, 34.0 (br), 31.3, 29.4, 22.1, 13.6;

^{19}F NMR (470 MHz, $CDCl_3$) δ –63.0;

HRMS (ESI$^+$): [M+Na]$^+$ 549.1866; found 549.1868.

Note: The ^{13}C signal of the weinreb amide carbonyl is not visible due to peak broadening.

(*syn*)-1-(benzylthio)-3-(4-cyanopyridin-2-yl)-*N*-methoxy-*N*-methylcyclobutane-1-carboxamide (**4q**)

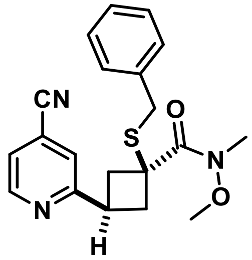

The title compound was synthesized according to **GP-1** using 2-(benzylthio)isonicotinonitrile (90.5 mg, 0.4 mmol, 2.0 equiv.) and *N*-methoxy-*N*-methylbicyclo[1.1.0]butane-1-carboxamide (28.3 mg, 0.20 mmol, 1.0 equiv.). The crude ^{1}H NMR yield was determined to be 25% (r.r. > 95:5) using CH_2Br_2 as internal standard. Purification by column chromatography (pentane/EtOAc = 50:50) afforded the desired product (16.5 mg, 0.045 mmol, 22%) as a yellow oil.

R$_f$ (pentane/EtOAc = 50:50) = 0.2;

^{1}H NMR (400 MHz, $CDCl_3$) δ 8.77 – 8.71 (m, 1H), 7.43 (s, 1H), 7.35 (dd, J = 5.0, 1.5 Hz, 1H), 7.31 – 7.26 (m, 2H), 7.26 – 7.18 (m, 3H), 3.73 (s, 3H), 3.70 (s, 2H), 3.68 – 3.50 (m, 1H), 3.33 – 3.23 (m, 5H), 2.66 – 2.56 (m, 2H);

^{13}C NMR (101 MHz, $CDCl_3$) δ 165.2, 150.2, 137.7, 129.3, 128.6, 127.2, 123.4, 122.8, 120.8, 116.8, 60.8, 47.7, 38.3, 36.1, 34.7, 33.9 (br);

HRMS (ESI$^+$): [M+Na]$^+$ 390.1247; found 390.1247.

Note: The ^{13}C signal of the weinreb amide carbonyl group is not visible due to peak broadening.

(*syn*)-1-(benzylthio)-3-(3-chloropyridin-2-yl)-N-methoxy-N-methylcyclobutane-1-carboxamide (**4r**)

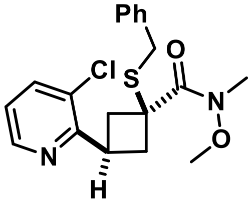

The title compound was synthesized according to **GP-1** using (94.0 mg, 0.4 mmol, 2.0 equiv.) and *N*-methoxy-*N*-methylbicyclo[1.1.0]butane-1-carboxamide (28.3 mg, 0.20 mmol, 1.0 equiv.). The crude ^{1}H NMR yield was determined to be 24% (r.r. > 95:5) using CH_2Br_2 as internal standard. Purification by column chromatography (pentane/EtOAc = 80:20) afforded the desired product (16.0 mg, 0.042 mmol, 21%) as a yellow oil.

R_f (pentane/EtOAc = 80:20) = 0.3;

^{1}H NMR (400 MHz, $CDCl_3$) δ 8.50 (dd, *J* = 4.7, 1.5 Hz, 1H), 7.60 (dd, *J* = 8.0, 1.5 Hz, 1H), 7.27 – 7.16 (m, 5H), 7.11 (ddd, *J* = 8.0, 4.7, 0.6 Hz, 1H), 3.83 (p, *J* = 8.7 Hz, 1H), 3.74 (s, 3H), 3.71 (s, 2H), 3.34 – 3.25 (m, 5H), 2.86 – 2.78 (m, 2H);

^{13}C NMR (101 MHz, $CDCl_3$) δ 159.6, 147.3, 138.1, 137.1, 131.3, 129.6, 128.8, 127.3, 122.8, 61.0, 47.4, 37.6, 34.8, 34.2;

HRMS (ESI$^+$): $[M+Na]^+$ 399.0905; found 399.0903.

Note: The ^{13}C signal of the weinreb amide carbonyl and OMe group is not visible due to peak broadening.

(*syn*)-*N*-methoxy-*N*-methyl-1-(methylthio)-3-(quinolin-2-yl)cyclobutane-1-carboxamide (**4s**)

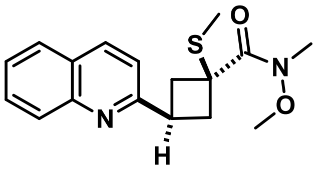

The title compound was synthesized according to **GP-1** using 2-(methylthio)quinoline (70.1 mg, 0.40 mmol, 2.0 equiv.) and *N*-methoxy-*N*-methylbicyclo[1.1.0]butane-1-carboxamide (28.2 mg, 0.20 mmol, 1.0 equiv.). The crude ^{1}H NMR yield was determined to be 44% (r.r. > 95:5) using CH_2Br_2 as internal standard. Purification by column chromatography (pentane/EtOAc = 50:50) afforded the desired product (23.1 mg, 0.073 mmol, 37%) as a yellow oil.

R_f (pentane/EtOAc = 50:50) = 0.3;

^{1}H NMR (400 MHz, $CDCl_3$) δ 8.11 (d, *J* = 8.5 Hz, 1H), 8.08 (d, *J* = 8.5 Hz, 1H), 7.78 (dd, *J* = 8.1, 1.5 Hz, 1H), 7.69 (ddd, *J* = 8.4, 6.9, 1.5 Hz, 1H), 7.53 – 7.45 (m, 2H), 3.79 (s, 3H), 3.80 – 3.66 (m, 1H), 3.40 – 3.30 (m, 2H), 3.29 (s, 3H), 2.83 – 2.73 (m, 2H), 2.02 (s, 3H);

^{13}C NMR (101 MHz, $CDCl_3$) δ 173.2 (br), 163.8, 147.6, 136.7, 129.6, 129.1, 127.6, 127.0, 126.1, 119.8, 60.8, 46.8, 37.7, 36.2, 34.1 (br), 12.4;

HRMS (ESI$^+$): $[M+Na]^+$ 339.1138; found 339.1138.

(*syn*)-1-(((2,2-dimethyl-1,3-dioxolan-4-yl)methyl)thio)-*N*-methoxy-*N*-methyl-3-(quinolin-2-yl)cyclobutane-1-carboxamide (**4t**)

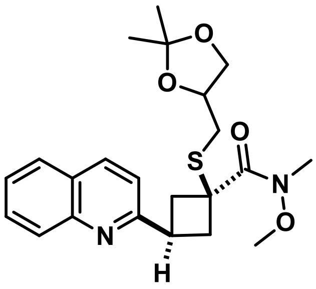

The title compound was synthesized according to **GP-1** using 2-(((2,2-dimethyl-1,3-dioxolan-4-yl)methyl)thio)quinoline (110.1 mg, 0.40 mmol, 2.0 equiv.) and *N*-methoxy-*N*-methylbicyclo[1.1.0]butane-1-carboxamide (28.2 mg, 0.20 mmol, 1.0 equiv.). The crude ^{1}H NMR yield was determined to be 46% (r.r. > 95:5; d.r. 55:45) using CH_2Br_2 as internal standard. Purification by column chromatography (pentane/EtOAc = 50:50) afforded the desired product (38.7 mg, 0.093 mmol, 46%) as a yellow oil.

R_f (pentane/EtOAc = 50:50) = 0.2;

^{1}H NMR (400 MHz, $CDCl_3$) δ 8.03 (dd, *J* = 13.7, 8.5 Hz, 2H), 7.72 (dd, *J* = 8.2, 1.5 Hz, 1H), 7.62 (ddd, *J* = 8.4, 6.9, 1.5 Hz, 1H), 7.47 – 7.34 (m, 2H), 4.18 – 4.07 (m, 1H), 3.95 (dd, *J* = 8.3, 6.1 Hz, 1H), 3.73

(s, 3H), 3.72 – 3.61 (m, 1H), 3.56 (dd, J = 8.3, 6.2 Hz, 1H), 3.35 – 3.24 (m, 2H), 3.22 (s, 3H), 2.79 – 2.68 (m, 3H), 2.58 (dd, J = 12.9, 6.9 Hz, 1H), 1.31 (s, 3H), 1.24 (s, 3H);

^{13}C NMR (101 MHz, $CDCl_3$) δ 173.5 (br), 163.4, 147.5 (br), 136.7, 129.7, 129.0, 127.6, 127.0, 126.1, 119.8, 109.7, 74.8, 68.9, 60.8, 46.7, 38.6, 38.3, 36.5, 34.1 (br), 33.3, 27.0, 25.6;

HRMS (ESI$^+$): [M+Na]$^+$ 439.1662; found 439.1660.

Methyl 3-(((*syn*)-1-(methoxy(methyl)carbamoyl)-3-(quinolin-2-yl)cyclobutyl)thio)propanoate (**4u**)

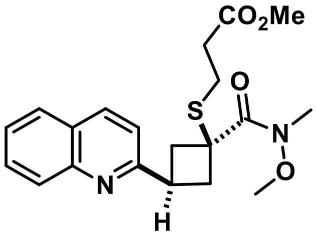

The title compound was synthesized according to **GP-1** using methyl 3-(quinolin-2-ylthio)propanoate (49.5 mg, 0.20 mmol, 2.0 equiv.) and *N*-methoxy-*N*-methylbicyclo[1.1.0]butane-1-carboxamide (14.1 mg, 0.10 mmol, 1.0 equiv.). The crude ^{1}H NMR yield was determined to be 54% (r.r. > 95:5) using CH_2Br_2 as internal standard. Purification by column chromatography (pentane/EtOAc = 50:50) afforded the desired product (17.6 mg, 0.045 mmol, 45%) as a yellow oil.

R_f (pentane/EtOAc = 50:50) = 0.2;

^{1}H NMR (400 MHz, $CDCl_3$) δ 8.13 (d, J = 9.7 Hz, 2H), 7.79 (d, J = 8.2 Hz, 1H), 7.70 (t, J = 7.8 Hz, 1H), 7.55 – 7.41 (m, 2H), 3.86 – 3.72 (m, 4H), 3.64 (s, 3H), 3.39 (dd, J = 12.4, 9.9 Hz, 2H), 3.30 (s, 3H), 2.85 – 2.75 (m, 4H), 2.57 (t, J = 7.2 Hz, 2H);

^{13}C NMR (101 MHz, $CDCl_3$) δ 173.5 (br), 172.3, 163.5, 147.4, 136.9, 129.8, 128.9, 127.6, 127.0, 126.2, 119.8, 60.8, 51.8, 46.9, 38.6, 36.6, 34.5, 34.1 (br), 25.2;

HRMS (ESI$^+$): [M+Na]$^+$ 411.1349; found 411.1348.

(*syn*)-1-(benzylthio)-3-(imidazo[1,2-*b*]pyridazin-6-yl)-*N*-methoxy-*N*-methylcyclobutane-1-carboxamide (**4v**)

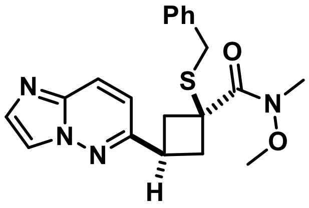

The title compound was synthesized according to **GP-1** using 6-(benzylthio)imidazo[1,2-*b*]pyridazine (48.3 mg, 0.20 mmol, 2.0 equiv.) and *N*-methoxy-*N*-methylbicyclo[1.1.0]butane-1-carboxamide (14.1 mg, 0.10 mmol, 1.0 equiv.). The crude ^{1}H NMR yield was determined to be 26% (r.r. = 67:33) using CH_2Br_2 as internal standard. Purification by column chromatography (EtOAc/MeOH = 97:3) afforded the desired product as a mixture of regioisomers (9.7 mg, 0.025 mmol, 25%) as a yellow oil.

R_f (EtOAc/MeOH = 97:3) = 0.4;

^{1}H NMR (400 MHz, $CDCl_3$) δ 7.72 – 7.66 (m, 1H), 7.58 – 7.50 (m, 1H), 7.47 – 7.41 (m, 2H), 7.36 – 7.20 (m, 4H), 6.85 – 6.75 (m, 1H), 4.48 – 4.36 (m, 2H), 4.13 – 3.82 (m, 1H), 3.70 – 3.61 (m, 3H), 3.25 – 3.11 (m, 3H), 2.95 – 2.81 (m, 1H), 2.68 – 2.63 (m, 2H), 2.57 – 2.45 (m, 1H);

^{13}C NMR (101 MHz, $CDCl_3$) δ 153.2, 153.1, 137.9, 137.6, 136.8, 136.7, 132.6, 132.1, 130.0, 129.2, 129.1, 129.1, 128.8, 128.8, 127.7, 127.6, 124.9, 124.8, 116.9, 116.7, 63.9, 61.6, 61.5, 35.0, 34.9, 33.5, 33.4, 32.6 (br), 30.9, 29.4, 26.7, 26.0;

HRMS (ESI$^+$): [M+Na]$^+$ 405.1361; found 405.1356.

Note: ^{13}C signals are given for the regioisomeric mixture. The ^{13}C signal of the weinreb amide carbonyl is not visible due to peak broadening.

Methyl 3-(((*syn*)-1-(methoxy(methyl)carbamoyl)-3-(quinoxalin-2-yl)cyclobutyl)thio)propanoate (4w)

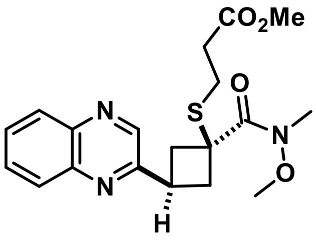

The title compound was synthesized according to **GP-1** using methyl 3-(quinoxalin-2-ylthio)propanoate (99.3 mg, 0.40 mmol, 2.0 equiv.) and *N*-methoxy-*N*-methylbicyclo[1.1.0]butane-1-carboxamide (28.2 mg, 0.20 mmol, 1.0 equiv.). The crude ^{1}H NMR yield was determined to be 53% (r.r. 64:36) using CH_2Br_2 as internal standard. Purification by column chromatography (pentane/EtOAc = 40:60) afforded the desired product (minor regioisomer: 16.7 mg, 0.043 mmol, 21%; major regioisomer: 25.4 mg, 0.065 mmol, 33%) as yellow oils.

R$_f$ (pentane/EtOAc = 50:50) = 0.3 (minor regioisomer), 0.1 (major regioisomer);

^{1}H NMR (400 MHz, $CDCl_3$) δ 8.82 (s, 1H), 8.08 (dd, J = 7.9, 1.8 Hz, 2H), 7.79 – 7.67 (m, 2H), 3.81 – 3.76 (m, 4H), 3.63 (s, 3H), 3.43 – 3.32 (m, 2H), 3.30 (s, 3H), 2.90 – 2.83 (m, 2H), 2.79 (t, J = 7.3 Hz, 2H), 2.56 (t, J = 7.3 Hz, 2H);

^{13}C NMR (101 MHz, $CDCl_3$) δ 172.2, 158.0, 144.8, 142.1, 141.4, 130.2, 129.3, 129.2, 129.2, 60.8, 51.8, 47.1, 38.0, 34.6, 34.4, 34.0, 25.2;

HRMS (ESI$^+$): [M+Na]$^+$ 412.1302; found 412.1302.

Note: The ^{13}C signal of the weinreb amide carbonyl is not visible due to peak broadening. The obtained minor regioisomer was determined by a HMBC NMR experiment.

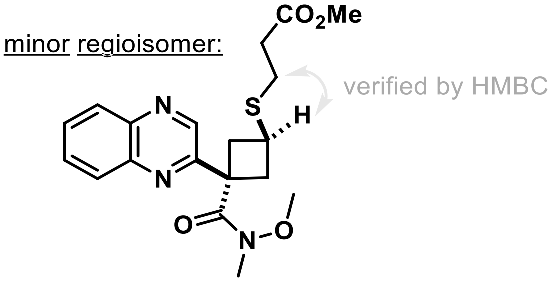

(*syn*)-1-(benzo[*d*]thiazol-2-yl)-*N*-methoxy-*N*-methyl-3-(methylthio)cyclobutane-1-carboxamide (4x)

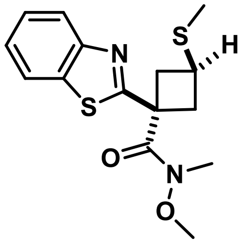

The title compound was synthesized according to **GP-1** using 2-(methylthio)benzo[*d*]thiazole (72.5 mg, 0.40 mmol, 2.0 equiv.) and *N*-methoxy-*N*-methylbicyclo[1.1.0]butane-1-carboxamide (28.2 mg, 0.20 mmol, 1.0 equiv.). The crude ^{1}H NMR yield was determined to be 40% (r.r. = 95:5) using CH_2Br_2 as internal standard. Purification by column chromatography (pentane/EtOAc = 90:10 – 80:20) afforded the desired product (21.3 mg, 0.066 mmol, 33%) as a yellow oil.

R$_f$ (pentane/EtOAc = 80:20) = 0.5;

^{1}H NMR (400 MHz, $CDCl_3$) δ 8.04 – 7.99 (m, 1H), 7.92 – 7.82 (m, 1H), 7.50 – 7.43 (m, 1H), 7.41 – 7.32 (m, 1H), 3.54 – 3.40 (m, 1H), 3.37 – 3.27 (m, 2H), 3.23 (s, 3H), 3.07 (s, 3H), 2.79 – 2.69 (m, 2H), 2.09 (s, 3H);

^{13}C NMR (151 MHz, $CDCl_3$) δ 173.3, 153.2, 135.2, 126.3, 125.2, 123.4, 121.8, 60.2, 49.4, 39.4, 34.6, 33.6, 13.5;

HRMS (ESI$^+$): $[M+Na]^+$ 345.0702; found 345.0700.

Note: The low-field shifted benzo[d]thiazole ^{13}C signal was not visible due to peak broadening. Its occurrence was verified by a HMBC experiment. The regioselectivity was determined by a HMBC experiment.

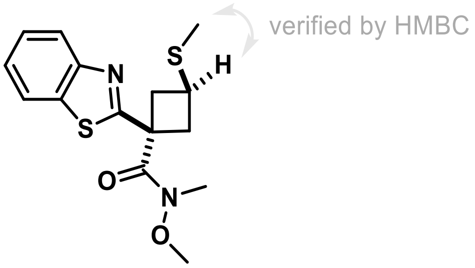

(*syn*)-1-(benzo[*d*]oxazol-2-yl)-*N*-methoxy-*N*-methyl-3-(methylthio)cyclobutane-1-carboxamide (4y)

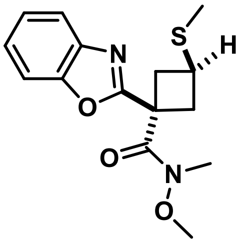

The title compound was synthesized according to **GP-1** using 2-methoxybenzo[*d*]oxazole (59.7 mg, 0.40 mmol, 2.0 equiv.) and *N*-methoxy-*N*-methylbicyclo[1.1.0]butane-1-carboxamide (28.2 mg, 0.20 mmol, 1.0 equiv.). The crude ^{1}H NMR yield was determined to be 44% (r.r. > 95:5) using CH_2Br_2 as internal standard. Purification by column chromatography (pentane/EtOAc = 80:20 – 66:33) afforded the desired product (20.6 mg, 0.067 mmol, 34%) as a colorless oil.

R$_f$ (pentane/EtOAc = 50:50) = 0.4;

^{1}H NMR (400 MHz, $CDCl_3$) δ 7.75 – 7.64 (m, 1H), 7.53 – 7.45 (m, 1H), 7.37 – 7.27 (m, 2H), 3.51 (p, J = 8.6 Hz, 1H), 3.27–3.19 (m, 5H), 3.14 (s, 3H), 2.91–2.80 (m, 2H), 2.10 (s, 3H);

^{13}C NMR (101 MHz, $CDCl_3$) δ 171.2, 166.5, 150.8, 141.3, 125.0, 124.6, 120.1, 110.8, 60.6, 44.5, 37.4, 34.7, 33.5, 13.3;

HRMS (ESI$^+$): $[M+Na]^+$ 329.0930; found 329.0924.

(*syn*)-3-(butylthio)-*N*-methoxy-*N*-methyl-1-(thiazol-2-yl)cyclobutane-1-carboxamide (**4z**)

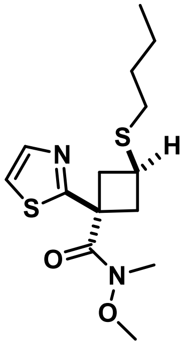

The title compound was synthesized according to **GP-1** using 2-(butylthio)thiazole (69.3 mg, 0.40 mmol, 2.0 equiv.) and *N*-methoxy-*N*-methylbicyclo[1.1.0]butane-1-carboxamide (28.2 mg, 0.20 mmol, 1.0 equiv.). The crude ^{1}H NMR yield was determined to be 28% (r.r. 93:7) using CH_2Br_2 as internal standard. Purification by column chromatography (pentane/EtOAc = 60:40) afforded the desired product (13.6 mg, 0.043 mmol, 21%) as a brown oil.

$\mathbf{R_f}$ (pentane/EtOAc = 60:40) = 0.2;

^{1}H NMR (400 MHz, $CDCl_3$) δ 7.69 (d, *J* = 3.3 Hz, 1H), 7.26 – 7.25 (m, 1H), 3.50 – 3.36 (m, 1H), 3.38 – 3.27 (m, 2H), 3.19 (s, 3H), 3.03 (s, 3H), 2.65 – 2.54 (m, 2H), 2.54 – 2.44 (m, 2H), 1.58 – 1.47 (m, 2H), 1.44 – 1.29 (m, 2H), 0.89 (t, *J* = 7.3 Hz, 3H);

^{13}C NMR (101 MHz, $CDCl_3$) δ 173.4, 142.5, 118.8, 59.9, 49.1, 41.0, 33.5, 32.3, 31.4, 22.2, 13.8;

HRMS (ESI$^+$): [M+Na]$^+$ 337.1015; found 337.1010.

Note: The weinreb amide carbonyl and OMe ^{13}C signals were not visible due to peak broadening.

2.4.2 Bicyclobutane scope

1-((*syn*)-1-(methylthio)-3-(pyrazin-2-yl)cyclobutyl)pentan-1-one (**4aa**)

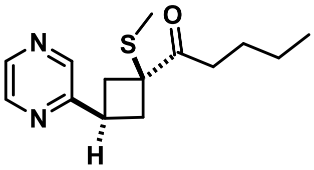

The title compound was synthesized according to **GP-1** using 2-(methylthio)pyrazine (50.5 mg, 0.40 mmol, 2.0 equiv.) and 1-(bicyclo[1.1.0]butan-1-yl)pentan-1-one (27.6 mg, 0.20 mmol, 1.0 equiv.). The crude ^{1}H NMR yield was determined to be 62% (r.r. > 95:5) using CH_2Br_2 as internal standard. Purification by column chromatography (pentane/EtOAc = 80:20) afforded the desired product (30.7 mg, 0.116 mmol, 58%) as a yellow oil.

$\mathbf{R_f}$ (pentane/EtOAc = 80:20) = 0.2;

^{1}H NMR (400 MHz, $CDCl_3$) δ 8.55 (dd, *J* = 2.6, 1.6 Hz, 1H), 8.45 (d, *J* = 1.6 Hz, 1H), 8.41 (d, *J* = 2.5 Hz, 1H), 3.47 (p, *J* = 8.8 Hz, 1H), 3.10 – 3.00 (m, 2H), 2.75 – 2.66 (m, 2H), 2.65 – 2.54 (m, 2H), 1.85 (s, 3H), 1.71 – 1.59 (m, 2H), 1.42 – 1.29 (m, 2H), 0.93 (t, *J* = 7.3 Hz, 3H);

^{13}C NMR (126 MHz, $CDCl_3$) δ 207.4, 158.1, 144.4, 143.7, 142.8, 52.7, 36.4, 35.6, 33.7, 27.3, 22.6, 14.0, 12.3;

HRMS (ESI$^+$): [M+Na]$^+$ 287.1188; found 287.1189.

1-((*syn*)-1-(methylthio)-3-(pyrazin-2-yl)cyclobutyl)-3-phenylpropan-1-one (**4ab**)

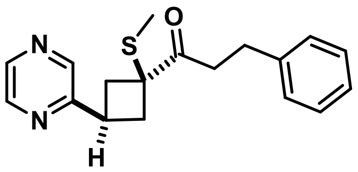

The title compound was synthesized according to **GP-1** using 2-(methylthio)pyrazine (25.2 mg, 0.20 mmol, 2.0 equiv.) and 1-(bicyclo[1.1.0]butan-1-yl)-3-phenylpropan-1-one (18.6 mg, 0.10 mmol, 1.0 equiv.). The crude ^{1}H NMR yield was determined to be 60% (r.r. > 95:5) using CH_2Br_2 as internal standard. Purification by column chromatography (pentane/EtOAc = 75:25) afforded the desired product (19.2 mg, 0.061 mmol, 61%) as a yellow oil.

R$_f$ (pentane/EtOAc = 70:30) = 0.3;

^{1}H NMR (400 MHz, $CDCl_3$) δ 8.54 (dd, J = 2.5, 1.6 Hz, 1H), 8.44 – 8.36 (m, 2H), 7.31 – 7.18 (m, 5H), 3.32 (p, J = 8.9 Hz, 1H), 3.09 – 2.99 (m, 4H), 2.96 – 2.85 (m, 2H), 2.59 – 2.48 (m, 2H), 1.77 (s, 3H);

^{13}C NMR (101 MHz, $CDCl_3$) δ 206.2, 158.0, 144.4, 143.7, 142.8, 141.3, 128.7, 128.6, 126.4, 52.7, 37.7, 36.2, 33.7, 31.2, 12.1;

HRMS (ESI$^+$): [M+Na]$^+$ 335.1189; found 335.1187.

1-(methylthio)-3-(pyrazin-2-yl)cyclobutyl)(phenyl)methanone (**4ac**)

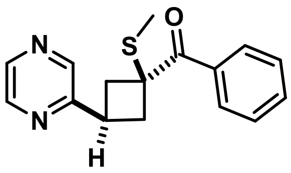

The title compound was synthesized according to **GP-1** using 2-(methylthio)pyrazine (25.2 mg, 0.20 mmol, 2.0 equiv.) and bicyclo[1.1.0]butan-1-yl(phenyl)methanone (15.8 mg, 0.10 mmol, 1.0 equiv.). The crude ^{1}H NMR yield was determined to be 36% (r.r. > 95:5) using CH_2Br_2 as internal standard. Purification by column chromatography (pentane/EtOAc = 80:20) afforded the desired product (8.3 mg, 0.029 mmol, 29%) as a yellow oil.

R$_f$ (pentane/EtOAc = 80:20) = 0.1;

^{1}H NMR (400 MHz, $CDCl_3$) δ 8.58 (dd, J = 2.5, 1.6 Hz, 1H), 8.51 (d, J = 1.6 Hz, 1H), 8.43 (d, J = 2.6 Hz, 1H), 8.14 – 8.07 (m, 2H), 7.59 – 7.50 (m, 1H), 7.49 – 7.40 (m, 2H), 3.58 (p, J = 8.3 Hz, 1H), 3.41 – 3.34 (m, 2H), 2.93 – 2.85 (m, 2H), 1.93 (s, 3H);

^{13}C NMR (126 MHz, $CDCl_3$) δ 196.7, 171.3, 158.3, 144.4, 143.8, 142.7, 133.0, 130.1, 128.3, 50.5, 38.3, 33.9, 12.6;

HRMS (ESI$^+$): [M+Na]$^+$ 307.0876; found 307.0875.

Isobutyl (*syn*)-1-(methylthio)-3-(pyrazin-2-yl)cyclobutane-1-carboxylate (**4ad**)

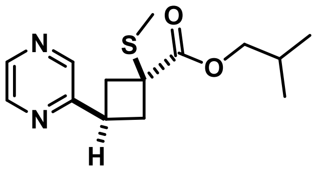

The title compound was synthesized according to **GP-1** using 2-(methylthio)pyrazine (50.5 mg, 0.40 mmol, 2.0 equiv.) and isobutyl bicyclo[1.1.0]butane-1-carboxylate (30.8 mg, 0.20 mmol, 1.0 equiv.). The crude ^{1}H NMR yield was determined to be 48% (r.r. > 95:5) using CH_2Br_2 as internal standard. Purification by column chromatography (pentane/EtOAc = 80:20) afforded the product (23.3 mg, 0.083 mmol, 42%) as a red oil.

R$_f$ (pentane/EtOAc = 70:30) = 0.3;

^{1}H NMR (400 MHz, $CDCl_3$) *δ* 8.55 (dd, *J* = 2.6, 1.5 Hz, 1H), 8.46 (d, *J* = 1.5 Hz, 1H), 8.41 (d, *J* = 2.6 Hz, 1H), 4.01 (d, *J* = 6.6 Hz, 2H), 3.66 (p, *J* = 8.9 Hz, 1H), 3.16 – 3.05 (m, 2H), 2.75 – 2.64 (m, 2H), 2.10 (s, 3H), 2.08 – 1.97 (m, 1H), 0.98 (d, *J* = 6.8 Hz, 6H);

^{13}C NMR (101 MHz, $CDCl_3$) *δ* 173.8, 158.0, 144.4, 143.7, 142.8, 71.7, 47.1, 38.4, 34.1, 28.0, 19.2, 13.0;

HRMS (ESI^+): $[M+Na]^+$ 303.1138; found 303.1138.

Benzyl (*syn*)-1-(methylthio)-3-(pyrazin-2-yl)cyclobutane-1-carboxylate (**4ae**)

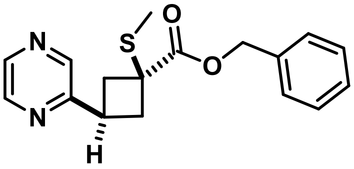

The title compound was synthesized according to **GP-1** using 2-(methylthio)pyrazine (50.5 mg, 0.40 mmol, 2.0 equiv.) and benzyl bicyclo[1.1.0]butane-1-carboxylate (37.6 mg, 0.20 mmol, 1.0 equiv.). Crude ^{1}H NMR yield was determined to be 52% (r.r. > 95:5) using CH_2Br_2 as internal standard. Purification by column chromatography (pentane/EtOAc = 70:30) afforded the product (30.8 mg, 0.098 mmol, 49%) as a red oil.

R_f (pentane/EtOAc = 70:30) = 0.2;

^{1}H NMR (400 MHz, $CDCl_3$) *δ* 8.55 (dd, *J* = 2.6, 1.5 Hz, 1H), 8.45 (d, *J* = 1.5 Hz, 1H), 8.41 (d, *J* = 2.6 Hz, 1H), 7.45 – 7.29 (m, 5H), 5.27 (s, 2H), 3.65 (p, *J* = 8.9 Hz, 1H), 3.13 (ddt, *J* = 12.7, 10.0, 1.5 Hz, 2H), 2.76 – 2.66 (m, 2H), 2.05 (s, 3H);

^{13}C NMR (101 MHz, $CDCl_3$) *δ* 173.6, 157.9, 144.4, 143.7, 142.8, 136.0, 128.7, 128.5, 128.2, 67.3, 47.0, 38.3, 34.1, 13.0;

HRMS (ESI^+): $[M+Na]^+$ 337.0981; found 337.0981.

2-((*syn*)-3-(methoxy(phenyl)methyl)-3-(methylthio)cyclobutyl)pyrazine (**4af**)

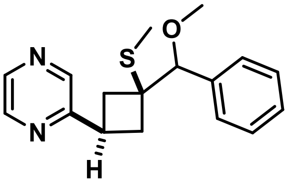

The title compound was synthesized according to **GP-1** using 2-(methylthio)pyrazine (50.5 mg, 0.40 mmol, 2.0 equiv.) and 1-(methoxy(phenyl)methyl)bicyclo[1.1.0]butane (34.8 mg, 0.20 mmol, 1.0 equiv.). The crude ^{1}H NMR yield was determined to be 25% (d.r. 56:44, r.r. > 95:5) using CH_2Br_2 as internal standard. Purification by column chromatography (pentane/EtOAc = 85:15) afforded the product as a mixture of diastereomers (15.2 mg, 0.051 mmol, 25%) as a red oil.

R_f (pentane/EtOAc = 85:15) = 0.2 (major diastereomer), 0.1 (minor diastereomer);

^{1}H NMR (400 MHz, $CDCl_3$) *δ* 8.53 – 8.08 (m, 3H), 7.50 – 7.30 (m, 2H), 7.25 – 7.17 (m, 2H), 6.94 – 6.88 (m, 1H), 4.40 – 4.35 (m, 1H), 3.49 – 3.19 (m, 4H), 3.15 – 2.96 (m, 1H), 2.94 – 2.79 (m, 1H), 2.66 – 2.35 (m, 2H), 1.98 – 1.90 (m, 3H);

^{13}C NMR (101 MHz, $CDCl_3$) *δ* 161.0, 159.5, 144.7, 144.2, 143.7, 143.1, 142.3, 141.7, 138.3, 137.6, 128.6, 128.5, 128.3, 128.2, 128.1, 128.1, 128.0, 127.7, 127.5, 127.2, 88.4, 88.1, 57.7, 57.5, 49.5, 48.2, 37.1, 36.5, 36.5, 35.4, 34.8, 33.1, 13.5, 12.3;

HRMS (ESI^+): $[M+Na]^+$ 323.1189; found 323.1188.

Note: ^{13}C signals are given for the mixture of diastereomers.

(*syn*)-*N*-methoxy-*N*-methyl-1-(methylthio)-3-phenyl-3-(pyrazin-2-yl)cyclobutane-1-carboxamide (**4ag**)

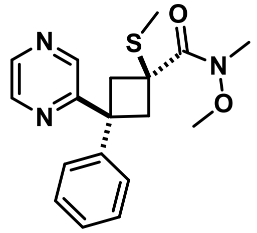

The title compound was synthesized according to **GP-1** using 2-(methylthio)pyrazine (50.5 mg, 0.40 mmol, 2.0 equiv.) and *N*-methoxy-*N*-methyl-3-phenylbicyclo[1.1.0]butane-1-carboxamide (43.5 mg, 0.20 mmol, 1.0 equiv.). Crude ^{1}H NMR yield was determined to be 49% (r.r. 88:12) using CH_2Br_2 as internal standard. Purification by column chromatography (pentane/EtOAc = 50:50 – 40:60) afforded the desired product (25.2 mg, 0.073 mmol, 37%) as a yellow oil.

R_f (pentane/EtOAc = 50:50) = 0.3 (major regioisomer), 0.1 (minor regioisomer);

^{1}H NMR (500 MHz, $CDCl_3$) δ 8.69 (d, *J* = 1.5 Hz, 1H), 8.48 (dd, *J* = 2.6, 1.6 Hz, 1H), 8.33 (d, *J* = 2.6 Hz, 1H), 7.31 – 7.21 (m, 4H), 7.18 – 7.10 (m, 1H), 3.75 (s, 3H), 3.48 – 3.37 (m, 4H), 3.17 (s, 3H), 1.93 (s, 3H);

^{13}C NMR (126 MHz, $CDCl_3$) δ 162.2, 147.5, 143.6, 143.2, 141.5, 128.8, 126.5, 125.9, 60.9, 46.1, 45.6, 42.9, 33.3, 12.6;

HRMS (ESI$^+$): $[M+Na]^+$ 366.1247; found 366.1248.

Note: The ^{13}C signal of the weinreb amide carbonyl is not visible due to peak broadening. The structure of the isolated major regioisomer was determined by HMBC and NOE experiments.

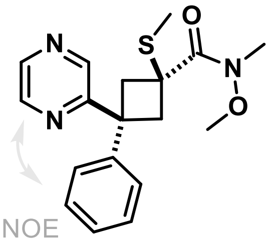

1-(methylthio)-3-phenyl-3-(pyrazin-2-yl)cyclobutyl)pentan-1-one (**4ah**)

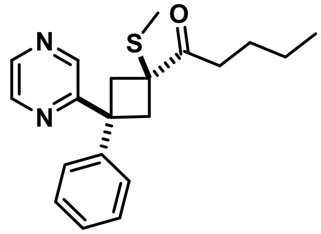

The title compound was synthesized according to **GP-1** using 2-(methylthio)pyrazine (25.2 mg, 0.20 mmol, 2.0 equiv.) and 1-(bicyclo[1.1.0]butan-1-yl)pentan-1-one (21.4 mg, 0.10 mmol, 1.0 equiv.). The crude ^{1}H NMR yield was determined to be 33% (r.r. = 80:20) using CH_2Br_2 as internal standard. Purification by column chromatography (pentane/EtOAc = 80:20) afforded the desired product (10.6 mg, 0.030 mmol, 30%) as a yellow solid.

R_f (pentane/EtOAc = 80:20) = 0.6;

^{1}H NMR (400 MHz, $CDCl_3$) δ 8.55 (d, J = 1.6 Hz, 1H), 8.50 (dd, J = 2.6, 1.5 Hz, 1H), 8.35 (d, J = 2.6 Hz, 1H), 7.28 (d, J = 4.3 Hz, 4H), 7.22 – 7.11 (m, 1H), 3.40 – 3.34 (m, 2H), 3.31 – 3.26 (m, 2H), 2.56 – 2.49 (m, 2H), 1.79 (s, 3H), 1.56 – 1.44 (m, 2H), 1.31 – 1.18 (m, 2H), 0.87 (t, J = 7.3 Hz, 3H);

^{13}C NMR (126 MHz, $CDCl_3$) δ 206.3, 162.0, 146.1, 143.6, 143.3, 141.8, 128.9, 126.8, 126.2, 51.1, 46.0, 41.4, 35.0, 26.8, 22.5, 14.0, 12.5;

HRMS (ESI$^+$): $[M+Na]^+$ 365.1502; found 363.1501.

Phenyl (*syn*)-1-(methylthio)-3-phenyl-3-(pyrazin-2-yl)cyclobutane (**4ai**)

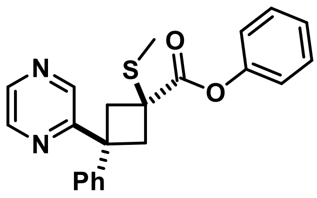

The title compound was synthesized according to **GP-1** using 2-(methylthio)pyrazine (50.4 mg, 0.40 mmol, 2.0 equiv.) and phenyl 3-phenylbicyclo[1.1.0]butane-1-carboxylate (50.0 mg, 0.2 mmol, 1.0 equiv.). The crude ^{1}H NMR yield was determined to be 33% (r.r. = 85:15) using CH_2Br_2 as internal standard. Purification by column chromatography (pentane/EtOAc = 80:20) afforded the desired product (24.4 mg, 0.065 mmol, 32%) as a yellow solid.

R_f (pentane/EtOAc = 80:20) = 0.3;

^{1}H NMR (400 MHz, $CDCl_3$) δ 8.53 (dd, J = 2.6, 1.5 Hz, 1H), 8.46 (d, J = 1.6 Hz, 1H), 8.37 (d, J = 2.6 Hz, 1H), 7.45 – 7.41 (m, 2H), 7.39 – 7.28 (m, 4H), 7.25 – 7.15 (m, 2H), 6.83 – 6.79 (m, 2H), 3.69 – 3.63 (m, 2H), 3.46 – 3.40 (m, 2H), 2.15 (s, 3H);

^{13}C NMR (126 MHz, $CDCl_3$) δ 171.5, 161.6, 150.8, 144.8, 143.5, 143.3, 142.0, 129.5, 129.1, 127.1, 126.8, 126.0, 121.4, 46.9, 45.6, 43.3, 13.3;

HRMS (ESI$^+$): $[M+Na]^+$ 399.1138; found 399.1137.

Methyl (*syn*)-1-(methylthio)-3-phenyl-3-(pyrazin-2-yl)cyclobutane-1-carboxylate (**4aj**)

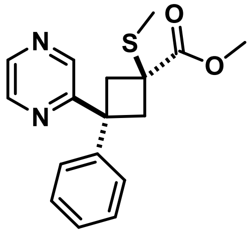

The title compound was synthesized according to **GP-1** using 2-(methylthio)pyrazine (50.5 mg, 0.40 mmol, 2.0 equiv.) and methyl 3-phenylbicyclo[1.1.0]butane-1-carboxylate (37.6 mg, 0.20 mmol, 1.0 equiv.). The crude ^{1}H NMR yield was determined to be 38% (r.r. 87:13) using CH_2Br_2 as internal standard. Purification by column chromatography (pentane/EtOAc = 70:30) afforded the product as a mixture of regioisomers (22.9 mg, 0.073 mmol, 36%) as a yellow oil.

R_f (pentane/EtOAc = 70:30) = 0.3 (major regioisomer), 0.2 (minor regioisomer);

^{1}H NMR (400 MHz, $CDCl_3$) δ 8.49 – 8.43 (m, 2H), 8.29 (d, J = 2.5 Hz, 1H), 7.33 – 7.27 (m, 1H), 7.24 – 7.10 (m, 4H), 3.58 (s, 3H), 3.48 – 3.37 (m, 2H), 3.34 – 3.22 (m, 2H), 1.98 (s, 3H);

^{13}C NMR (101 MHz, $CDCl_3$) δ 173.6, 161.8, 145.8, 143.4, 143.4, 141.8, 128.9, 126.8, 126.3, 52.5, 46.4, 45.2, 42.9, 13.3;

HRMS (ESI$^+$): $[M+Na]^+$ 337.0981; found 337.0981.

Note: The two regioisomers were later separated and recrystallized by diffusion of pentane into concentrated EtOAc solutions to obtain both regioisomers as colorless crystals, that could be taken forward for X-ray crystallography.

2.5 Scale-up experiment

Procedure for the scale-up of the catalytic reaction:

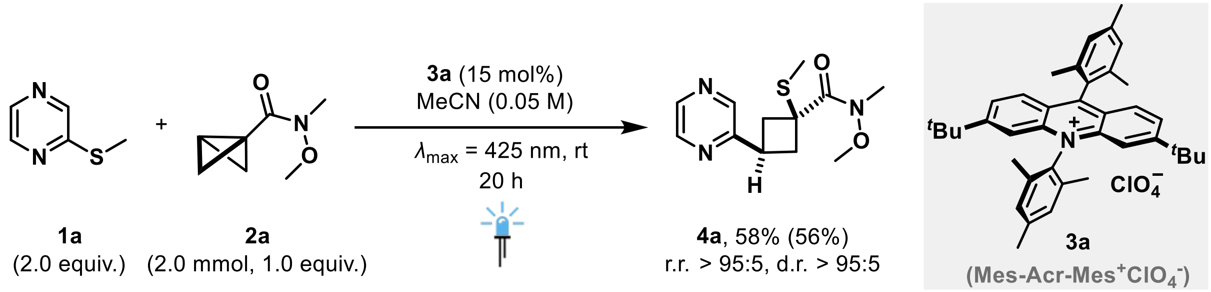

An oven-dried 150 mL Schlenk tube was equipped with a Teflon-coated stir bar and 2-(methylthio)pyrazine (555 mg, 4.40 mmol, 2.0 equiv.) and [MesAcrMes]ClO_4 (207 mg, 0.33 mmol, 15 mol%) were added. The Schlenk tube was evacuated and backfilled with argon three times, before *N*-methoxy-*N*-methylbicyclo[1.1.0]butane-1-carboxamide (311 mg, 2.20 mmol, 1.0 equiv.) and dry MeCN (44 mL, 0.05 M) were added. Then, one aliquot (4 mL) was transferred to another 10 mL Schlenk tube as a control smaple, which was irradiated in the standard photosetup with 425 nm irradiation for 20 h. The Schlenk tube for large scale reaction was irradiated with two lamps in 2 cm distance under 425 nm irradiation for 20 h with fan cooling. Upon completion, the crude reaction mixtures were transferred to a flask and the Schlenk tubes were rinsed with EtOAc. The volatiles were removed in vacuo and crude ^{1}H NMR yield was determined to be 56% (r.r. > 95:5; d.r. > 95:5) using CH_2Br_2 as internal standard. Purification by column chromatography (pentane/EtOAc = 20:80) yielded the desired product (308 mg, 1.15 mmol, 58%) as a yellow oil.

2.6 Product diversification

(*syn*)-*N*-methoxy-*N*-methyl-1-(methylsulfonyl)-3-(pyrazin-2-yl)cyclobutane-1-carboxamide (5)

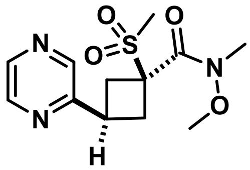

Under air, an 20 mL drum vial was equipped with a stir bar, (*syn*)-*N*-methoxy-*N*-methyl-1-(methylthio)-3-(pyrazin-2-yl)cyclobutane-1-carboxamide (53.5 mg, 0.20 mmol, 1.0 equiv.) and DCM (0.1 M, 2 mL). The solution was cooled to 0 °C and mCPBA (70-75%; 184.1 mg, 0.80 mmol, 4.0 equiv.) was added. The reaction mixture was stirred at 0 °C for 2 h and quenched by the addition of aq. sat. $NaHSO_3$ and diluted with DCM (5 mL). The layers were separated and the aq. layer was extracted with DCM (3 x 5 mL). The combined org. layers were washed with sat. aq. $NaHCO_3$ (5 mL), brine (5 mL) and dried over $MgSO_4$. Upon filtration and concentration in vacuo, column chromatography on silica gel (EtOAc) yielded the product (33.0 mg, 0.110 mmol, 55%) as a colorless oil.

R_f (EtOAc) = 0.1;

^{1}H NMR (400 MHz, $CDCl_3$) δ 8.56 – 8.49 (m, 2H), 8.42 (d, J = 2.5 Hz, 1H), 3.81 (s, 3H), 3.65 (p, J = 9.1 Hz, 1H), 3.37 (s, 3H), 3.28 (d, J = 9.0 Hz, 4H), 2.86 (s, 3H);

^{13}C NMR (101 MHz, $CDCl_3$) δ 166.6, 156.8, 144.2, 143.5, 143.0, 66.2, 61.2, 37.0, 34.4, 32.9, 32.5;

HRMS (ESI$^+$): [M+Na]$^+$ 322.0832; found 322.0831.

***N*-methoxy-*N*-methyl-1-(S-methylsulfonimidoyl)-3-(pyrazin-2-yl)cyclobutane-1-carboxamide** (**6**)

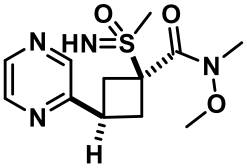

According to a modified literature procedure,[31] to a flask containing a PTFE-coated stir bar was added successively (*syn*)-*N*-methoxy-*N*-methyl-1-(methylthio)-3-(pyrazin-2-yl)cyclobutane-1-carboxamide (26.7 mg, 0.1 mmol, 1.0 equiv.), ammonium carbamate (16.0 mg, 0.20 mmol, 2.0 equiv.) and then MeOH (0.4 mL, 0.25 M). PIDA (80.5 mg, 0.25 mmol, 2.5 equiv.) was added in one portion and the reaction was stirred at 20 °C for 3 h (open flask to the atmosphere). The solvent was removed under reduced pressure and the crude was purified by flash chromatography on silica gel (MeOH/DCM = 1:99 – 10:90) to afford the title compound (23.2 mg, 0.078 mmol, 78%) as a yellow oil.

R_f (MeOH/DCM = 90:10) = 0.2;

^{1}H NMR (400 MHz, $CDCl_3$) δ 8.55 (dd, J = 2.6, 1.6 Hz, 1H), 8.50 (d, J = 1.6 Hz, 1H), 8.44 (d, J = 2.5 Hz, 1H), 3.81 (s, 3H), 3.65 (p, J = 9.0 Hz, 1H), 3.36 j(s, 3H), 3.29 – 3.20 (m, 4H), 2.90 (s, 3H), 1.70 (s, 1H);

^{13}C NMR (101 MHz, $CDCl_3$) δ 168.5 (br), 157.1, 144.3, 143.8, 143.1, 68.3, 61.1, 38.6, 34.1 (br), 32.8 (br), 32.2 (br);

HRMS (ESI$^+$): [M+Na]$^+$ 321.0992, found 321.0989.

Note: The NH signal is not visible in the ^{1}H NMR due to peak broadening.

(*syn*)-1-(methylthio)-3-(pyrazin-2-yl)cyclobutane-1-carbaldehyde (**7**)

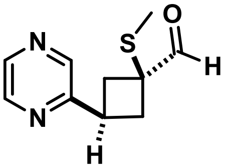

Under air, an 20 mL drum vial was equipped with a stir bar, (*syn*)-*N*-methoxy-*N*-methyl-1-(methylthio)-3-(pyrazin-2-yl)cyclobutane-1-carboxamide (53.5 mg, 0.2 mmol, 1.0 equiv.) and THF (0.1 M, 2 mL). The solution was cooled to 0 °C and $LiAlH_4$ (15.2 mg, 0.4 mmol, 2.0 equiv.) was added portionwise. The reaction mixture was stirred for 3 h at 0 °C, before being quenched by the addition of sat. aq. NH_4Cl (3 mL) and diluted with EtOAc (5 mL). The layers were separated and the aq. layer was extracted with EtOAc (3 x 5 mL). The combined org. layers were washed with brine (3 mL), dired over $MgSO_4$, filtered and concentrated in vacuo. Column chromatography on silica gel (pentane/EtOAc = 50:50) afforded the title compound (28.8 mg, 0.128 mmol, 64%) as a yellow oil.

R_f (pentane/EtOAc = 50:50) = 0.5;

^{1}H NMR (400 MHz, $CDCl_3$) δ 9.38 (s, 1H), 8.56 (dd, J = 2.6, 1.5 Hz, 1H), 8.43 (dd, J = 6.0, 2.1 Hz, 2H), 3.51 (p, J = 8.9 Hz, 1H), 3.00 – 2.90 (m, 2H), 2.60 – 2.49 (m, 2H), 1.85 (s, 3H);

^{13}C NMR (101 MHz, $CDCl_3$) δ 193.4, 157.5, 144.5, 143.7, 143.0, 51.3, 33.8, 33.7, 11.4;

HRMS (ESI$^+$): [M+H]$^+$ 209.0749; found 209.0743.

dibenzyl 2-(3-(methoxy(methyl)carbamoyl)-3-(methylthio)cyclobutyl)piperazine-1,4-dicarboxylate (**8**)

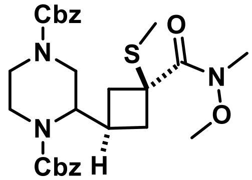

Under air, a 5 mL glass vile with a septum and syringe was equipped with a stir bar and (*syn*)-*N*-methoxy-*N*-methyl-1-(methylthio)-3-(pyrazin-2-yl)cyclobutane-1-carboxamide (53.5 mg, 0.20 mmol, 1.0 equiv.), PtO_2 (4.6 mg, 0.02 mmol, 10 mol%) and acetic acid (2 mL, 0.1 M) were added. The reaction mixture was set under 50 bar hydrogen pressure and heated at 50 °C overnight. After cooling to rt, the reaction mixture was filtered over celite and eluted with acetic acid. Upon thorough concentration in vacuo, the crude material was dissolved in dioxane (0.66 mL, 0.33 M) and dist. H_2O (0.40 mL, 0.5 M) and aq. NaOH (50%) was added until pH ≈ 11. CbzCl (90.2 µL, 0.64 mmol, 3.2 equiv.) was added dropwise at rt and the reaction mixture was stirred at rt for 12h. Upon completion, dist. H_2O (5 mL) was added and the reaction mixture was extracted with EtOAc (3 x 5 mL). The combined organic layers were dried over $MgSO_4$, filtered and concentrated in vacuo. Column chromatography on silica gel (pentane/EtOAc = 50:50) afforded the title compound as a mixture of diastereomers (50.1 mg, 0.092, 46% over 2 steps; d.r. 68:32) as an orange oil.

$\mathbf{R_f}$ (pentane/EtOAc = 50:50) = 0.4;

^{1}H NMR (500 MHz, DMSO-d_6, 373 K) δ 7.42 – 7.26 (m, 10H), 5.19 – 5.04 (m, 4H), 4.42 – 4.04 (m, 1H), 3.98 – 3.79 (m, 3H), 3.67 – 3.58 (m, 3H), 3.15 – 3.05 (m, 3H), 3.00 – 2.61 (m, 3H), 2.47 – 2.27 (m, 2H), 2.08 – 1.95 (m, 1H), 1.96 – 1.86 (m, 3H);

^{13}C NMR (126 MHz, DMSO-d_6, 373 K) δ 171.7, 171.3, 154.4, 154.3, 136.4, 136.3, 127.9, 127.8, 127.8, 127.3, 127.3, 127.3, 127.2, 127.1, 127.0, 127.0, 66.1, 66.1, 66.1, 66.0, 59.8, 59.6, 55.5, 55.2, 45.1, 45.0, 43.2, 43.2, 42.7, 38.6, 38.3, 34.6, 34.4, 33.6, 33.5, 33.0, 32.9, 27.0, 26.4, 11.5, 11.1;

HRMS (ESI$^+$): [M+Na]$^+$ 564.2138; found 564.2141.

Note: The NMR data is given for the mixture of diastereoisomers. The ^{13}C signal of the weinreb amide carbonyl and the OMe group is not visible due to peak broadening.

Benzo[d]thiazol-2-yl((*syn*)-1-(methylthio)-3-(pyrazin-2-yl)cyclobutyl)methanone (**9**)

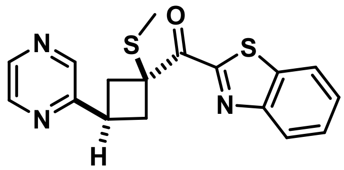

An oven-dried Schlenk tube equipped with a PTFE-coated stirring bar was charged with benzothiazole (18.8 µL, 0.173 mmol, 1.73 equiv.) in THF and cooled to –78 °C. Under a positive argon flow, *n*-BuLi (1.6 M in THF, 1.67 equiv.) was added and stirred for 90 min at –78 °C. A solution of (*syn*)-*N*-methoxy-*N*-methyl-1-(methylthio)-3-(pyrazin-2-yl)cyclobutane-1-carboxamide (0.1 mmol, 1.0 equiv.) in THF (1 mL) was added. The reaction mixture was stirred for 30 min at –78 °C before allowing it to warm up to room temperature for 40 min. The reaction was quenched by the addition of sat. aq. $NaHCO_3$ (3 mL) and diluted with EtOAc (5 mL). The layers were separated and the aq. layer was extracted with EtOAc (3 x 5 mL). The combined org. layers were dried over $MgSO_4$, filtered and concentrated in vacuo. Column chromatography on silica gel (pentane/EtOAc = 80:20) afforded the title compound (24.5 mg, 0.072 mmol, 72%) as a colorless solid.

$\mathbf{R_f}$ (pentane/EtOAc = 80:20) = 0.2;

^{1}H NMR (400 MHz, $CDCl_3$) δ 8.60 (dd, J = 2.6, 1.6 Hz, 1H), 8.55 (d, J = 1.5 Hz, 1H), 8.45 (d, J = 2.5 Hz, 1H), 8.19 – 8.13 (m, 1H), 8.02 – 7.97 (m, 1H), 7.59 – 7.50 (m, 2H), 3.76 – 3.55 (m, 3H), 3.06 – 2.91 (m, 2H), 1.97 (s, 3H);

^{13}C NMR (101 MHz, $CDCl_3$) δ 189.3, 164.2, 158.3, 153.8, 144.4, 143.7, 142.8, 137.0, 127.7, 127.0, 125.9, 122.3, 51.0, 37.4, 34.4, 12.7;

HRMS (ESI^+): $[M+Na]^+$ 364.0549, found 364.0547.

Note: The product was recrystallized by diffusion of pentane into a concentrated EtOAc solution to obtain colorless crystals, that could be taken forward for X-ray crystallography.

2.7 Scope Limitations

2.7.1 (Hetero-)arene scope limitations – A practical guide

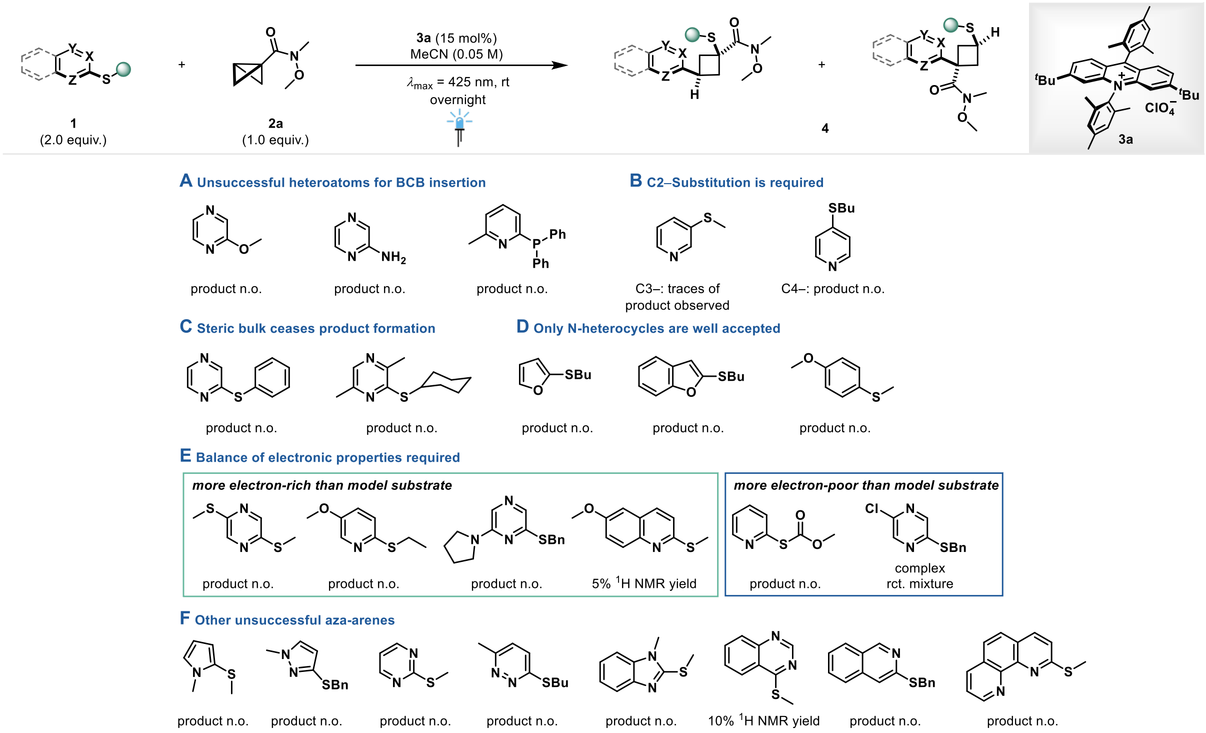

Figure S4. Unsuccessful (hetero-)arene scope entries. Reactions were performed under standard reaction conditions following **GP-1**.

The choice of S-substitution in C2–position of the heterocycle is key to achieve the desired reactivity (**A**, **B**). While sterically demanding substituents on the thioether or the adjacent aza-arene substitution is detrimental for the reactivity (**C**), only N-heterocycles undergo the observed insertion reaction (**D**). Furthermore, a delicate balance of the electronic properties of the aza-arene appears to be important (**E**). While electron-poor substrates may not be feasible for photooxidation, more electron-rich substrates should be less favorable radical acceptors in the plausible intramolecular Minisci-type

attack during the insertion reaction (see computational studies). It should be noted, that a range of other N-heterocycles was also not feasible to undergo the insertion reaction (**F**).

2.7.2 BCB scope limitations

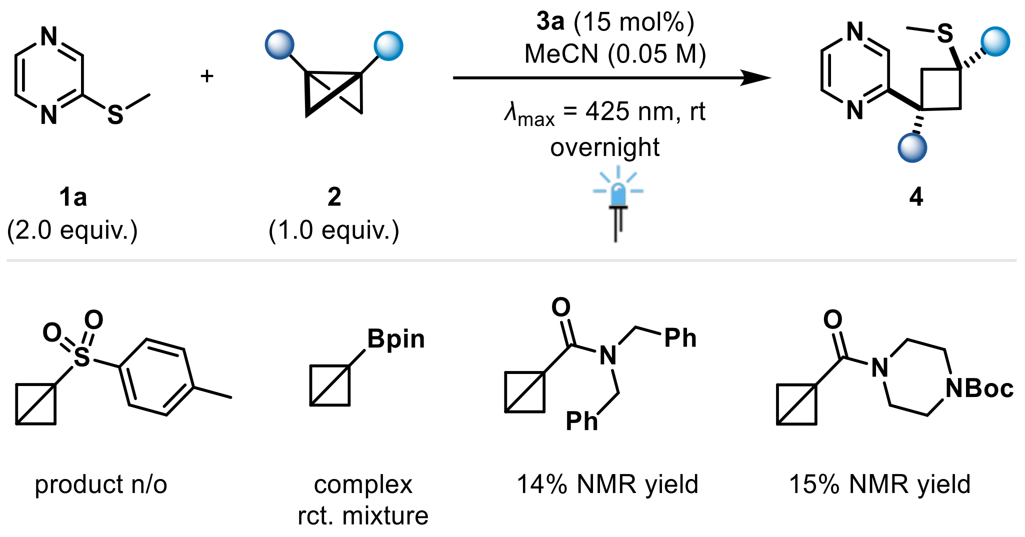

Figure S5. Unsuccessful bicyclo[1.1.0]butane scope entries. Reactions were performed under standard reaction conditions following **GP-1**.

While electron-poor BCB and Bpin-substituted BCB did not yield any insertion product, amide substituted BCBs led to product formation in low yield. We expect that —similar to effects observed for the N-heteroarene scope— steric factors might be most crucial for the reduction in yield compared to weinreb amide substituted BCB **2a**.

2.8 Screening

2.8.1 Sensitivity assessment

A sensitivity assessment was performed following a modified literature procedure.[32] The effect in changing the concentration, addition of water, oxygen quantity, temperature, light intensity and scalability on the product yield were evaluated. Of all the above parameters, only one was varied, while all other parameters were kept constant. The product yield was determined by ^{1}H NMR analysis using CH_2Br_2 as internal standard. The respective deviation in yield was calculated in reference to a control reaction under standard reaction conditions. The results are shown below with the respective radar diagram. Only small deviations from the standard yield are observed for water addition, as well as the variation of concentration, intensity and temperature. Furthermore, only minor deviations are observed for low oxygen and scale up of the reaction, however high oxygen levels led to a complete shutdown of the reactivity.

Table S1. Results of the sensitivity assessment.

Entry	Parameter		Variation	Deviation
1	H_2O		$+H_2O$, V_{H2O} = 10 µL	0%
2	Concentration	Low	V_{rxn} + 10% V_{rxn}	–3.4%
		High	V_{rxn} – 10% V_{rxn}	–1.7%
3	Oxygen	Low	degassed rct mixture	–3.4%
		High	under air	–100%
4	Temperature	Low	*T* – 10 °C	+1.7%
		High	*T* + 10 °C	–1.7%
5	Intensity	Low	d = 8.8 cm	–1.7%
		High	d = 0.5 cm	+3.4%
6	Big Scale		*n* *20	+3.7%

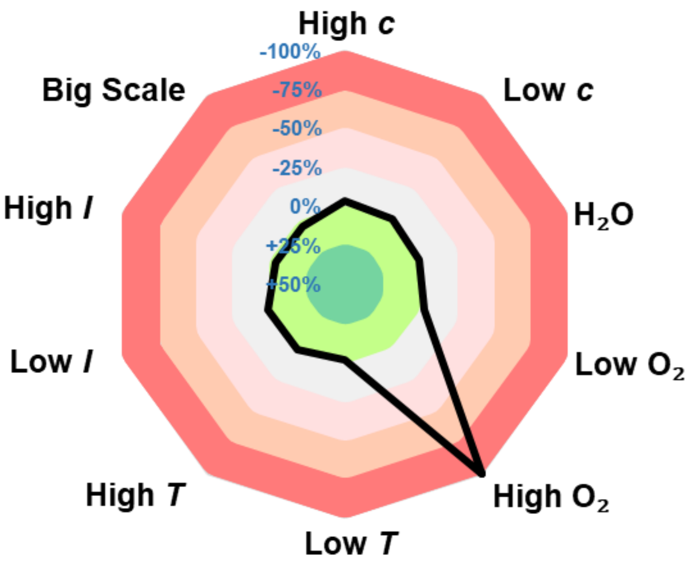

Figure S6. Radar diagram of the sensitivity assessment.

2.8.2 Additive-based robustness screen

The functional group tolerance and robustness of the presented protocol was examined using an additive-based screening procedure.[33] The additive-based robustness screen includes a variety of additives containing different functional groups and heterocycles. Therefore, the stability of additives and tolerance of functional groups in the disclosed methodology is presented. Prior to addition of additives, a stock solution was prepared: To a flame-dried 100 mL Schlenk tube was added $MesAcrMes^+ClO_4^-$ (169.6 mg, 0.27 mmol, 15 mol%), 2-(methylthio)pyrazine (454 mg, 3.6 mmol, 2.0 equiv.). The reaction tube was evacuated and backfilled with argon three times before *N*-methoxy-*N*-methylbicyclo[1.1.0]butane-1-carboxamide (254 mg, 1.8 mmol, 1.0 equiv.) and acetonitrile (36 mL) were added. Fifteen oven-dried 10 mL Schlenk tubes were evacuated and backfilled with argon three

times, before the stock solution (2 mL, approx. 0.1 mmol with respect to the BCB) was added to each reaction tube before the respective (liquid) additive (0.1 mmol, 1.0 equiv.) was added. If the additive was a solid, the additive (0.1 mmol, 1.0 equiv.) was added to the Schlenk tube, the Schlenk tube was evacuated and backfilled with argon three times and stock solution (2 mL, approx. 0.1 mmol with respect to the BCB) was added. In total 15 additives were evaluated. One reaction tube did not contain any additive as a control to check on the change with respect to the change of volume due to the addition of reagents and therefore approximation of appropriate addition volume of the stock solution. All Schlenk tubes were irradiated with 425 nm blue LEDs for 18 h. Mesitylene (13.9 µL, 0.1 mmol) internal standard for the additive recovery and for the product yield were added to reaction mixtures. The additive recovery and product yield was evaluated by GC-FID using prior one-spot calibration of the additive and 5-point calibration of the product.

The color coding is defined as such:

Recovered additive: green (>66%), yellow (33-66%), red (<33%).

Product yield: green (>36%), yellow (18-36%), red (<18%).

Table S2. Results of the additive-based robustness screen.

Entry	Additive	Product yield / %	Additive recovery / %	Entry	Additive	Product yield / %	Additive recovery / %
1	none	54		9	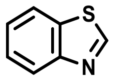	47	81
2	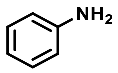	16	96	10	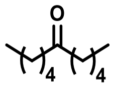	54	92
3	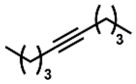	56	82	11	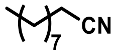	8	91
4	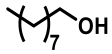	53	87	12	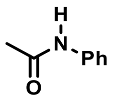	38	72
5	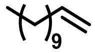	45	95	13	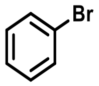	46	95
6	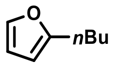	16	22	14	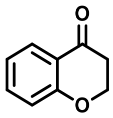	54	54

7	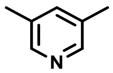	24	80
8	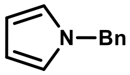	37	58
15	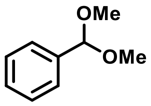	52	99
16	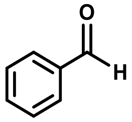	33	59

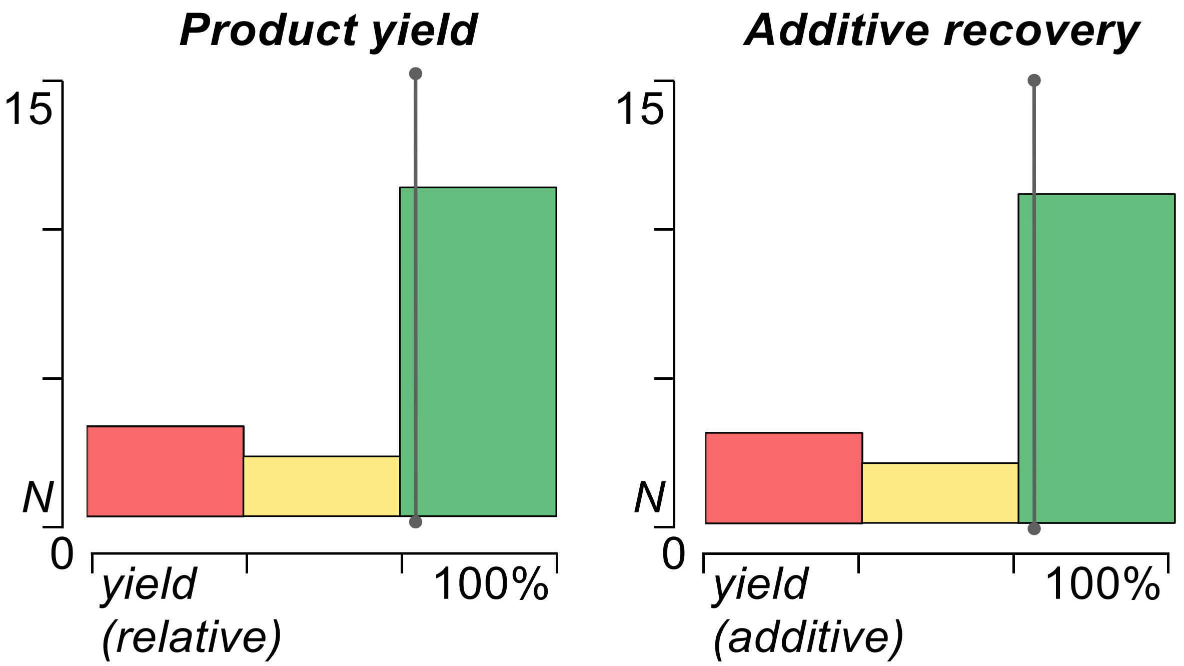

Figure S7. Graphical outline of the robustness screen. Left: Influence of the additives on the product yield (normed on the control reaction); right: recovery of the additive; grey Line: average (normed on the control reaction for product yield). The height of each bar represents the frequency of screen entries occurring within each of the three colored segments.

2.9 Crystallographic data

X-Ray diffraction: Data sets for compounds **4aj (major regioisomer)**, **4aj (minor regioisomer)** and **9** were collected with a Bruker D8 Venture Photon III Diffractometer. Programs used: data collection: *APEX4* Version 2021.4-0 [1] (Bruker AXS Inc., **2021**); cell refinement: *SAINT* Version 8.40B (Bruker AXS Inc., **2021**); data reduction: *SAINT* Version 8.40B (Bruker AXS Inc., **2021**); absorption correction, *SADABS* Version 2016/2 (Bruker AXS Inc., **2021**); structure solution *SHELXT*-Version 2018-3 [2] (Sheldrick, G. M. *Acta Cryst.,* **2015**, *A71*, 3-8); structure refinement *SHELXL*- Version 2018-3 [3] (Sheldrick, G. M. *Acta Cryst.,* **2015**, *C71* (1), 3-8) and graphics, *XP* [4] (Version 5.1, Bruker AXS Inc., Madison, Wisconsin, USA, **1998**). *R*-values are given for observed reflections, and wR^2 values are given for all reflections.
Exceptions and special features: For compound **4aj (minor regioisomer)** the phenyl group and the methoxycarbonyl group were found disordered over two positions in the asymmetric unit. Several restraints (SADI, SAME, ISOR and SIMU) were used in order to improve refinement stability.

X-ray crystal structure analysis of 4aj (major regioisomer): A colorless, needle-like specimen of $C_{17}H_{18}N_2O_2S$, approximate dimensions 0.051 mm x 0.081 mm x 0.279 mm, was used for the X-ray crystallographic analysis. The X-ray intensity data were measured on a single crystal diffractometer Bruker D8 Venture Photon III system equipped with a micro focus tube Cu ImS (CuKα, λ = 1.54178 Å) and a MX mirror monochromator. A total of 1298 frames were collected. The total exposure time was 17.82 hours. The frames were integrated with the Bruker SAINT software package using a wide-frame algorithm. The integration of the data using a triclinic unit cell yielded a total of 11242 reflections to a maximum θ angle of 66.67° (0.84 Å resolution), of which 2710 were independent (average redundancy 4.148, completeness = 99.2%, R_{int} = 3.61%, R_{sig} = 3.10%) and 2468 (91.07%) were greater than 2σ(F^2). The final cell constants of a = 6.00840(10) Å, b = 9.2515(2) Å, c = 15.0308(3) Å, α = 102.5420(10)°, β = 99.9030(10)°, γ = 102.1910(10)°, volume = 776.13(3) $Å^3$, are based upon the refinement of the XYZ-centroids of 7285 reflections above 20 σ(I) with 10.39° < 2θ < 133.3°. Data were corrected for absorption effects using the multi-scan method (SADABS). The ratio of minimum to maximum apparent transmission was 0.843. The calculated minimum and maximum transmission coefficients (based on crystal size) are 0.6160 and 0.9080. The structure was solved and refined using the Bruker SHELXTL Software Package, using the space group *P*-1, with Z = 2 for the formula unit, $C_{17}H_{18}N_2O_2S$. The final anisotropic full-matrix least-squares refinement on F^2 with 201 variables converged at R1 = 3.36%, for the observed data and wR2 = 8.75% for all data. The goodness-of-fit was 1.058. The largest peak in the final difference electron density synthesis was 0.310 $e^-/Å^3$ and the largest hole was -0.260 $e^-/Å^3$ with an RMS deviation of 0.048 $e^-/Å^3$. On the basis of the final model, the calculated density was 1.345 g/cm^3 and F(000), 332 e^-. CCDC Nr.: 2369683.

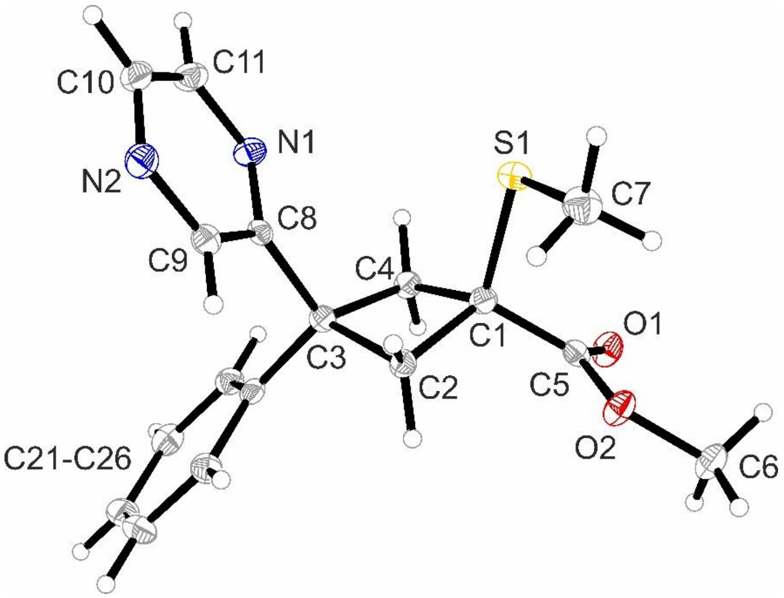

Figure S8: Crystal structure of compound **4aj (major regioisomer)**.
Thermal ellipsoids are shown at 50% probability.

X-ray crystal structure analysis of 4aj (minor regioisomer): A colorless, prism-like specimen of $C_{17}H_{18}N_2O_2S$, approximate dimensions 0.057 mm x 0.073 mm x 0.185 mm, was used for the X-ray crystallographic analysis. The X-ray intensity data were measured on a single crystal diffractometer Bruker D8 Venture Photon III system equipped with a micro focus tube Cu ImS (CuKα, λ = 1.54178 Å) and a MX mirror monochromator. A total of 1429 frames were collected. The total exposure time was 17.23 hours. The frames were integrated with the Bruker SAINT software package using a wide-frame algorithm. The integration of the data using a monoclinic unit cell yielded a total of 28260 reflections to a maximum θ angle of 66.74° (0.84 Å resolution), of which 2755 were independent (average redundancy 10.258, completeness = 99.8%, R_{int} = 5.22%, R_{sig} = 2.40%) and 2397 (87.01%) were greater than 2σ(F^2). The final cell constants of a = 5.75790(10) Å, b = 16.2633(4) Å, c = 16.7223(4) Å, β = 95.8740(10)°, volume = 1557.70(6) $Å^3$, are based upon the refinement of the XYZ-centroids of 9899 reflections above 20 σ(I) with 7.602° < 2θ < 133.3°. Data were corrected for absorption effects using the multi-scan method (SADABS). The ratio of minimum to maximum apparent transmission was 0.823. The calculated minimum and maximum transmission coefficients (based on crystal size) are 0.7180 and 0.8990. The structure was solved and refined using the Bruker SHELXTL Software Package, using the space group *P*2₁/*c*, with Z = 4 for the formula unit, $C_{17}H_{18}N_2O_2S$. The final anisotropic full-matrix least-squares refinement on F^2 with 270 variables converged at R1 = 3.21%, for the observed data and wR2 = 7.96% for all data. The goodness-of-fit was 1.039. The largest peak in the final difference electron density synthesis was 0.263 $e^-/Å^3$ and the largest hole was -0.260 $e^-/Å^3$ with an RMS deviation of 0.044 $e^-/Å^3$. On the basis of the final model, the calculated density was 1.341 g/cm^3 and F(000), 664 e^-. CCDC Nr.: 2369684.

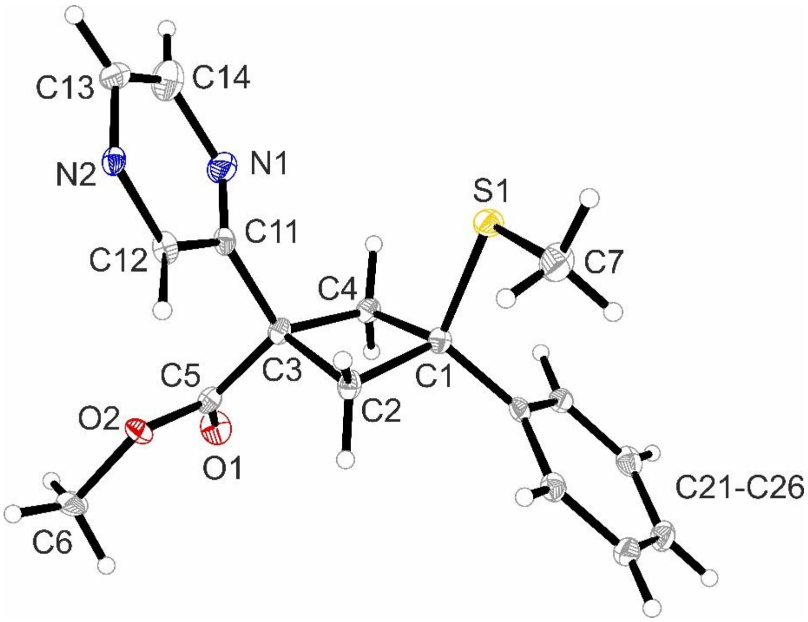

Figure S9: Crystal structure of compound **4aj (minor regioisomer)**.
Thermal ellipsoids are shown at 50% probability.

X-ray crystal structure analysis of 9: A colorless, prism-like specimen of $C_{17}H_{15}N_3OS_2$, approximate dimensions 0.039 mm x 0.157 mm x 0.178 mm, was used for the X-ray crystallographic analysis. The X-ray intensity data were measured on a single crystal diffractometer Bruker D8 Venture Photon III system equipped with a micro focus tube Mo ImS (MoKα, λ = 0.71073 Å) and a MX mirror monochromator. A total of 681 frames were collected. The total exposure time was 4.92 hours. The frames were integrated with the Bruker SAINT software package using a narrow-frame algorithm. The integration of the data using an orthorhombic unit cell yielded a total of 35150 reflections to a maximum θ angle of 27.51° (0.77 Å resolution), of which 3686 were independent (average redundancy 9.536, completeness = 99.9%, R_{int} = 6.27%, R_{sig} = 3.44%) and 2932 (79.54%) were greater than $2\sigma(F^2)$. The final cell constants of a = 10.7580(4) Å, b = 7.7718(3) Å, c = 38.4713(17) Å, volume = 3216.5(2) $Å^3$, are based upon the refinement of the XYZ-centroids of 6252 reflections above 20 σ(I) with 4.338° < 2θ < 54.24°. Data were corrected for absorption effects using the multi-scan method (SADABS). The ratio of minimum to maximum apparent transmission was 0.843. The calculated minimum and maximum transmission coefficients (based on crystal size) are 0.9420 and 0.9870. The structure was solved and refined using the Bruker SHELXTL Software Package, using the space group *Pbca*, with Z = 8 for the formula unit, $C_{17}H_{15}N_3OS_2$. The final anisotropic full-matrix least-squares refinement on F^2 with 209 variables converged at R1 = 3.73%, for the observed data and wR2 = 10.41% for all data. The goodness-of-fit was 1.062. The largest peak in the final difference electron density synthesis was 0.346 $e^-/Å^3$ and the largest hole was -0.323 $e^-/Å^3$ with an RMS deviation of 0.060 $e^-/Å^3$. On the basis of the final model, the calculated density was 1.410 g/cm^3 and F(000), 1424 e^-. CCDC Nr.: 2369685.

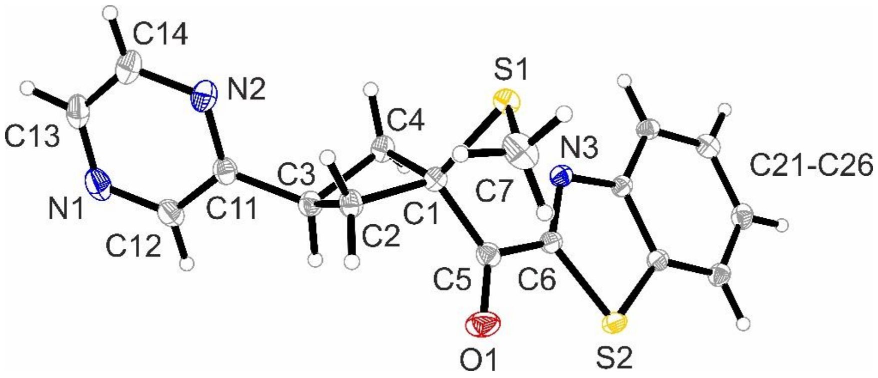

Figure S10: Crystal structure of compound **9**.
Thermal ellipsoids are shown at 50% probability.

3 Mechanistic Analysis

3.1 Cyclic voltammetry studies

In order to determine the redox potentials of the reagents, cyclic voltammetry studies (CV) with a standard three electrode set up was conducted. The set up was equipped with a reference electrode (Ag/AgCl; 2 M LiCl solution in EtOH), a working electrode (3 mm glassy carbon disc electrode) and counter electrode (platinum wire) on a Metrohm µ-Stat-i 400s potentiostat (*Metrohm*, Steinhagen, Germany). For the measurements a solution of *n*-$Bu_4N^+PF_6^-$ (0.05 M) in MeCN (dry, LC-MS grade) was prepared as electrolyte. Prior to the measurement the solution was degassed inside the setup, while bubbling argon through it. First a blank CV and ferrocene was measured, followed by the first substrate (0.05 mmol), which was dissolved in the previously prepared electrolyte solution (20 mL, 0.05 M). The process was repeated independently for all the reagents of the described reactions. In between the measurements all electrodes and the solvent bulb were washed with MeCN (LC-MS grade). No additional blank CV was performed. All CV studies were carried out under argon atmosphere at rt and with a scan rate of 0.1 mV·s^{-1}. Fc^+/Fc was found to be +0.505 V vs Ag/Ag^+ (2 M LiCl in EtOH) and hence the CV measurements of starting materials were referenced against Fc^+/Fc.

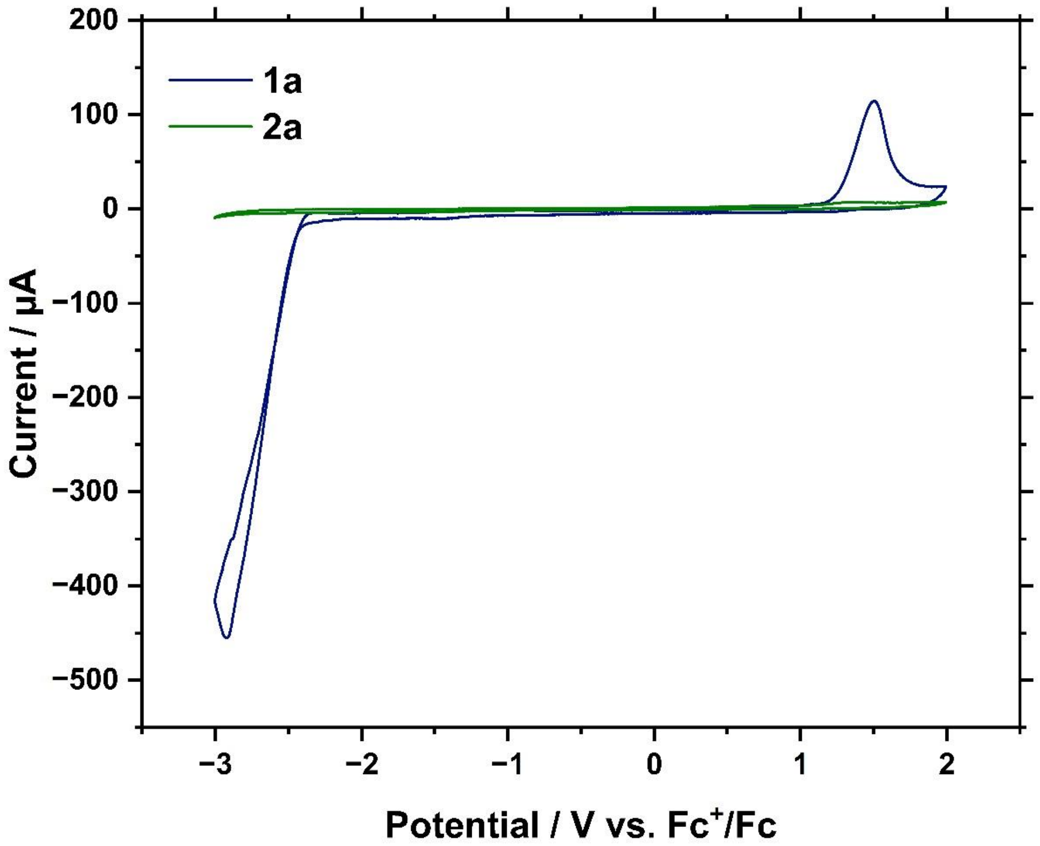

Figure S11. CV spectrum of **1a** and **2a** referenced against Fc^+/Fc.

For comparison of the result, the given potential for 2-(methylthio)pyrazine was recalculated according to the determined half redox potential of the saturated calomel electrode (in MeCN) as internal standard by equation:[34]

$$E_{1/2}'\text{(vs SCE)} = E_{1/2}\text{ (vs Fc/Fc}^{+}\text{)} + 0.382\text{ V}$$

Hence, the oxidation potential of 2-(methylthio)pyrazine is calculated to be $E_{1/2}$ = +1.89 V vs SCE.

Comment: While no obvious redox interactions of the BCB are observed, an irreversible oxidation as indicated above and an irreversible reduction at $E_{1/2}$ = –2.54 V vs SCE. While the reduction of 2-(methylthio)pyrazine should not feasible with the utilized photoexcited $MesAcrMes^+$ photocatalyst, the oxidation of 2-(methylthio)pyrazine is expected to be exergonic ($E_{1/2}$ [PC*]/[PC$^-$] = +2.00 V vs. SCE).[1]

3.2 UV/vis absorption spectroscopy

The UV/vis absorption spectra were recorded on a Jasco V-730 spectrophotometer, which is equipped with a temperature control unit at 25 °C. The samples were measured in Starna® fluorescence quartz cuvettes (type: 29-F, chamber volume = 1.400 mL, H × W × D = 48 mm × 12.5 mm × 12.5 mm, path length = 10 mm).

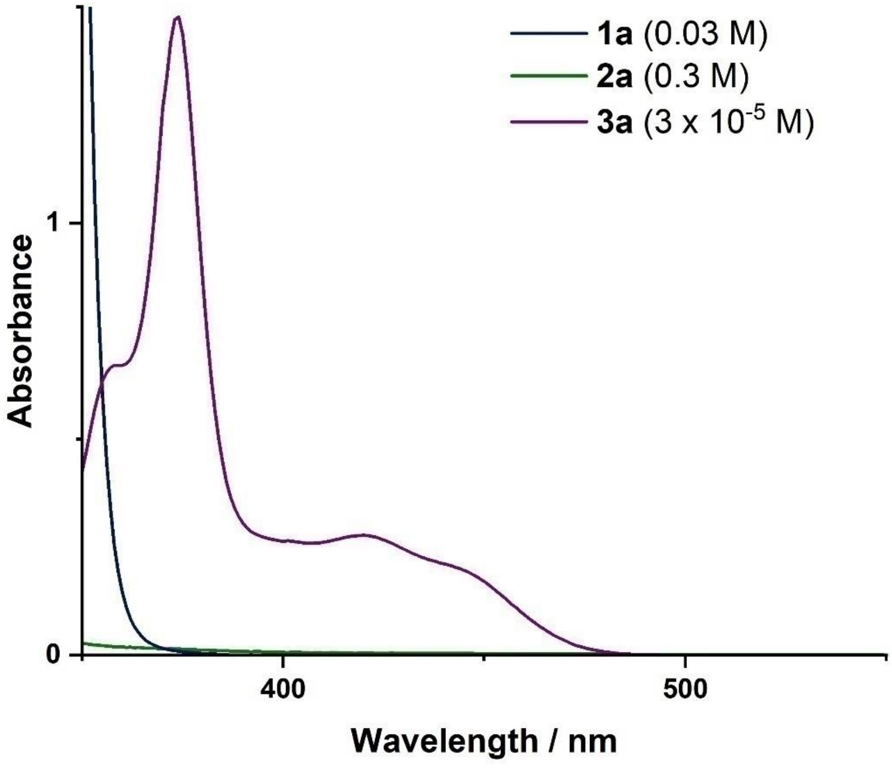

Figure S12. UV/vis absorption of starting materials and photocatalyst.

Comment: Only the photocatalyst absorbs at 425 nm (the operational wavelength).

3.3 Spectroelectrochemical measurement

The spectroelectrochemical measurements were carried out using an AVA-Light DH-S-BAL Light Source, an AVASpec2024 spectrometer and a Metrohm PGSTAT204 potentiostat. Acetonitrile was previously degassed, dried over mole sieves and stored in the glove box. Compound **1a** was dissolved in the electrolyte (0.1 M $TBA^+PF_6^-$ in acetoniltrile) to deliver a concentration of 5 mM and the closed measurement cell was charged with 3 mL of the solution. A platinum gauze electrode was used as a working electrode and the potential was monitored with a pseudo Ag/AgCl reference electrode (previously referenced to ferrocene). Glassy carbon press-fitted into PEEK was used as a counter electrode. The scan rate was set to 50 mV/s and the UV/vis measurements (200 – 800 nm) were taken every 0.0244 V.

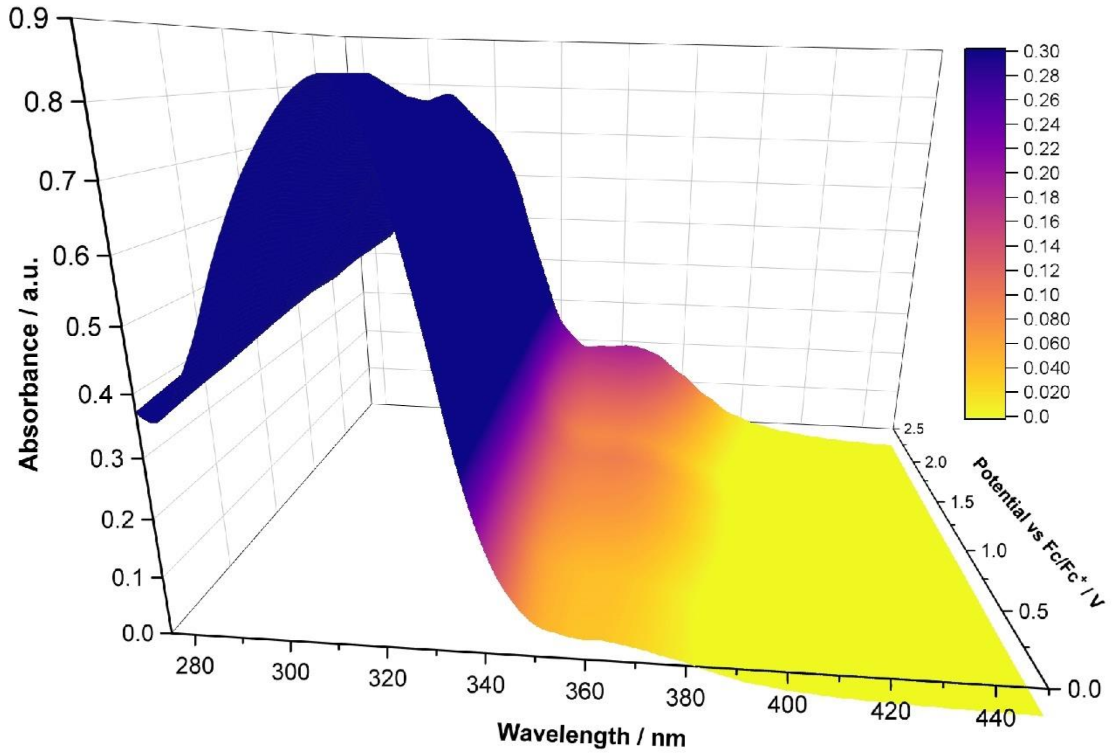

Figure S13. Spectroelectrochemical measurement of **1a**.

Comment: A new absorption band is appearing at approx. 360 nm with increasing the potential, especially in the range where an irreversible oxidation event of **1a** is observed for the CV studies (see **3.1**). This hints towards the intermediacy of the radical cation of **1a**.

3.4 Stern–Volmer quenching studies

Stern–Volmer luminescence quenching analysis was carried out to identify the potential quenchers among the applied reactants, which can hint towards the most feasible initiation step of the overall reaction. For this, the luminescence of the excited photocatalyst ($MesAcrMes^+ClO_4^-$) was measured in the presence of varying concentrations of starting materials as potential quenchers.

The quenching studies were performed on a JASCO FP-8300 spectrofluorometer using Starna® fluorescence quartz cuvettes (type: 29-F, chamber volume = 1.400 mL, H × W × D = 48 mm × 12.5 mm × 12.5 mm, path length = 10 mm). The following parameters were set: data interval = 0.5 nm, scan-speed = 1000 nm/min, excitation wavelength λex = 420 nm, measured luminescence wavelength λ = 488 nm. All samples were prepared in an argon-filled glovebox with degassed and dry MeCN. Stock solution for both the photocatalyst (2.0×10^{-5} M) and the reactants (0.25 M and 0.025 M) were prepared. Inside an argon-filled glovebox, a mixture of the photocatalyst and varying amounts of the quencher were prepared, to obtain a 2.0×10^{-6} M photocatalyst concentration and quencher concentrations as indicated in **Figure S14.** for the mixtures (V = 1 mL).

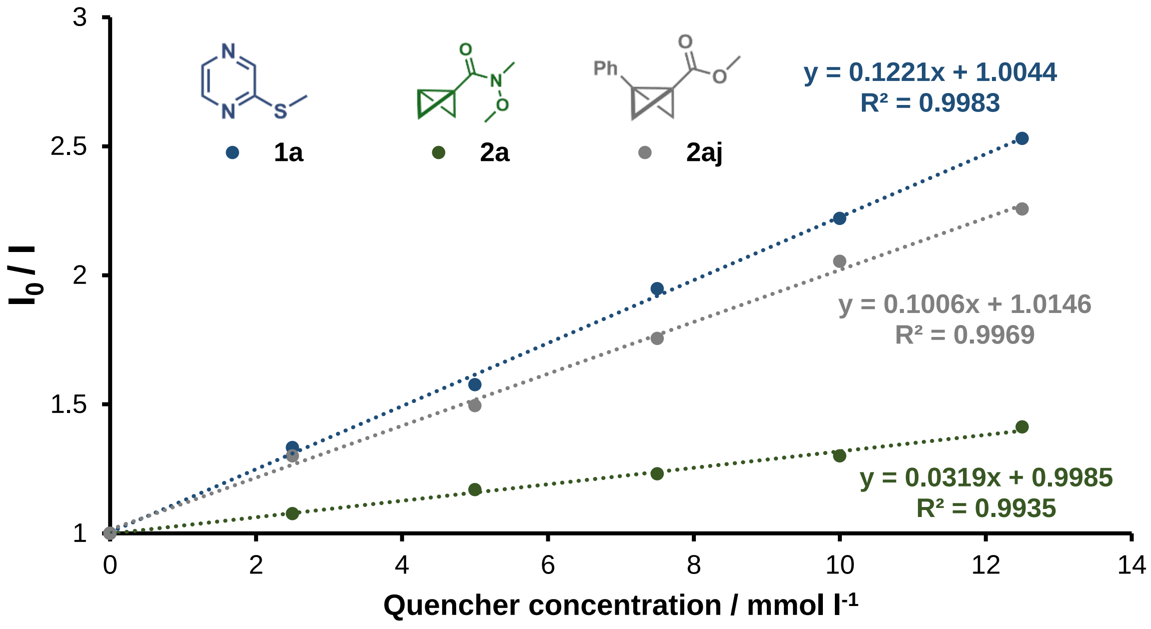

Figure S14. Stern-Volmer quenching studies of mono-substituted BCB **2a**, disubstituted BCB **2aj** and 2-(methylthio)pyrazine (**1a**).

Comment: While quenching is observed for all substrates, the quenching constant of heterocycle **2a** is approx. 4.0 times higher than BCB **1a**. Hence, the interaction of the heterocycle with photoexcited acridinium photocatalyst should be more feasible for the reaction between C2–thioether heterocycles and monosubstituted BCBs. In comparison to **2a**, disubstituted BCB **2aj** is showing only a slightly lower quenching constant, a competitive quenching behavior in the presence of acridinium photocatalyst.

3.5 Trapping experiments

3.5.1 Radical trapping experiments

According to modified **GP-1**, **1a** (25.2 mg, 0.2 mmol, 1.0 equiv.) and **2a** (14.1 mg, 0.1 mmol, 1.0 equiv.) were irradiated in the presence of **3a** photocatalyst (9.4 mg, 0.015 mmol, 15 mol%) and MeCN (2 mL, 0.05 M) overnight at λ_{max} = 425 nm in the presence of TEMPO or BHT (2.0 equiv. each). Crude ^{1}H NMR measurement using dibromomethane as internal standard was performed to quantify product formation. ESI-HRMS measurement was performed to detect any minor addition products with the radical trapping agents.

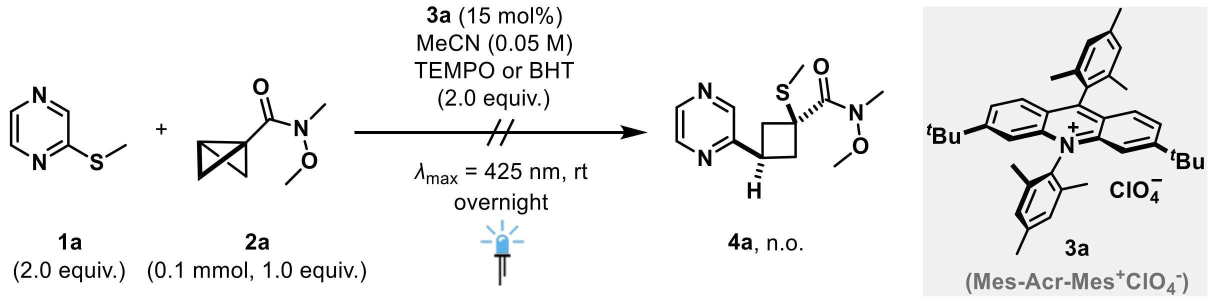

Figure S15. Radical trapping experiment with TEMPO and BHT.

Comment: Addition of TEMPO or BHT led to a complete shutdown in reactivity. However, no adducts of the radical trap agents with open-shell species could be observed by ESI-HRMS.

3.5.2 Trapping with nucleophiles

According to modified **GP-1**, **1a** (25.2 mg, 0.2 mmol, 1.0 equiv.) was irradiated in the presence of **3a** photocatalyst (18.9 mg, 0.03 mmol, 15 mol%) and MeCN (4 mL, 0.05 M) overnight at λ_{max} = 425 nm in the presence of excess nucleophiles (*p*-TolSO$_2$NH$_2$, KCN, NaN$_3$, TBAF, BnNH$_2$, imidazole, TMSCN (all 1.5 equiv. each); MeOH (10 equiv.). Then, GC-MS measurement was performed to detect any addition or substitution products.

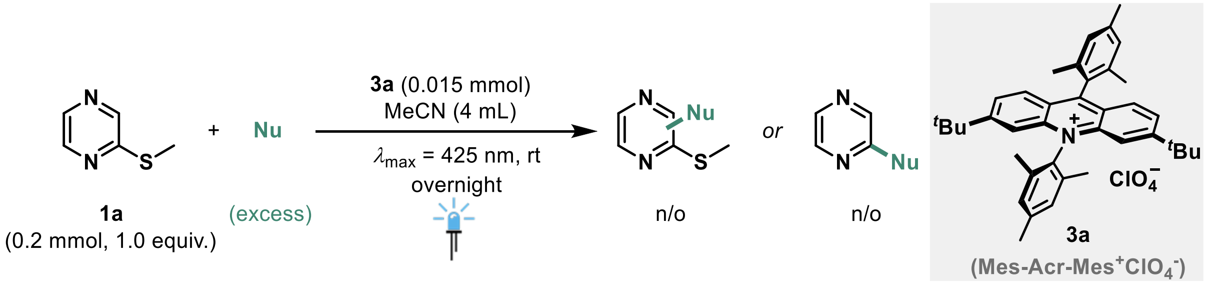

Nucleophiles: *p*-TolSO$_2$NH$_2$, KCN, NaN$_3$, TBAF, BnNH$_2$, Imidazole, TMSCN (all 1.5 equiv. each) or MeOH (10 equiv.)

Figure S16. Attempted trapping of oxidized **1a** with nucleophiles.

Comment: No nucleophile addition or substitution to / of **1a** could be observed by GC-MS.

3.6 Quantum yield measurement

3.6.1 Determination of the photon flux

The photon flux of the used blue LED (3 W, λ_{max} = 420 nm) was determined by ferrioxalate actinometry in accordance to a modified literature procedure of Yoon and coworkers.[35]

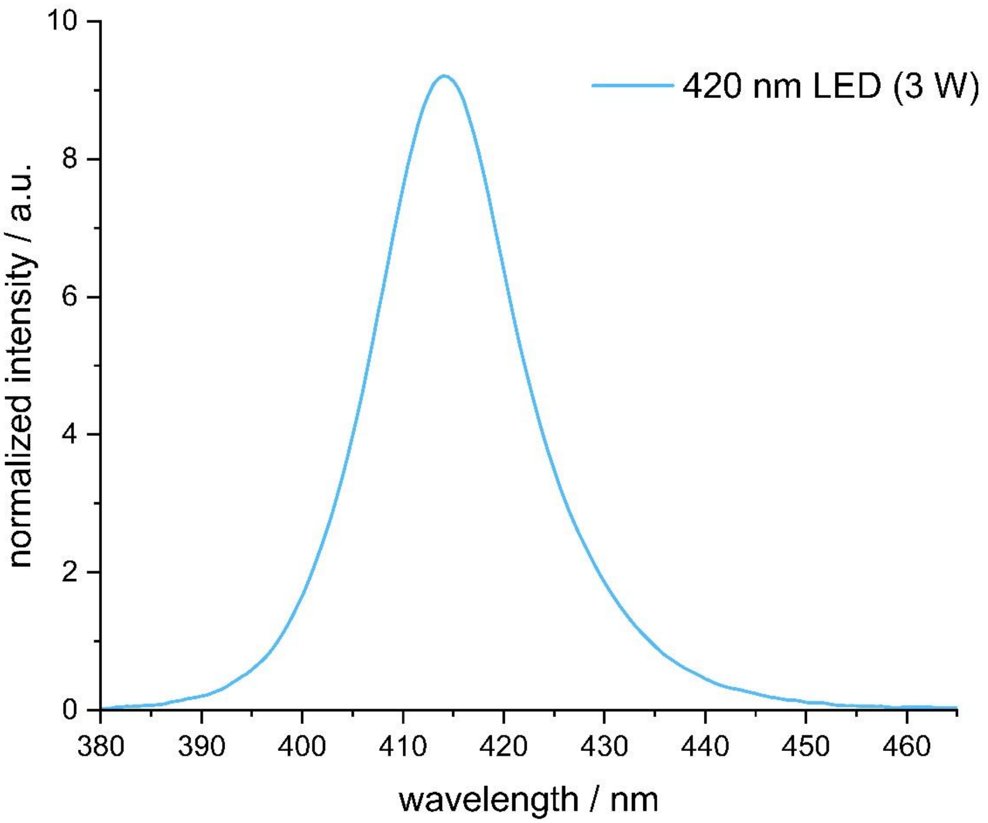

Figure S17. Emission spectra of the utilized blue LED (3 W, λ_{max} = 420 nm).

The following steps were performed in a darkened lab, including the preparation (only applies for ***solution 1***) and storage of the two solutions mentioned below, to ensure no undesired irradiation occurs. ***Solution 1*** was further wrapped with aluminum foil. The two solutions were prepared in volumetric flasks.

solution 1: Potassium ferrioxalate trihydrate (1.47 g, 3.0 mmol) was dissolved in aq. H_2SO_4 (0.05 M, 20 mL) to afford a 0.15 M ferrioxalate solution (attention: light sensitive!).

solution 2: 1,10-phenanthroline monohydrate (20 mg, 0.1 mmol), NaOAc (4.50 g) were dissolved in aq. H_2SO_4 (0.5 M, 20 mL).

By measuring the reduction of $[Fe(C_2O_4)_3]^{3-}$ to $[Fe(C_2O_4)_2]^{2-}$ by irradiation on a fixed time, the photon flux was determined. To do so, a standard Schlenk tube was charged with ***solution 1*** (1 mL) and irradiated for 60 s at λ_{max} = 420 nm (distance: 5 cm). ***Solution 2*** (175 μL) was added and the reaction mixture was sealed and stirred for 1 h at rt in darkness (covering the stirrer with a cardbox) to ensure that all Fe^{II} ions were coordinated by the phenanthroline ligand. UV/vis absorption measurements were performed at λ = 510 nm. UV/vis absorbance spectra of non-irradiated control sample were also measured after stirring Schlenk tube equipped with ***solution 1*** 60 s without LED irradiation in the prepared setup, adding ***solution 2*** and stirring for 1 h in darkness. The process was repeated for irradiated samples three times and for non-irradiated samples two times to determine the average absorbance of both. Based on the average the general amount of Fe^{II} ions ($n_{Fe(II)}$) could be calculated using the Labert-Beer law (equation 1),

$$\eta_{Fe(II)} = \frac{V \cdot \Delta A_{510\,\mathrm{nm}}}{l \cdot \varepsilon} \quad (1)$$

where V is the total volume ($1.175 \cdot 10^{-3}$ L), $\Delta A_{510\,\mathrm{nm}}$ is the difference in absorbance of irradiated and non-irradiated control samples (at λ = 510 nm), l the path length of the cuvette (1.0 cm), and ε is the molar extinction coefficient of the ferrioxalate actinometer at λ = 510 nm (11100 mol^{-1} cm^{-1}). The photon flux ϕ_q was determined by equation 2,

$$\phi_\mathrm{q} = \frac{\eta_{Fe(II)}}{\phi_\mathrm{F} \cdot t \cdot f} \quad (2)$$

where ϕ_F is the quantum yield of the ferrioxalate actinometer (1.12 at λ = 420 nm) and t is the irradiation time (60 s). The fraction of light absorbed at λ = 420 nm by the actinometer (f) is calculated by using equation 3,

$$f = 1 - 10^{-A_{420\,\mathrm{nm}}} \quad (3)$$

where $A_{420\,\mathrm{nm}}$ is the absorbance of the **solution 1** at λ = 420 nm. The absorbance value ($A_{420\,\mathrm{nm}}$) of ***solution 1*** was > 3 indicating that > 99.9% of the photons were absorbed and t f is > 0.999.

Table S3. Results of ferrioxalate actinometry.

measurement	1	2	3	4	average
$A_{510\,\mathrm{nm}}$	1.76182	1.74892	1.79725	1.80573	1.77843
$A_{510\,\mathrm{nm}}$(control)	0.521029	0.545711	0.572366	–	0.546369

$$\Delta A_{510\,\mathrm{nm}}(\mathrm{avg}) = 1.232$$

$$\boldsymbol{\phi_\mathrm{q} = 1.94 \bullet 10^{-9}\ \mathrm{mol\ s^{-1}}}$$

3.6.2 Determination of the quantum yield

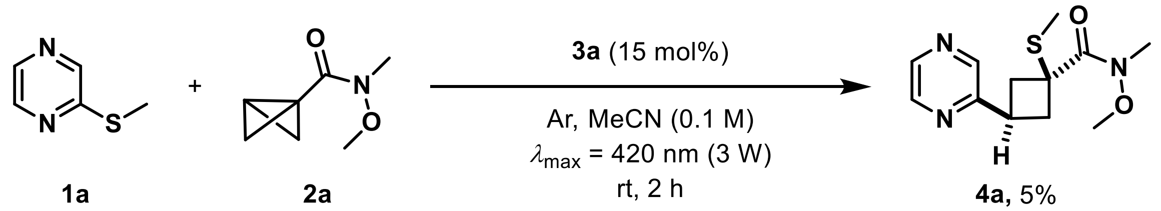

1a (2.0 equiv.) **2a** (0.1 mmol, 1.0 equiv.) **4a,** 5%

Figure S18. Quantum yield experiment.

To an oven-dried Schlenk tube equipped with a PTFE-coated stirring bar was charged with 2-(methylthio)pyrazine (25.2 mg, 0.2 mmol, 2.0 equiv.) and $[Acr\text{-}Mes_2]^+[ClO_4]^-$ (9.4 mg, 0.015 mmol, 15 mol%). The tube was evacuated and backfilled with argon three times. Under a positive argon flow *N*-methoxy-*N*-methylbicyclo[1.1.0]butane-1-carboxamide (14.1 mg, 0.1 mmol, 1.0 equiv.) were added and dissolved in MeCN (0.1 M) (*Note*: The concentration was adapted to the volume used in the ferrioxalate actinometry). The reaction was sealed and stirred under irradiation in the calibrated set-up (3 W, 420 nm) for 2 h. Mesitylene (13.9 μL, 0.1 mmol, 1.0 equiv.) was added as internal standard, the reaction mixture was homogenized, filtered over a short plug of celite eluating with EtOAc and analyzed by GC-FID. The process was repeated two times, affording an average yield of 5%.

The quantum yield of the reaction can be calculated using equation 4,

$$\phi = \frac{n_{\text{product}}}{\phi_{\text{q}} \cdot t \cdot f_{\text{R}}} \tag{4}$$

where ϕ_{q} is the photon flux and t is the time of irradiation (7200 s). The fraction of light absorbed (f_{R}) was determined by analyzing the absorbance of a non-irradiated control reaction (equation 3). f_{R} was identified as > 0.999 as $A_{420\text{ nm}}(\text{sample})$ > 3.

Quantum Yield = 0.36

3.7 Evaluation of exogenous oxidizing agents

To evaluate whether the use of exogenous oxidizing agents under thermal conditions is a viable substitution for the employed photoredox system, peroxides H_2O_2 (aq.), DBPO and DTBP were evaluated.

According to modified **GP-1**, **1a** (50.4 mg, 0.4 mmol, 2.0 equiv.), **2a** (28.2 mg, 0.2 mmol, 1.0 equiv.) and the respective peroxide (0.3 mmol, 1.5 equiv.) in MeCN (4 mL, 0.05 M) were stirred at 60 °C for 16 h.

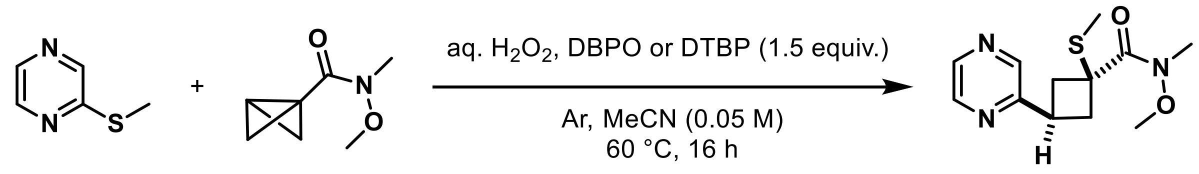

1a (2.0 equiv.) **2a** (0.2 mmol, 1.0 equiv.) **4a,** n.o.

Figure S19. Reaction with exogenous oxidizing agents under thermal conditions. DBPO = dibenzoyl peroxide. DTBP = di-*tert*-butyl peroxide.

Comment: No product formation could be detected by GC-MS and ESI-HRMS.

3.8 Crossover experiment

To evaluate whether a free radical process is a possible process, a crossover experiment using a mixture of **1a** and **1m** under standard reaction conditions was performed.

According to modified **GP-1**, **1a** (12.6 mg, 0.1 mmol, 1.0 equiv.), **1u** (24.8 mg, 0.1 mmol, 1.0 equiv.) and **2a** (14.1 mg, 0.1 mmol, 1.0 equiv.) were irradiated in the presence of **3a** photocatalyst (9.4 mg, 0.015 mmol, 15 mol%) and MeCN (2 mL, 0.05 M) overnight at λ_{max} = 425 nm. Crude ^{1}H NMR yield was determined by using dibromomethane as internal standard, further determining the diastereomeric ratio to be d.r. > 95:5. ESI-HRMS measurement was performed to detect any minor products.

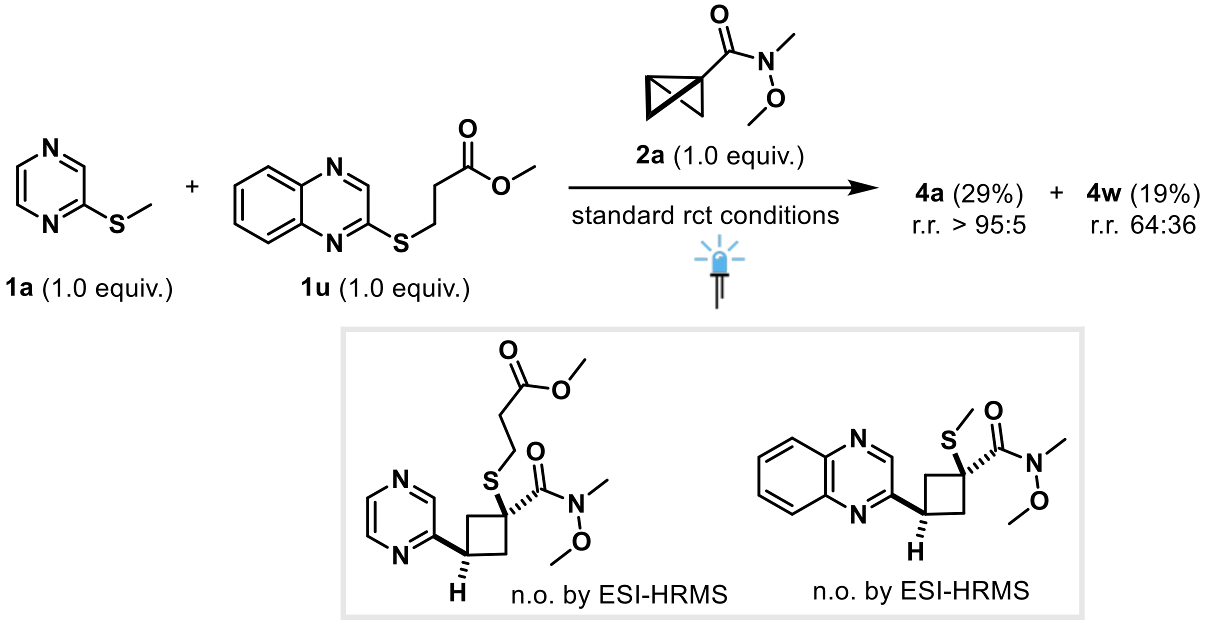

Figure S20. Crossover experiment.

Comment: No crossover product was observed, supporting the absence of free radical species / speaking against cleavage of the C–S bond prior to addition to the BCB. Notably, the regioselectivity of products **4a** and **4u** remains largely unchanged compared to the standard reaction setup.

3.9 Temperature variation study

To check whether the manipulation of reaction temperature has an impact on the regioisomeric ratio, the reaction of **1w** with **2a** under standard conditions was compared with a reaction performed under higher reaction temperature (approx. 40 °C, no fan cooling) and lower higher reaction temperature (approx. 16 °C, water-cooling).

According to modified **GP-1**, one Schlenk tube and one water-cooling Schlenk tube were equipped with a PTFE-coated stir bar, **1w** (49.6 mg, 0.2 mmol, 2.0 equiv.), **2a** (14.1 mg, 0.1 mmol, 1.0 equiv.), **3a** photocatalyst (9.4 mg, 0.015 mmol, 15 mol%) and MeCN (2 mL, 0.05 M). Both Schlenk tubes were irradiated overnight at λ_{max} = 425 nm either without fan cooling or with water cooling. Crude ^{1}H NMR yield was determined by using dibromomethane as internal standard and the regioisomeric ratios were determined. The diastereomeric ratios were determined to be d.r. > 95:5.

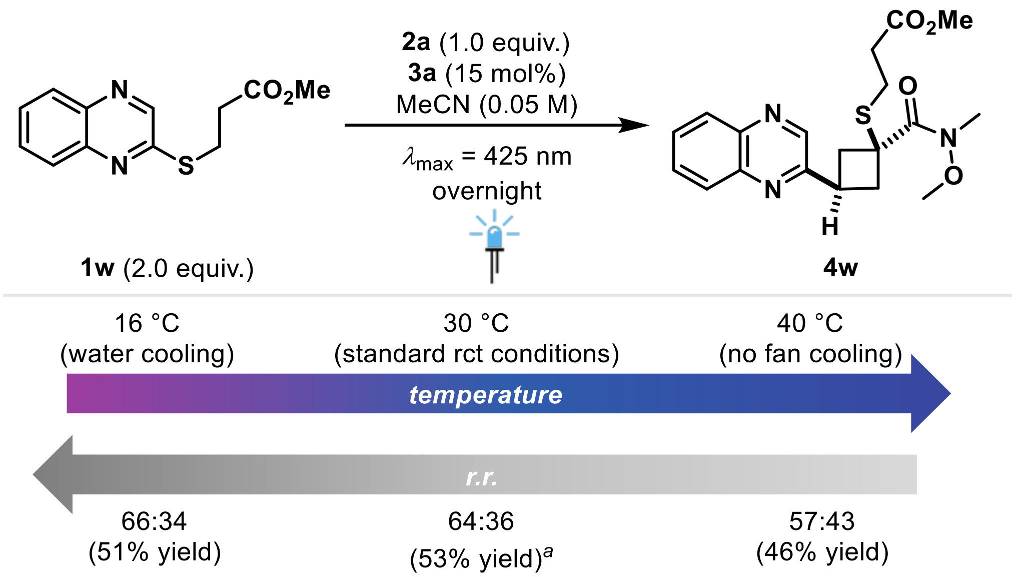

Figure S21. Temperature-dependancy of the regioisomeric ratio. Crude ^{1}H NMR yields using dibromomethane as internal standard are given. Reactions were performed at 0.1 mmol scale. [a]Reaction was performed on a 0.2 mmol scale.

4 Computational studies

All geometry optimizations of intermediates and transition states were achieved using spin-unrestricted UB3LYP[36]-D3[37–40]/def2-SVP[41,42] method, in acetonitrile as solvent using the CPCM solvent model[38–42] with "opt=noeigen" and "guess=mix" keywords as implemented in Gaussian16.[43] Frequency calculations were also conducted at the same level of theory to obtain vibrational frequencies to determine the identity of stationary points as intermediates (no imaginary frequencies) or transition states (only one imaginary frequency), as well as obtaining the thermochemistry:enthalpy (ΔH) and free energy (ΔG) at the temperature of 298 K. Also, extensive conformational search was performed for all the intermediates and transition states, whereby only the lowest energy species are shown and discussed. To compare energetics, we also carried out single-point energy calculations for the lowest energy structures using uB3LYP-D3/def2-TZVPP[41,42] CPCM(ACN), uB3LYP-D3/def2-TZVPP-gas and uM062X-D3/def2-TZVPP-CPCM(ACN). All structural figures were generated with CYLview.[44] Distances in structural figures are shown in Å and energies in kcal/mol. Barriers for single electron transfer processes were calculated at uB3LYP-D3/def2-svp-CPCM(ACN) level of theory following Four-Point Approach to the Electron-Transfer Marcus–Hush Theory.[45]

4.1 Proposed mechanism with pyrazine 1a

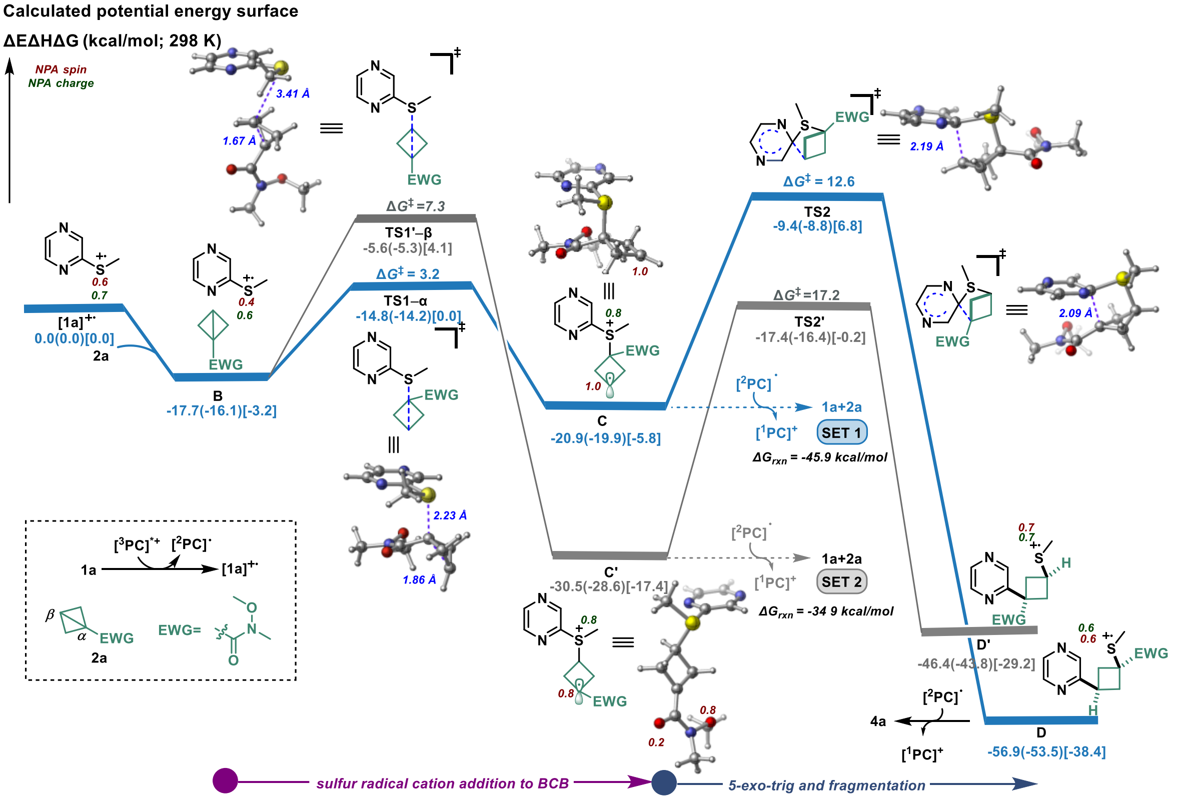

Figure S22: Proposed Mechanism supported by computational studies. Calculated energies [uB3LYP-D3/def2-svp-CPCM(ACN)] are given in kcal/mol.

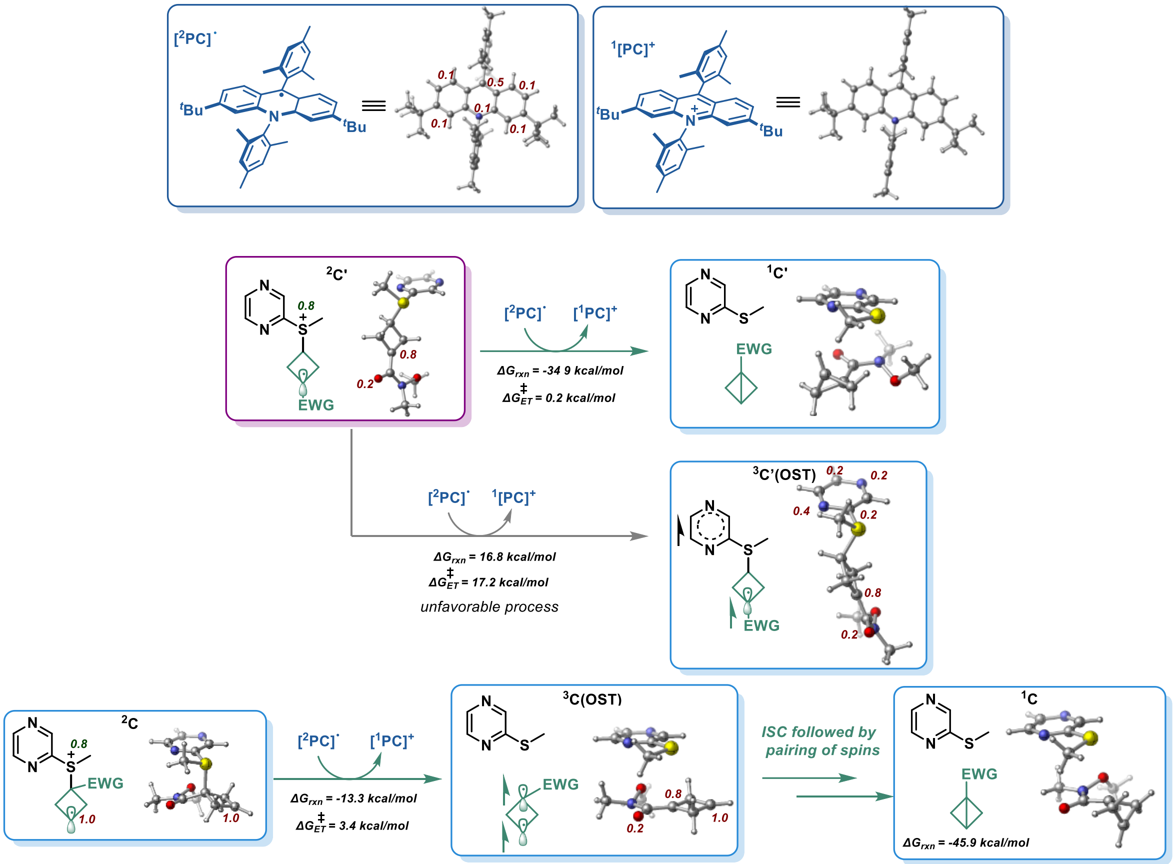

Figure S23. Plausible favorable SET (single electron reduction process) via reduced photocatalyst calculated at uB3LYP-D3/def2-svp-CPCM(ACN)]. Energies are given in kcal/mol.

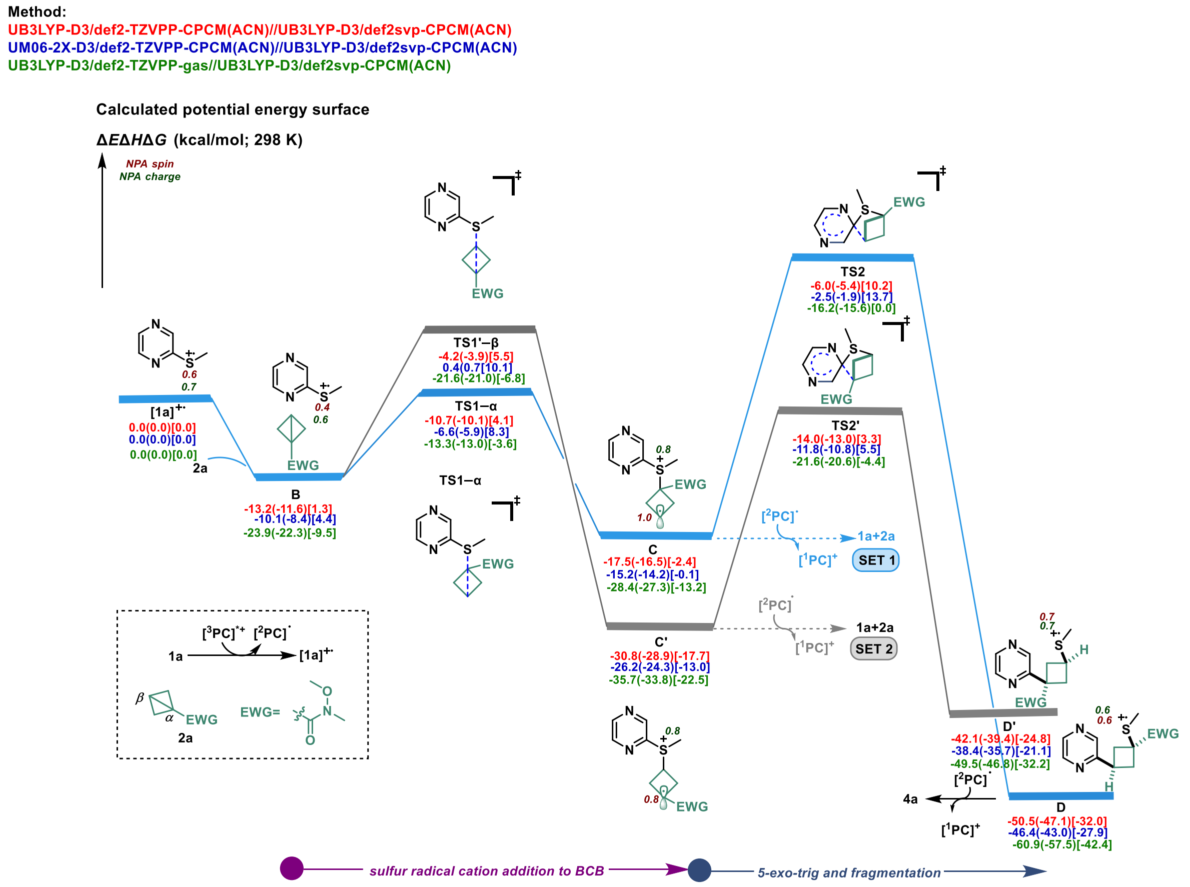

Figure S24. Method comparison on the energetics for the model system. Optimizations were carried out using uB3LYP-D3/def2-svp-CPCM(ACN) level of theory followed by single point energy calculations with chosen functionals (uB3LYP-D3, uM06-2X-D3, uB3LYP-D3-gas) with the def2TZVPP basis set.

4.2 Proposed mechanism with benzothiazole 1x

4.2.1 Activation strain/ distortion analysis for TS3–β and TS3'–α

DFT calculation revealed that, **[1x]**$^{+\cdot}$ regioselectively undergoes addition to the β–carbon of the BCB (via **TS3-β**) to generate a more stable radical cationic intermediate **F** irreversibly (downhill by 9.4 kcal/mol from radical cation intermediate species **[1x]**$^{+\cdot}$) which in turn involves in 5-*exo*-trig type addition / fragmentation via **TS4** ($\Delta G^{‡}$=12.7 kcal/mol) to deliver major regioisomer product **G** (downhill by 32.1 kcal/mol) followed by facile C-S bond cleavage. In addition, the alternative addition to the α–carbon via a relatively higher energetic transition state **TS3–α** ($\Delta G^{‡}$=8.7 kcal/mol) could lead to radical cation intermediate **F'** reversibly (uphill by 2.8 kcal/mol in energy with respect to **[1x]**$^{+\cdot}$). However, these data indicate that in this case the first step (in addition to the BCB) is crucial for determining the regioselectivity.

To gain further insight into the origins of regioselectivity, **TS3–β** and **TS3'–α** were analyzed with regard to their distortion and interaction (Table S4).[46] Single-point energy calculations at the uB3LYP-D3/def2SVP-CPCM(ACN) level of theory were conducted for: (i) the whole structure, (ii) just benzothiazole core **fragment1** and (iii) the BCB fragment (i.e. the remaining fragment after removing C2–thioether containing benzothiazole; **fragment2**). The contributions of distortion and interaction were then calculated as follows:

$$\text{Potential energy surface } (\Delta E) = [E\text{ (TS)} - E]$$
$$\Delta E\text{ (distortion)} = [E\text{ (fragment 1)} + E\text{ (fragment 2)}] - [E\text{ ([1x]}^{+\cdot}) + E\text{(2a)}]$$
$$\Delta E\text{ (interaction)} = \Delta E - \Delta E\text{ (distortion)}$$

Table S4. Single point energies are used for the distortion/interaction analysis and resulting ΔE values. Energies are given in kcal/mol.

Structure	*E(BCB)*	*E(heterocycle)*	*E(frag1)*	*E(frag2)*	ΔE_{dis}	ΔE_{int}
TS3–β	-478.106638	-1159.524005	-1159.519632	-478.087997	14.4	-8.9
TS3'–α	-478.106638	-1159.524005	-1159.517741	-478.065149	30.0	-22.7

Overall, this analysis revealed **TS3–β** has a relatively low activation energy of 1.7 kcal/mol compared to **TS3'–α**, presumably due to steric interaction between the EWG group and bulkier benzothiazole suggested by higher distortion energy **(**$\Delta\Delta E_{dis(rel)}$ = 15.6 kcal/mol) between the two fragments for **TS3–β.**

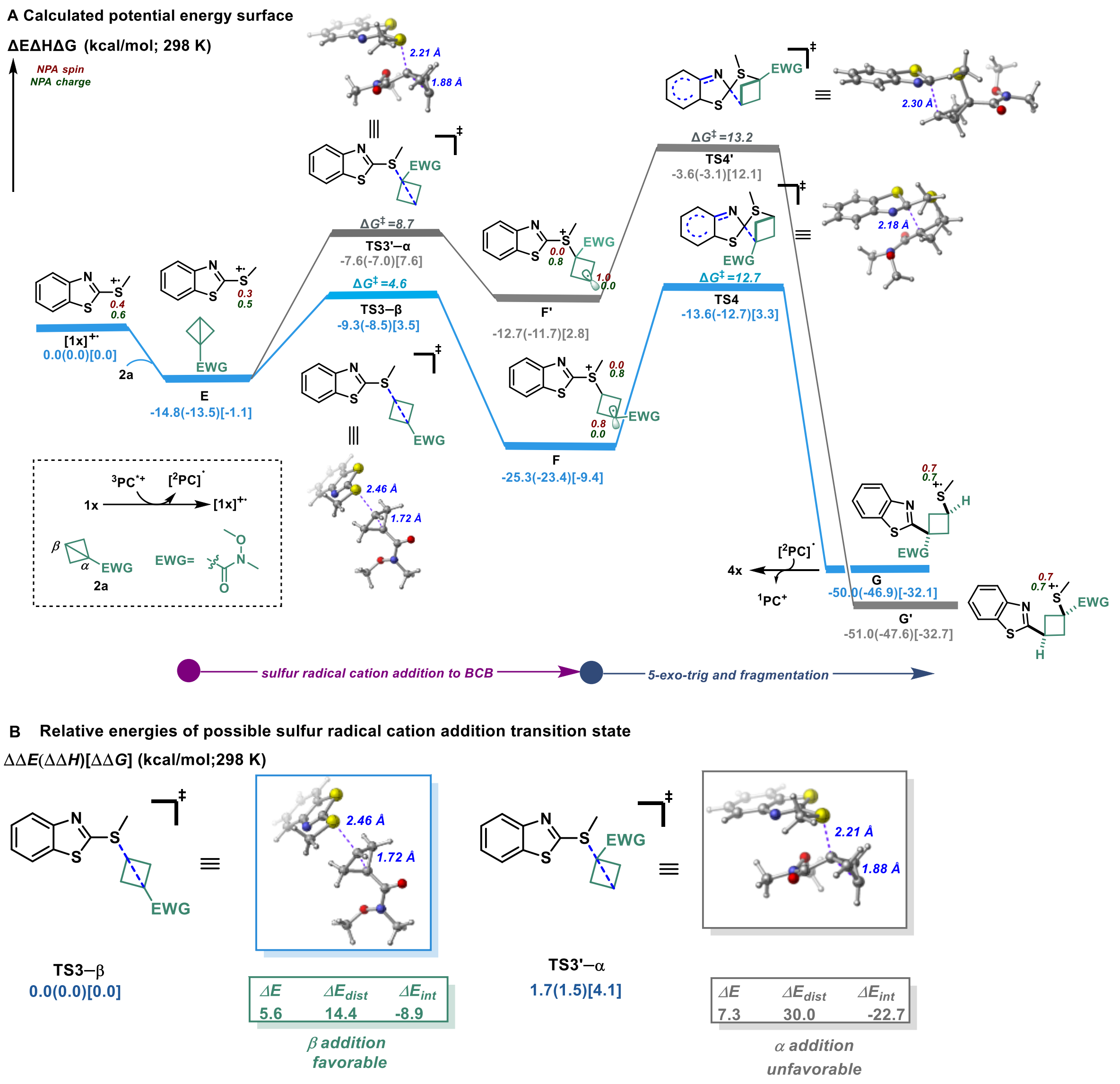

Figure S25: Proposed mechanism with benzothiazole substrate supported by computational studies. Calculated energies [uB3LYP-D3/def2-svp-CPCM(ACN)] are given in kcal/mol.

4.3 Proposed mechanism with disubstituted BCB 2ag

DFT calculations revealed that **[1a]**$^{+\cdot}$ regioselectively undergoes a reversible, rapid addition to the α–carbon at **2ag** (via **TS5–α**) through a small energy barrier of 0.5 kcal/mol (with respect to **J**) generating a higher stabilized radical cationic intermediate **I** (downhill by 15.7 kcal/mol from radical cationic species **[1a]**$^{+\cdot}$), which in turn undergoes a 5-*exo*-trig type addition/fragmentation via **TS6** (with an energy barrier of 15.5 kcal/mol) to deliver the major regioisomer product **J** followed by facile C–S bond cleavage.

Alternatively, addition to the β–carbon via a relatively higher energetic transition state **TS5'–β** ($\Delta G^{\ddagger}$=3.7 kcal/mol) could lead to the reversible formation of relatively less stable radical cationic intermediate **I'** (downhill by 13.3 kcal/mol), which could involve in the cyclization/fragmentation process ($\Delta G^{\ddagger}$=14.7 kcal/mol) to offer the minor regioisomeric product **J'**. However, with similar cyclization barriers, we suggest that the first step, which is the formation of radical cationic intermediate through the addition of sulfur radical cation to BCB, is the key regiodetermining step. Overall, this data explains the formation of the major regioisomeric product, plausibly driven by the formation of the benzylic radical intermediate in the first step, which corroborates with experimental findings.

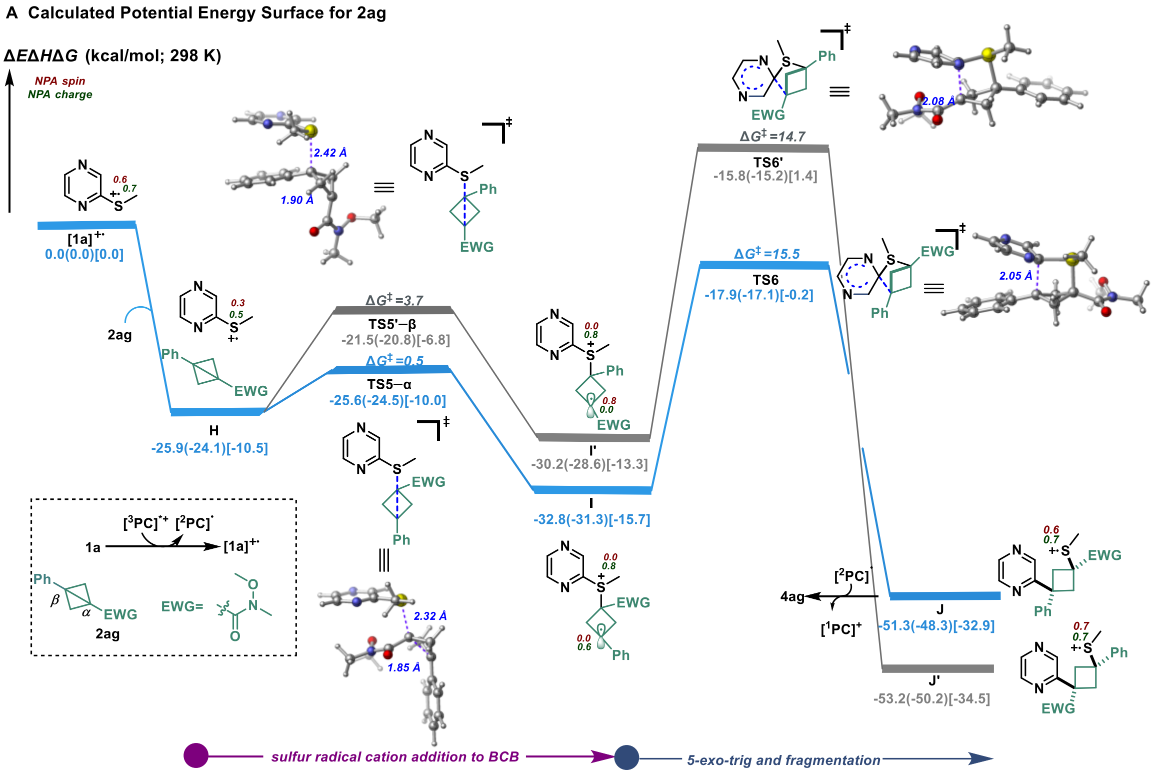

Figure S26: Proposed mechanism with disubstituted BCB supported by computational studies. Calculated energies [uB3LYP-D3/def2-svp-CPCM(ACN)] are given in kcal/mol.

To gain further insight on the origins of regioselectivity, **TS5–α** and **TS5'–β** were analyzed with regard to their distortion and interaction (Table S5).[46]

Table S5. Single point energies are used for the distortion/interaction analysis and resulting Δ*E* values. Energies are given in kcal/mol.

Structure	*E(BCB)*	*E(heterocycle)*	*E(frag1)*	*E(frag2)*	*ΔE*	ΔE_{dis}	ΔE_{int}
**TS5–α**	**-709.015402**	**-701.275314**	**-701.272079**	**-708.987685**	**0.4**	**19.4**	**-19.0**
**TS5'–β**	**-709.015402**	**-701.275314**	**-701.272844**	**-708.981564**	**4.4**	**22.8**	**-18.4**

Overall, this analysis revealed that the lower barrier associated with α–addition transition state **TS5–α** is due to lower distortion energy required for **TS5–α** ($\Delta\Delta E_{dis}$= 3.4 kcal/mol) compared to **TS5'–β.** A similar trend was observed with ester as an electron withdrawing group (EWG) on the disubstituted BCB (Figure S27).

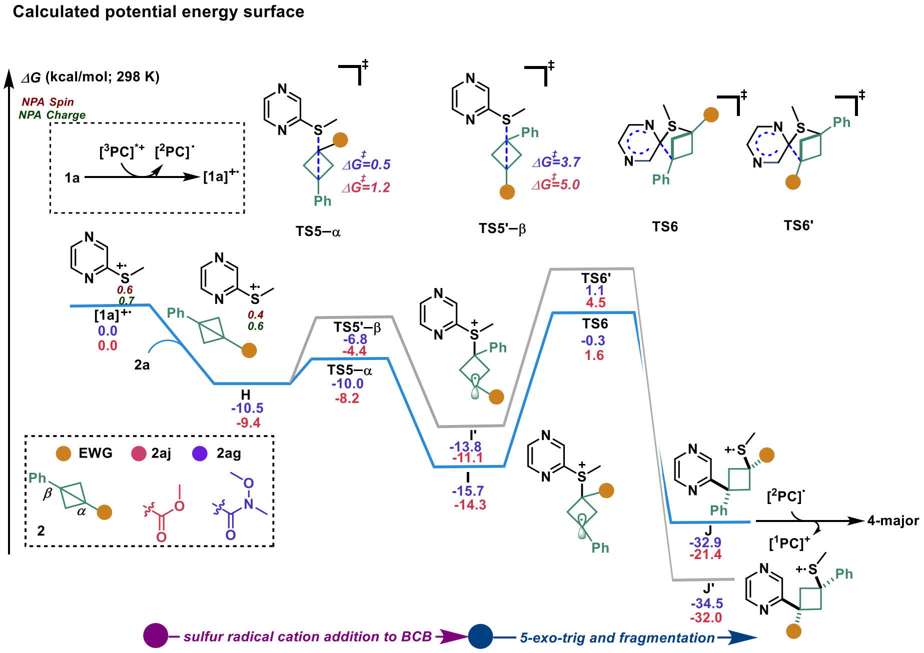

Figure S27: Proposed mechanism with ester as an electron withdrawing group (EWG) on the disubstituted BCB. supported by computational studies. Calculated free Gibbs energies [uB3LYP-D3/def2-svp-CPCM(ACN)] are given in kcal/mol.

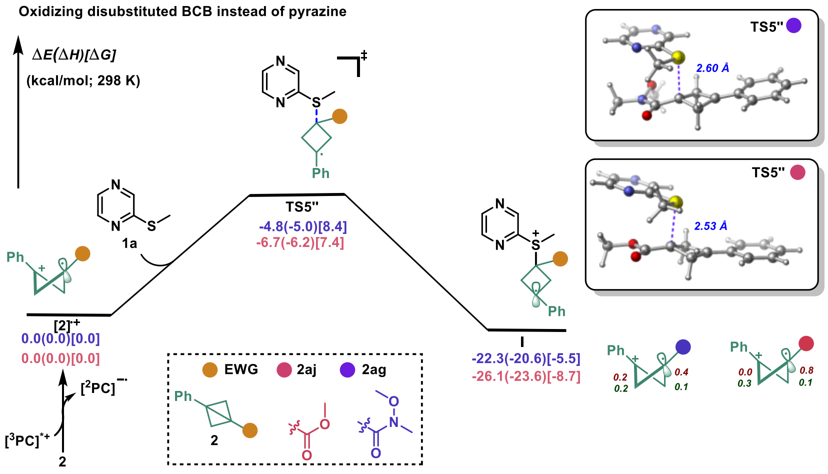

Figure S28: Alternative pathway of oxidized disubstituted BCB addition **[2ag]** $^{+\cdot}$ and **[2aj]** $^{+\cdot}$ onto neutral **1a** was ruled out based on a higher energy barrier of 8.4 kcal/mol and 7.4 kcal/mol vs 0.5 kcal/mol and 1.2 kcal/mol with BCB addition onto oxidized **[1a]** $^{+\cdot}$ respectively. This hints towards a lower reactivity of the former radical cationic species. Calculated energies [uB3LYP-D3/def2-svp-CPCM(ACN)] are given in kcal/mol.

4.4 Coordinates

Table S6. Cartesian coordinates (xyz format) and energies of all the structures involved in each reaction mechanism studied calculated at the CPCM(ACN) uB3LYP-d3/def2-svp level of theory.

2a

E(scf) = -478.106638317 a.u.

ν_{min} = 38.4304 cm^{-1}

C	-2.329755	-0.282079	0.525801	C	2.328733	-1.286431	0.760031
C	-1.989939	-0.960424	-0.753665	H	-2.736549	0.731292	0.457098
C	-0.922966	-1.889642	-0.269262	H	-2.696045	-0.879689	1.373850
C	-0.882127	-0.436737	0.124619	H	-2.204989	-0.640288	-1.773087
C	-0.005498	0.717412	-0.190410	H	-0.180755	-2.222074	-0.998171
N	1.334014	0.484646	-0.376147	H	-1.172134	-2.630896	0.505404
O	-0.456923	1.861221	-0.239373	H	1.810808	2.470794	-0.719133
C	2.265024	1.483212	-0.857386	H	3.203631	1.429804	-0.284985
O	1.801607	-0.818202	-0.482756	H	2.487685	1.326998	-1.926727

H	2.729589	-2.288762	0.554716	H	1.536474	-1.354287	1.524174
H	3.140712	-0.633969	1.123210				

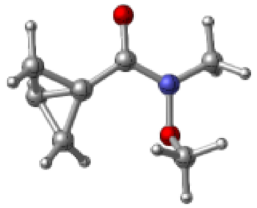

Zero-point correction= 0.173010 (Hartree/Particle)

Thermal correction to Energy= 0.183721

Thermal correction to Enthalpy= 0.184665

Thermal correction to Gibbs Free Energy= 0.136262

Sum of electronic and zero-point Energies= -477.933628

Sum of electronic and thermal Energies= -477.922918

Sum of electronic and thermal Enthalpies= -477.921973

Sum of electronic and thermal Free Energies= -477.970376

UB3LYP-D3/def2TZVPP

E(scf)= -478.647352929

UM062X-D3/def2TZVPP

E(scf)= -478.432320363

UB3LYP-D3/def2TZVPP (gas)

E(scf)= -478.639158467

[1a]$^{+\cdot}$

E(scf) = -701.275314222 a.u.

ν_{min} = 80.2136 cm^{-1}

C	-0.982308	0.208802	-0.980841	C	-0.892842	2.467957	-0.960871
C	0.434467	0.127239	-0.834577	N	-1.633135	1.380475	-1.043130
N	1.170055	1.230936	-0.755136	H	0.942238	-0.841851	-0.782186
C	0.523256	2.394567	-0.816581	H	1.115074	3.313091	-0.754610

H	-1.393477	3.439805	-1.008543	H	-3.666780	-0.101537	-2.123750
S	-1.862595	-1.276935	-1.070512	H	-4.166145	-1.656881	-1.328472
C	-3.573558	-0.738855	-1.233064	H	-3.855729	-0.169898	-0.335407

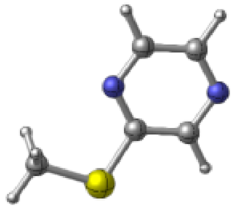

Zero-point correction= 0.104238 (Hartree/Particle)

Thermal correction to Energy= 0.111902

Thermal correction to Enthalpy= 0.112846

Thermal correction to Gibbs Free Energy= 0.070793

Sum of electronic and zero-point Energies= -701.171076

Sum of electronic and thermal Energies= -701.163412

Sum of electronic and thermal Enthalpies= -701.162468

Sum of electronic and thermal Free Energies= -701.204522

UB3LYP-D3/def2TZVPP

E(scf)= -701.754065276

UM062X-D3/def2TZVPP

E(scf)= -701.572490991

UB3LYP-D3/def2TZVPP (gas)

E(scf)= -701.675216611

B

E(scf) = -1179.41017397 a.u.

ν_{min} = 23.7068 cm^{-1}

C	-2.246424	0.964085	0.434163	C	-1.694478	-1.193576	-0.037906
C	-2.268631	0.005235	-0.676197	C	-0.983438	0.105468	0.319030

C	0.279605	0.727138	-0.190097	S	-0.297930	-0.110447	2.893712
N	1.316496	-0.080557	-0.551634	C	0.351831	1.566912	3.091866
O	0.370690	1.953639	-0.230721	H	0.727287	1.944878	2.130779
C	2.631577	0.425456	-0.901974	H	-0.497231	2.171463	3.435770
O	1.237289	-1.433131	-0.272575	H	1.155431	1.549545	3.839276
C	1.357828	-2.251616	-1.442503	C	1.160586	-1.074669	2.642024
H	-2.149975	2.026774	0.197899	C	1.014047	-2.476100	2.556231
H	-2.854908	0.735046	1.320372	N	2.333779	-0.467825	2.500860
H	-2.247742	-1.633673	0.805042	N	2.061868	-3.250414	2.299173
H	-1.192536	-1.926361	-0.669705	H	0.037520	-2.952578	2.693753
H	2.618988	1.514163	-0.782236	C	3.381242	-1.251301	2.252105
H	3.379067	-0.017986	-0.228450	C	3.241757	-2.644216	2.134981
H	2.887458	0.179637	-1.943759	H	4.359031	-0.773905	2.137321
H	1.161017	-3.273955	-1.094648	H	4.110339	-3.270507	1.909499
H	0.621782	-1.960060	-2.209383	H	-2.167915	0.261321	-1.733213
H	2.374310	-2.200562	-1.863294				

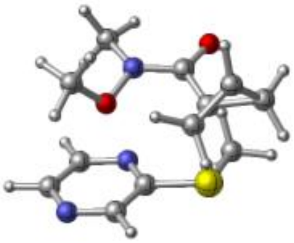

Zero-point correction= 0.279571 (Hartree/Particle)

Thermal correction to Energy= 0.299197

Thermal correction to Enthalpy= 0.300141

Thermal correction to Gibbs Free Energy= 0.230114

Sum of electronic and zero-point Energies= -1179.130603

Sum of electronic and thermal Energies= -1179.110977

Sum of electronic and thermal Enthalpies= -1179.110033

Sum of electronic and thermal Free Energies= -1179.180060

UB3LYP-D3/def2TZVPP

E(scf)= -1180.42247249

UM062X-D3/def2TZVPP

E(scf)= -1180.02086442

UB3LYP-D3//def2TZVPP (gas)

E(scf)= -1180.35252005

TS1–α

E(scf) = -1179.40558208 a.u.

ν_{min} = -320.3632 cm^{-1}

C	-2.127320	1.068108	0.534122	H	0.482807	-2.027930	-2.202317
C	-2.385060	0.195308	-0.646257	H	2.227255	-2.348359	-1.879920
C	-1.742764	-1.035176	-0.096639	S	-0.343267	-0.270286	2.628991
C	-0.933573	0.109946	0.509160	C	0.144163	1.419718	3.074681
C	0.334824	0.689541	-0.111921	H	0.829810	1.826934	2.322724
N	1.286640	-0.169483	-0.566241	H	-0.786960	1.998832	3.117658
O	0.483334	1.906217	-0.143893	H	0.618073	1.381320	4.063997
C	2.604913	0.267898	-0.993607	C	1.231142	-1.112052	2.539119
O	1.152591	-1.516320	-0.278569	C	1.230447	-2.517833	2.562723
C	1.216003	-2.347719	-1.444390	N	2.334726	-0.393698	2.410297
H	-1.923764	2.132688	0.378705	N	2.368401	-3.190353	2.422985
H	-2.799150	0.886539	1.390427	H	0.302322	-3.084446	2.690795
H	-2.341158	-1.563160	0.665619	C	3.476460	-1.073378	2.284504
H	-1.263829	-1.737179	-0.781986	C	3.488040	-2.475094	2.273773
H	2.663684	1.352902	-0.855210	H	4.402399	-0.499708	2.183544
H	3.364991	-0.231644	-0.375401	H	4.428530	-3.020309	2.147689
H	2.777227	0.027813	-2.053239	H	-2.357162	0.513866	-1.691597
H	0.974843	-3.356185	-1.084767				

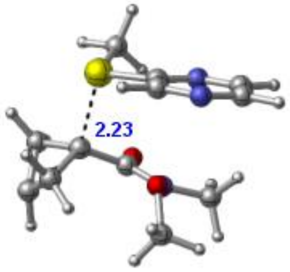

Zero-point correction= 0.278763 (Hartree/Particle)

Thermal correction to Energy= 0.297628

Thermal correction to Enthalpy= 0.298572

Thermal correction to Gibbs Free Energy= 0.230695

Sum of electronic and zero-point Energies= -1179.126819

Sum of electronic and thermal Energies= -1179.107954

Sum of electronic and thermal Enthalpies= -1179.107010

Sum of electronic and thermal Free Energies= -1179.174887

UB3LYP-D3/def2TZVPP

E(scf)= -1180.41853825

UM062X-D3/def2TZVPP

E(scf)= -1180.01526239

UB3LYP-D3/def2TZVPP (gas)

E(scf)= -1180.34886643

TS1'–β

E(scf) = -1179.39086874 a.u.

ν_{min} = -112.1449 cm^{-1}

C	-2.442492	1.114174	-1.456532	H	-1.910298	1.642700	0.598612
C	-1.901771	1.953228	-0.452401	H	-0.935365	4.473780	-2.293724
N	-1.368248	3.130895	-0.750462	H	-1.905794	2.982742	-4.079482
C	-1.371182	3.502703	-2.039621	S	-3.096735	-0.464997	-0.983703
C	-1.909072	2.677084	-3.028138	C	-3.799264	-1.051060	-2.557250
N	-2.437602	1.482163	-2.736511	H	-4.558086	-0.346693	-2.922893

H	-3.018145	-1.176710	-3.318157
H	-4.261299	-2.021452	-2.330318
C	-0.022047	-2.122523	-2.273798
C	-0.115144	-0.691547	-2.628912
C	0.812453	-0.106836	-1.640511
C	1.308657	-1.426216	-2.170666
C	2.423554	-1.564911	-3.160439
N	3.526437	-2.185073	-2.735886
O	2.207536	-1.088282	-4.271816
C	4.767987	-2.294345	-3.478114
O	3.545539	-2.500861	-1.391334
C	3.386090	-3.913131	-1.156702
H	-0.435212	-2.392477	-1.286279
H	-0.177190	-2.878901	-3.049397
H	-0.270596	-0.304477	-3.638331
H	0.500395	-0.174607	-0.583528
H	1.348413	0.813840	-1.891265
H	4.587927	-1.897176	-4.485362
H	5.548495	-1.704605	-2.975169
H	5.080682	-3.345402	-3.552163
H	3.381326	-4.017574	-0.064562
H	2.434747	-4.275486	-1.576418
H	4.229059	-4.481291	-1.579192

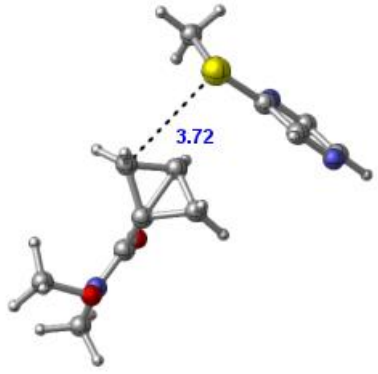

Zero-point correction= 0.277068 (Hartree/Particle)

Thermal correction to Energy= 0.297016

Thermal correction to Enthalpy= 0.297960

Thermal correction to Gibbs Free Energy= 0.222512

Sum of electronic and zero-point Energies= -1179.113800

Sum of electronic and thermal Energies= -1179.093853

Sum of electronic and thermal Enthalpies= -1179.092909

Sum of electronic and thermal Free Energies= -1179.168357

UB3LYP-D3/def2TZVPP

E(scf)= -1180.40813520

UM062X-D3/def2TZVPP

E(scf) = -1180.00414054

UB3LYP-D3/def2TZVPP (gas)

E(scf) = -1180.33549152

C

E(scf) = -1179.41533440 a.u.

ν_{min} = 33.2508 cm^{-1}

C	-1.667557	-0.164434	-0.705424	C	-0.023741	-1.128845	-3.294591
C	-1.463851	-0.504855	0.635697	N	0.973626	-0.537797	-2.581259
N	-0.856775	0.360024	1.448460	O	-0.282294	-0.822526	-4.451426
C	-0.486696	1.531884	0.930887	C	1.677992	0.645837	-3.042691
C	-0.743743	1.868849	-0.408794	O	1.024491	-0.783716	-1.217947
N	-1.334827	0.999785	-1.227164	C	2.307869	-1.243442	-0.776168
H	-1.789292	-1.466695	1.044593	H	-0.494384	-3.191967	-0.570814
H	0.024380	2.240893	1.589545	H	0.825586	-3.333314	-1.742847
H	-0.462440	2.845173	-0.813058	H	-1.071483	-5.406242	-2.379097
S	-2.368079	-1.420334	-1.809675	H	-2.452325	-3.320167	-3.843254
C	-3.080407	-0.411052	-3.136212	H	-0.793987	-3.393382	-4.462476
H	-3.314306	-1.096981	-3.958829	H	1.379657	0.829145	-4.080661
H	-3.997974	0.025469	-2.721354	H	1.399118	1.506148	-2.414844
H	-2.362270	0.358228	-3.439053	H	2.766150	0.495929	-2.999534
C	-0.273496	-3.289729	-1.647859	H	2.153173	-1.554380	0.264946
C	-0.998113	-4.321551	-2.458146	H	2.655193	-2.097004	-1.379502
C	-1.392450	-3.353907	-3.533393	H	3.053821	-0.433990	-0.808346
C	-0.865445	-2.206678	-2.605134				

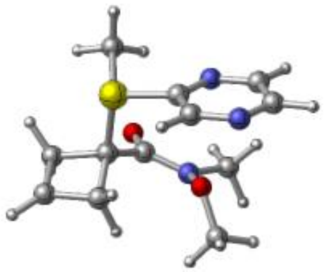

Zero-point correction= 0.278995 (Hartree/Particle)

Thermal correction to Energy= 0.298267

Thermal correction to Enthalpy= 0.299211

Thermal correction to Gibbs Free Energy= 0.231140

Sum of electronic and zero-point Energies= -1179.136339

Sum of electronic and thermal Energies= -1179.117067

Sum of electronic and thermal Enthalpies= -1179.116123

Sum of electronic and thermal Free Energies= -1179.184195

UB3LYP-D3/def2TZVPP

E(scf)= -1180.42937616

UM062X-D3/def2TZVPP

E(scf)= -1180.02908660

UB3LYP-D3/def2TZVPP (gas)

E(scf)= -1180.35957235

C’

E(scf) = -1179.43063690 a.u.

ν_{min} = 7.0885 cm^{-1}

C	-2.520960	-2.784063	-0.728265	H	-3.310065	-1.163702	0.501774
C	-3.476165	-2.157938	0.075865	H	-5.744945	-4.509822	0.021012
N	-4.627446	-2.783194	0.338068	H	-3.983578	-5.597515	-1.428061
C	-4.803890	-3.992800	-0.191468	S	-0.966663	-1.915949	-1.096153
C	-3.826796	-4.604065	-0.998941	C	0.260143	-3.209978	-0.751628
N	-2.679846	-3.982045	-1.261972	H	-0.074852	-4.144296	-1.219586

H	1.212763	-2.858482	-1.168907	H	0.887385	-0.694851	-2.947201
H	0.327921	-3.301924	0.339872	H	0.793974	-1.888637	-4.266066
C	0.191731	-1.229227	-3.619739	H	-1.194356	-2.971669	-3.224018
C	-1.015319	-1.930188	-2.926683	H	-2.436896	-0.169342	-2.838503
C	-1.986369	-0.885248	-3.548042	H	-2.806148	-1.324432	-4.137562
C	-0.805641	-0.339992	-4.300761	H	-0.380896	2.606753	-7.014412
C	-0.512911	0.682227	-5.289777	H	-2.136915	2.735683	-7.336695
N	-1.566907	1.395557	-5.826437	H	-1.437622	3.502156	-5.870950
O	0.651109	0.886796	-5.651609	H	-4.686177	0.622762	-5.385107
C	-1.373845	2.635093	-6.551342	H	-3.979172	1.265319	-6.904625
O	-2.791432	1.298635	-5.172013	H	-3.463607	-0.345007	-6.287548
C	-3.775394	0.672177	-5.997438				

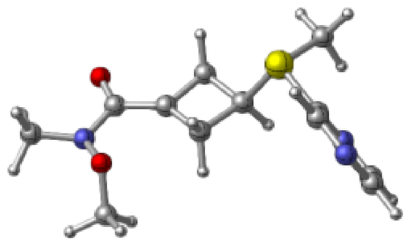

Zero-point correction= 0.280263 (Hartree/Particle)

Thermal correction to Energy= 0.299606

Thermal correction to Enthalpy= 0.300551

Thermal correction to Gibbs Free Energy= 0.227994

Sum of electronic and zero-point Energies= -1179.150373

Sum of electronic and thermal Energies= -1179.131030

Sum of electronic and thermal Enthalpies= -1179.130086

Sum of electronic and thermal Free Energies= -1179.202643

UB3LYP-D3/def2TZVPP

E(scf)= -1180.45054400

UM062X-D3/def2TZVPP

E(scf)= -1180.04653812

UB3LYP-D3/def2TZVPP (gas)

E(scf)= -1180.37123043

D

E(scf) = -1179.47269150 a.u.

ν_{min} = 42.4133 cm^{-1}

S	2.658208	-0.209753	0.842203	H	1.931082	-2.757803	1.889606
C	2.791782	1.582154	1.048236	H	2.674795	-2.774175	3.517726
H	1.834315	1.991309	1.398654	H	6.962252	-0.626567	2.938462
H	3.588977	1.793152	1.776735	H	7.182445	-2.367440	2.587987
H	3.041259	1.996915	0.063100	H	7.011687	-1.197641	1.232674
C	1.975231	-0.162298	3.611791	H	4.250019	-4.352908	0.927798
C	0.987497	-1.352728	3.436036	H	5.898501	-4.125518	1.597909
C	2.155553	-2.151782	2.777488	H	4.465081	-4.023575	2.685223
C	2.915424	-0.813724	2.569241	C	-0.126909	-1.044305	2.478943
C	4.417308	-0.663330	2.856970	C	-1.479936	-1.347689	2.722031
N	5.245670	-1.583832	2.294025	N	0.180307	-0.442415	1.326224
O	4.814684	0.265171	3.547892	N	-2.440194	-1.057151	1.847250
C	6.690470	-1.441334	2.257730	H	-1.764176	-1.838895	3.658266
O	4.691261	-2.425350	1.341095	C	-0.752657	-0.144570	0.430125
C	4.845374	-3.811081	1.674074	C	-2.090367	-0.458671	0.708249
H	1.595965	0.850162	3.424438	H	-0.447039	0.340725	-0.500540
H	2.465385	-0.178374	4.593664	H	-2.874841	-0.215387	-0.014742
H	0.580752	-1.781045	4.360193				

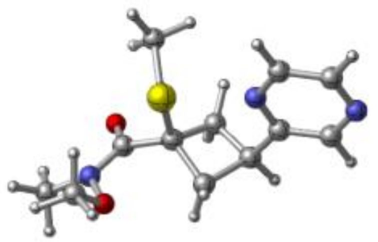

Zero-point correction= 0.283706 (Hartree/Particle)

Thermal correction to Energy= 0.301993

Thermal correction to Enthalpy= 0.302937

Thermal correction to Gibbs Free Energy= 0.236521

Sum of electronic and zero-point Energies= -1179.188986

Sum of electronic and thermal Energies= -1179.170699

Sum of electronic and thermal Enthalpies= -1179.169755

Sum of electronic and thermal Free Energies= -1179.236171

UB3LYP-D3/def2TZVPP

E(scf)= -1180.48185703

UM062X-D3/def2TZVPP

E(scf) = -1180.07877213

UB3LYP-D3/def2TZVPP (gas)

E(scf)= -1180.41139463

D'

E(scf) = -1179.45597519 a.u.

ν_{min} = 35.8377cm^{-1}

C	-0.724304	0.429484	-2.485042	H	-3.185086	-2.953007	-1.243721
C	0.438004	0.470652	-1.677276	H	-2.428244	-2.588868	0.361420
N	0.483689	1.164001	-0.534596	C	0.082666	-1.611354	-3.844109
C	-0.611659	1.832295	-0.191149	C	-1.080012	-2.395143	-3.182428
C	-1.772555	1.813420	-0.997825	C	-2.054963	-1.312404	-3.696298
N	-1.824204	1.114053	-2.122461	C	-0.833401	-0.353348	-3.777284
H	1.347841	-0.051161	-1.975247	C	-0.881997	0.605222	-4.986978
H	-0.593650	2.408659	0.739506	N	0.284557	1.242393	-5.291133
H	-2.661342	2.382163	-0.705939	O	-1.910383	0.771746	-5.621522
S	-0.838526	-2.445106	-1.380339	C	0.363618	2.385522	-6.183055
C	-2.497720	-2.293356	-0.693558	O	1.337449	1.101263	-4.400075
H	-2.836647	-1.248214	-0.767977	C	2.479062	0.476740	-5.001434

H	1.063883	-1.599797	-3.355341	H	0.519922	3.305771	-5.597827
H	0.208799	-1.954128	-4.879390	H	1.187431	2.262586	-6.900899
H	-1.247629	-3.439391	-3.499201	H	3.192193	0.325244	-4.180344
H	-2.906458	-1.001557	-3.080815	H	2.212034	-0.491709	-5.452262
H	-2.413073	-1.572934	-4.700434	H	2.936637	1.128065	-5.763083
H	-0.583805	2.447883	-6.730412				

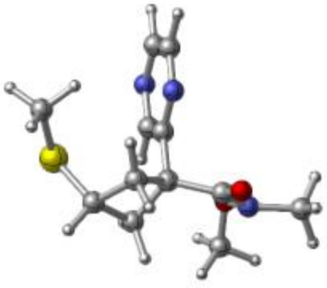

Zero-point correction= 0.282320 (Hartree/Particle)

Thermal correction to Energy= 0.300840

Thermal correction to Enthalpy= 0.301784

Thermal correction to Gibbs Free Energy= 0.234592

Sum of electronic and zero-point Energies= -1179.173655

Sum of electronic and thermal Energies= -1179.155135

Sum of electronic and thermal Enthalpies= -1179.154191

Sum of electronic and thermal Free Energies= -1179.221383

UB3LYP-D3/def2TZVPP

E(scf)= -1180.46849622

UM062X-D3/def2TZVPP

E(scf)= -1180.0659508

UB3LYP-D3/def2TZVPP (gas)

E(scf)= -1180.39329908

1a

E(scf) = -701.506498539 a.u.

ν_{min} = 75.1165 cm^{-1}

C	-0.969871	0.189121	-0.973877	H	1.109922	3.334299	-0.753669
C	0.439945	0.138264	-0.836375	H	-1.395035	3.425550	-0.997094
N	1.168460	1.243312	-0.759247	S	-1.866361	-1.335449	-1.070728
C	0.517429	2.416161	-0.816397	C	-3.574926	-0.733141	-1.237038
C	-0.869931	2.465379	-0.951299	H	-3.684104	-0.125112	-2.144975
N	-1.612244	1.354096	-1.029884	H	-4.202679	-1.632104	-1.306327
H	0.960762	-0.825258	-0.790724	H	-3.862846	-0.138204	-0.360046

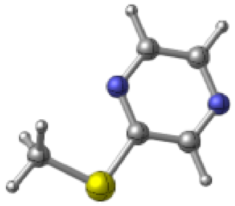

Zero-point correction= 0.105120 (Hartree/Particle)

Thermal correction to Energy= 0.112518

Thermal correction to Enthalpy= 0.113462

Thermal correction to Gibbs Free Energy= 0.072649

Sum of electronic and zero-point Energies= -701.401379

Sum of electronic and thermal Energies= -701.393981

Sum of electronic and thermal Enthalpies= -701.393036

Sum of electronic and thermal Free Energies= -701.433849

UB3LYP-D3/def2TZVPP

E(scf)= -701.985336858

UM062X-D3/def2TZVPP

E(scf)= -701.811342735

UB3LYP-D3/def2TZVPP (gas)

E(scf)= -701.985251745

H(EWG=C(O)NMeOMe)

E(scf) = -1410.33206619 a.u.

ν_{min} = 14.3577 cm^{-1}

C	-2.240442	1.053597	0.490036	C	-2.661203	2.171285	-3.840356
C	-2.390158	0.152838	-0.691062	H	-2.778250	2.622756	-1.740094
C	-1.787393	-1.064283	-0.062472	C	-2.294340	-0.163122	-4.425114
C	-1.027107	0.156234	0.400226	H	-2.126988	-1.542471	-2.783053
C	0.244313	0.755786	-0.130065	C	-2.487916	1.172363	-4.810478
N	1.237827	-0.070458	-0.558040	H	-2.818490	3.207862	-4.145482
O	0.366578	1.979564	-0.140602	H	-2.169495	-0.938821	-5.183372
C	2.557136	0.407596	-0.932606	H	-2.509339	1.434527	-5.870808
O	1.122222	-1.428352	-0.322894	S	-0.279604	-0.191113	2.857563
C	1.217229	-2.208134	-1.521210	C	0.330287	1.490407	3.163236
H	-2.086045	2.124056	0.331793	H	0.794429	1.902658	2.258306
H	-2.898936	0.821738	1.340165	H	-0.554716	2.077540	3.441448
H	-2.388002	-1.523832	0.737210	H	1.051872	1.463559	3.989836
H	-1.293671	-1.803481	-0.693218	C	1.223800	-1.100222	2.595280
H	2.598275	1.483994	-0.733354	C	1.140393	-2.505204	2.499931
H	3.310510	-0.116478	-0.327355	N	2.369316	-0.446574	2.457893
H	2.756012	0.230209	-2.000420	N	2.219286	-3.232899	2.239740
H	0.981474	-3.234513	-1.211965	H	0.184181	-3.023024	2.631579
H	0.495488	-1.861670	-2.278656	C	3.453586	-1.185339	2.206473
H	2.236042	-2.178590	-1.938385	C	3.376008	-2.577335	2.082499
C	-2.425440	0.500363	-2.083454	H	4.408535	-0.662702	2.094711
C	-2.635267	1.843686	-2.489829	H	4.270490	-3.166678	1.858348
C	-2.264110	-0.500781	-3.077345				

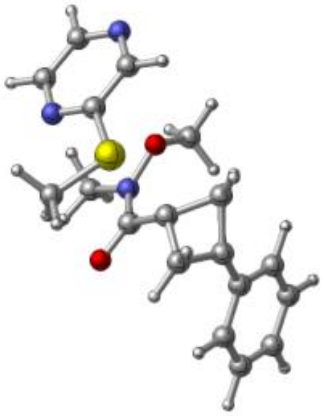

Zero-point correction= 0.361485 (Hartree/Particle)

Thermal correction to Energy= 0.385554

Thermal correction to Enthalpy= 0.386499

Thermal correction to Gibbs Free Energy= 0.305405

Sum of electronic and zero-point Energies= -1409.970581

Sum of electronic and thermal Energies= -1409.946512

Sum of electronic and thermal Enthalpies= -1409.945568

Sum of electronic and thermal Free Energies= -1410.026661

UB3LYP-D3/def2TZVPP

E(scf) = -1411.590257

UM062X-D3/def2TZVPP

E(scf)= -1411.07916

UB3LYP-D3/def2TZVPP (gas)

E(scf)= -1411.52573107

TS5’’(EWG=C(O)NMeOMe)

E(scf) = -1410.31520272 a.u.

ν_{min} = -338.4790 cm^{-1}

C	-0.069243	-0.236599	-1.922355	C	-0.626526	2.220089	-1.111730
C	1.322141	-0.690052	-1.581128	N	-0.081313	3.361431	-0.583334
C	1.648721	0.657763	-1.005816	O	-1.769882	2.195831	-1.579001
C	0.175588	0.992453	-1.077366	C	-0.843534	4.569962	-0.345708

O	1.096741	3.233411	0.133767
C	2.178633	3.977680	-0.437263
H	-0.113194	0.078855	-2.986497
H	-0.930953	-0.898109	-1.751267
H	2.206240	0.744717	-0.061753
H	2.198203	1.265596	-1.753718
H	-1.789534	4.479682	-0.890916
H	-1.044964	4.685845	0.730662
H	-0.297508	5.451947	-0.711294
H	3.056915	3.709597	0.164381
H	2.341826	3.700057	-1.491209
H	2.004007	5.062851	-0.361932
C	2.005102	-1.909430	-1.666127
C	1.376719	-3.055697	-2.241215
C	3.343751	-2.027046	-1.182419
C	2.063442	-4.256772	-2.330631
H	0.352072	-2.978202	-2.610366
C	4.016912	-3.235256	-1.278563
H	3.832074	-1.158449	-0.735954
C	3.381651	-4.351163	-1.851049
H	1.580315	-5.131001	-2.771744
H	5.040317	-3.321668	-0.907847
H	3.916457	-5.301178	-1.921699
C	0.003547	1.330205	2.162705
C	1.336537	1.362968	2.621108
N	1.803583	2.418081	3.279395
C	0.952851	3.426003	3.501459
C	-0.380800	3.365026	3.080753
N	-0.850892	2.316769	2.400362
H	2.013799	0.518447	2.452635
H	1.328985	4.304554	4.034699
H	-1.075291	4.183194	3.295533
S	-0.511256	-0.066446	1.192163
C	-2.300470	0.191292	1.032989
H	-2.513021	1.028904	0.356113
H	-2.696143	-0.745725	0.619694
H	-2.722505	0.379093	2.028271

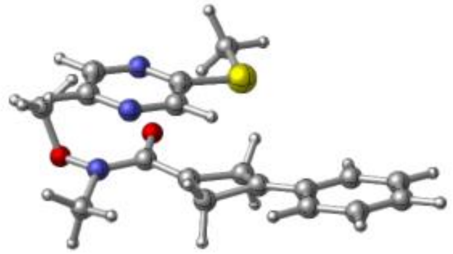

Zero-point correction= 0.358199 (Hartree/Particle)

Thermal correction to Energy= 0.382149

Thermal correction to Enthalpy= 0.383093

Thermal correction to Gibbs Free Energy= 0.302358

Sum of electronic and zero-point Energies= -1409.957004

Sum of electronic and thermal Energies= -1409.933054

Sum of electronic and thermal Enthalpies= -1409.932109

Sum of electronic and thermal Free Energies= -1410.012845

TS5–α (EWG=C(O)NMeOMe)

E(scf) = -1410.33149353 a.u.

ν_{min} = -152.4232 cm^{-1}

C	-2.111680	1.076054	0.486127	C	-2.324304	-0.413726	-3.105585
C	-2.346403	0.203096	-0.709406	C	-2.767645	2.268012	-3.805508
C	-1.733371	-1.027314	-0.105670	H	-2.781979	2.686517	-1.694339
C	-0.925633	0.121752	0.475231	C	-2.419842	-0.053810	-4.444512
C	0.349998	0.689265	-0.115777	H	-2.165042	-1.459000	-2.835148
N	1.304341	-0.171038	-0.565457	C	-2.638418	1.286263	-4.800172
O	0.505525	1.907039	-0.146223	H	-2.942526	3.309066	-4.085687
C	2.629688	0.263345	-0.971913	H	-2.325998	-0.816819	-5.220291
O	1.163497	-1.518886	-0.285165	H	-2.712259	1.565105	-5.853882
C	1.233246	-2.341756	-1.456028	S	-0.333142	-0.262185	2.684380
H	-1.898889	2.139839	0.341953	C	0.168851	1.431843	3.099685
H	-2.805308	0.893202	1.322744	H	0.804750	1.845508	2.308256
H	-2.360829	-1.520710	0.654521	H	-0.763598	2.003377	3.192434
H	-1.246291	-1.758043	-0.753111	H	0.700682	1.406861	4.059476
H	2.687736	1.348455	-0.834209	C	1.233458	-1.107540	2.567662
H	3.379455	-0.236468	-0.341242	C	1.226894	-2.515101	2.555492
H	2.818813	0.021893	-2.028463	N	2.343681	-0.395327	2.452483
H	0.974196	-3.350364	-1.109474	N	2.358790	-3.192143	2.397951
H	0.515859	-2.005928	-2.222184	H	0.294892	-3.078576	2.669791
H	2.250253	-2.351467	-1.878230	C	3.480471	-1.081100	2.307157
C	-2.443506	0.569274	-2.085078	C	3.484085	-2.480994	2.263948
C	-2.674824	1.919738	-2.463129	H	4.409957	-0.511128	2.216678

H	4.420744	-3.029540	2.124498

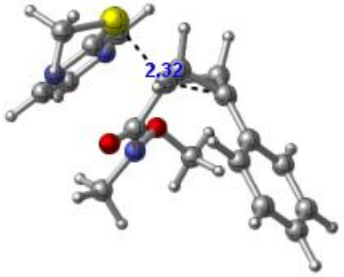

Zero-point correction= 0.360942 (Hartree/Particle)

Thermal correction to Energy= 0.384369

Thermal correction to Enthalpy= 0.385313

Thermal correction to Gibbs Free Energy= 0.305656

Sum of electronic and zero-point Energies= -1409.970552

Sum of electronic and thermal Energies= -1409.947124

Sum of electronic and thermal Enthalpies= -1409.946180

Sum of electronic and thermal Free Energies= -1410.025838

UB3LYP-D3/def2TZVPP

E(scf)= -1411.59009275

UM062X-D3/def2TZVPP

E(scf)= -1411.07873178

UB3LYP-D3/def2TZVPP (gas)

E(scf) = -1411.52542369

TS5'–β (EWG=C(O)NMeOMe)

E(scf) = -1410.32503146 a.u.

ν_{min} = -148.5623 cm^{-1}

C	-2.184624	0.150726	-0.550926	C	-1.308832	1.324585	-0.211375
C	-0.820513	-0.502656	-0.360866	C	-0.860375	2.451165	-1.061331
C	-0.351575	0.545696	0.641683	N	-1.822213	3.083724	-1.796776

O	0.322956	2.787050	-1.081615	H	1.903308	-0.593391	-0.497714
C	-1.551864	4.089080	-2.802141	C	1.548063	-2.029402	-3.564240
O	-3.115325	2.593668	-1.776091	H	-0.336811	-2.415185	-4.560790
C	-3.947190	3.311333	-0.853569	H	3.260975	-1.523807	-2.340783
H	-2.660923	0.124647	-1.535438	H	2.150865	-2.448550	-4.373041
H	-2.903524	-0.104022	0.244601	S	-1.168753	-2.547797	0.882584
H	-0.727806	0.372606	1.662800	C	-0.972901	-3.711701	-0.453830
H	0.698407	0.856014	0.640149	C	-2.107897	-4.059229	-1.210557
H	-0.497929	4.378087	-2.717327	N	0.238436	-4.172918	-0.736674
H	-1.745920	3.681816	-3.807779	N	-1.996757	-4.860288	-2.267624
H	-2.187418	4.972905	-2.640821	H	-3.101233	-3.678037	-0.950541
H	-4.943598	2.859848	-0.948358	C	0.344771	-4.975600	-1.795631
H	-3.580389	3.193626	0.179610	C	-0.775768	-5.312592	-2.568181
H	-4.001079	4.381220	-1.112345	H	1.338047	-5.360412	-2.044770
C	-0.003299	-0.962492	-1.479236	H	-0.673019	-5.964049	-3.441466
C	-0.620184	-1.480171	-2.641213	C	0.460967	-2.615646	1.670817
C	1.405650	-0.987552	-1.384654	H	1.244986	-2.448398	0.922501
C	0.150702	-2.011258	-3.671381	H	0.463078	-1.831549	2.438602
H	-1.706927	-1.477603	-2.730987	H	0.588972	-3.602801	2.133563
C	2.172331	-1.512560	-2.423185				

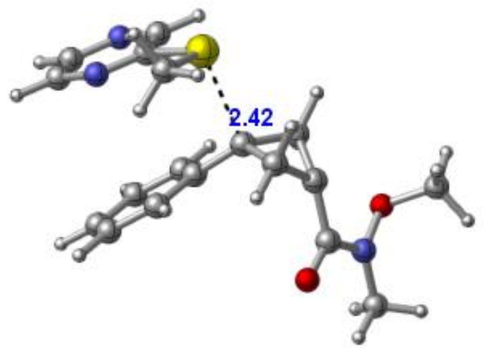

Zero-point correction= 0.360150 (Hartree/Particle)

Thermal correction to Energy= 0.383849

Thermal correction to Enthalpy= 0.384793

Thermal correction to Gibbs Free Energy= 0.304308

Sum of electronic and zero-point Energies= -1409.964881

Sum of electronic and thermal Energies= -1409.941183

Sum of electronic and thermal Enthalpies= -1409.940239

Sum of electronic and thermal Free Energies= -1410.020723

UB3LYP-D3/def2TZVPP

E(scf)= -1411.58583654

UM062X-D3/def2TZVPP

E(scf)= -1411.07364608

UB3LYP-D3/def2TZVPP (gas)

E(scf)= -1411.51811793

[1b]$^{+\cdot}$

E(scf) = -1159.52400544 a.u.

ν_{min} = 68.3439 cm^{-1}

C	-0.673668	-0.142810	0.436416	H	-2.177738	1.316635	1.017550
C	-0.433002	-1.490347	-0.049622	N	0.428267	0.594571	0.647237
C	-1.487278	-2.362508	-0.308504	S	1.273348	-1.756425	-0.220994
C	-2.782566	-1.891300	-0.083539	S	3.109478	0.522789	0.500506
C	-3.040872	-0.569714	0.392937	C	1.523482	-0.089326	0.356525
C	-2.010098	0.302484	0.653230	C	2.813039	2.206377	1.115268
H	-1.314744	-3.375708	-0.672788	H	2.298091	2.154095	2.083189
H	-3.627644	-2.555140	-0.278469	H	3.810462	2.650996	1.222437
H	-4.075168	-0.258359	0.548920	H	2.210062	2.759870	0.384025

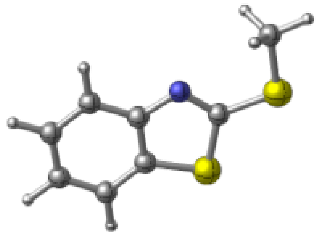

UB3LYP-D3/def2TZVPP

E(scf)= -1160.20270761

UM062X-D3/def2TZVPP

E(scf)= -1159.95969592

UB3LYP-D3/def2TZVPP (gas)

E(scf)= -1160.13539234

TS3–β

E(scf) = -1637.64545225 a.u.

ν_{min} = -71.0382 cm^{-1}

C	-0.816425	0.067214	1.076133	C	0.622044	-1.574885	0.771313
C	-1.668809	-1.020949	1.415377	C	3.086740	-1.087434	-0.376309
C	-2.999815	-0.816767	1.799144	H	3.053228	-0.175821	0.231472
C	-3.468430	0.495204	1.839711	H	4.111021	-1.472309	-0.463662
C	-2.633982	1.584301	1.506538	H	2.655694	-0.902187	-1.368760
C	-1.313139	1.383436	1.125127	C	3.107332	-0.592494	2.999080
H	-3.649200	-1.654745	2.057716	C	3.085853	-2.068697	2.672918
H	-4.503050	0.682598	2.135738	C	2.128752	-2.490089	3.748942
H	-3.035420	2.599045	1.550385	C	3.011558	-1.394015	4.249595
H	-0.655441	2.214708	0.865475	C	4.091612	-1.656930	5.251870
N	0.468523	-0.289091	0.725882	N	4.513458	-0.566676	5.947794
S	-0.789369	-2.525901	1.258383	O	4.543615	-2.786049	5.403916
S	2.132627	-2.407122	0.429140	C	5.387343	-0.647772	7.103095

O	3.707141	0.560051	5.867527	H	5.838279	-1.647053	7.110665
C	4.408096	1.697155	5.351058	H	6.184168	0.107291	7.040025
H	4.023243	-0.026638	2.804014	H	4.804269	-0.492465	8.024691
H	2.190134	-0.053596	2.707123	H	3.647876	2.483100	5.253396
H	4.008355	-2.626207	2.493157	H	5.194094	2.035135	6.045469
H	2.245921	-3.482349	4.195004	H	4.852988	1.480979	4.366912
H	1.081251	-2.197807	3.571049				

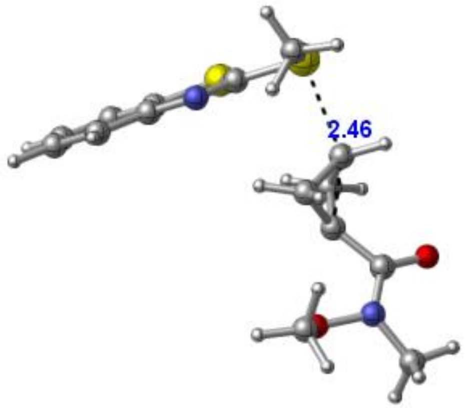

Zero-point correction= 0.304420 (Hartree/Particle)

Thermal correction to Energy= 0.326133

Thermal correction to Enthalpy= 0.327077

Thermal correction to Gibbs Free Energy= 0.250010

Sum of electronic and zero-point Energies= -1637.341032

Sum of electronic and thermal Energies= -1637.319319

Sum of electronic and thermal Enthalpies= -1637.318375

Sum of electronic and thermal Free Energies= -1637.395443

UB3LYP-D3/def2TZVPP

E(scf)= -1638.86099957

UM062X-D3/def2TZVPP

E(scf)= -1638.39763664

UB3LYP-D3/def2TZVPP (gas)

E(scf)= -1638.79450828

TS4'

E(scf) = -1637.63642399 a.u.

ν_{min} = -400.1370 cm^{-1}

C	-1.575641	0.454980	0.980722
C	-1.866929	-0.850807	0.481000
C	-3.177573	-1.246579	0.193752
C	-4.198579	-0.319202	0.401693
C	-3.925811	0.976696	0.891829
C	-2.627228	1.372835	1.183299
H	-3.395759	-2.246477	-0.184979
H	-5.229665	-0.603149	0.179510
H	-4.751052	1.675413	1.045271
H	-2.400713	2.368824	1.567469
N	-0.250143	0.701787	1.220077
S	-0.385050	-1.767763	0.304343
S	2.272243	-0.239857	0.739294
C	0.465588	-0.382360	1.058879
C	2.549670	1.556453	0.666994
H	1.660669	2.061564	1.063792
H	3.444295	1.756242	1.272868
H	2.716304	1.802794	-0.388488
C	1.855585	0.015129	3.462289
C	0.912883	-1.123268	3.193521
C	2.044025	-2.019018	2.771244
C	2.814628	-0.697872	2.470501
C	4.315831	-0.462092	2.618523
N	5.148384	-1.433564	2.167859
O	4.706624	0.591193	3.107919
C	6.578878	-1.447561	2.415476
O	4.586892	-2.626862	1.747011
C	4.796069	-2.871312	0.348715
H	1.533952	1.049061	3.274682
H	2.303788	-0.031704	4.471362
H	-0.092617	-1.337789	3.557819
H	1.901589	-2.760870	1.971634
H	2.530272	-2.530664	3.620843
H	6.847062	-0.496559	2.889427
H	6.826196	-2.286022	3.084655
H	7.136755	-1.551738	1.473341
H	4.231162	-3.787423	0.132841
H	4.409837	-2.041398	-0.263967
H	5.861548	-3.037579	0.124269

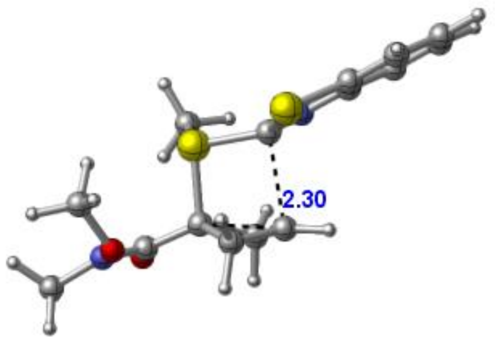

Zero-point correction= 0.305250 (Hartree/Particle)

Thermal correction to Energy= 0.325798

Thermal correction to Enthalpy= 0.326742

Thermal correction to Gibbs Free Energy= 0.254723

Sum of electronic and zero-point Energies= -1637.331174

Sum of electronic and thermal Energies= -1637.310626

Sum of electronic and thermal Enthalpies= -1637.309682

Sum of electronic and thermal Free Energies= -1637.381701

UB3LYP-D3/def2TZVPP

E(scf)= -1638.84974375

UM062X-D3/def2TZVPP

E(scf)= -1638.38746514

UB3LYP-D3/def2TZVPP (gas)

E(scf)= -1638.78443369

G

E(scf) = -1637.71038451 a.u.

ν_{min} = 24.7428 cm^{-1}

C	-0.407373	-0.042236	0.243876	S	3.032452	0.383450	0.692800
C	-1.417101	-0.997832	0.517822	C	0.433775	-0.907405	2.146186
C	-2.529383	-1.140298	-0.319025	C	3.035747	2.165790	0.976534
C	-2.604822	-0.320863	-1.445299	H	2.132570	2.453516	1.531070
C	-1.599773	0.625287	-1.729244	H	3.940145	2.433477	1.542398
C	-0.498166	0.776867	-0.891694	H	3.043433	2.644306	-0.011435
H	-3.311255	-1.868958	-0.098709	C	2.774393	-1.737372	2.492437
H	-3.460136	-0.415558	-2.117873	C	3.327963	-0.296842	2.364538
H	-1.688242	1.251696	-2.619419	C	2.319029	0.151756	3.447996
H	0.282481	1.509325	-1.103171	C	1.498187	-1.148469	3.182921
N	0.594701	-0.028344	1.197524	C	0.998731	-1.950868	4.390185
S	-1.024505	-1.863562	1.993835	N	0.077401	-1.322954	5.176484

O	1.409349	-3.077805	4.624769	H	2.803128	0.119993	4.432933
C	-0.713961	-1.996834	6.188591	H	-0.284480	-2.994137	6.337797
O	-0.419406	-0.107872	4.725478	H	-0.684667	-1.445831	7.139978
C	-0.121388	0.969405	5.622030	H	-1.757454	-2.084620	5.846323
H	2.625045	-2.330126	1.579665	H	-0.450752	1.877217	5.099724
H	3.361614	-2.321583	3.212178	H	-0.678936	0.870165	6.567255
H	4.398346	-0.137807	2.565443	H	0.957927	1.027677	5.832224
H	1.793610	1.106304	3.329999				

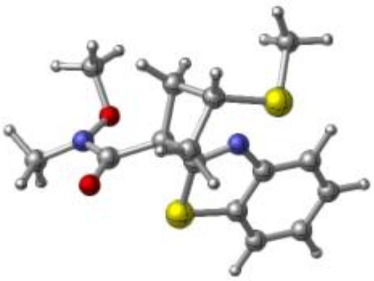

Zero-point correction= 0.309094 (Hartree/Particle)

Thermal correction to Energy= 0.329925

Thermal correction to Enthalpy= 0.330869

Thermal correction to Gibbs Free Energy= 0.258206

Sum of electronic and zero-point Energies= -1637.401291

Sum of electronic and thermal Energies= -1637.380459

Sum of electronic and thermal Enthalpies= -1637.379515

Sum of electronic and thermal Free Energies= -1637.45217

UB3LYP-D3/def2TZVPP

E(scf) = -1638.91994312

UM062X-D3/def2TZVPP

E(scf) = -1638.45935201

UB3LYP-D3/def2TZVPP (gas)

E(scf) = -1638.85136422

G’

E(scf) = -1637.71189225 a.u.

ν_{min} = 28.1068 cm^{-1}

C	-1.376370	-0.149766	1.026534	C	2.062531	-2.092238	2.678766
C	-2.435985	-0.893474	1.605158	C	2.704946	-0.688252	2.514528
C	-3.724396	-0.865468	1.059088	C	4.223586	-0.463472	2.625497
C	-3.931017	-0.091668	-0.082432	N	5.025690	-1.263659	1.874238
C	-2.880286	0.645118	-0.665863	O	4.652284	0.421564	3.353528
C	-1.599860	0.627495	-0.120460	C	6.465478	-1.342221	2.044657
H	-4.539930	-1.432234	1.511639	O	4.429215	-2.314438	1.197941
H	-4.926280	-0.056742	-0.530951	C	4.614019	-2.232568	-0.221900
H	-3.075205	1.241011	-1.560119	H	1.439281	0.784882	3.729151
H	-0.781440	1.196122	-0.564864	H	2.512234	-0.313349	4.642262
N	-0.189271	-0.287884	1.719713	H	0.667979	-1.985187	4.469671
S	-1.846953	-1.756413	3.017012	H	1.754398	-2.627051	1.771457
S	2.174445	0.077142	0.921613	H	2.719097	-2.742098	3.271361
C	-0.255714	-1.090444	2.744597	H	6.766365	-0.538773	2.726450
C	2.297606	1.841175	1.296206	H	6.732991	-2.320681	2.473300
H	1.379241	2.168299	1.802373	H	6.979380	-1.209903	1.081363
H	3.174519	2.003615	1.940492	H	3.989998	-3.034622	-0.637004
H	2.409139	2.364010	0.337746	H	4.278435	-1.257452	-0.609372
C	1.889345	-0.215174	3.744142	H	5.665293	-2.406358	-0.501482
C	0.947179	-1.438362	3.561254				

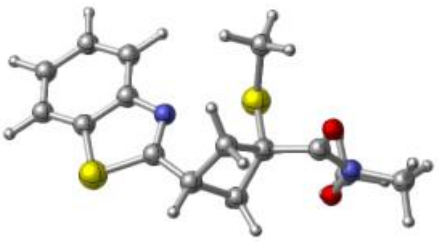

Zero-point correction= 0.309555 (Hartree/Particle)

Thermal correction to Energy= 0.330245

Thermal correction to Enthalpy= 0.331189

Thermal correction to Gibbs Free Energy= 0.258824

Sum of electronic and zero-point Energies= -1637.402337

Sum of electronic and thermal Energies= -1637.381647

Sum of electronic and thermal Enthalpies= -1637.380703

Sum of electronic and thermal Free Energies= -1637.453068

UB3LYP-D3/def2TZVPP

E(scf)= -1638.92005573

UM062X-D3/def2TZVPP

E(scf) = -1638.45837334

UB3LYP-D3/def2TZVPP (gas)

E(scf) = -1638.85536369

TS5''(EWG=CO_2Me)

E(scf) -1315.76893333 a.u.

ν_{min} = -404.3887 cm^{-1}

C	-0.846767	0.603583	-1.909312	H	2.330674	4.348870	1.140329
C	0.037429	-0.600884	-1.744701	H	1.611096	4.949289	-0.401352
C	1.002155	0.182259	-0.900056	C	-0.058459	-1.942738	-2.130089
C	-0.035215	1.281110	-0.827991	C	-1.161515	-2.394603	-2.917518
C	0.111811	2.710930	-0.571422	C	0.949797	-2.881397	-1.751414
O	-0.704141	3.550004	-0.911730	C	-1.241022	-3.721650	-3.309708
C	1.440672	4.343938	0.501392	H	-1.939644	-1.686507	-3.209358
H	-0.636020	1.104522	-2.877357	C	0.855435	-4.205185	-2.151281
H	-1.939603	0.500112	-1.835141	H	1.794424	-2.548356	-1.144912
H	1.431271	-0.266238	0.008604	C	-0.236536	-4.628897	-2.929099
H	1.854857	0.535270	-1.516399	H	-2.084381	-4.064041	-3.912721
H	0.574421	4.746485	1.045523	H	1.627605	-4.920417	-1.860966

H	-0.306435	-5.674198	-3.238882	H	1.934063	3.291476	4.750691
C	-0.328156	1.450767	2.381558	H	-0.186157	4.339621	3.876169
C	0.840301	0.844136	2.884707	S	-1.321514	0.539609	1.217944
N	1.640239	1.502057	3.717709	C	-2.743076	1.639065	0.975670
C	1.280066	2.742523	4.066306	H	-3.439362	1.084747	0.332971
C	0.105371	3.327807	3.578484	H	-3.203944	1.844163	1.950055
N	-0.691790	2.679736	2.725797	H	-2.424057	2.573042	0.495280
H	1.113869	-0.180408	2.609631	O	1.213993	2.972358	0.147400

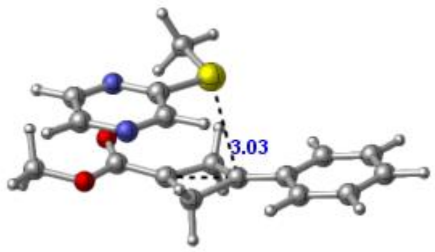

Zero-point correction= 0.314245 (Hartree/Particle)

Thermal correction to Energy= 0.335452

Thermal correction to Enthalpy= 0.336396

Thermal correction to Gibbs Free Energy= 0.261581

Sum of electronic and zero-point Energies= -1315.454688

Sum of electronic and thermal Energies= -1315.433481

Sum of electronic and thermal Enthalpies= -1315.432537

Sum of electronic and thermal Free Energies= -1315.507353

H(EWG=CO_2Me)

E(scf) = -1315.78838240 a.u.

ν_{min} = 17.0612 cm^{-1}

C	-2.283302	1.132236	0.506334	C	-1.834810	-1.007574	-0.005942
C	-2.390259	0.214348	-0.674864	C	-1.097124	0.205836	0.494872

C	0.219578	0.660050	-0.031716	H	-2.774054	3.233128	-4.163559
O	0.578657	1.817821	-0.011279	H	-1.904161	-0.887302	-5.144236
C	2.237948	-0.048392	-1.030022	H	-2.313617	1.465613	-5.861153
H	-2.097063	2.197263	0.338509	S	-0.305580	-0.203866	2.898671
H	-2.995683	0.933706	1.320473	C	0.197294	1.494069	3.289441
H	-2.490099	-1.466839	0.749119	H	0.672060	1.957634	2.415265
H	-1.280302	-1.737529	-0.602153	H	-0.727673	2.020657	3.558148
H	2.832444	0.509952	-0.294006	H	0.892298	1.473445	4.138649
H	2.708377	-1.010872	-1.257475	C	1.254739	-0.982041	2.537792
H	2.126867	0.554521	-1.942757	C	1.263464	-2.363581	2.255996
C	-2.362917	0.552721	-2.068723	N	2.360364	-0.247935	2.517882
C	-2.607332	1.885803	-2.492832	N	2.398008	-2.985111	1.952147
C	-2.115349	-0.445892	-3.047844	H	0.340177	-2.952577	2.278005
C	-2.586953	2.205736	-3.844703	C	3.498888	-0.880607	2.224255
H	-2.818135	2.661249	-1.755139	C	3.516961	-2.251128	1.936302
C	-2.097010	-0.114895	-4.396990	H	4.418923	-0.288599	2.208058
H	-1.943875	-1.478476	-2.740673	H	4.455341	-2.755882	1.687150
C	-2.328890	1.209668	-4.799194	O	0.943125	-0.361414	-0.486141

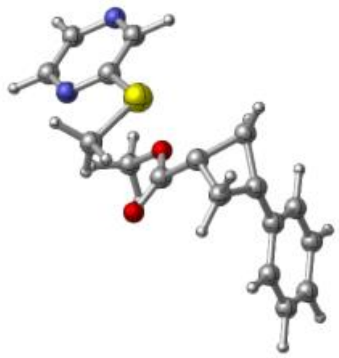

Zero-point correction= 0.317108 (Hartree/Particle)

Thermal correction to Energy= 0.338688

Thermal correction to Enthalpy= 0.339632

Thermal correction to Gibbs Free Energy= 0.263203

Sum of electronic and zero-point Energies= -1315.471274

Sum of electronic and thermal Energies= -1315.449694

Sum of electronic and thermal Enthalpies= -1315.448750

Sum of electronic and thermal Free Energies= -1315.525179

TS5–α (EWG=CO_2Me)

E(scf) = -1315.78792167 a.u.

ν_{min} = -144.9439 cm^{-1}

C	-2.147188	1.132288	0.505308	C	-2.238717	-0.075467	-4.417265
C	-2.315616	0.231212	-0.687194	H	-1.976706	-1.447928	-2.780456
C	-1.716979	-0.986309	-0.037029	C	-2.501390	1.249763	-4.798475
C	-0.968106	0.182844	0.569404	H	-2.914479	3.267320	-4.123898
C	0.336085	0.647284	-0.012261	H	-2.085773	-0.842674	-5.179303
O	0.686303	1.806777	-0.011419	H	-2.551089	1.511800	-5.857901
C	2.342822	-0.058283	-1.033797	S	-0.348644	-0.225432	2.759110
H	-1.930949	2.194721	0.348559	C	0.123249	1.462337	3.228025
H	-2.882946	0.967377	1.308413	H	0.723391	1.920851	2.433189
H	-2.380013	-1.491373	0.683554	H	-0.820065	2.003467	3.376532
H	-1.149843	-1.697141	-0.646680	H	0.690407	1.411817	4.166172
H	2.952704	0.484611	-0.298446	C	1.240961	-1.013387	2.529207
H	2.803357	-1.019806	-1.284136	C	1.276533	-2.408390	2.349565
H	2.219754	0.560203	-1.934282	N	2.332604	-0.262007	2.507931
C	-2.369646	0.575758	-2.070116	N	2.433692	-3.029238	2.137868
C	-2.643392	1.911348	-2.474184	H	0.362213	-3.010497	2.378488
C	-2.174123	-0.414896	-3.071481	C	3.492421	-0.891945	2.310165
C	-2.705374	2.238446	-3.823174	C	3.540513	-2.279369	2.117779
H	-2.810021	2.682173	-1.720059	H	4.403705	-0.286905	2.295277

H	4.495927	-2.783922	1.944393	O	1.055744	-0.372485	-0.471719

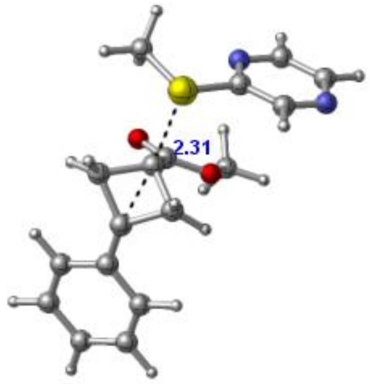

Zero-point correction= 0.316954 (Hartree/Particle)

Thermal correction to Energy= 0.337692

Thermal correction to Enthalpy= 0.338637

Thermal correction to Gibbs Free Energy= 0.264702

Sum of electronic and zero-point Energies= -1315.470968

Sum of electronic and thermal Energies= -1315.450229

Sum of electronic and thermal Enthalpies= -1315.449285

Sum of electronic and thermal Free Energies= -1315.523220

I(EWG=CO_2Me)

E(scf) = -1315.79975686 a.u.

ν_{min} = 27.7567 cm^{-1}

C	-0.292764	-0.148747	-0.996858	H	-0.359505	-1.208722	-0.702402
C	1.057510	0.458097	-1.271665	H	1.510807	2.065394	0.231270
C	0.748470	1.696348	-0.471215	H	0.384428	2.546835	-1.073344
C	-0.478253	0.932458	0.124420	H	-4.092255	2.040833	2.401636
C	-1.817518	1.663040	0.118929	H	-3.643458	3.300970	1.188823
O	-2.215949	2.233505	-0.863993	H	-4.437871	1.783061	0.648118
C	-3.745923	2.223648	1.379117	C	2.259132	-0.075966	-1.773759
H	-1.035648	0.027173	-1.794033	C	2.316241	-1.406555	-2.287685

C	3.471104	0.678193	-1.733059	H	-0.685817	2.185541	2.900160
C	3.522077	-1.951274	-2.711811	H	0.419767	1.136772	3.870378
H	1.402681	-2.003928	-2.331883	C	1.468785	-0.593164	1.566372
C	4.668381	0.120162	-2.164032	C	1.539614	-1.964641	1.296269
H	3.453148	1.699888	-1.347022	N	2.530809	0.186970	1.660133
C	4.706260	-1.197562	-2.649937	N	2.725052	-2.529560	1.057483
H	3.548503	-2.975508	-3.092001	H	0.648132	-2.598733	1.272184
H	5.587319	0.709840	-2.117799	C	3.712652	-0.381054	1.432183
H	5.651624	-1.633092	-2.981480	C	3.802293	-1.747575	1.111668
S	-0.176198	0.155601	1.803416	H	4.604704	0.247034	1.497676
C	0.214592	1.561983	2.879456	H	4.774368	-2.202024	0.898629
H	1.090008	2.092751	2.489297	O	-2.456618	1.582738	1.280733

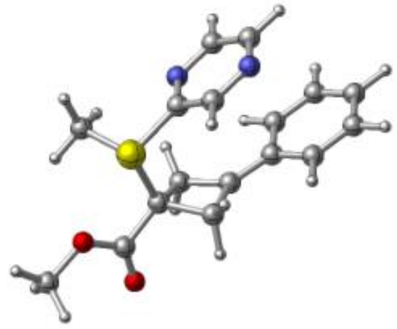

Zero-point correction= 0.317690 (Hartree/Particle)

Thermal correction to Energy= 0.338477

Thermal correction to Enthalpy= 0.339421

Thermal correction to Gibbs Free Energy= 0.266799

Sum of electronic and zero-point Energies= -1315.482066

Sum of electronic and thermal Energies= -1315.461280

Sum of electronic and thermal Enthalpies= -1315.460336

Sum of electronic and thermal Free Energies= -1315.532958

TS5'–β (EWG=CO_2Me)

E(scf) = -1315.77005326 a.u.

ν_{min} = -524.8473 cm^{-1}

C	0.769630	-1.498439	-2.138205	H	-0.922318	1.771880	-0.105112
C	0.129900	-0.528292	-1.110341	C	2.221213	2.628392	0.909896
C	-1.032815	-0.318819	-2.126769	H	4.002429	1.439406	0.593890
C	-0.634664	-1.673621	-2.668883	H	0.289087	3.593711	1.070465
C	-1.127082	-2.343186	-3.884695	H	2.752527	3.430600	1.427511
O	-2.207115	-2.110312	-4.386742	S	-0.747203	-1.651046	0.154021
C	-0.665942	-4.044382	-5.456947	C	-1.341052	-2.887499	-1.130279
H	1.386596	-0.916116	-2.842761	C	-2.772614	-2.941249	-1.320822
H	1.337455	-2.377948	-1.809886	N	-0.598728	-4.016378	-1.230689
H	-2.057219	-0.135460	-1.772222	N	-3.328893	-3.996098	-1.877578
H	-0.756955	0.484782	-2.829371	H	-3.408938	-2.090565	-1.055865
H	0.160941	-4.733249	-5.661723	C	-1.170825	-5.058044	-1.806553
H	-1.586199	-4.604602	-5.233118	C	-2.535639	-5.043203	-2.172611
H	-0.845903	-3.388546	-6.320905	H	-0.566360	-5.959234	-1.949518
C	0.857857	0.574674	-0.422366	H	-2.996214	-5.916552	-2.642219
C	2.244654	0.486033	-0.220400	C	0.590469	-2.519305	1.004773
C	0.157046	1.698101	0.048352	H	1.069138	-3.229421	0.320648
C	2.922959	1.512083	0.443818	H	1.282699	-1.738755	1.349876
H	2.798502	-0.379856	-0.590373	H	0.137212	-3.036754	1.859684
C	0.838048	2.720700	0.710313	O	-0.265336	-3.270865	-4.314943

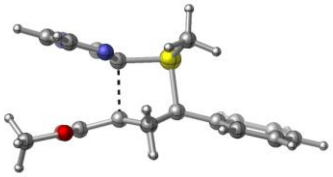

Zero-point correction= 0.316797 (Hartree/Particle)

Thermal correction to Energy= 0.336931

Thermal correction to Enthalpy= 0.337876

Thermal correction to Gibbs Free Energy= 0.267048

Sum of electronic and zero-point Energies= -1315.453256

Sum of electronic and thermal Energies= -1315.433122

Sum of electronic and thermal Enthalpies= -1315.432178

Sum of electronic and thermal Free Energies= -1315.503006

J'(EWG=CO_2Me)

E(scf) = -1315.83089065 a.u.

ν_{min} = 28.2601 cm^{-1}

C	0.526745	-1.857295	-1.914053	C	0.875234	2.591005	0.436705
C	-0.006529	-0.887153	-0.831570	H	-0.952027	1.499591	0.073072
C	-1.303506	-0.713983	-1.670559	C	2.274161	2.562275	0.391705
C	-0.920897	-1.975626	-2.501264	H	4.026438	1.389542	-0.100745
C	-0.974465	-1.785943	-4.010545	H	0.355532	3.486199	0.785813
O	-1.436553	-0.818667	-4.565689	H	2.849653	3.435928	0.706708
C	-0.318577	-2.745943	-6.069571	S	-0.470459	-1.734316	0.763661
H	1.190749	-1.316088	-2.601310	C	-1.627238	-3.226835	-2.017419
H	1.016974	-2.789760	-1.609280	C	-2.433561	-4.067054	-2.809036
H	-2.268596	-0.731138	-1.146226	N	-1.487316	-3.530703	-0.724142
H	-1.245685	0.189445	-2.290787	N	-3.045388	-5.137684	-2.305891
H	0.194255	-3.660259	-6.387914	H	-2.576938	-3.859942	-3.872043
H	-1.329174	-2.701667	-6.500973	C	-2.086805	-4.590709	-0.195349
H	0.247265	-1.856798	-6.383108	C	-2.875438	-5.410844	-1.012997
C	0.797787	0.319367	-0.425109	H	-1.946897	-4.788824	0.870561
C	2.201726	0.295932	-0.465270	H	-3.373599	-6.291637	-0.596658
C	0.139761	1.475103	0.034154	C	1.031008	-2.612565	1.240447
C	2.935113	1.414183	-0.059868	H	1.225335	-3.438555	0.543230
H	2.727493	-0.591946	-0.824138	H	1.871986	-1.903715	1.245755

H	0.857701	-3.001886	2.252016	O	-0.390315	-2.810069	-4.634764

Zero-point correction= 0.320442 (Hartree/Particle)
Thermal correction to Energy= 0.340822
Thermal correction to Enthalpy= 0.341766
Thermal correction to Gibbs Free Energy= 0.269707
Sum of electronic and zero-point Energies= -1315.510448
Sum of electronic and thermal Energies= -1315.490069
Sum of electronic and thermal Enthalpies= -1315.489125
Sum of electronic and thermal Free Energies= -1315.561184

TS6 (EWG=CO_2Me)

E(scf) = -1315.77509547 a.u.

ν_{min} = -471.1458 cm^{-1}

C	-0.273655	-0.238256	-0.862403	C	3.485513	0.573659	-1.575294
C	1.188811	0.168673	-0.727397	C	3.473281	-1.913045	-2.867836
C	0.745840	1.576349	-0.355008	H	1.465197	-2.141440	-2.110775
C	-0.565330	0.914118	0.143336	C	4.609197	0.153111	-2.285847
C	-1.883222	1.657143	0.070685	H	3.488353	1.541747	-1.070241
O	-2.284923	2.128655	-0.962893	C	4.606920	-1.090526	-2.929715
C	-3.764328	2.409163	1.295310	H	3.471383	-2.880853	-3.374347
H	-0.674382	0.044936	-1.848583	H	5.491323	0.795258	-2.337118
H	-0.607043	-1.257801	-0.620769	H	5.489105	-1.419282	-3.484252
H	1.347914	2.178832	0.336596	S	-0.195211	0.109983	1.772821
H	0.520652	2.168136	-1.256633	C	0.204261	1.457478	2.911439
H	-4.096863	2.350603	2.336961	H	1.067325	2.022529	2.543798
H	-3.634236	3.455813	0.986494	H	-0.706536	2.064616	2.981614
H	-4.484893	1.915679	0.628302	H	0.432680	0.981956	3.873997
C	2.338270	-0.247683	-1.505214	C	1.503355	-0.485817	1.182772
C	2.346994	-1.498622	-2.160122	C	1.631271	-1.927405	1.103373

N	2.549344	0.255447	1.633386	C	3.891970	-1.689218	1.280246
N	2.819745	-2.486741	1.093001	H	4.601869	0.277334	1.906033
H	0.747930	-2.565247	0.990119	H	4.883400	-2.148132	1.247898
C	3.735780	-0.323156	1.608808	O	-2.497978	1.720735	1.247033

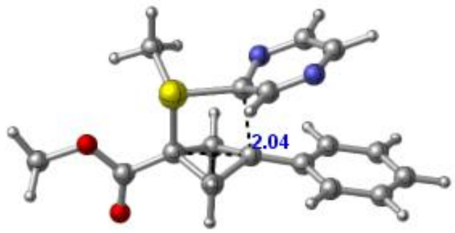

Zero-point correction= 0.316984 (Hartree/Particle)

Thermal correction to Energy= 0.337051

Thermal correction to Enthalpy= 0.337995

Thermal correction to Gibbs Free Energy= 0.267399

Sum of electronic and zero-point Energies= -1315.458112

Sum of electronic and thermal Energies= -1315.438045

Sum of electronic and thermal Enthalpies= -1315.437101

Sum of electronic and thermal Free Energies= -1315.507697

J(EWG=CO_2Me)

E(scf) = -1315.81149747 a.u.

ν_{min} = 32.3105 cm^{-1}

C	-0.032692	-0.611405	-0.515546	H	-0.434691	-0.447639	-1.523724
C	1.450624	-0.142267	-0.419757	H	-0.321220	-1.612706	-0.170160
C	0.909420	1.279030	-0.088727	H	1.464167	1.894731	0.627183
C	-0.419179	0.603425	0.365241	H	0.717973	1.853330	-1.004565
C	-1.707348	1.356591	-0.006196	H	-3.916894	2.841323	1.845912
O	-2.041943	1.517572	-1.149450	H	-3.304955	3.502701	0.282057
C	-3.543640	2.591147	0.847396	H	-4.278924	1.991524	0.293557

C	2.337503	-0.278164	-1.646411	H	1.256823	1.576257	2.908492
C	2.092724	-1.258975	-2.617039	H	-0.317766	2.420089	2.608155
C	3.442195	0.575876	-1.795617	H	-0.107882	1.362929	4.066759
C	2.936296	-1.379152	-3.727300	C	2.168580	-0.721445	0.795813
H	1.242074	-1.936583	-2.512019	C	2.072008	-2.087201	1.150464
C	4.281480	0.457184	-2.905731	N	2.987013	0.097032	1.479488
H	3.647327	1.330765	-1.032758	N	2.777346	-2.611556	2.154287
C	4.030119	-0.521099	-3.875546	H	1.420532	-2.765688	0.592647
H	2.734364	-2.146118	-4.479038	C	3.693927	-0.424906	2.474316
H	5.135527	1.130343	-3.013902	C	3.589810	-1.788534	2.815079
H	4.686385	-0.613553	-4.744365	H	4.358159	0.240577	3.034779
S	-0.434438	0.082548	2.110322	H	4.176063	-2.199842	3.643047
C	0.161560	1.518521	3.014118	O	-2.341842	1.819568	1.063314

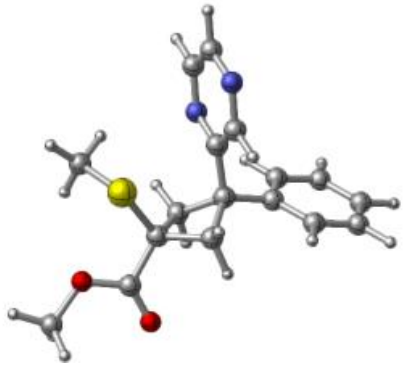

Zero-point correction= 0.319035 (Hartree/Particle)

Thermal correction to Energy= 0.339745

Thermal correction to Enthalpy= 0.340689

Thermal correction to Gibbs Free Energy= 0.267267

Sum of electronic and zero-point Energies= -1315.492462

Sum of electronic and thermal Energies= -1315.471753

Sum of electronic and thermal Enthalpies= -1315.470809

Sum of electronic and thermal Free Energies= -1315.544231

I'(**EWG=CO_2Me)**

E(scf) = -1315.79404044 a.u.

ν_{min} = 27.2441 cm^{-1}

C	0.857039	-1.465711	-2.255098	H	0.196732	3.499465	1.075857
C	0.128779	-0.582516	-1.192950	H	2.652706	3.358303	1.493947
C	-0.968129	-0.249542	-2.261499	S	-0.716073	-1.637449	0.166702
C	-0.376689	-1.341461	-3.095345	C	-1.441294	-3.022361	-0.759208
C	-0.996814	-2.201147	-4.076151	C	-2.808330	-2.977056	-1.062049
O	-2.135998	-2.054380	-4.491178	N	-0.648110	-4.016266	-1.121691
C	-0.708483	-4.128132	-5.414359	N	-3.358814	-3.957810	-1.778954
H	1.724436	-0.921857	-2.667878	H	-3.449348	-2.156022	-0.725697
H	1.185092	-2.479694	-1.984052	C	-1.200620	-4.995242	-1.834964
H	-2.032721	-0.323590	-1.988142	C	-2.565467	-4.955274	-2.170358
H	-0.797450	0.763160	-2.666792	H	-0.560834	-5.823422	-2.151139
H	0.078738	-4.870328	-5.589830	H	-3.012216	-5.758934	-2.763681
H	-1.615342	-4.619375	-5.029677	C	0.676812	-2.429927	1.006907
H	-0.960549	-3.607526	-6.350479	H	1.241093	-3.042817	0.294702
C	0.839436	0.514508	-0.457977	H	1.281723	-1.618166	1.429414
C	2.220767	0.436888	-0.216128	H	0.245427	-3.046497	1.806006
C	0.114807	1.621928	0.016726	O	-0.183450	-3.209193	-4.450300
C	2.869373	1.458232	0.483588	H	-4.108340	-4.532816	-2.945319
H	2.795973	-0.418336	-0.577553	C	0.560898	-2.521070	0.656281
C	0.765833	2.639840	0.714902	H	1.339044	-1.902203	1.119144
H	-0.961511	1.687853	-0.160859	H	0.134757	-3.218651	1.388881
C	2.143977	2.559593	0.949063	H	0.922744	-3.053017	-0.230823
H	3.944847	1.392826	0.662523				

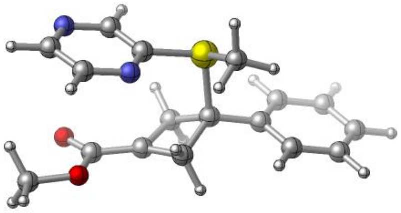

Zero-point correction= 0.317677 (Hartree/Particle)

Thermal correction to Energy= 0.338594

Thermal correction to Enthalpy= 0.339538

Thermal correction to Gibbs Free Energy= 0.266251

Sum of electronic and zero-point Energies= -1315.476363

Sum of electronic and thermal Energies= -1315.455446

Sum of electronic and thermal Enthalpies= -1315.454502

Sum of electronic and thermal Free Energies= -1315.527790

TS6' (EWG=CO_2Me)

E(scf) = -1315.77005326 a.u.

ν_{min} = -524.8473 cm^{-1}

C	0.769630	-1.498439	-2.138205	C	2.922959	1.512083	0.443818
C	0.129900	-0.528292	-1.110341	H	2.798502	-0.379856	-0.590373
C	-1.032815	-0.318819	-2.126769	C	0.838048	2.720700	0.710313
C	-0.634664	-1.673621	-2.668883	H	-0.922318	1.771880	-0.105112
C	-1.127082	-2.343186	-3.884695	C	2.221213	2.628392	0.909896
O	-2.207115	-2.110312	-4.386742	H	4.002429	1.439406	0.593890
C	-0.665942	-4.044382	-5.456947	H	0.289087	3.593711	1.070465
H	1.386596	-0.916116	-2.842761	H	2.752527	3.430600	1.427511
H	1.337455	-2.377948	-1.809886	S	-0.747203	-1.651046	0.154021
H	-2.057219	-0.135460	-1.772222	C	-1.341052	-2.887499	-1.130279
H	-0.756955	0.484782	-2.829371	C	-2.772614	-2.941249	-1.320822
H	0.160941	-4.733249	-5.661723	N	-0.598728	-4.016378	-1.230689
H	-1.586199	-4.604602	-5.233118	N	-3.328893	-3.996098	-1.877578
H	-0.845903	-3.388546	-6.320905	H	-3.408938	-2.090565	-1.055865
C	0.857857	0.574674	-0.422366	C	-1.170825	-5.058044	-1.806553
C	2.244654	0.486033	-0.220400	C	-2.535639	-5.043203	-2.172611
C	0.157046	1.698101	0.048352	H	-0.566360	-5.959234	-1.949518

H	-2.996214	-5.916552	-2.642219	H	1.282699	-1.738755	1.349876
C	0.590469	-2.519305	1.004773	H	0.137212	-3.036754	1.859684
H	1.069138	-3.229421	0.320648	O	-0.265336	-3.270865	-4.314943

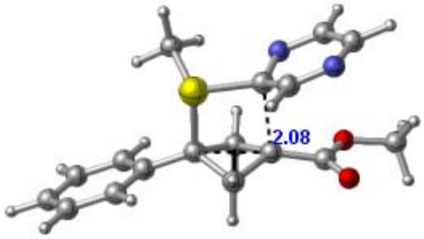

Zero-point correction= 0.316797 (Hartree/Particle)

Thermal correction to Energy= 0.336931

Thermal correction to Enthalpy= 0.337876

Thermal correction to Gibbs Free Energy= 0.267048

Sum of electronic and zero-point Energies= -1315.453256

Sum of electronic and thermal Energies= -1315.433122

Sum of electronic and thermal Enthalpies= -1315.432178

Sum of electronic and thermal Free Energies= -1315.503006

TS2

E(scf) = -1179.39691827 a.u.

ν_{min} = -442.7861 cm^{-1}

S	2.347265	-0.533893	0.987227	C	0.999400	-1.131087	3.472414
C	2.694633	1.233821	0.753824	C	2.104059	-2.108497	3.156092
H	1.810797	1.801500	1.075630	C	2.920858	-0.850173	2.739415
H	3.576047	1.472385	1.364718	C	4.430661	-0.660322	2.836995
H	2.900302	1.376496	-0.313726	N	5.208124	-1.657860	2.343805
C	2.001326	-0.022816	3.671832	O	4.875081	0.373260	3.320254

C	6.640131	-1.533503	2.139588	H	5.777557	-4.287005	2.142241
O	4.568142	-2.649774	1.616842	H	4.395262	-3.957686	3.250819
C	4.730827	-3.949164	2.202004	C	0.510075	-0.565391	1.412085
H	1.734894	1.010902	3.413078	C	-0.223021	-1.699288	0.928938
H	2.432677	-0.030351	4.688300	N	-0.080390	0.631405	1.546382
H	0.012351	-1.287475	3.912243	N	-1.539239	-1.658157	0.870964
H	1.948687	-2.920358	2.431893	H	0.287528	-2.626476	0.647749
H	2.540763	-2.540459	4.073406	C	-1.406958	0.656419	1.494120
H	6.971594	-0.620220	2.646754	C	-2.147177	-0.500263	1.191078
H	7.170151	-2.397071	2.566227	H	-1.905221	1.618472	1.648055
H	6.854664	-1.463863	1.061656	H	-3.238987	-0.466784	1.141215
H	4.099401	-4.615179	1.599740				

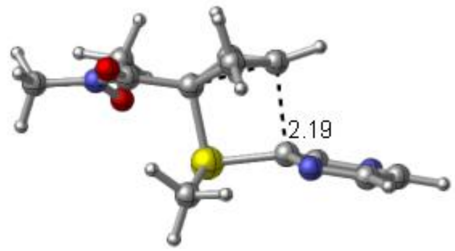

Zero-point correction= 0.279453 (Hartree/Particle)

Thermal correction to Energy= 0.297516

Thermal correction to Enthalpy= 0.298461

Thermal correction to Gibbs Free Energy= 0.232913

Sum of electronic and zero-point Energies= -1179.117465

Sum of electronic and thermal Energies= -1179.099402

Sum of electronic and thermal Enthalpies= -1179.098458

Sum of electronic and thermal Free Energies= -1179.164006

UB3LYP-D3/def2TZVPP

E(scf)= -1180.411024

UM062X-D3/def2TZVPP

E(scf) = -1180.00882986

UB3LYP-D3/def2TZVPP (gas)

E(scf)= -1180.340177

TS2'

E(scf) = -1179.40971503 a.u.

ν_{min} = -508.6327 cm^{-1}

C	0.378628	0.417282	-2.514211
C	0.635938	0.522514	-0.992821
C	-0.782668	1.136024	-0.856129
C	-1.087517	0.381876	-2.140309
C	-2.200077	0.339179	-3.119196
N	-3.453602	0.654701	-2.674125
O	-1.979095	-0.025393	-4.272694
C	-4.667882	0.352089	-3.405841
O	-3.617660	0.882535	-1.317064
C	-3.926111	2.253669	-1.035256
H	0.637040	1.376163	-2.994787
H	0.801672	-0.403994	-3.106087
H	-1.377263	0.953730	0.049034
H	-0.732701	2.222985	-1.031708
H	-5.336513	1.225100	-3.429019
H	-4.385035	0.085706	-4.430567
H	-5.185734	-0.495154	-2.928076
H	-3.988238	2.317987	0.059009
H	-3.133248	2.922198	-1.407206
H	-4.893824	2.544581	-1.474390
C	-1.078788	-1.564134	-1.390130
C	-2.351411	-1.721298	-0.722161
N	-3.330478	-2.374957	-1.310983
C	-3.071939	-2.968214	-2.492258
C	-1.770135	-2.977786	-3.039184
N	-0.770887	-2.340342	-2.456094
H	-2.542100	-1.247295	0.244847
H	-3.888466	-3.503919	-2.983698
H	-1.548341	-3.561806	-3.937929
S	0.384819	-1.161088	-0.269452
C	1.715320	-2.186088	-0.939726
H	1.716963	-2.145254	-2.033939
H	2.644122	-1.793444	-0.504361
H	1.528210	-3.208078	-0.585795
H	1.528257	0.986219	-0.555866

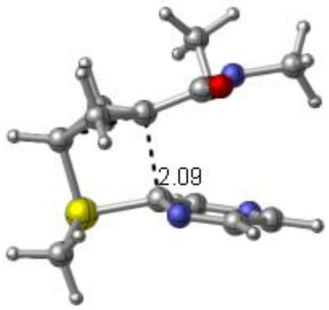

Zero-point correction= 0.280193 (Hartree/Particle)

Thermal correction to Energy= 0.298095

Thermal correction to Enthalpy= 0.299039

Thermal correction to Gibbs Free Energy= 0.234368

Sum of electronic and zero-point Energies= -1179.129522

Sum of electronic and thermal Energies= -1179.111620

Sum of electronic and thermal Enthalpies= -1179.110676

Sum of electronic and thermal Free Energies= -1179.175347

UB3LYP-D3/def2TZVPP

E(scf)= -1180.423674

UM062X-D3/def2TZVPP

E(scf)= -1180.023636

UB3LYP-D3/def2TZVPP (gas)

E(scf)= -1180.348843

TS6 (EWG=C(O)NMeOMe)

E(scf) = -1410.31921403 a.u.

ν_{min} = -463.0868 cm^{-1}

C	0.756541	-0.702302	-1.359061	H	2.036110	1.357106	0.654842
C	1.992234	-0.623870	-0.469853	H	2.188425	1.440009	-1.126631
C	1.758716	0.869315	-0.287868	H	-2.430854	3.253890	-1.965862
C	0.278187	0.561742	-0.595300	H	-3.113427	2.110039	-3.161292
C	-0.611131	1.680907	-1.120269	H	-3.676510	2.060689	-1.454081
N	-1.744152	1.312380	-1.771215	H	-2.320464	-1.700661	-2.827992
O	-0.293401	2.845517	-0.913899	H	-2.971550	-0.225463	-3.614183
C	-2.810843	2.236326	-2.111909	H	-1.191171	-0.499163	-3.554157
O	-2.090113	-0.029114	-1.715850	C	3.244798	-1.344841	-0.567371
C	-2.142070	-0.633851	-3.015375	C	3.299852	-2.609585	-1.193702
H	1.008823	-0.437137	-2.398189	C	4.419589	-0.811516	0.008705
H	0.122573	-1.598370	-1.367354	C	4.498727	-3.317459	-1.243653

H	2.397163	-3.032302	-1.640503
C	5.616338	-1.524951	-0.045303
H	4.386097	0.164769	0.496621
C	5.657915	-2.778964	-0.667576
H	4.533534	-4.293868	-1.732172
H	6.520484	-1.104232	0.400445
H	6.596068	-3.337669	-0.707088
S	-0.439598	-0.167412	0.964960
C	-0.319699	1.194174	2.160483
H	0.647917	1.109134	2.674779
H	-0.398427	2.132332	1.593834
H	-1.159567	1.078224	2.855858
C	1.125435	-1.199551	1.296355
C	0.921403	-2.630090	1.199090
N	1.939732	-0.705073	2.259259
N	1.772576	-3.458042	1.760867
H	0.095920	-3.043633	0.609922
C	2.793710	-1.550167	2.808866
C	2.756746	-2.932357	2.519804
H	3.502229	-1.154356	3.543567
H	3.478994	-3.618594	2.969200

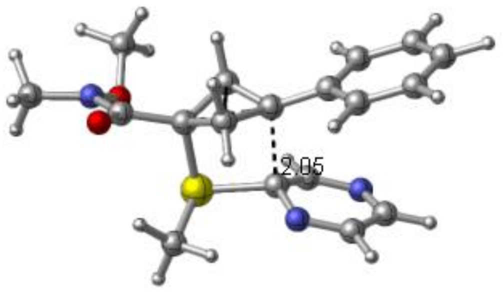

Zero-point correction= 0.361157 (Hartree/Particle)

Thermal correction to Energy= 0.383796

Thermal correction to Enthalpy= 0.384740

Thermal correction to Gibbs Free Energy= 0.308970

Sum of electronic and zero-point Energies= -1409.958057

Sum of electronic and thermal Energies= -1409.935418

Sum of electronic and thermal Enthalpies= -1409.934474

Sum of electronic and thermal Free Energies= -1410.010244

UB3LYP-D3/def2TZVPP

E(scf)= -1411.578198

UM062X-D3/def2TZVPP

E(scf)= -1411.072585

UB3LYP-D3/def2TZVPP (gas)

E(scf)= -1411.510591

TS6' (EWG=C(O)NMeOMe)

E(scf) = -1410.31587404 a.u.

ν_{min} = -512.4774 cm^{-1}

C	0.347220	-1.096737	-2.333003	C	3.530340	0.854541	0.203729
C	0.124396	-0.213477	-1.079504	H	2.796809	-0.743520	-1.050419
C	-1.082650	0.425292	-1.824507	C	1.866244	2.414154	1.026313
C	-1.118583	-0.866254	-2.621850	H	-0.173066	2.032602	0.423719
C	-1.809559	-1.339517	-3.844442	C	3.198981	1.990708	0.948922
N	-3.019079	-0.779847	-4.150619	H	4.569517	0.524480	0.137863
O	-1.311217	-2.244631	-4.511354	H	1.606122	3.303500	1.604841
C	-3.952460	-1.352538	-5.100856	H	3.980470	2.550046	1.468756
O	-3.570448	0.101873	-3.234115	S	-0.816203	-1.319615	0.151542
C	-3.620134	1.442354	-3.739117	C	-1.845137	-2.172247	-1.168432
H	0.977882	-0.543552	-3.049065	C	-3.267828	-1.919833	-1.109619
H	0.723868	-2.123288	-2.245586	N	-1.387020	-3.388739	-1.549619
H	-1.966069	0.756838	-1.263108	N	-4.109916	-2.736607	-1.706449
H	-0.720419	1.265332	-2.439879	H	-3.659417	-1.018336	-0.630079
H	-3.415377	-2.107256	-5.686471	C	-2.245186	-4.187341	-2.157678
H	-4.788534	-1.823878	-4.559466	C	-3.611436	-3.850565	-2.276493
H	-4.343117	-0.578388	-5.777322	H	-1.875235	-5.149709	-2.525089
H	-4.026033	2.046658	-2.917221	H	-4.311160	-4.524152	-2.778292
H	-2.614075	1.801471	-4.009008	C	0.384735	-2.576610	0.644035
H	-4.288039	1.516809	-4.612200	H	1.251779	-2.024583	1.032743
C	1.194446	0.559564	-0.387945	H	-0.083214	-3.167396	1.441628
C	2.531867	0.138661	-0.463165	H	0.641352	-3.207223	-0.214640
C	0.866604	1.701669	0.362261				

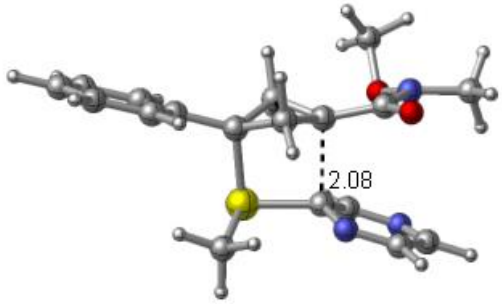

Zero-point correction= 0.360695 (Hartree/Particle)

Thermal correction to Energy= 0.383513

Thermal correction to Enthalpy= 0.384457

Thermal correction to Gibbs Free Energy= 0.308245

Sum of electronic and zero-point Energies= -1409.955179

Sum of electronic and thermal Energies= -1409.932361

Sum of electronic and thermal Enthalpies= -1409.931417

Sum of electronic and thermal Free Energies= -1410.007629

UB3LYP-D3/def2TZVPP

E(scf)= -1411.574883

UM062X-D3/def2TZVPP

E(scf)= -1411.068946

UB3LYP-D3/def2TZVPP (gas)

E(scf)= -1411.505997

I(EWG=C(O)NMeOMe)

E(scf) = -1410.34304988 a.u.

ν_{min} = 34.7453 cm^{-1}

C	0.868160	-0.578753	-1.515088	O	-0.311448	2.850209	-0.934483
C	2.255911	-0.309436	-0.994846	C	-2.838563	2.116249	-2.021899
C	1.859546	1.011599	-0.386461	O	-2.006409	-0.114352	-1.625115
C	0.380042	0.583631	-0.596370	C	-2.180615	-0.718806	-2.913926
C	-0.583189	1.668878	-1.101768	H	0.727405	-0.304630	-2.574593
N	-1.724162	1.240855	-1.702753	H	0.426630	-1.576857	-1.371239

H	2.200600	1.230526	0.636192
H	2.073961	1.896711	-1.009691
H	-2.513805	3.147895	-1.845136
H	-3.133700	2.005783	-3.075361
H	-3.692532	1.872886	-1.370812
H	-2.238025	-1.797161	-2.717451
H	-3.116432	-0.383967	-3.388710
H	-1.327466	-0.501234	-3.575253
C	3.379599	-1.139831	-0.817157
C	3.392309	-2.478262	-1.313372
C	4.519418	-0.687509	-0.085654
C	4.473327	-3.315945	-1.066906
H	2.533481	-2.848344	-1.878068
C	5.594354	-1.536149	0.149323
H	4.535894	0.333772	0.301658
C	5.578749	-2.856158	-0.331623
H	4.459653	-4.341458	-1.444396
H	6.454395	-1.174586	0.718531
H	6.423646	-3.520899	-0.137727
S	-0.421697	-0.104522	0.963723
C	-0.245009	1.283229	2.122174
H	0.785387	1.302938	2.497398
H	-0.492875	2.193997	1.561350
H	-0.965254	1.107863	2.930928
C	0.844933	-1.276284	1.552107
C	0.682505	-2.629926	1.234806
N	1.866688	-0.806570	2.241989
N	1.626158	-3.505492	1.586123
H	-0.200799	-2.999034	0.704354
C	2.801991	-1.682988	2.600771
C	2.682994	-3.040101	2.251098
H	3.660382	-1.314548	3.167886
H	3.467721	-3.751261	2.525196

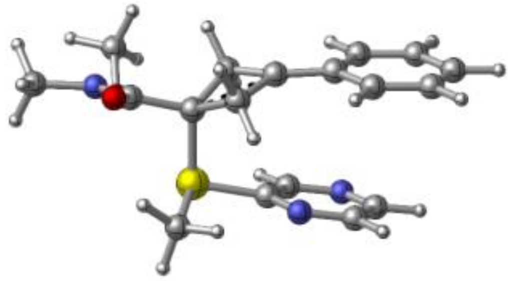

Zero-point correction= 0.361665 (Hartree/Particle)

Thermal correction to Energy= 0.385125

Thermal correction to Enthalpy= 0.386070

Thermal correction to Gibbs Free Energy= 0.308112

Sum of electronic and zero-point Energies= -1409.981385

Sum of electronic and thermal Energies= -1409.957924

Sum of electronic and thermal Enthalpies= -1409.956980

Sum of electronic and thermal Free Energies= -1410.034938

UB3LYP-D3/def2TZVPP

E(scf)= -1411.6035

UM062X-D3/def2TZVPP

E(scf)= -1411.096196

UB3LYP-D3/def2TZVPP (gas)

E(scf)= -1411.535573

I'(EWG=C(O)NMeOMe)

E(scf) = -1410.33884561 a.u.

ν_{min} = 25.4778 cm^{-1}

C	0.326162	-1.032168	-2.452727	H	-4.680778	-0.651142	-5.543698
C	0.044991	-0.257491	-1.129871	H	-3.776210	2.447917	-3.413425
C	-1.122518	0.490774	-1.847627	H	-2.366830	1.899834	-4.391358
C	-0.986626	-0.512501	-2.960377	H	-4.044002	1.672492	-5.012238
C	-1.861431	-1.191173	-3.904337	C	1.114306	0.500284	-0.403975
N	-3.085744	-0.626492	-4.181185	C	2.459979	0.107709	-0.493122
O	-1.510030	-2.254823	-4.430606	C	0.766563	1.601989	0.397260
C	-4.157968	-1.329854	-4.853759	C	3.444359	0.813239	0.204297
O	-3.500863	0.442204	-3.399457	H	2.744138	-0.747703	-1.109850
C	-3.410427	1.681567	-4.110058	C	1.751989	2.303666	1.093164
H	1.199989	-0.593678	-2.965857	H	-0.279036	1.910071	0.475066
H	0.459104	-2.123652	-2.424635	C	3.092305	1.910116	0.997639
H	-2.094668	0.583115	-1.343670	H	4.489221	0.504757	0.125867
H	-0.798781	1.508523	-2.127591	H	1.474102	3.160942	1.710436
H	-3.717615	-2.157694	-5.421066	H	3.863318	2.460913	1.541574
H	-4.873615	-1.727770	-4.115123	S	-0.772670	-1.389461	0.193496

C	-1.855949	-2.468641	-0.791001	H	-1.704809	-5.200446	-2.543360
C	-3.197790	-2.102525	-0.959051	H	-4.108340	-4.532816	-2.945319
N	-1.325947	-3.553585	-1.328830	C	0.560898	-2.521070	0.656281
N	-3.991256	-2.843915	-1.734506	H	1.339044	-1.902203	1.119144
H	-3.623995	-1.216549	-0.478761	H	0.134757	-3.218651	1.388881
C	-2.124818	-4.300457	-2.086723	H	0.922744	-3.053017	-0.230823
C	-3.463230	-3.929924	-2.299378				

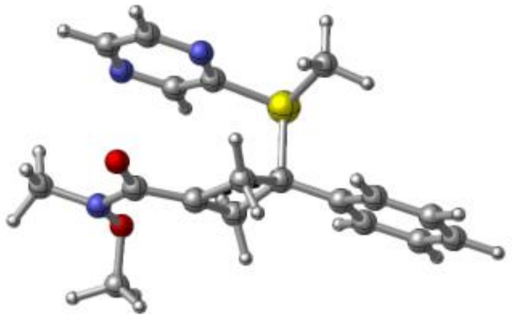

Zero-point correction= 0.361727 (Hartree/Particle)

Thermal correction to Energy= 0.385201

Thermal correction to Enthalpy= 0.386146

Thermal correction to Gibbs Free Energy= 0.307763

Sum of electronic and zero-point Energies= -1409.977119

Sum of electronic and thermal Energies= -1409.953644

Sum of electronic and thermal Enthalpies= -1409.952700

Sum of electronic and thermal Free Energies= -1410.031082

UB3LYP-D3/def2TZVPP

E(scf)= -1411.599427

UM062X-D3/def2TZVPP

E(scf)= -1411.092502

UB3LYP-D3/def2TZVPP (gas)

E(scf)= -1411.528745

[2ag]$^{+\cdot}$ (EWG=C(O)NMeOMe)

E(scf) = -708.801008147 a.u.

ν_{min} = 25.9282 cm^{-1}

C	-2.206138	-0.286263	0.389787	H	2.823848	1.066262	-1.794803
C	-2.016826	-0.982195	-0.930039	H	2.695252	-2.210432	1.090229
C	-0.848649	-1.837089	-0.517055	H	3.197166	-0.511436	1.393348
C	-0.760951	-0.472241	0.078536	H	1.541042	-1.060104	1.856867
C	0.122995	0.684144	-0.284908	C	-2.369010	-0.526014	-2.235422
N	1.454305	0.418673	-0.345060	C	-3.265194	0.568924	-2.402331
O	-0.369628	1.785412	-0.494586	C	-1.849873	-1.181513	-3.389381
C	2.449527	1.367009	-0.803292	C	-3.610449	0.996771	-3.674000
O	1.884336	-0.885994	-0.206949	H	-3.680456	1.069972	-1.527623
C	2.355085	-1.167314	1.121329	C	-2.205253	-0.745973	-4.655909
H	-2.593069	0.735618	0.409719	H	-1.175286	-2.030663	-3.273985
H	-2.590023	-0.938227	1.189798	C	-3.081771	0.343969	-4.802158
H	-0.097141	-2.129115	-1.254010	H	-4.292630	1.839248	-3.800962
H	-1.100371	-2.635967	0.198810	H	-1.805760	-1.247783	-5.538993
H	1.975300	2.353001	-0.868971	H	-3.356752	0.685879	-5.802627
H	3.288303	1.407763	-0.093280				

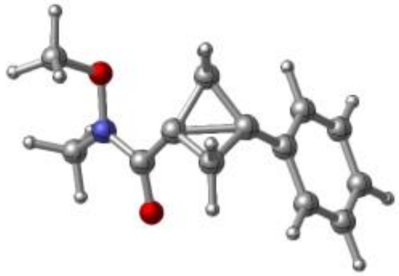

Zero-point correction= 0.253500 (Hartree/Particle)

Thermal correction to Energy= 0.268984

Thermal correction to Enthalpy= 0.269928

Thermal correction to Gibbs Free Energy= 0.208655

Sum of electronic and zero-point Energies= -708.547508

Sum of electronic and thermal Energies= -708.532024

Sum of electronic and thermal Enthalpies= -708.531080

Sum of electronic and thermal Free Energies= -708.592353

UB3LYP-D3/def2TZVPP

E(scf)= -709.5872532

UM062X-D3/def2TZVPP

E(scf)= -709.2516482

UB3LYP-D3/def2TZVPP (gas)

E(scf)= -709.5201381

2ag (EWG=C(O)NMeOMe)

E(scf) = -709.015401625 a.u.

ν_{min} = 19.5333 cm^{-1}

C	-2.339308	-0.287617	0.530327	H	2.445046	1.278698	-1.986570
C	-1.999727	-0.946197	-0.766414	H	2.739371	-2.275741	0.560560
C	-0.943431	-1.888559	-0.270024	H	3.150081	-0.605353	1.082418
C	-0.888652	-0.452059	0.167255	H	1.558534	-1.326112	1.530594
C	-0.018849	0.700600	-0.164665	C	-2.289786	-0.517588	-2.144479
N	1.309160	0.466074	-0.410568	C	-3.098175	0.607504	-2.398300
O	-0.477052	1.842676	-0.202470	C	-1.747677	-1.214371	-3.241794
C	2.225960	1.462698	-0.921265	C	-3.337286	1.033390	-3.706835
O	1.782198	-0.835564	-0.493390	H	-3.546926	1.151962	-1.565659
C	2.336067	-1.271444	0.750358	C	-1.989207	-0.786569	-4.549713
H	-2.727379	0.733516	0.488349	H	-1.137826	-2.103733	-3.070536
H	-2.734302	-0.903185	1.351193	C	-2.781973	0.341402	-4.789360
H	-0.184959	-2.214177	-0.983980	H	-3.965744	1.910458	-3.882106
H	-1.220787	-2.644140	0.479651	H	-1.558807	-1.341862	-5.387158
H	1.758414	2.447271	-0.808112	H	-2.973318	0.673659	-5.812716
H	3.168273	1.438690	-0.352516				

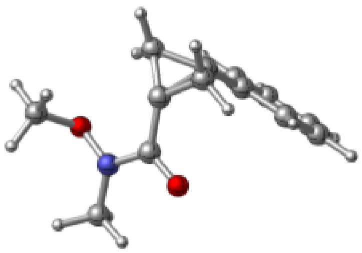

Zero-point correction= 0.254559 (Hartree/Particle)

Thermal correction to Energy= 0.269779

Thermal correction to Enthalpy= 0.270723

Thermal correction to Gibbs Free Energy= 0.210051

Sum of electronic and zero-point Energies= -708.760843

Sum of electronic and thermal Energies= -708.745623

Sum of electronic and thermal Enthalpies= -708.744678

Sum of electronic and thermal Free Energies= -708.805350

UB3LYP-D3/def2TZVPP

E(scf)= -709.801659

UM062X-D3/def2TZVPP

E(scf)= -709.4814739

UB3LYP-D3/def2TZVPP (gas)

E(scf) = -709.791370197

TS4

E(scf) = -1637.65235656 a.u.

ν_{min} = -483.8834 cm^{-1}

C	-0.432188	0.147728	0.852780	H	-3.920322	-1.277397	-0.105714
C	-0.573413	-1.204112	0.398115	H	-3.704013	1.070080	0.676904
C	-1.823611	-1.728354	0.055251	H	-1.461782	1.996210	1.310041
C	-2.933869	-0.889710	0.157901	N	0.833614	0.520301	1.180568
C	-2.809803	0.447941	0.600736	S	0.986091	-1.994727	0.371374
C	-1.574779	0.973883	0.947402	S	3.457023	-0.212998	0.961045
H	-1.929508	-2.760785	-0.281398	C	1.650124	-0.520662	1.175403

C	3.532363	1.574354	0.683638
H	2.893398	2.099805	1.400514
H	4.591207	1.846525	0.787588
H	3.189879	1.741419	-0.345487
C	3.211257	-1.840196	3.055747
C	3.856124	-0.458746	2.751562
C	2.726134	0.187256	3.590963
C	1.934464	-1.061036	3.264896
C	0.635342	-1.655128	3.638469
N	-0.375323	-0.811266	4.011840
O	0.475731	-2.872691	3.557429
C	-1.766022	-1.208685	4.095253
O	-0.141128	0.552635	3.987141
C	-0.030399	1.099449	5.306512
H	3.245197	-2.648129	2.309081
H	3.606872	-2.230275	4.008960
H	4.921523	-0.256516	2.913765
H	2.313344	1.166719	3.322584
H	3.038155	0.206477	4.649305
H	-1.805480	-2.302599	4.045296
H	-2.211726	-0.872737	5.043270
H	-2.328817	-0.775980	3.252138
H	0.184152	2.166519	5.161321
H	-0.971574	0.989261	5.869341
H	0.792792	0.624223	5.864392

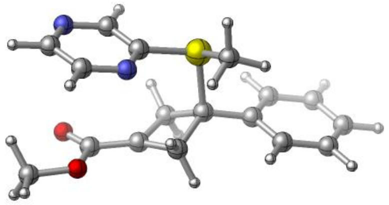

Zero-point correction= 0.305931 (Hartree/Particle)

Thermal correction to Energy= 0.326360

Thermal correction to Enthalpy= 0.327304

Thermal correction to Gibbs Free Energy= 0.256600

Sum of electronic and zero-point Energies= -1637.346426

Sum of electronic and thermal Energies= -1637.325997

Sum of electronic and thermal Enthalpies= -1637.325053

Sum of electronic and thermal Free Energies= -1637.395757

UB3LYP-D3/def2TZVPP

E(scf)= -1638.86587823

UM062X-D3/def2TZVPP

E(scf)= -1638.40483840

UB3LYP-D3/def2TZVPP (gas)

E(scf)= -1638.79480820

TS3'–α

E(scf) = -1637.64273264 a.u.

ν_{min} = -312.0797 cm^{-1}

C	-1.930871	1.128873	0.608006	S	-0.307712	-0.444471	2.638048
C	-2.226686	0.352290	-0.632320	C	0.279558	1.176013	3.215839
C	-1.643634	-0.940553	-0.159649	H	1.067773	1.545508	2.550046
C	-0.791321	0.106250	0.556471	H	-0.597949	1.834815	3.198348
C	0.526051	0.652566	0.008486	H	0.644995	1.048033	4.242544
N	1.429380	-0.228689	-0.497892	H	-2.182258	0.738060	-1.654096
O	0.747088	1.856873	0.073979	C	3.340214	-1.774797	2.315068
C	2.791268	0.150717	-0.836687	C	2.865300	-3.116249	2.343982
O	1.214185	-1.577629	-0.273534	C	3.737475	-4.203141	2.205556
C	1.279397	-2.363527	-1.468664	H	3.369385	-5.230215	2.228199
H	-1.660493	2.187269	0.527180	C	4.715191	-1.524147	2.144823
H	-2.635922	0.940665	1.436365	H	5.074107	-0.493650	2.124571
H	-2.284939	-1.499901	0.543772	C	5.093111	-3.929748	2.032562
H	-1.179799	-1.612535	-0.884787	H	5.793635	-4.759931	1.917286
H	2.884348	1.233168	-0.698648	C	5.577754	-2.604219	2.002510
H	3.488005	-0.375294	-0.167725	H	6.646715	-2.427801	1.864975
H	3.019656	-0.102077	-1.882266	C	1.199748	-1.360694	2.567710
H	0.955702	-3.366734	-1.163091	N	2.362752	-0.811604	2.441272
H	0.605960	-1.966676	-2.245508	S	1.131988	-3.119680	2.575946
H	2.308248	-2.415765	-1.857725				

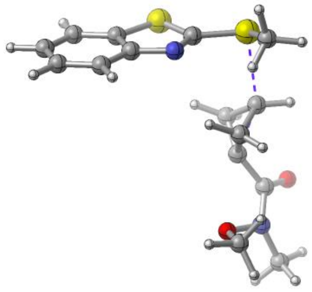

Zero-point correction= 0.304719 (Hartree/Particle)

Thermal correction to Energy= 0.325854

Thermal correction to Enthalpy= 0.326798

Thermal correction to Gibbs Free Energy= 0.253839

Sum of electronic and zero-point Energies= -1637.338014

Sum of electronic and thermal Energies= -1637.316879

Sum of electronic and thermal Enthalpies= -1637.315935

Sum of electronic and thermal Free Energies= -1637.388894

UB3LYP-D3/def2TZVPP

E(scf) = -1638.85562174

UM062X-D3/def2TZVPP

E(scf)= -1638.393712

UB3LYP-D3/def2TZVPP (gas)

E(scf) = -1638.790814

E

E(scf) = -1637.65429935 a.u.

ν_{min} = 27.4890 cm^{-1}

C	-2.117951	-0.309559	0.718552	N	1.597086	-0.055347	-0.280554
C	-1.982724	-0.676918	-0.713572	O	0.146465	1.584883	0.301076
C	-1.078837	-1.862356	-0.587711	C	2.743093	0.817941	-0.439112
C	-0.746794	-0.602034	0.154881	O	1.815110	-1.400461	-0.243203
C	0.326673	0.394283	0.060841	C	2.493603	-1.954698	-1.380620

H	-2.333308	0.738258	0.946852	H	-2.186350	-0.067077	-1.593679
H	-2.530724	-1.039873	1.429836	C	2.891756	-1.617973	2.284984
H	-1.407453	-2.732010	0.000261	C	1.992326	-2.740210	2.383851
H	-0.464770	-2.109928	-1.457978	C	2.397857	-4.026211	2.030612
H	2.401117	1.842995	-0.264230	H	1.717630	-4.875878	2.100180
H	3.515404	0.549171	0.296514	C	4.221172	-1.823304	1.830567
H	3.156844	0.742981	-1.455919	H	4.902766	-0.973607	1.772458
H	2.459661	-3.038306	-1.217064	C	3.708367	-4.191631	1.576964
H	1.961567	-1.691477	-2.307991	H	4.048127	-5.189402	1.290957
H	3.540852	-1.622002	-1.426933	C	4.611788	-3.100466	1.479672
S	0.086964	0.751823	3.481762	H	5.627525	-3.281054	1.123345
C	1.206084	2.177603	3.328718	C	1.105663	-0.546322	3.004313
H	1.403951	2.353793	2.264531	N	2.362846	-0.421825	2.634059
H	0.655507	3.020996	3.765070	S	0.444138	-2.198072	2.966272
H	2.131371	1.982650	3.884390				

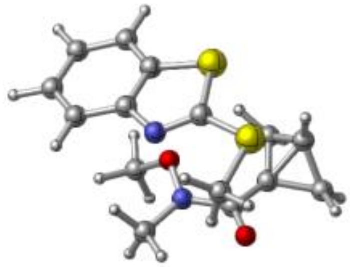

Zero-point correction= 0.304704 (Hartree/Particle)

Thermal correction to Energy= 0.326986

Thermal correction to Enthalpy= 0.327930

Thermal correction to Gibbs Free Energy= 0.251517

Sum of electronic and zero-point Energies= -1637.349596

Sum of electronic and thermal Energies= -1637.327313

Sum of electronic and thermal Enthalpies= -1637.326369

Sum of electronic and thermal Free Energies= -1637.402782

UB3LYP-D3/def2TZVPP

E(scf)= -1638.86514438

UM062X-D3/def2TZVPP

E(scf)= -1638.40443837

UB3LYP-D3/def2TZVPP(gas)

E(scf)= -1638.80227458

F'

E(scf) = -1637.65092109 a.u.

ν_{min} = 32.6359 cm^{-1}

C	-1.900926	0.994909	0.514124	H	1.275364	1.550131	2.604940
C	-2.623836	-0.256007	0.111692	H	-0.429884	2.074188	2.942642
C	-1.365742	-1.055545	-0.047712	H	0.477619	1.125691	4.183287
C	-0.597474	0.132543	0.611126	H	-3.665739	-0.457854	-0.136961
C	0.670533	0.765003	0.031812	C	3.227346	-1.828433	2.299318
N	1.591767	-0.053847	-0.543297	C	2.665434	-3.135765	2.312460
O	0.819695	1.977739	0.117803	C	3.470487	-4.278397	2.221424
C	2.933838	0.389377	-0.881526	H	3.036559	-5.279298	2.233147
O	1.437111	-1.418646	-0.356686	C	4.621314	-1.665062	2.191814
C	1.468981	-2.159593	-1.581284	H	5.045881	-0.659753	2.183543
H	-1.785491	1.760014	-0.276195	C	4.846331	-4.091425	2.110665
H	-2.238338	1.528593	1.420250	H	5.497231	-4.965159	2.033318
H	-1.278942	-2.044232	0.434807	C	5.416738	-2.799456	2.095391
H	-1.046403	-1.186535	-1.096617	H	6.500020	-2.694131	2.006456
H	2.957701	1.481220	-0.796741	C	1.112896	-1.285901	2.452016
H	3.652048	-0.058590	-0.177542	N	2.306347	-0.806713	2.378912
H	3.192670	0.099880	-1.909818	S	0.926155	-3.028087	2.462345
H	1.173068	-3.178887	-1.301802	H	-1.704809	-5.200446	-2.543360
H	0.759942	-1.747069	-2.316609	H	-4.108340	-4.532816	-2.945319
H	2.483287	-2.178931	-2.009952	C	0.560898	-2.521070	0.656281
S	-0.335465	-0.239825	2.435355	H	1.339044	-1.902203	1.119144
C	0.335182	1.306824	3.110447	H	0.134757	-3.218651	1.388881

H	0.922744	-3.053017	-0.230823

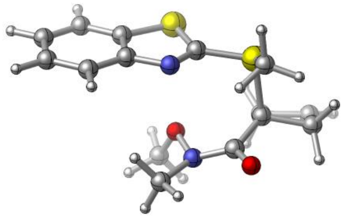

Zero-point correction= 0.305060 (Hartree/Particle)

Thermal correction to Energy= 0.326569

Thermal correction to Enthalpy= 0.327513

Thermal correction to Gibbs Free Energy= 0.254450

Sum of electronic and zero-point Energies= -1637.345862

Sum of electronic and thermal Energies= -1637.324352

Sum of electronic and thermal Enthalpies= -1637.323408

Sum of electronic and thermal Free Energies= -1637.396471

UB3LYP-D3/def2TZVPP

E(scf)= -1638.86478052

UM062X-D3/def2TZVPP

E(scf)= -1638.40577489

UB3LYP-D3/def2TZVPP (gas)

E(scf)= -1638.80016028

F

E(scf) -1637.67096245 a.u.

ν_{min} = 21.5118 cm^{-1}

C	0.154462	0.206218	2.245679	H	-2.292275	-1.439064	4.050853
C	-0.639963	-0.857454	2.757669	H	-2.738874	0.912587	4.732878
C	-1.687952	-0.620728	3.657008	H	-1.362466	2.783145	3.856578
C	-1.929407	0.698601	4.031588	H	0.517473	2.338979	2.257613
C	-1.146543	1.763245	3.531816	N	1.154010	-0.189636	1.383445
C	-0.105194	1.530855	2.643478	S	-0.092758	-2.378214	2.088806

S	2.368658	-2.330792	0.243165	O	2.960195	0.728779	4.167227
C	1.142963	-1.470656	1.234691	C	4.178920	1.272655	4.681871
C	2.864970	-1.031292	-0.921354	H	5.119742	-0.965828	2.418000
H	3.074746	-0.107711	-0.370226	H	3.587082	-0.186321	1.941948
H	3.751152	-1.417238	-1.441464	H	4.630629	-2.788799	0.776382
H	2.029559	-0.900906	-1.620451	H	4.293406	-3.826082	3.019769
C	4.039017	-1.133370	2.263665	H	2.539557	-3.822755	2.712536
C	3.814475	-2.409898	1.407056	H	1.176841	-0.676503	6.565279
C	3.444795	-3.195245	2.700587	H	1.893163	0.965640	6.586987
C	3.371024	-1.868089	3.397489	H	0.577329	0.575697	5.428755
C	2.539520	-1.572523	4.553524	H	4.475537	2.056057	3.971325
N	2.458959	-0.268953	4.992283	H	4.030067	1.719350	5.678929
O	1.928316	-2.480522	5.128374	H	4.964501	0.500770	4.735934
C	1.466500	0.180168	5.946410				

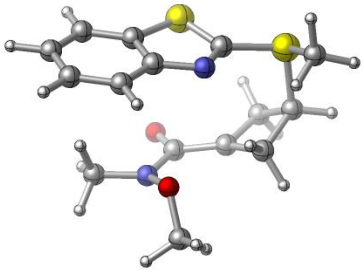

Zero-point correction= 0.306588 (Hartree/Particle)

Thermal correction to Energy= 0.327881

Thermal correction to Enthalpy= 0.328825

Thermal correction to Gibbs Free Energy= 0.255042

Sum of electronic and zero-point Energies= -1637.364374

Sum of electronic and thermal Energies= -1637.343081

Sum of electronic and thermal Enthalpies= -1637.342137

Sum of electronic and thermal Free Energies= -1637.415921

UB3LYP-D3/def2TZVPP

E(scf)= -1638.887427

UM062X-D3/def2TZVPP

E(scf)= -1638.426551

UB3LYP-D3/def2TZVPP (gas)

E(scf)= -1638.81548

1b

E(scf) = -1159.74554035a.u.

ν_{min} = 62.9094-cm^{-1}

C	-0.689249	-0.157430	0.433718	H	-2.186257	1.288903	1.013092
C	-0.458791	-1.475470	-0.042883	N	0.450306	0.599391	0.649781
C	-1.513289	-2.355881	-0.306478	S	1.268994	-1.736745	-0.213289
C	-2.816725	-1.903856	-0.087370	S	3.167110	0.526146	0.501614
C	-3.060978	-0.600375	0.384698	C	1.521377	-0.069023	0.365053
C	-2.008838	0.275708	0.646760	C	2.844437	2.211902	1.113419
H	-1.325231	-3.367085	-0.672692	H	2.316327	2.173750	2.075191
H	-3.656479	-2.573931	-0.286235	H	3.831934	2.676206	1.239026
H	-4.090089	-0.271411	0.547720	H	2.248893	2.775379	0.383196

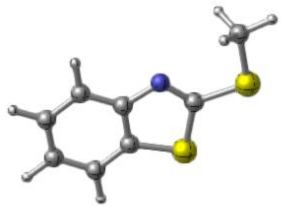

Zero-point correction= 0.130999 (Hartree/Particle)

Thermal correction to Energy= 0.140743

Thermal correction to Enthalpy= 0.141688

Thermal correction to Gibbs Free Energy= 0.095029

Sum of electronic and zero-point Energies= -1159.614541

Sum of electronic and thermal Energies= -1159.604797

Sum of electronic and thermal Enthalpies= -1159.603853

Sum of electronic and thermal Free Energies= -1159.650511

UB3LYP-D3/def2TZVPP

E(scf)= -1160.42394632

UM062X-D3/def2TZVPP

E(scf)= -1160.19234564

UB3LYP-D3/def2TZVPP (gas)

E(scf)= -1160.41894088

G

E(scf) = -1637.71038451 a.u.

ν_{min} = 24.7428 cm^{-1}

C	-0.407373	-0.042236	0.243876	C	2.774393	-1.737372	2.492437
C	-1.417101	-0.997832	0.517822	C	3.327963	-0.296842	2.364538
C	-2.529383	-1.140298	-0.319025	C	2.319029	0.151756	3.447996
C	-2.604822	-0.320863	-1.445299	C	1.498187	-1.148469	3.182921
C	-1.599773	0.625287	-1.729244	C	0.998731	-1.950868	4.390185
C	-0.498166	0.776867	-0.891694	N	0.077401	-1.322954	5.176484
H	-3.311255	-1.868958	-0.098709	O	1.409349	-3.077805	4.624769
H	-3.460136	-0.415558	-2.117873	C	-0.713961	-1.996834	6.188591
H	-1.688242	1.251696	-2.619419	O	-0.419406	-0.107872	4.725478
H	0.282481	1.509325	-1.103171	C	-0.121388	0.969405	5.622030
N	0.594701	-0.028344	1.197524	H	2.625045	-2.330126	1.579665
S	-1.024505	-1.863562	1.993835	H	3.361614	-2.321583	3.212178
S	3.032452	0.383450	0.692800	H	4.398346	-0.137807	2.565443
C	0.433775	-0.907405	2.146186	H	1.793610	1.106304	3.329999
C	3.035747	2.165790	0.976534	H	2.803128	0.119993	4.432933
H	2.132570	2.453516	1.531070	H	-0.284480	-2.994137	6.337797
H	3.940145	2.433477	1.542398	H	-0.684667	-1.445831	7.139978
H	3.043433	2.644306	-0.011435	H	-1.757454	-2.084620	5.846323

H	-0.450752	1.877217	5.099724	H	0.957927	1.027677	5.832224
H	-0.678936	0.870165	6.567255				

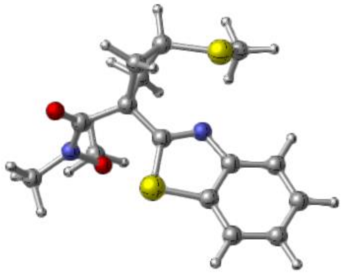

Zero-point correction= 0.309094 (Hartree/Particle)

Thermal correction to Energy= 0.329925

Thermal correction to Enthalpy= 0.330869

Thermal correction to Gibbs Free Energy= 0.258206

Sum of electronic and zero-point Energies= -1637.401291

Sum of electronic and thermal Energies= -1637.380459

Sum of electronic and thermal Enthalpies= -1637.379515

Sum of electronic and thermal Free Energies= -1637.452178

UB3LYP-D3/def2TZVPP

E(scf)= -1638.919943

UM062X-D3/def2TZVPP

E(scf)= -1638.459352

UB3LYP-D3/def2TZVPP (gas)

E(scf)= -1638.851364

G’

E(scf) = -1637.71189225 a.u.

ν_{min} = 28.1068 cm^{-1}

C	-1.376370	-0.149766	1.026534	C	-3.931017	-0.091668	-0.082432
C	-2.435985	-0.893474	1.605158	C	-2.880286	0.645118	-0.665863
C	-3.724396	-0.865468	1.059088	C	-1.599860	0.627495	-0.120460

H	-4.539930	-1.432234	1.511639	N	5.025690	-1.263659	1.874238
H	-4.926280	-0.056742	-0.530951	O	4.652284	0.421564	3.353528
H	-3.075205	1.241011	-1.560119	C	6.465478	-1.342221	2.044657
H	-0.781440	1.196122	-0.564864	O	4.429215	-2.314438	1.197941
N	-0.189271	-0.287884	1.719713	C	4.614019	-2.232568	-0.221900
S	-1.846953	-1.756413	3.017012	H	1.439281	0.784882	3.729151
S	2.174445	0.077142	0.921613	H	2.512234	-0.313349	4.642262
C	-0.255714	-1.090444	2.744597	H	0.667979	-1.985187	4.469671
C	2.297606	1.841175	1.296206	H	1.754398	-2.627051	1.771457
H	1.379241	2.168299	1.802373	H	2.719097	-2.742098	3.271361
H	3.174519	2.003615	1.940492	H	6.766365	-0.538773	2.726450
H	2.409139	2.364010	0.337746	H	6.732991	-2.320681	2.473300
C	1.889345	-0.215174	3.744142	H	6.979380	-1.209903	1.081363
C	0.947179	-1.438362	3.561254	H	3.989998	-3.034622	-0.637004
C	2.062531	-2.092238	2.678766	H	4.278435	-1.257452	-0.609372
C	2.704946	-0.688252	2.514528	H	5.665293	-2.406358	-0.501482
C	4.223586	-0.463472	2.625497				

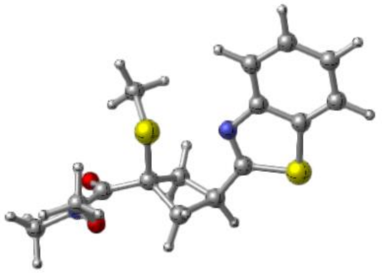

Zero-point correction= 0.309555 (Hartree/Particle)

Thermal correction to Energy= 0.330245

Thermal correction to Enthalpy= 0.331189

Thermal correction to Gibbs Free Energy= 0.258824

Sum of electronic and zero-point Energies= -1637.402337

Sum of electronic and thermal Energies= -1637.381647

Sum of electronic and thermal Enthalpies= -1637.380703

Sum of electronic and thermal Free Energies= -1637.453068

UB3LYP-D3/def2TZVPP

E(scf)= -1638.920056

UM062X-D3/def2TZVPP

E(scf)= -1638.458373

UB3LYP-D3/def2TZVPP (gas)

E(scf)= -1638.855364

2aj (EWG=CO_2Me)

E(scf) = -614.476000208 a.u.

ν_{min} = 24.8765 cm^{-1}

C	-2.240363	0.300756	-0.201259	H	-2.172169	3.390555	-3.799695
C	-0.815399	-0.152325	-0.297086	C	-0.106029	-0.761403	-1.435174
C	-0.249206	0.620006	0.856085	C	-0.797178	-1.131165	-2.605752
C	-1.159886	1.336253	-0.093990	C	1.286050	-0.967004	-1.384071
C	-0.747201	2.346375	-1.073114	C	-0.112002	-1.667090	-3.698125
O	0.311029	2.942547	-1.043582	H	-1.879627	-1.002859	-2.662676
C	-1.320233	3.394711	-3.109407	C	1.968327	-1.505102	-2.478241
H	-2.831670	0.303742	-1.120770	H	1.838703	-0.712385	-0.477637
H	-2.809705	0.062172	0.707899	C	1.274147	-1.853990	-3.641710
H	-0.620990	0.412950	1.868969	H	-0.665928	-1.945168	-4.598404
H	0.810009	0.889807	0.804044	H	3.049109	-1.656746	-2.418513
H	-1.149327	4.410686	-2.722550	H	1.808021	-2.277452	-4.495986
H	-0.410146	3.055810	-3.627380	O	-1.662108	2.497903	-2.047380

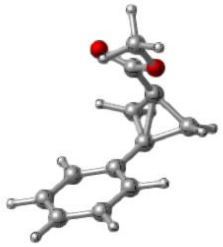

Zero-point correction= 0.210872 (Hartree/Particle)

Thermal correction to Energy= 0.223283

Thermal correction to Enthalpy= 0.224227

Thermal correction to Gibbs Free Energy= 0.170350

Sum of electronic and zero-point Energies= -614.265129

Sum of electronic and thermal Energies= -614.252717

Sum of electronic and thermal Enthalpies= -614.251773

Sum of electronic and thermal Free Energies= -614.305650

$[2aj]^{+\cdot}$ (EWG=CO_2Me)

E(scf) = -614.251720288 a.u.

ν_{min} = 33.1935 cm^{-1}

C	-0.664828	1.883479	-0.697653	H	3.857096	3.783172	-0.021943
C	-1.055135	0.592527	-1.367372	H	2.849219	4.664475	1.185217
C	0.353672	0.060561	-1.335033	H	3.571591	3.063289	1.590721
C	0.744901	1.353251	-0.672290	C	-2.260213	0.086446	-1.838455
C	2.020545	1.853092	-0.217477	C	-3.460356	0.857661	-1.714196
O	3.065913	1.230163	-0.308006	C	-2.308582	-1.208270	-2.448321
C	3.125794	3.678417	0.794134	C	-4.656476	0.346665	-2.182568
H	-0.848062	2.789580	-1.307400	H	-3.421930	1.844436	-1.249097
H	-1.152527	2.055680	0.281673	C	-3.512802	-1.704270	-2.912005
H	0.477112	-0.863565	-0.736817	H	-1.393027	-1.795050	-2.541950
H	0.790876	-0.145717	-2.331297	C	-4.680156	-0.928647	-2.778430

H	-5.577767	0.924098	-2.093106	H	-5.628922	-1.326780	-3.146806
H	-3.562829	-2.689310	-3.378297	O	1.914648	3.084421	0.314265

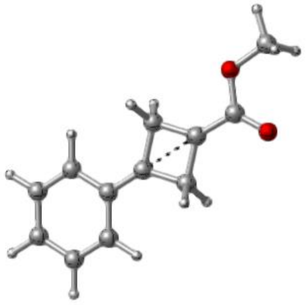

Zero-point correction= 0.207916 (Hartree/Particle)

Thermal correction to Energy= 0.221108

Thermal correction to Enthalpy= 0.222053

Thermal correction to Gibbs Free Energy= 0.166413

Sum of electronic and zero-point Energies= -614.043804

Sum of electronic and thermal Energies= -614.030612

Sum of electronic and thermal Enthalpies= -614.029668

Sum of electronic and thermal Free Energies= -614.085307

J(EWG=C(O)NMeOMe)

E(scf) = -1410.37240829 a.u.

ν_{min} = 27.1787 cm^{-1}

C	0.633486	-0.999270	-0.755677	O	-1.964493	0.047435	-1.545109
C	2.008632	-0.996720	0.000348	C	-2.056734	-0.766547	-2.721803
C	1.812431	0.541603	0.214261	H	0.786720	-0.927993	-1.839483
C	0.321699	0.397575	-0.163201	H	-0.069169	-1.815164	-0.543273
C	-0.236304	1.539540	-1.028417	H	2.072644	0.955556	1.195653
N	-1.331793	1.258335	-1.783549	H	2.324377	1.125029	-0.560935
O	0.291350	2.643102	-0.994517	H	-1.565157	3.216412	-2.407987
C	-2.125433	2.275463	-2.450533	H	-2.301141	2.006662	-3.502067

H	-3.090444	2.388498	-1.931745	S	-0.819240	0.240464	1.278225
H	-2.435843	-1.735101	-2.370889	C	-0.368622	1.684224	2.269789
H	-2.765791	-0.339067	-3.448482	H	0.527421	1.465400	2.866714
H	-1.071333	-0.897263	-3.195442	H	-0.180023	2.530179	1.591920
C	3.224952	-1.427684	-0.791876	H	-1.219957	1.892685	2.930465
C	3.145754	-2.496025	-1.697728	C	1.893869	-1.733590	1.326441
C	4.459972	-0.791098	-0.596690	C	2.757943	-2.761052	1.754617
C	4.279454	-2.917237	-2.399910	N	0.901250	-1.379583	2.148362
H	2.193244	-3.008514	-1.855301	N	2.607550	-3.371687	2.929087
C	5.594104	-1.211012	-1.298214	H	3.583954	-3.079613	1.114651
H	4.539354	0.038346	0.110686	C	0.720529	-1.972586	3.321715
C	5.506523	-2.275290	-2.202199	C	1.602469	-2.988038	3.714966
H	4.202503	-3.749203	-3.104209	H	-0.115060	-1.647113	3.946882
H	6.548824	-0.703938	-1.138424	H	1.475323	-3.485994	4.680976
H	6.392163	-2.602465	-2.752349				

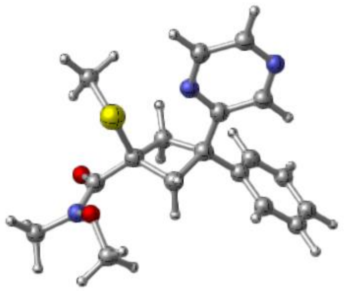

Zero-point correction= 0.364138 (Hartree/Particle)

Thermal correction to Energy= 0.387292

Thermal correction to Enthalpy= 0.388236

Thermal correction to Gibbs Free Energy= 0.310053

Sum of electronic and zero-point Energies= -1410.008270

Sum of electronic and thermal Energies= -1409.985117

Sum of electronic and thermal Enthalpies= -1409.984172

Sum of electronic and thermal Free Energies= -1410.062355

UB3LYP-D3/def2TZVPP

E(scf)= -1411.627419

UM062X-D3/def2TZVPP

E(scf)= -1411.120801

UB3LYP-D3/def2TZVPP (gas)

E(scf)= -1411.559357

J′(EWG=C(O)NMeOMe)

E(scf) = -1410.37548042 a.u.

ν_{min} = 35.9056 cm^{-1}

C	0.138469	-1.509924	-2.040754	C	-1.316438	0.031155	-1.578688
C	-0.046692	-0.513809	-0.870616	C	-1.322084	-1.228512	-2.507262
C	-1.435847	-0.967503	-4.014179	C	1.793487	2.545481	0.452641
N	-2.562379	-0.315420	-4.428651	H	-0.275307	1.974957	0.216017
O	-0.553723	-1.305077	-4.790516	C	3.130321	2.167907	0.278277
C	-2.957988	-0.206813	-5.821177	H	4.478519	0.624573	-0.420383
O	-3.583373	-0.176021	-3.495790	H	1.549948	3.519235	0.883835
C	-3.958657	1.190712	-3.291817	H	3.933613	2.846776	0.574210
H	0.855780	-1.112374	-2.769292	S	-0.592066	-1.306466	0.727318
H	0.403947	-2.550835	-1.819282	C	-2.264542	-2.311656	-2.019418
H	-2.215576	0.210426	-0.974810	C	-3.159127	-3.037560	-2.830292
H	-1.069114	0.943187	-2.137293	N	-2.208736	-2.622789	-0.723084
H	-2.093778	-0.484841	-6.435037	N	-3.939765	-3.996837	-2.340345
H	-3.798989	-0.888403	-6.028130	H	-3.233802	-2.825778	-3.899586
H	-3.255104	0.823853	-6.063000	C	-2.980072	-3.571038	-0.202889
H	-4.658249	1.173301	-2.445862	C	-3.862532	-4.267825	-1.036893
H	-3.084903	1.812636	-3.043014	H	-2.897287	-3.779112	0.867048
H	-4.468928	1.603790	-4.176803	H	-4.506311	-5.053126	-0.629430
C	1.068000	0.424962	-0.489362	C	0.628173	-2.606767	0.999785
C	2.411252	0.051741	-0.660122	H	1.635085	-2.168565	0.936437
C	0.767063	1.678233	0.074895	H	0.449129	-2.998909	2.009269
C	3.436728	0.920982	-0.278328	H	0.507440	-3.405454	0.255634
H	2.660537	-0.914757	-1.103955				

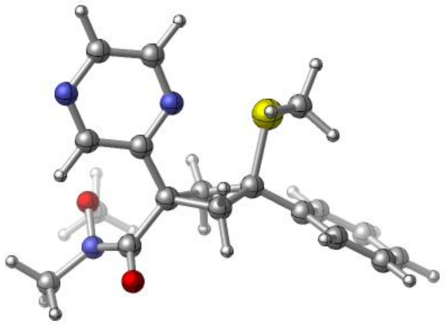

Zero-point correction= 0.364169 (Hartree/Particle)

Thermal correction to Energy= 0.387332

Thermal correction to Enthalpy= 0.388276

Thermal correction to Gibbs Free Energy= 0.310581

Sum of electronic and zero-point Energies= -1410.011312

Sum of electronic and thermal Energies= -1409.988149

Sum of electronic and thermal Enthalpies= -1409.987205

Sum of electronic and thermal Free Energies= -1410.064900

UB3LYP-D3/def2TZVPP

E(scf)= -1411.63065796

UM062X-D3/def2TZVPP

E(scf)= -1411.12303731

UB3LYP-D3/def2TZVPP (gas)

E(scf)= -1411.56192657

^{3}C'(OST)

E(scf) = -1179.54146405 a.u.

ν_{min} = 18.5742 cm^{-1}

C	-2.524936	-2.763723	-0.725409	N	-2.717612	-4.025893	-1.243995
C	-3.460150	-2.106548	0.102111	H	-3.234170	-1.097744	0.476239
N	-4.607162	-2.657596	0.451212	H	-5.760672	-4.422926	0.203635
C	-4.819987	-3.931086	-0.061924	H	-4.114768	-5.575883	-1.253261
C	-3.896708	-4.568834	-0.874418	S	-1.050852	-1.939624	-1.139014

C	0.271739	-3.181075	-0.842074
H	-0.108745	-4.151771	-1.185210
H	1.166371	-2.872208	-1.398542
H	0.468264	-3.187303	0.237314
C	0.188425	-1.273570	-3.658801
C	-1.012350	-2.009861	-2.996399
C	-1.997513	-1.012007	-3.658721
C	-0.809817	-0.371020	-4.320969
C	-0.514108	0.737257	-5.209442
N	-1.569976	1.436100	-5.766746
O	0.656321	1.031798	-5.482039
C	-1.400526	2.735838	-6.382838
O	-2.830360	1.229577	-5.213111
C	-3.704836	0.587222	-6.141521
H	0.882604	-0.760096	-2.967740
H	0.798986	-1.898086	-4.333751
H	-1.152698	-3.065120	-3.261793
H	-2.551620	-0.355931	-2.963412
H	-2.741874	-1.477231	-4.325480
H	-0.381111	2.793445	-6.781247
H	-2.119028	2.858788	-7.206404
H	-1.552896	3.544901	-5.646803
H	-4.656999	0.454763	-5.609527
H	-3.871879	1.209706	-7.036850
H	-3.309143	-0.395468	-6.447987
C	-2.524936	-2.763723	-0.725409
C	-3.460150	-2.106548	0.102111
N	-4.607162	-2.657596	0.451212
C	-4.819987	-3.931086	-0.061924
C	-3.896708	-4.568834	-0.874418
N	-2.717612	-4.025893	-1.243995
H	-3.234170	-1.097744	0.476239
H	-5.760672	-4.422926	0.203635
H	-4.114768	-5.575883	-1.253261
S	-1.050852	-1.939624	-1.139014

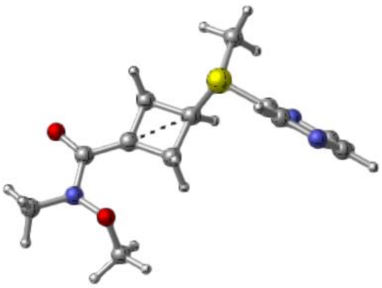

Zero-point correction= 0.276918 (Hartree/Particle)

Thermal correction to Energy= 0.296472

Thermal correction to Enthalpy= 0.297416

Thermal correction to Gibbs Free Energy= 0.225570

Sum of electronic and zero-point Energies= -1179.264546

Sum of electronic and thermal Energies= -1179.244992

Sum of electronic and thermal Enthalpies= -1179.244048

Sum of electronic and thermal Free Energies= -1179.315894

[²PC]•

E(scf) = -1567.65497813 a.u.

ν_{min} = 7.6398 cm^{-1}

C	-3.469948	1.197925	0.037039	C	0.587363	2.822718	-2.430137
C	-3.689746	-0.196755	0.038494	C	0.452700	2.848892	2.625051
C	-2.562094	-1.029385	0.059839	C	1.245998	7.164316	0.094277
C	-1.254111	-0.516830	0.079244	C	-0.680377	-4.861456	-1.108044
C	-1.034106	0.894799	0.078349	C	-0.811025	-5.568319	0.097014
C	-2.186039	1.724290	0.056220	C	-0.716433	-4.857513	1.302442
N	-0.153527	-1.379822	0.099347	C	-0.501970	-3.473015	1.326431
C	1.163107	-0.907086	0.119389	C	-0.380503	-2.800851	0.098515
C	1.398857	0.505173	0.119325	C	-0.465284	-3.477671	-1.130616
C	0.296611	1.413388	0.098540	C	-0.394577	-2.707958	2.620156
C	2.243122	-1.797637	0.139343	C	-0.319919	-2.716706	-2.423055
C	3.580485	-1.360782	0.159531	C	-1.077601	-7.053797	0.092629
C	3.810730	0.026989	0.159642	C	-5.122314	-0.755726	0.017304
C	2.750244	0.929701	0.140141	C	-5.149523	-2.294577	0.021103
C	0.674951	3.576433	-1.125089	C	-5.844585	-0.261784	-1.257256
C	0.532819	2.885227	0.097506	C	-5.884380	-0.254799	1.265697
C	0.609445	3.589040	1.318741	C	4.707164	-2.407543	0.179910
C	0.827967	4.973526	1.297546	C	6.102110	-1.758906	0.200667
C	0.974954	5.678914	0.094863	C	4.604140	-3.296635	-1.081182
C	0.892669	4.961019	-1.106645	C	4.563924	-3.291214	1.440879

H	-4.317748	1.885961	0.020491	H	2.028014	-2.864761	0.138912
H	-2.681300	-2.109112	0.061736	H	4.825905	0.422885	0.174968
H	-2.042129	2.806556	0.054460				

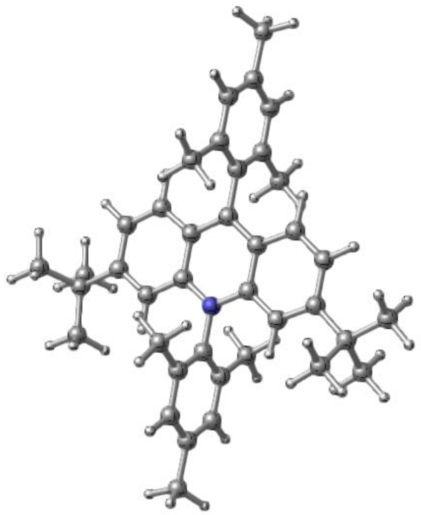

Zero-point correction= 0.741409 (Hartree/Particle)

Thermal correction to Energy= 0.782301

Thermal correction to Enthalpy= 0.783246

Thermal correction to Gibbs Free Energy= 0.663200

Sum of electronic and zero-point Energies= -1566.913569

Sum of electronic and thermal Energies= -1566.872677

Sum of electronic and thermal Enthalpies= -1566.871732

Sum of electronic and thermal Free Energies= -1566.991778

[^{3}PC]$^{+*}$

E(scf) = -1567.44837215 a.u.

ν_{min} = 13.9953 cm^{-1}

C	-3.466466	1.185805	0.039273	C	-1.024733	0.915872	0.076809
C	-3.690904	-0.195163	0.039125	C	-2.165884	1.729139	0.057785
C	-2.551248	-1.022367	0.058499	N	-0.154241	-1.361629	0.096523
C	-1.246260	-0.496295	0.077035	C	1.156761	-0.891638	0.115937

C	1.403593	0.519042	0.115503
C	0.312096	1.443146	0.095972
C	2.223089	-1.801846	0.135120
C	3.580545	-1.384509	0.154103
C	3.813995	-0.014241	0.153570
C	2.747918	0.918485	0.134639
C	0.671358	3.600956	-1.130242
C	0.551879	2.910964	0.095612
C	0.652493	3.604568	1.321135
C	0.873650	4.987718	1.298481
C	0.998890	5.696047	0.094962
C	0.892245	4.984169	-1.108248
C	0.558786	2.851748	-2.435917
C	0.518879	2.859354	2.627118
C	1.272555	7.180214	0.094945
C	-0.703344	-4.839362	-1.107728
C	-0.834788	-5.542187	0.097598
C	-0.738665	-4.831013	1.304344
C	-0.514001	-3.450857	1.333166
C	-0.387757	-2.792922	0.096541
C	-0.477503	-3.456570	-1.137326
C	-0.410555	-2.687407	2.628121
C	-0.337017	-2.702085	-2.434048
C	-1.058941	-7.033238	0.112497
C	-5.117406	-0.764191	0.019727
C	-5.124770	-2.303000	0.018104
C	-5.841008	-0.267525	-1.252861
C	-5.873417	-0.269724	1.274272
C	4.682364	-2.449988	0.172985
C	6.085453	-1.821795	0.191330
C	4.551848	-3.333083	-1.090365
C	4.515390	-3.326081	1.436871
H	-4.309570	1.878515	0.024925
H	-2.662968	-2.102802	0.059679
H	-2.037513	2.812695	0.057555
H	1.991011	-2.865025	0.135074
H	4.829689	0.378962	0.167650

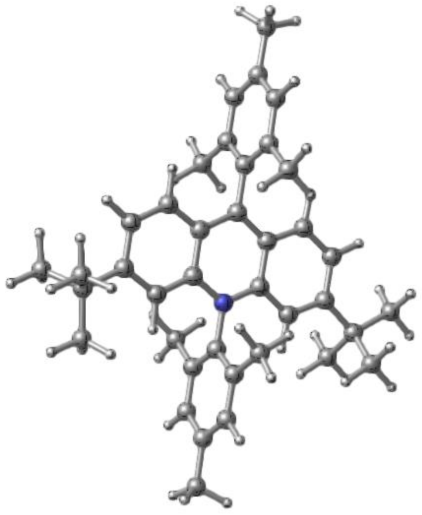

Zero-point correction= 0.740622 (Hartree/Particle)
Thermal correction to Energy= 0.781850
Thermal correction to Enthalpy= 0.782794
Thermal correction to Gibbs Free Energy= 0.662223
Sum of electronic and zero-point Energies= -1566.707750
Sum of electronic and thermal Energies= -1566.666522
Sum of electronic and thermal Enthalpies= -1566.665578
Sum of electronic and thermal Free Energies= -1566.786149

^{3}C(OST)

E(scf) = -1179.56794672 a.u.

ν_{min} = 18.3510 cm^{-1}

C	-1.911215	-0.090271	-0.669138	C	-0.250542	-2.593498	-2.892924
C	-1.498410	-0.629640	0.574121	C	0.210752	-1.339822	-3.442214
N	-0.660273	0.020158	1.368163	N	1.075374	-0.555525	-2.693874
C	-0.218827	1.215495	0.947060	O	-0.128697	-0.972069	-4.578023
C	-0.633767	1.749563	-0.271341	C	1.333247	0.818466	-3.087243
N	-1.478804	1.098076	-1.079350	O	1.045308	-0.759140	-1.314487
H	-1.869066	-1.605829	0.907609	C	2.318785	-1.138144	-0.803952
H	0.479716	1.759171	1.590740	H	-0.462807	-2.920785	-0.709516
H	-0.267857	2.725186	-0.608259	H	0.936760	-3.753022	-1.405210
S	-3.026179	-1.040620	-1.665125	H	-1.438265	-5.366643	-1.959461
C	-3.168982	-0.010364	-3.158091	H	-2.177539	-3.224397	-3.789295
H	-3.970901	-0.468000	-3.753780	H	-0.766941	-4.048559	-4.489859
H	-3.439214	1.018124	-2.886788	H	1.588318	0.836328	-4.153525
H	-2.229727	-0.022404	-3.727575	H	0.451121	1.457921	-2.917004
C	-0.096199	-3.413125	-1.631942	H	2.182251	1.201286	-2.505690
C	-1.013468	-4.417194	-2.288590	H	2.167920	-1.281085	0.274889
C	-1.160194	-3.600553	-3.551868	H	2.671285	-2.077338	-1.261318

H	3.072158	-0.347370	-0.962563	N	-1.478804	1.098076	-1.079350
C	-1.911215	-0.090271	-0.669138	H	-1.869066	-1.605829	0.907609
C	-1.498410	-0.629640	0.574121	H	0.479716	1.759171	1.590740
N	-0.660273	0.020158	1.368163	H	-0.267857	2.725186	-0.608259
C	-0.218827	1.215495	0.947060	S	-3.026179	-1.040620	-1.665125
C	-0.633767	1.749563	-0.271341				

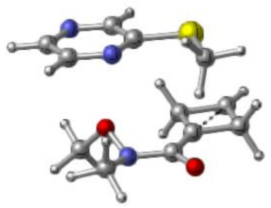

Zero-point correction= 0.274411 (Hartree/Particle)

Thermal correction to Energy= 0.295184

Thermal correction to Enthalpy= 0.296128

Thermal correction to Gibbs Free Energy= 0.222442

Sum of electronic and zero-point Energies= -1179.293535

Sum of electronic and thermal Energies= -1179.272763

Sum of electronic and thermal Enthalpies= -1179.271818

Sum of electronic and thermal Free Energies= -1179.345505

^{1}C(OSS)

E(scf) = -1179.56630581 a.u.

ν_{min} = 19.5556 cm^{-1}

C	-1.920294	-0.115918	-0.663632	N	-1.502798	1.077953	-1.073357
C	-1.499941	-0.651158	0.578817	H	-1.858722	-1.631857	0.912121
N	-0.669058	0.008237	1.372686	H	0.449772	1.760823	1.595608
C	-0.242632	1.209110	0.952133	H	-0.311629	2.719426	-0.601848
C	-0.665202	1.739059	-0.265535	S	-3.024016	-1.079210	-1.659553

C	-3.178657	-0.050997	-3.152648	H	-0.726163	-4.048016	-4.487416
H	-3.973359	-0.519624	-3.749481	H	1.574620	0.872481	-4.145506
H	-3.463664	0.973578	-2.881689	H	0.431580	1.474428	-2.904885
H	-2.238473	-0.049877	-3.720748	H	2.165661	1.234210	-2.495909
C	-0.079685	-3.401790	-1.640896	H	2.173741	-1.265345	0.272355
C	-0.999997	-4.409025	-2.292188	H	2.681924	-2.052040	-1.267142
C	-1.135194	-3.594834	-3.560572	H	3.072420	-0.321026	-0.961145
C	-0.232385	-2.578908	-2.901965	C	-1.920294	-0.115918	-0.663632
C	0.218933	-1.321953	-3.446161	C	-1.499941	-0.651158	0.578817
N	1.078402	-0.533595	-2.693922	N	-0.669058	0.008237	1.372686
O	-0.122451	-0.950579	-4.580901	C	-0.242632	1.209110	0.952133
C	1.320500	0.845430	-3.079198	C	-0.665202	1.739059	-0.265535
O	1.048189	-0.743392	-1.315161	N	-1.502798	1.077953	-1.073357
C	2.323674	-1.116891	-0.805905	H	-1.858722	-1.631857	0.912121
H	-0.443736	-2.907908	-0.720502	H	0.449772	1.760823	1.595608
H	0.952454	-3.744745	-1.423078	H	-0.311629	2.719426	-0.601848
H	-1.389260	-5.378686	-1.979737	S	-3.024016	-1.079210	-1.659553
H	-2.149433	-3.222987	-3.808463				

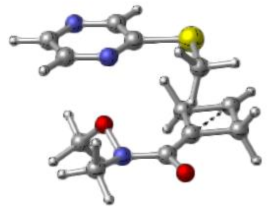

Zero-point correction= 0.274325 (Hartree/Particle)

Thermal correction to Energy= 0.295213

Thermal correction to Enthalpy= 0.296158

Thermal correction to Gibbs Free Energy= 0.223206

Sum of electronic and zero-point Energies= -1179.291981

Sum of electronic and thermal Energies= -1179.271092

Sum of electronic and thermal Enthalpies= -1179.270148

Sum of electronic and thermal Free Energies= -1179.343100

[1]C'

E(scf) = -1179.62073384 a.u.

ν_{min} = -13.6257 cm^{-1}

C	-2.507528	-2.634497	-0.674980	O	-2.847864	1.076798	-4.833345
C	-3.669311	-1.835856	-0.523376	C	-4.063045	0.363490	-4.610669
N	-4.650051	-1.863994	-1.414617	H	1.243260	0.421831	-2.473921
C	-4.505329	-2.689020	-2.464894	H	1.635625	-0.579371	-3.984072
C	-3.368009	-3.483844	-2.606497	H	-0.366210	-2.219623	-3.271229
N	-2.370316	-3.455286	-1.714225	H	-1.127097	0.469940	-1.737016
H	-3.775750	-1.165478	0.337131	H	-2.334263	-0.507957	-2.721275
H	-5.308171	-2.717264	-3.207794	H	-1.568114	0.180431	-7.743672
H	-3.256788	-4.155316	-3.464316	H	-3.309919	0.388833	-7.394661
S	-1.230659	-2.498529	0.541198	H	-2.206311	1.802169	-7.309981
C	0.018245	-3.645804	-0.115822	H	-4.564745	0.877540	-3.779334
H	0.398286	-3.286442	-1.081118	H	-4.718675	0.383516	-5.498071
H	0.828197	-3.659920	0.626162	H	-3.865965	-0.683400	-4.330132
H	-0.405110	-4.651833	-0.233821	C	-2.507528	-2.634497	-0.674980
C	0.877212	-0.242124	-3.271231	C	-3.669311	-1.835856	-0.523376
C	-0.242462	-1.176283	-2.983886	N	-4.650051	-1.863994	-1.414617
C	-1.297447	-0.188481	-2.601773	C	-4.505329	-2.689020	-2.464894
C	-0.491460	0.034520	-3.848618	C	-3.368009	-3.483844	-2.606497
C	-0.768165	0.034386	-5.303763	N	-2.370316	-3.455286	-1.714225
N	-2.021238	0.411847	-5.733870	H	-3.775750	-1.165478	0.337131
O	0.095025	-0.303947	-6.111752	H	-5.308171	-2.717264	-3.207794
C	-2.295637	0.717758	-7.125677	H	-3.256788	-4.155316	-3.464316

S	-1.230659	-2.498529	0.541198

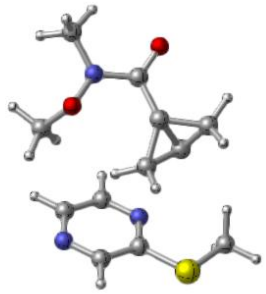

Zero-point correction= 0.278950 (Hartree/Particle)

Thermal correction to Energy= 0.297430

Thermal correction to Enthalpy= 0.298374

Thermal correction to Gibbs Free Energy= 0.230263

Sum of electronic and zero-point Energies= -1179.341783

Sum of electronic and thermal Energies= -1179.323304

Sum of electronic and thermal Enthalpies= -1179.322360

Sum of electronic and thermal Free Energies= -1179.390471

^{1}C

E(scf) = -1179.62610538 a.u.

ν_{min} = 23.9150 cm^{-1}

C	-1.791942	0.482173	-0.577234
C	-1.051142	0.441861	0.631265
N	-0.380016	1.498430	1.066706
C	-0.419577	2.609255	0.311250
C	-1.128390	2.639842	-0.889213
N	-1.814993	1.578875	-1.331977
H	-1.011133	-0.476122	1.227127
H	0.130662	3.487344	0.663164
H	-1.140485	3.541669	-1.510445
S	-2.697850	-0.958569	-1.054533
C	-3.306075	-0.475506	-2.699227
H	-3.905413	-1.325910	-3.051772
H	-3.928740	0.425849	-2.631702
H	-2.458793	-0.307876	-3.377368
C	0.028022	-4.109111	-2.275474
C	-0.202537	-4.043135	-3.755162
C	-1.550183	-3.412493	-3.759547
C	-0.459657	-2.835147	-2.894592

C	0.096606	-1.477940	-3.083717
N	0.928880	-0.974052	-2.117925
O	-0.178748	-0.813616	-4.085801
C	1.430830	0.387487	-2.137984
O	0.972010	-1.604955	-0.882619
C	2.286585	-2.048783	-0.551690
H	-0.665886	-4.687780	-1.646395
H	1.068289	-4.140326	-1.940721
H	0.519733	-4.128310	-4.567046
H	-2.390065	-3.917674	-3.258729
H	-1.819663	-2.822901	-4.640732
H	0.988547	0.901928	-2.998109
H	1.136269	0.895545	-1.209515
H	2.528717	0.412399	-2.227374
H	2.179008	-2.614690	0.383625
H	2.700071	-2.702352	-1.338001
H	2.972180	-1.201033	-0.383958
C	-1.791942	0.482173	-0.577234
C	-1.051142	0.441861	0.631265
N	-0.380016	1.498430	1.066706
C	-0.419577	2.609255	0.311250
C	-1.128390	2.639842	-0.889213
N	-1.814993	1.578875	-1.331977
H	-1.011133	-0.476122	1.227127
H	0.130662	3.487344	0.663164
H	-1.140485	3.541669	-1.510445
S	-2.697850	-0.958569	-1.054533

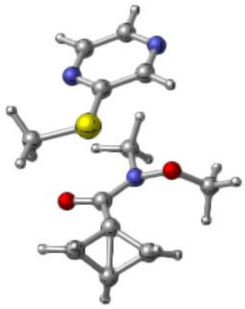

Zero-point correction= 0.279542 (Hartree/Particle)

Thermal correction to Energy= 0.299458

Thermal correction to Enthalpy= 0.300402

Thermal correction to Gibbs Free Energy= 0.228612

Sum of electronic and zero-point Energies= -1179.346563

Sum of electronic and thermal Energies= -1179.326648

Sum of electronic and thermal Enthalpies= -1179.325703

Sum of electronic and thermal Free Energies= -1179.397493

[[1]PC]+

E(scf) = -1567.51985650 a.u.

ν_{min} = 15.8832 cm^{-1}

C	-3.432280	1.220179	0.040297	C	0.663627	3.552457	-1.131612
C	-3.656864	-0.193846	0.039874	C	0.540882	2.869800	0.096665
C	-2.557135	-1.035312	0.058796	C	0.638761	3.556072	1.325007
C	-1.236898	-0.524733	0.077798	C	0.862910	4.938415	1.300356
C	-1.017409	0.891120	0.077808	C	0.992590	5.645963	0.097100
C	-2.165393	1.741450	0.058600	C	0.887427	4.934705	-1.106528
N	-0.159590	-1.377770	0.096458	C	0.551912	2.804830	-2.438076
C	1.137873	-0.916166	0.114963	C	0.500066	2.812558	2.631251
C	1.384001	0.495696	0.114793	C	1.269201	7.129151	0.096986
C	0.298256	1.398897	0.096470	C	-0.707970	-4.859156	-1.106609
C	2.222954	-1.817324	0.133548	C	-0.837936	-5.561605	0.099209
C	3.539165	-1.373927	0.151343	C	-0.741107	-4.850122	1.305925
C	3.779536	0.032218	0.150952	C	-0.517533	-3.469580	1.332652
C	2.740723	0.931785	0.133370	C	-0.393586	-2.811818	0.096971

C	-0.483253	-3.475902	-1.135300
C	-0.411739	-2.704056	2.626173
C	-0.342759	-2.720088	-2.431229
C	-1.062356	-7.052882	0.115111
C	-5.093062	-0.728632	0.019216
C	-5.144249	-2.266009	0.017532
C	-5.800487	-0.211057	-1.255548
C	-5.835627	-0.212424	1.274456
C	4.676285	-2.407358	0.170514
C	6.065875	-1.747195	0.185709
C	4.568398	-3.295708	-1.091413
C	4.534972	-3.284576	1.436954
H	-4.282749	1.902395	0.025836
H	-2.685378	-2.113085	0.059369
H	-2.010188	2.820928	0.058883
H	2.006999	-2.883094	0.133437
H	4.798667	0.413998	0.164562
H	2.940219	2.004057	0.133011
H	0.936624	5.476941	2.249554
H	0.980696	5.470433	-2.055615
H	-0.426249	2.305057	-2.529922
H	0.671978	3.478821	-3.297587
H	1.31788	2.015736	-2.513069
H	1.265544	2.025044	2.725395
H	0.600508	3.489568	3.490906
H	-0.479014	2.311395	2.703644
H	2.356214	7.32202	0.090922
H	0.846974	7.619182	-0.793447
H	0.856329	7.618052	0.992296
H	-0.783273	-5.398643	-2.054329
H	-0.843117	-5.385498	2.253854
H	-0.530645	-3.370202	3.491103
H	0.565748	-2.201742	2.712733
H	-1.183345	-1.919264	2.689661
H	-1.115028	-1.938708	-2.52304
H	0.634995	-2.214769	-2.493657
H	-0.434506	-3.393264	-3.294009
H	-0.172422	-7.576043	0.503853
H	-1.903898	-7.319952	0.773968
H	-1.27186	-7.444975	-0.890439
H	-4.674469	-2.692825	0.917274
H	-6.193118	-2.598443	0.001857
H	-4.648016	-2.691607	-0.86851
H	-5.843762	0.887783	-1.289866
H	-6.835602	-0.586422	-1.284465
H	-5.282658	-0.562173	-2.161974
H	-6.871747	-0.586197	1.273367
H	-5.344119	-0.566069	2.19445
H	-5.877957	0.886441	1.309642
H	6.212392	-1.119488	1.078277
H	6.841567	-2.527759	0.198858
H	6.235224	-1.126751	-0.70793
H	3.614776	-3.84336	-1.130464
H	5.381687	-4.038558	-1.095927
H	4.65197	-2.689814	-2.007408
H	5.347671	-4.027364	1.469589
H	4.594356	-2.670621	2.349463
H	3.580423	-3.831712	1.455453

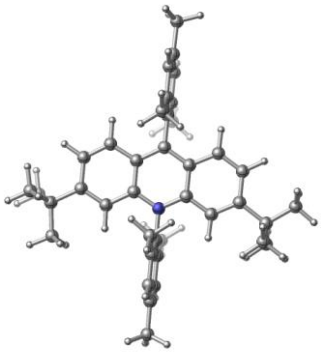

Zero-point correction= 0.744318 (Hartree/Particle)

Thermal correction to Energy= 0.785085

Thermal correction to Enthalpy= 0.786029

Thermal correction to Gibbs Free Energy= 0.668174

Sum of electronic and zero-point Energies= -1566.775539

Sum of electronic and thermal Energies= -1566.734771

Sum of electronic and thermal Enthalpies= -1566.733827

Sum of electronic and thermal Free Energies= -1566.851682

5 NMR spectra

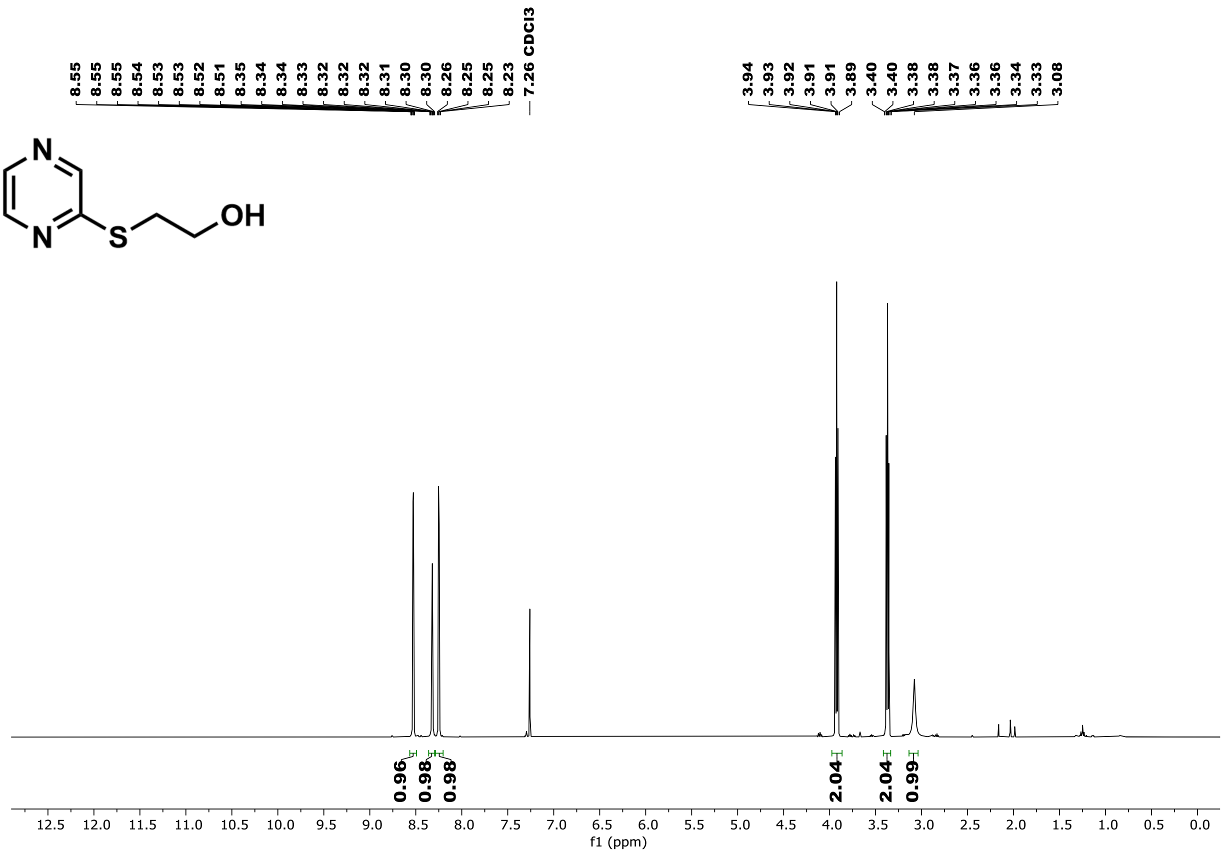

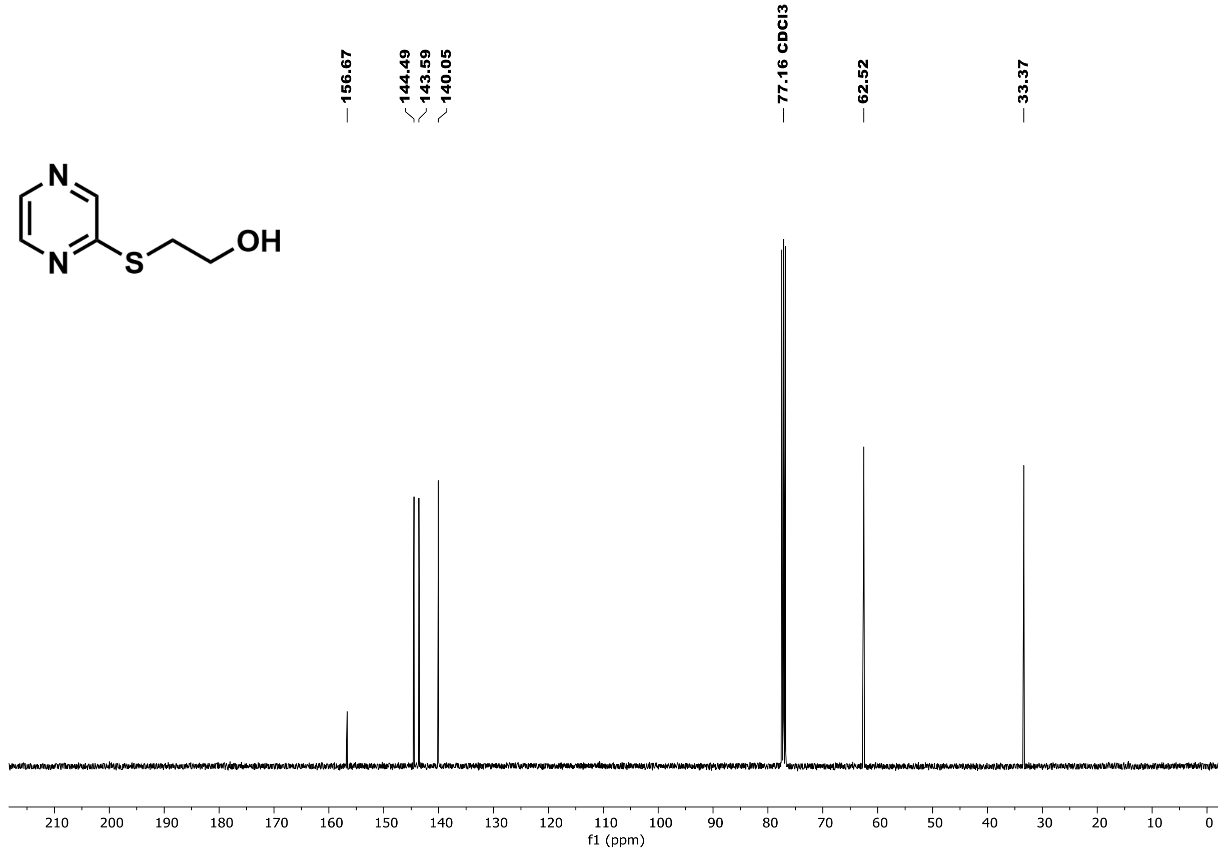

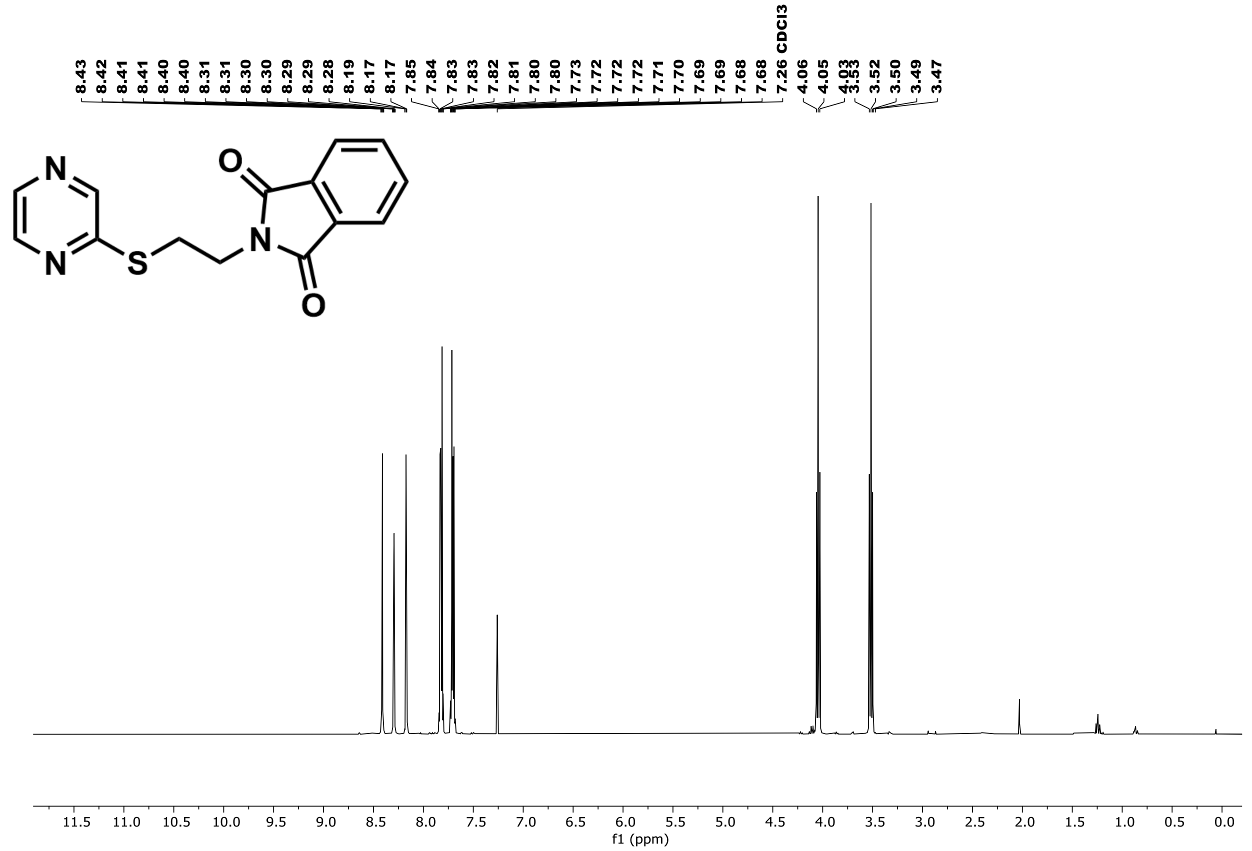
7.26 CDCl3
11.5 11.0 10.5 10.0 9.5 9.0 8.5 8.0 7.5 7.0 6.5 6.0 5.5 5.0 4.5 4.0 3.5 3.0 2.5 2.0 1.5 1.0 0.5 0.0
f1 (ppm)

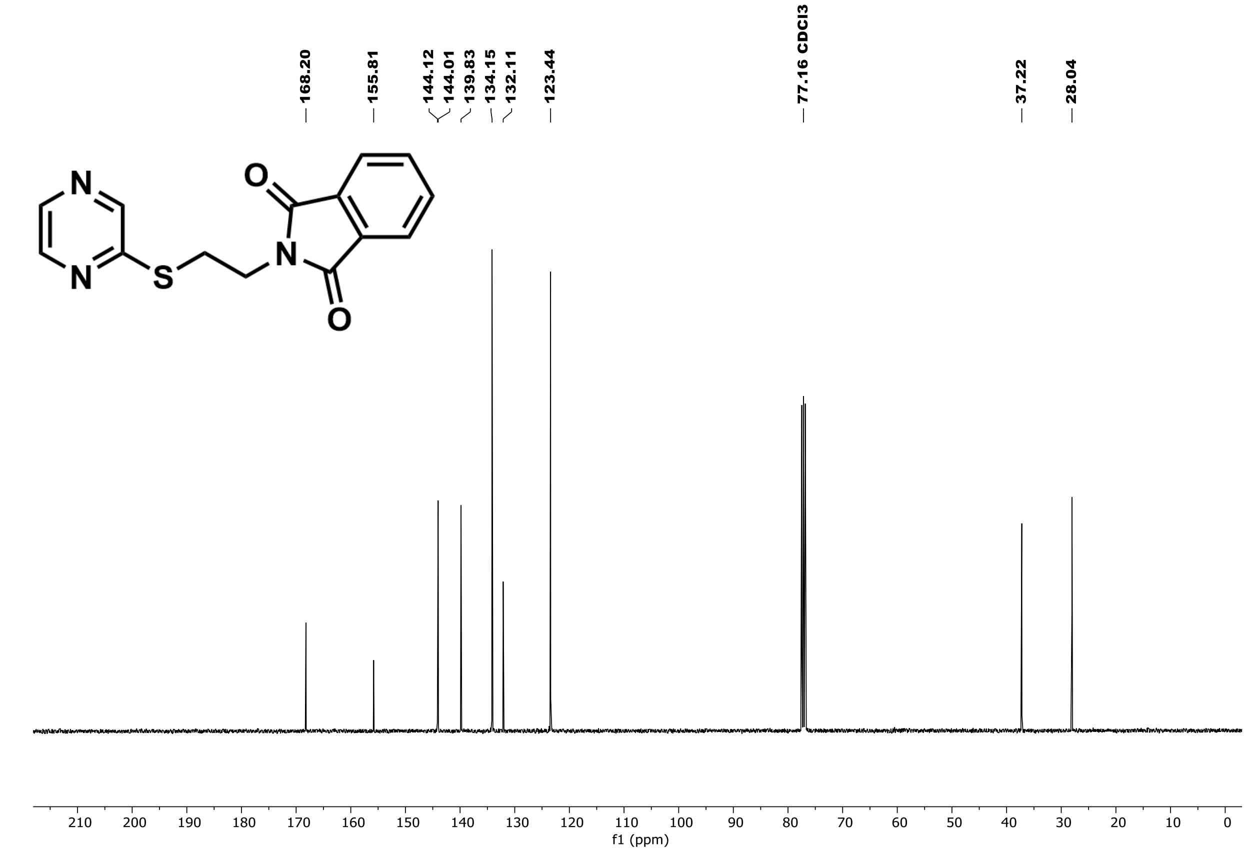
168.20
155.81
144.12
144.01
139.83
134.15
132.11
123.44
77.16 CDCl3
37.22
28.04
210 200 190 180 170 160 150 140 130 120 110 100 90 80 70 60 50 40 30 20 10 0
f1 (ppm)

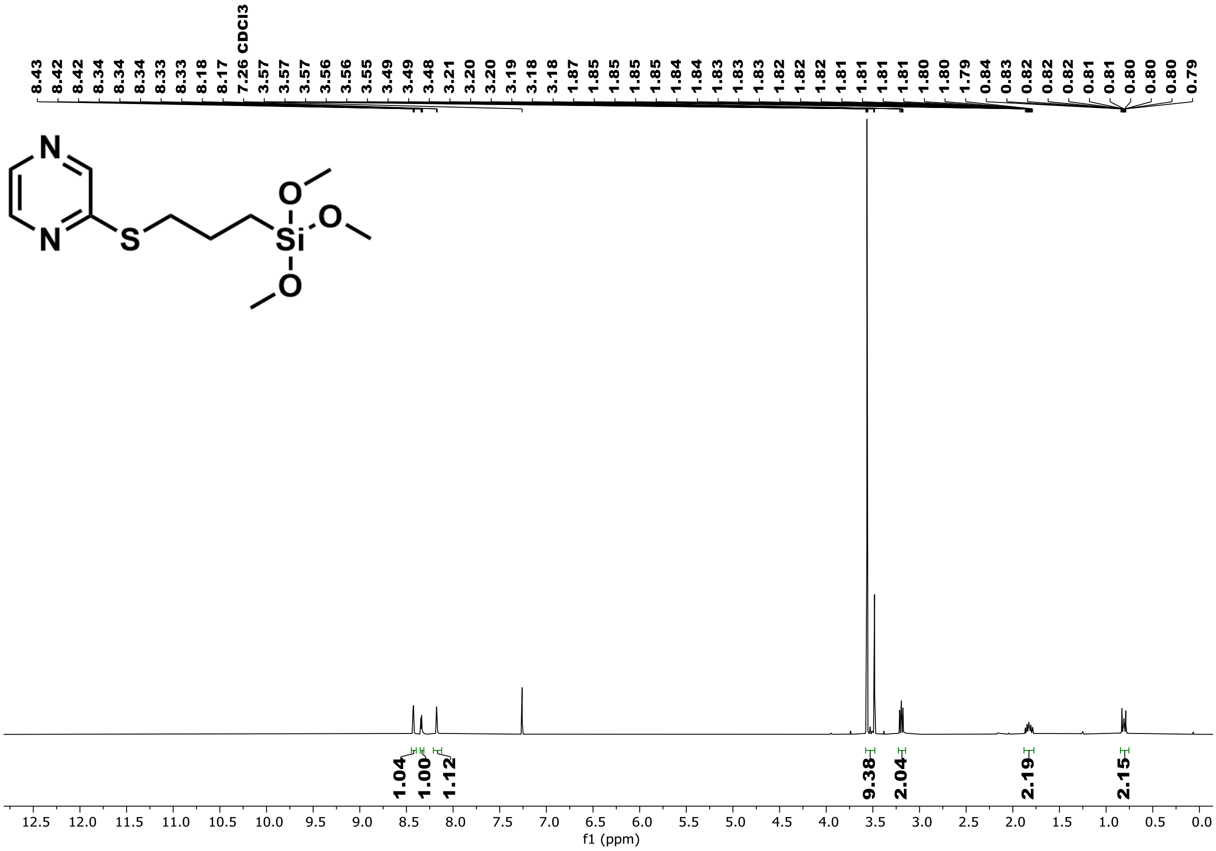
8.43
8.42
8.42
8.34
8.34
8.34
8.33
8.33
8.18
8.17
7.26 CDCl3
3.57
3.57
3.57
3.56
3.56
3.55
3.49
3.48
3.21
3.20
3.19
3.18
3.18
1.87
1.85
1.85
1.85
1.85
1.84
1.84
1.83
1.83
1.83
1.82
1.82
1.82
1.81
1.81
1.81
1.81
1.80
1.80
1.79
0.84
0.83
0.82
0.82
0.81
0.81
0.80
0.80
0.80
0.79
1.04
1.00
1.12
9.38
2.04
2.19
2.15
f1 (ppm)

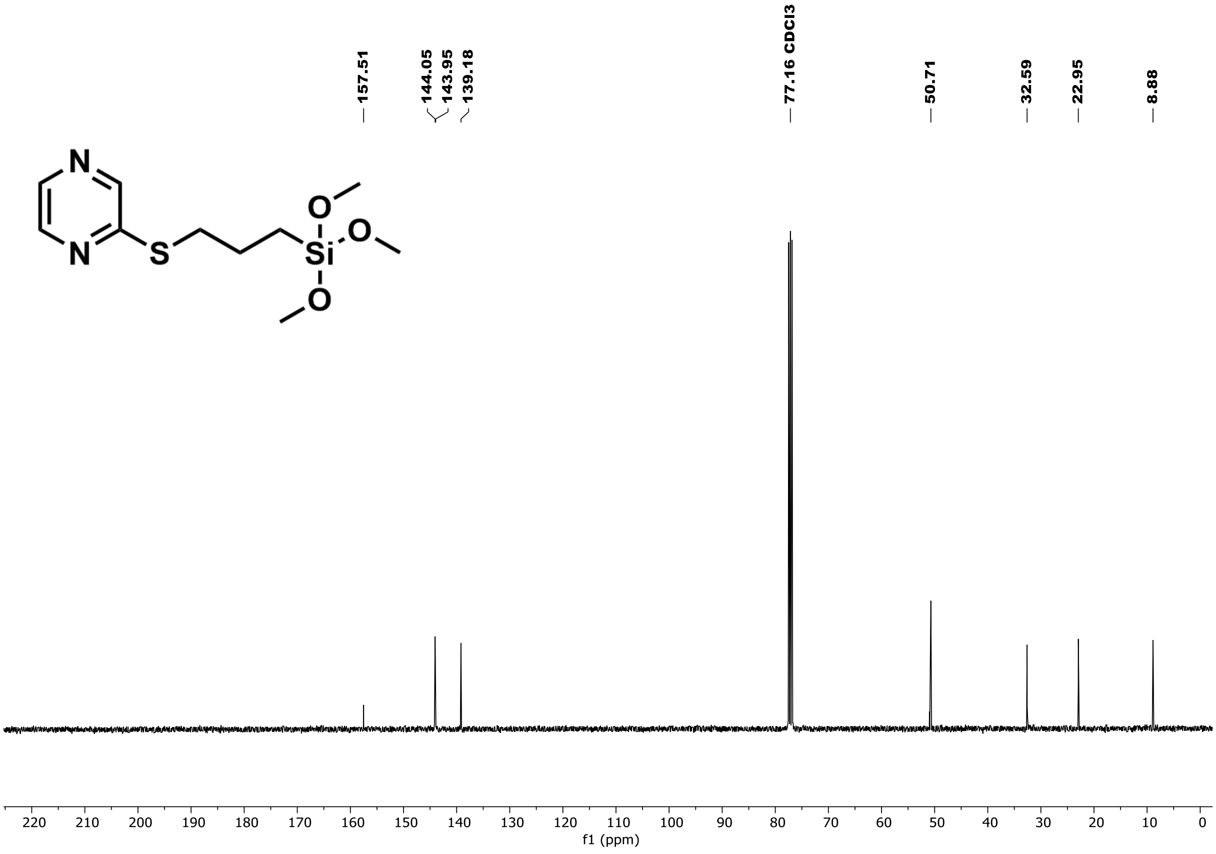
157.51
144.05
143.95
139.18
77.16 CDCl3
50.71
32.59
22.95
8.88
f1 (ppm)

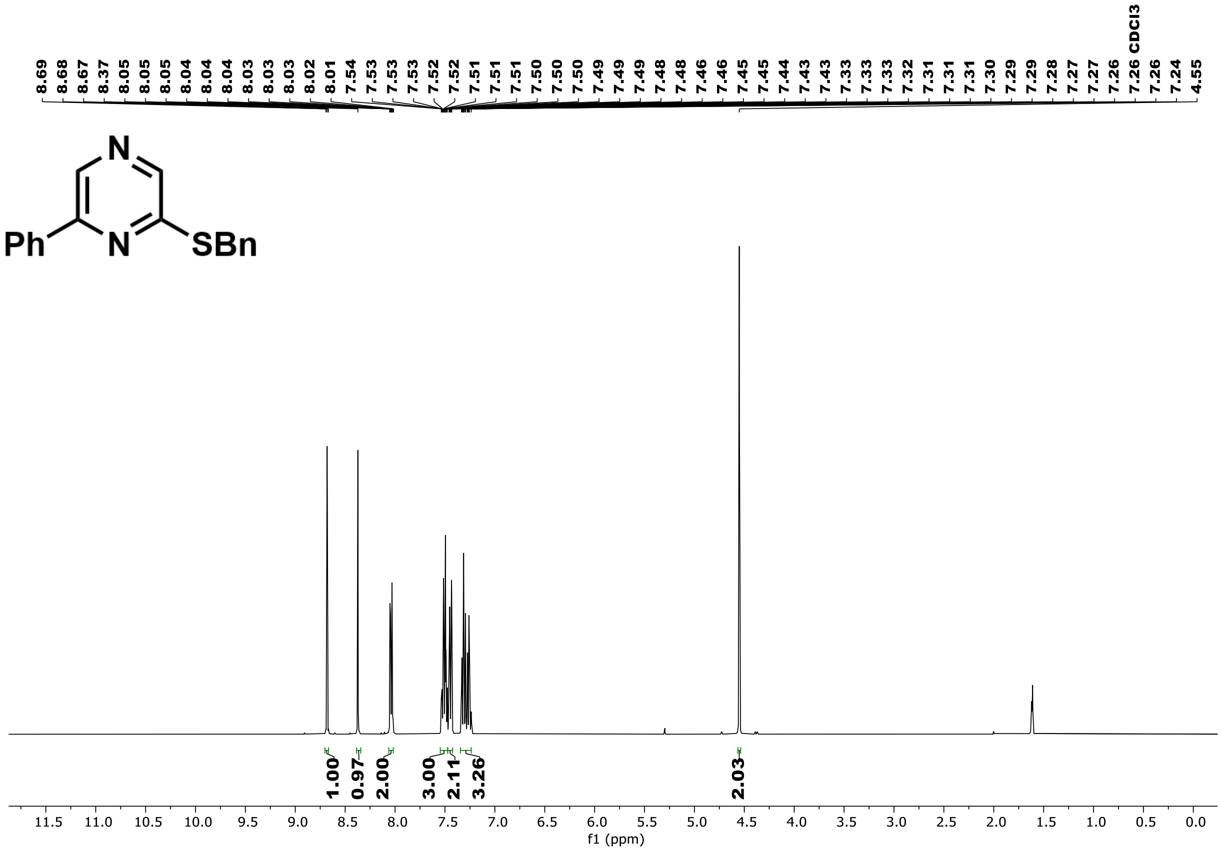
8.69 8.68 8.67 8.37 8.05 8.05 8.04 8.04 8.04 8.03 8.03 8.03 8.02 8.01 7.54 7.53 7.53 7.52 7.52 7.51 7.51 7.50 7.50 7.49 7.49 7.49 7.48 7.48 7.46 7.46 7.45 7.45 7.44 7.43 7.43 7.33 7.33 7.33 7.32 7.31 7.31 7.31 7.30 7.29 7.29 7.28 7.27 7.27 7.26 7.26 CDCl3 7.26 7.24 4.55
Ph
N
N
SBn
1.00
0.97
2.00
3.00
2.11
3.26
2.03
f1 (ppm)

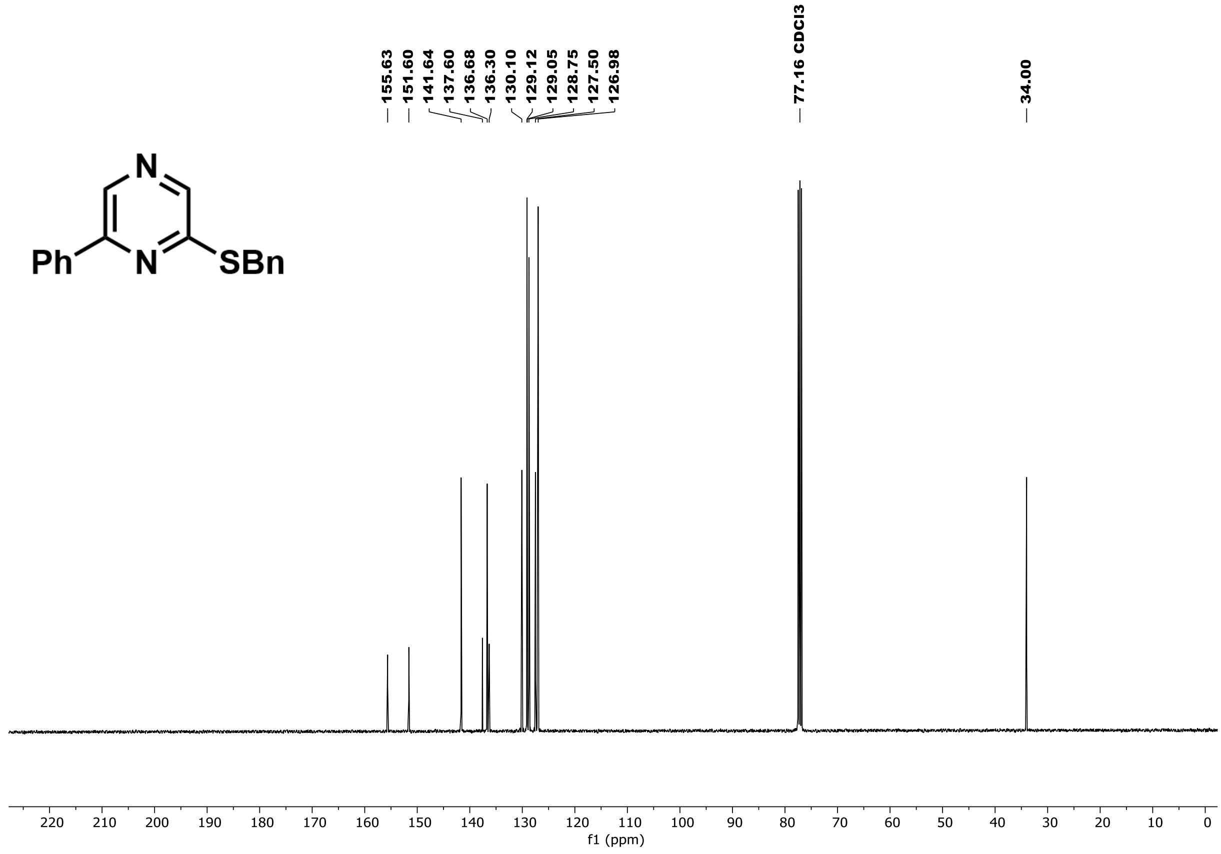
155.63 151.60 141.64 137.60 136.68 136.30 130.10 129.12 129.05 128.75 127.50 126.98
77.16 CDCl3
34.00
Ph
N
N
SBn
f1 (ppm)

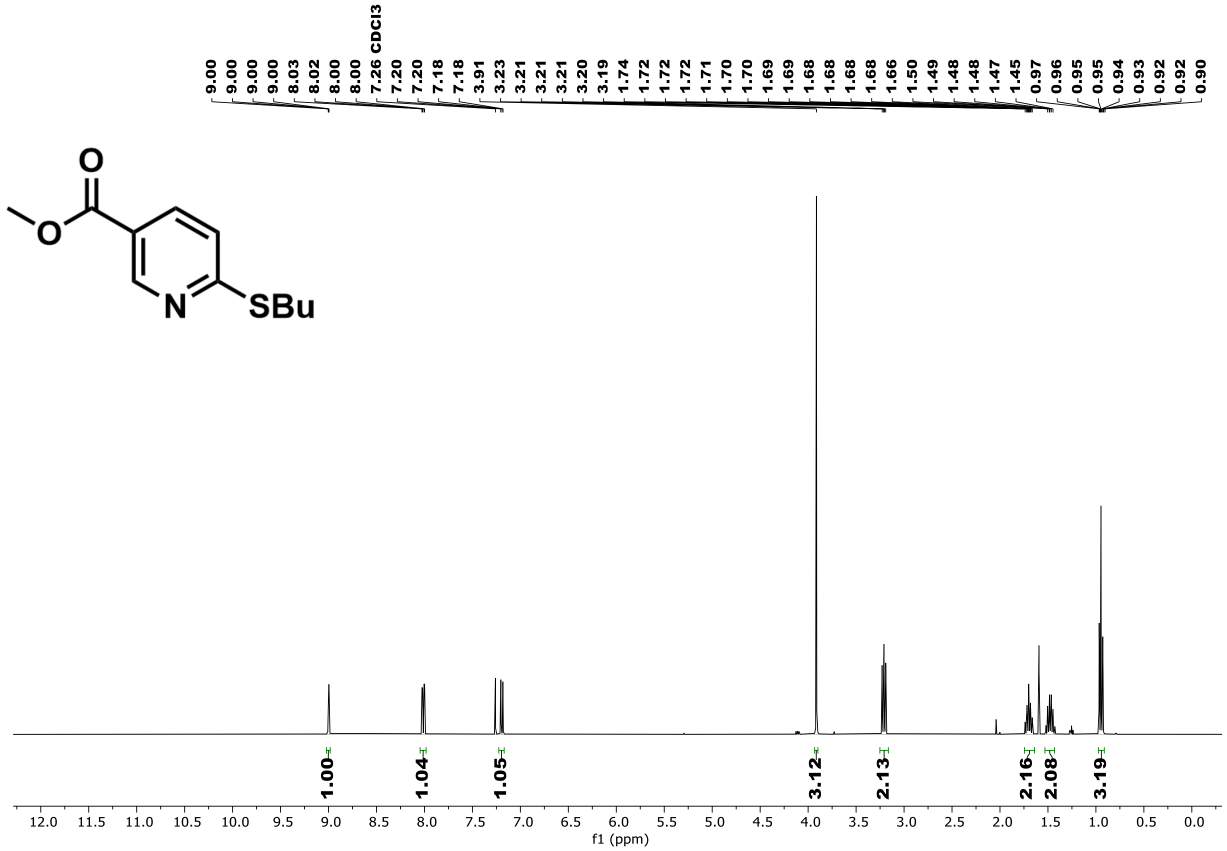
9.00
9.00
9.00
9.00
8.03
8.02
8.00
8.00
7.26 CDCl3
7.20
7.20
7.18
7.18
3.91
3.23
3.21
3.21
3.21
3.20
3.19
1.74
1.72
1.72
1.72
1.71
1.70
1.70
1.69
1.69
1.68
1.68
1.68
1.68
1.66
1.50
1.49
1.48
1.48
1.47
1.45
0.97
0.96
0.95
0.95
0.94
0.93
0.92
0.92
0.90
O
O
N
SBu
1.00
1.04
1.05
3.12
2.13
2.16
2.08
3.19
12.0 11.5 11.0 10.5 10.0 9.5 9.0 8.5 8.0 7.5 7.0 6.5 6.0 5.5 5.0 4.5 4.0 3.5 3.0 2.5 2.0 1.5 1.0 0.5 0.0
f1 (ppm)
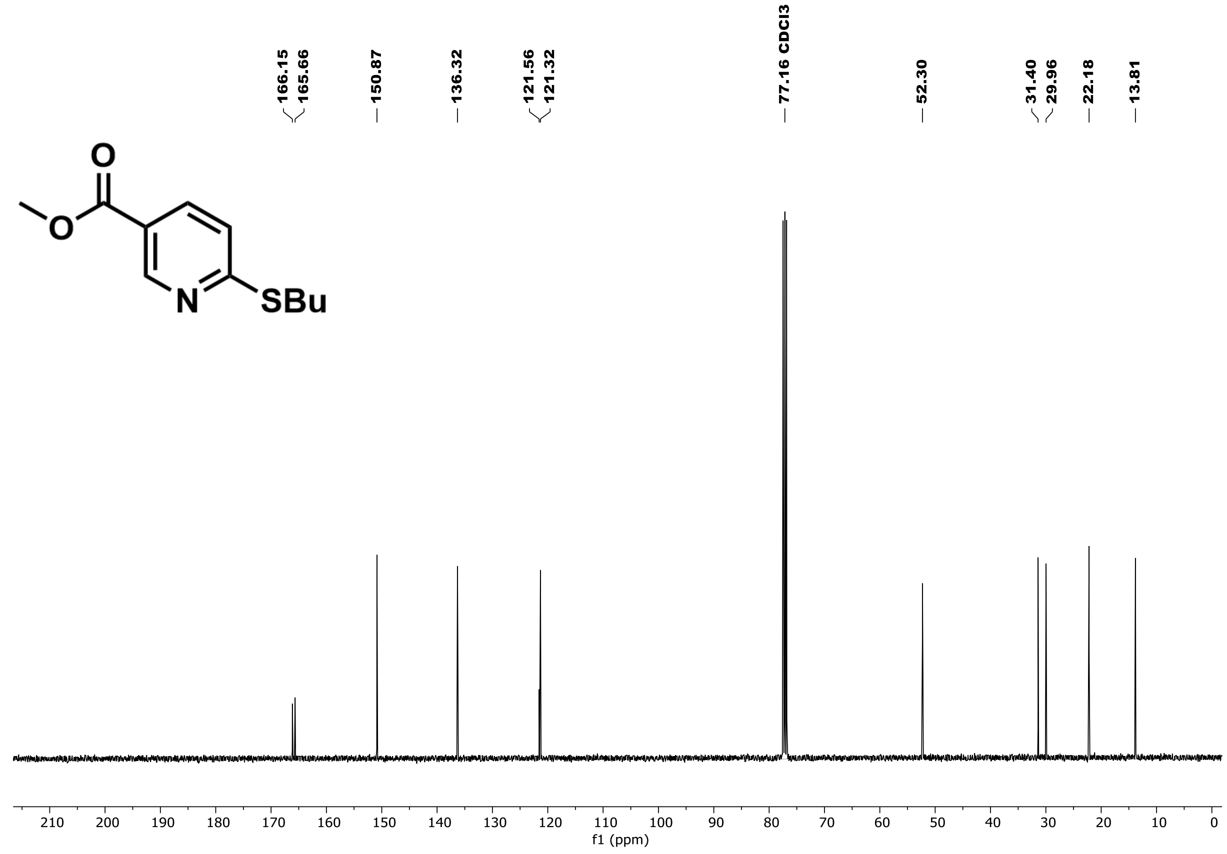
166.15
165.66
150.87
136.32
121.56
121.32
77.16 CDCl3
52.30
31.40
29.96
22.18
13.81
O
O
N
SBu
210 200 190 180 170 160 150 140 130 120 110 100 90 80 70 60 50 40 30 20 10 0
f1 (ppm)

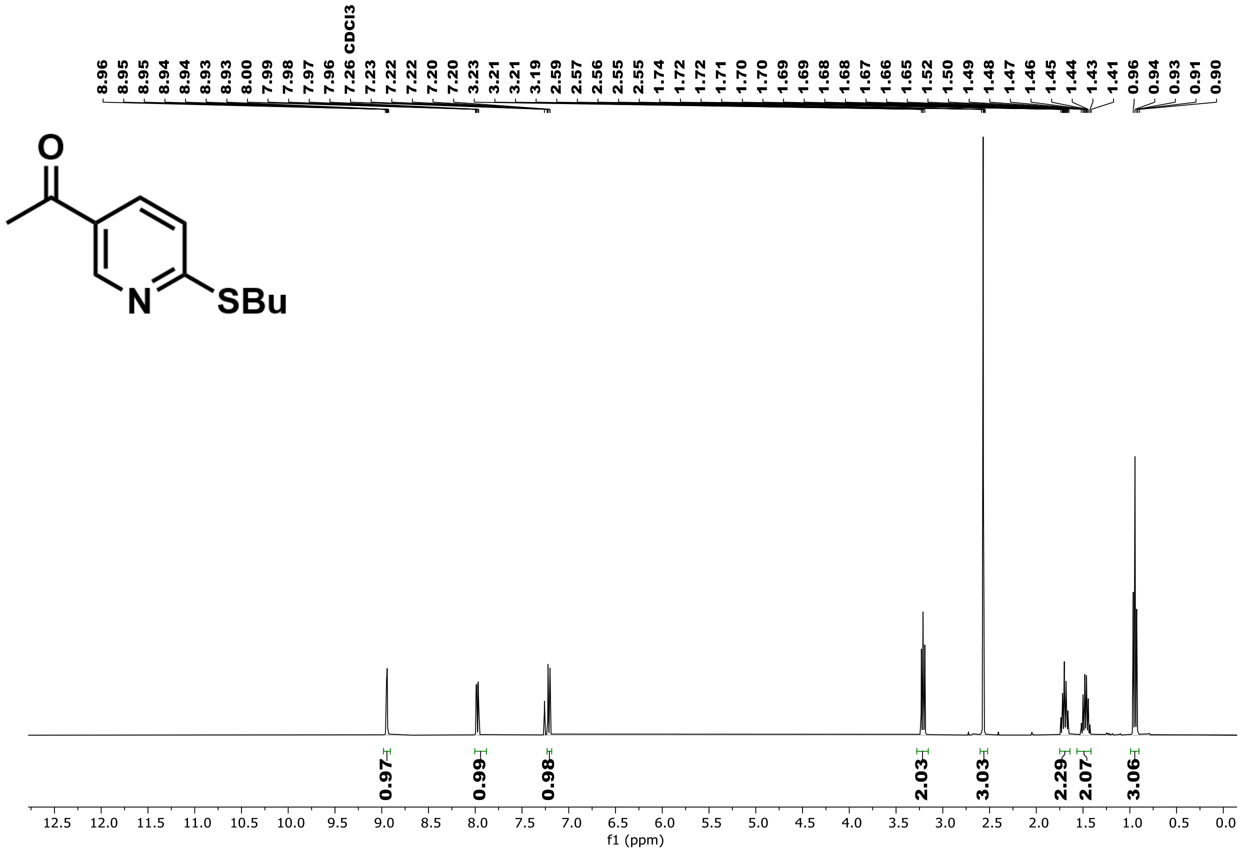
8.96 8.95 8.95 8.94 8.94 8.93 8.93 8.00 7.99 7.98 7.97 7.96 7.26 CDCl3 7.23 7.22 7.22 7.20 7.20 3.23 3.21 3.19 2.59 2.57 2.56 2.55 2.55 1.74 1.72 1.72 1.71 1.70 1.70 1.69 1.69 1.68 1.68 1.67 1.66 1.65 1.52 1.50 1.49 1.48 1.47 1.46 1.45 1.44 1.43 1.41 0.96 0.94 0.93 0.91 0.90
O
N
SBu
0.97
0.99
0.98
2.03
3.03
2.29
2.07
3.06
f1 (ppm)

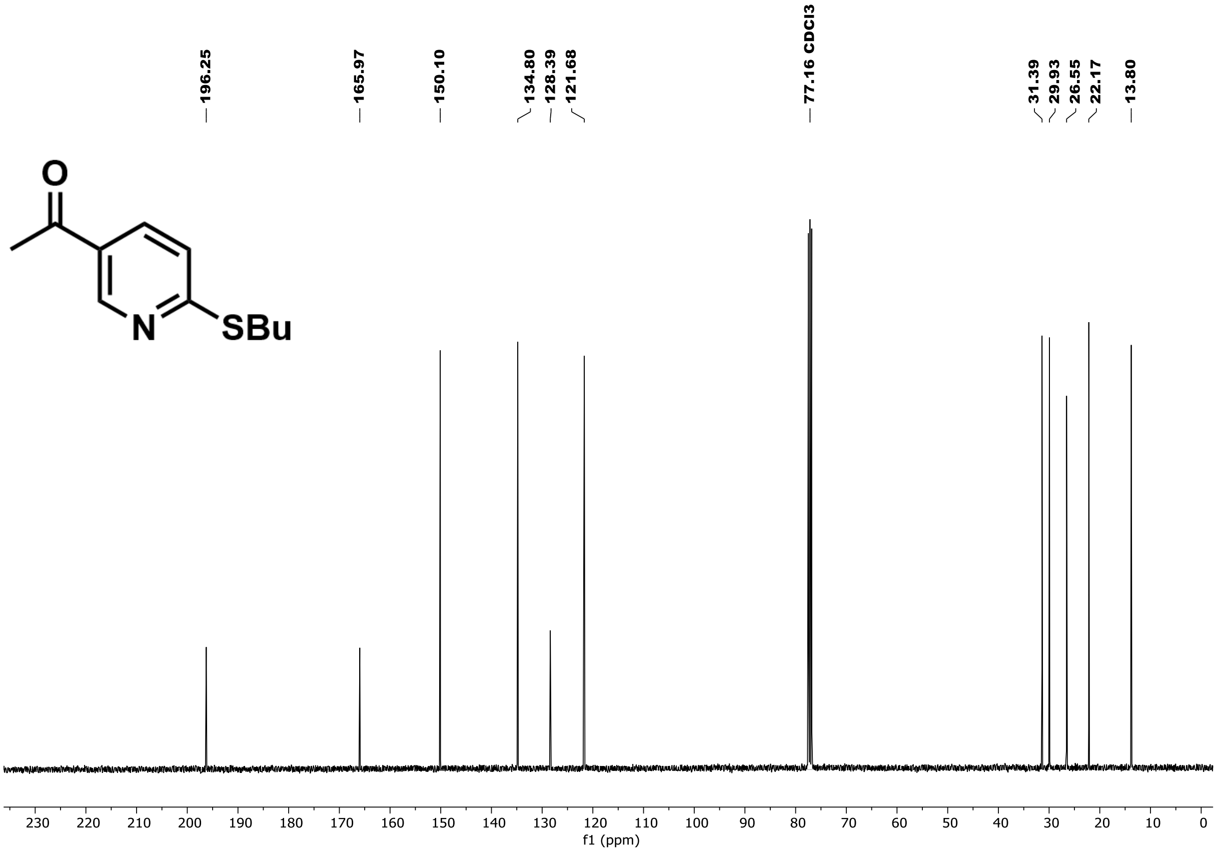
196.25
165.97
150.10
134.80 128.39 121.68
77.16 CDCl3
31.39 29.93 26.55 22.17
13.80
O
N
SBu
f1 (ppm)

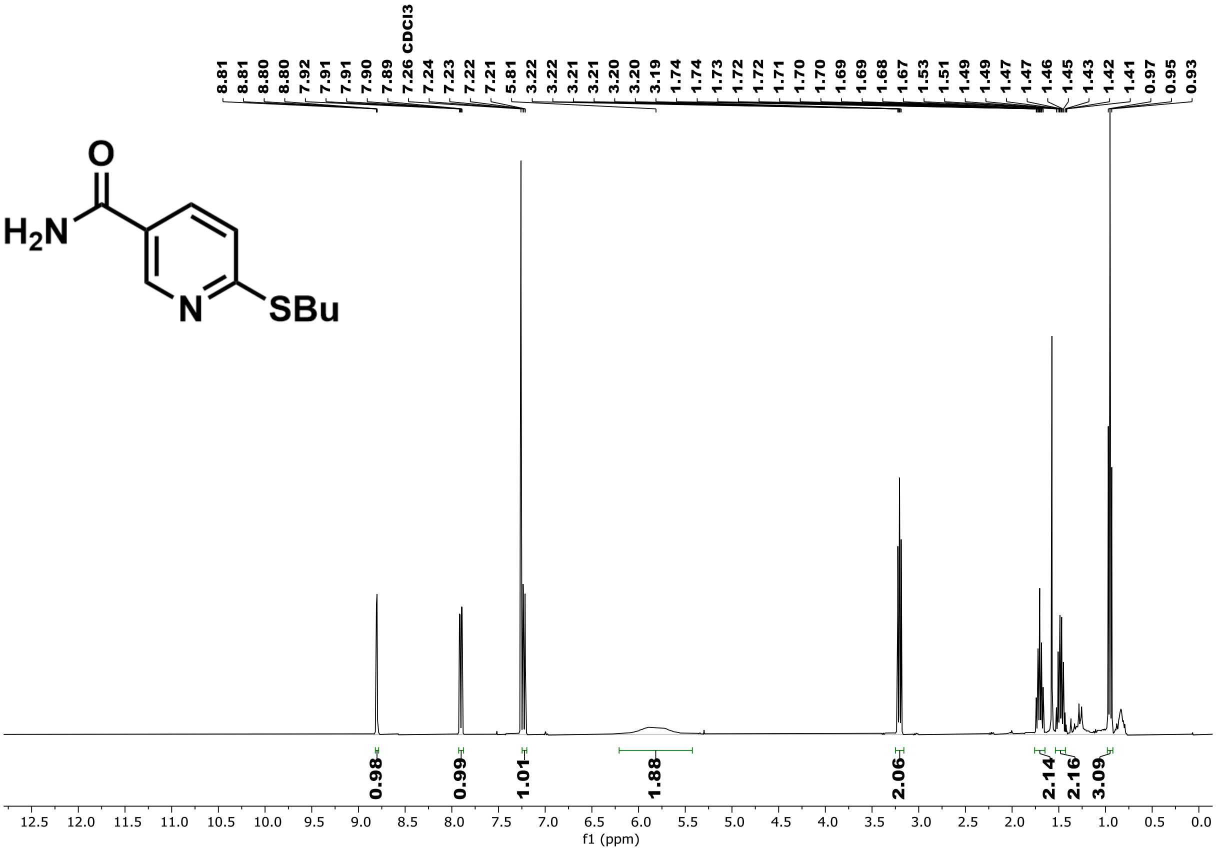

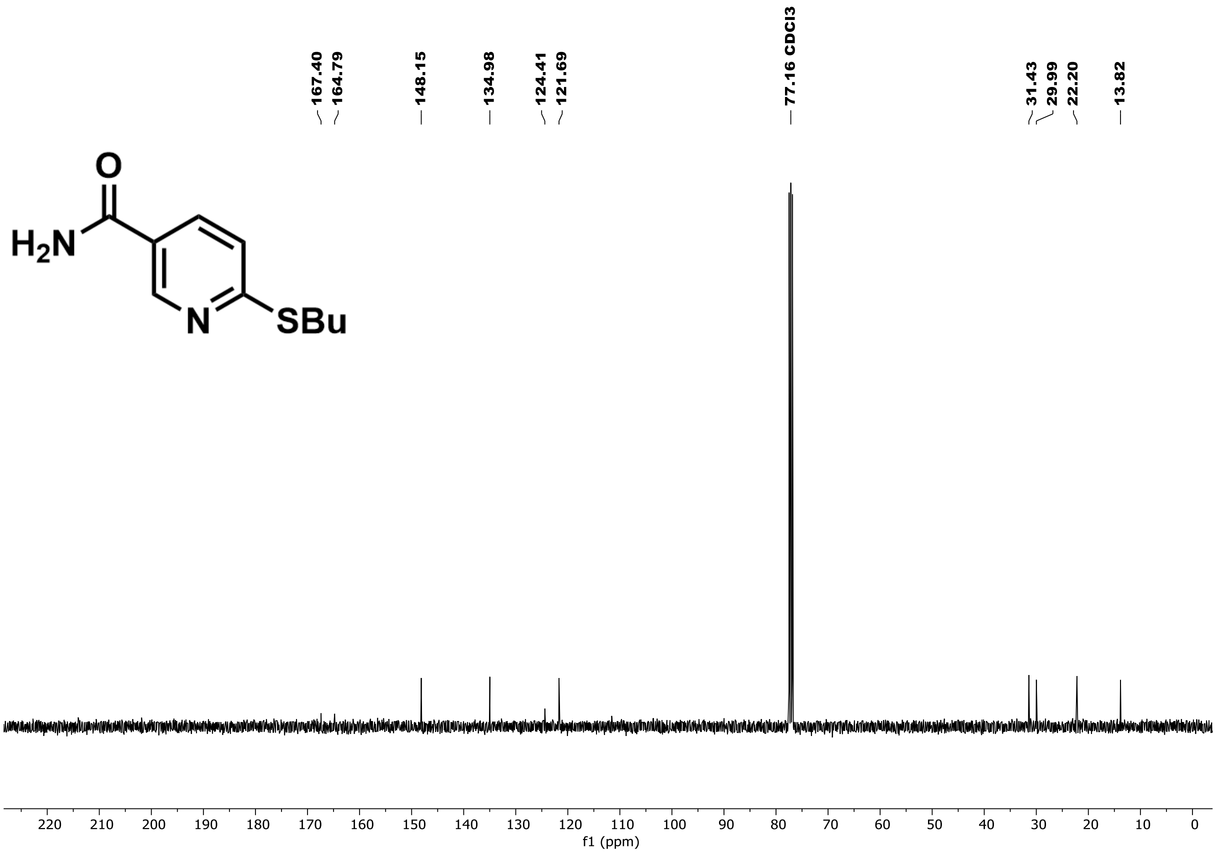

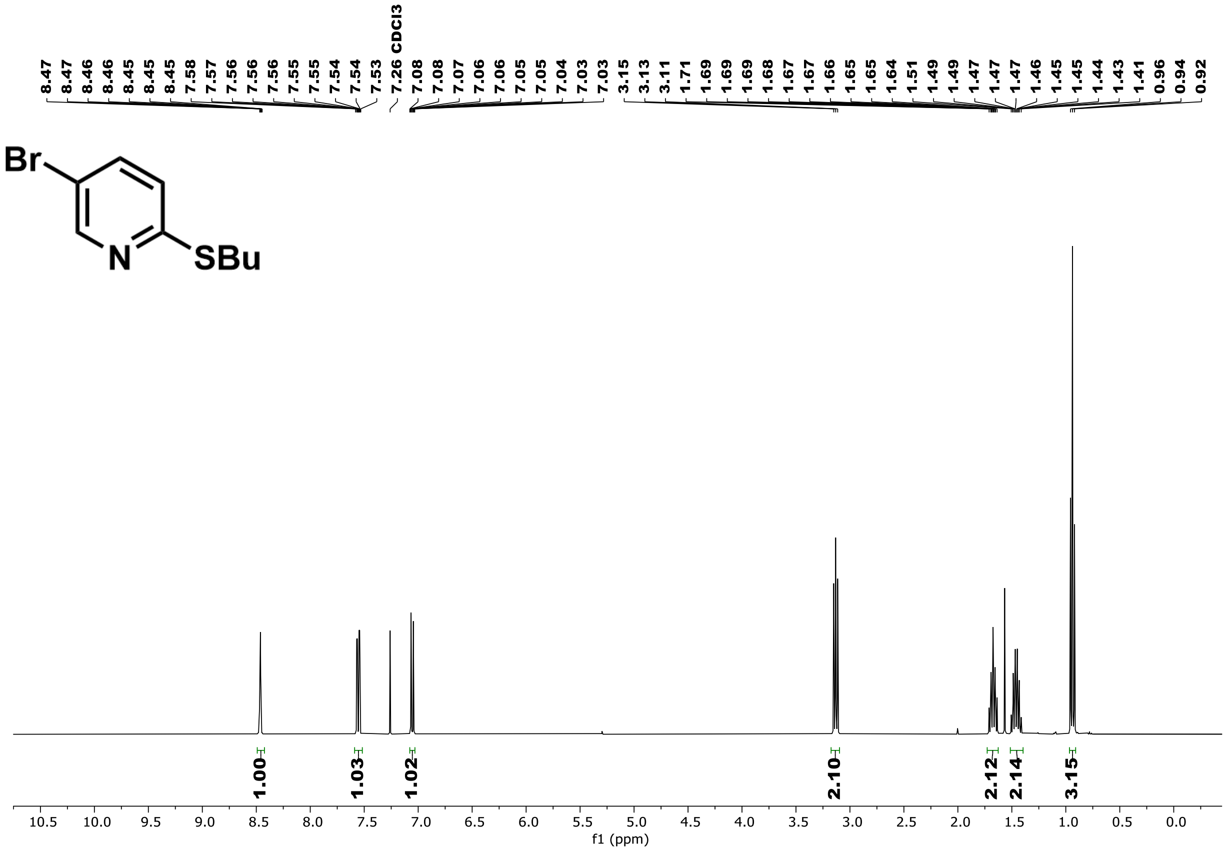
8.47 8.47 8.46 8.46 8.45 8.45 8.45 7.58 7.57 7.56 7.56 7.55 7.55 7.54 7.54 7.53 7.26 CDCl3 7.08 7.08 7.07 7.06 7.06 7.05 7.05 7.04 7.03 7.03 3.15 3.13 3.11 1.71 1.69 1.69 1.68 1.67 1.67 1.66 1.65 1.65 1.64 1.51 1.49 1.49 1.47 1.47 1.47 1.46 1.45 1.45 1.44 1.43 1.41 0.96 0.94 0.92
Br
N
SBu
1.00
1.03
1.02
2.10
2.12
2.14
3.15
10.5 10.0 9.5 9.0 8.5 8.0 7.5 7.0 6.5 6.0 5.5 5.0 4.5 4.0 3.5 3.0 2.5 2.0 1.5 1.0 0.5 0.0
f1 (ppm)

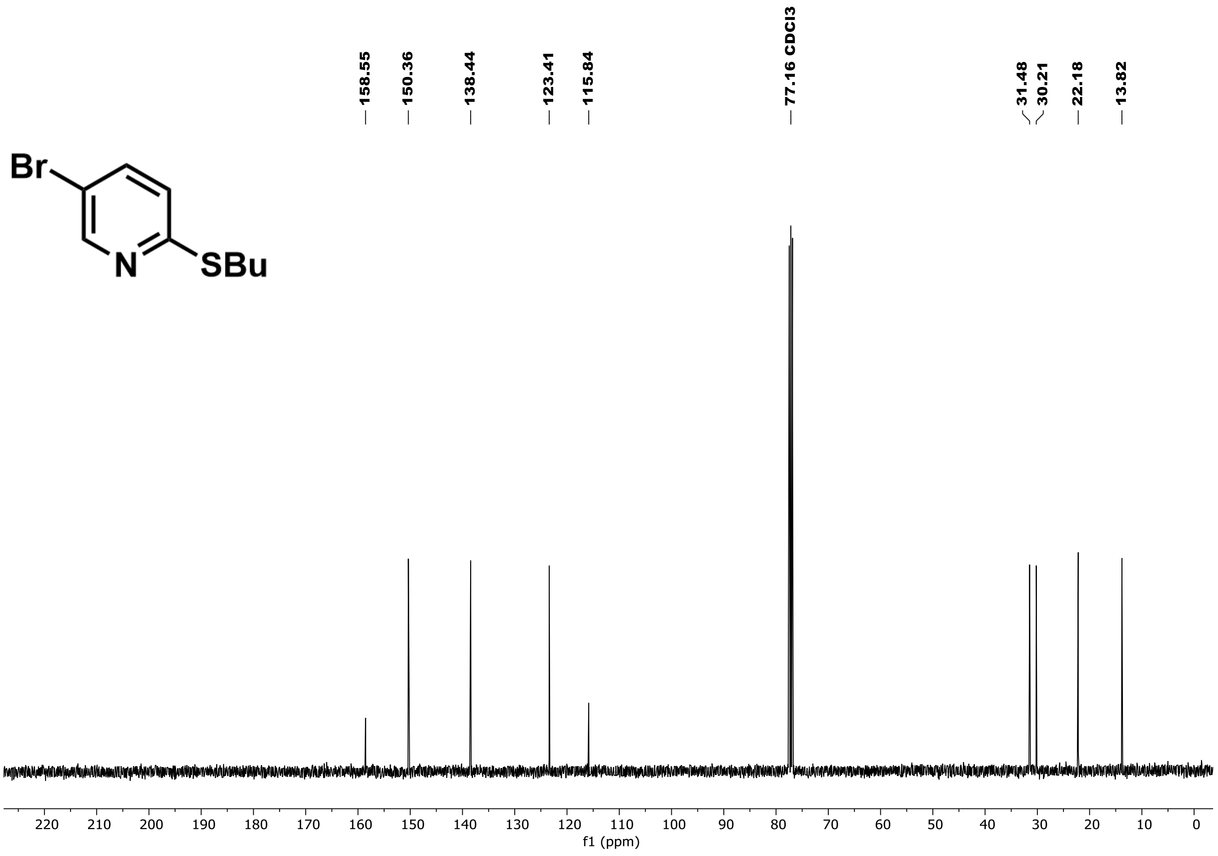
158.55
150.36
138.44
123.41
115.84
77.16 CDCl3
31.48
30.21
22.18
13.82
Br
N
SBu
220 210 200 190 180 170 160 150 140 130 120 110 100 90 80 70 60 50 40 30 20 10 0
f1 (ppm)

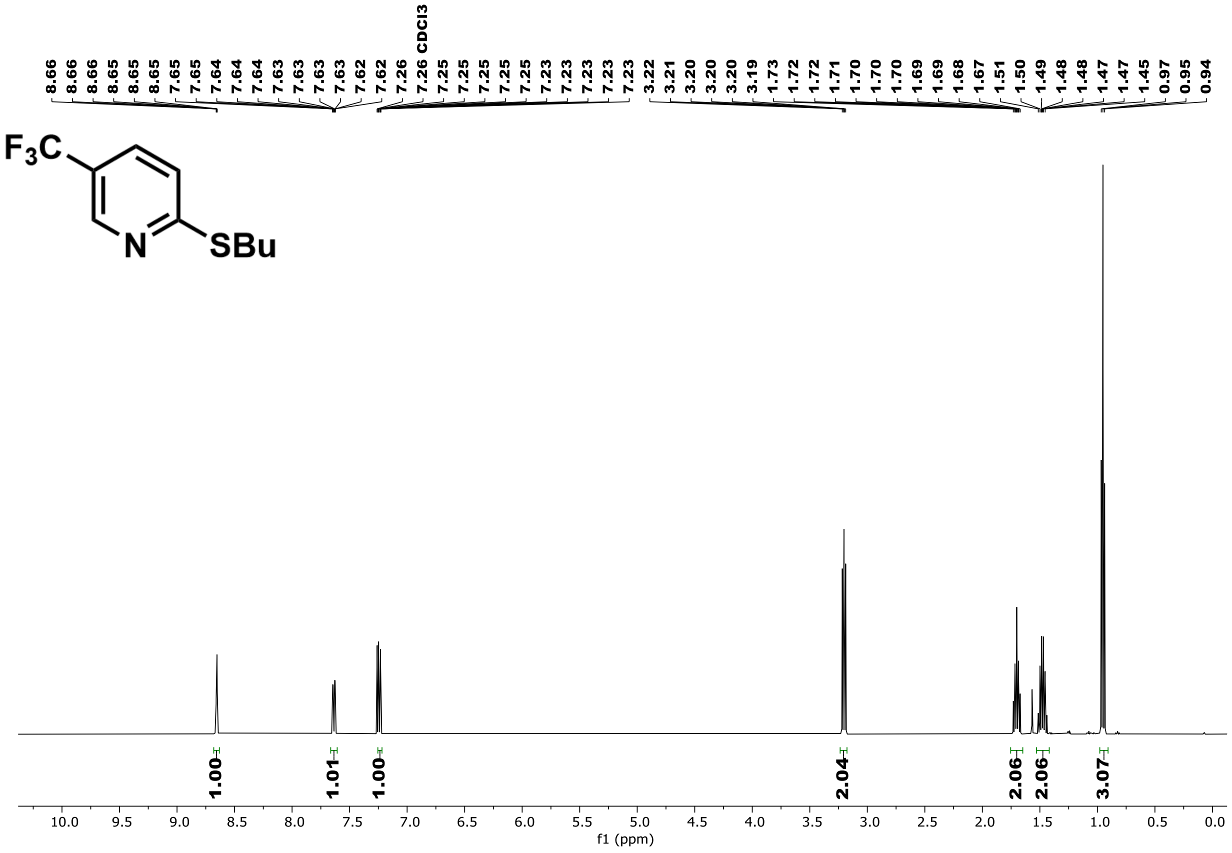
8.66 8.66 8.66 8.65 8.65 8.65 7.65 7.65 7.64 7.64 7.64 7.63 7.63 7.63 7.63 7.62 7.62 7.26 7.26 CDCl3 7.25 7.25 7.25 7.25 7.25 7.23 7.23 7.23 7.23 7.23 3.22 3.21 3.20 3.20 3.19 1.73 1.72 1.72 1.71 1.70 1.70 1.70 1.69 1.69 1.68 1.67 1.51 1.50 1.49 1.48 1.48 1.47 1.47 1.45 0.97 0.95 0.94
F_3C
N
SBu
1.00
1.01
1.00
2.04
2.06
2.06
3.07
10.0 9.5 9.0 8.5 8.0 7.5 7.0 6.5 6.0 5.5 5.0 4.5 4.0 3.5 3.0 2.5 2.0 1.5 1.0 0.5 0.0
f1 (ppm)

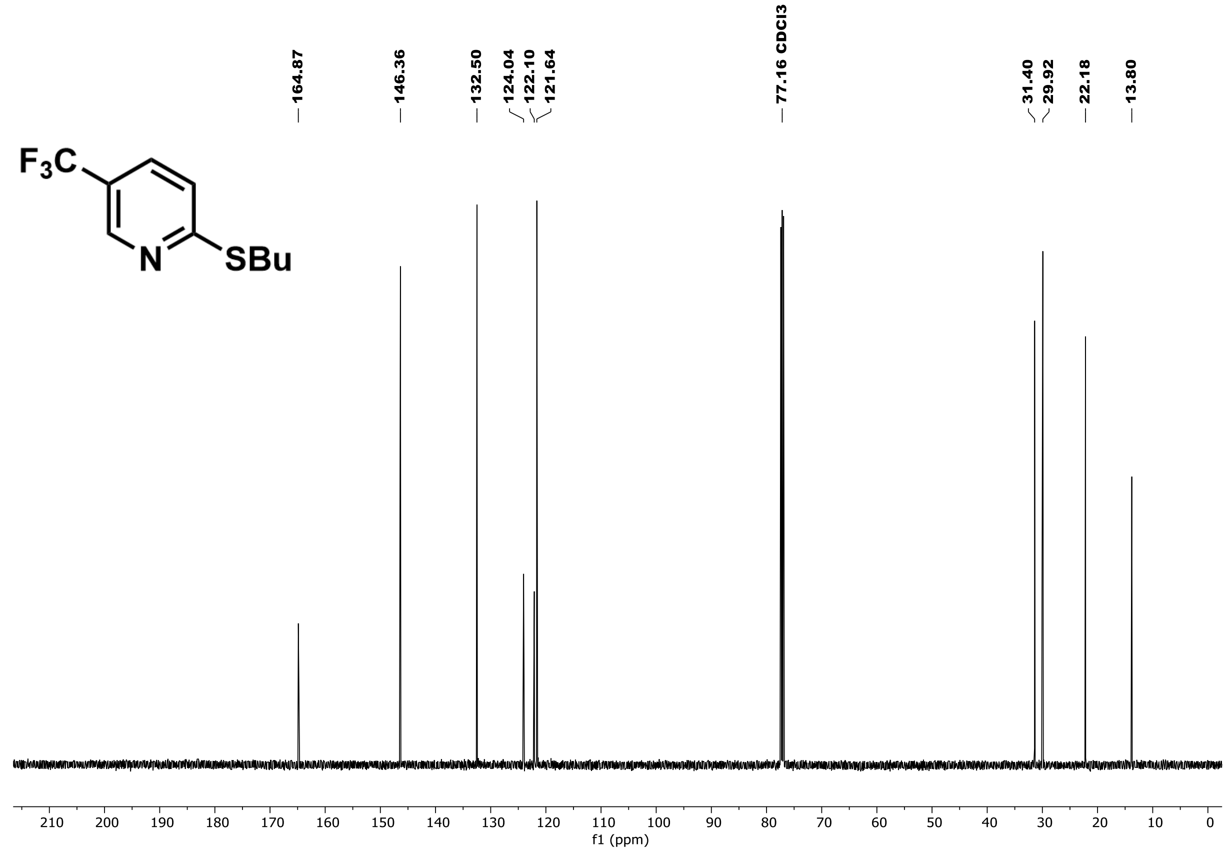
164.87
146.36
132.50
124.04
122.10
121.64
77.16 CDCl3
31.40
29.92
22.18
13.80
F_3C
N
SBu
210 200 190 180 170 160 150 140 130 120 110 100 90 80 70 60 50 40 30 20 10 0
f1 (ppm)

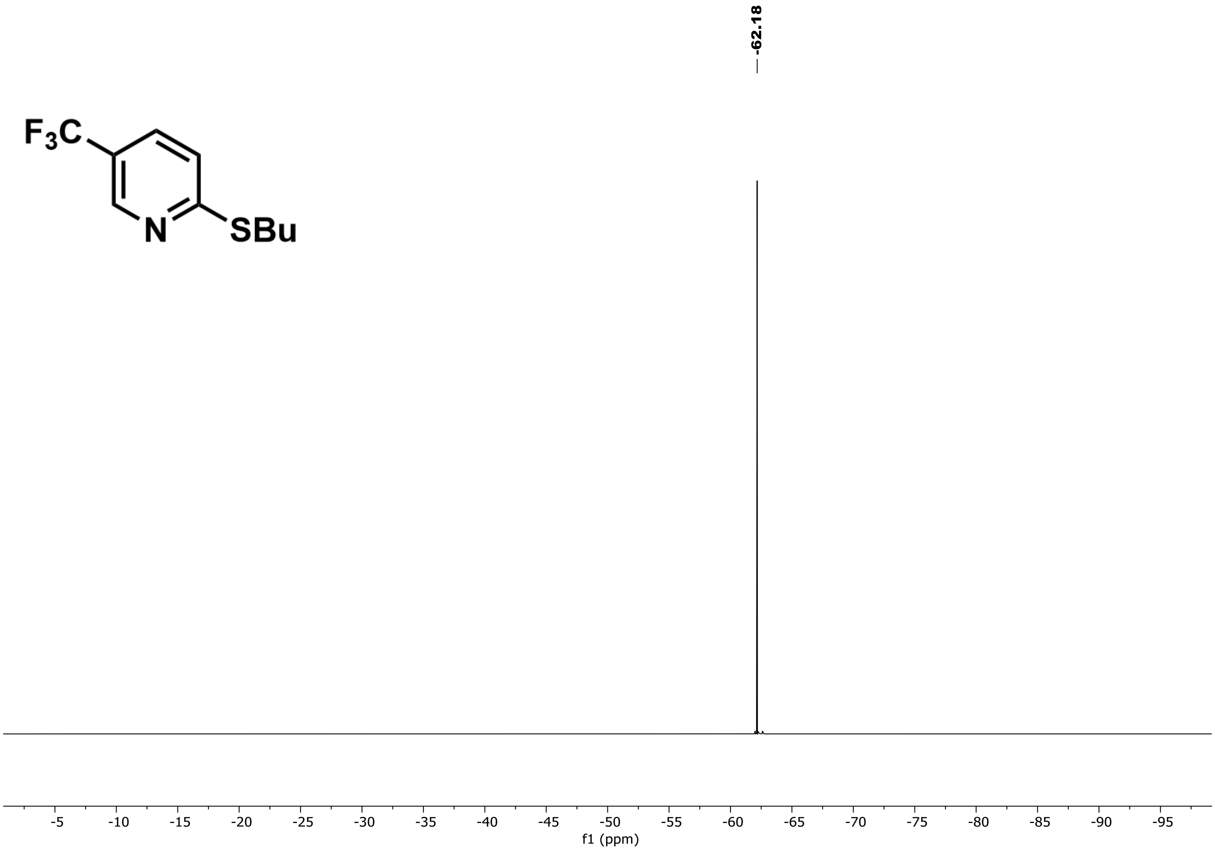

-62.18
F3C
N
SBu
-5 -10 -15 -20 -25 -30 -35 -40 -45 -50 -55 -60 -65 -70 -75 -80 -85 -90 -95
f1 (ppm)

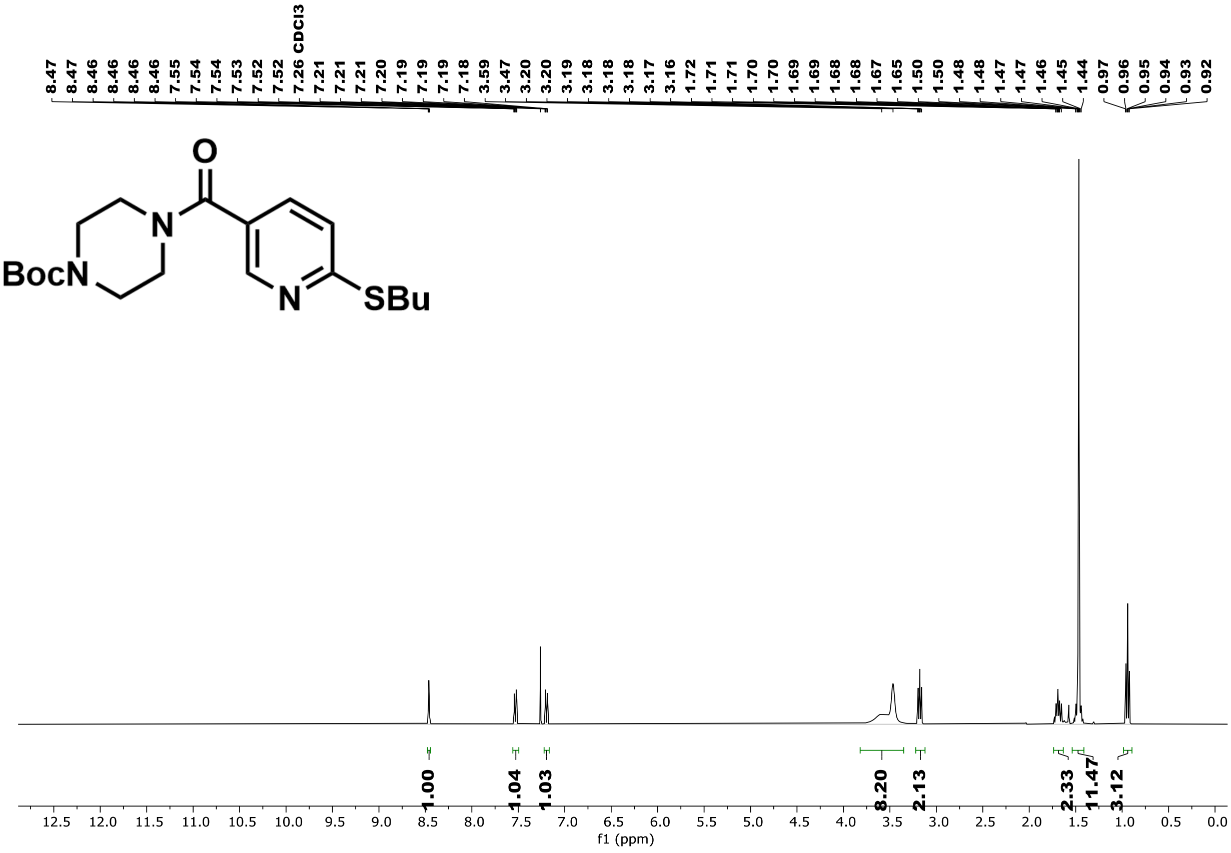

8.47 8.47 8.46 8.46 8.46 7.55 7.54 7.54 7.53 7.52 7.52 7.26 CDCl3 7.21 7.21 7.21 7.20 7.19 7.19 7.18 3.59 3.47 3.20 3.20 3.19 3.18 3.18 3.17 3.16 1.72 1.71 1.71 1.70 1.69 1.68 1.68 1.67 1.65 1.50 1.50 1.48 1.48 1.47 1.46 1.45 1.44 0.97 0.96 0.95 0.94 0.93 0.92
O
BocN
N
N
SBu
1.00
1.04
1.03
8.20
2.13
2.33
11.47
3.12
12.5 12.0 11.5 11.0 10.5 10.0 9.5 9.0 8.5 8.0 7.5 7.0 6.5 6.0 5.5 5.0 4.5 4.0 3.5 3.0 2.5 2.0 1.5 1.0 0.5 0.0
f1 (ppm)

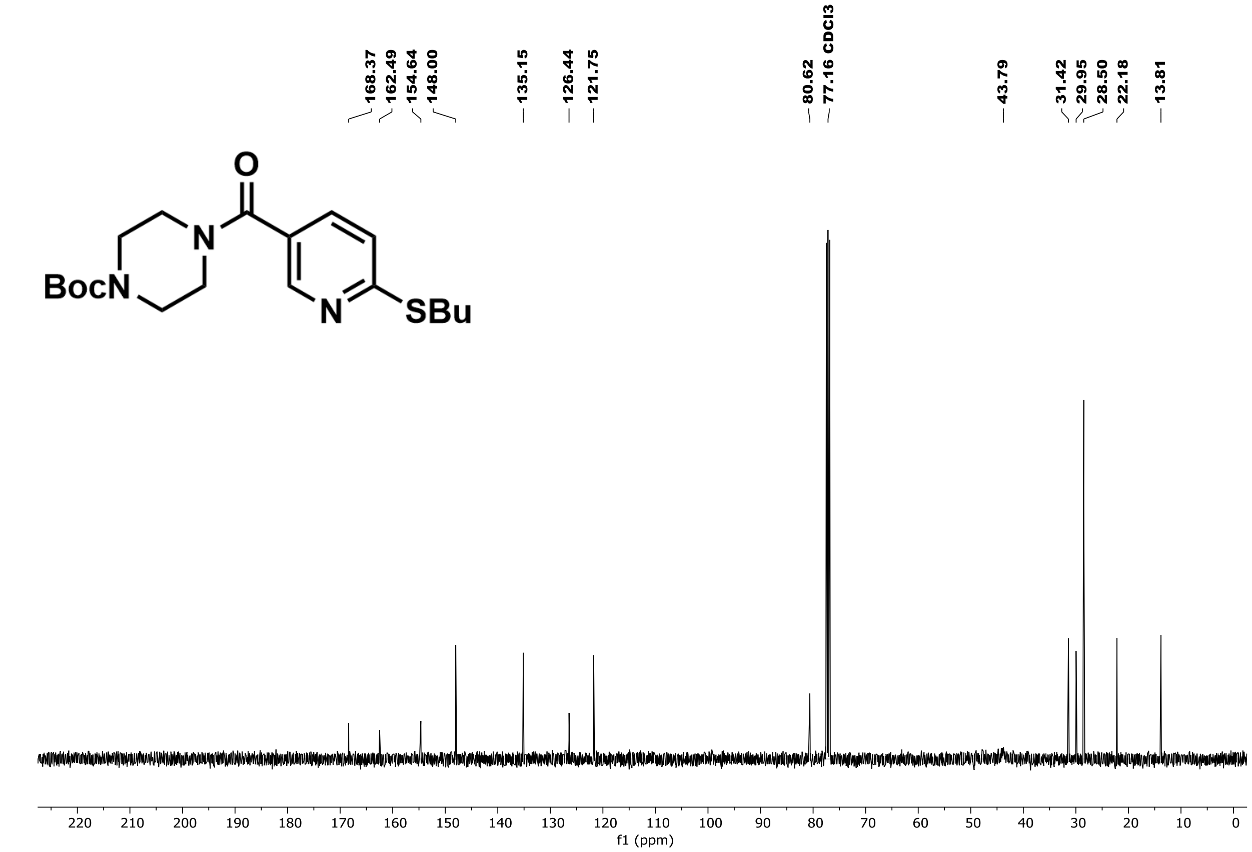
168.37
162.49
154.64
148.00
135.15
126.44
121.75
80.62
77.16 CDCl3
43.79
31.42
29.95
28.50
22.18
13.81
BocN
N
O
N
SBu
220 210 200 190 180 170 160 150 140 130 120 110 100 90 80 70 60 50 40 30 20 10 0
f1 (ppm)

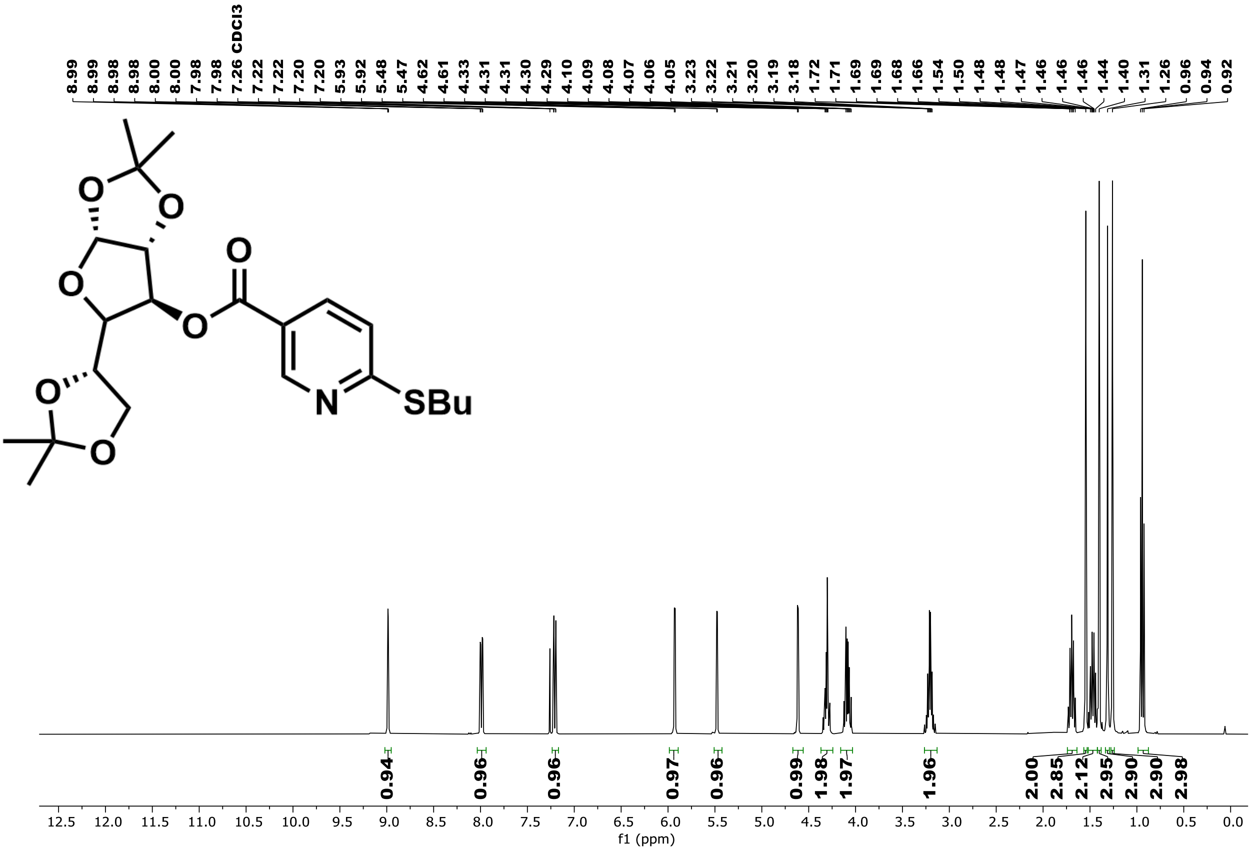
8.99
8.99
8.98
8.98
8.00
8.00
7.98
7.98
7.26 CDCl3
7.22
7.22
7.20
7.20
5.93
5.92
5.48
5.47
4.62
4.61
4.33
4.31
4.31
4.30
4.29
4.10
4.09
4.08
4.07
4.06
4.05
3.23
3.22
3.21
3.20
3.19
3.18
1.72
1.71
1.69
1.69
1.68
1.66
1.54
1.50
1.48
1.48
1.47
1.46
1.46
1.46
1.44
1.40
1.31
1.26
0.96
0.94
0.92
O
O
O
O
O
O
O
N
SBu
0.94
0.96
0.96
0.97
0.96
0.99
1.98
1.97
1.96
2.00
2.85
2.12
2.95
2.90
2.90
2.98
12.5 12.0 11.5 11.0 10.5 10.0 9.5 9.0 8.5 8.0 7.5 7.0 6.5 6.0 5.5 5.0 4.5 4.0 3.5 3.0 2.5 2.0 1.5 1.0 0.5 0.0
f1 (ppm)

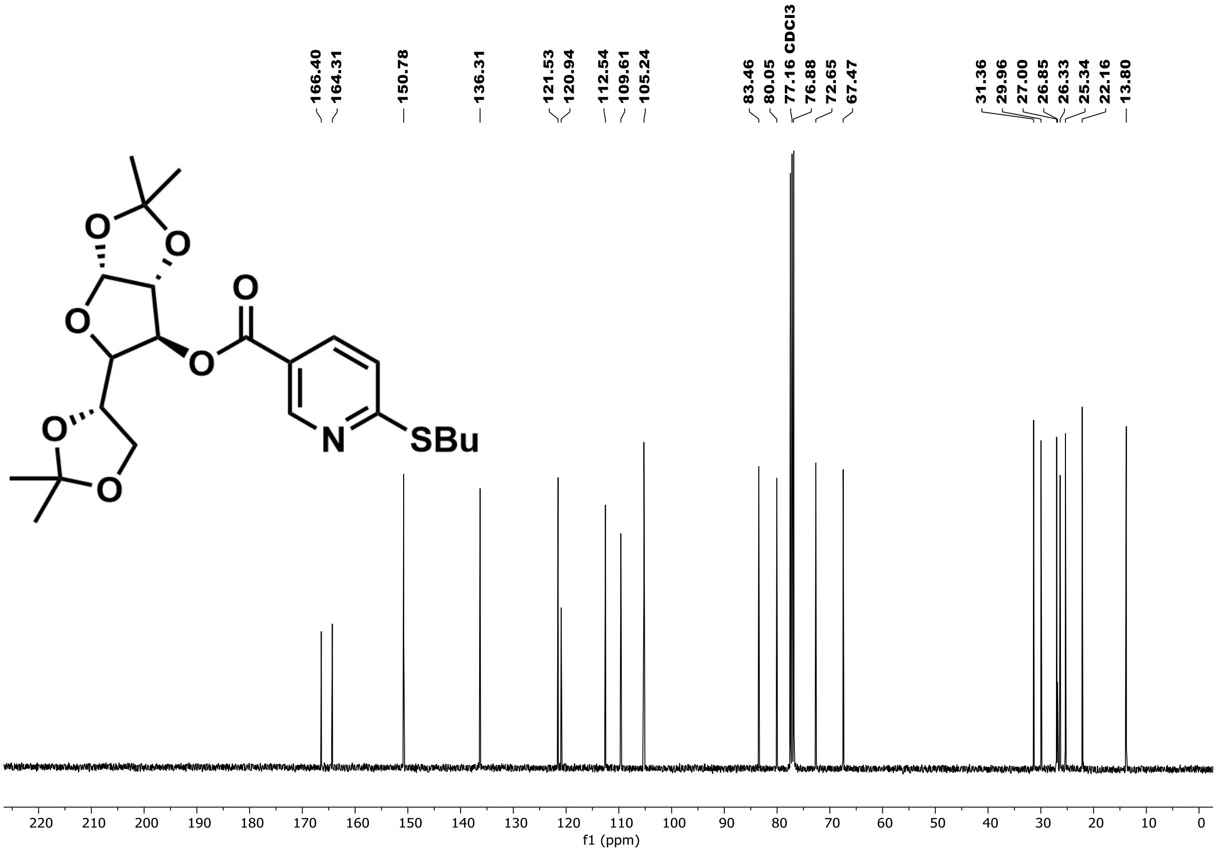
166.40
164.31
150.78
136.31
121.53
120.94
112.54
109.61
105.24
83.46
80.05
77.16 CDCl3
76.88
72.65
67.47
31.36
29.96
27.00
26.85
26.33
25.34
22.16
13.80
O
O
O
O
O
O
O
N
SBu
220 210 200 190 180 170 160 150 140 130 120 110 100 90 80 70 60 50 40 30 20 10 0
f1 (ppm)

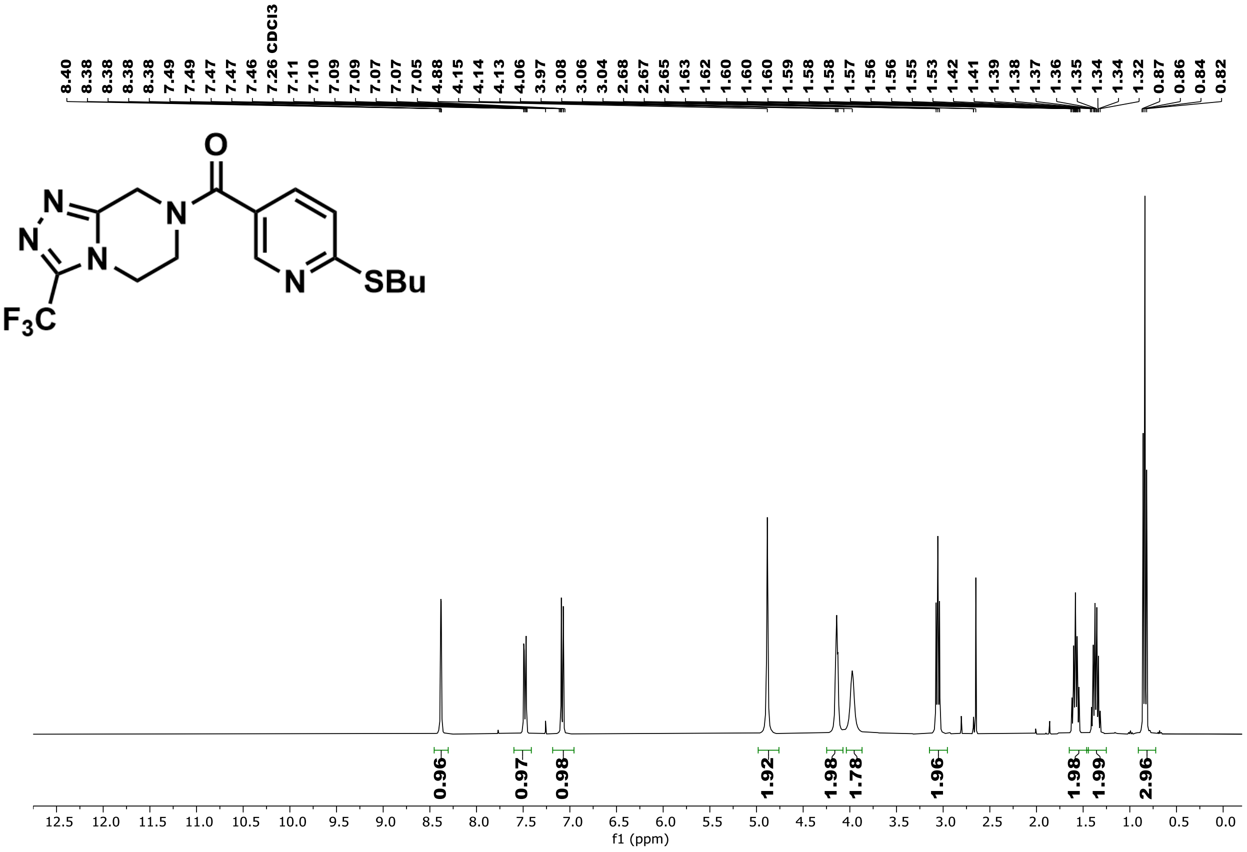
8.40
8.38
8.38
8.38
8.38
7.49
7.49
7.47
7.47
7.46
7.26 CDCl3
7.11
7.10
7.09
7.09
7.07
7.07
7.05
4.88
4.15
4.14
4.13
4.06
3.97
3.08
3.06
3.04
2.68
2.67
2.65
1.63
1.62
1.60
1.60
1.60
1.59
1.58
1.58
1.57
1.56
1.56
1.55
1.53
1.42
1.41
1.39
1.38
1.37
1.36
1.35
1.34
1.34
1.32
0.87
0.86
0.84
0.82
O
N
N
N
N
F3C
N
SBu
0.96
0.97
0.98
1.92
1.98
1.78
1.96
1.98
1.99
2.96
12.5 12.0 11.5 11.0 10.5 10.0 9.5 9.0 8.5 8.0 7.5 7.0 6.5 6.0 5.5 5.0 4.5 4.0 3.5 3.0 2.5 2.0 1.5 1.0 0.5 0.0
f1 (ppm)

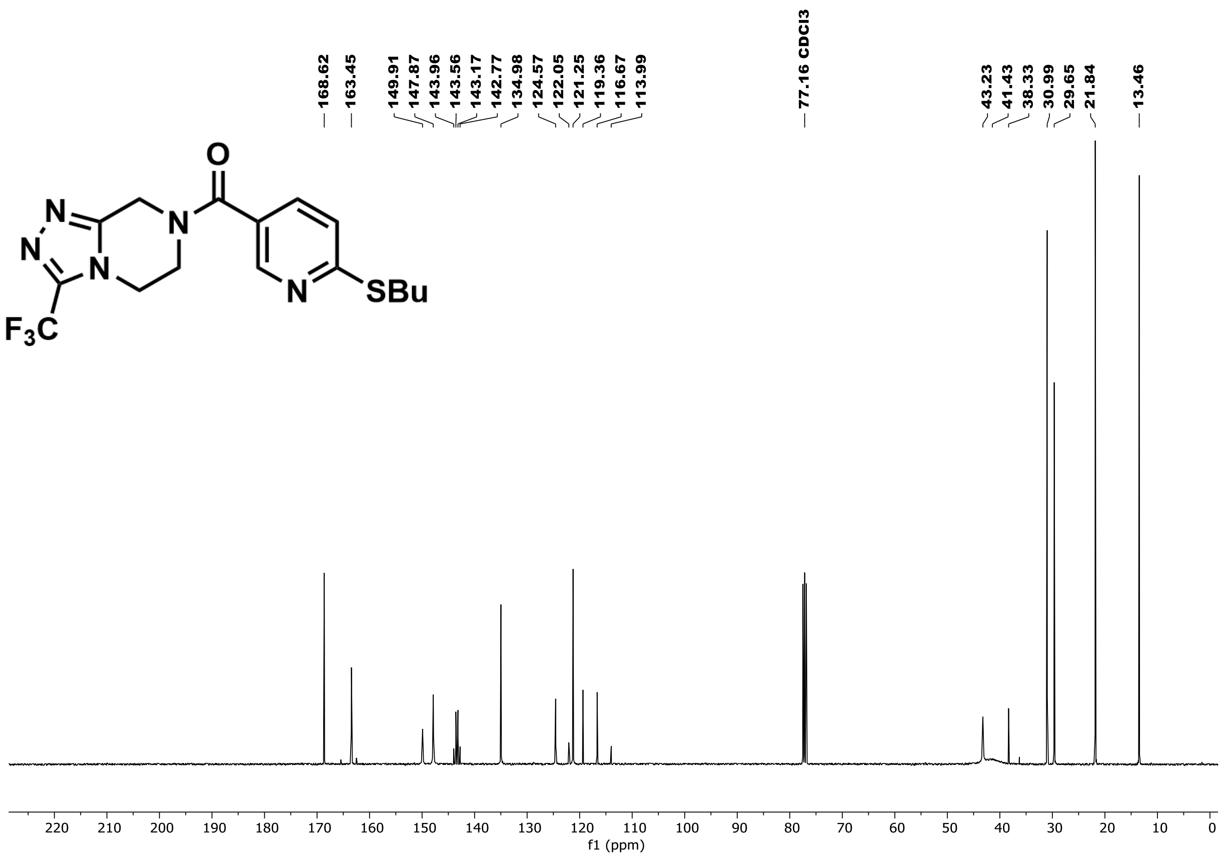
168.62
163.45
149.91
147.87
143.96
143.56
143.17
142.77
134.98
124.57
122.05
121.25
119.36
116.67
113.99
77.16 CDCl3
43.23
41.43
38.33
30.99
29.65
21.84
13.46
f1 (ppm)

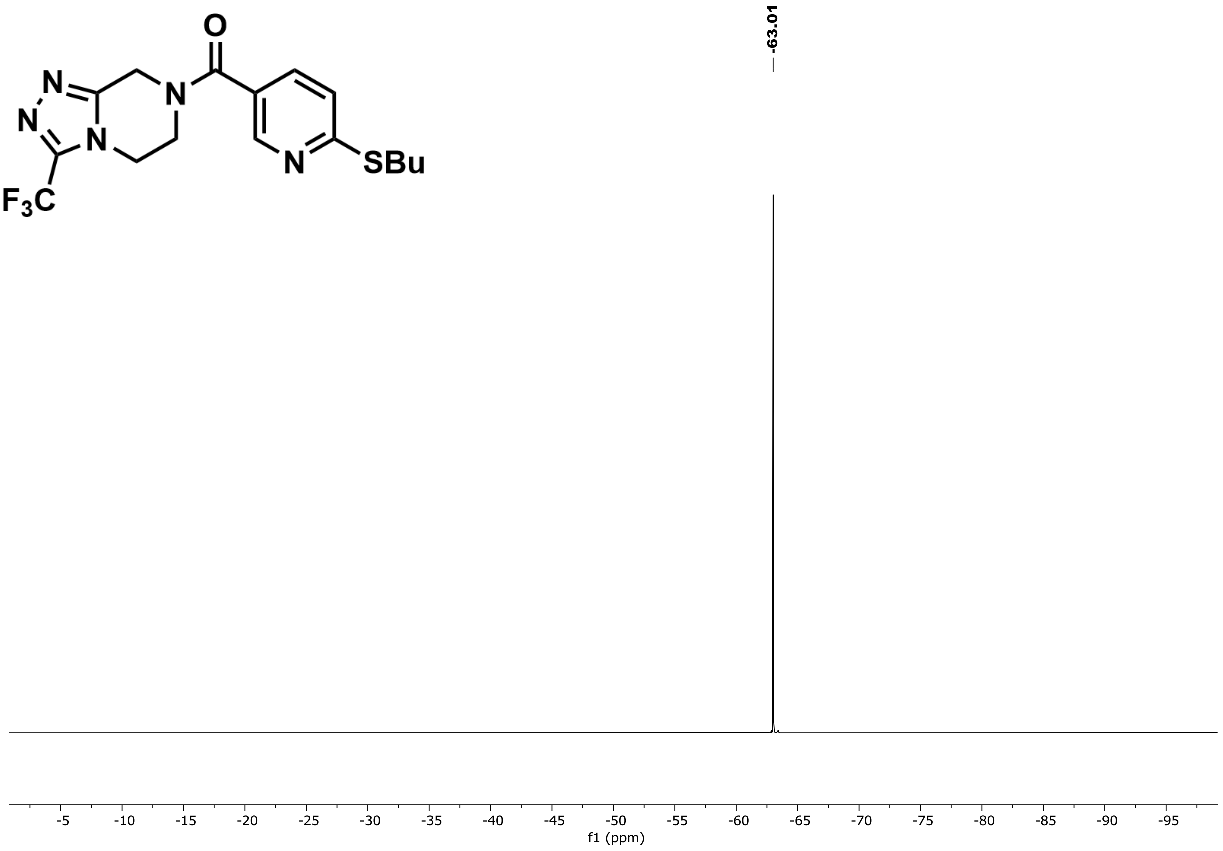
-63.01
f1 (ppm)

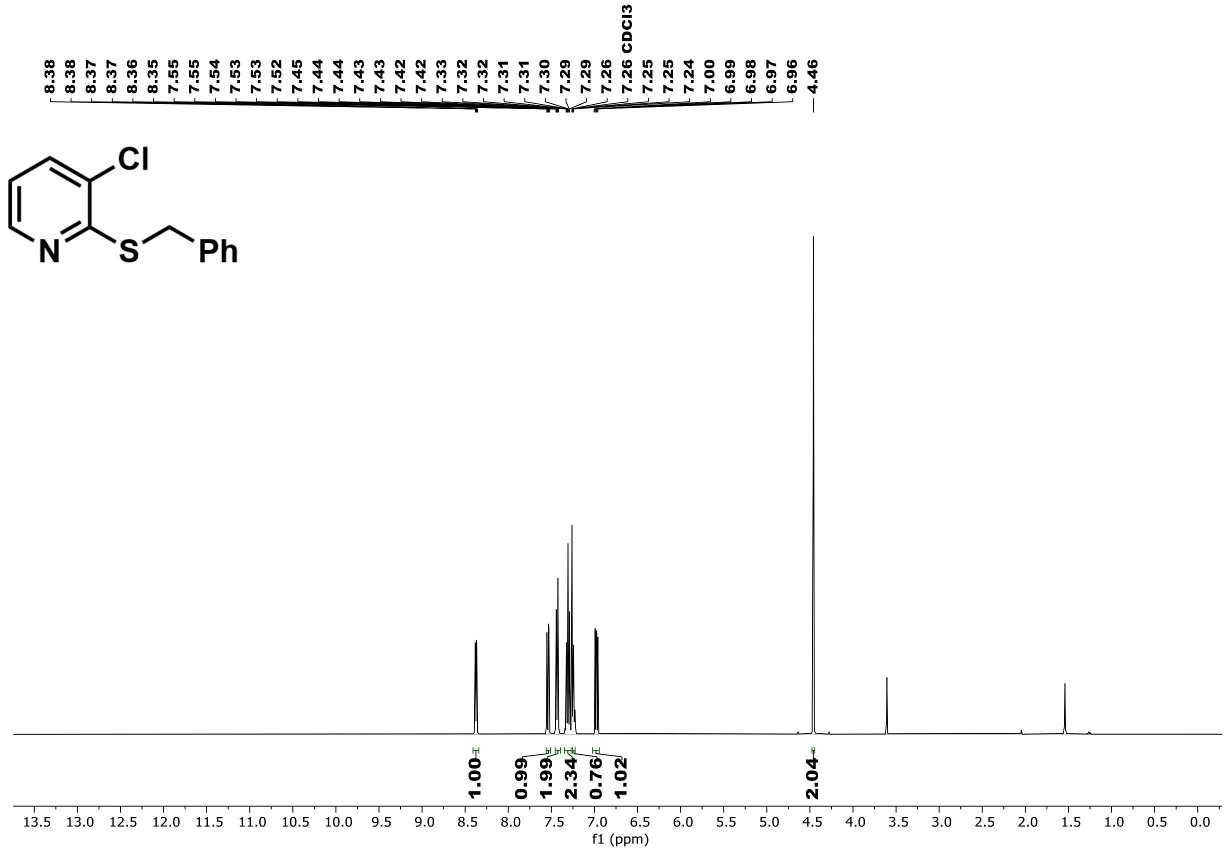
8.38 8.38 8.37 8.37 8.36 8.35 7.55 7.55 7.54 7.53 7.53 7.52 7.45 7.44 7.44 7.43 7.43 7.42 7.42 7.33 7.32 7.32 7.31 7.31 7.30 7.29 7.29 7.26 7.26 CDCl3 7.25 7.25 7.24 7.00 6.99 6.98 6.97 6.96
4.46
Cl
N
S
Ph
1.00
0.99
1.99
2.34
0.76
1.02
2.04
f1 (ppm)

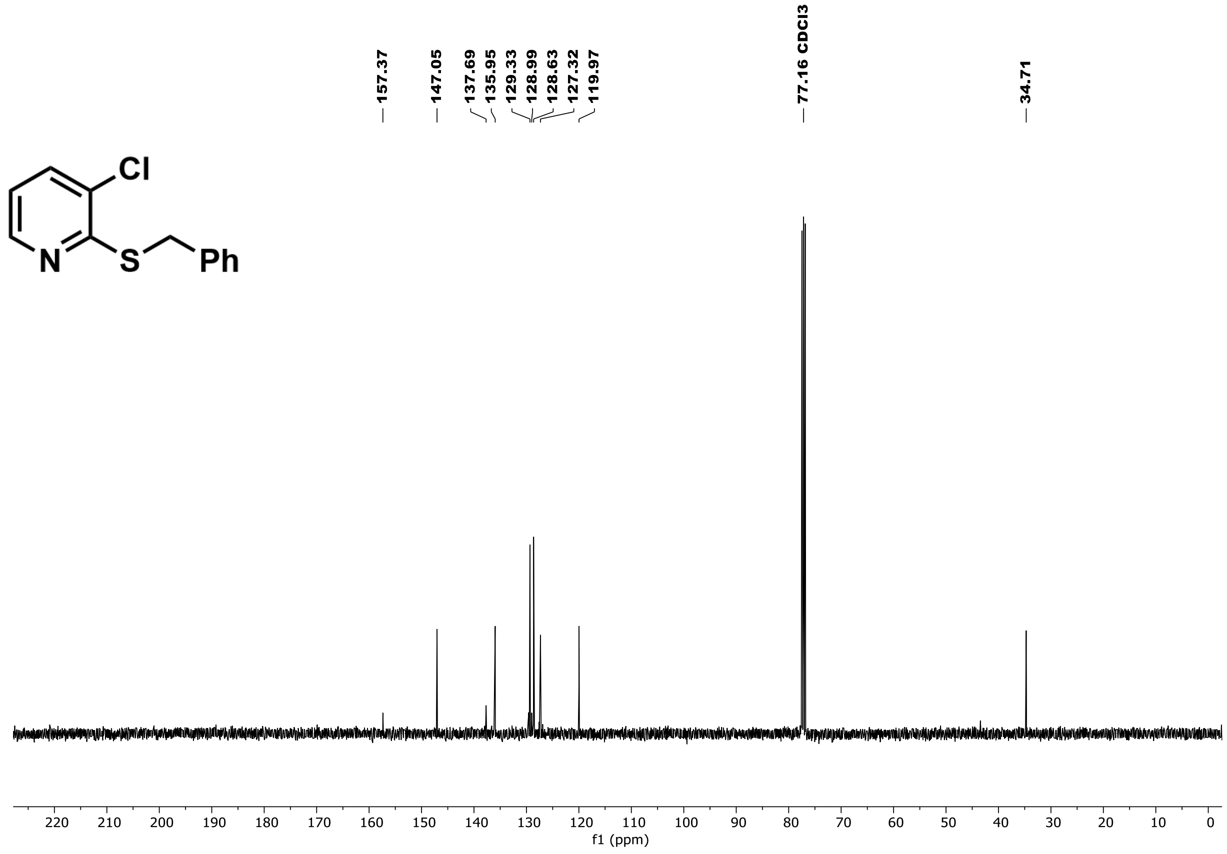
157.37
147.05
137.69 135.95 129.33 128.99 128.63 127.32 119.97
77.16 CDCl3
34.71
Cl
N
S
Ph
f1 (ppm)

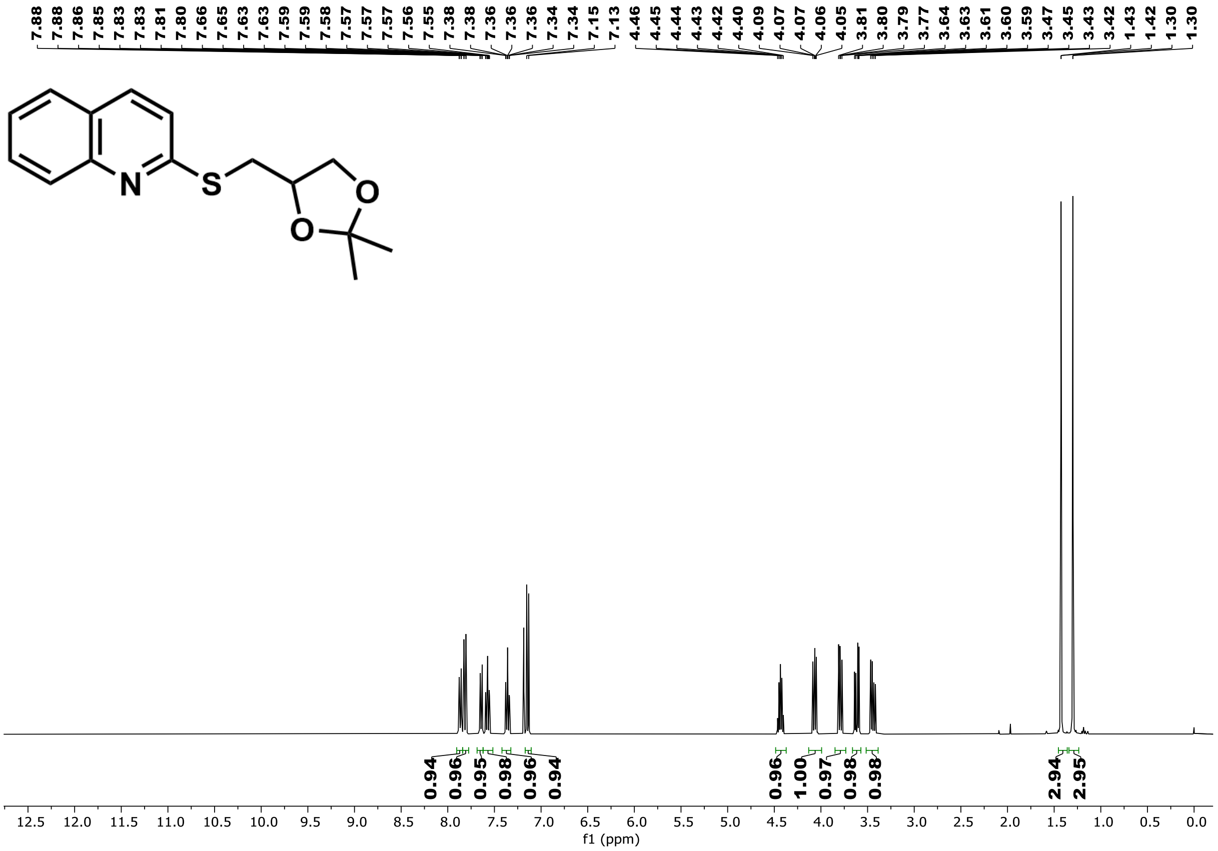
7.88 7.88 7.86 7.85 7.83 7.83 7.81 7.80 7.66 7.65 7.63 7.63 7.59 7.59 7.58 7.57 7.57 7.56 7.55 7.38 7.38 7.36 7.36 7.36 7.34 7.34 7.15 7.13 4.46 4.45 4.44 4.43 4.42 4.40 4.09 4.07 4.07 4.06 4.05 3.81 3.80 3.79 3.77 3.64 3.63 3.61 3.60 3.59 3.47 3.45 3.43 3.42 1.43 1.42 1.30 1.30
N
S
O
O
0.94 0.96 0.95 0.98 0.96 0.94
0.96 1.00 0.97 0.98 0.98
2.94 2.95
12.5 12.0 11.5 11.0 10.5 10.0 9.5 9.0 8.5 8.0 7.5 7.0 6.5 6.0 5.5 5.0 4.5 4.0 3.5 3.0 2.5 2.0 1.5 1.0 0.5 0.0
f1 (ppm)

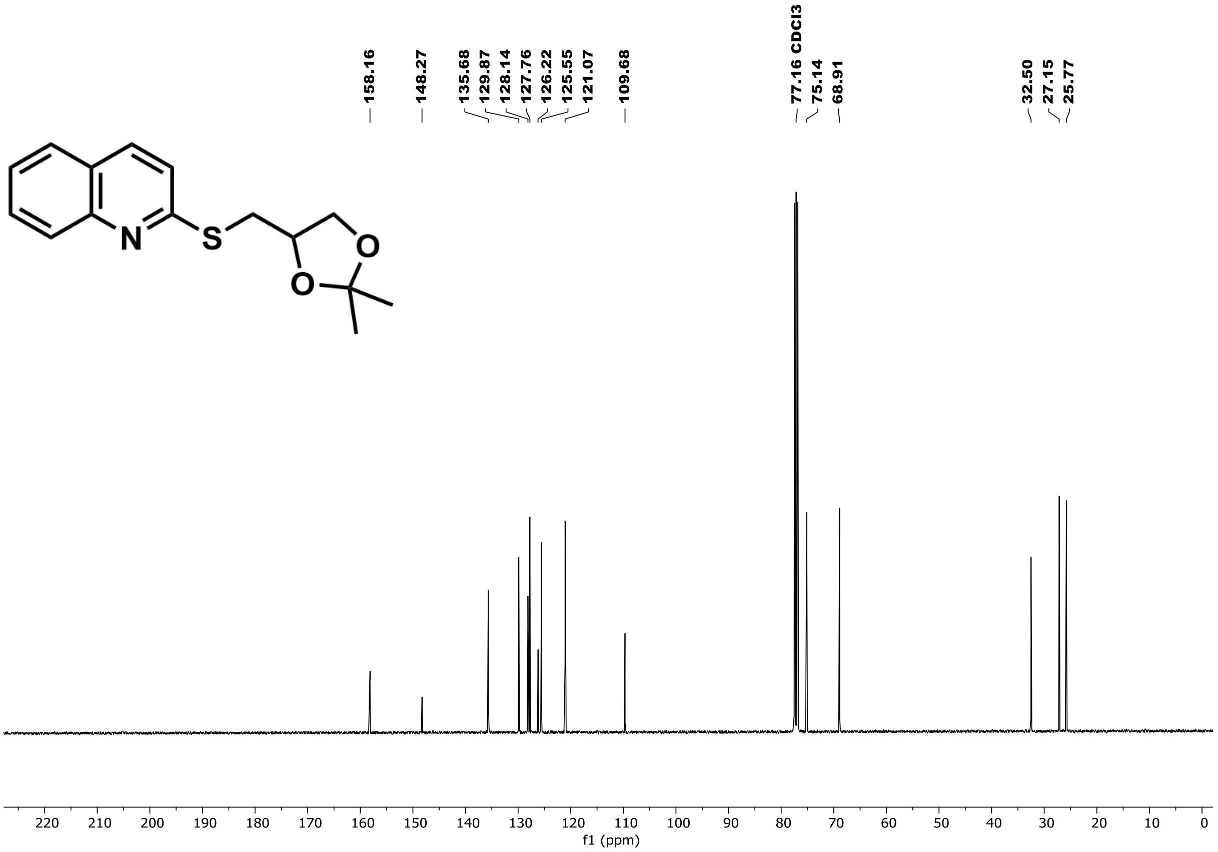
158.16 148.27 135.68 129.87 128.14 127.76 126.22 125.55 121.07 109.68 77.16 CDCl3 75.14 68.91 32.50 27.15 25.77
N
S
O
O
220 210 200 190 180 170 160 150 140 130 120 110 100 90 80 70 60 50 40 30 20 10 0
f1 (ppm)

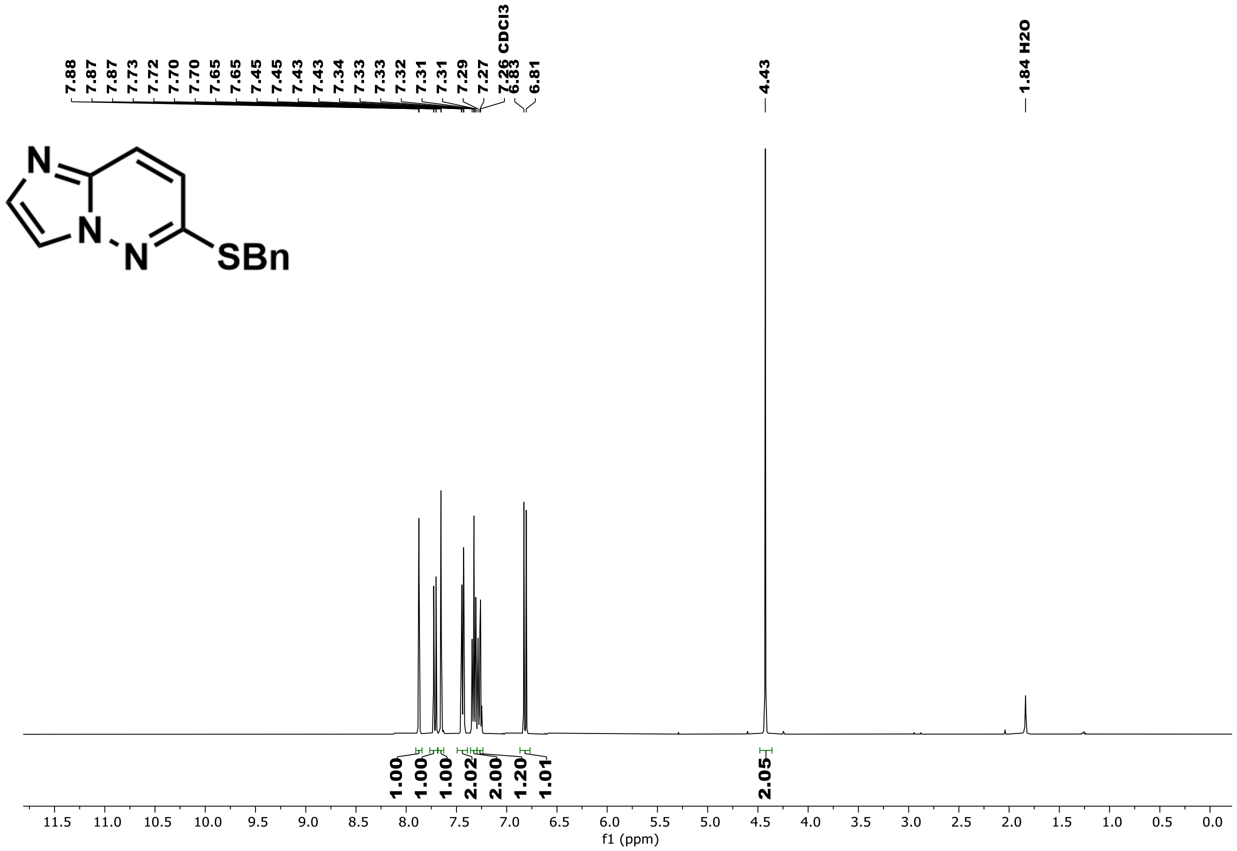
7.88
7.87
7.87
7.73
7.72
7.70
7.70
7.65
7.65
7.45
7.45
7.43
7.43
7.34
7.33
7.33
7.32
7.31
7.31
7.29
7.27
7.26 CDCl3
6.83
6.81
4.43
1.84 H2O
N
N
N
SBn
1.00
1.00
1.00
2.02
2.00
1.20
1.01
2.05
f1 (ppm)

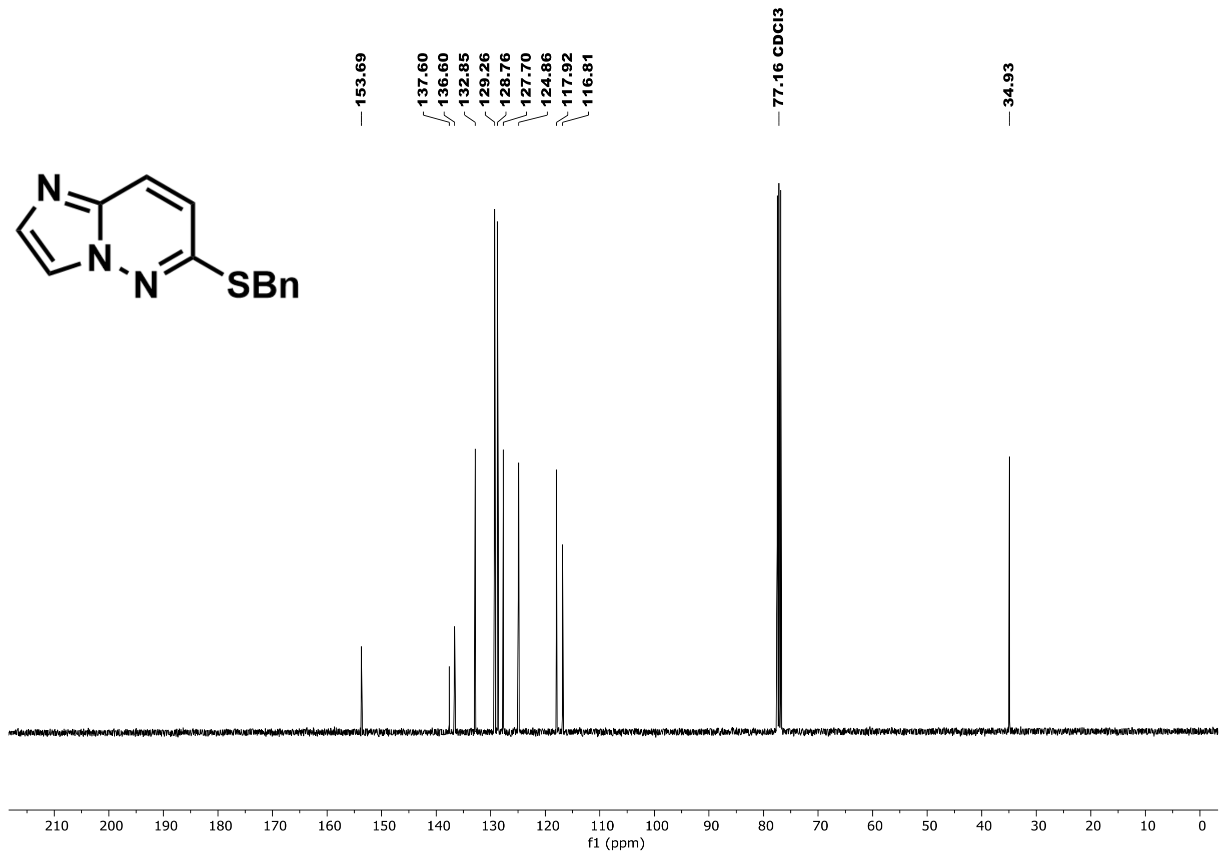
153.69
137.60
136.60
132.85
129.26
128.76
127.70
124.86
117.92
116.81
77.16 CDCl3
34.93
N
N
N
SBn
f1 (ppm)

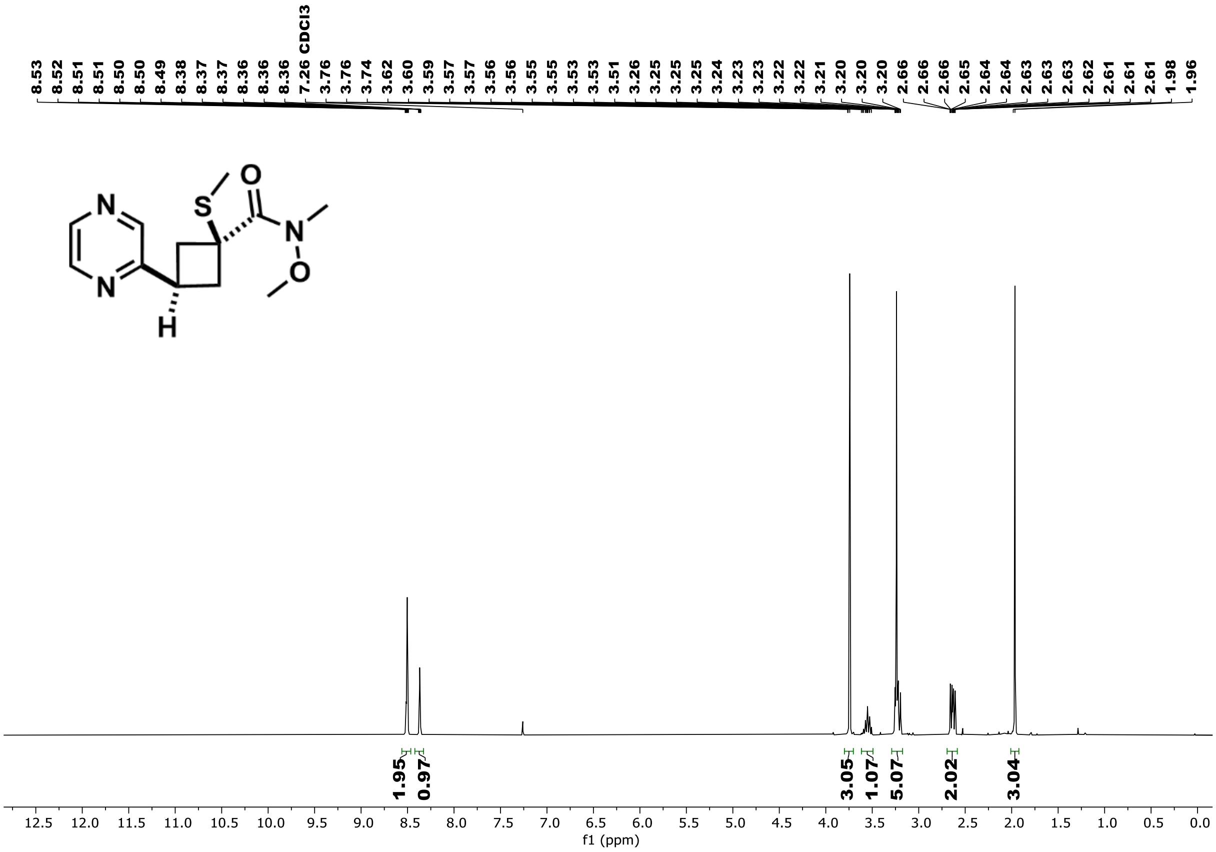
8.53
8.52
8.51
8.51
8.50
8.50
8.49
8.38
8.37
8.37
8.36
8.36
8.36
7.26 CDCl3
3.76
3.76
3.74
3.62
3.60
3.59
3.57
3.57
3.56
3.56
3.55
3.55
3.53
3.53
3.51
3.26
3.25
3.25
3.25
3.24
3.23
3.23
3.22
3.22
3.21
3.20
3.20
3.20
2.66
2.66
2.66
2.65
2.64
2.64
2.63
2.63
2.63
2.62
2.61
2.61
2.61
1.98
1.96
N
N
S
O
N
O
H
1.95
0.97
3.05
1.07
5.07
2.02
3.04
12.5 12.0 11.5 11.0 10.5 10.0 9.5 9.0 8.5 8.0 7.5 7.0 6.5 6.0 5.5 5.0 4.5 4.0 3.5 3.0 2.5 2.0 1.5 1.0 0.5 0.0
f1 (ppm)

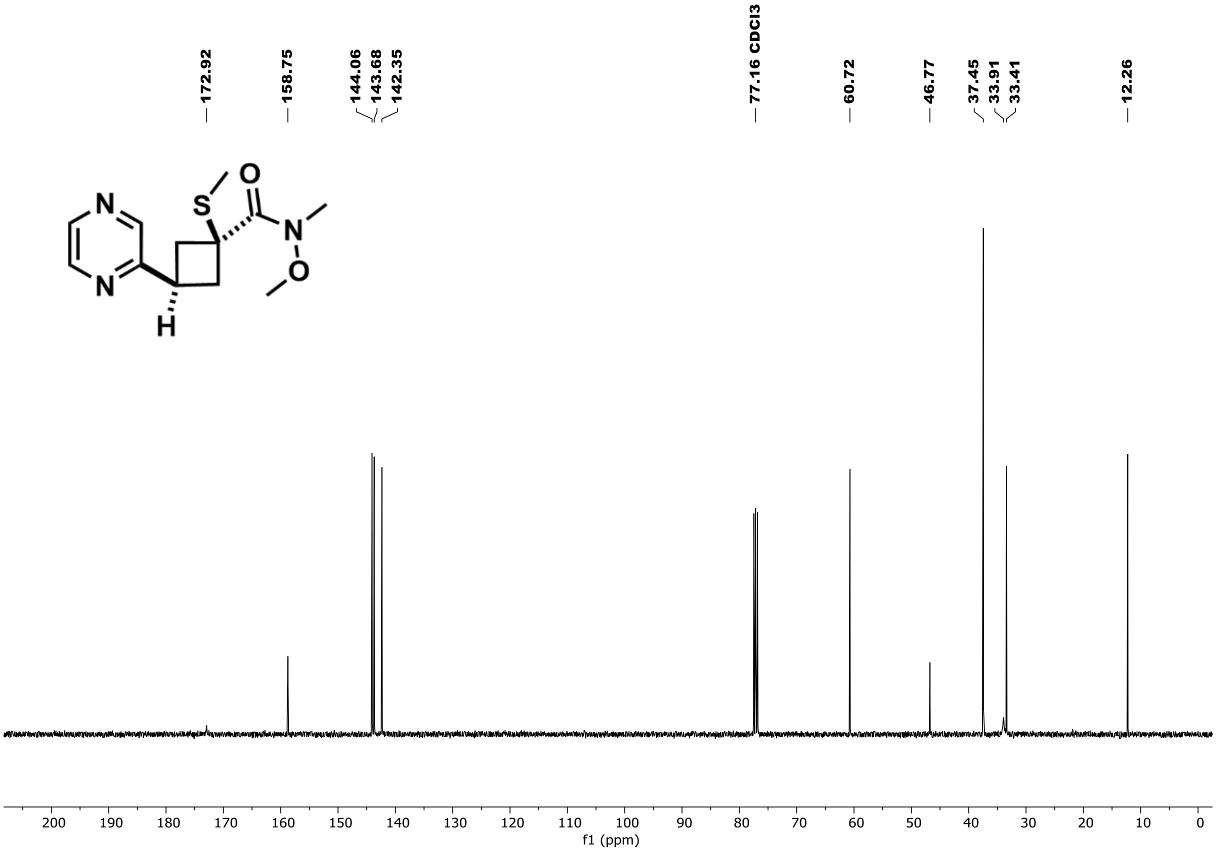
77.16 CDCl3
172.92
158.75
144.06
143.68
142.35
60.72
46.77
37.45
33.91
33.41
12.26
N
N
S
O
N
O
H
200 190 180 170 160 150 140 130 120 110 100 90 80 70 60 50 40 30 20 10 0
f1 (ppm)

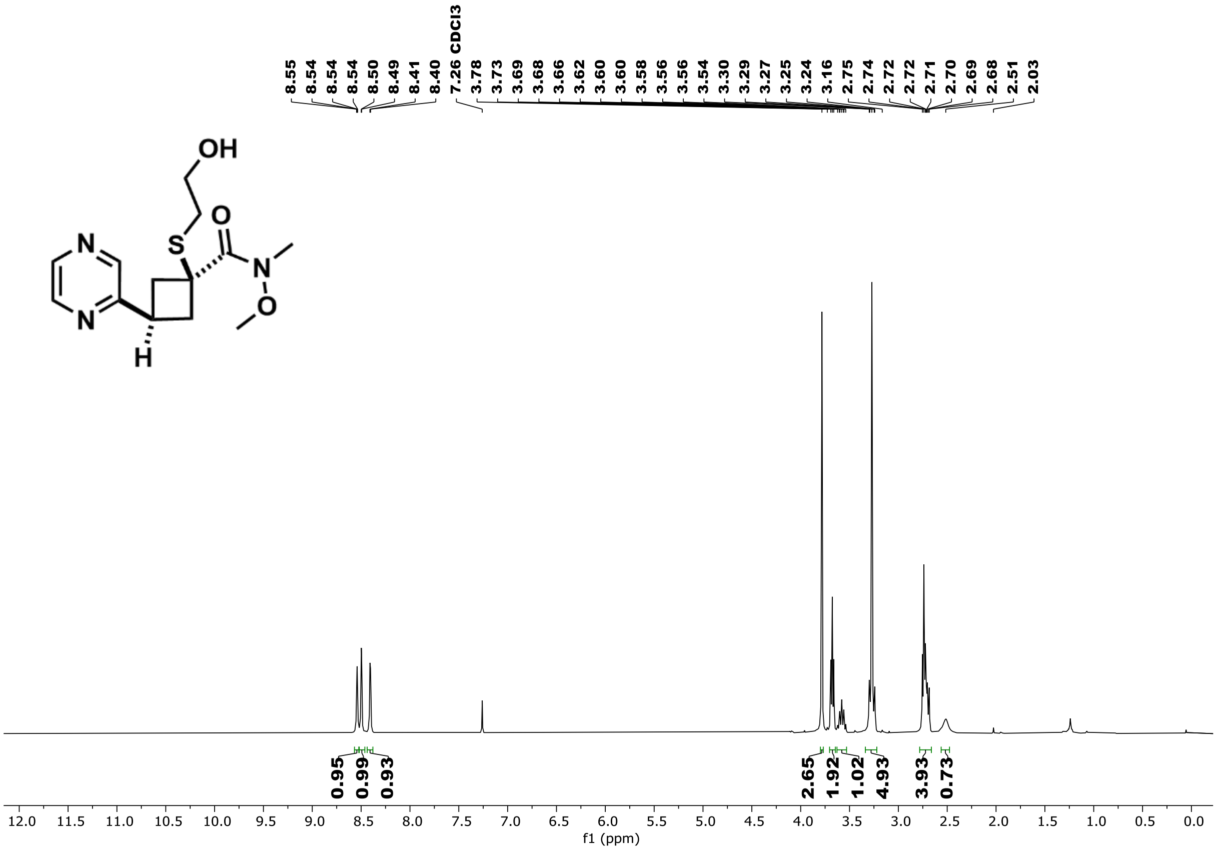

8.55 8.54 8.54 8.54 8.50 8.49 8.41 8.40 7.26 CDCl3 3.78 3.73 3.69 3.68 3.66 3.62 3.60 3.60 3.58 3.56 3.56 3.54 3.30 3.29 3.27 3.25 3.24 3.16 2.75 2.74 2.72 2.72 2.71 2.70 2.69 2.68 2.51 2.03
OH
O
S
N
N
N
O
H
0.95 0.99 0.93
2.65 1.92 1.02 4.93 3.93 0.73
12.0 11.5 11.0 10.5 10.0 9.5 9.0 8.5 8.0 7.5 7.0 6.5 6.0 5.5 5.0 4.5 4.0 3.5 3.0 2.5 2.0 1.5 1.0 0.5 0.0
f1 (ppm)

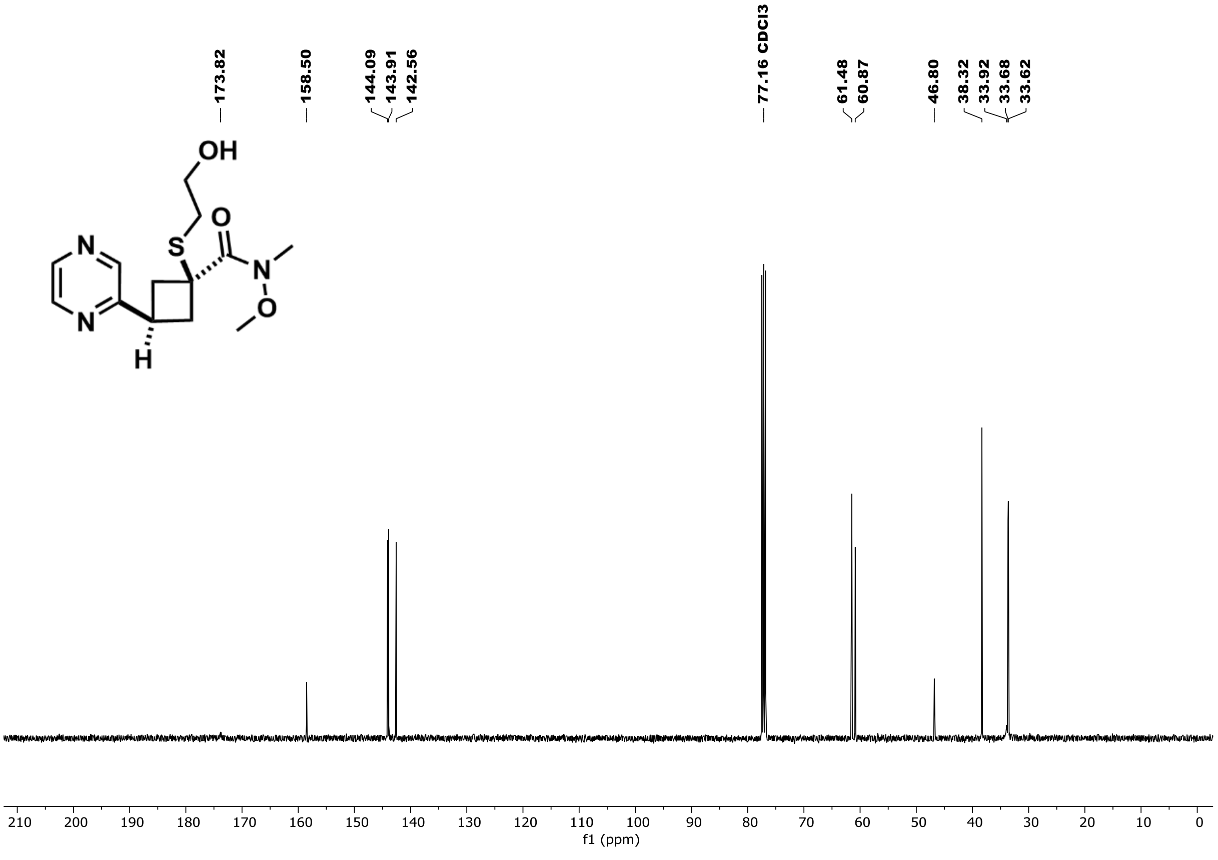

173.82 158.50 144.09 143.91 142.56 77.16 CDCl3 61.48 60.87 46.80 38.32 33.92 33.68 33.62
OH
O
S
N
N
N
O
H
210 200 190 180 170 160 150 140 130 120 110 100 90 80 70 60 50 40 30 20 10 0
f1 (ppm)

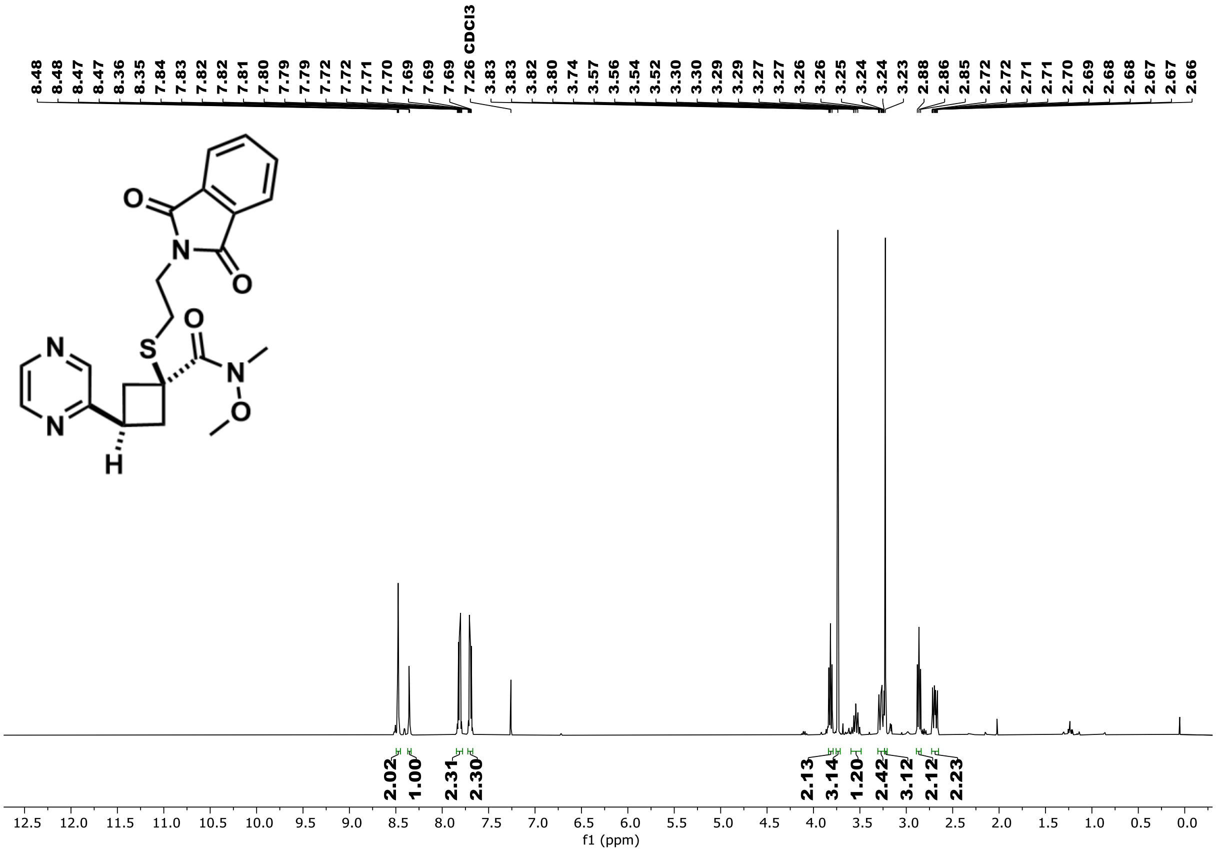
8.48 8.48 8.47 8.47 8.36 8.35 7.84 7.83 7.82 7.82 7.81 7.80 7.79 7.79 7.72 7.72 7.71 7.70 7.69 7.69 7.69 7.26 CDCl3 3.83 3.83 3.82 3.80 3.74 3.57 3.56 3.54 3.52 3.30 3.30 3.29 3.29 3.27 3.27 3.26 3.25 3.24 3.24 3.23 2.88 2.86 2.85 2.72 2.72 2.71 2.71 2.70 2.69 2.68 2.68 2.67 2.67 2.66
O
N
O
S
O
N
O
N
N
H
2.02 1.00 2.31 2.30 2.13 3.14 1.20 2.42 3.12 2.12 2.23
12.5 12.0 11.5 11.0 10.5 10.0 9.5 9.0 8.5 8.0 7.5 7.0 6.5 6.0 5.5 5.0 4.5 4.0 3.5 3.0 2.5 2.0 1.5 1.0 0.5 0.0
f1 (ppm)

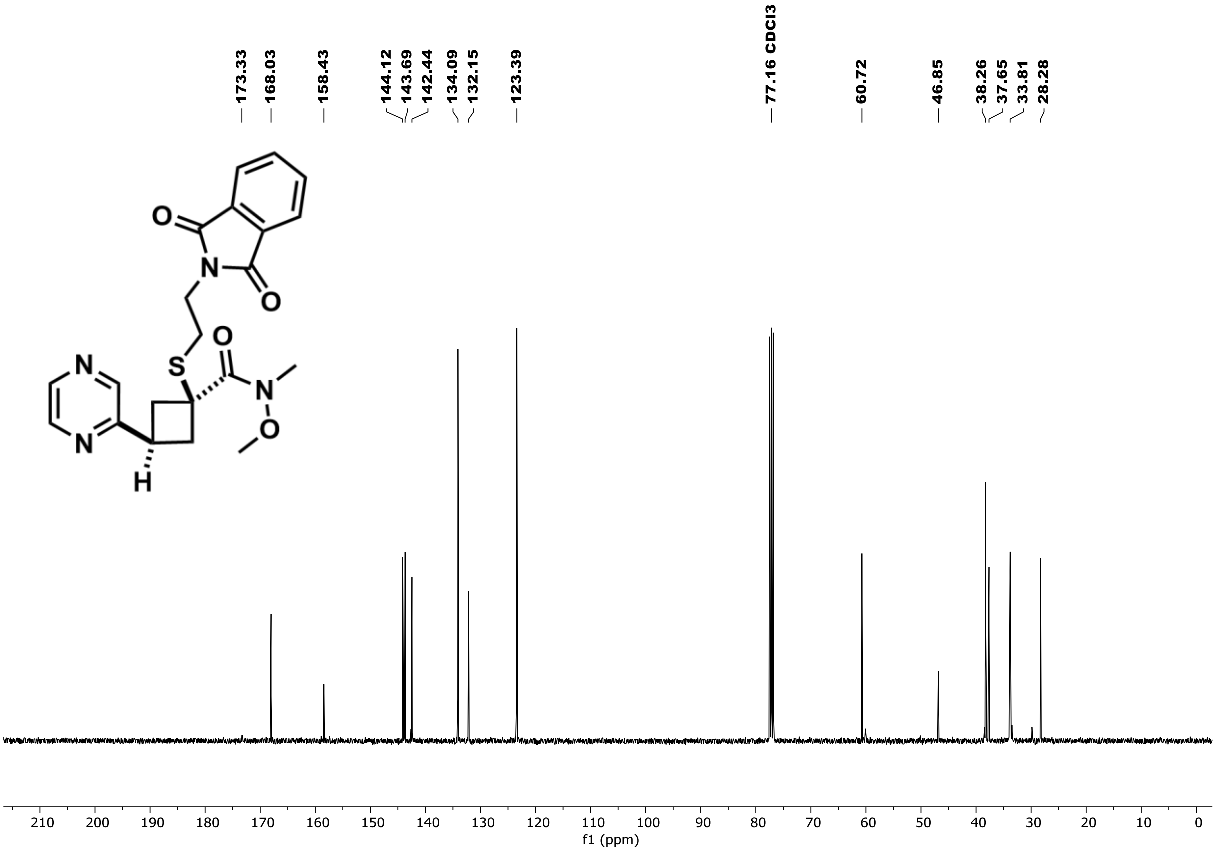
173.33 168.03 158.43 144.12 143.69 142.44 134.09 132.15 123.39 77.16 CDCl3 60.72 46.85 38.26 37.65 33.81 28.28
O
N
O
S
O
N
O
N
N
H
210 200 190 180 170 160 150 140 130 120 110 100 90 80 70 60 50 40 30 20 10 0
f1 (ppm)

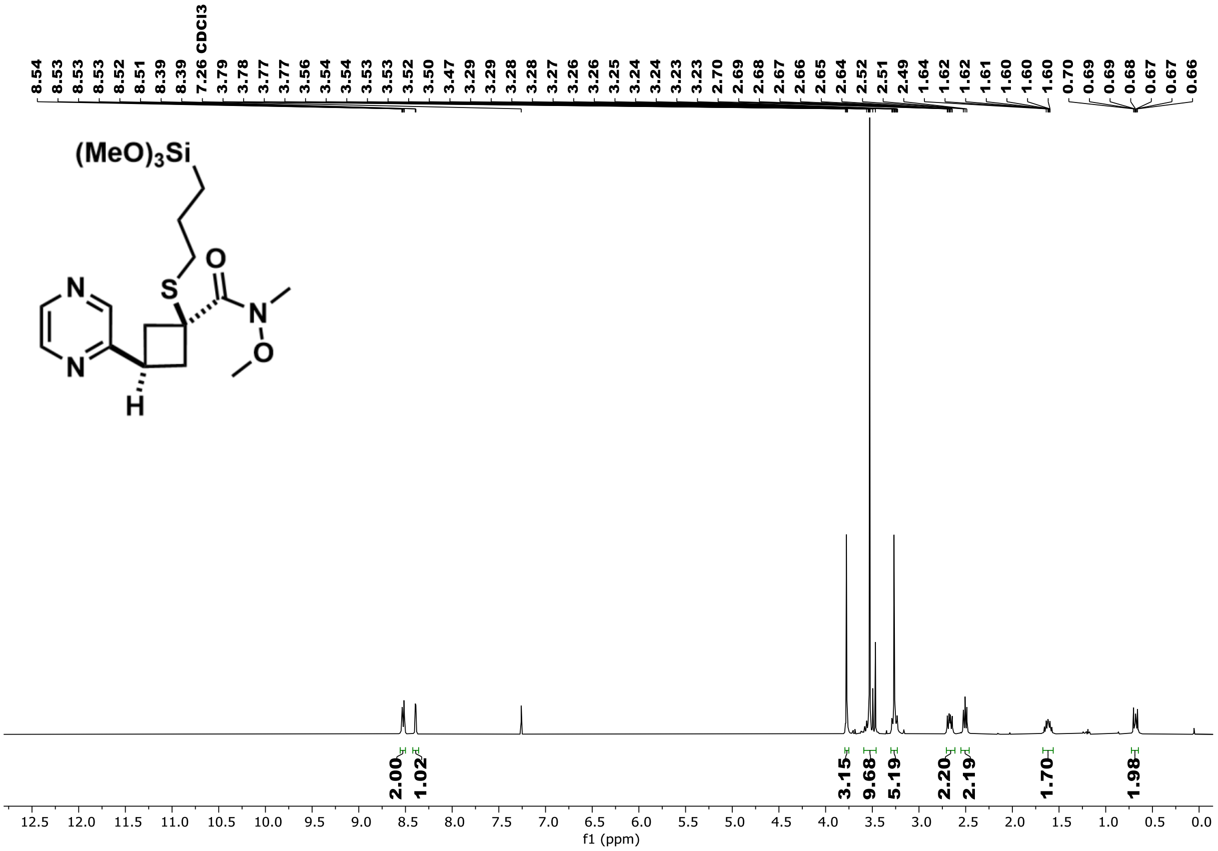
8.54 8.53 8.53 8.53 8.52 8.51 8.39 8.39 7.26 CDCl3 3.79 3.78 3.77 3.77 3.56 3.54 3.54 3.53 3.53 3.52 3.50 3.47 3.29 3.29 3.28 3.28 3.27 3.26 3.26 3.25 3.24 3.24 3.23 3.23 2.70 2.69 2.68 2.67 2.66 2.65 2.64 2.52 2.51 2.49 1.64 1.62 1.62 1.61 1.60 1.60 1.60 0.70 0.69 0.69 0.68 0.67 0.66
(MeO)3Si
S
O
N
N
N
O
H
2.00
1.02
3.15
9.68
5.19
2.20
2.19
1.70
1.98
12.5 12.0 11.5 11.0 10.5 10.0 9.5 9.0 8.5 8.0 7.5 7.0 6.5 6.0 5.5 5.0 4.5 4.0 3.5 3.0 2.5 2.0 1.5 1.0 0.5 0.0
f1 (ppm)

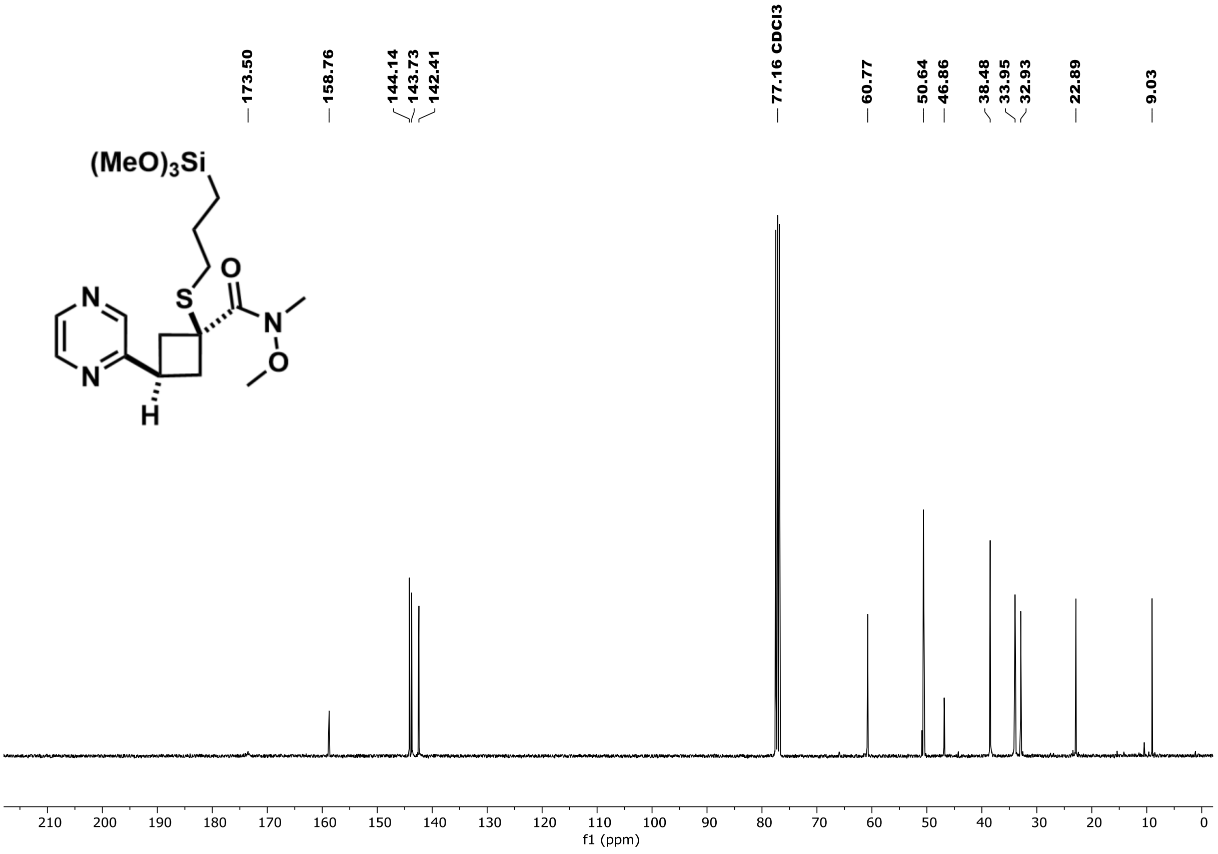
173.50
158.76
144.14
143.73
142.41
77.16 CDCl3
60.77
50.64
46.86
38.48
33.95
32.93
22.89
9.03
(MeO)3Si
S
O
N
N
N
O
H
210 200 190 180 170 160 150 140 130 120 110 100 90 80 70 60 50 40 30 20 10 0
f1 (ppm)

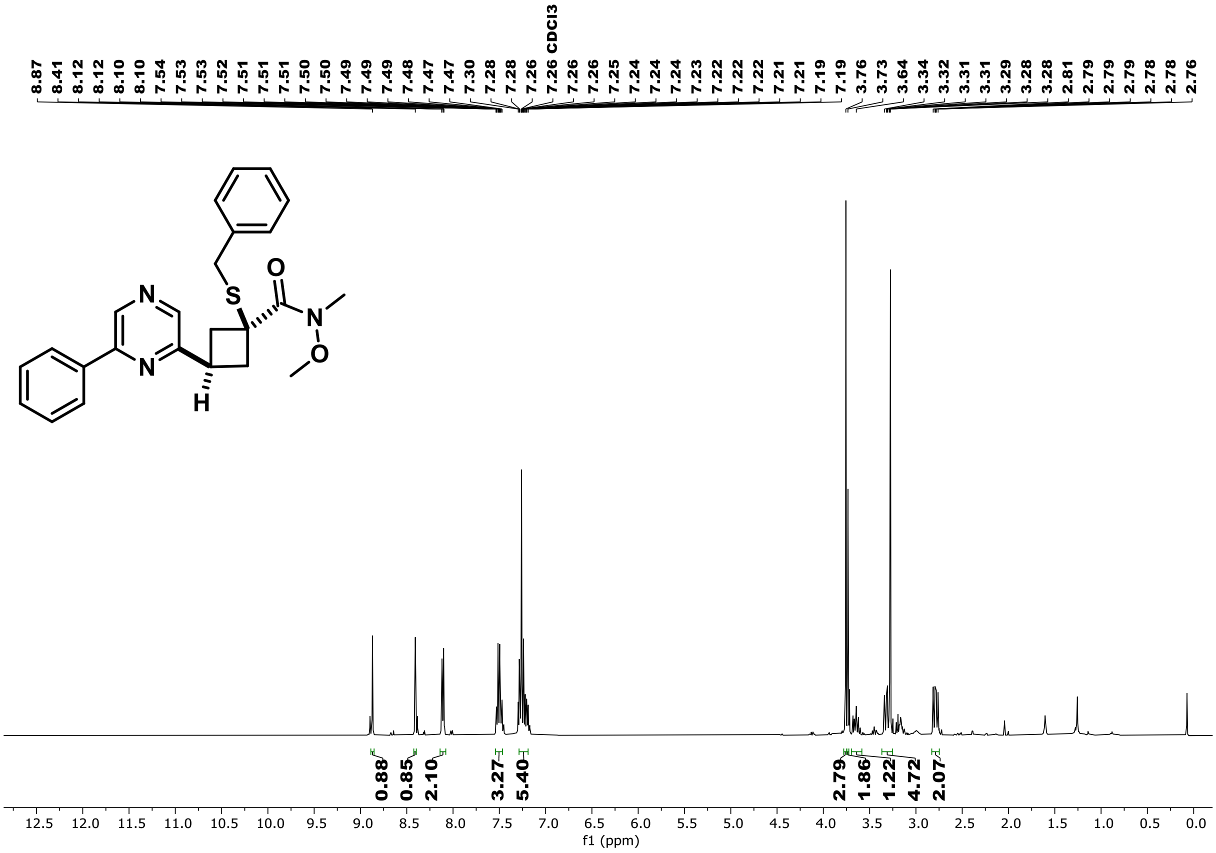
8.87
8.41
8.12
8.12
8.10
8.10
7.54
7.53
7.53
7.52
7.51
7.51
7.51
7.50
7.50
7.49
7.49
7.49
7.48
7.47
7.47
7.30
7.28
7.28
7.26 CDCl3
7.26
7.26
7.25
7.24
7.24
7.24
7.23
7.22
7.22
7.22
7.21
7.21
7.19
7.19
3.76
3.73
3.64
3.34
3.32
3.31
3.31
3.29
3.28
3.28
2.81
2.79
2.79
2.79
2.78
2.78
2.76
N
N
S
O
N
O
H
0.88
0.85
2.10
3.27
5.40
2.79
1.86
1.22
4.72
2.07
12.5 12.0 11.5 11.0 10.5 10.0 9.5 9.0 8.5 8.0 7.5 7.0 6.5 6.0 5.5 5.0 4.5 4.0 3.5 3.0 2.5 2.0 1.5 1.0 0.5 0.0
f1 (ppm)

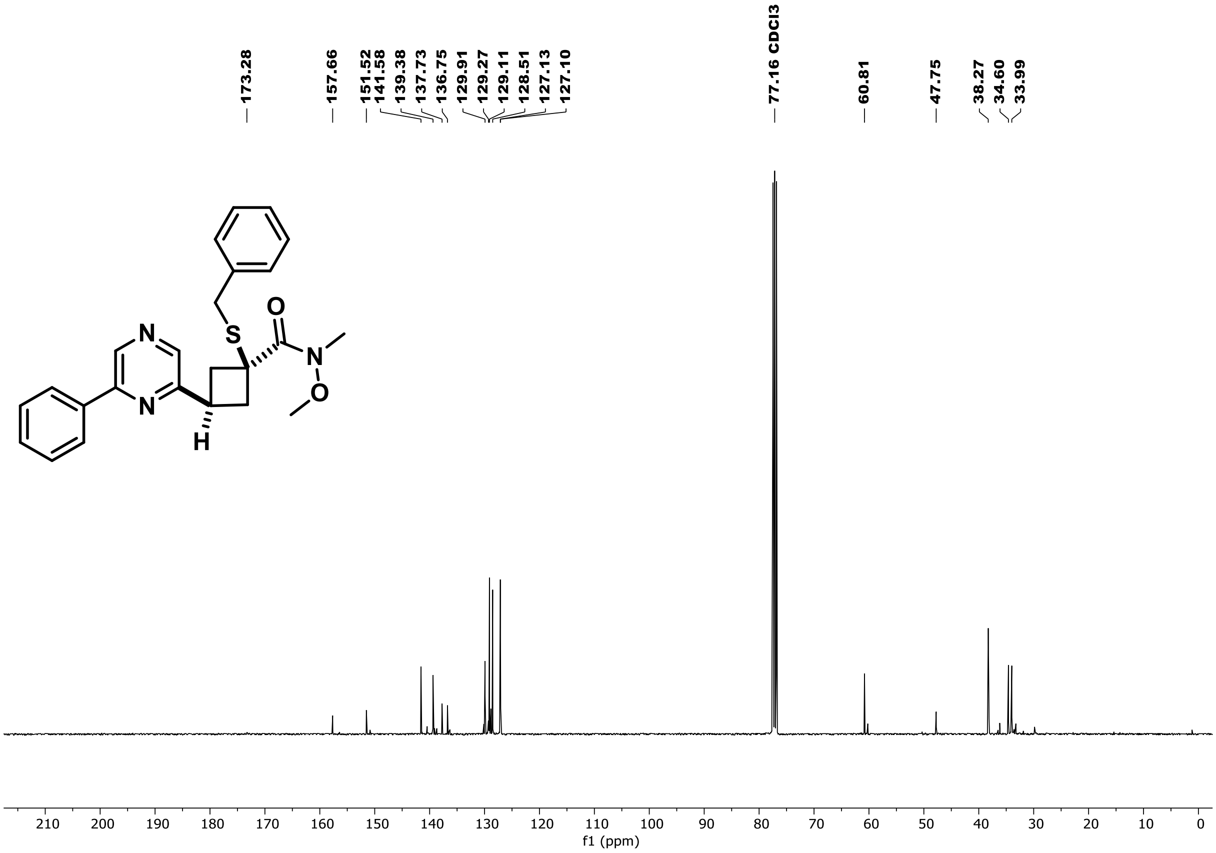
173.28
157.66
151.52
141.58
139.38
137.73
136.75
129.91
129.27
129.11
128.51
127.13
127.10
77.16 CDCl3
60.81
47.75
38.27
34.60
33.99
N
N
S
O
N
O
H
210 200 190 180 170 160 150 140 130 120 110 100 90 80 70 60 50 40 30 20 10 0
f1 (ppm)

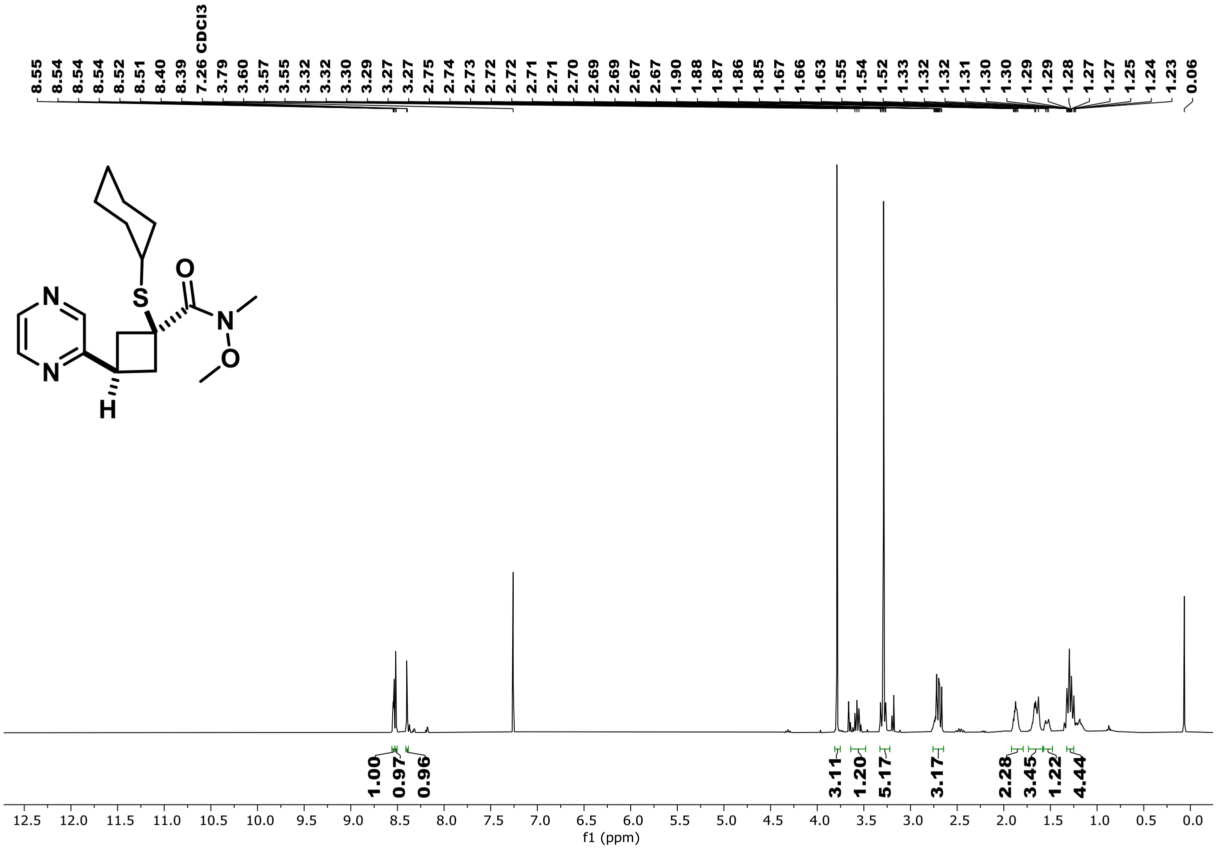
8.55
8.54
8.54
8.54
8.52
8.51
8.40
8.39
7.26 CDCl3
3.79
3.60
3.57
3.55
3.32
3.32
3.30
3.29
3.27
3.27
2.75
2.74
2.73
2.72
2.72
2.71
2.71
2.70
2.69
2.69
2.67
2.67
1.90
1.88
1.87
1.86
1.85
1.67
1.66
1.63
1.55
1.54
1.52
1.33
1.32
1.32
1.31
1.30
1.29
1.29
1.28
1.27
1.27
1.25
1.24
1.23
0.06
N
N
S
O
N
O
H
1.00
0.97
0.96
3.11
1.20
5.17
3.17
2.28
3.45
1.22
4.44
f1 (ppm)

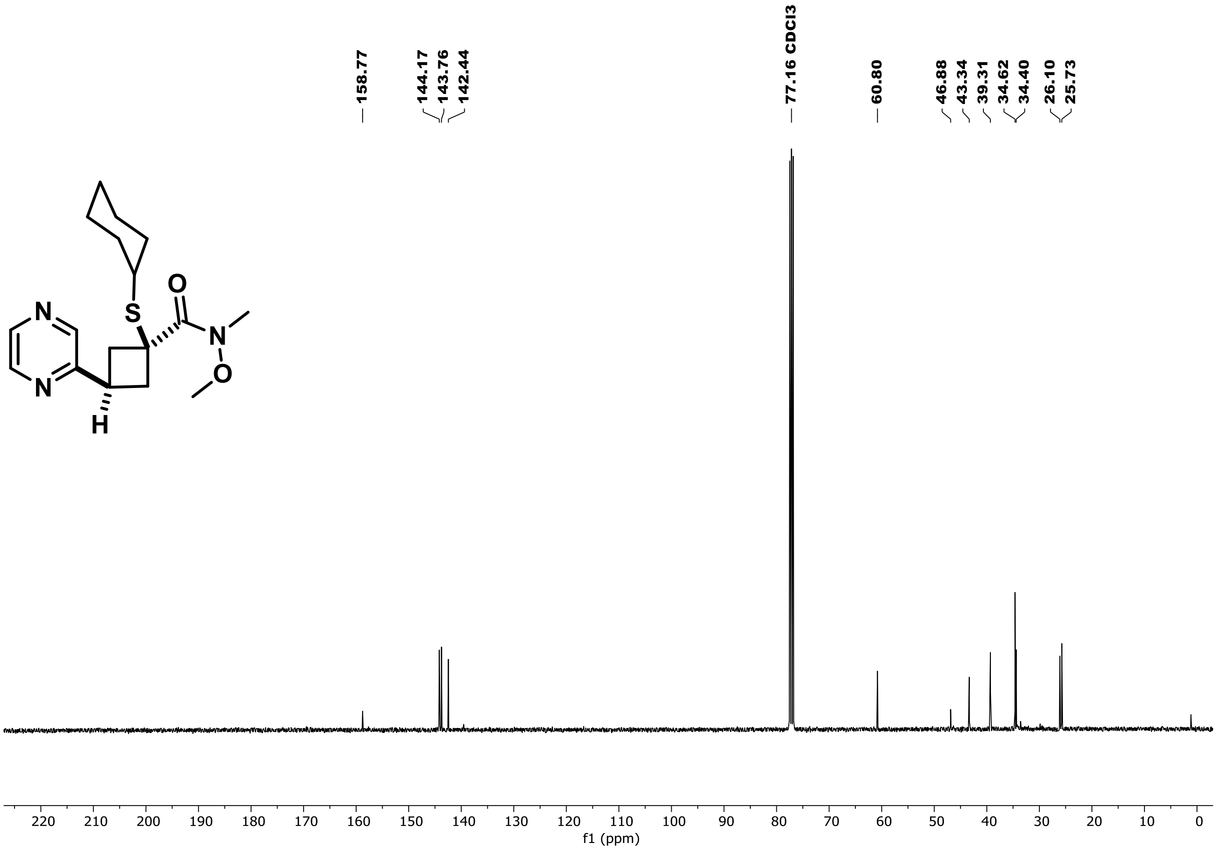
158.77
144.17
143.76
142.44
77.16 CDCl3
60.80
46.88
43.34
39.31
34.62
34.40
26.10
25.73
N
N
S
O
N
O
H
f1 (ppm)

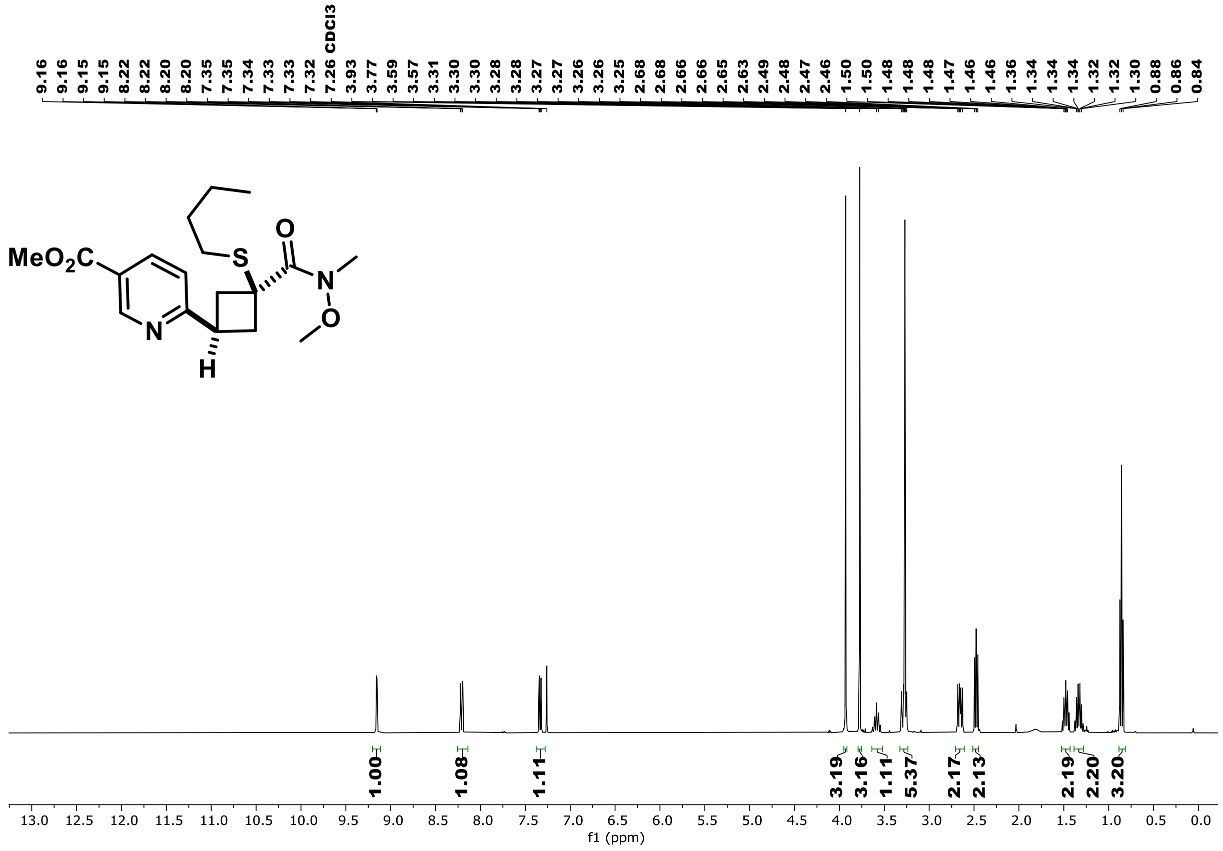
9.16 9.16 9.15 9.15 8.22 8.22 8.20 8.20 7.35 7.35 7.34 7.33 7.33 7.32 7.26 CDCl3 3.93 3.77 3.59 3.57 3.31 3.30 3.30 3.28 3.28 3.27 3.27 3.26 3.26 3.25 2.68 2.68 2.66 2.66 2.65 2.63 2.49 2.48 2.47 2.46 1.50 1.50 1.48 1.48 1.48 1.47 1.46 1.46 1.36 1.34 1.34 1.34 1.32 1.32 1.30 0.88 0.86 0.84
MeO2C
S
O
N
O
N
H
1.00
1.08
1.11
3.19
3.16
1.11
5.37
2.17
2.13
2.19
2.20
3.20
13.0 12.5 12.0 11.5 11.0 10.5 10.0 9.5 9.0 8.5 8.0 7.5 7.0 6.5 6.0 5.5 5.0 4.5 4.0 3.5 3.0 2.5 2.0 1.5 1.0 0.5 0.0
f1 (ppm)

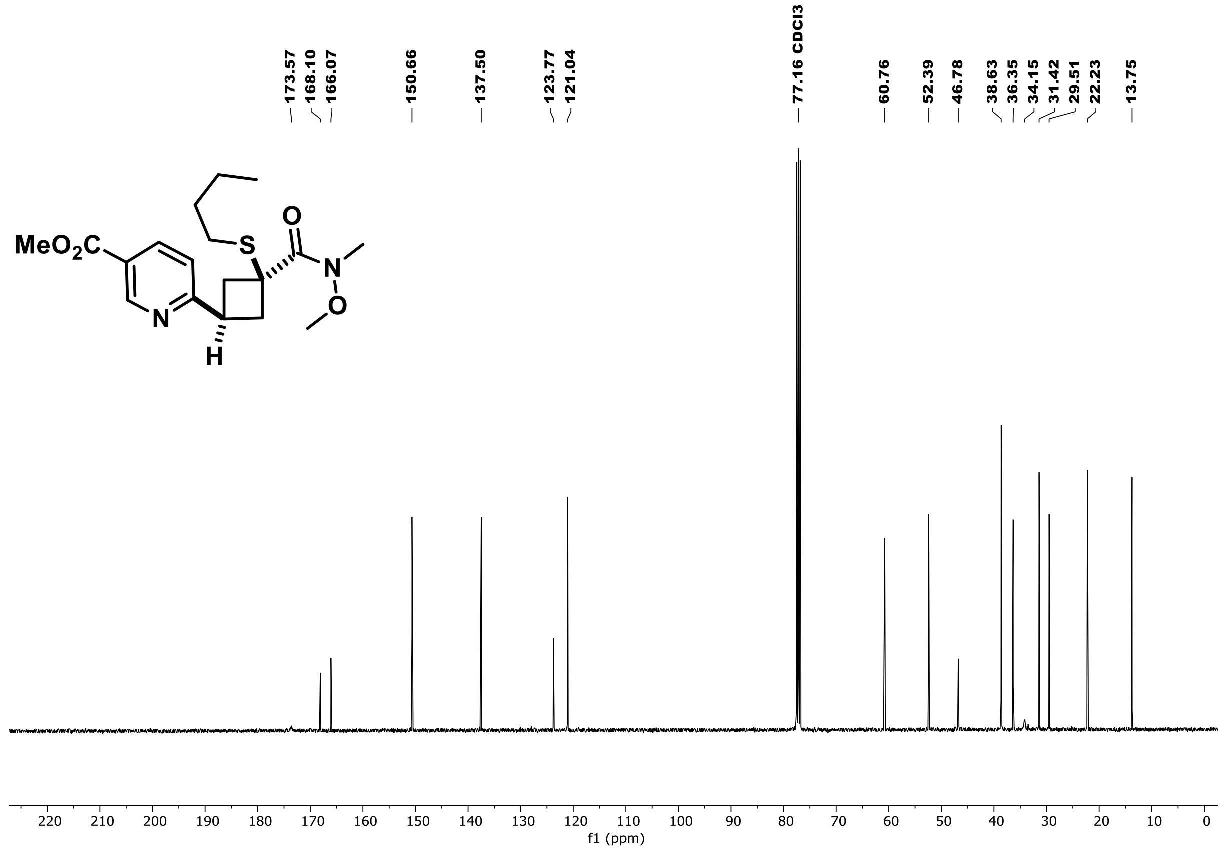
173.57 168.10 166.07 150.66 137.50 123.77 121.04 77.16 CDCl3 60.76 52.39 46.78 38.63 36.35 34.15 31.42 29.51 22.23 13.75
MeO2C
S
O
N
O
N
H
220 210 200 190 180 170 160 150 140 130 120 110 100 90 80 70 60 50 40 30 20 10 0
f1 (ppm)

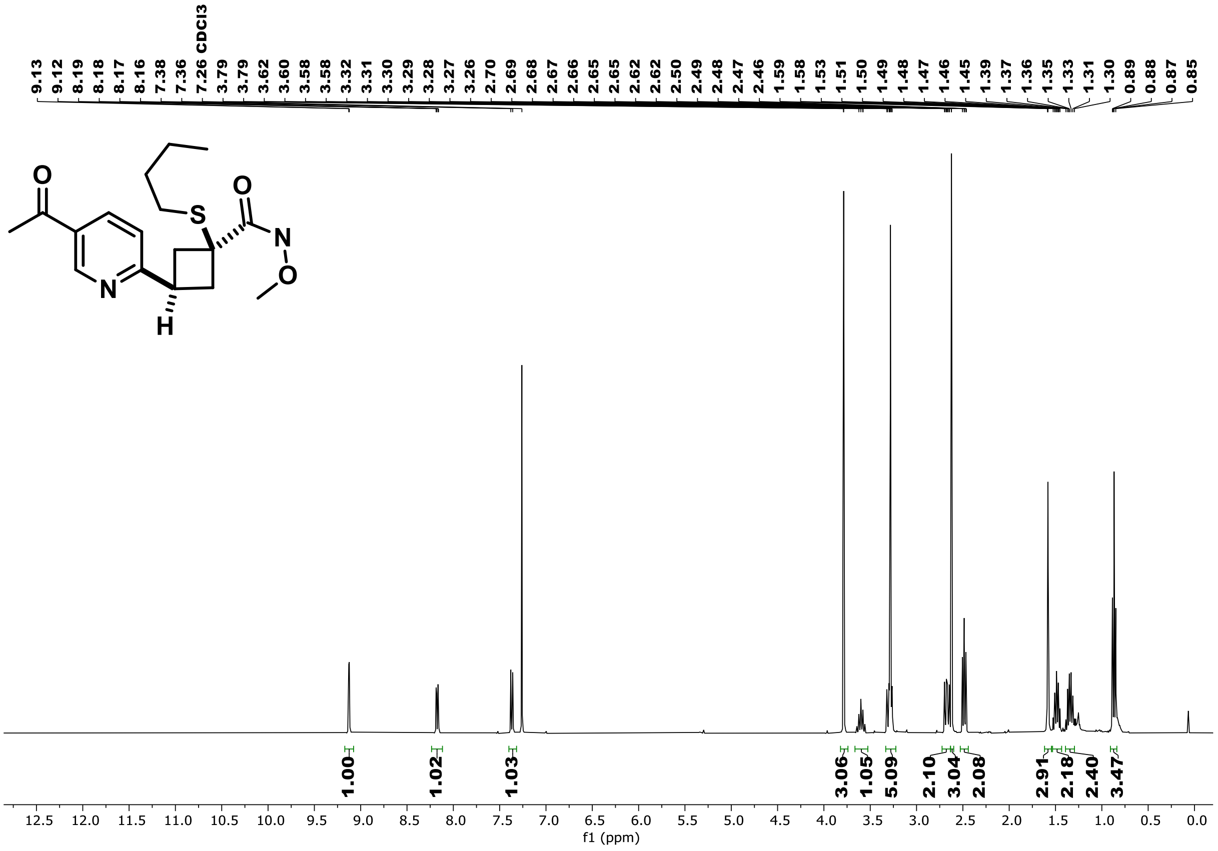
9.13
9.12
8.19
8.18
8.17
8.16
7.38
7.36
7.26 CDCl3
3.79
3.79
3.62
3.60
3.58
3.58
3.32
3.31
3.30
3.29
3.28
3.27
3.26
2.70
2.69
2.68
2.67
2.66
2.65
2.65
2.62
2.62
2.50
2.49
2.48
2.47
2.46
1.59
1.58
1.53
1.51
1.50
1.49
1.48
1.47
1.46
1.45
1.39
1.37
1.36
1.35
1.33
1.31
1.30
0.89
0.88
0.87
0.85
O
S
O
N
O
N
H
1.00
1.02
1.03
3.06
1.05
5.09
2.10
3.04
2.08
2.91
2.18
2.40
3.47
f1 (ppm)

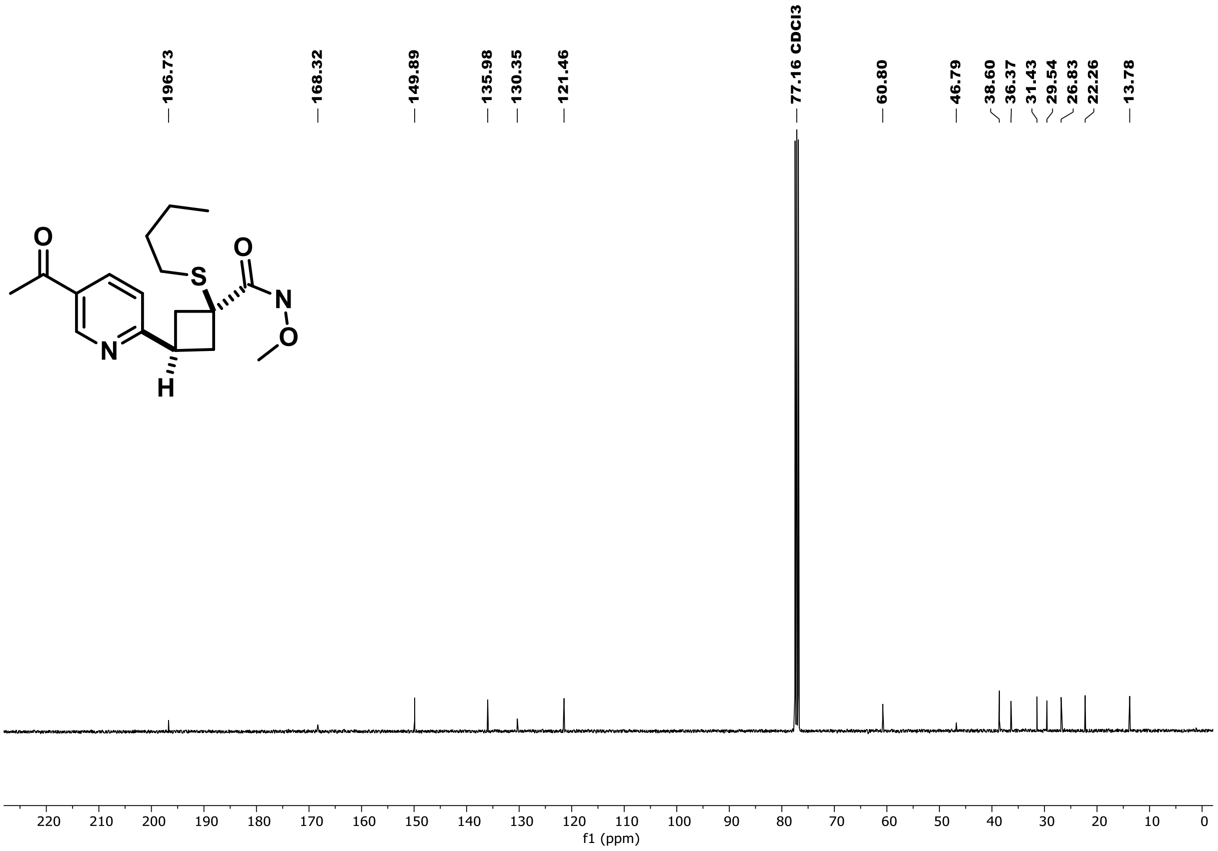
196.73
168.32
149.89
135.98
130.35
121.46
77.16 CDCl3
60.80
46.79
38.60
36.37
31.43
29.54
26.83
22.26
13.78
O
S
O
N
O
N
H
f1 (ppm)

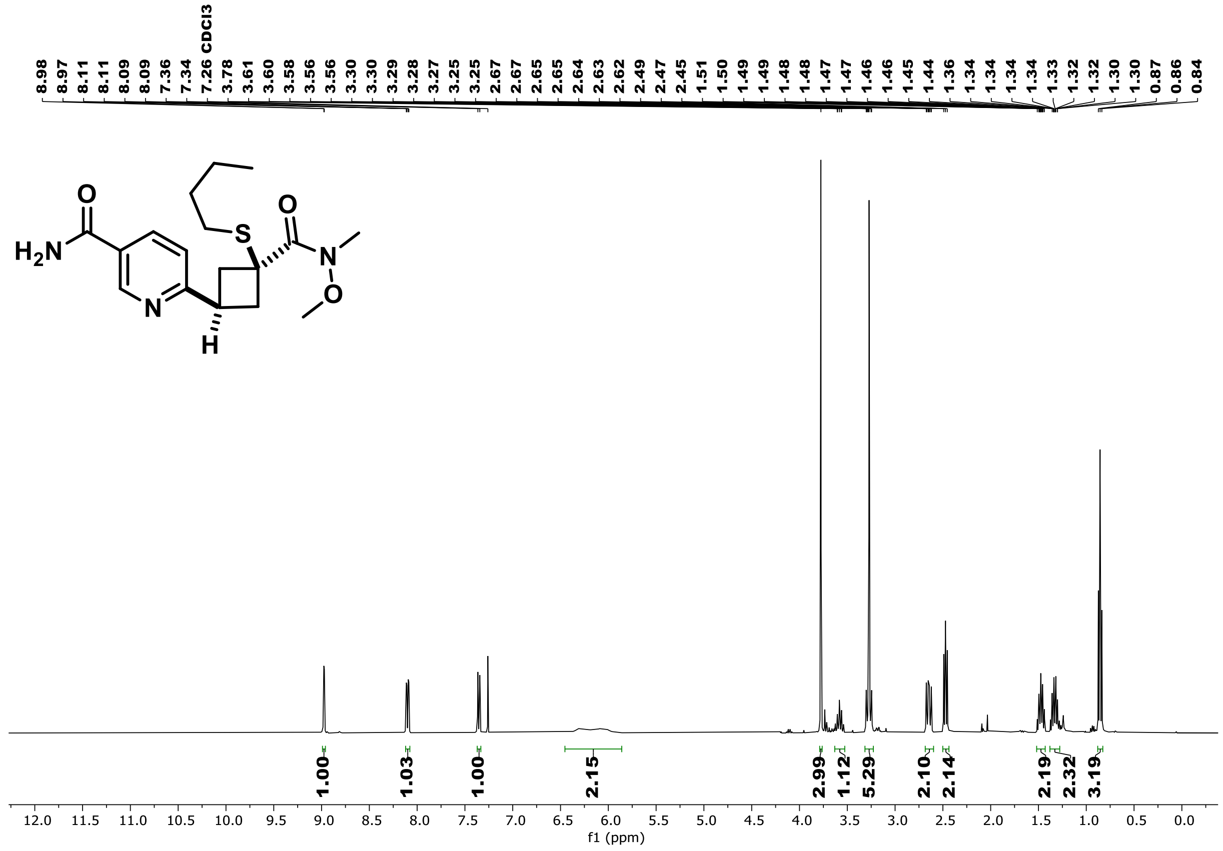
8.98 8.97 8.11 8.11 8.09 8.09 7.36 7.34 7.26 CDCl3 3.78 3.61 3.60 3.58 3.56 3.56 3.30 3.30 3.29 3.28 3.27 3.25 3.25 2.67 2.67 2.65 2.65 2.64 2.63 2.62 2.49 2.47 2.45 1.51 1.50 1.49 1.49 1.48 1.48 1.47 1.47 1.46 1.46 1.45 1.44 1.36 1.34 1.34 1.34 1.34 1.33 1.32 1.32 1.30 1.30 0.87 0.86 0.84
H2N
O
N
H
S
O
N
O
1.00 1.03 1.00 2.15 2.99 1.12 5.29 2.10 2.14 2.19 2.32 3.19
12.0 11.5 11.0 10.5 10.0 9.5 9.0 8.5 8.0 7.5 7.0 6.5 6.0 5.5 5.0 4.5 4.0 3.5 3.0 2.5 2.0 1.5 1.0 0.5 0.0
f1 (ppm)

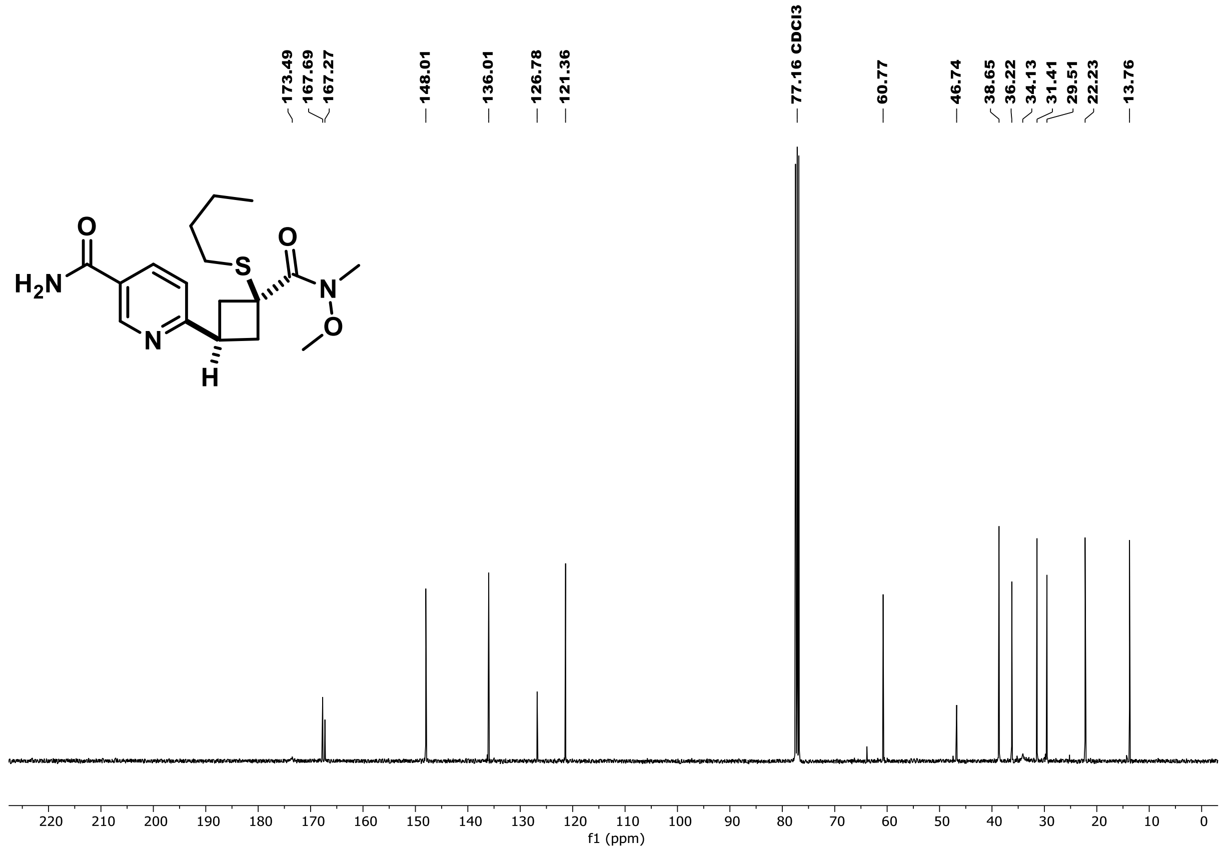
173.49 167.69 167.27 148.01 136.01 126.78 121.36 77.16 CDCl3 60.77 46.74 38.65 36.22 34.13 31.41 29.51 22.23 13.76
H2N
O
N
H
S
O
N
O
220 210 200 190 180 170 160 150 140 130 120 110 100 90 80 70 60 50 40 30 20 10 0
f1 (ppm)

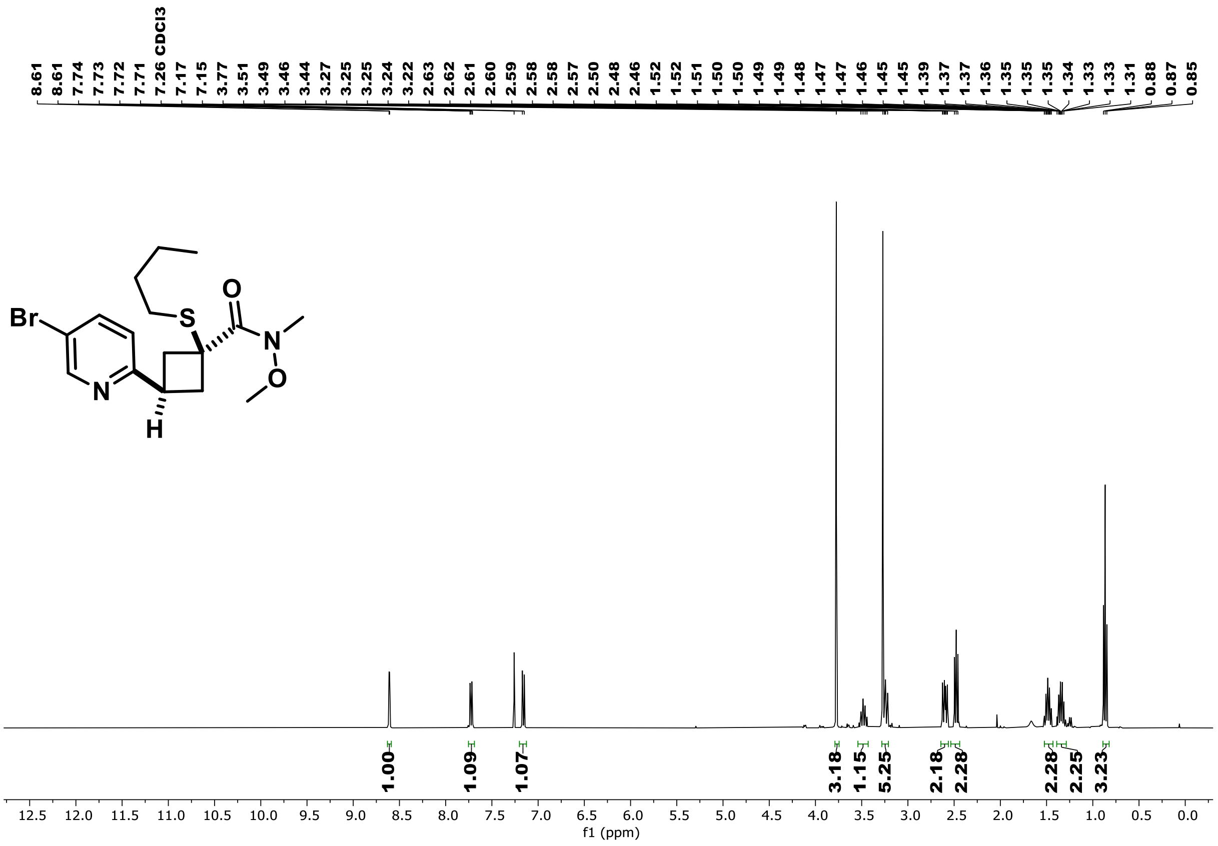
8.61
8.61
7.74
7.73
7.72
7.71
7.26 CDCl3
7.17
7.15
3.77
3.51
3.49
3.46
3.44
3.27
3.25
3.25
3.24
3.22
2.63
2.62
2.61
2.60
2.59
2.58
2.58
2.57
2.50
2.48
2.46
1.52
1.52
1.51
1.50
1.50
1.49
1.49
1.48
1.47
1.47
1.46
1.45
1.45
1.39
1.37
1.37
1.36
1.35
1.35
1.35
1.34
1.33
1.33
1.31
0.88
0.87
0.85
Br
S
O
N
O
N
H
1.00
1.09
1.07
3.18
1.15
5.25
2.18
2.28
2.28
2.25
3.23
f1 (ppm)

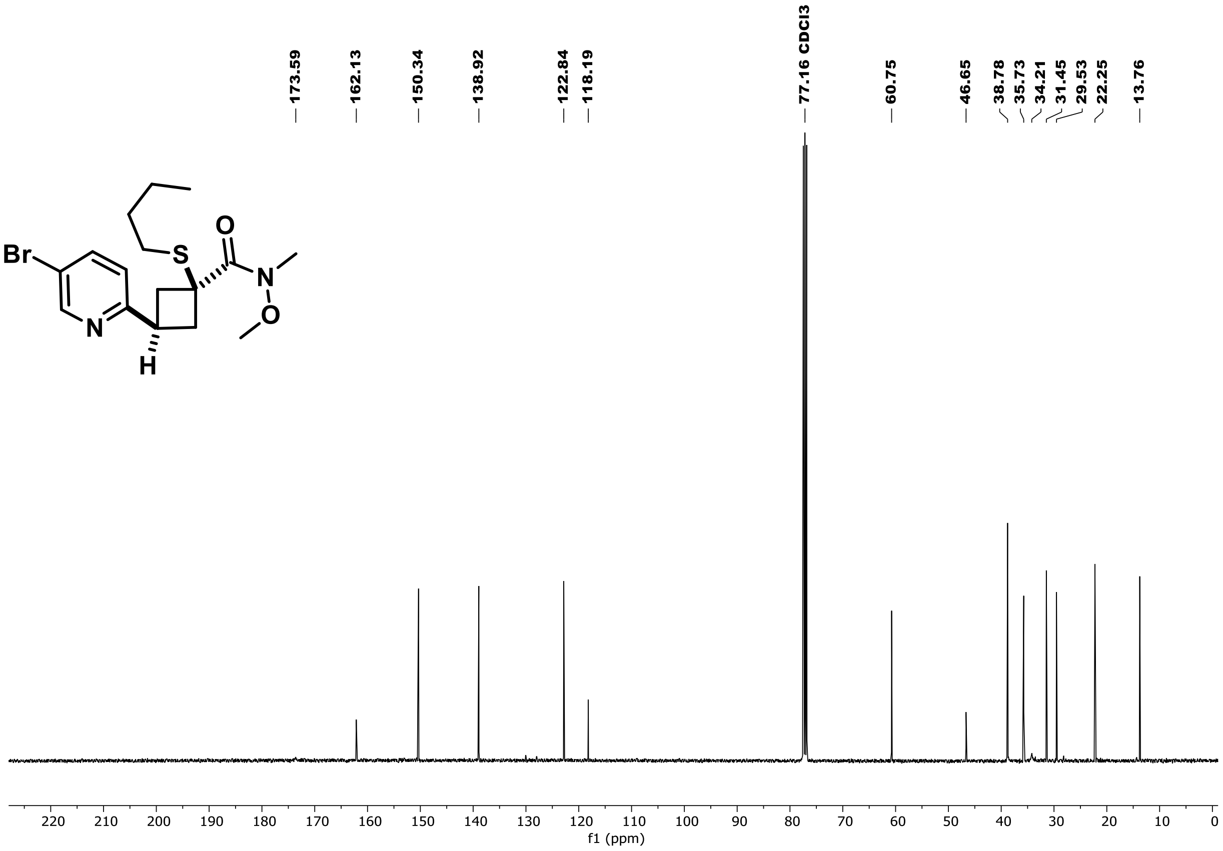
173.59
162.13
150.34
138.92
122.84
118.19
77.16 CDCl3
60.75
46.65
38.78
35.73
34.21
31.45
29.53
22.25
13.76
Br
S
O
N
O
N
H
f1 (ppm)

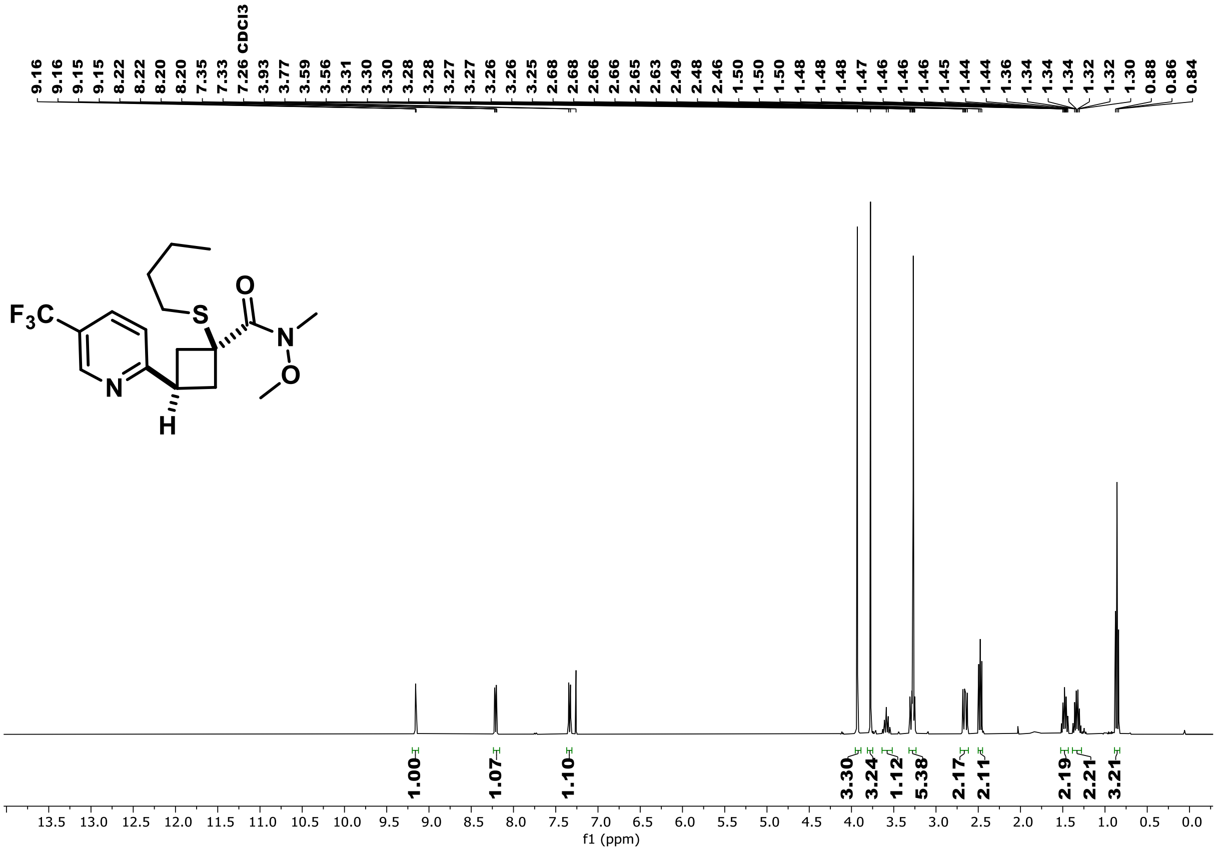
9.16 9.16 9.15 9.15 8.22 8.22 8.20 8.20 7.35 7.33 7.26 CDCl3 3.93 3.77 3.59 3.56 3.31 3.30 3.30 3.28 3.28 3.27 3.27 3.26 3.26 3.25 2.68 2.68 2.66 2.66 2.65 2.63 2.49 2.48 2.46 1.50 1.50 1.50 1.48 1.48 1.48 1.47 1.46 1.46 1.46 1.45 1.44 1.44 1.36 1.34 1.34 1.34 1.32 1.32 1.30 0.88 0.86 0.84
F3C
S
O
N
O
N
H
1.00
1.07
1.10
3.30
3.24
1.12
5.38
2.17
2.11
2.19
2.21
3.21
13.5 13.0 12.5 12.0 11.5 11.0 10.5 10.0 9.5 9.0 8.5 8.0 7.5 7.0 6.5 6.0 5.5 5.0 4.5 4.0 3.5 3.0 2.5 2.0 1.5 1.0 0.5 0.0
f1 (ppm)

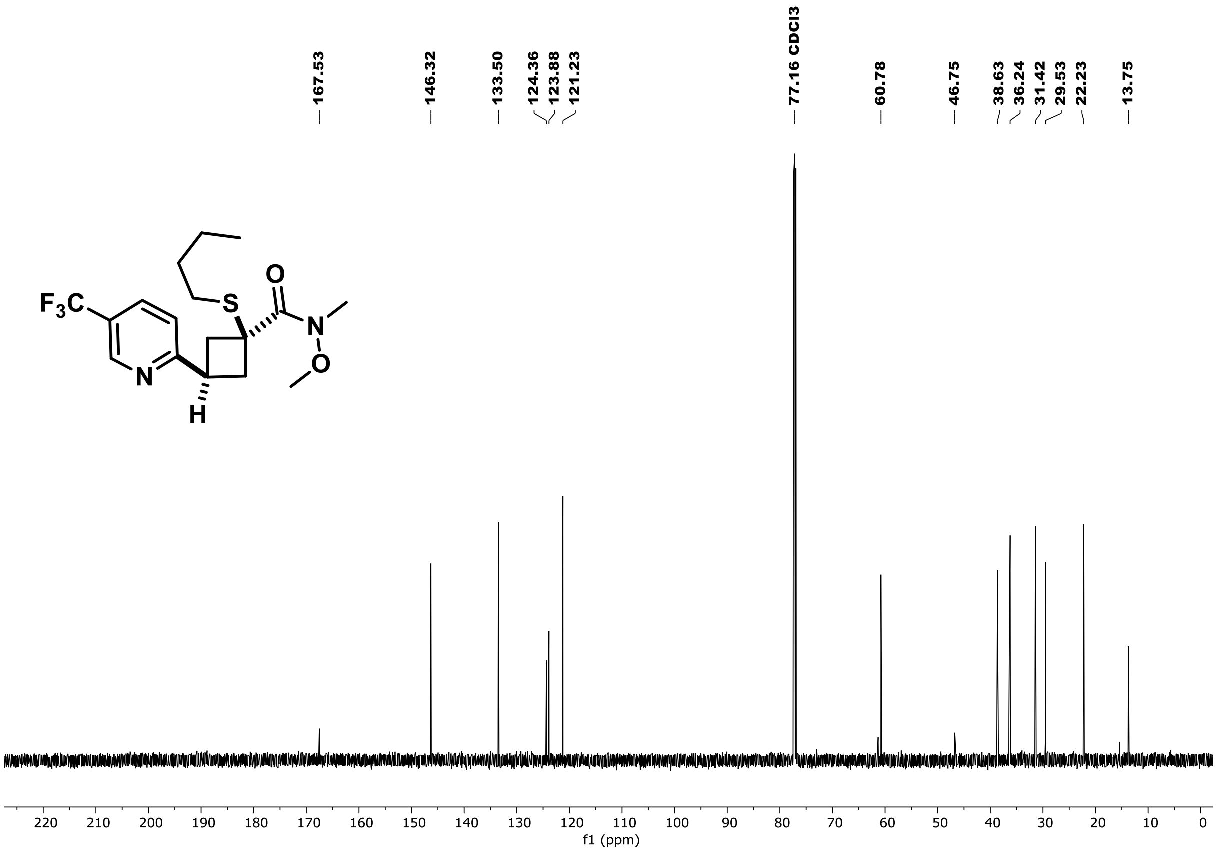
167.53
146.32
133.50
124.36 123.88 121.23
77.16 CDCl3
60.78
46.75
38.63 36.24 31.42 29.53 22.23
13.75
F3C
S
O
N
O
N
H
220 210 200 190 180 170 160 150 140 130 120 110 100 90 80 70 60 50 40 30 20 10 0
f1 (ppm)

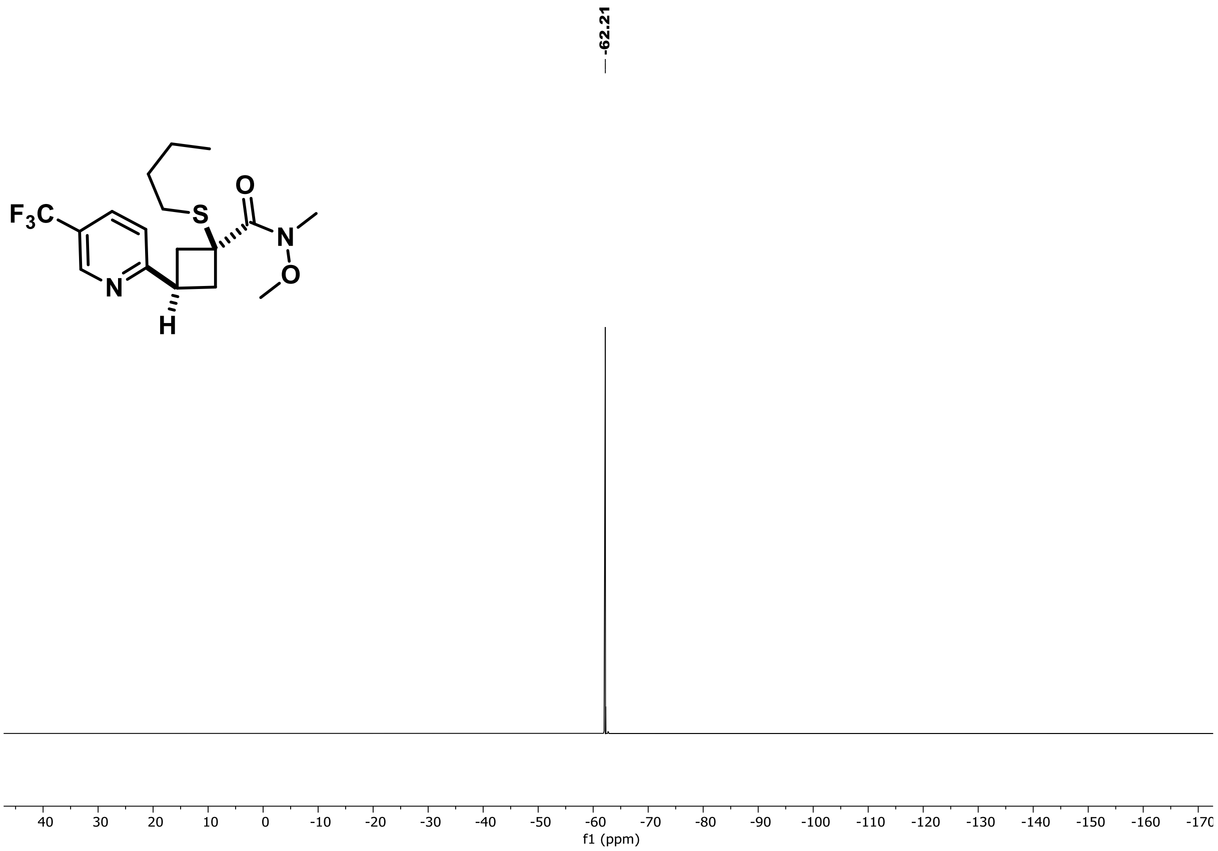

-62.21
F3C
S
O
N
O
N
H
40 30 20 10 0 -10 -20 -30 -40 -50 -60 -70 -80 -90 -100 -110 -120 -130 -140 -150 -160 -170
f1 (ppm)

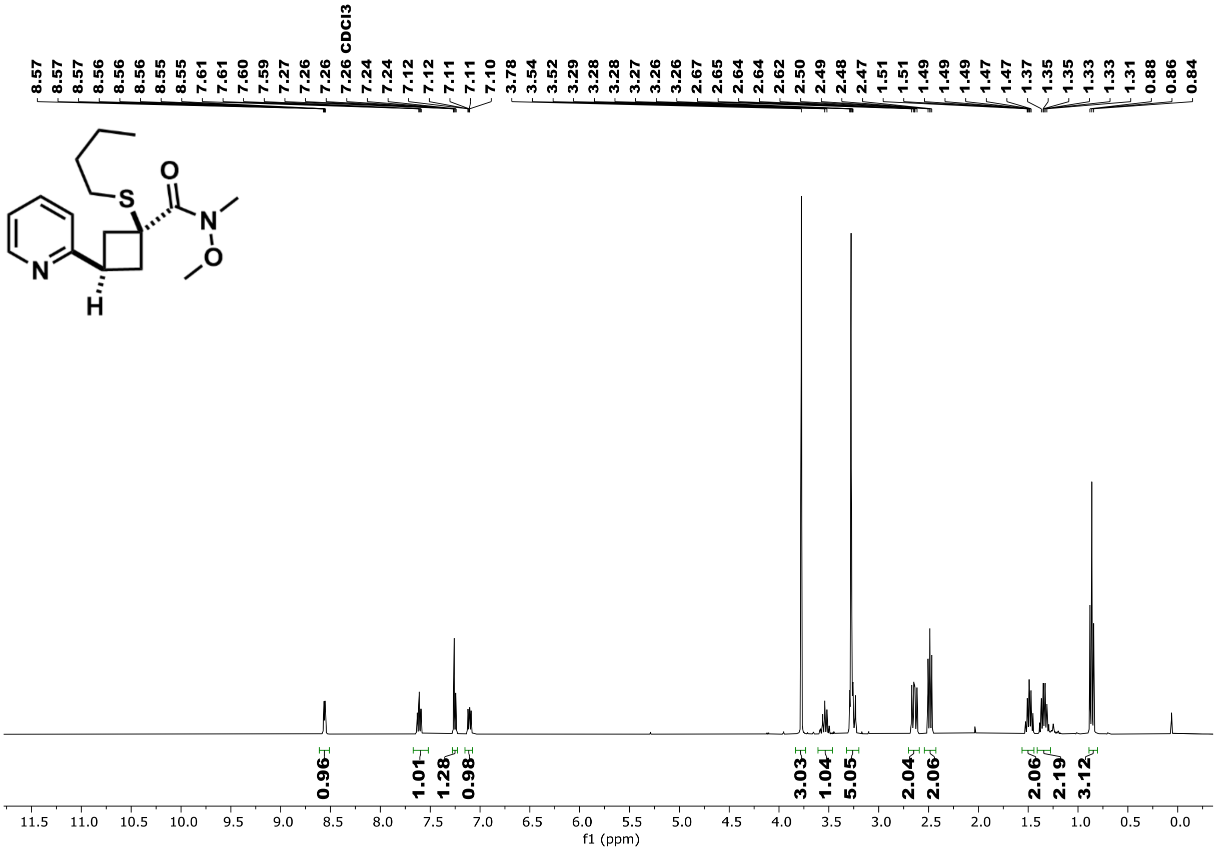

8.57 8.57 8.57 8.56 8.56 8.56 8.55 8.55 7.61 7.61 7.60 7.59 7.27 7.26 7.26 CDCl3 7.24 7.24 7.12 7.12 7.11 7.11 7.10 3.78 3.54 3.52 3.29 3.28 3.28 3.27 3.26 3.26 2.67 2.65 2.64 2.64 2.62 2.50 2.49 2.48 2.47 1.51 1.51 1.49 1.49 1.49 1.47 1.47 1.37 1.35 1.35 1.33 1.33 1.31 0.88 0.86 0.84
S
O
N
O
N
H
0.96 1.01 1.28 0.98 3.03 1.04 5.05 2.04 2.06 2.06 2.19 3.12
11.5 11.0 10.5 10.0 9.5 9.0 8.5 8.0 7.5 7.0 6.5 6.0 5.5 5.0 4.5 4.0 3.5 3.0 2.5 2.0 1.5 1.0 0.5 0.0
f1 (ppm)

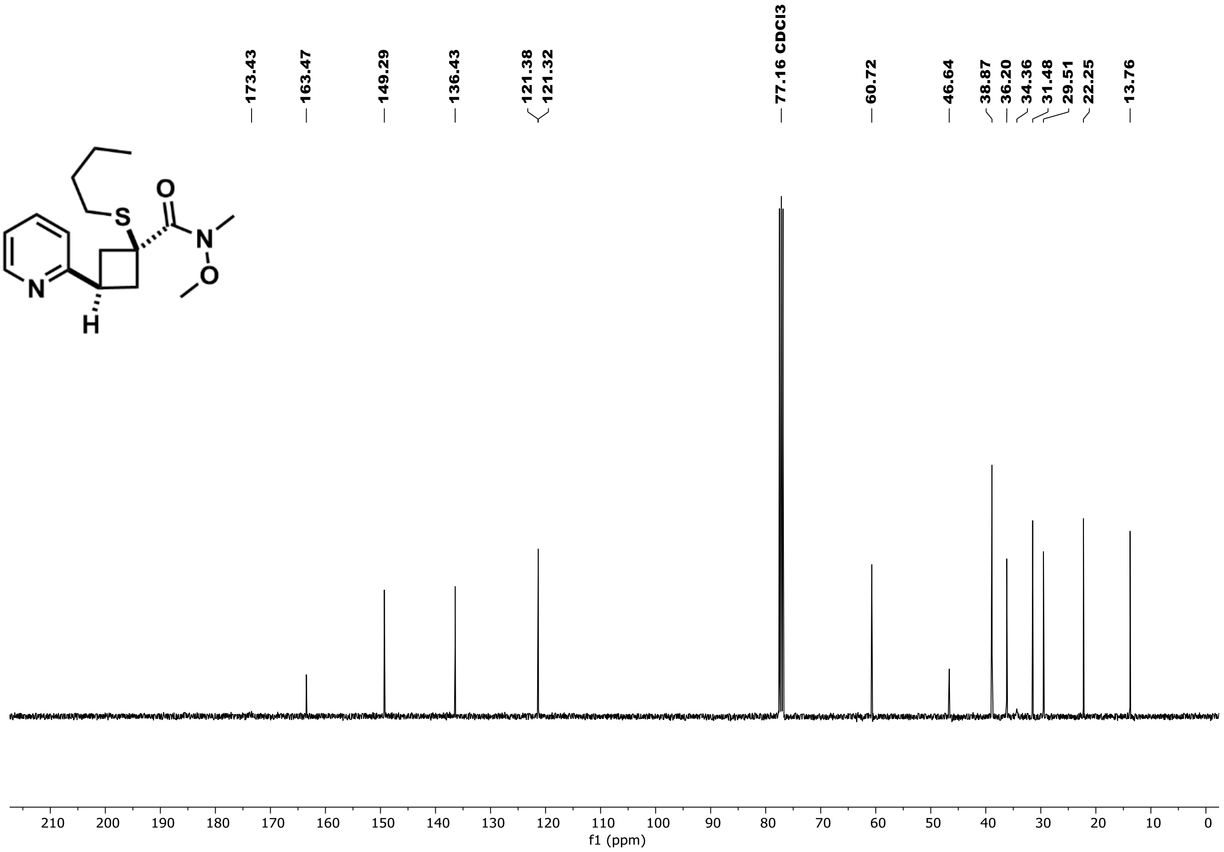

173.43
163.47
149.29
136.43
121.38
121.32
77.16 CDCl3
60.72
46.64
38.87
36.20
34.36
31.48
29.51
22.25
13.76
S
O
N
O
N
H
210 200 190 180 170 160 150 140 130 120 110 100 90 80 70 60 50 40 30 20 10 0
f1 (ppm)

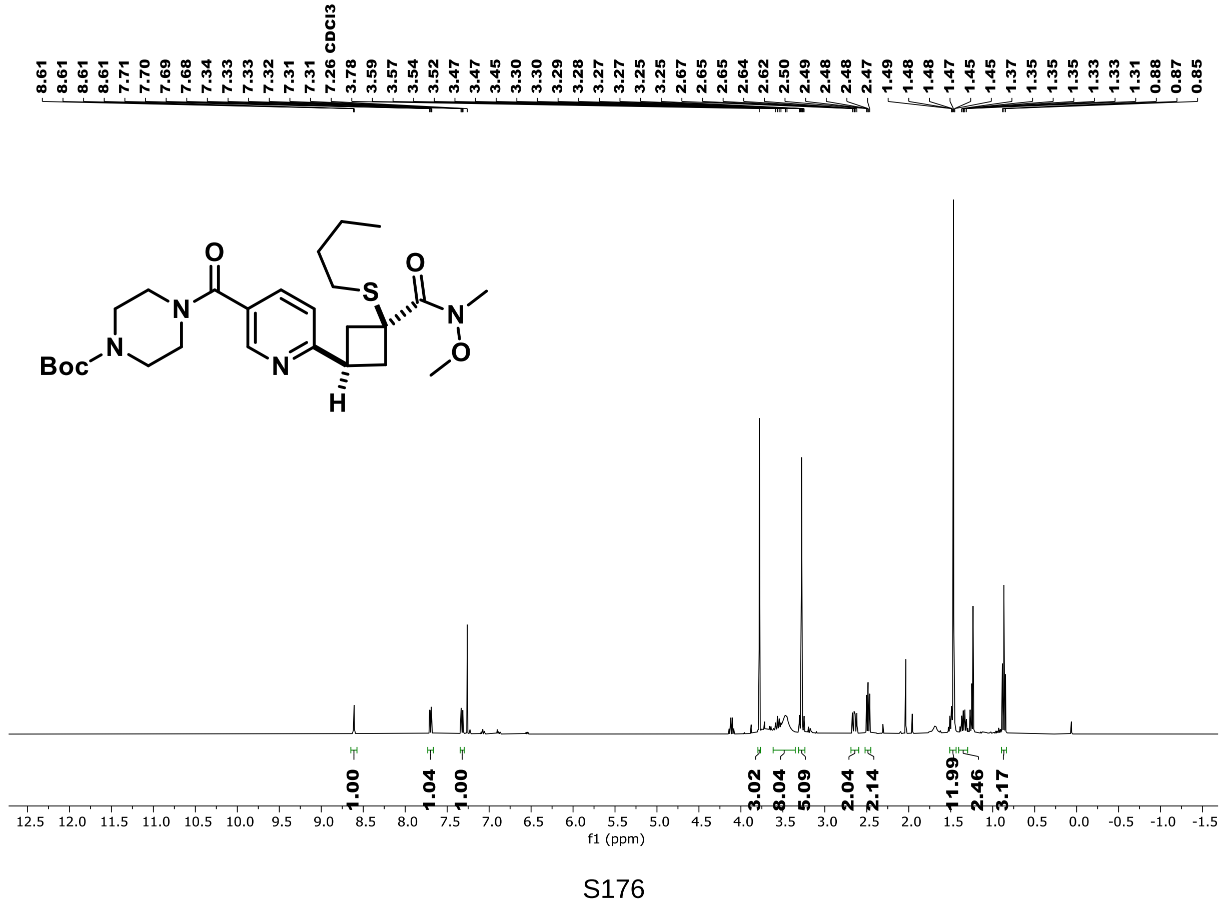

8.61 8.61 8.61 8.61 7.71 7.70 7.69 7.68 7.34 7.33 7.33 7.32 7.31 7.26 CDCl3 3.78 3.59 3.57 3.54 3.52 3.47 3.47 3.45 3.30 3.30 3.29 3.28 3.27 3.27 3.25 3.25 2.67 2.65 2.64 2.62 2.50 2.49 2.48 2.48 2.47 1.49 1.48 1.48 1.47 1.45 1.45 1.37 1.35 1.35 1.35 1.33 1.33 1.31 0.88 0.87 0.85
O
N
N
Boc
N
S
O
N
O
H
1.00
1.04
1.00
3.02
8.04
5.09
2.04
2.14
11.99
2.46
3.17
12.5 12.0 11.5 11.0 10.5 10.0 9.5 9.0 8.5 8.0 7.5 7.0 6.5 6.0 5.5 5.0 4.5 4.0 3.5 3.0 2.5 2.0 1.5 1.0 0.5 0.0 -0.5 -1.0 -1.5
f1 (ppm)

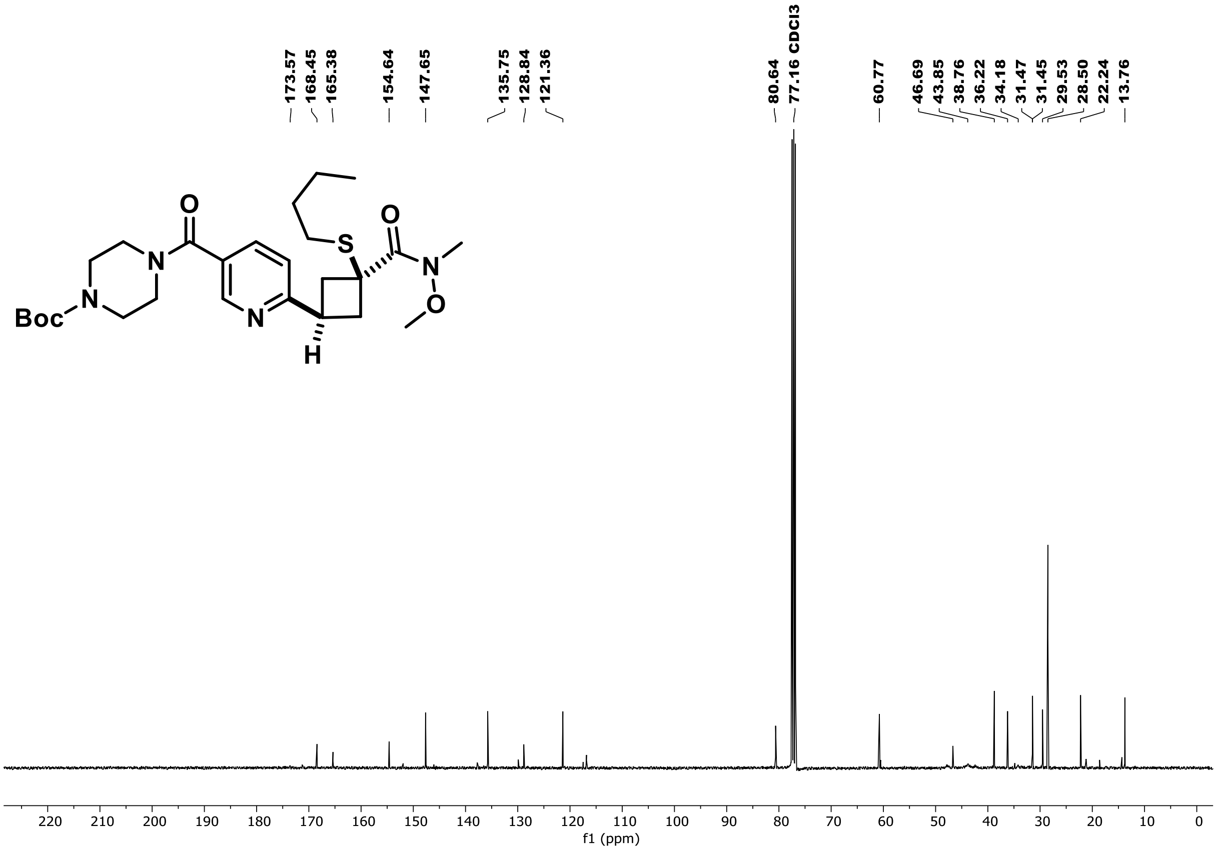
173.57
168.45
165.38
154.64
147.65
135.75
128.84
121.36
80.64
77.16 CDCl3
60.77
46.69
43.85
38.76
36.22
34.18
31.47
31.45
29.53
28.50
22.24
13.76
Boc
N
O
S
H
f1 (ppm)

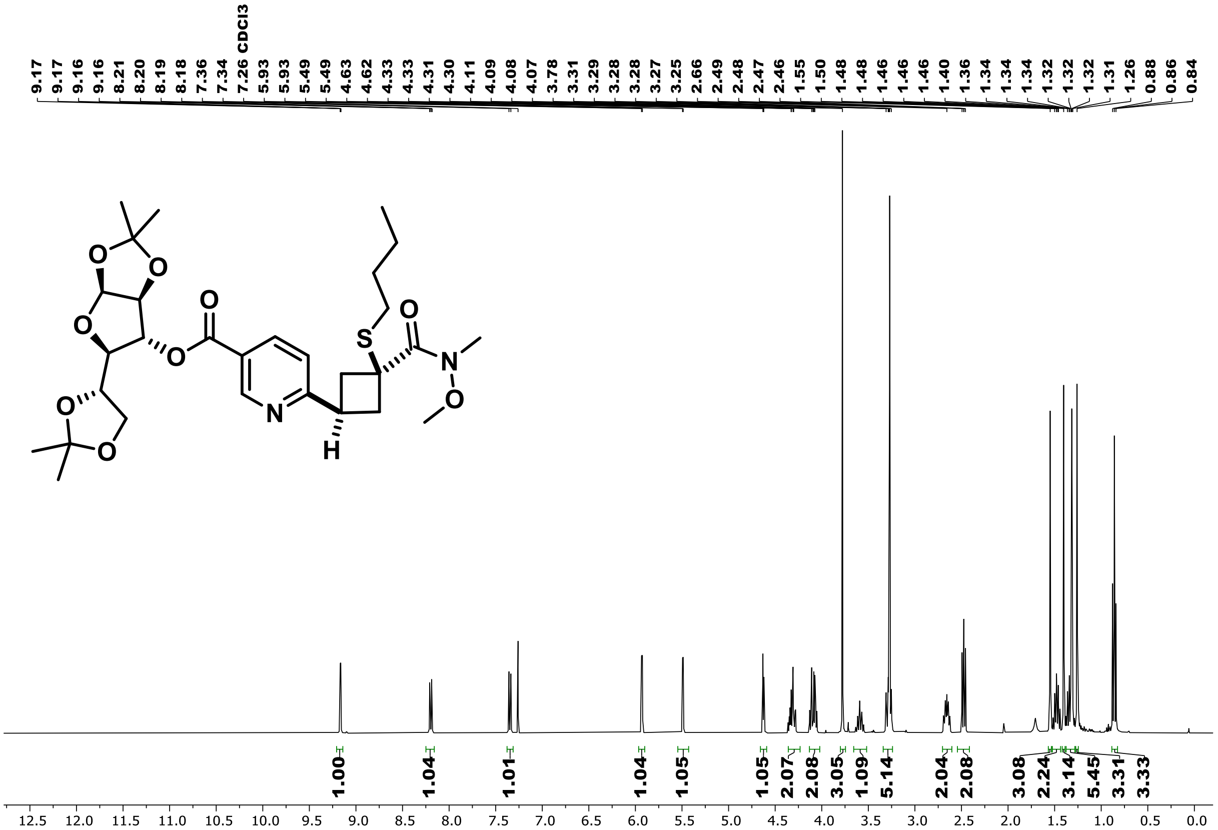
9.17
9.17
9.16
9.16
8.21
8.20
8.19
8.18
7.36
7.34
7.26 CDCl3
5.93
5.93
5.49
5.49
4.63
4.62
4.33
4.33
4.31
4.30
4.11
4.09
4.08
4.07
3.78
3.31
3.29
3.28
3.28
3.27
3.25
2.66
2.49
2.48
2.47
2.46
1.55
1.50
1.48
1.48
1.46
1.46
1.46
1.40
1.36
1.34
1.34
1.32
1.32
1.32
1.31
1.26
0.88
0.86
0.84
1.00
1.04
1.01
1.04
1.05
1.05
2.07
2.08
3.05
1.09
5.14
2.04
2.08
3.08
2.24
3.14
5.45
3.31
3.33
O
N
S
H
f1 (ppm)

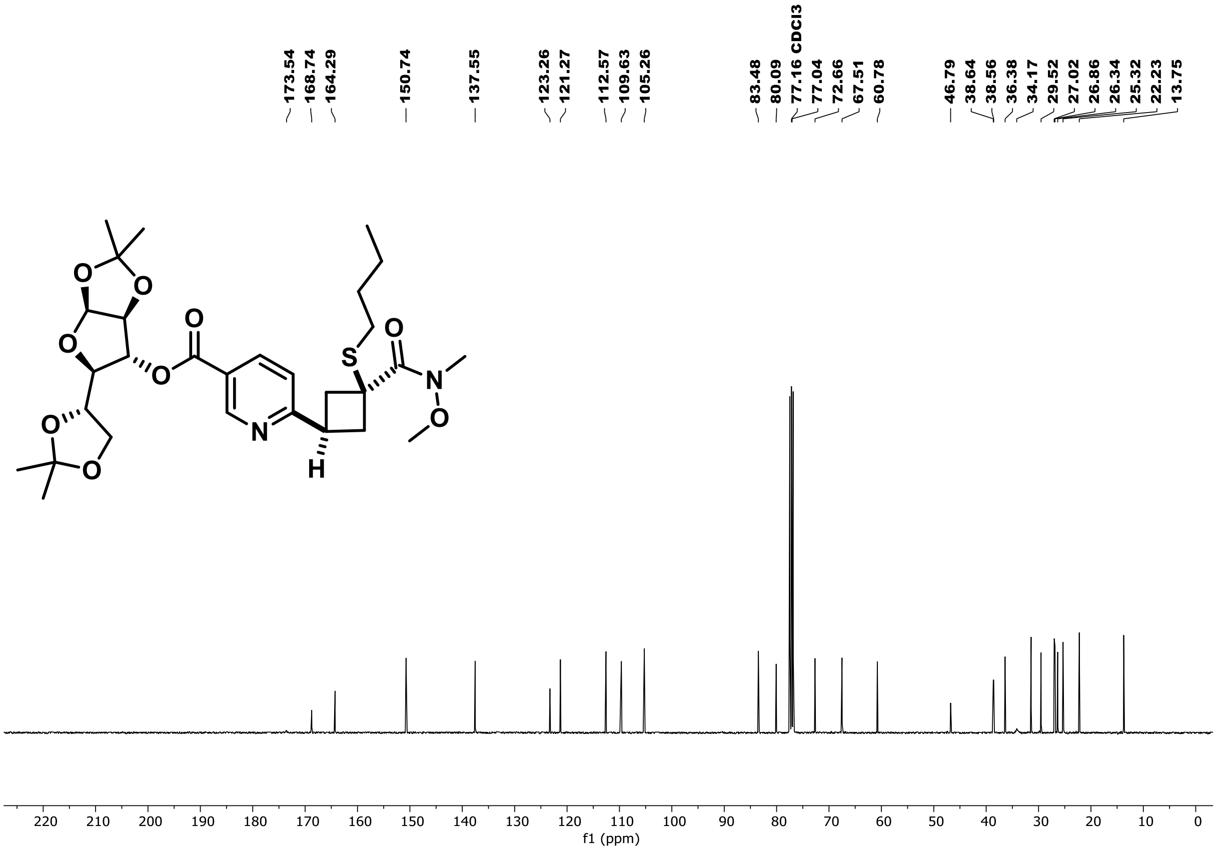
173.54
168.74
164.29
150.74
137.55
123.26
121.27
112.57
109.63
105.26
83.48
80.09
77.16 CDCl3
77.04
72.66
67.51
60.78
46.79
38.64
38.56
36.38
34.17
29.52
27.02
26.86
26.34
25.32
22.23
13.75
220 210 200 190 180 170 160 150 140 130 120 110 100 90 80 70 60 50 40 30 20 10 0
f1 (ppm)

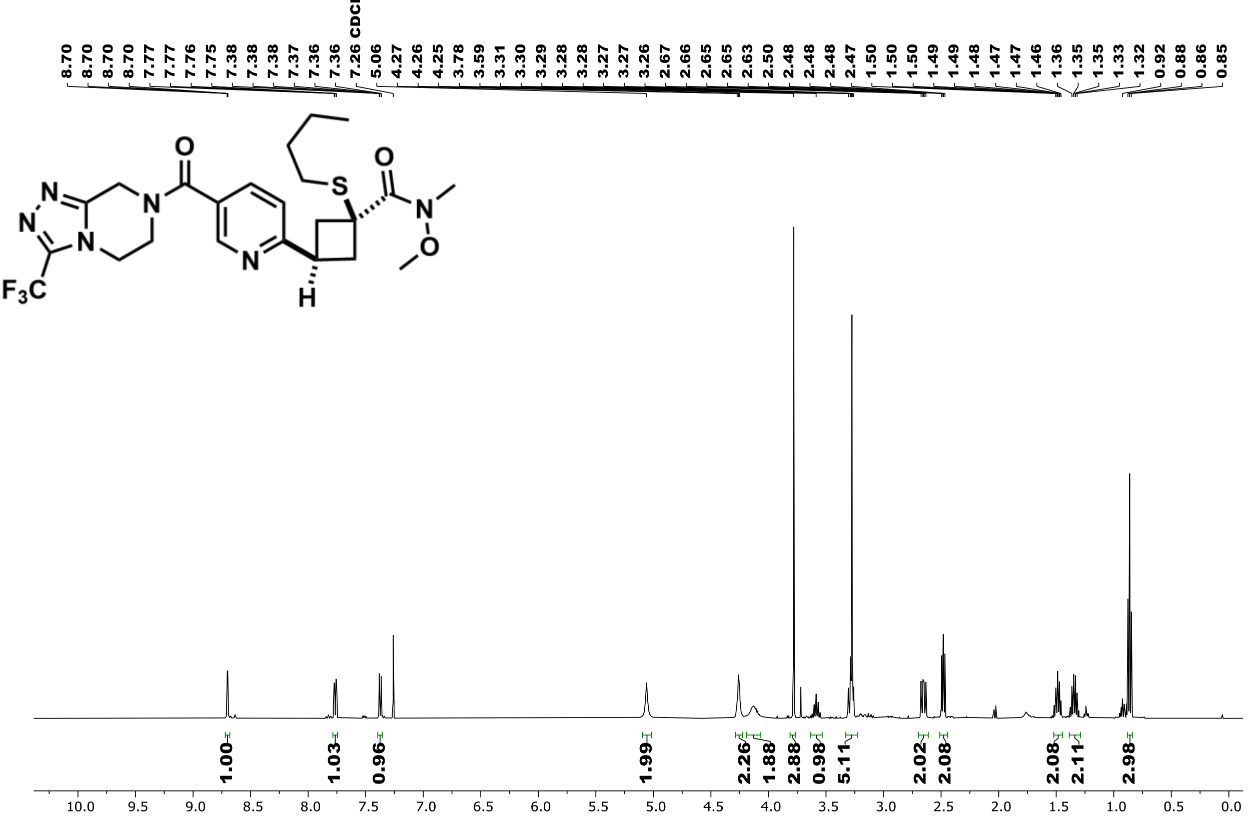
8.70
8.70
8.70
8.70
7.77
7.77
7.76
7.75
7.38
7.38
7.38
7.37
7.36
7.36
7.26 CDCl3
5.06
4.27
4.26
4.25
3.78
3.59
3.31
3.30
3.29
3.28
3.28
3.27
3.27
3.26
2.67
2.66
2.65
2.65
2.63
2.50
2.48
2.48
2.48
2.47
1.50
1.50
1.50
1.49
1.49
1.48
1.47
1.47
1.46
1.36
1.35
1.35
1.33
1.32
0.92
0.88
0.86
0.85
F3C
H
1.00
1.03
0.96
1.99
2.26
1.88
2.88
0.98
5.11
2.02
2.08
2.08
2.11
2.98
10.0 9.5 9.0 8.5 8.0 7.5 7.0 6.5 6.0 5.5 5.0 4.5 4.0 3.5 3.0 2.5 2.0 1.5 1.0 0.5 0.0
f1 (ppm)

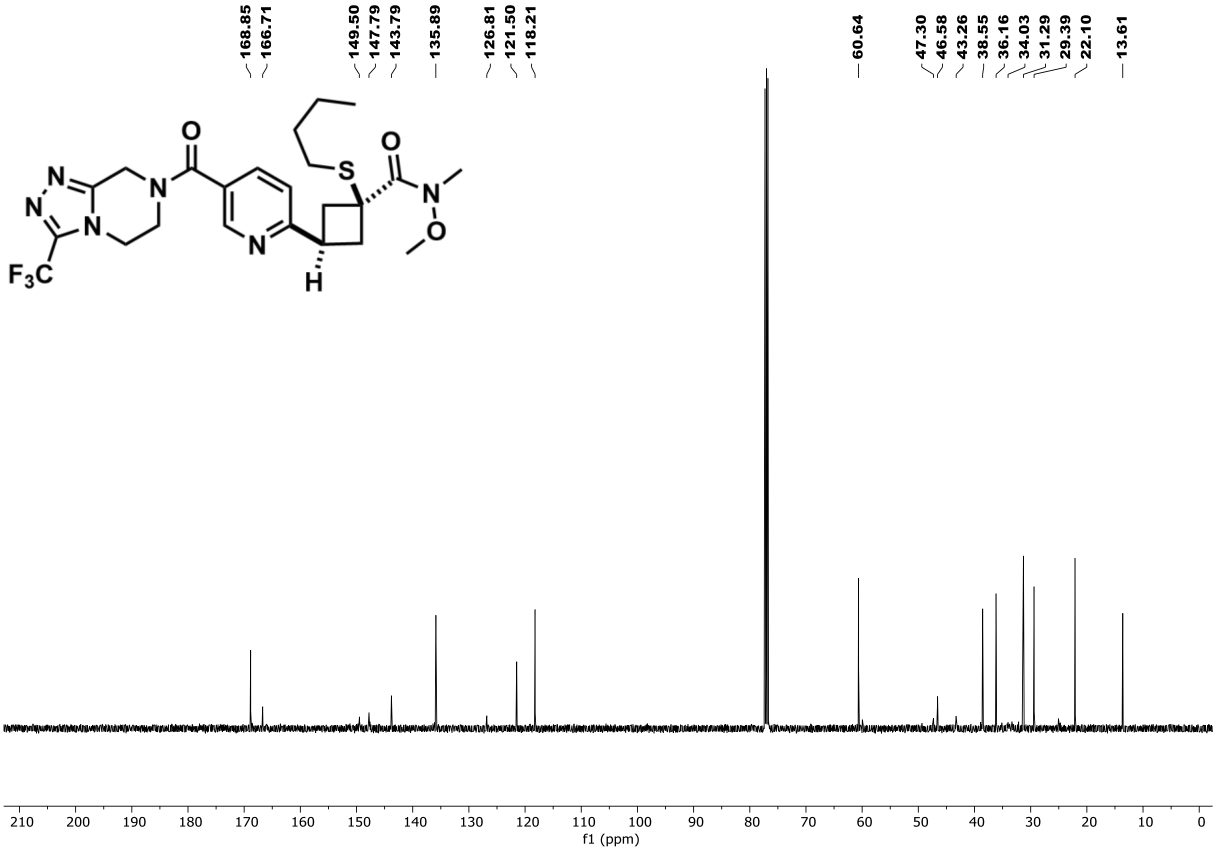
168.85
166.71
149.50
147.79
143.79
135.89
126.81
121.50
118.21
60.64
47.30
46.58
43.26
38.55
36.16
34.03
31.29
29.39
22.10
13.61
f1 (ppm)

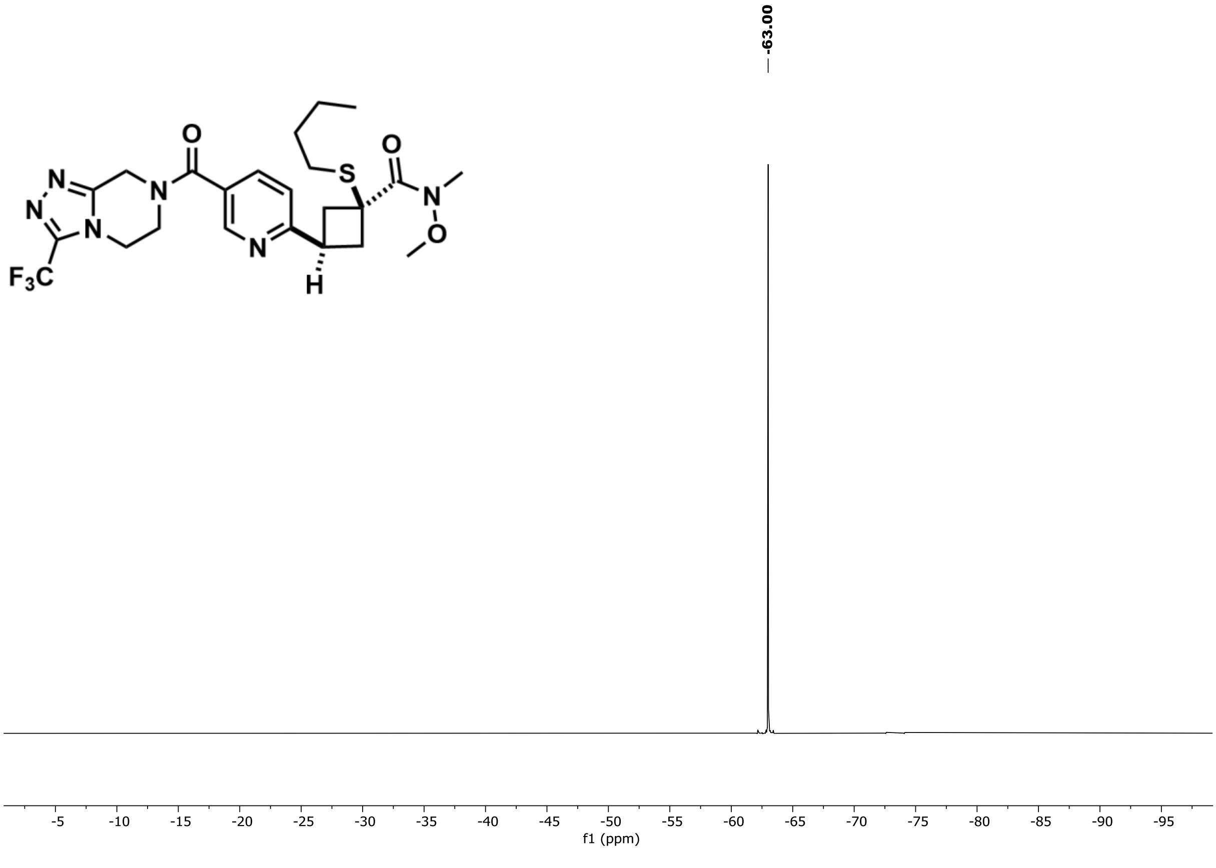
-63.00
f1 (ppm)

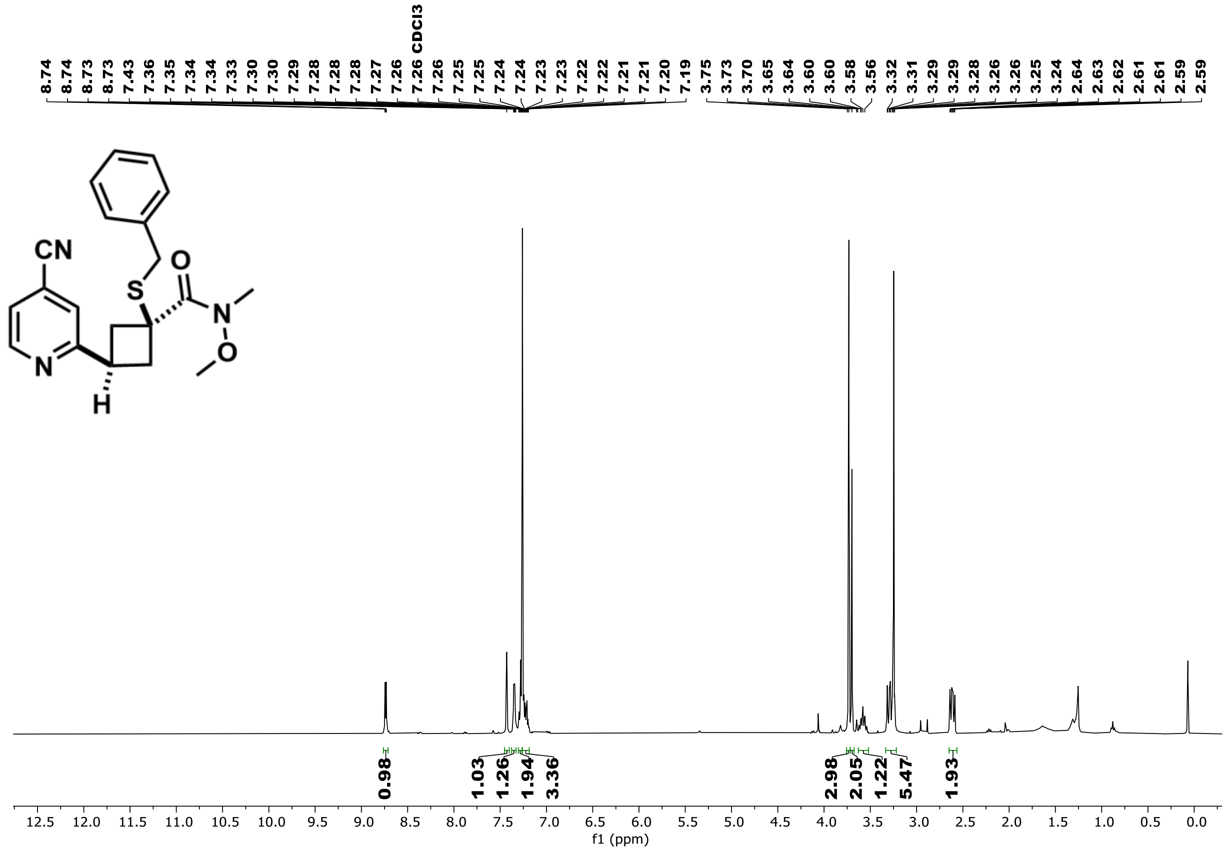
8.74 8.74 8.73 8.73 7.43 7.36 7.35 7.34 7.34 7.33 7.30 7.30 7.29 7.28 7.28 7.28 7.27 7.26 CDCl3 7.26 7.26 7.25 7.25 7.24 7.24 7.24 7.23 7.23 7.22 7.22 7.21 7.21 7.20 7.19 3.75 3.73 3.70 3.65 3.64 3.60 3.60 3.58 3.56 3.32 3.31 3.29 3.29 3.28 3.26 3.26 3.25 3.24 2.64 2.63 2.62 2.61 2.59 2.59
CN
S
O
N
O
N
H
0.98
1.03
1.26
1.94
3.36
2.98
2.05
1.22
5.47
1.93
12.5 12.0 11.5 11.0 10.5 10.0 9.5 9.0 8.5 8.0 7.5 7.0 6.5 6.0 5.5 5.0 4.5 4.0 3.5 3.0 2.5 2.0 1.5 1.0 0.5 0.0
f1 (ppm)

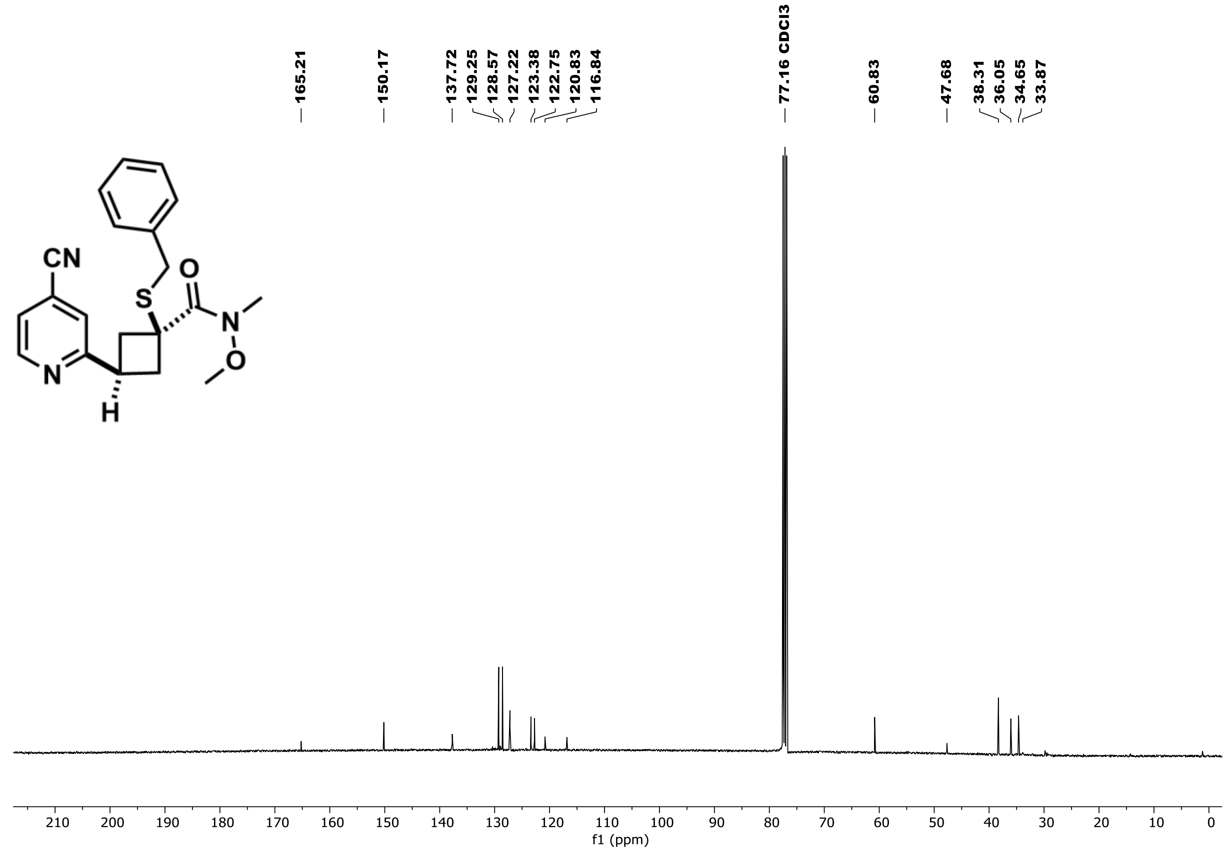
165.21
150.17
137.72 129.25 128.57 127.22 123.38 122.75 120.83 116.84
77.16 CDCl3
60.83
47.68
38.31 36.05 34.65 33.87
CN
S
O
N
O
N
H
210 200 190 180 170 160 150 140 130 120 110 100 90 80 70 60 50 40 30 20 10 0
f1 (ppm)

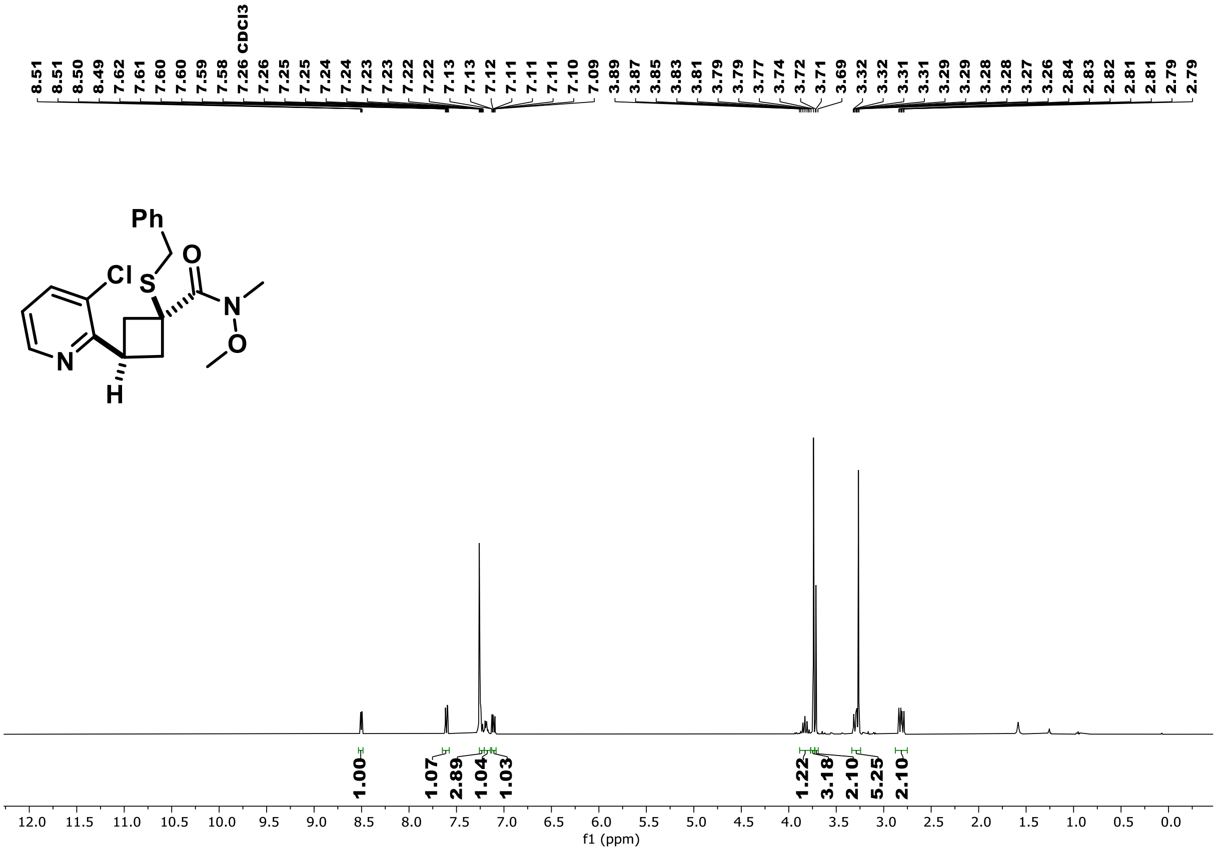
8.51 8.51 8.50 8.49 7.62 7.61 7.60 7.60 7.59 7.58 7.26 CDCl3 7.26 7.25 7.24 7.24 7.23 7.23 7.22 7.22 7.13 7.13 7.12 7.11 7.11 7.11 7.10 7.09 3.89 3.87 3.85 3.83 3.81 3.79 3.79 3.77 3.74 3.72 3.71 3.69 3.32 3.32 3.31 3.31 3.29 3.29 3.28 3.28 3.27 3.26 2.84 2.83 2.82 2.81 2.79 2.79
Ph
O
Cl
S
N
N
O
H
1.00
1.07
2.89
1.04
1.03
1.22
3.18
2.10
5.25
2.10
12.0 11.5 11.0 10.5 10.0 9.5 9.0 8.5 8.0 7.5 7.0 6.5 6.0 5.5 5.0 4.5 4.0 3.5 3.0 2.5 2.0 1.5 1.0 0.5 0.0
f1 (ppm)

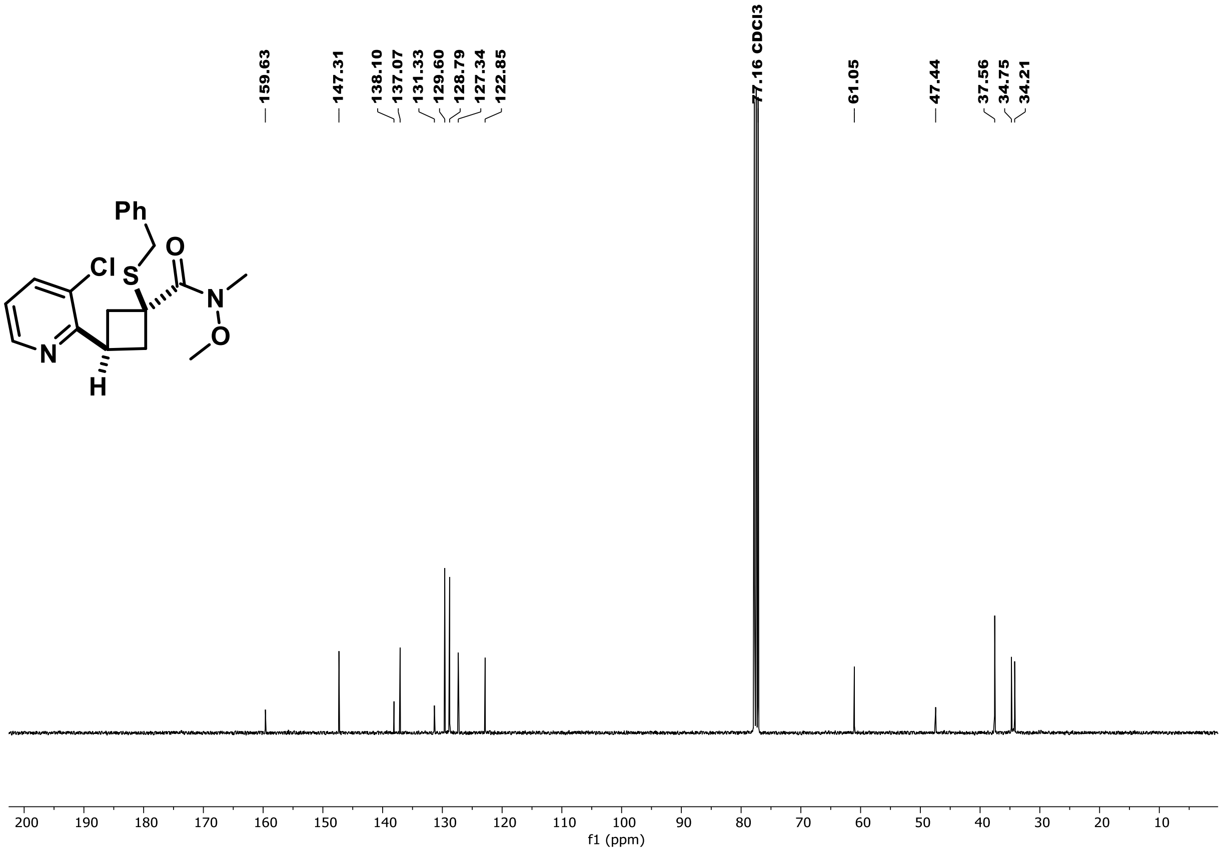
159.63
147.31
138.10 137.07 131.33 129.60 128.79 127.34 122.85
77.16 CDCl3
61.05
47.44
37.56 34.75 34.21
Ph
O
Cl
S
N
N
O
H
200 190 180 170 160 150 140 130 120 110 100 90 80 70 60 50 40 30 20 10
f1 (ppm)

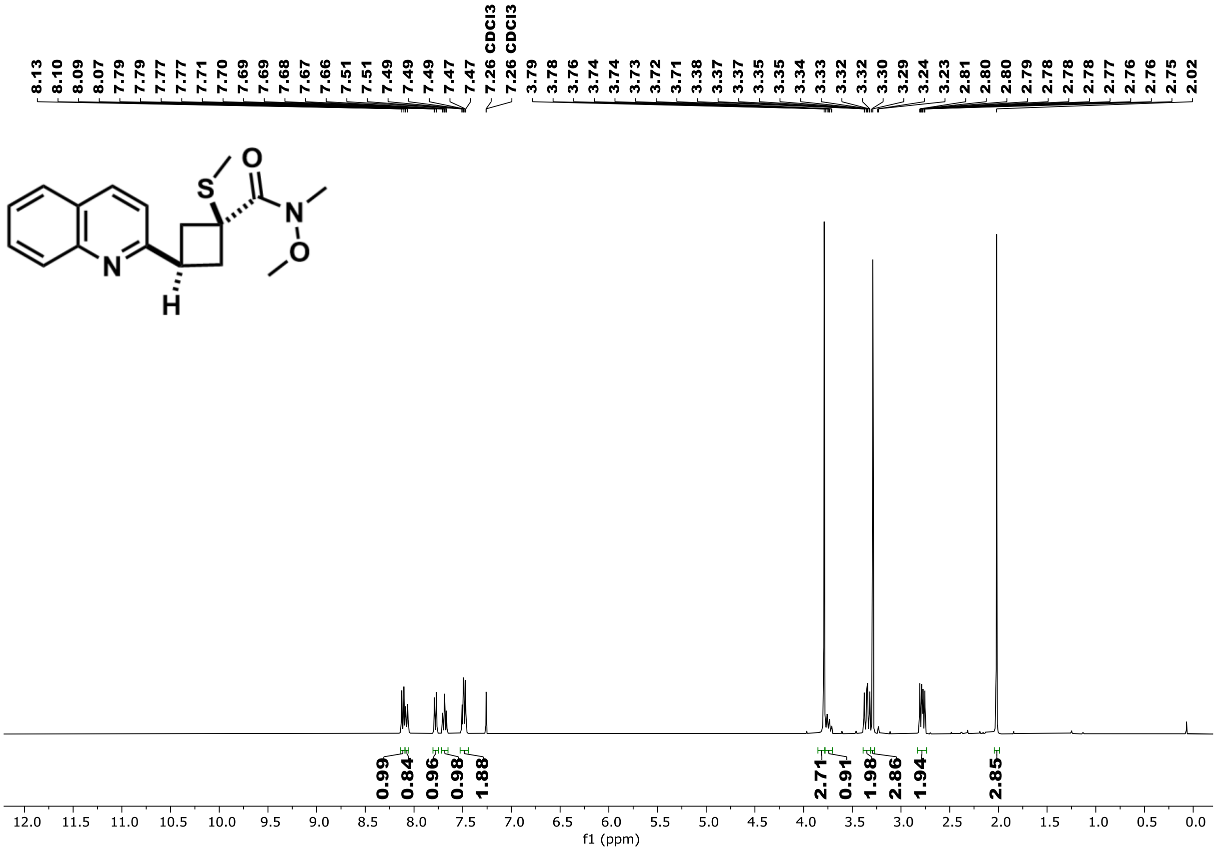
8.13 8.10 8.09 8.07 7.79 7.79 7.77 7.77 7.71 7.70 7.69 7.69 7.68 7.67 7.66 7.51 7.51 7.49 7.49 7.49 7.47 7.47 7.26 CDCl3 7.26 CDCl3 3.79 3.78 3.76 3.74 3.74 3.73 3.72 3.71 3.38 3.37 3.35 3.35 3.34 3.33 3.32 3.32 3.30 3.29 3.24 3.23 2.81 2.80 2.80 2.79 2.78 2.78 2.77 2.76 2.76 2.75 2.02
S
O
N
O
N
H
0.99 0.84 0.96 0.98 1.88 2.71 0.91 1.98 2.86 1.94 2.85
12.0 11.5 11.0 10.5 10.0 9.5 9.0 8.5 8.0 7.5 7.0 6.5 6.0 5.5 5.0 4.5 4.0 3.5 3.0 2.5 2.0 1.5 1.0 0.5 0.0
f1 (ppm)

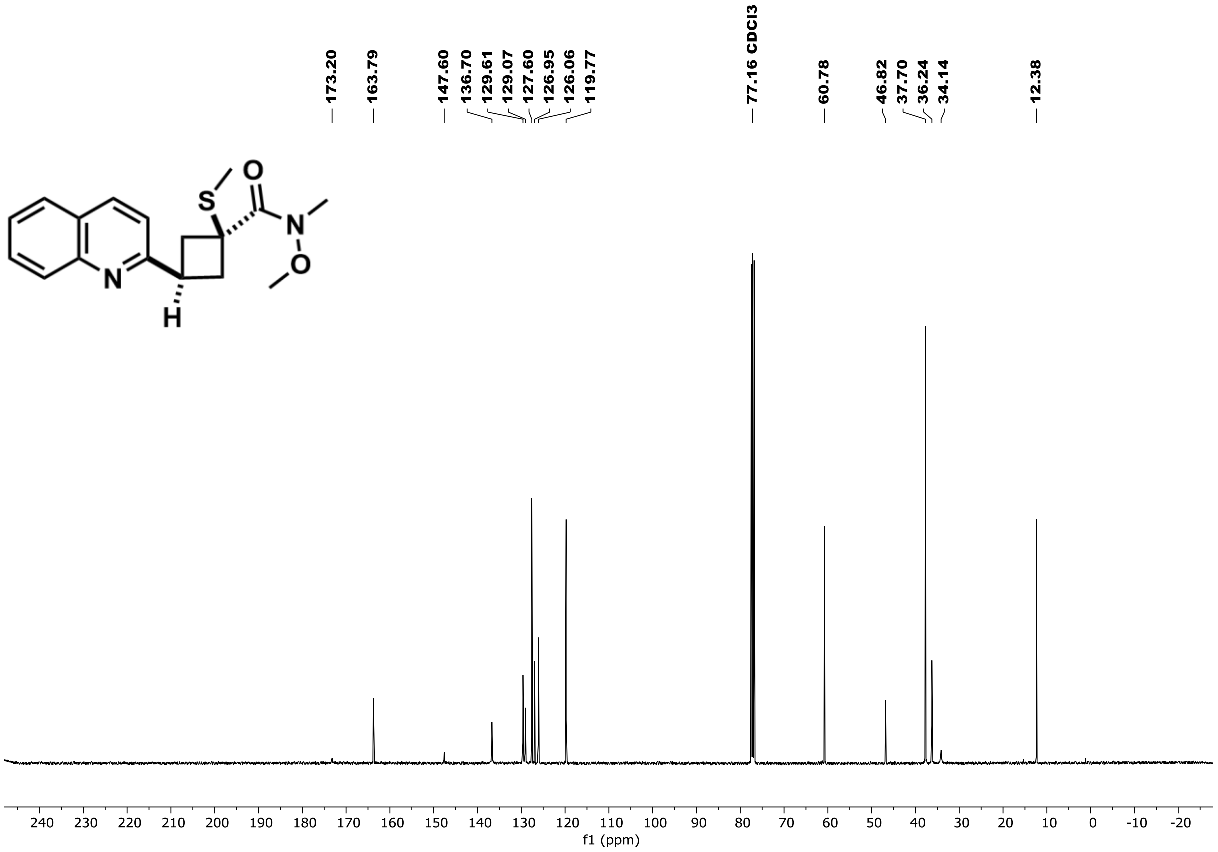
173.20 163.79 147.60 136.70 129.61 129.07 127.60 126.95 126.06 119.77 77.16 CDCl3 60.78 46.82 37.70 36.24 34.14 12.38
S
O
N
O
N
H
240 230 220 210 200 190 180 170 160 150 140 130 120 110 100 90 80 70 60 50 40 30 20 10 0 -10 -20
f1 (ppm)

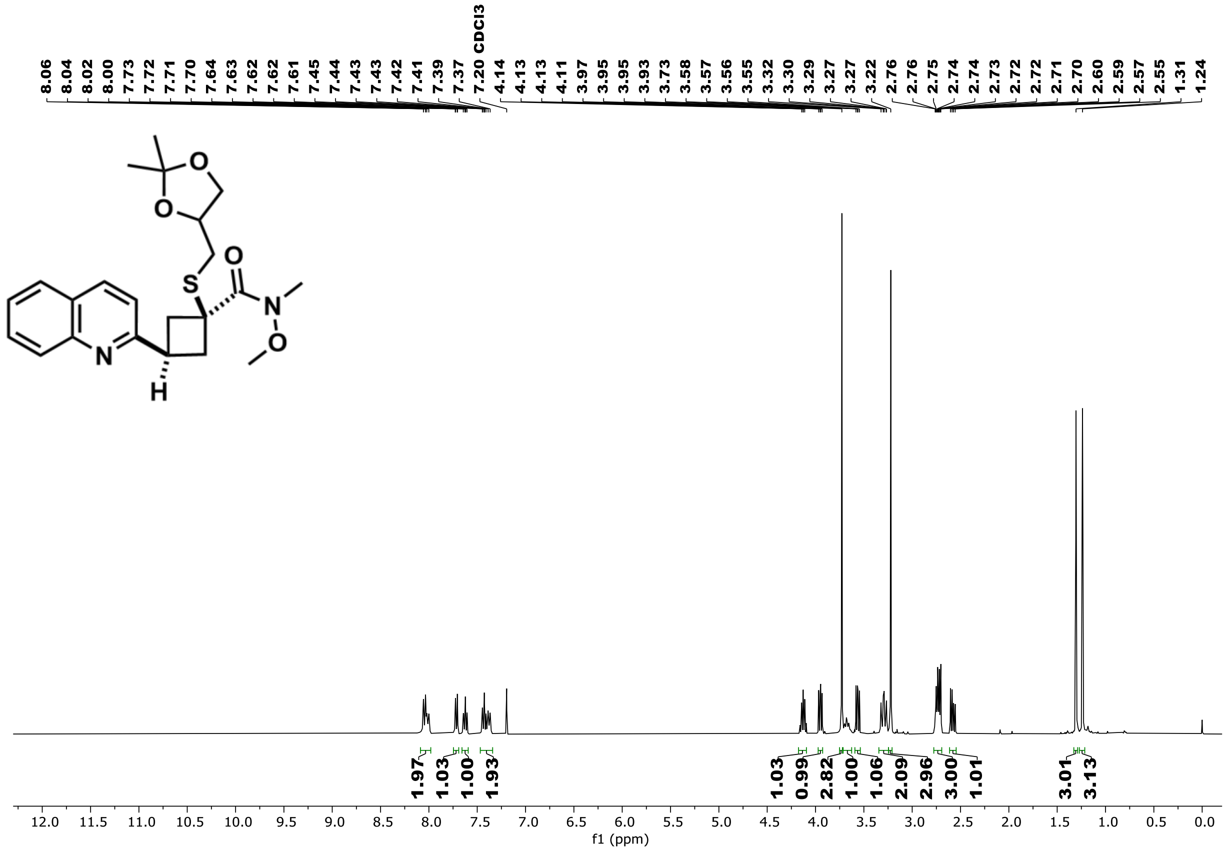

8.06
8.04
8.02
8.00
7.73
7.72
7.71
7.70
7.64
7.63
7.62
7.62
7.61
7.45
7.44
7.43
7.43
7.42
7.41
7.39
7.37
7.20 CDCl3
4.14
4.13
4.13
4.11
3.97
3.95
3.95
3.93
3.73
3.58
3.57
3.56
3.55
3.32
3.30
3.29
3.27
3.27
3.22
2.76
2.76
2.75
2.74
2.74
2.73
2.72
2.72
2.71
2.70
2.60
2.59
2.57
2.55
1.31
1.24
O
O
S
O
N
O
N
H
1.97
1.03
1.00
1.93
1.03
0.99
2.82
1.00
1.06
2.09
2.96
3.00
1.01
3.01
3.13
12.0
11.5
11.0
10.5
10.0
9.5
9.0
8.5
8.0
7.5
7.0
6.5
6.0
5.5
5.0
4.5
4.0
3.5
3.0
2.5
2.0
1.5
1.0
0.5
0.0
f1 (ppm)

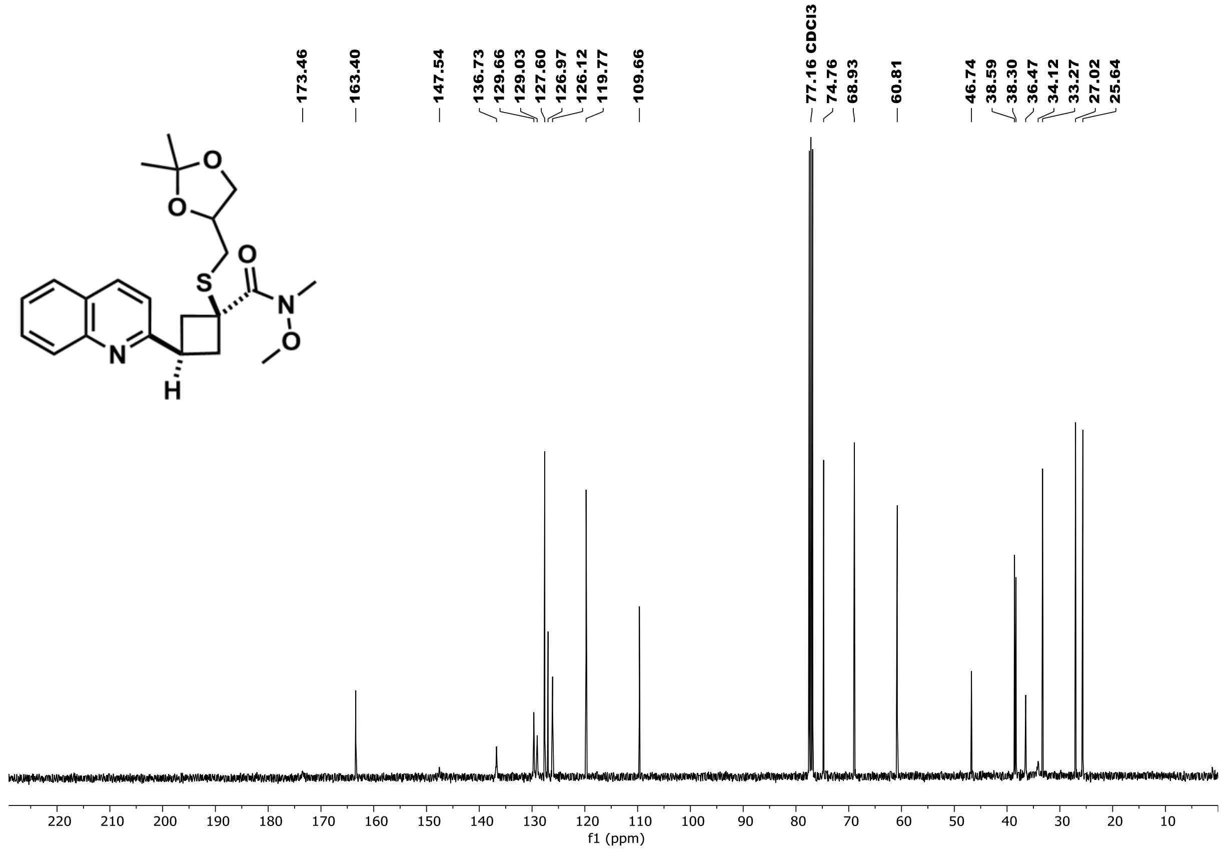

173.46
163.40
147.54
136.73
129.66
129.03
127.60
126.97
126.12
119.77
109.66
77.16 CDCl3
74.76
68.93
60.81
46.74
38.59
38.30
36.47
34.12
33.27
27.02
25.64
O
O
S
O
N
O
N
H
220
210
200
190
180
170
160
150
140
130
120
110
100
90
80
70
60
50
40
30
20
10
f1 (ppm)

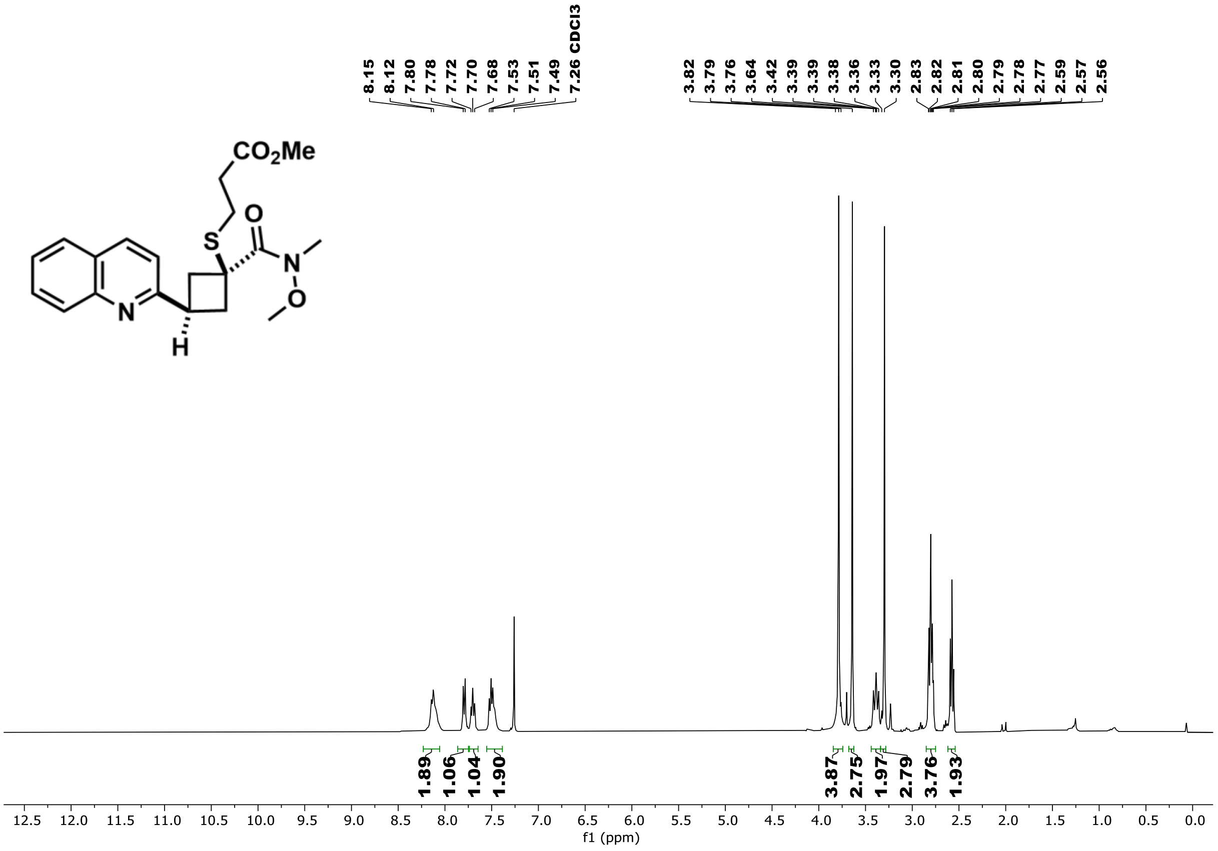
8.15
8.12
7.80
7.78
7.72
7.70
7.68
7.53
7.51
7.49
7.26 CDCl3
3.82
3.79
3.76
3.64
3.42
3.39
3.39
3.38
3.36
3.33
3.30
2.83
2.82
2.81
2.80
2.79
2.78
2.77
2.59
2.57
2.56
CO2Me
S
O
N
O
N
H
1.89
1.06
1.04
1.90
3.87
2.75
1.97
2.79
3.76
1.93
f1 (ppm)

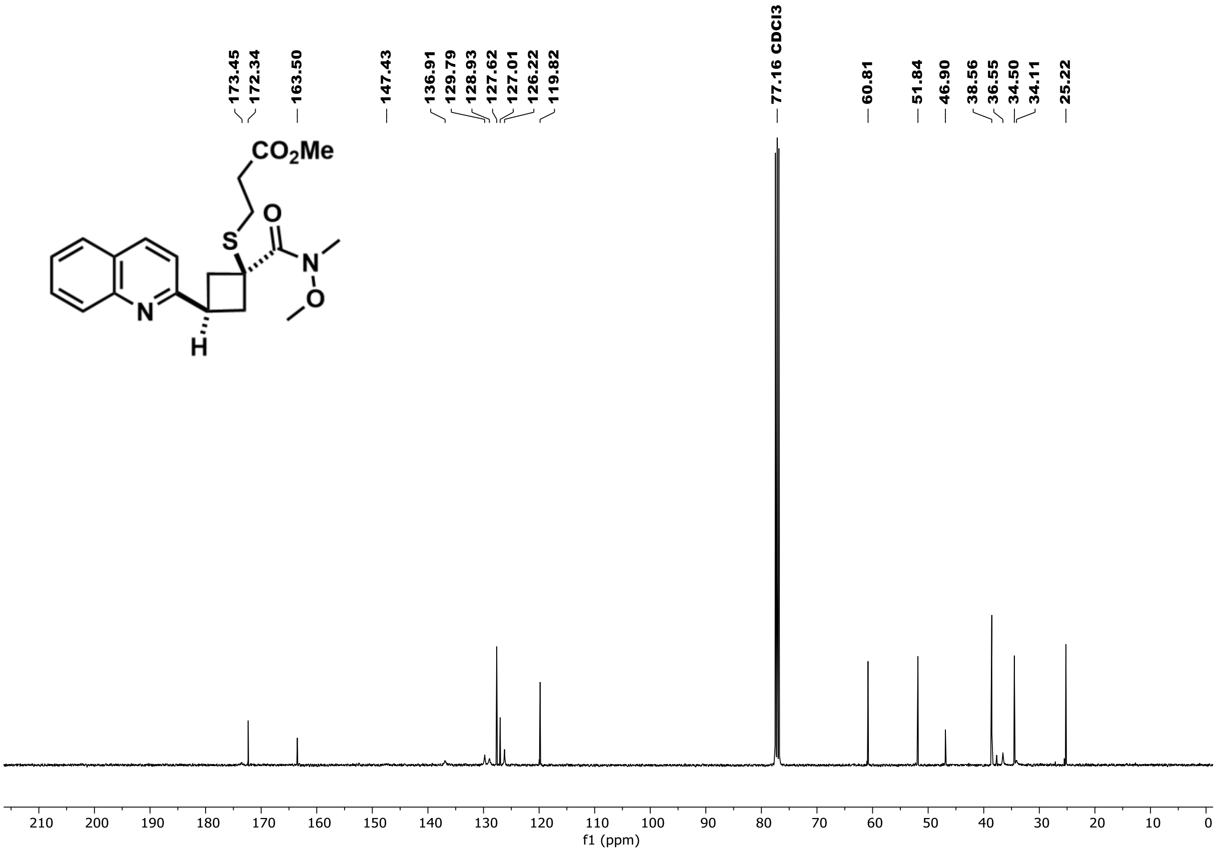
173.45
172.34
163.50
147.43
136.91
129.79
128.93
127.62
127.01
126.22
119.82
77.16 CDCl3
60.81
51.84
46.90
38.56
36.55
34.50
34.11
25.22
CO2Me
S
O
N
O
N
H
f1 (ppm)

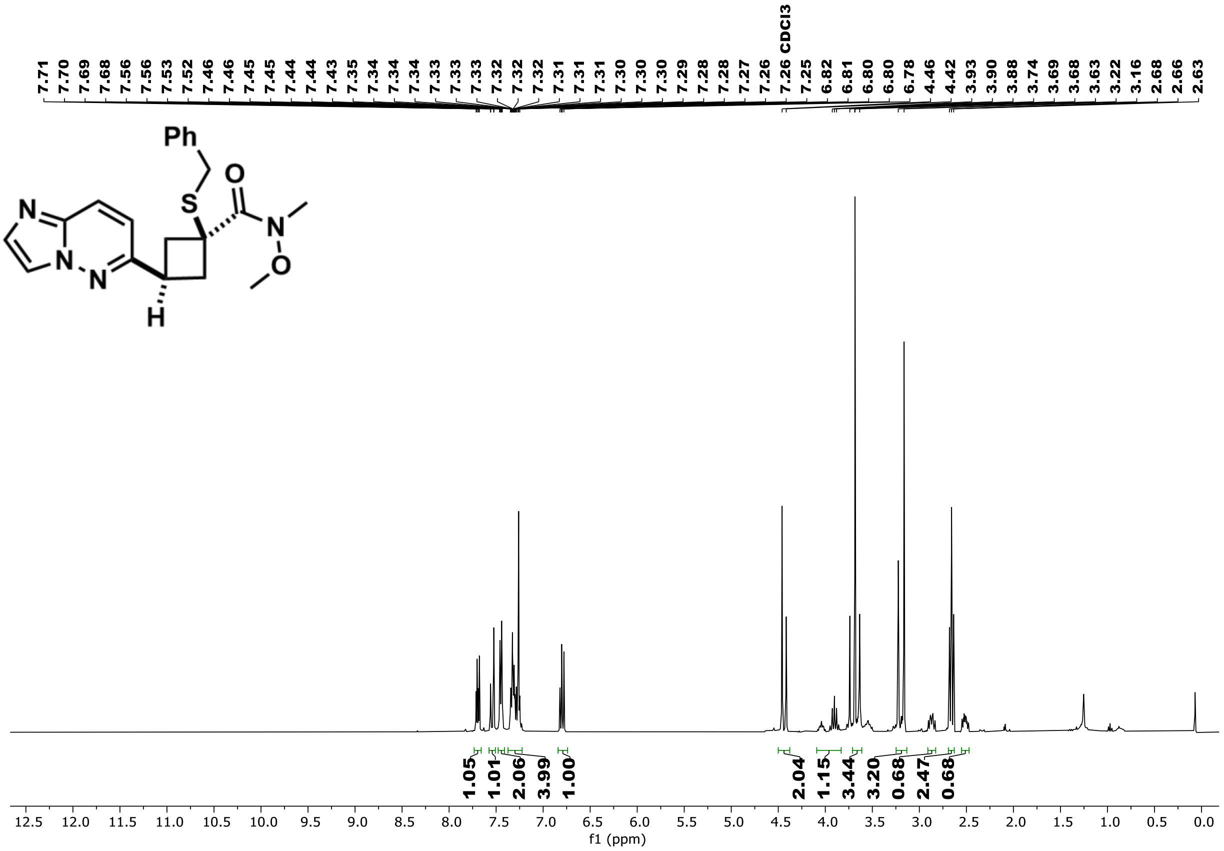
7.71 7.70 7.69 7.68 7.56 7.56 7.53 7.52 7.46 7.46 7.45 7.45 7.44 7.44 7.43 7.35 7.34 7.34 7.34 7.33 7.33 7.33 7.32 7.32 7.32 7.31 7.31 7.31 7.30 7.30 7.30 7.29 7.28 7.28 7.27 7.26 7.26 CDCl3 7.25 6.82 6.81 6.80 6.80 6.78 4.46 4.42 3.93 3.90 3.88 3.74 3.69 3.68 3.63 3.22 3.16 2.68 2.66 2.63
Ph
S
O
N
N
N
N
O
H
1.05 1.01 2.06 3.99 1.00
2.04 1.15 3.44 3.20 0.68 2.47 0.68
12.5 12.0 11.5 11.0 10.5 10.0 9.5 9.0 8.5 8.0 7.5 7.0 6.5 6.0 5.5 5.0 4.5 4.0 3.5 3.0 2.5 2.0 1.5 1.0 0.5 0.0
f1 (ppm)

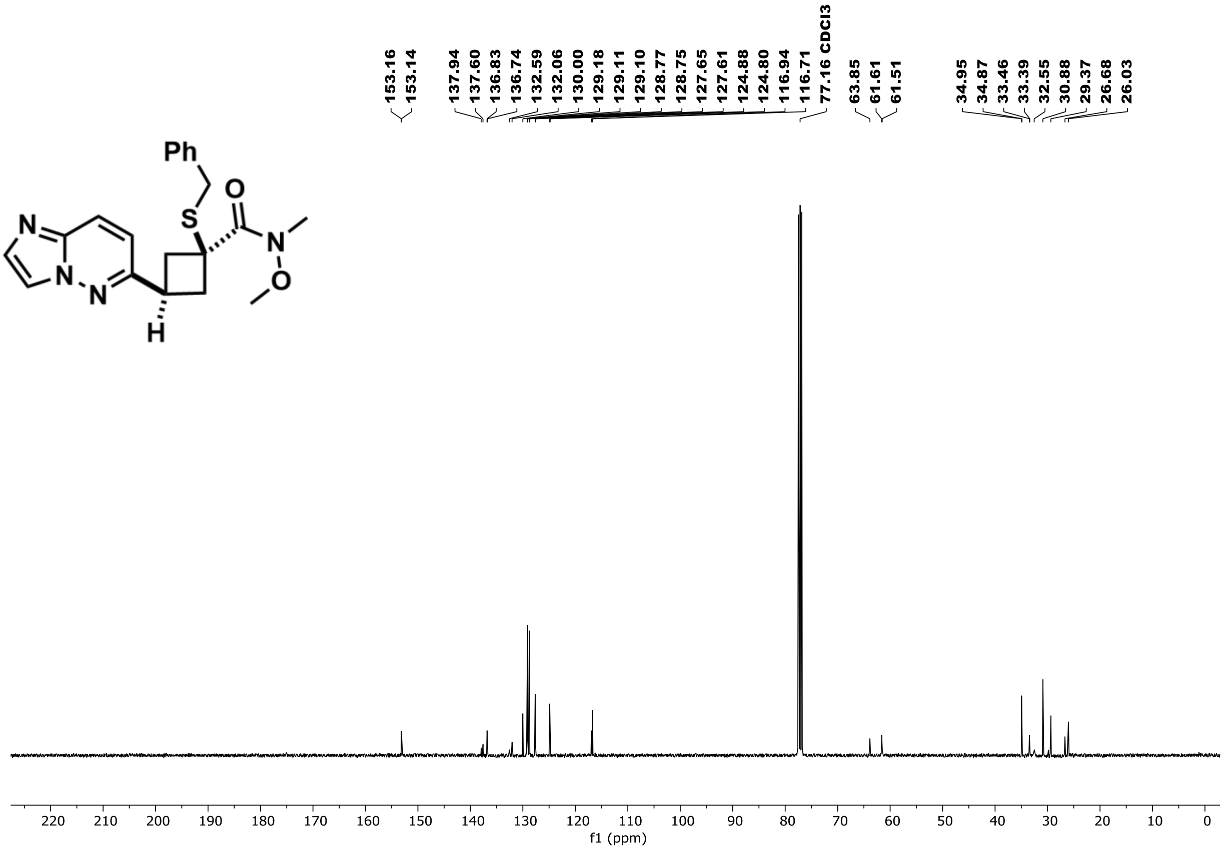
153.16 153.14 137.94 137.60 136.83 136.74 132.59 132.06 130.00 129.18 129.11 129.10 128.77 128.75 127.65 127.61 124.88 124.80 116.94 116.71 77.16 CDCl3 63.85 61.61 61.51 34.95 34.87 33.46 33.39 32.55 30.88 29.37 26.68 26.03
Ph
S
O
N
N
N
N
O
H
220 210 200 190 180 170 160 150 140 130 120 110 100 90 80 70 60 50 40 30 20 10 0
f1 (ppm)

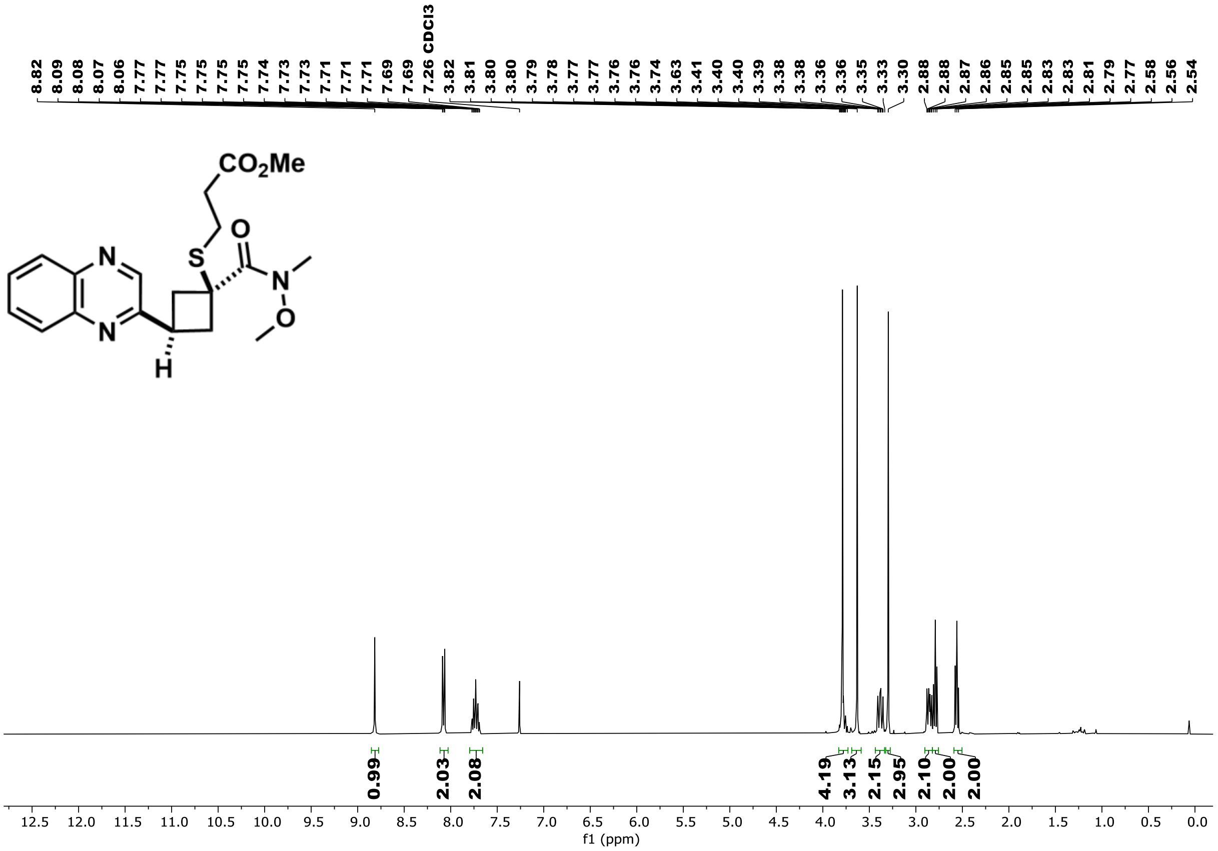

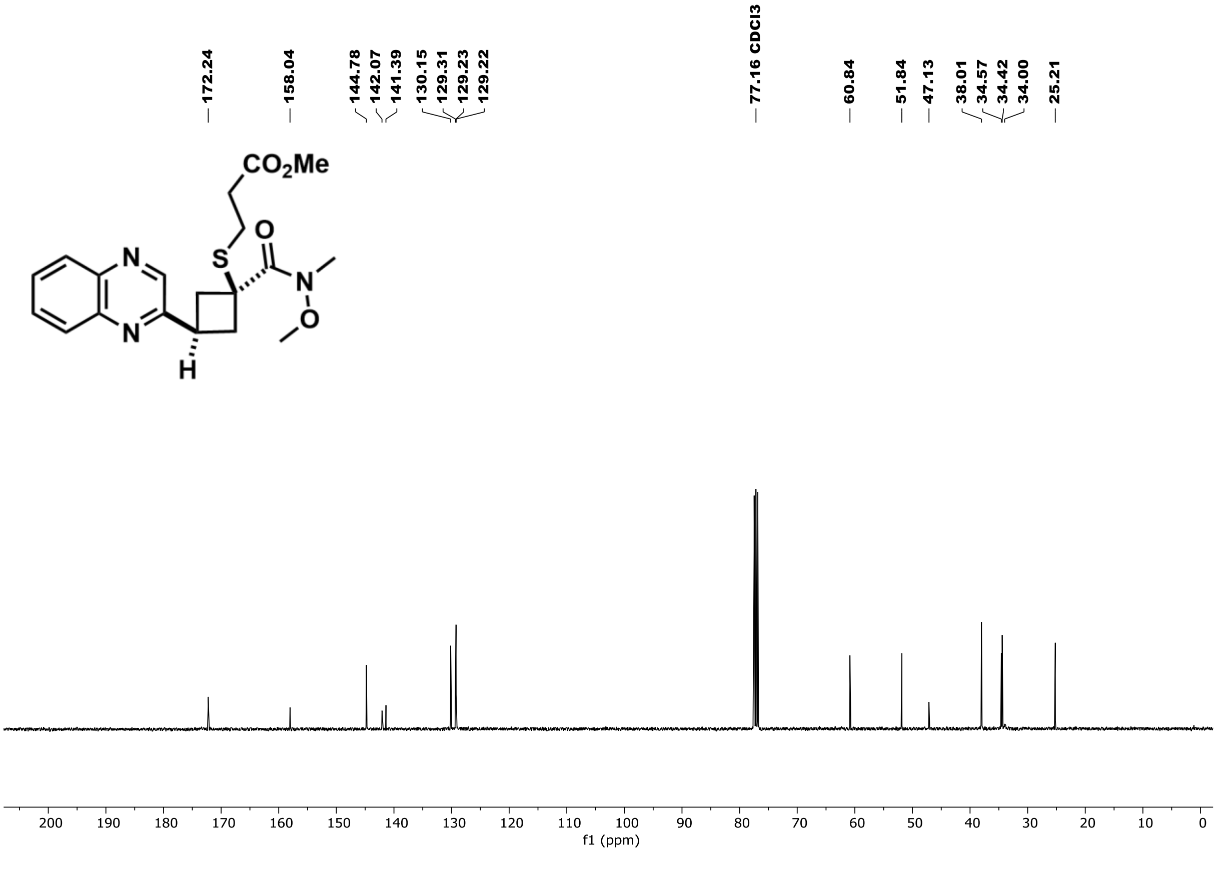

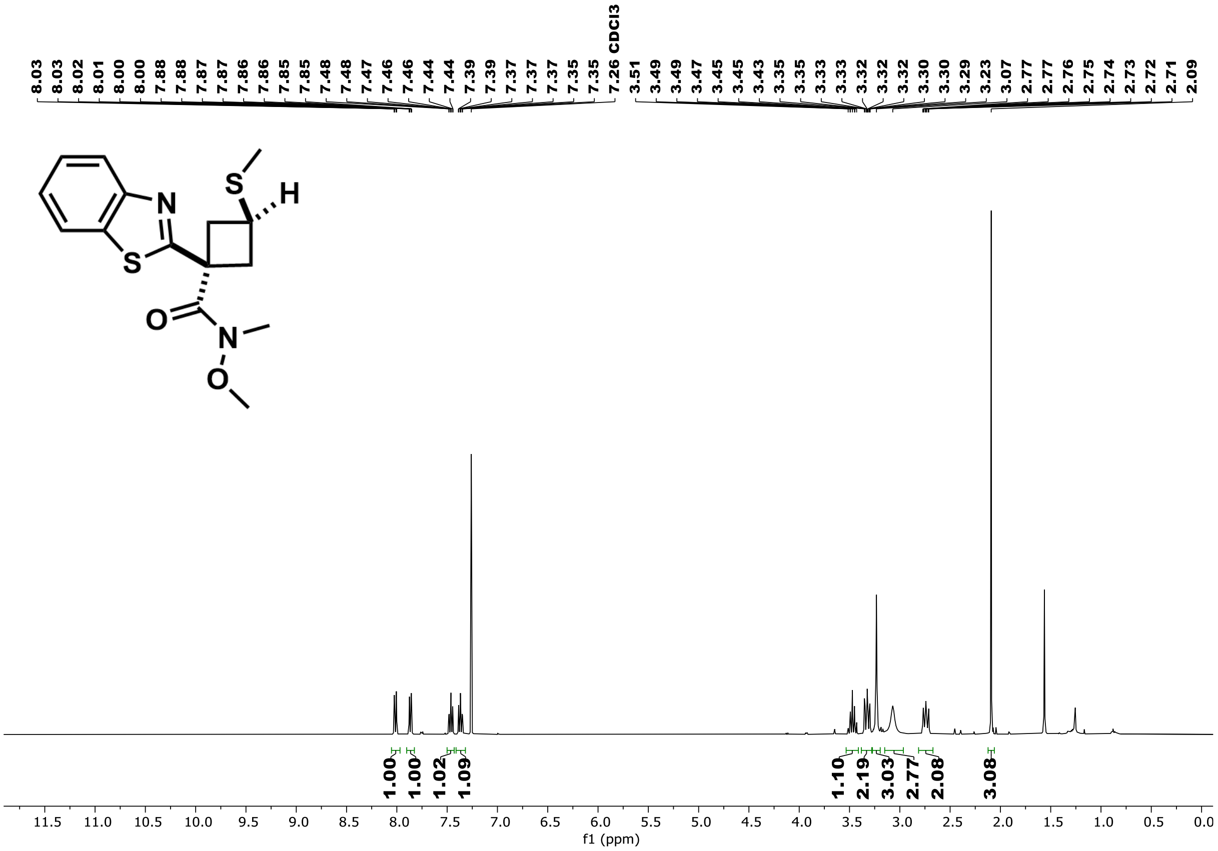
8.03 8.03 8.02 8.01 8.00 7.88 7.88 7.87 7.87 7.86 7.86 7.85 7.85 7.48 7.48 7.47 7.46 7.46 7.44 7.44 7.39 7.39 7.37 7.37 7.37 7.35 7.35 7.26 CDCl3 3.51 3.49 3.49 3.47 3.45 3.45 3.43 3.35 3.35 3.33 3.33 3.32 3.32 3.32 3.30 3.30 3.29 3.23 3.07 2.77 2.77 2.76 2.75 2.74 2.73 2.72 2.71 2.09
N
S
H
O
1.00
1.00
1.02
1.09
1.10
2.19
3.03
2.77
2.08
3.08
f1 (ppm)

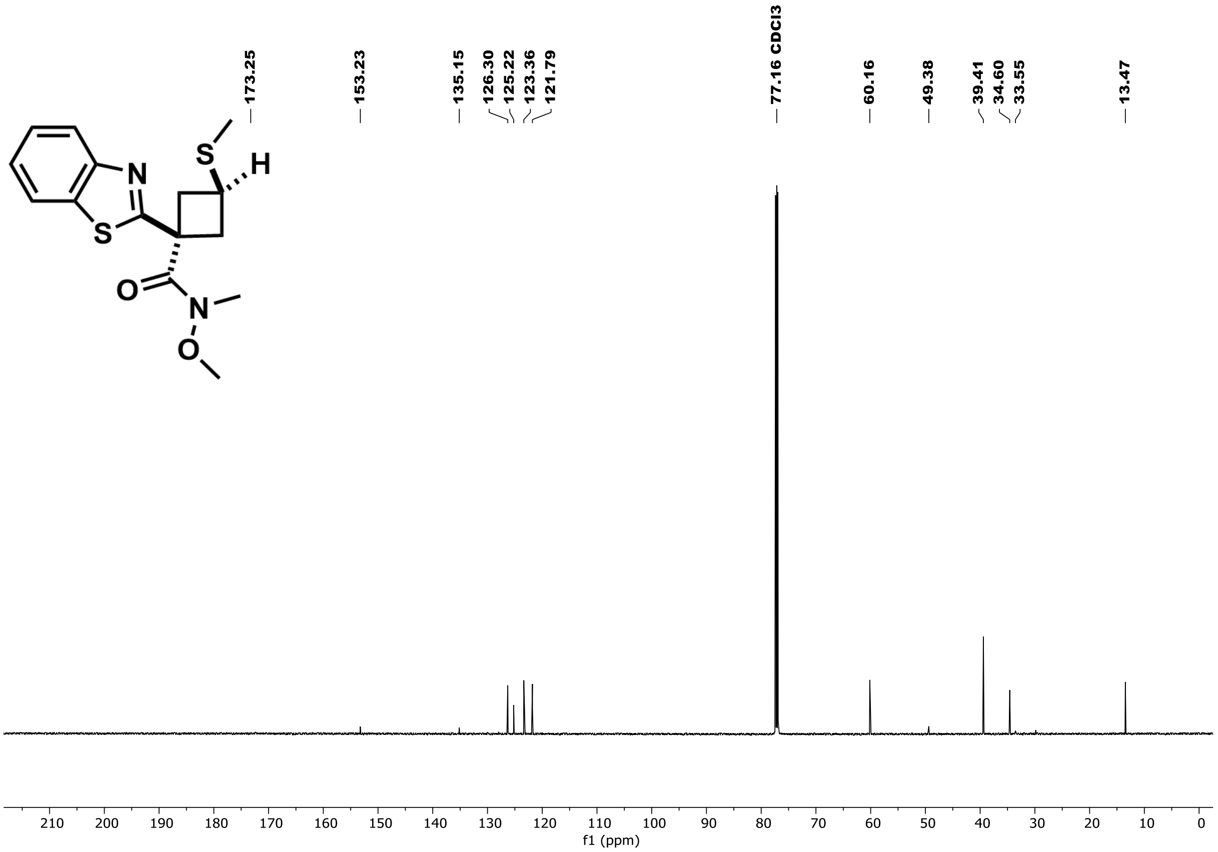
173.25
153.23
135.15
126.30
125.22
123.36
121.79
77.16 CDCl3
60.16
49.38
39.41
34.60
33.55
13.47
N
S
H
O
f1 (ppm)

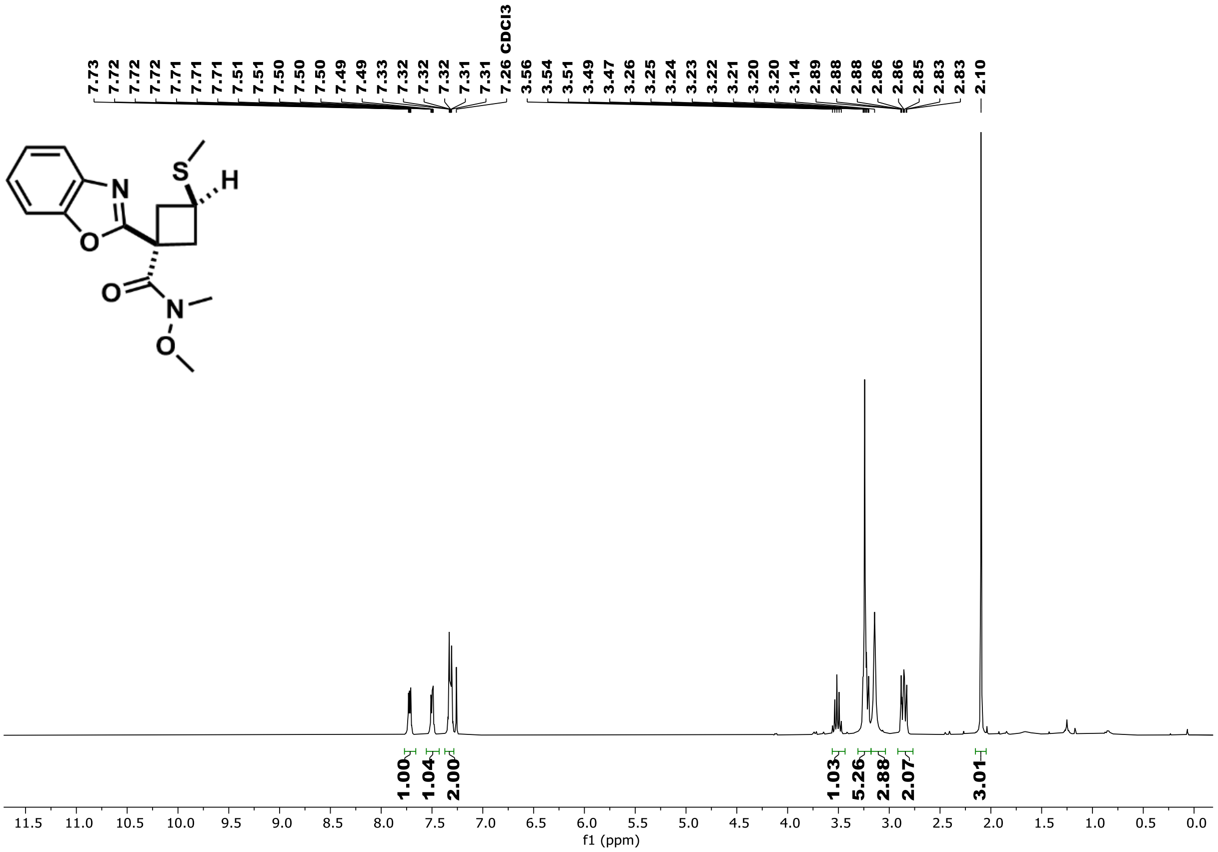
7.73 7.72 7.72 7.72 7.71 7.71 7.71 7.51 7.51 7.50 7.50 7.49 7.49 7.33 7.32 7.32 7.32 7.31 7.31 7.26 CDCl3 3.56 3.54 3.51 3.49 3.47 3.26 3.25 3.24 3.23 3.22 3.21 3.20 3.20 3.14 2.89 2.88 2.88 2.86 2.86 2.85 2.83 2.83 2.10
1.00 1.04 2.00 1.03 5.26 2.88 2.07 3.01
11.5 11.0 10.5 10.0 9.5 9.0 8.5 8.0 7.5 7.0 6.5 6.0 5.5 5.0 4.5 4.0 3.5 3.0 2.5 2.0 1.5 1.0 0.5 0.0
f1 (ppm)

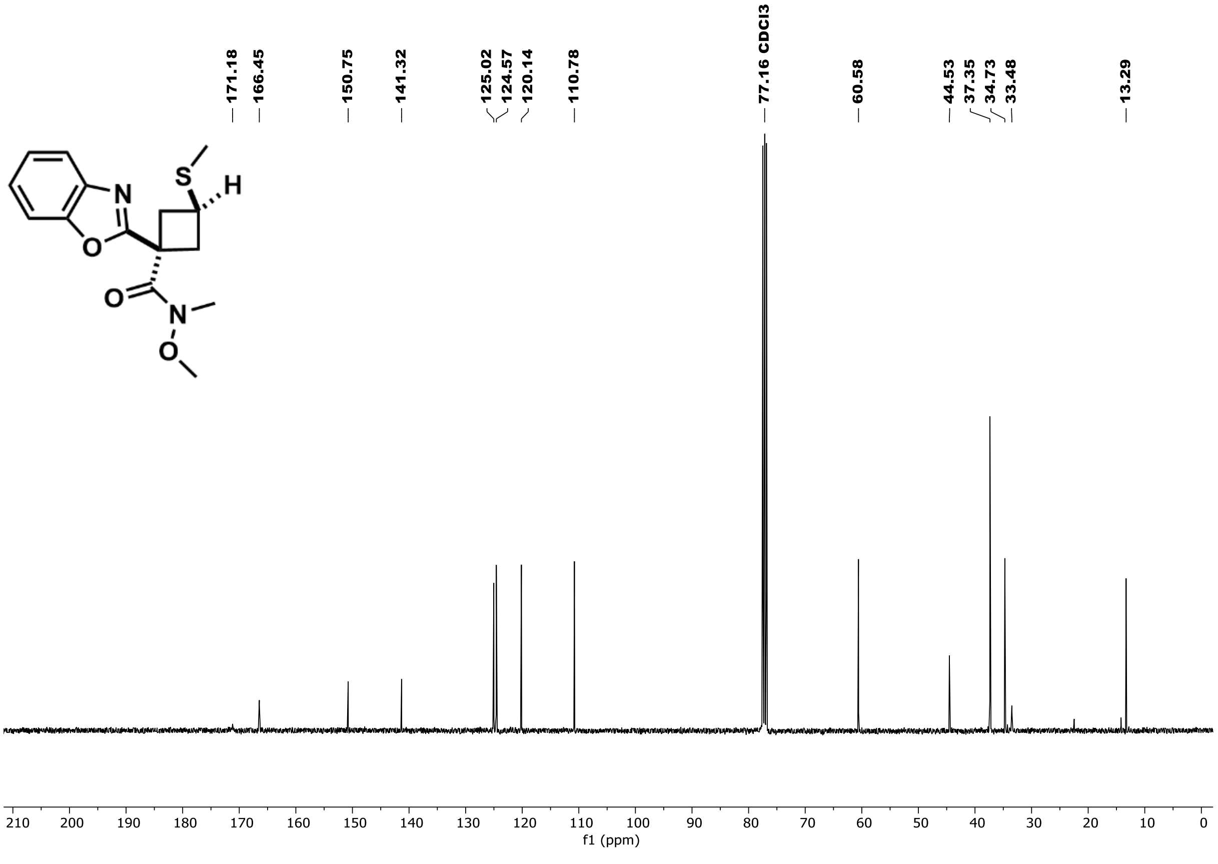
171.18 166.45 150.75 141.32 125.02 124.57 120.14 110.78 77.16 CDCl3 60.58 44.53 37.35 34.73 33.48 13.29
210 200 190 180 170 160 150 140 130 120 110 100 90 80 70 60 50 40 30 20 10 0
f1 (ppm)

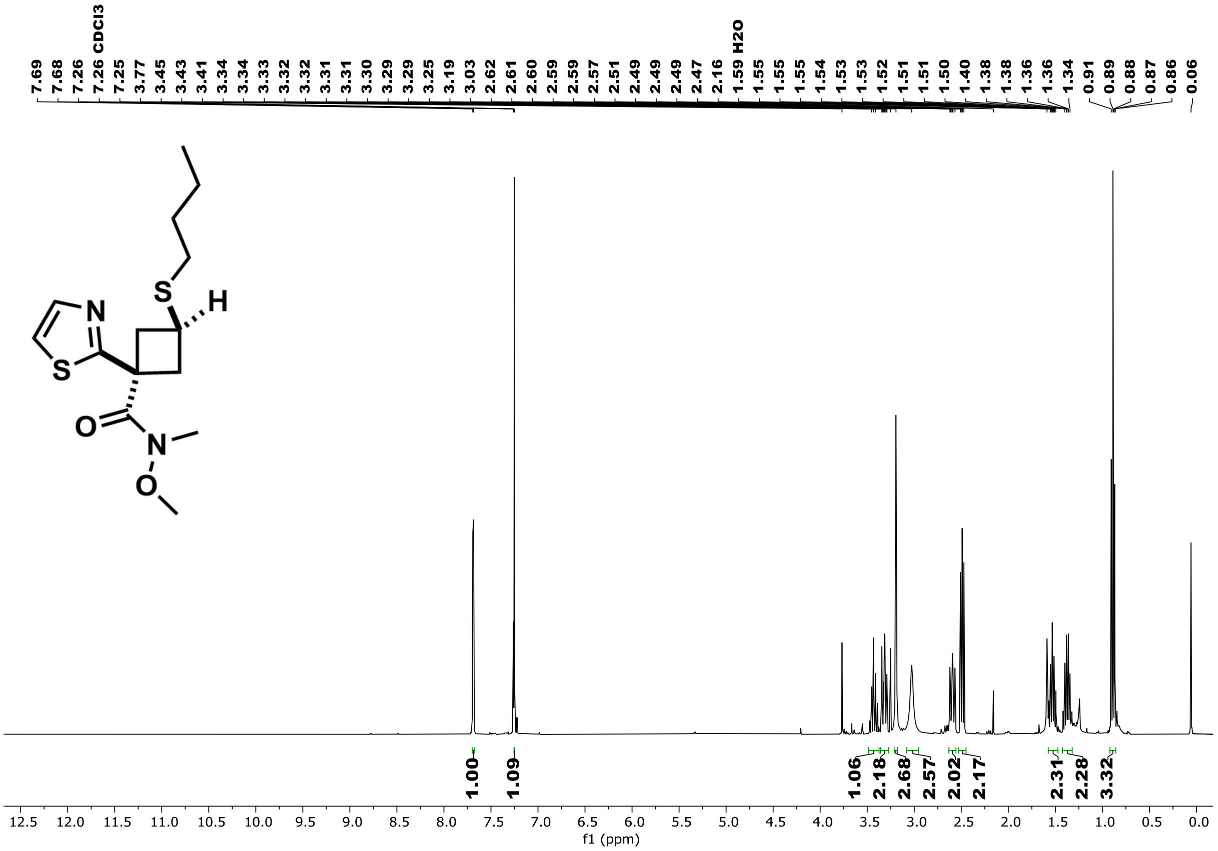
7.69
7.68
7.26
7.26 CDCl3
7.25
3.77
3.45
3.43
3.41
3.34
3.34
3.33
3.32
3.32
3.31
3.31
3.30
3.29
3.29
3.25
3.19
3.03
2.62
2.61
2.60
2.59
2.59
2.57
2.51
2.49
2.49
2.49
2.47
2.16
1.59 H2O
1.55
1.55
1.55
1.54
1.53
1.53
1.52
1.51
1.51
1.50
1.40
1.38
1.38
1.36
1.36
1.34
0.91
0.89
0.88
0.87
0.86
0.06
1.00
1.09
1.06
2.18
2.68
2.57
2.02
2.17
2.31
2.28
3.32
f1 (ppm)

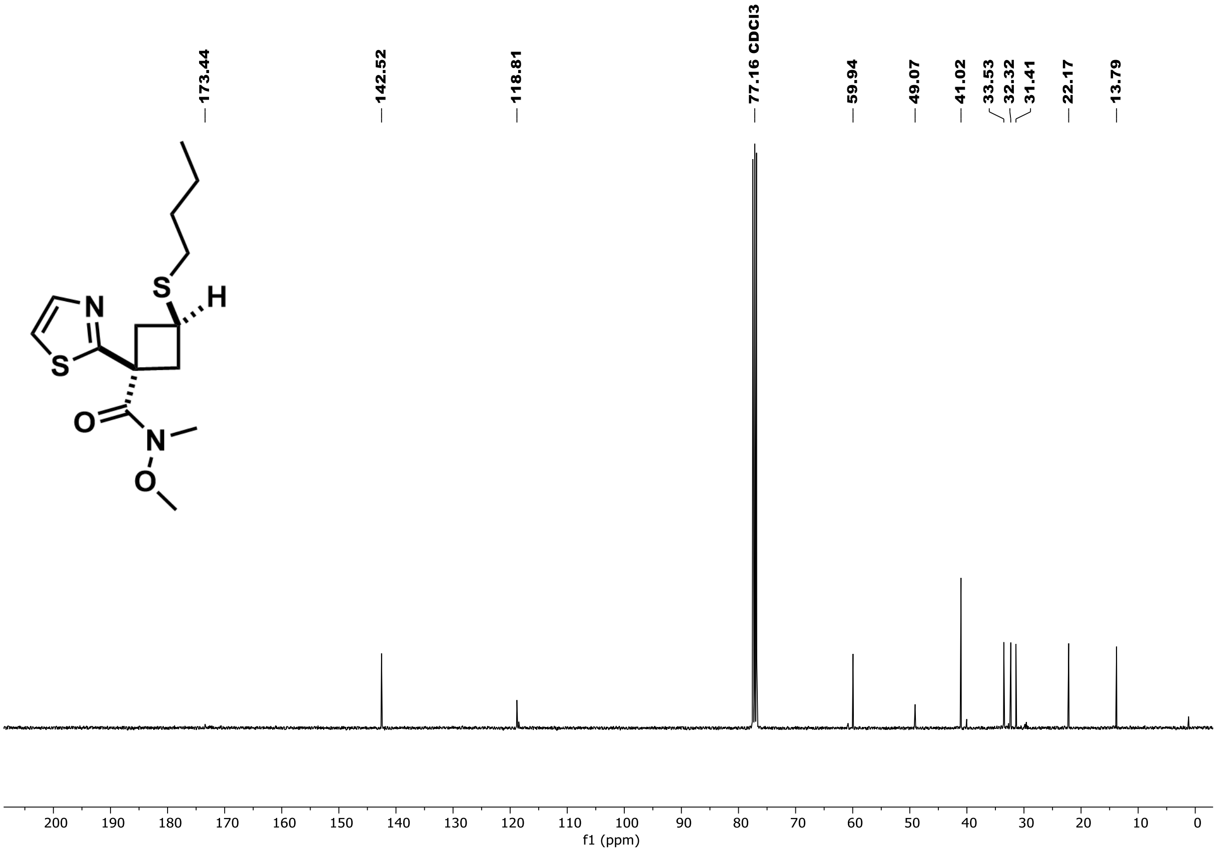
173.44
142.52
118.81
77.16 CDCl3
59.94
49.07
41.02
33.53
32.32
31.41
22.17
13.79
f1 (ppm)

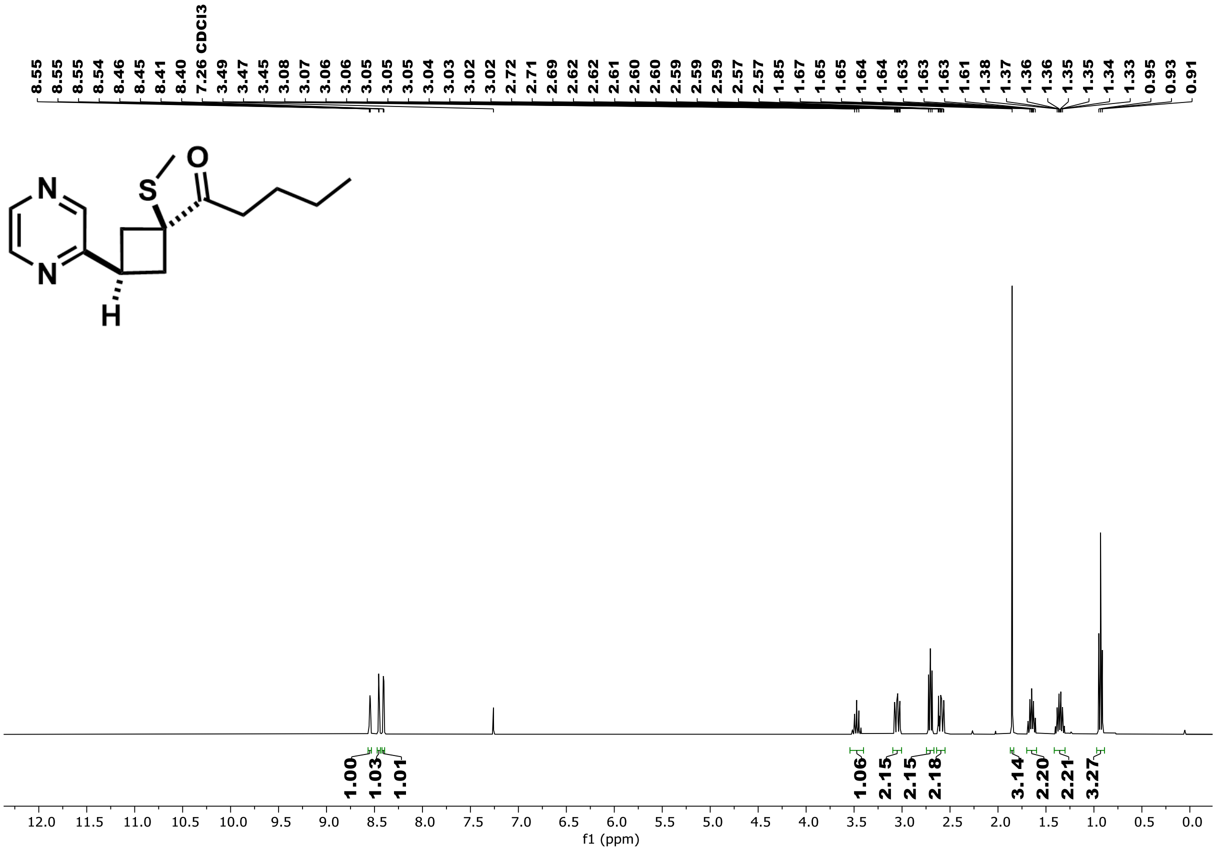
8.55 8.55 8.55 8.54 8.46 8.45 8.41 8.40 7.26 CDCl3 3.49 3.47 3.45 3.08 3.07 3.06 3.06 3.05 3.05 3.05 3.04 3.03 3.02 3.02 2.72 2.71 2.69 2.62 2.62 2.61 2.60 2.60 2.59 2.59 2.59 2.57 2.57 1.85 1.67 1.65 1.65 1.64 1.64 1.63 1.63 1.63 1.63 1.61 1.38 1.37 1.36 1.36 1.35 1.35 1.34 1.33 0.95 0.93 0.91
N
N
S
O
H
1.00 1.03 1.01
1.06 2.15 2.15 2.18
3.14 2.20 2.21 3.27
12.0 11.5 11.0 10.5 10.0 9.5 9.0 8.5 8.0 7.5 7.0 6.5 6.0 5.5 5.0 4.5 4.0 3.5 3.0 2.5 2.0 1.5 1.0 0.5 0.0
f1 (ppm)

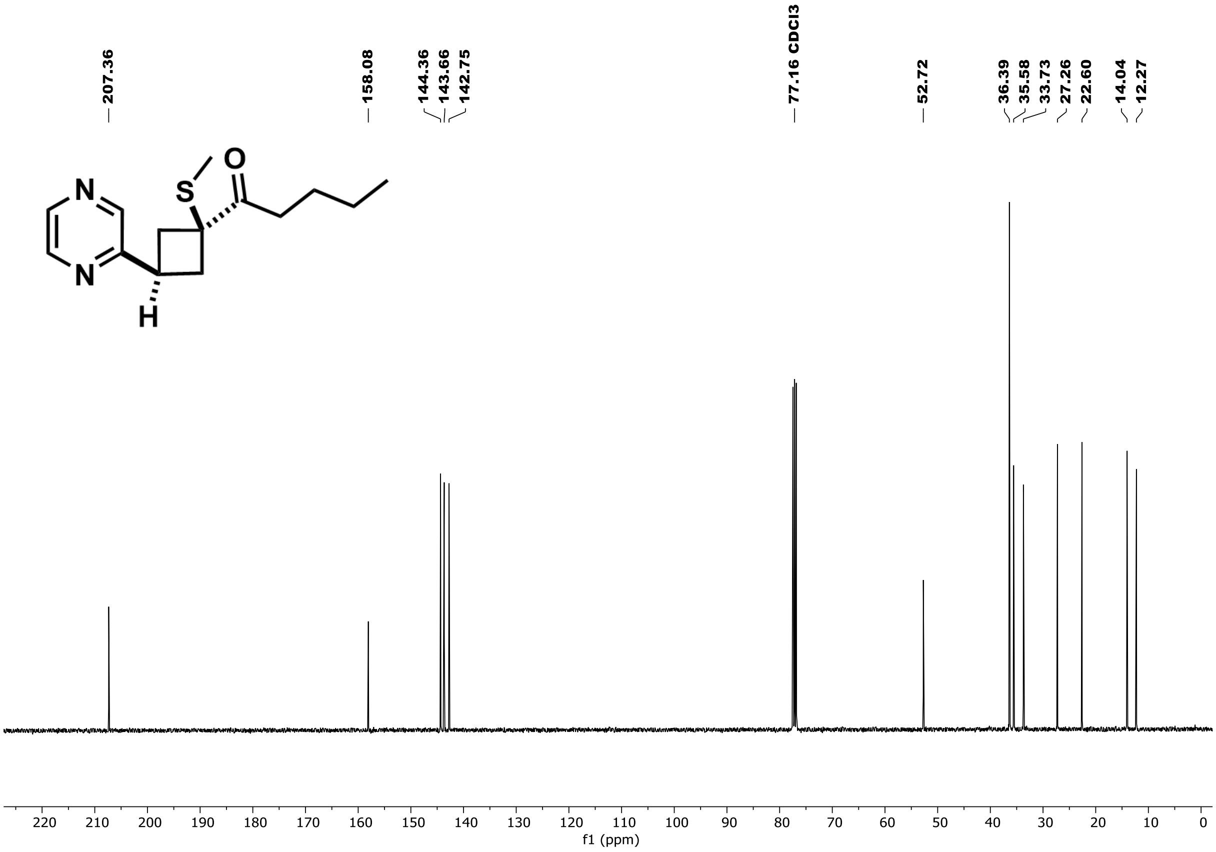
207.36
158.08
144.36 143.66 142.75
77.16 CDCl3
52.72
36.39 35.58 33.73 27.26 22.60 14.04 12.27
N
N
S
O
H
220 210 200 190 180 170 160 150 140 130 120 110 100 90 80 70 60 50 40 30 20 10 0
f1 (ppm)

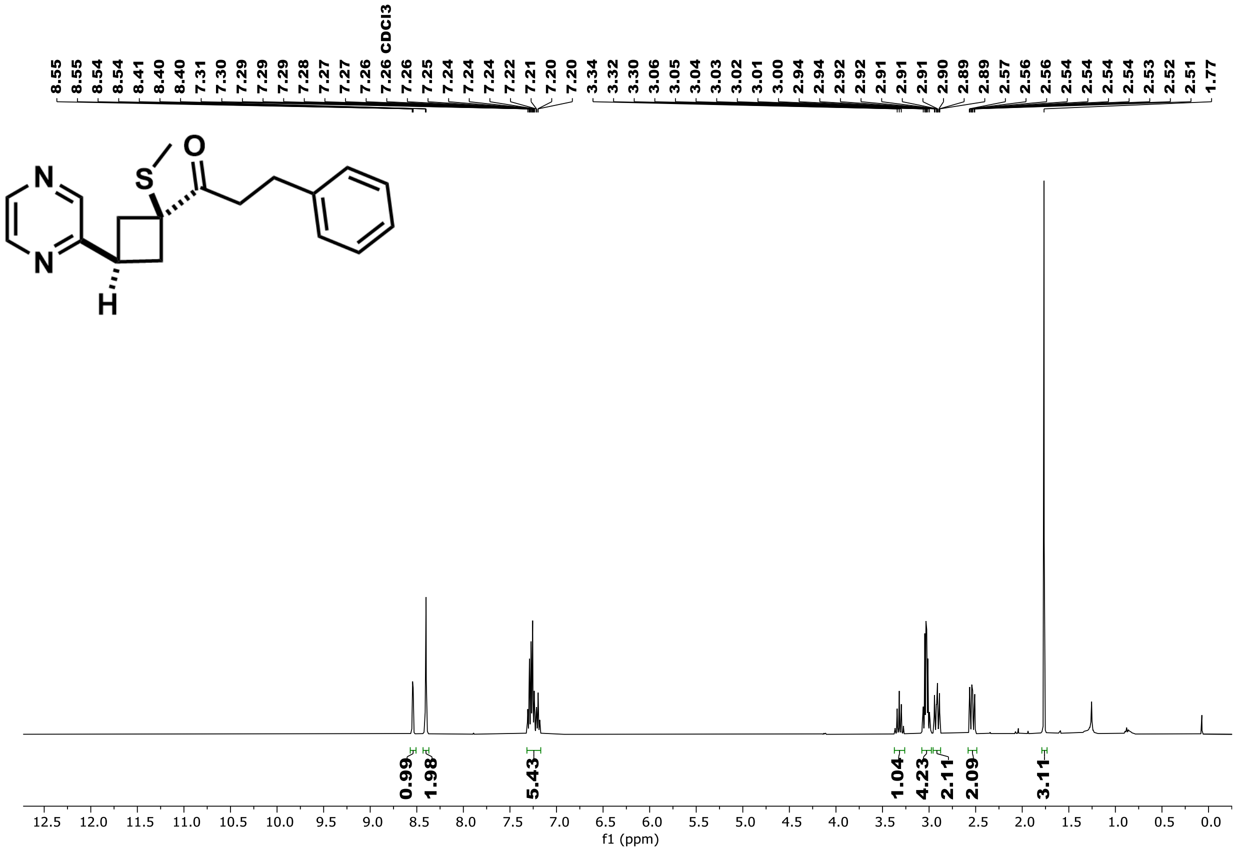

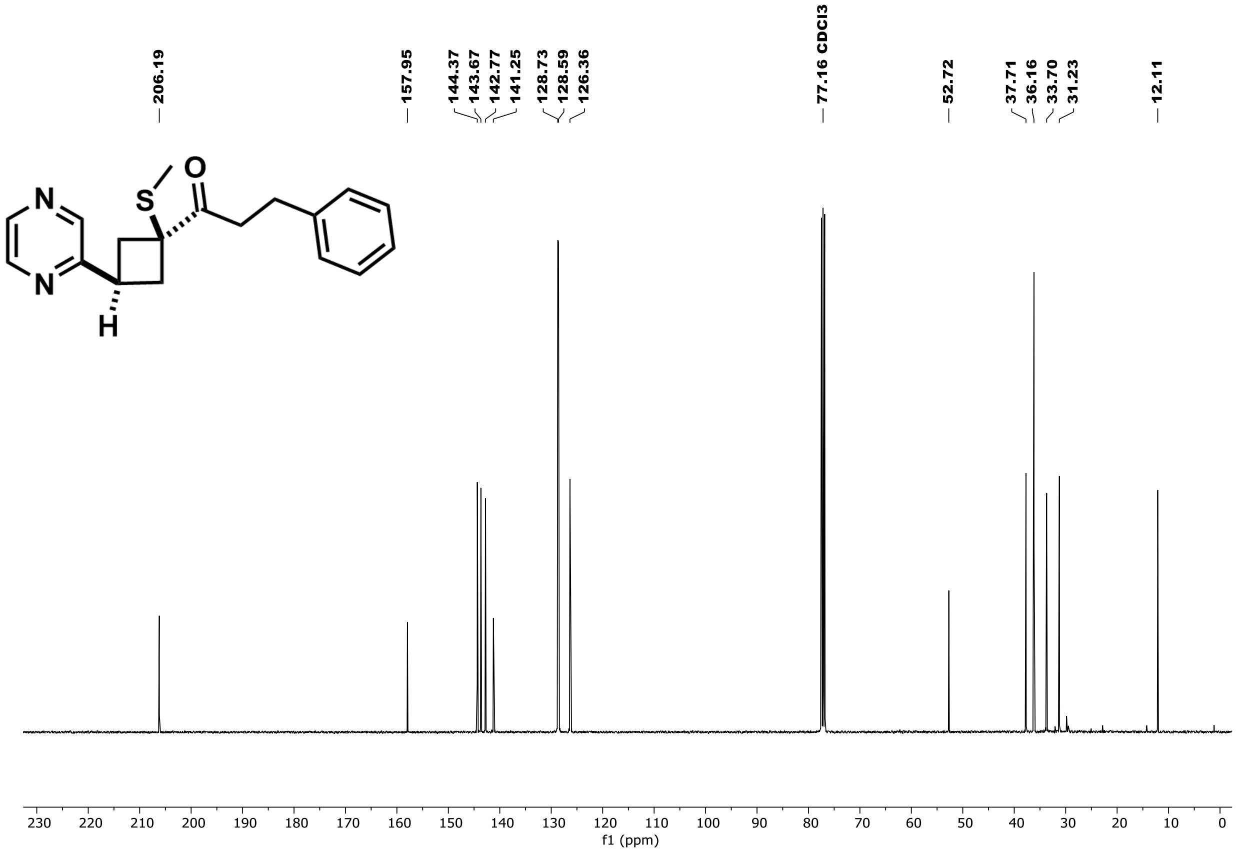

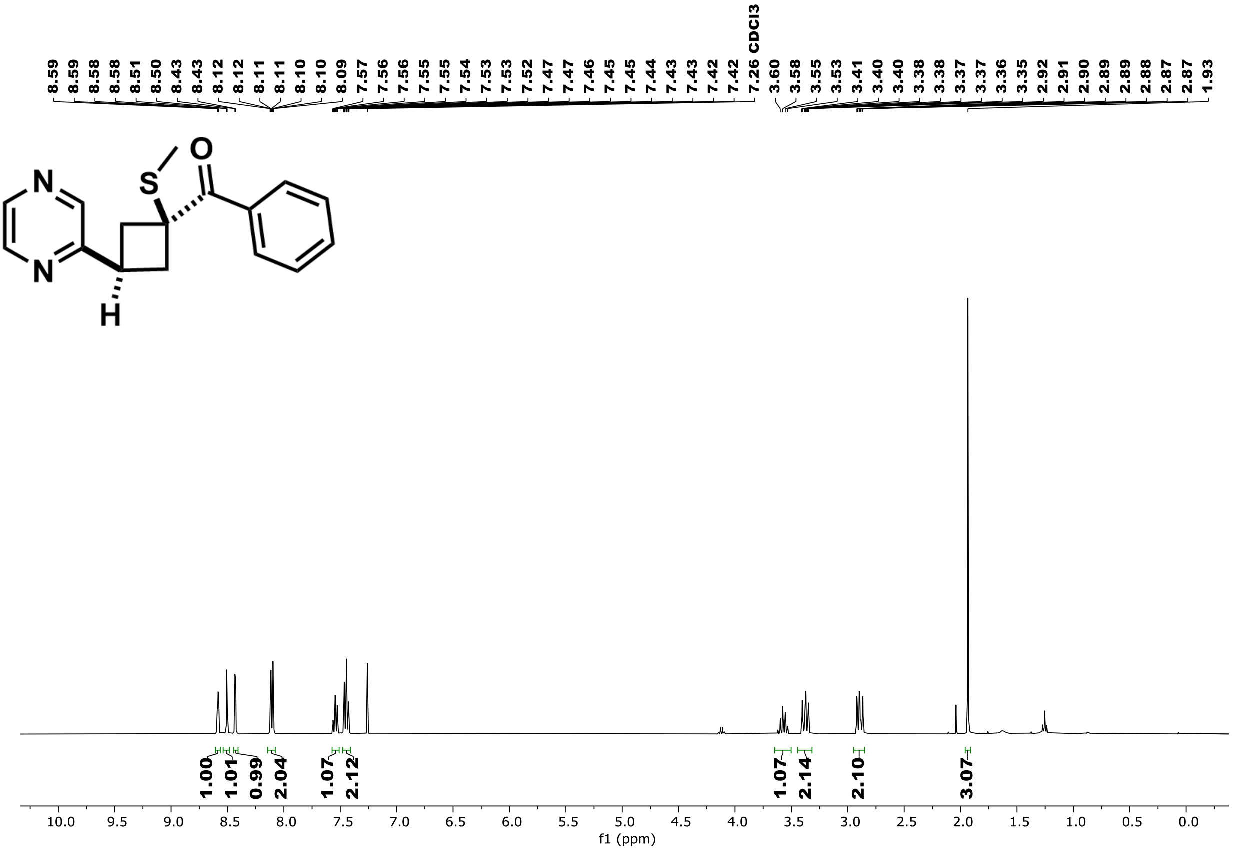
8.59 8.59 8.58 8.58 8.51 8.50 8.43 8.43 8.12 8.12 8.11 8.11 8.10 8.09 7.57 7.56 7.56 7.55 7.55 7.54 7.53 7.53 7.52 7.47 7.47 7.46 7.45 7.45 7.44 7.43 7.43 7.42 7.42 7.26 CDCl3 3.60 3.58 3.55 3.53 3.41 3.40 3.40 3.38 3.38 3.37 3.37 3.36 3.35 2.92 2.91 2.90 2.89 2.89 2.88 2.87 2.87 1.93
N
N
S
O
H
1.00
1.01
0.99
2.04
1.07
2.12
1.07
2.14
2.10
3.07
10.0 9.5 9.0 8.5 8.0 7.5 7.0 6.5 6.0 5.5 5.0 4.5 4.0 3.5 3.0 2.5 2.0 1.5 1.0 0.5 0.0
f1 (ppm)

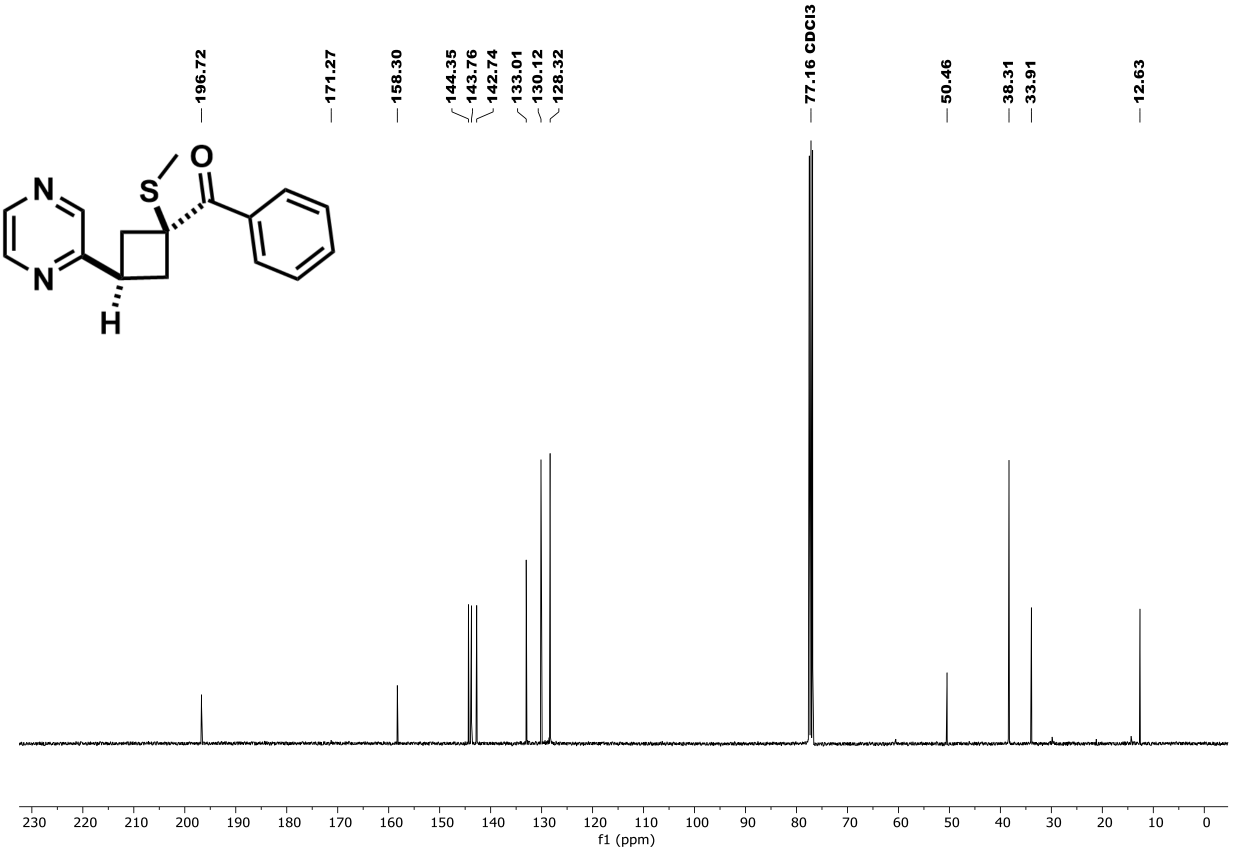
196.72
171.27
158.30
144.35 143.76 142.74
133.01 130.12 128.32
77.16 CDCl3
50.46
38.31 33.91
12.63
N
N
S
O
H
230 220 210 200 190 180 170 160 150 140 130 120 110 100 90 80 70 60 50 40 30 20 10 0
f1 (ppm)

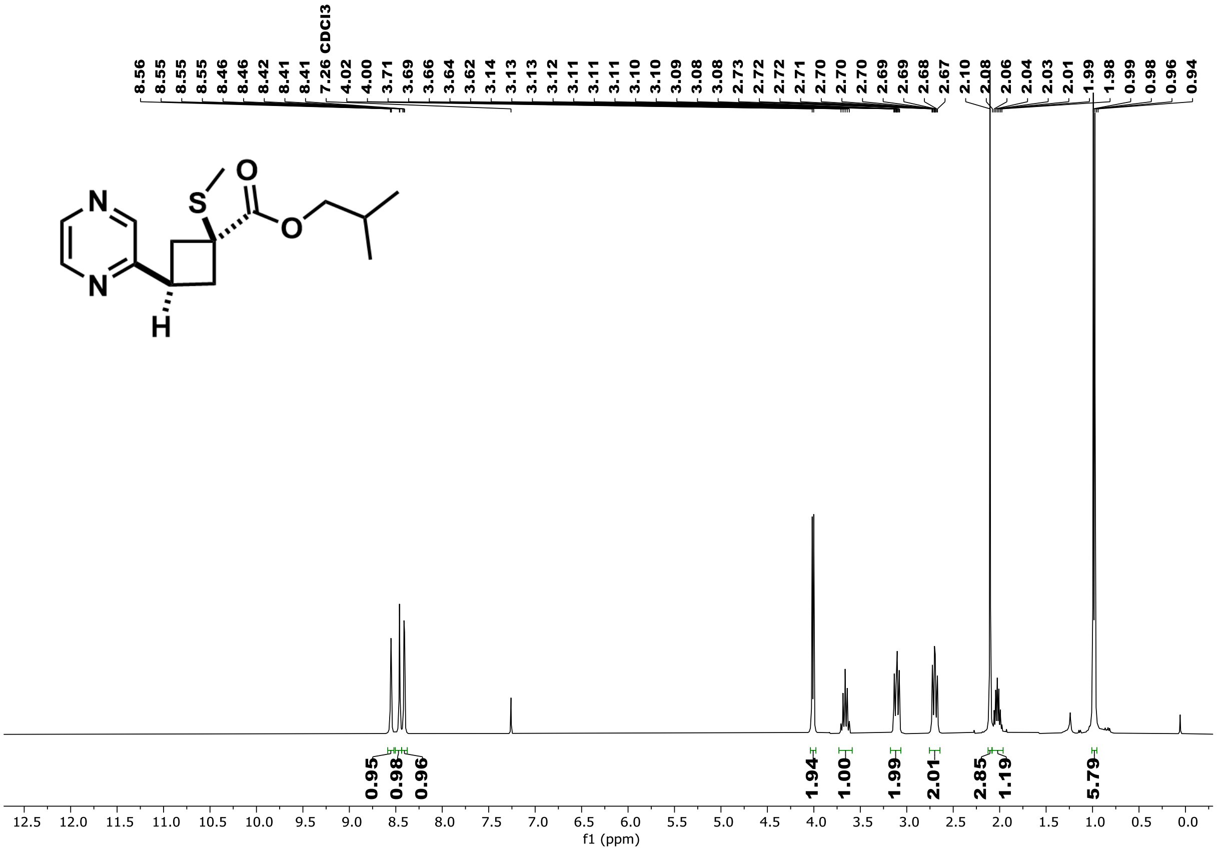
8.56 8.55 8.55 8.55 8.46 8.42 8.41 7.26 CDCl3 4.02 4.00 3.71 3.69 3.66 3.64 3.62 3.14 3.13 3.13 3.12 3.11 3.11 3.11 3.10 3.10 3.09 3.08 3.08 2.73 2.72 2.72 2.71 2.70 2.70 2.70 2.69 2.69 2.68 2.67 2.10 2.08 2.06 2.04 2.03 2.01 1.99 1.98 0.99 0.98 0.96 0.94
N
N
S
O
O
H
0.95 0.98 0.96 1.94 1.00 1.99 2.01 2.85 1.19 5.79
12.5 12.0 11.5 11.0 10.5 10.0 9.5 9.0 8.5 8.0 7.5 7.0 6.5 6.0 5.5 5.0 4.5 4.0 3.5 3.0 2.5 2.0 1.5 1.0 0.5 0.0
f1 (ppm)

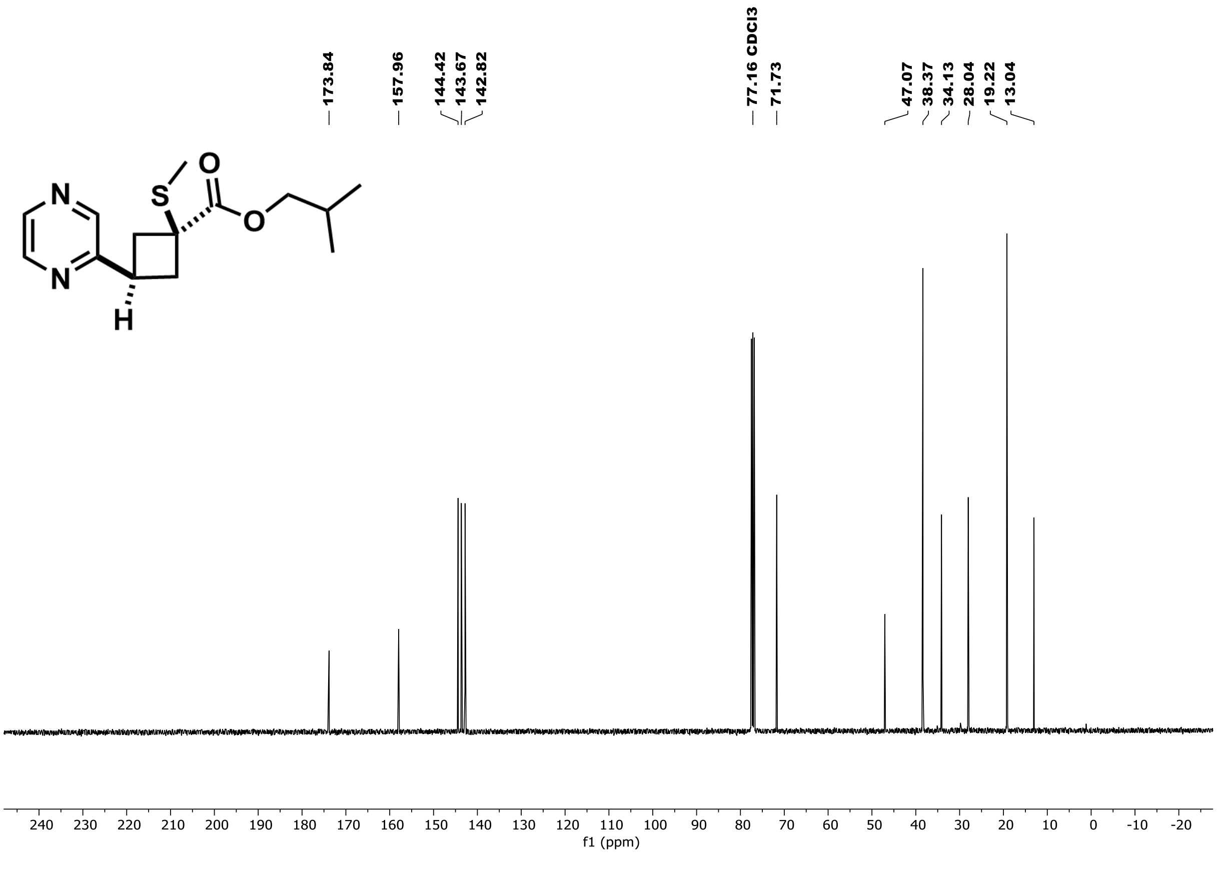
173.84 157.96 144.42 143.67 142.82 77.16 CDCl3 71.73 47.07 38.37 34.13 28.04 19.22 13.04
N
N
S
O
O
H
240 230 220 210 200 190 180 170 160 150 140 130 120 110 100 90 80 70 60 50 40 30 20 10 0 -10 -20
f1 (ppm)

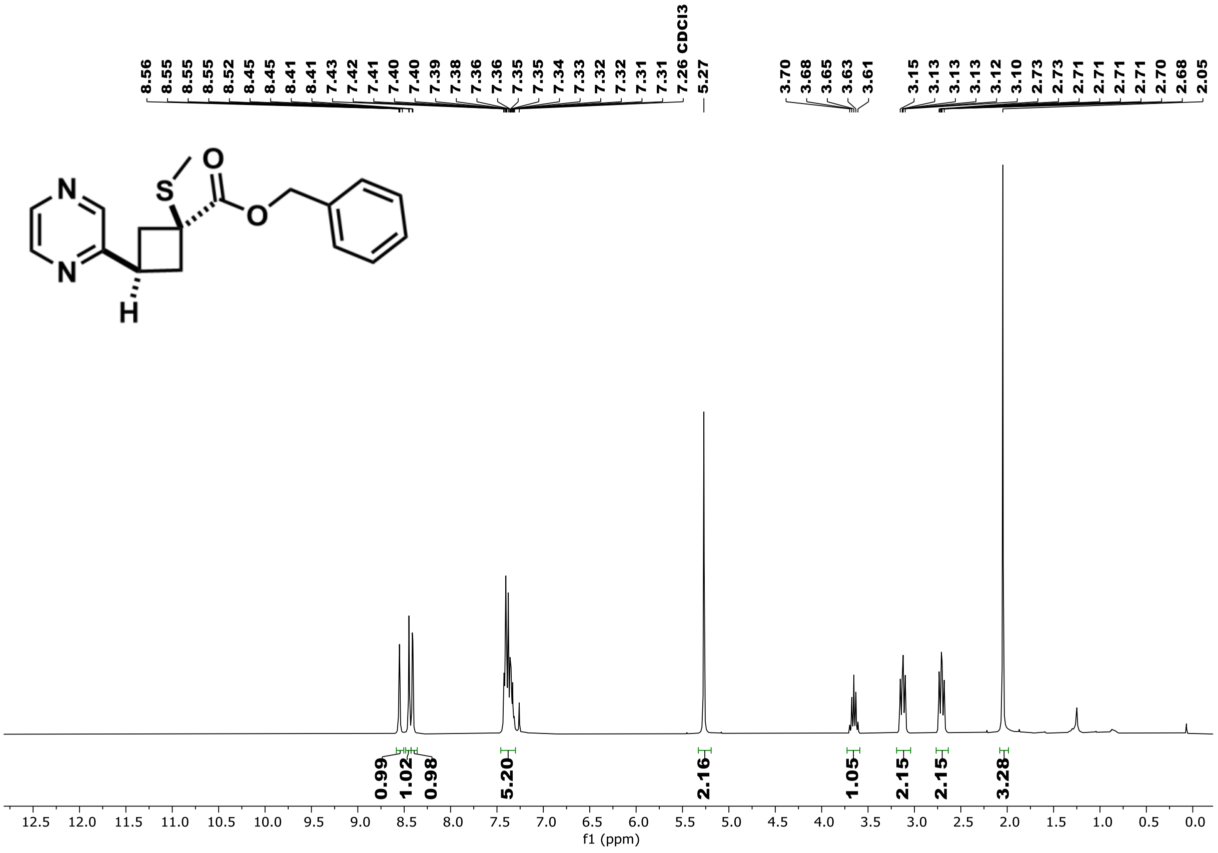
8.56
8.55
8.55
8.55
8.52
8.45
8.45
8.41
8.41
7.43
7.42
7.41
7.40
7.40
7.39
7.38
7.36
7.36
7.35
7.35
7.34
7.33
7.32
7.32
7.31
7.31
7.26 CDCl3
5.27
3.70
3.68
3.65
3.63
3.61
3.15
3.13
3.13
3.13
3.12
3.10
2.73
2.73
2.71
2.71
2.71
2.71
2.70
2.68
2.05
0.99
1.02
0.98
5.20
2.16
1.05
2.15
2.15
3.28
f1 (ppm)

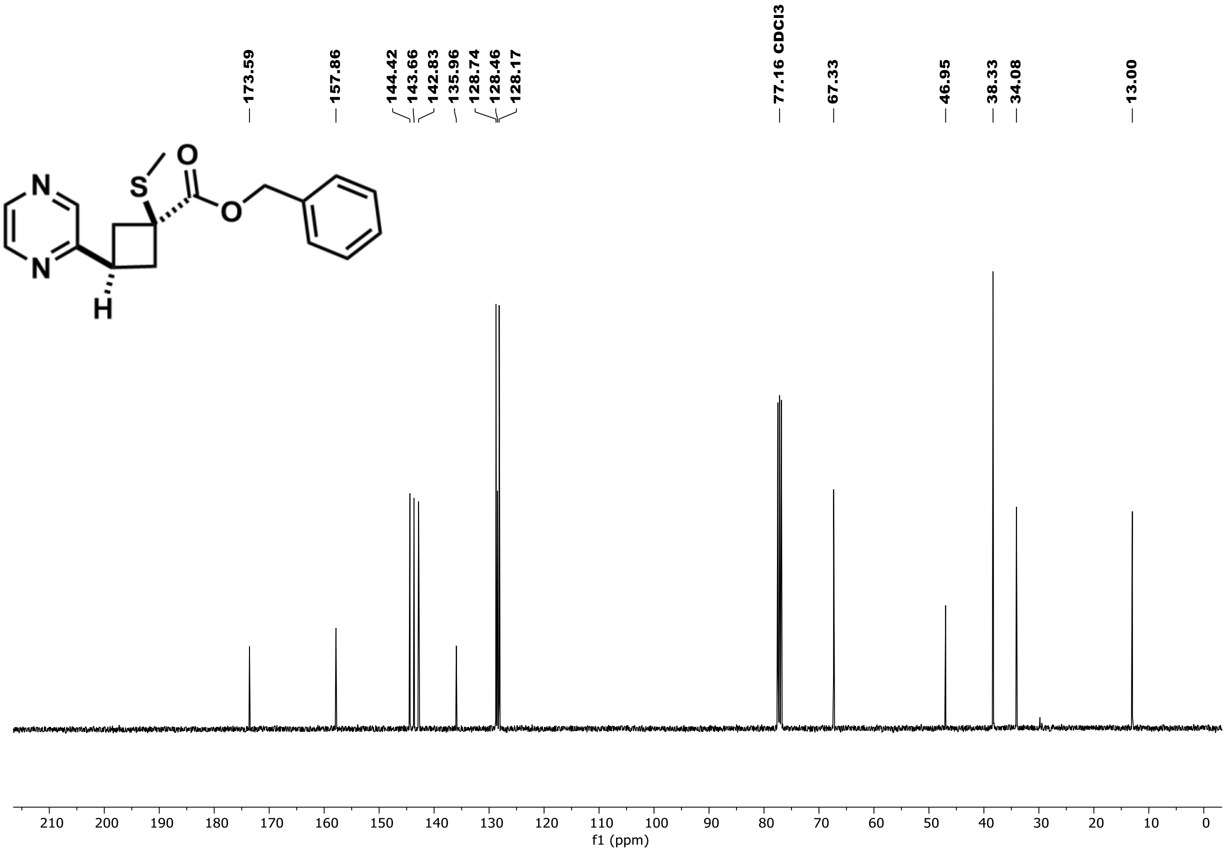
173.59
157.86
144.42
143.66
142.83
135.96
128.74
128.46
128.17
77.16 CDCl3
67.33
46.95
38.33
34.08
13.00
f1 (ppm)

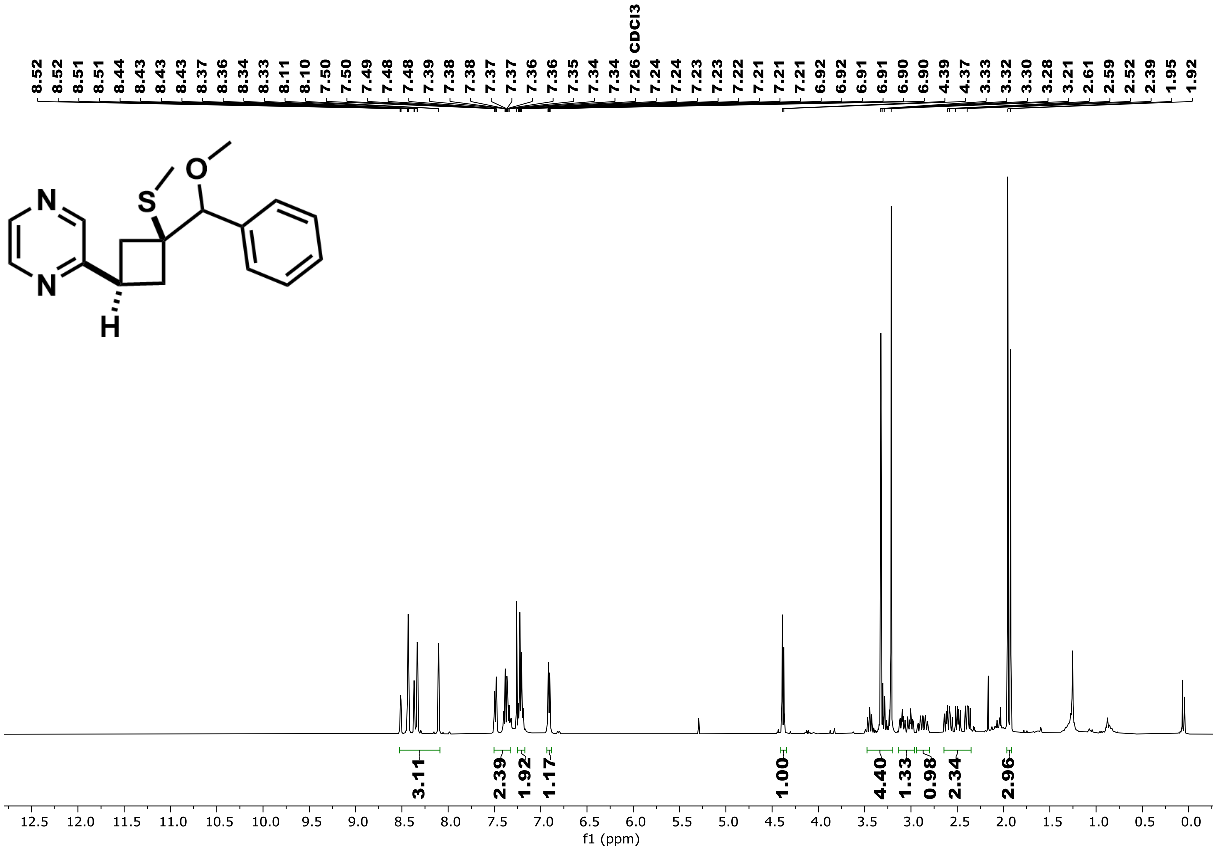
8.52 8.52 8.51 8.51 8.44 8.43 8.43 8.43 8.37 8.36 8.34 8.33 8.11 8.10 7.50 7.50 7.49 7.48 7.48 7.39 7.38 7.38 7.37 7.37 7.36 7.36 7.35 7.34 7.34 7.26 CDCl3 7.24 7.24 7.23 7.23 7.22 7.21 7.21 7.21 6.92 6.92 6.91 6.91 6.90 6.90 4.39 4.37 3.33 3.32 3.30 3.28 3.21 2.61 2.59 2.52 2.39 1.95 1.92
N
N
S
O
H
3.11
2.39
1.92
1.17
1.00
4.40
1.33
0.98
2.34
2.96
12.5 12.0 11.5 11.0 10.5 10.0 9.5 9.0 8.5 8.0 7.5 7.0 6.5 6.0 5.5 5.0 4.5 4.0 3.5 3.0 2.5 2.0 1.5 1.0 0.5 0.0
f1 (ppm)

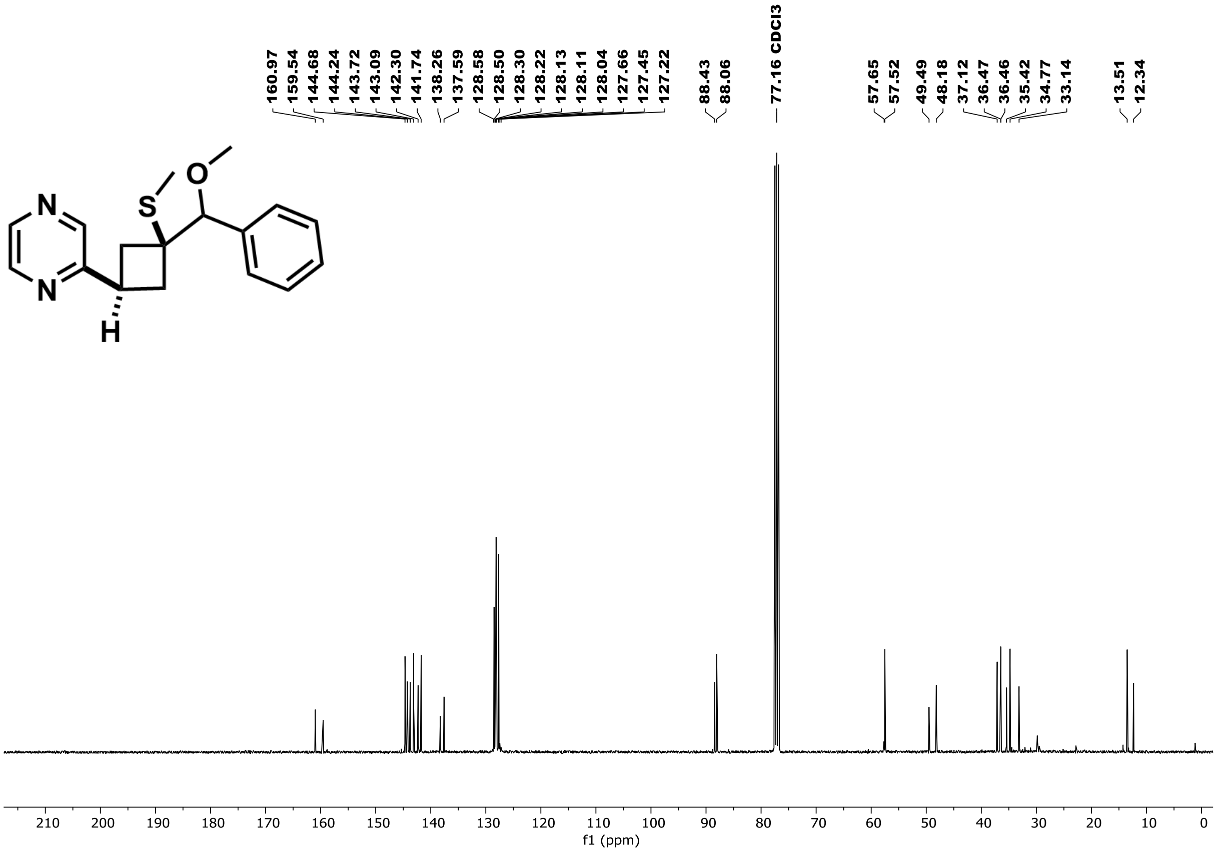
160.97 159.54 144.68 144.24 143.72 143.09 142.30 141.74 138.26 137.59 128.58 128.50 128.30 128.22 128.13 128.11 128.04 127.66 127.45 127.22 88.43 88.06 77.16 CDCl3 57.65 57.52 49.49 48.18 37.12 36.47 36.46 35.42 34.77 33.14 13.51 12.34
N
N
S
O
H
210 200 190 180 170 160 150 140 130 120 110 100 90 80 70 60 50 40 30 20 10 0
f1 (ppm)

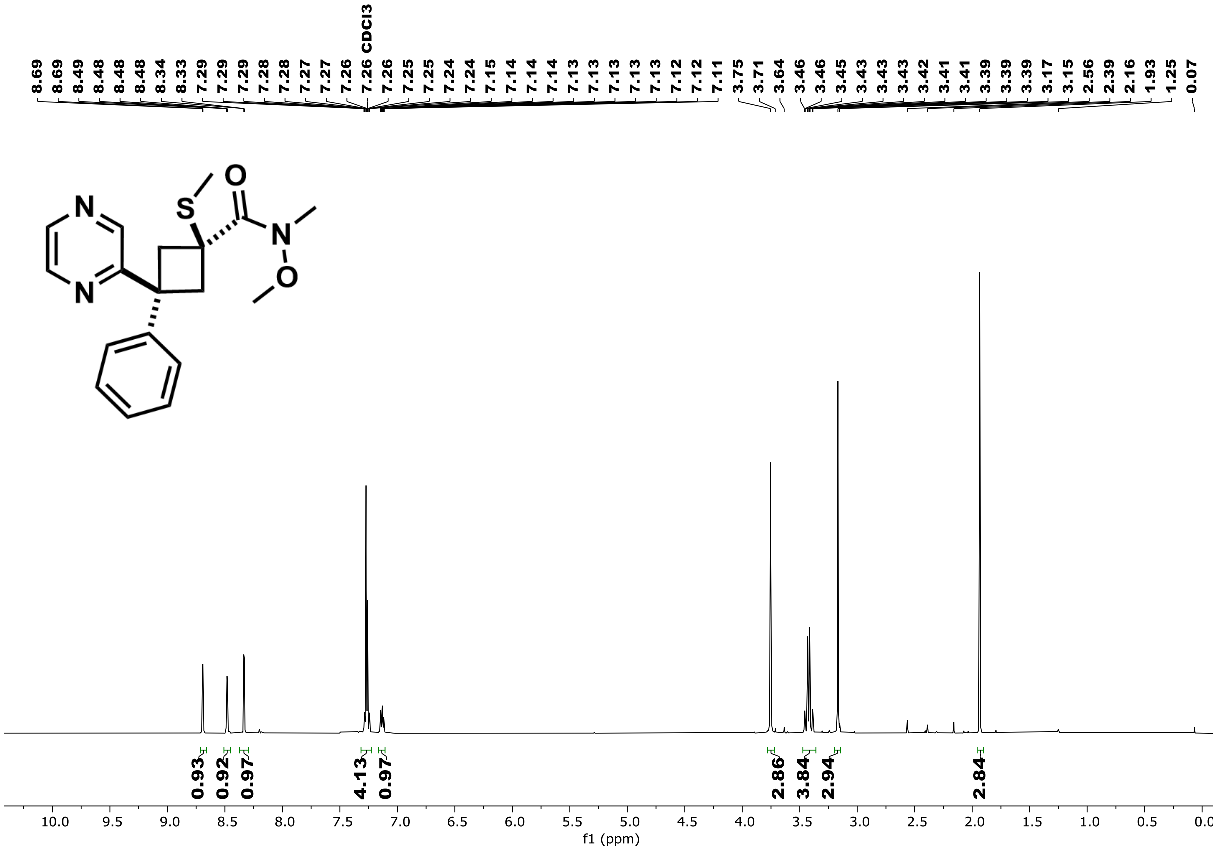
8.69 8.69 8.49 8.48 8.48 8.48 8.34 8.33 7.29 7.29 7.29 7.28 7.28 7.27 7.27 7.26 7.26 CDCl3 7.26 7.25 7.25 7.24 7.24 7.15 7.14 7.14 7.14 7.13 7.13 7.13 7.13 7.13 7.12 7.12 7.11 3.75 3.71 3.64 3.46 3.46 3.45 3.43 3.43 3.43 3.42 3.41 3.41 3.39 3.39 3.39 3.17 3.15 2.56 2.39 2.16 1.93 1.25 0.07
N
N
S
O
N
O
0.93
0.92
0.97
4.13
0.97
2.86
3.84
2.94
2.84
10.0 9.5 9.0 8.5 8.0 7.5 7.0 6.5 6.0 5.5 5.0 4.5 4.0 3.5 3.0 2.5 2.0 1.5 1.0 0.5 0.0
f1 (ppm)

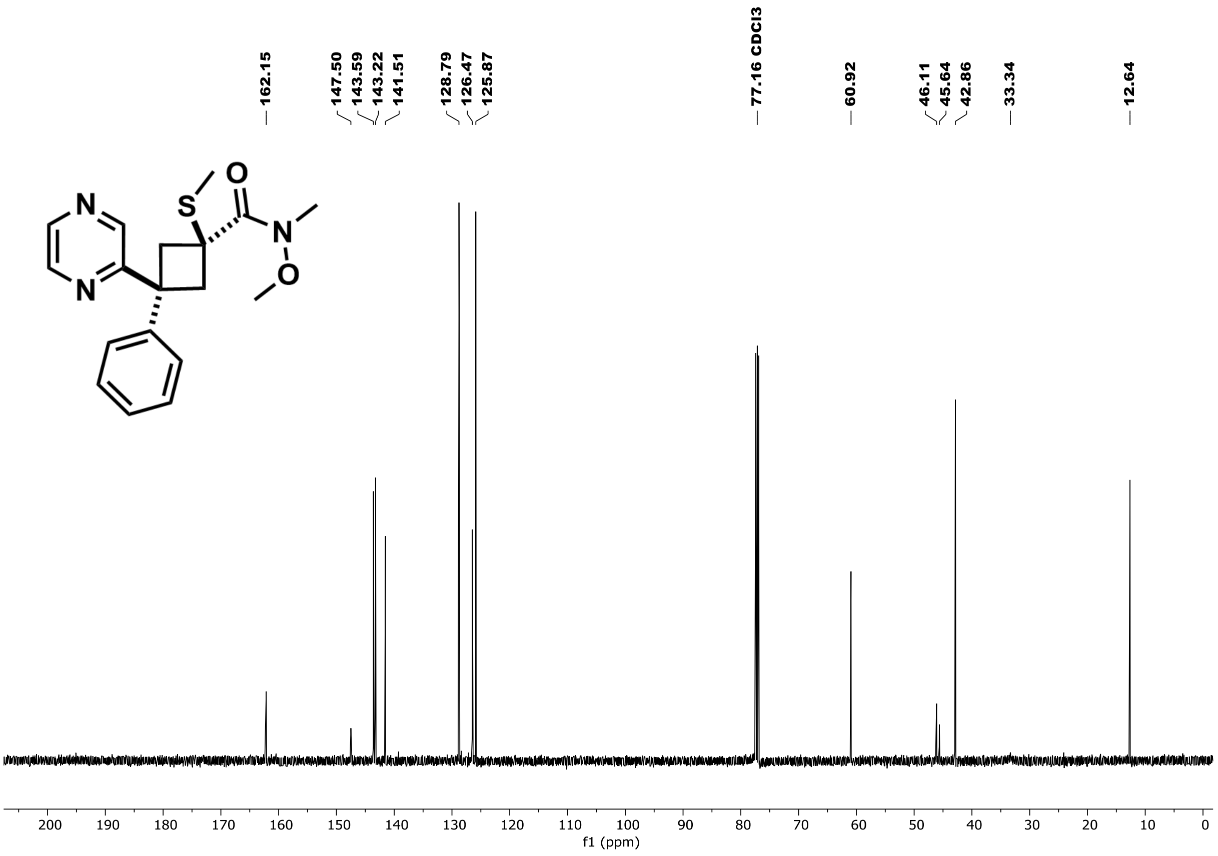
162.15
147.50 143.59 143.22 141.51
128.79 126.47 125.87
77.16 CDCl3
60.92
46.11 45.64 42.86
33.34
12.64
N
N
S
O
N
O
200 190 180 170 160 150 140 130 120 110 100 90 80 70 60 50 40 30 20 10 0
f1 (ppm)

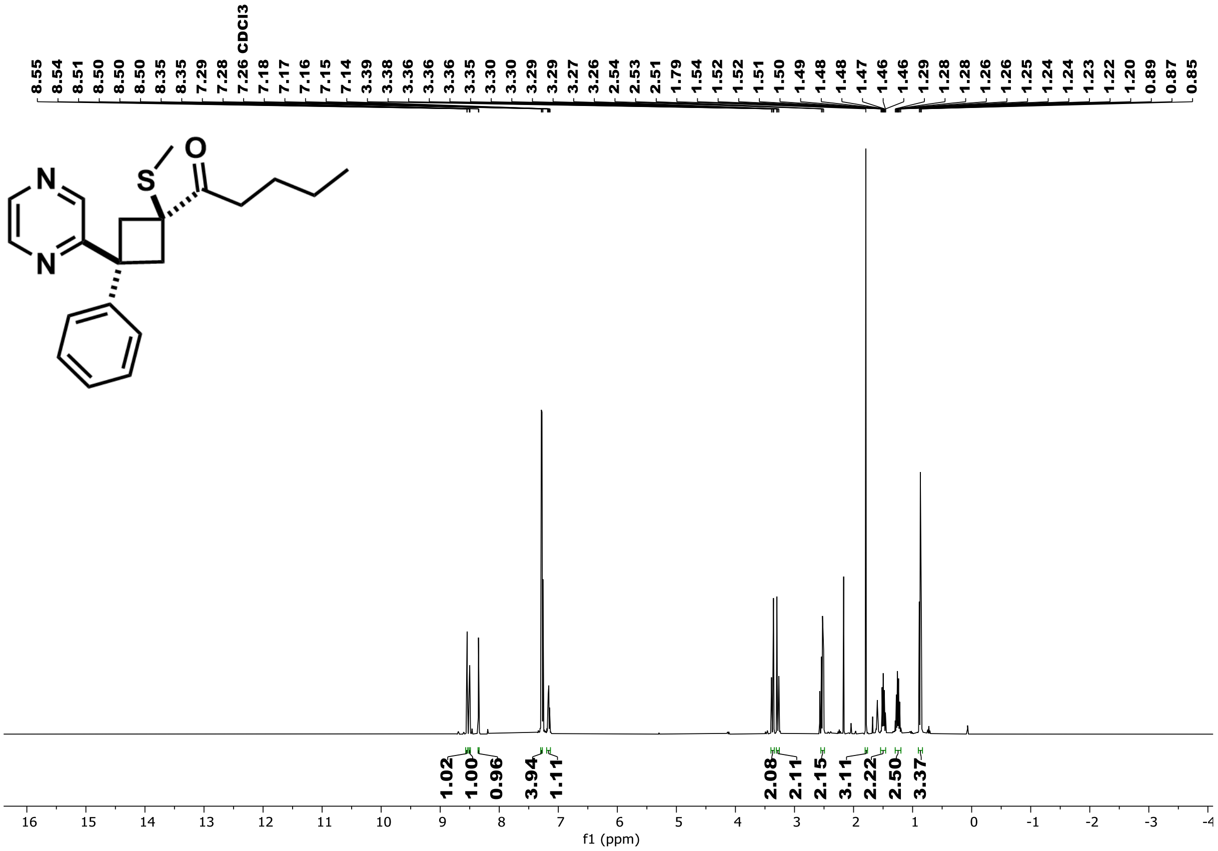
8.55 8.54 8.51 8.50 8.50 8.50 8.35 8.35 7.29 7.28 7.26 CDCl3 7.18 7.17 7.16 7.15 7.14 3.39 3.38 3.36 3.36 3.36 3.35 3.30 3.30 3.29 3.29 3.27 3.26 2.54 2.53 2.51 1.79 1.54 1.52 1.52 1.51 1.50 1.49 1.48 1.48 1.47 1.46 1.46 1.29 1.28 1.28 1.26 1.26 1.25 1.24 1.24 1.23 1.22 1.20 0.89 0.87 0.85
O
S
N
N
1.02 1.00 0.96 3.94 1.11 2.08 2.11 2.15 3.11 2.22 2.50 3.37
f1 (ppm)

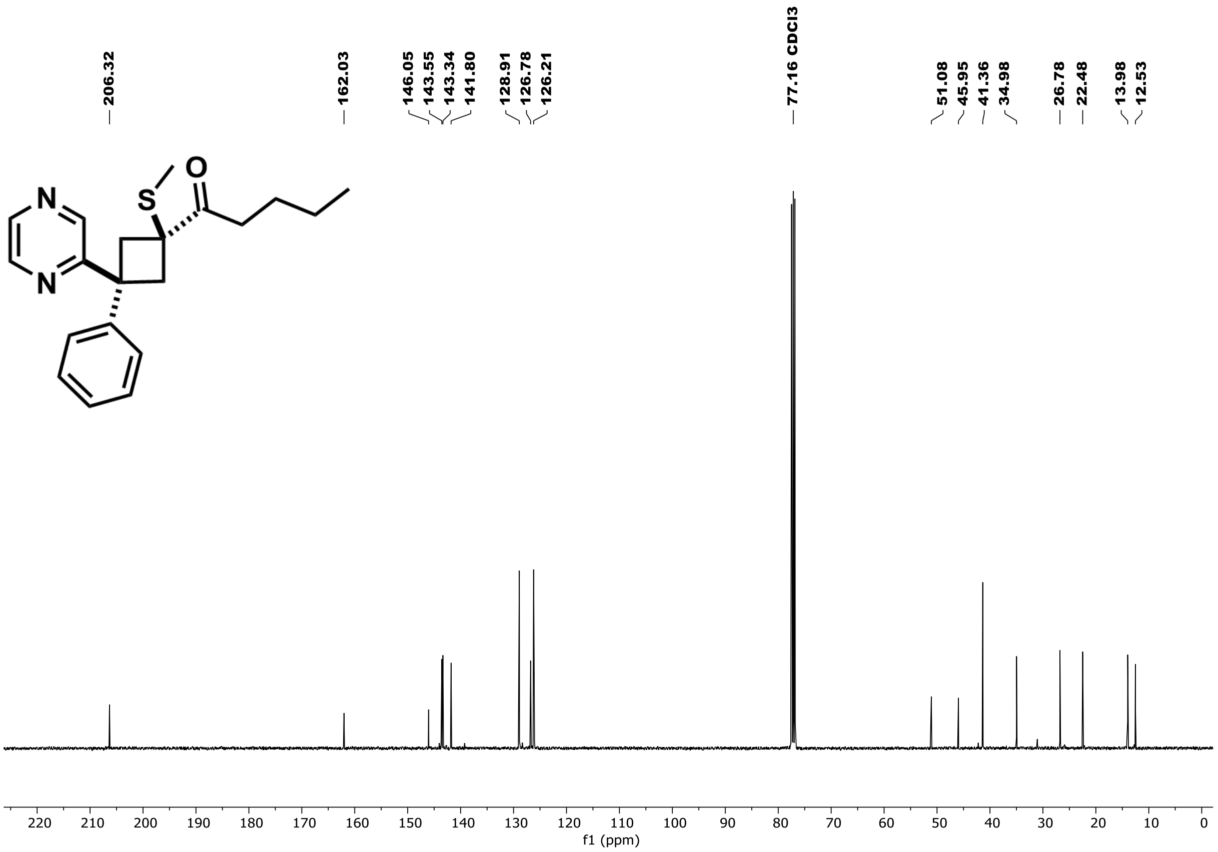
206.32 162.03 146.05 143.55 143.34 141.80 128.91 126.78 126.21 77.16 CDCl3 51.08 45.95 41.36 34.98 26.78 22.48 13.98 12.53
O
S
N
N
f1 (ppm)

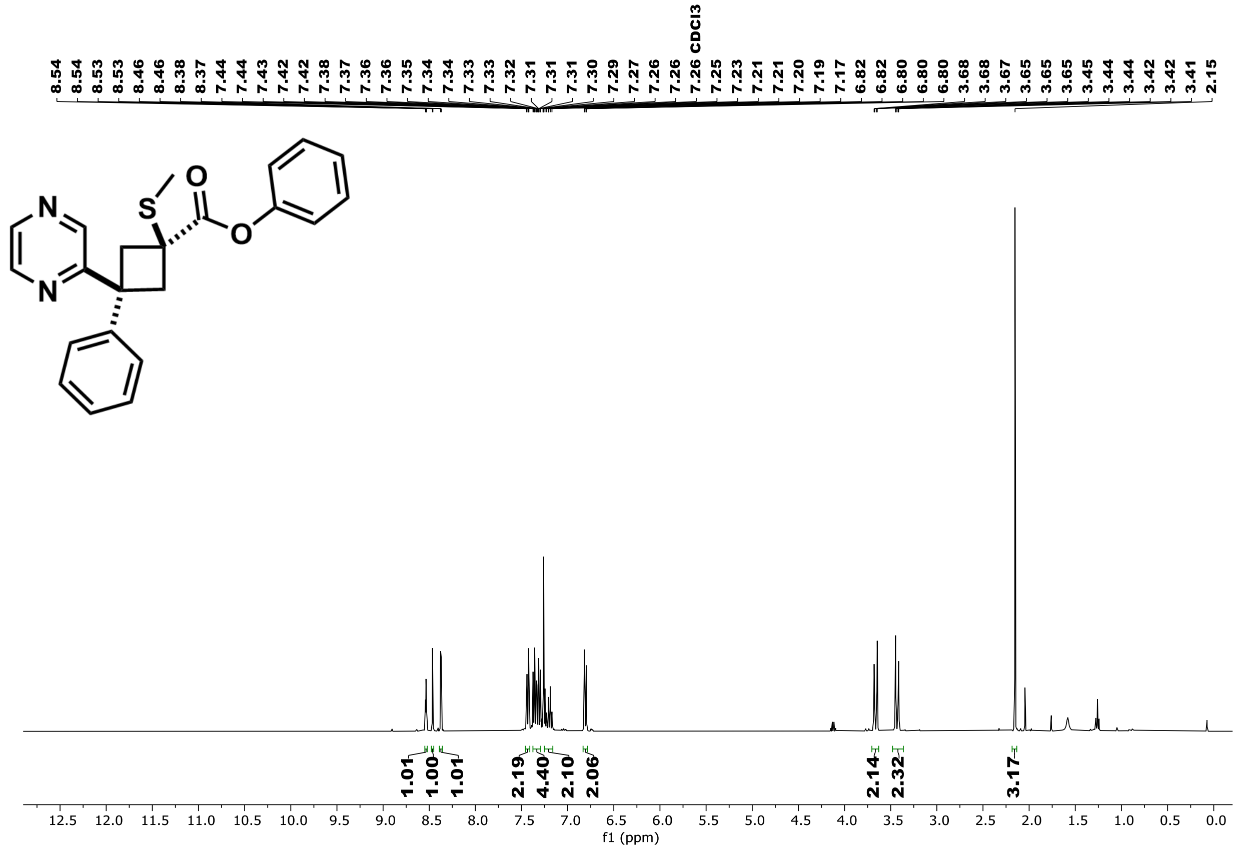
8.54 8.54 8.53 8.53 8.46 8.46 8.38 8.37 7.44 7.44 7.43 7.42 7.42 7.38 7.37 7.36 7.36 7.35 7.34 7.34 7.33 7.33 7.32 7.31 7.31 7.31 7.30 7.29 7.27 7.26 7.26 7.26 CDCl3 7.25 7.23 7.21 7.21 7.20 7.19 7.17 6.82 6.82 6.80 6.80 6.80 3.68 3.68 3.67 3.65 3.65 3.65 3.45 3.44 3.44 3.42 3.42 3.41 2.15
N
N
S
O
O
1.01
1.00
1.01
2.19
4.40
2.10
2.06
2.14
2.32
3.17
12.5 12.0 11.5 11.0 10.5 10.0 9.5 9.0 8.5 8.0 7.5 7.0 6.5 6.0 5.5 5.0 4.5 4.0 3.5 3.0 2.5 2.0 1.5 1.0 0.5 0.0
f1 (ppm)

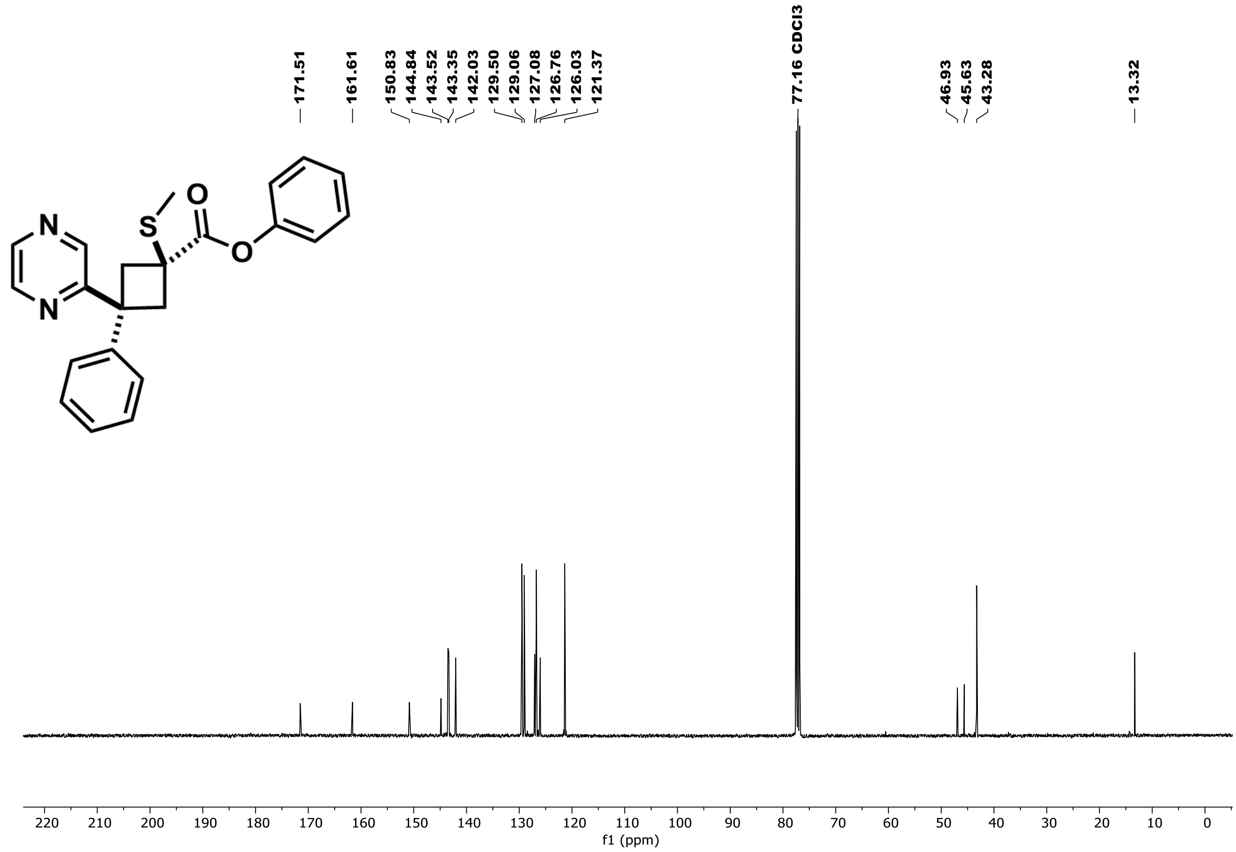
171.51
161.61
150.83 144.84 143.52 143.35 142.03 129.50 129.06 127.08 126.76 126.03 121.37
77.16 CDCl3
46.93 45.63 43.28
13.32
N
N
S
O
O
220 210 200 190 180 170 160 150 140 130 120 110 100 90 80 70 60 50 40 30 20 10 0
f1 (ppm)

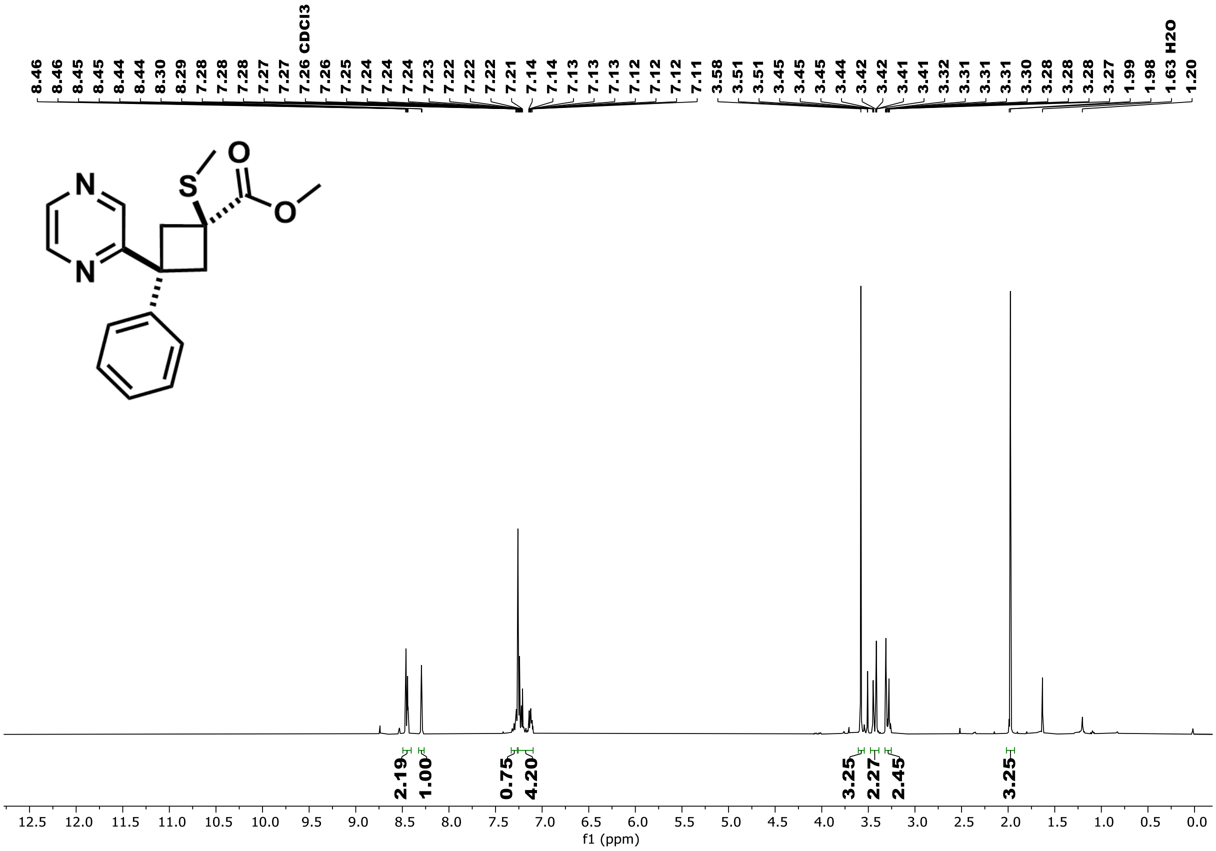

8.46 8.46 8.45 8.45 8.44 8.44 8.30 8.29 7.28 7.28 7.28 7.27 7.27 7.26 CDCl3 7.26 7.25 7.24 7.24 7.24 7.23 7.22 7.22 7.22 7.21 7.14 7.14 7.13 7.13 7.13 7.12 7.12 7.12 7.11 3.58 3.51 3.51 3.45 3.45 3.45 3.44 3.42 3.42 3.41 3.41 3.32 3.31 3.31 3.31 3.30 3.28 3.28 3.28 3.27 1.99 1.98 1.63 H2O 1.20
N
N
S
O
O
2.19
1.00
0.75
4.20
3.25
2.27
2.45
3.25
12.5 12.0 11.5 11.0 10.5 10.0 9.5 9.0 8.5 8.0 7.5 7.0 6.5 6.0 5.5 5.0 4.5 4.0 3.5 3.0 2.5 2.0 1.5 1.0 0.5 0.0
f1 (ppm)

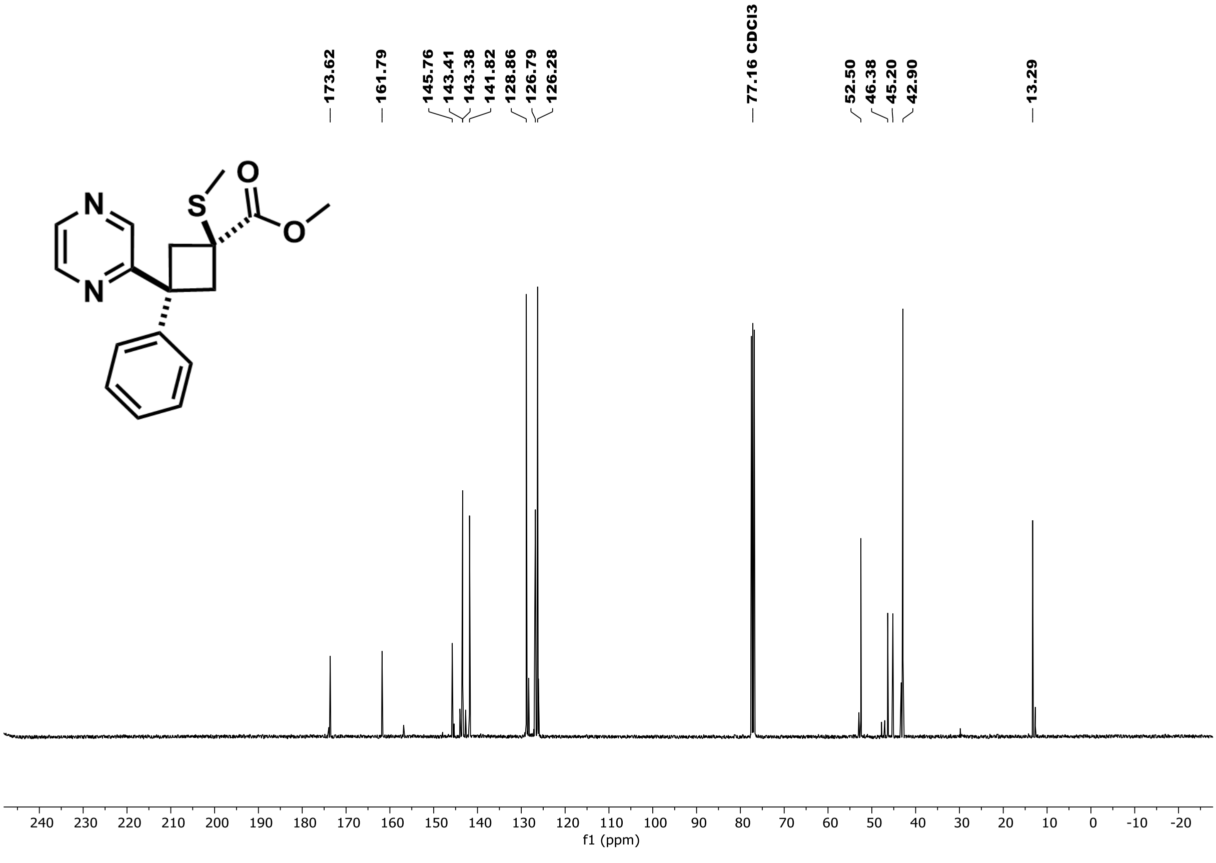

173.62
161.79
145.76 143.41 143.38 141.82 128.86 126.79 126.28
77.16 CDCl3
52.50 46.38 45.20 42.90
13.29
N
N
S
O
O
240 230 220 210 200 190 180 170 160 150 140 130 120 110 100 90 80 70 60 50 40 30 20 10 0 -10 -20
f1 (ppm)

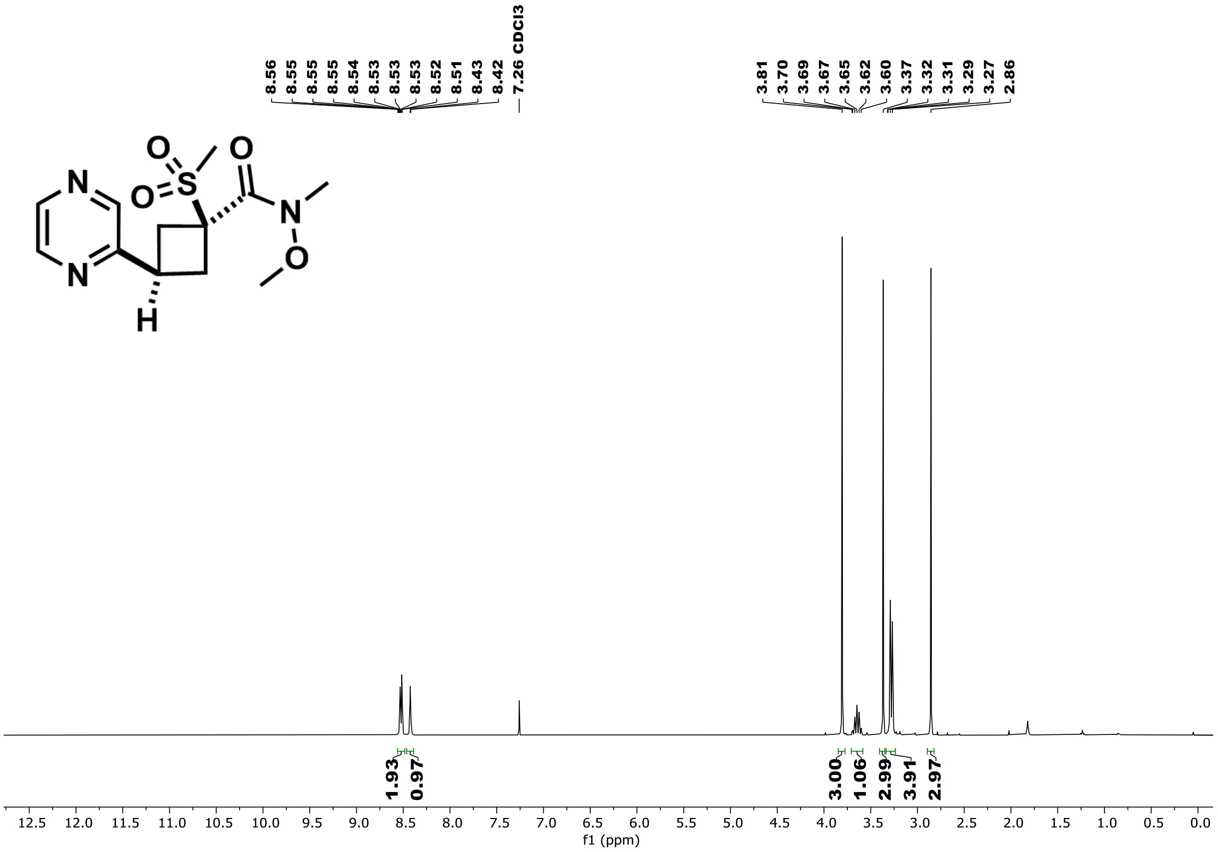
8.56
8.55
8.55
8.55
8.54
8.53
8.53
8.53
8.52
8.51
8.43
8.42
7.26 CDCl3
3.81
3.70
3.69
3.67
3.65
3.62
3.60
3.37
3.32
3.31
3.29
3.27
2.86
1.93
0.97
3.00
1.06
2.99
3.91
2.97
f1 (ppm)

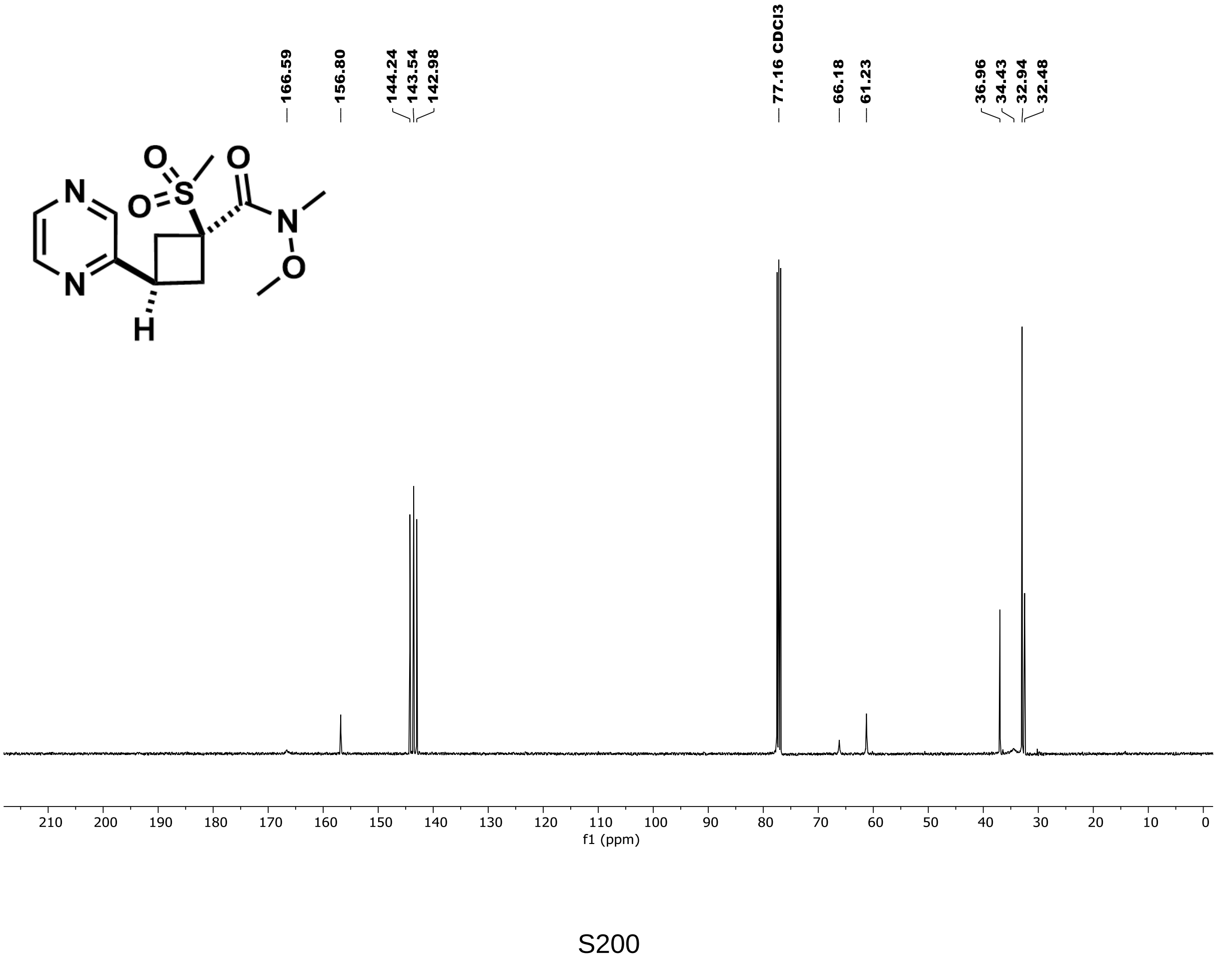
166.59
156.80
144.24
143.54
142.98
77.16 CDCl3
66.18
61.23
36.96
34.43
32.94
32.48
f1 (ppm)

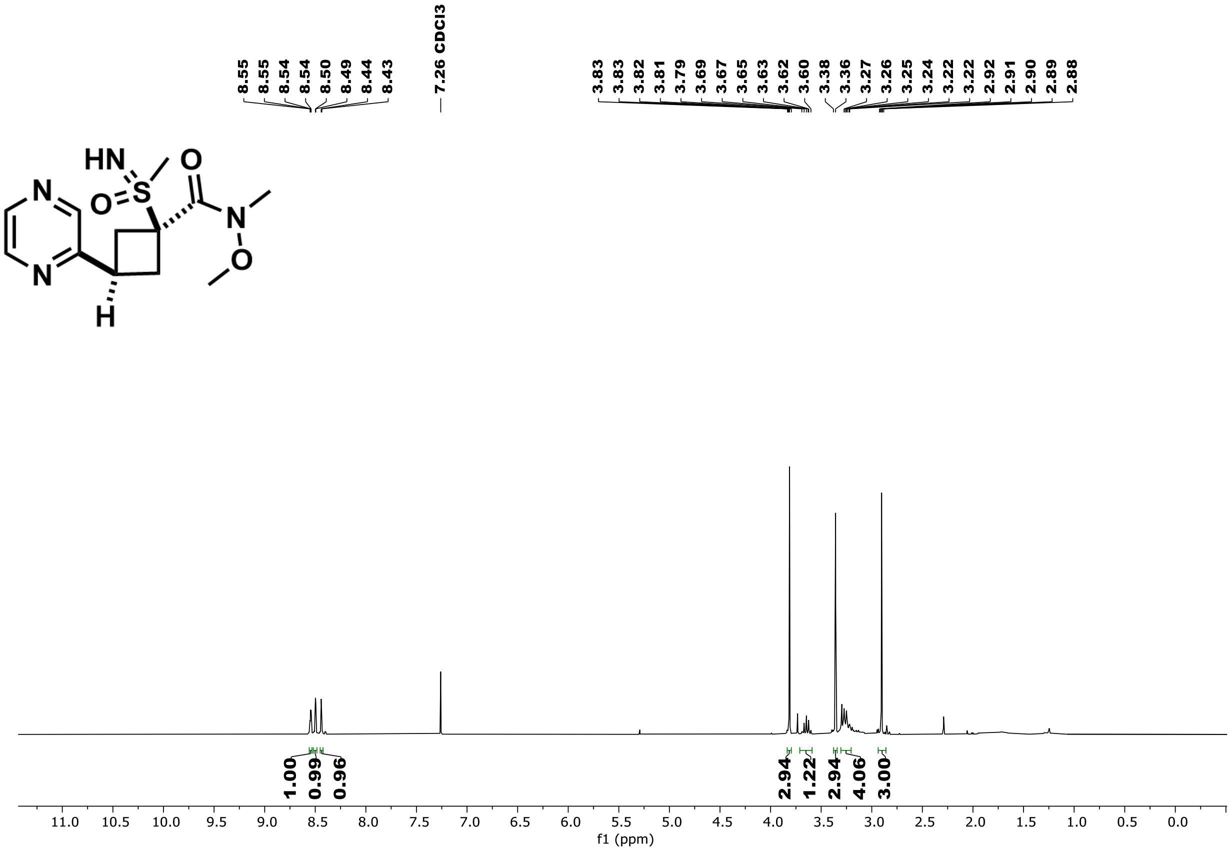
8.55 8.55 8.54 8.54 8.50 8.49 8.44 8.43
7.26 CDCl3
3.83 3.83 3.82 3.81 3.79 3.69 3.67 3.65 3.63 3.62 3.60 3.38 3.36 3.27 3.26 3.25 3.24 3.22 3.22 2.92 2.91 2.90 2.89 2.88
HN
O
S
N
O
N
N
H
1.00 0.99 0.96
2.94 1.22 2.94 4.06 3.00
f1 (ppm)

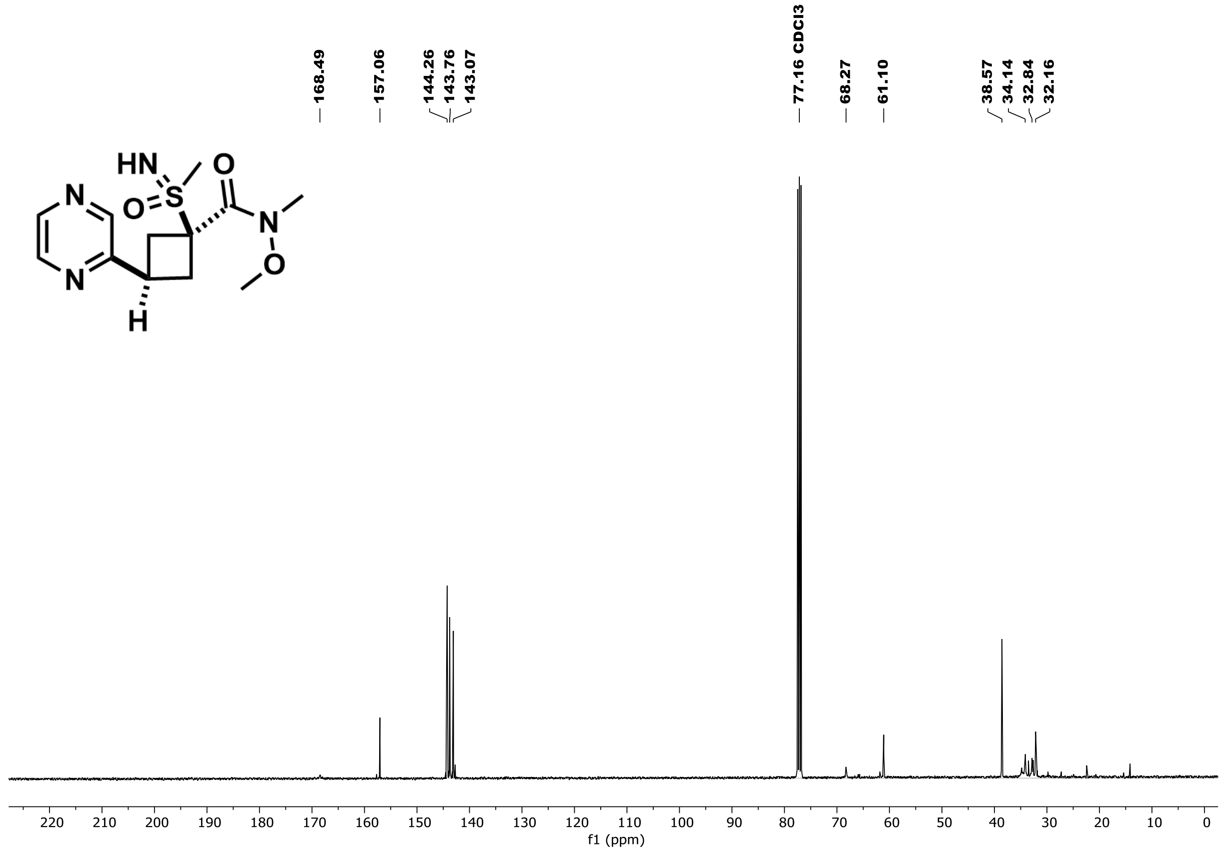
168.49
157.06
144.26 143.76 143.07
77.16 CDCl3
68.27
61.10
38.57 34.14 32.84 32.16
HN
O
S
N
O
N
N
H
f1 (ppm)

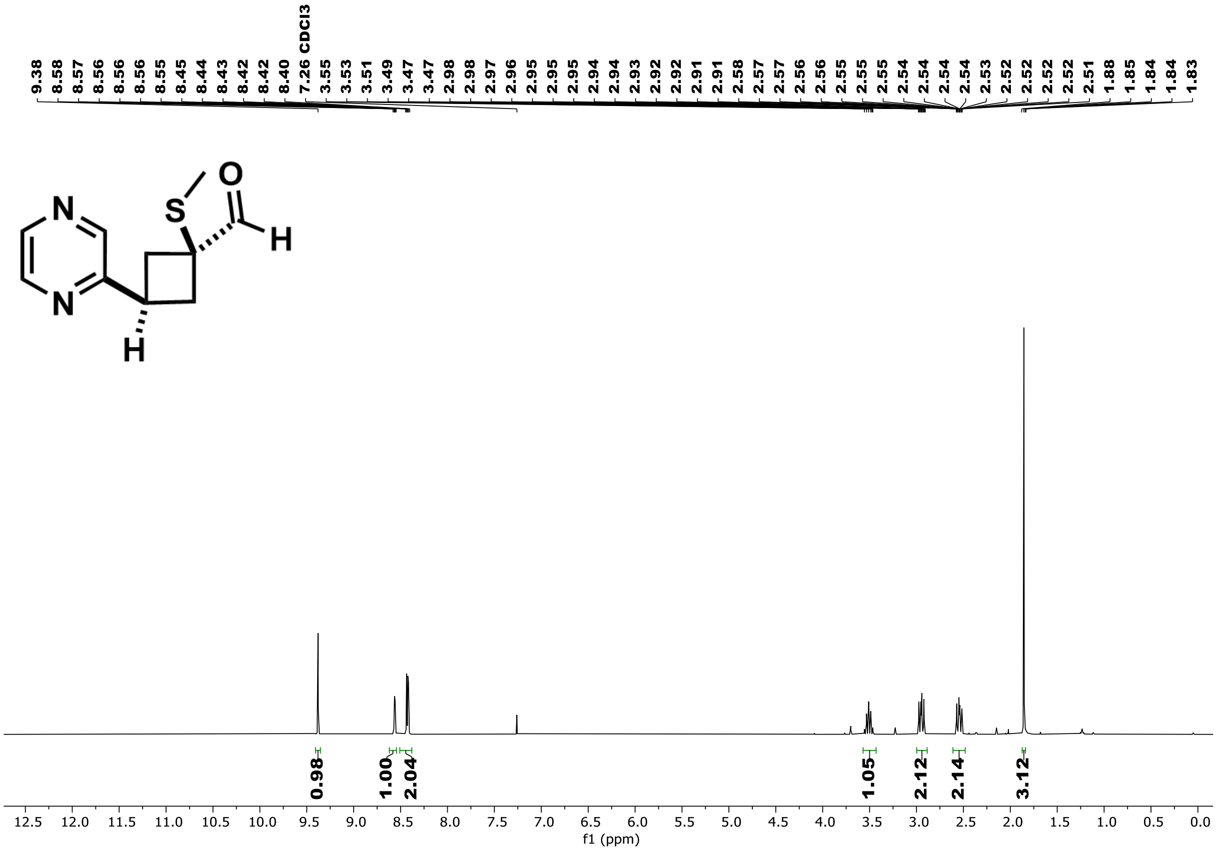
9.38 8.58 8.57 8.56 8.56 8.56 8.55 8.45 8.44 8.43 8.42 8.42 8.40 7.26 CDCl3 3.55 3.53 3.51 3.49 3.47 2.98 2.98 2.97 2.96 2.95 2.95 2.95 2.94 2.94 2.93 2.92 2.92 2.91 2.91 2.58 2.57 2.57 2.56 2.56 2.55 2.55 2.55 2.54 2.54 2.54 2.54 2.53 2.52 2.52 2.52 2.52 2.51 1.88 1.85 1.84 1.84 1.83
O
S
H
N
N
H
0.98
1.00
2.04
1.05
2.12
2.14
3.12
12.5 12.0 11.5 11.0 10.5 10.0 9.5 9.0 8.5 8.0 7.5 7.0 6.5 6.0 5.5 5.0 4.5 4.0 3.5 3.0 2.5 2.0 1.5 1.0 0.5 0.0
f1 (ppm)

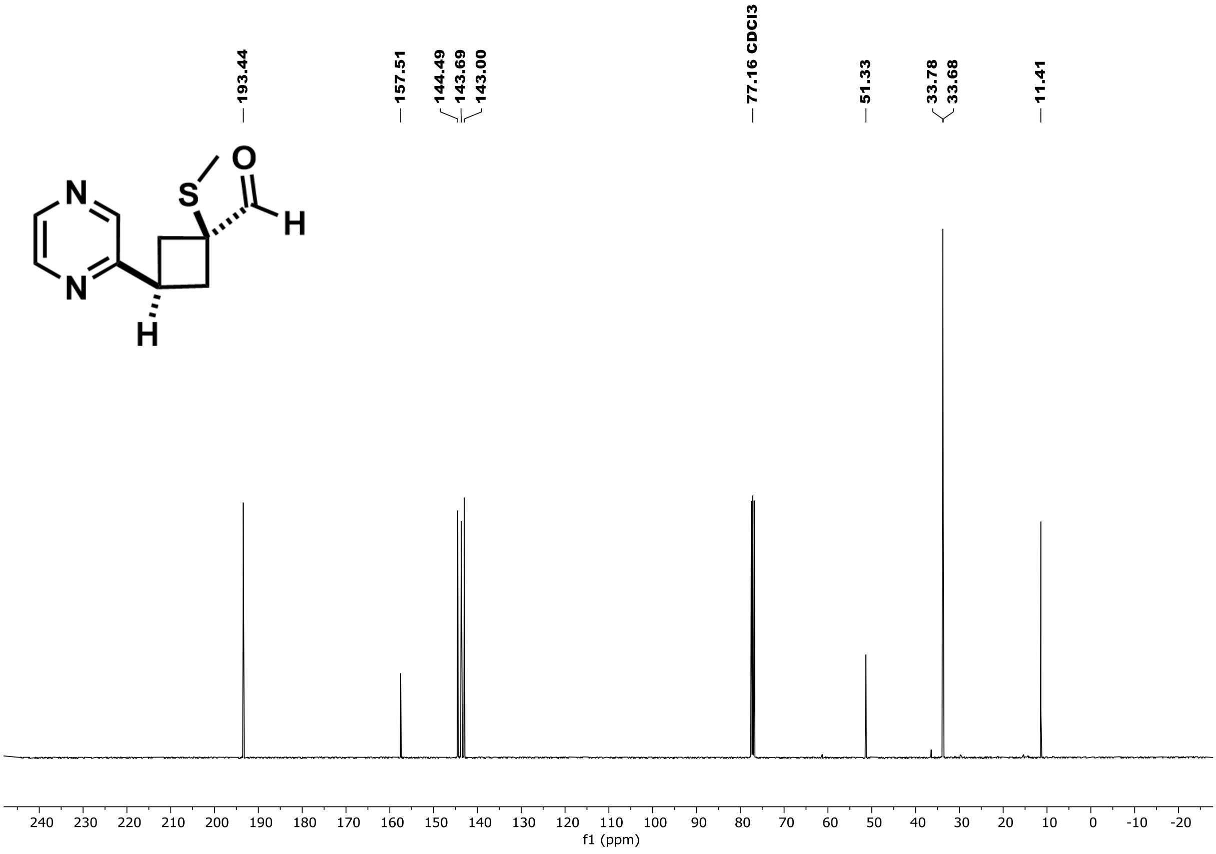
193.44
157.51
144.49 143.69 143.00
77.16 CDCl3
51.33
33.78 33.68
11.41
O
S
H
N
N
H
240 230 220 210 200 190 180 170 160 150 140 130 120 110 100 90 80 70 60 50 40 30 20 10 0 -10 -20
f1 (ppm)

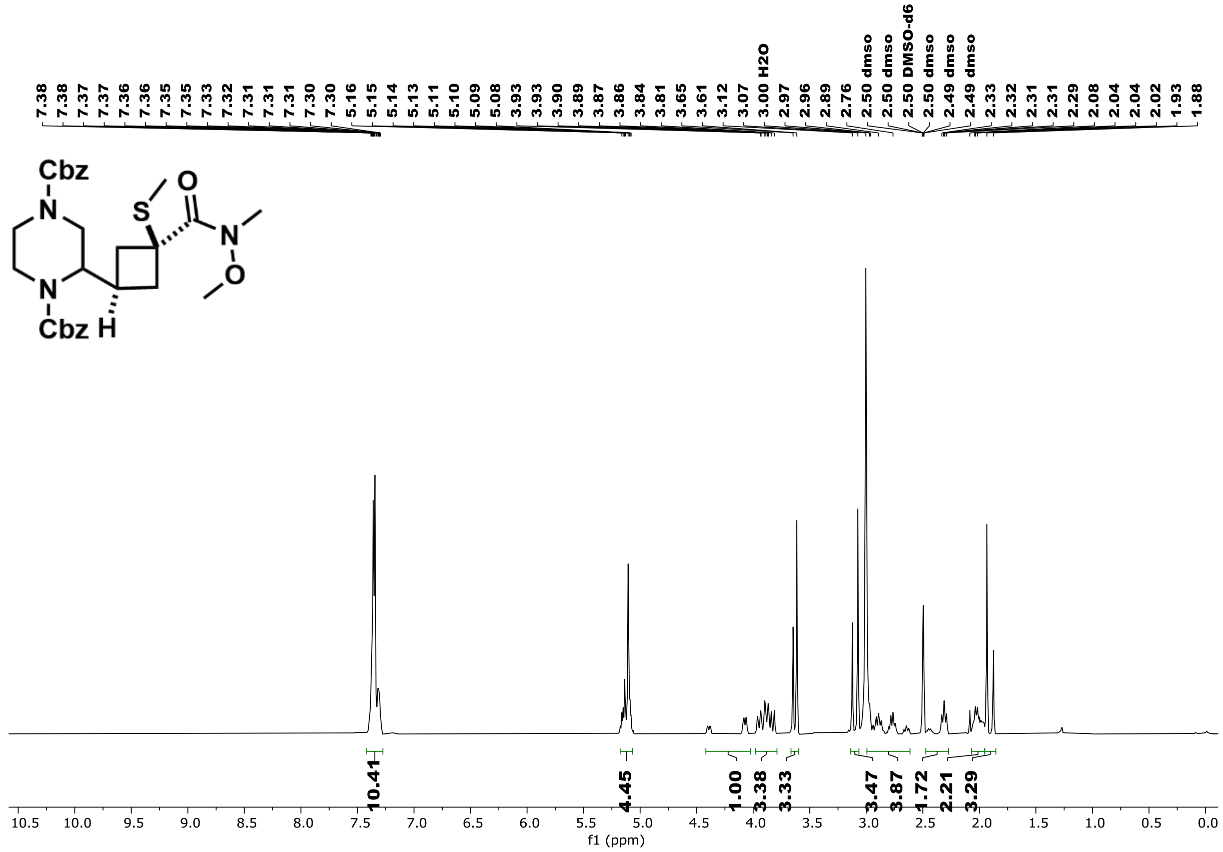

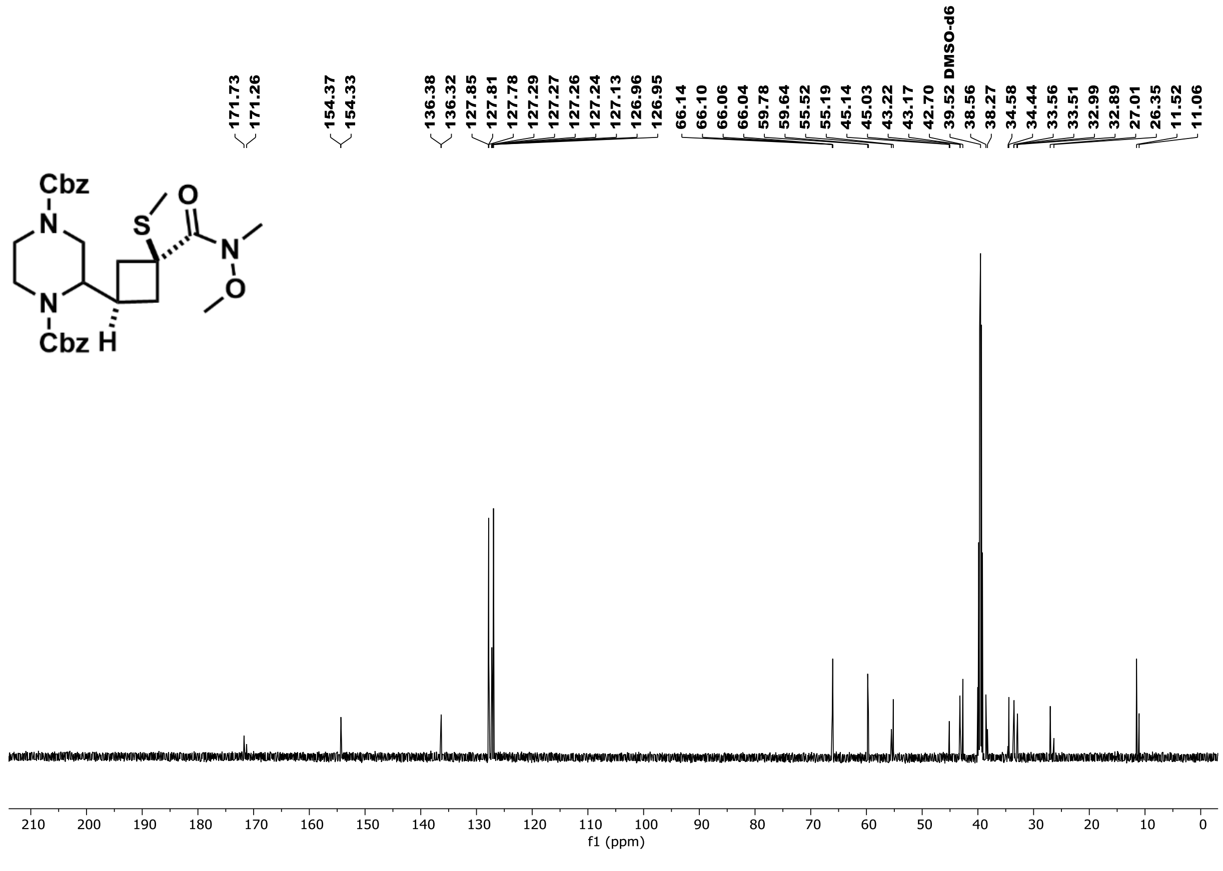

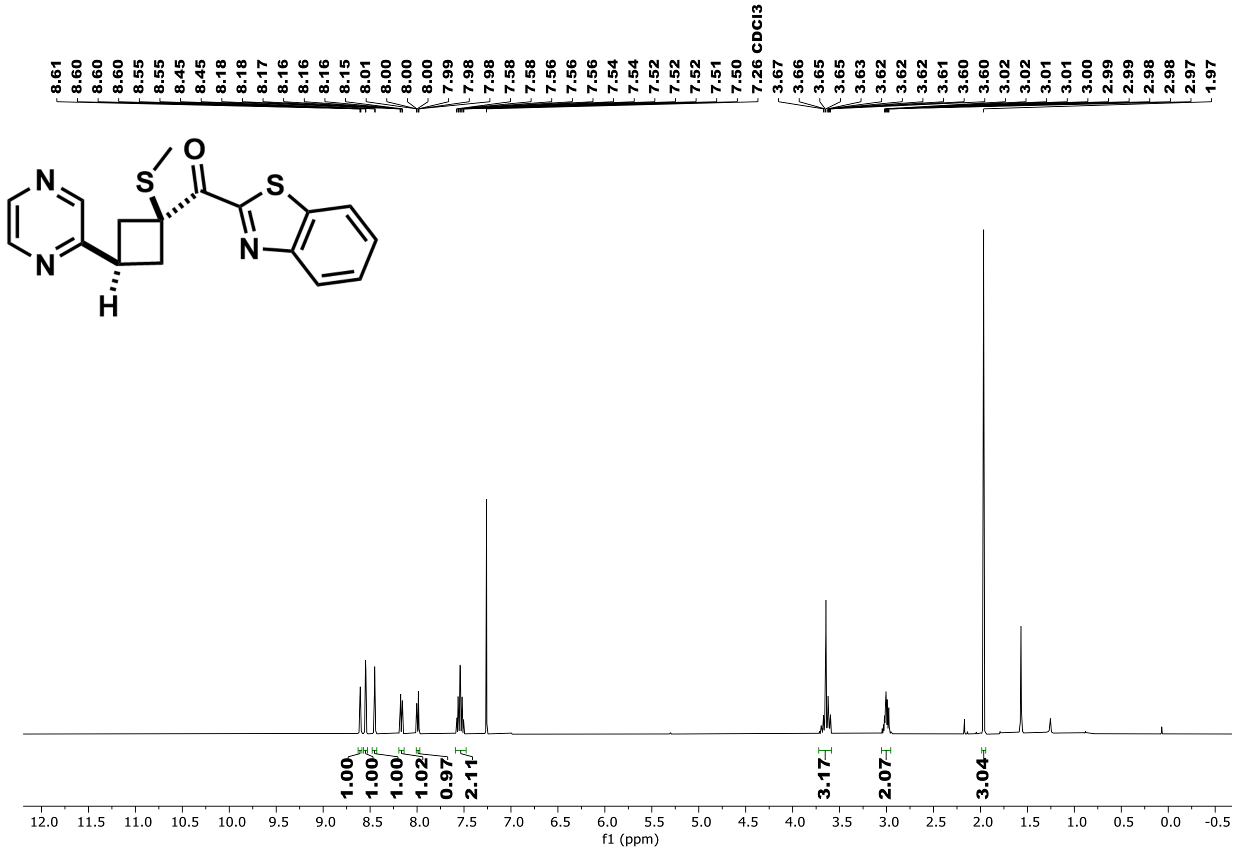
8.61 8.60 8.60 8.60 8.55 8.55 8.45 8.45 8.18 8.17 8.16 8.16 8.16 8.15 8.01 8.00 8.00 7.99 7.98 7.98 7.58 7.58 7.56 7.56 7.56 7.54 7.54 7.52 7.52 7.52 7.51 7.50 7.26 CDCl3 3.67 3.66 3.65 3.65 3.63 3.62 3.62 3.62 3.61 3.60 3.60 3.02 3.02 3.01 3.01 3.00 2.99 2.99 2.98 2.98 2.97 1.97
1.00 1.00 1.00 1.02 0.97 2.11 3.17 2.07 3.04
f1 (ppm)

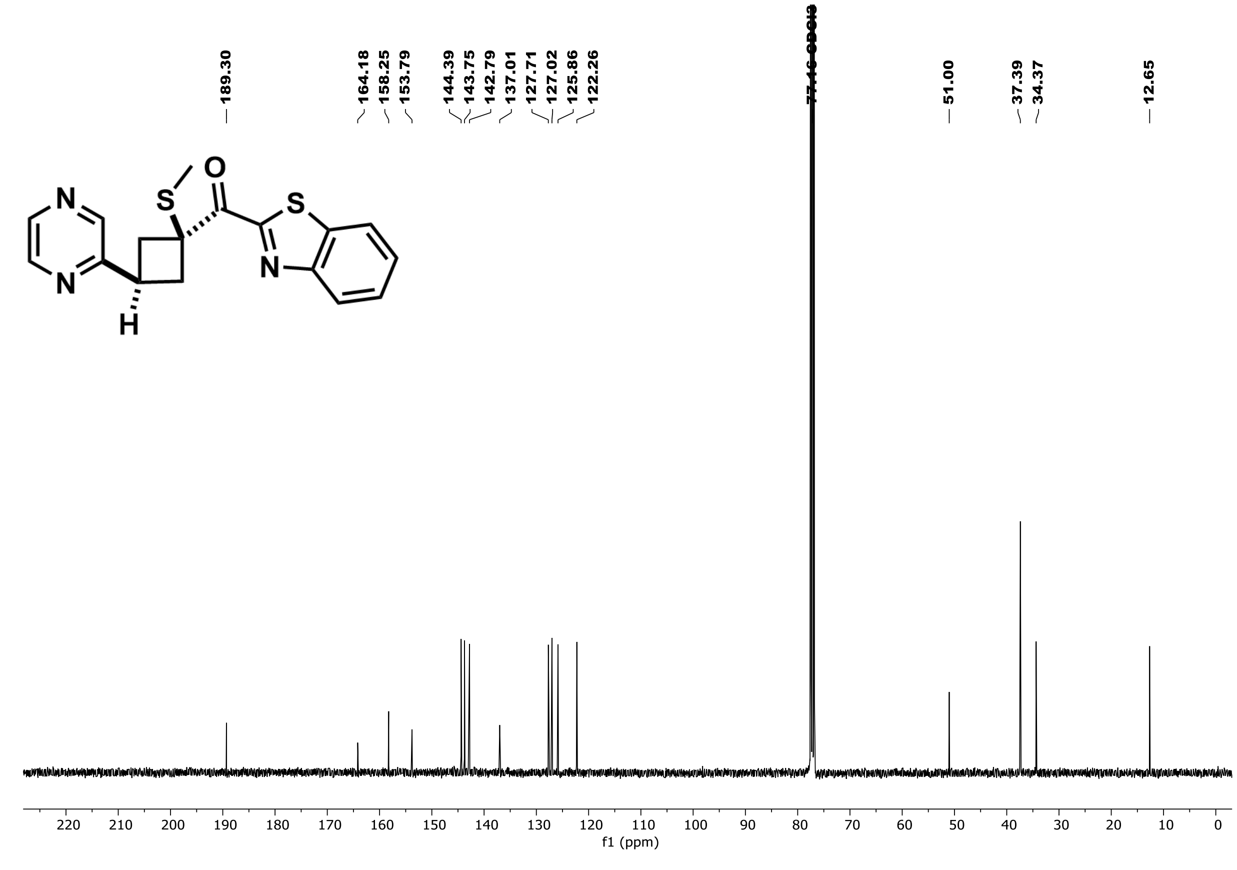
189.30
164.18 158.25 153.79 144.39 143.75 142.79 137.01 127.71 127.02 125.86 122.26
77.16 CDCl3
51.00 37.39 34.37 12.65
f1 (ppm)

6 References

1. L. Pitzer, F. Sandfort, F. Strieth-Kalthoff, F. Glorius, *Angew. Chem. Int. Ed.* 2018, **57**, 16219.
2. V. A. Pistritto, M. E. Schutzbach-Horton, D. A. Nicewicz, *J. Am. Chem. Soc.* 2020, **142**, 17187.
3. X. A. F. Cook, L. R. E. Pantaine, D. C. Blakemore, I. B. Moses, N. W. Sach, A. Shavnya, M. C. Willis, *Angew. Chem. Int. Ed.* 2021, **60**, 22461.
4. H. Yorimitsu, H. Yamagishi, S. Tsuchiya, H. Saito, K. Nogi, J. Shimokawa, *Heterocycles* 2019, **99**, 301.
5. F. Danton, M. Othman, A. M. Lawson, J. Moncol, A. Ghinet, B. Rigo, A. Daïch, *Chemistry* 2019, **25**, 6113.
6. J. Yang, S. Liu, J.-F. Zheng, J. Zhou, *Eur. J. Org. Chem.* 2012, **2012**, 6248.
7. Z. Lian, B. Morandi, B. N. Bhawal, P. Yu, T. Delcaillau, WO2018162364A1, 2018.
8. J. S. Dhau, A. Singh, A. Singh, R. Dhir, P. Brandão, V. Félix, *Inorg. Chim. Acta* 2014, **421**, 359.
9. S. Ruf, M. S. Hallur, N. K. Anchan, I. N. Swamy, K. R. Murugesan, S. Sarkar, L. K. Narasimhulu, V. P. R. K. Putta, S. Shaik, D. V. Chandrasekar, *Bioorg. Med. Chem. Lett.* 2018, **28**, 922.
10. A. Rioz-Martínez, G. de Gonzalo, D. E. T. Pazmiño, M. W. Fraaije, V. Gotor, *Eur. J. Org. Chem.* 2010, **2010**, 6409.
11. M. Chen, H. Huang, K. Wu, Y. Liu, L. Jiang, Y. Li, G. Tang, J. Peng, X. Cao, *Drug Dev. Res.* 2022, **83**, 55.
12. K. Nakamoto, Y. Ueno, *J. Org. Chem.* 2014, **79**, 2463.
13. X. Ma, J. Yu, R. Yan, M. Yan, Q. Xu, *J. Org. Chem.* 2019, **84**, 11294.
14. V. Alcolea, D. Plano, I. Encío, J. A. Palop, A. K. Sharma, C. Sanmartín, *Eur. J. Med. Chem.* 2016, **123**, 407.
15. C. Dai, Z. Xu, F. Huang, Z. Yu, Y.-F. Gao, *J. Org. Chem.* 2012, **77**, 4414.
16. K.-C. Tang, J. Cao, L. M. Boatner, L. Li, J. Farhi, K. N. Houk, J. Spangle, K. M. Backus, M. Raj, *Angew. Chem. Int. Ed.* 2022, **61**, e202112107.
17. R. Guo, Y.-C. Chang, L. Herter, C. Salome, S. E. Braley, T. C. Fessard, M. K. Brown, *J. Am. Chem. Soc.* 2022, **144**, 7988.
18. R. Kleinmans, T. Pinkert, S. Dutta, T. O. Paulisch, H. Keum, C. G. Daniliuc, F. Glorius, *Nature* 2022, **605**, 477.
19. S. Agasti, F. Beltran, E. Pye, N. Kaltsoyannis, G. E. M. Crisenza, D. J. Procter, *Nat. Chem.* 2023, **15**, 535.
20. T. Pinkert, M. Das, M. L. Schrader, F. Glorius, *J. Am. Chem. Soc.* 2021, **143**, 7648.
21. S. Dutta, Y.-L. Lu, J. E. Erchinger, H. Shao, E. Studer, F. Schäfer, H. Wang, D. Rana, C. G. Daniliuc, K. N. Houk, *J. Am. Chem. Soc.* 2024, **146**, 5232.
22. M. A. A. Walczak, P. Wipf, *J. Am. Chem. Soc.* 2008, **130**, 6924.
23. S. Dutta, D. Lee, K. Ozols, C. G. Daniliuc, R. Shintani, F. Glorius, *J. Am. Chem. Soc.* 2024, **146**, 2789.
24. J. L. Tyler, F. Schäfer, H. Shao, C. Stein, A. Wong, C. G. Daniliuc, K. N. Houk, F. Glorius, *J. Am. Chem. Soc.* 2024, **146**, 16237.
25. X. Ma, D. L. Sloman, Y. Han, D. J. Bennett, *Org. Lett.* 2019, **21**, 7199.
26. S.-L. Lin, Y.-H. Chen, H.-H. Liu, S.-H. Xiang, B. Tan, *J. Am. Chem. Soc.* 2023, **145**, 21152.
27. R. Kleinmans, S. Dutta, K. Ozols, H. Shao, F. Schäfer, R. E. Thielemann, H. T. Chan, C. G. Daniliuc, K. N. Houk, F. Glorius, *J. Am. Chem. Soc.* 2023, **145**, 12324.
28. J. M. Lopchuk, K. Fjelbye, Y. Kawamata, L. R. Malins, C.-M. Pan, R. Gianatassio, J. Wang, L. Prieto, J. Bradow, T. A. Brandt, *J. Am. Chem. Soc.* 2017, **139**, 3209.

29 L. Guo, A. Noble, V. K. Aggarwal, *Angew. Chem. Int. Ed.* 2021, **133**, 214.
30 M. Ociepa, A. J. Wierzba, J. Turkowska, D. Gryko, *J. Am. Chem. Soc.* 2020, **142**, 5355.
31 J.-F. Lohier, T. Glachet, H. Marzag, A.-C. Gaumont, V. Reboul, *Chem. Commun.* 2017, **53**, 2064.
32 L. Pitzer, F. Schäfers, F. Glorius, *Angew. Chem. Int. Ed.* 2019, **58**, 8572.
33 K. D. Collins, F. Glorius, *Nat. Chem.* 2013, **5**, 597.
34 J. R. Aranzaes, M.-C. Daniel, D. Astruc, *Can. J. Chem.* 2006, **84**, 288.
35 M. A. Cismesia, T. P. Yoon, *Chem. Sci.* 2015, **6**, 5426.
36 a) C. Lee, W. Yang, R. G. Parr, *Phys. Rev. B* 1988, **37**, 785; b) A. D. Becke, *J. Chem. Phys.* 1993, **98**, 5648.
37 S. Grimme, *J. Comput. Chem.* 2004, **25**, 1463.
38 S. Grimme, J. Antony, S. Ehrlich, H. Krieg, *J. Chem. Phys.* 2010, **132**, 154104.
39 S. Grimme, *WIREs Comput. Mol. Sci.* 2011, **1**, 211.
40 S. Ehrlich, J. Moellmann, S. Grimme, *Acc. Chem. Res.* 2013, **46**, 916.
41 F. Weigend, R. Ahlrichs, *Phys. Chem. Chem. Phys.* 2005, **7**, 3297.
42 F. Weigend, *Phys. Chem. Chem. Phys.* 2006, **8**, 1057.
43 Gaussian, *Gaussian 16, revision C.01*, Gaussian Inc., 2019.
44 a) C. Y. Legault, *CYLview20*, Université de Sherbrooke, 2020; b) W. Humphrey, A. Dalke, K. Schulten, *J. Mol. Graph.* 1996, **14**, 33-8, 27-8.
45 O. López-Estrada, H. G. Laguna, C. Barrueta-Flores, C. Amador-Bedolla, *ACS Omega* 2018, **3**, 2130.
46 F. M. Bickelhaupt, K. N. Houk, *Angew. Chem. Int. Ed.* 2017, **56**, 10070.
